# Intermolecular Reductive Coupling of Ketones and Alkenes under Ferrioxalate Photocatalysis

**DOI:** 10.1021/jacs.6c10799

**Published:** 2026-07-13

**Authors:** Dalila Arnaldi, Niccolò Intini, Partha Pratim Sen, Sergio Adalid, Kristýna Kellovská, Michael G. Guerzoni, Thomas D. Svejstrup, Fabio Juliá

**Affiliations:** † Facultad de Química, Centro de Investigación Multidisciplinar Pleiades-Vitalis, 16751 Universidad de Murcia; Campus de Espinardo, 30100 Murcia, Spain; ‡ Early Chemical Development, Pharmaceutical Sciences, R&D, AstraZeneca Pharmaceuticals, Gothenburg 43183, Sweden

## Abstract

Ketones and alkenes are among the most frequent functional groups found in natural products, feedstock, and bioactive compounds. Hence, the use of both functionalities as cross-coupling handles retains a marked synthetic potential for Csp^3^–Csp^3^ bond formation. However, the reaction between ketones and alkenes has traditionally relied on intramolecular systems or the use of activated coupling partners, while intermolecular reactions involving unbiased substrates have found limited success. Here we report a strategy for the direct coupling of unactivated ketones with unactivated alkenes enabled by ferrioxalate photocatalysis. This catalytic platform allows the inner-sphere reduction of ketones to access ketyl radicals that effectively react with alkenes despite the adverse polarity mismatch. This method gives access to a wide range of tertiary alcohols, which are generally assembled through Grignard reagent additions, using alkenes in place of organometallic nucleophiles, allowing this transformation to proceed with broad tolerance to diverse polar and protic functional groups. Overall, this reactivity blueprint constitutes a platform for the fast exploration of Csp^3^-rich chemical space, capitalizing on the diversity of readily available alkene and ketone building blocks. The utility of the method is demonstrated in several applications, showcasing the enabling potential of this platform for synthetic and medicinal chemistry.

## Graphical abstract

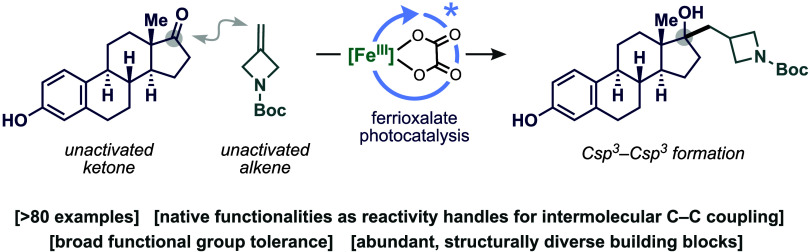

## Introduction

The inclusion of tertiary alcohol motifs in drug candidates constitutes a useful strategy in drug design because it induces desirable effects on permeability, solubility, or pharmacokinetics, among others, while avoiding typical pitfalls of lower-substituted alcohols, which present reactive sites for oxidative metabolic degradation (Figure 1a).^1^ When approaching their synthesis, tertiary alcohols are generally accessed by the addition of organomagnesium Grignard reagents to ketones. The Grignard reaction is a textbook transformation that is also largely used due to its utility to form new C–C bonds in a straightforward manner.^2^ However, the use of these air-sensitive organometallic reagents can be problematic due to their incompatibility with many functional groups, particularly those protic or polar, compelling protection–deprotection sequences that result in lengthy synthetic routes. Moreover, the use of alkyl nucleophiles poses an additional challenge because these reagents are more difficult to access through halogen–magnesium exchange reactions in comparison to aryl or alkenyl counterparts.^3^ Conversely, the use of alkenes in place of Grignard reagents as coupling partners would constitute a functional group-tolerant, streamlined approach to access tertiary alcohols (Figure 1b). Notably, such a desirable synthetic disconnection would enable the fast exploration of chemical space provided by the vast and diverse pool of commercially available ketone (>7.7 million compounds) and alkene (>2.4 million compounds) building blocks (Figure S2). Moreover, in view of the ubiquity of these motifs in bioactive compounds and natural products^4–6^ — ^4–6^ 40% of natural products contain alkenes, 16% contain ketones—this strategy would pose an interesting tool to adopt the coupling of these structurally complex Csp^3^-rich fragments, a desirable feature for medicinal chemistry.^7–9^ However, the development of a direct coupling between alkenes and ketones is not a trivial task. To achieve this goal, several approaches have emerged over the years,^10–13^ with some of the most successful examples relying on radical chemistry (Figure 1c). Historically, the use of stoichiometric SmI_2_ has dominated the field,^14^ due to its ability to reduce carbonyls via inner-sphere single-electron transfer (SET) to access ketyl radicals that present umpolung reactivity with respect to the parent carbonyl compounds.^15,16^ While this approach has been extraordinarily useful in intramolecular settings,^17^ the nucleophilic character of ketyl radicals hinders their addition to unactivated alkenes due to a polarity mismatch.^18,19^ Therefore, intermolecular couplings generally rely on the use of activated alkenes (electron-deficient alkenes, styrene derivatives) which present favorable polar or enthalpic effects.^20–22^ More recently, the development of highly reductive systems based on photoredox catalysis has enabled access to ketyl radicals by SET reduction even for alkyl ketones (*E*_red_ < −2.5 V vs SCE); however, these methods display similar limitations on the scope of suitable alkenes as coupling partners,^23–28^ with a notable exception reported during the preparation of this manuscript.^29^ Alternative approaches based on metal hydride hydrogen atom transfer (MHAT)^13^ can also mediate couplings between alkenes and carbonyls, although presenting Markovnikov regioselectivity and similarly precluding intermolecular reactions with ketones.^30–32^ Recently, Baran and co-workers developed the first direct method to couple unactivated ketones and unactivated alkenes.^33^ This elegant electrochemical method provides access to an array of couplings between ketones and terminal alkenes as limiting reagents, observing an enhanced performance of homoallylic alcohol substrates due to directing effects associated with their binding to the electrode surface. These efforts underscore the challenge of using unactivated alkenes and ketones as coupling partners for intermolecular C–C bond formation reactions irrespective of the regioselectivity or the reactivity manifold, rendering the development of a broadly applicable method a longstanding ambition.

**Figure 1:**
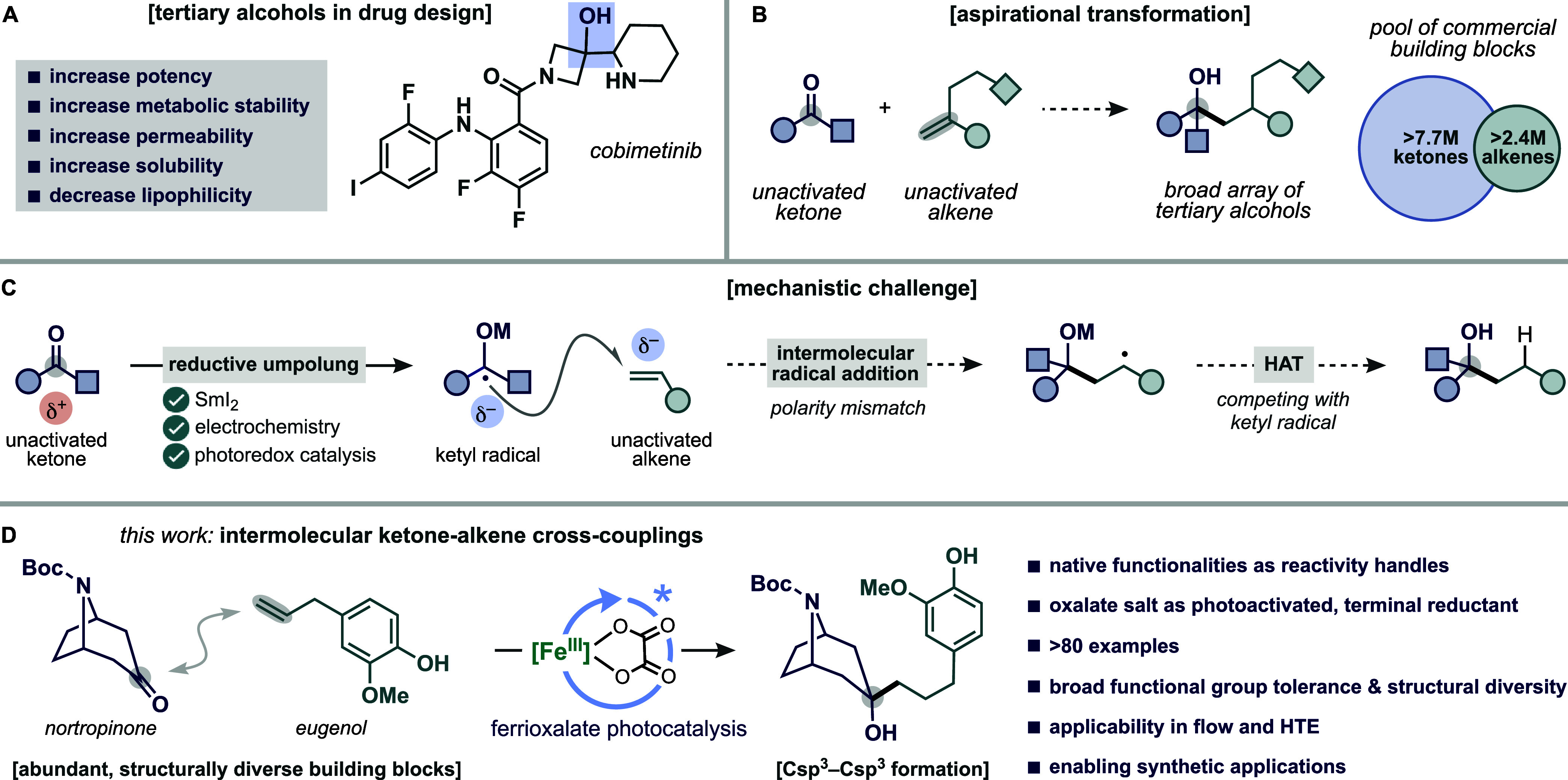
Relevance and overview of ketone–alkene coupling. (a) Tertiary alcohol motifs in drug discovery. (b) Synthetic logic for Csp^3^–Csp^3^ bond formation via cross-coupling of readily available ketone and alkene substrates. (c) Mechanistic challenges associated with the coupling of unactivated substrates under radical mechanisms. (d) This work: ferrioxalate photocatalysis enabling the intermolecular cross-coupling of unactivated ketones and alkenes.

We recently unveiled the use of ferrioxalate photocatalysis to promote several synthetic transformations harnessing oxalate salts as stoichiometric reductants upon ligand-to-metal charge transfer (LMCT) excitation.^34^ This strategy, inspired by the classic photochemistry of ferrioxalate,^35,36^ was leveraged to access versatile and highly reducing low-valent Fe species that are reactive toward aryl halides and electron-deficient alkenes. However, the reactivity profile of this catalytic system remains essentially unexplored, offering exciting opportunities for reaction discovery. In this work, we introduce the application of this concept to enable the broad intermolecular coupling of unactivated ketones and unactivated alkenes (Figure 1d). This strategy represents a useful catalytic approach to this transformation and benefits from the wide tolerance of ferrioxalate photocatalysis to many polar and protic groups, which constitutes a key conceptual advantage for approaching target compounds otherwise inaccessible with routes based on Grignard reagents.

## Results and Discussion

### Reaction Design, Development, and Scope

At the outset, we wondered whether ferrioxalate photocatalysis could constitute a new avenue to generate ketyl radicals via inner-sphere activation, mirroring classical SmI_2_ chemistry. This notion was based on precedents reporting this type of reactivity for low-valent Fe­(I) species with carbonyl groups.^37,38^ With this idea in mind, we hypothesized a mechanistic scenario for the coupling between ketones and alkenes under iron catalysis, which is depicted in Figure 2a. Under light excitation, Fe­(III) oxalate **I** undergoes LMCT reactivity,^39^ resulting in the overall oxidation of oxalate to carbon dioxide (C_2_O_4_^2–^ → 2CO_2_ + 2e^–^) with concomitant 2-electron reduction of the metal center, leading to a formal Fe­(I) species (**II**).^34,40,41^ This species would then undergo inner-sphere activation of ketone substrates to generate a Fe-bound ketyl radical (**III**).^37,38^ The addition of the ketyl radical to the alkene would result in an alkyl radical that should be rapidly intercepted by the nearby Fe­(II) center, leading to the rebound alkyl Fe­(III) intermediate **IV**. We conjectured that this path would provide a framework to overcome the polarity mismatch between ketyl radicals and unactivated alkenes, expediting this challenging radical addition through the formation of a stabilized intermediate. Hydrolysis of **IV** ^34^ would then lead to the formation of the desired product **V**, allowing catalytic turnover after ligand exchange on **VI**.

**Figure 2:**
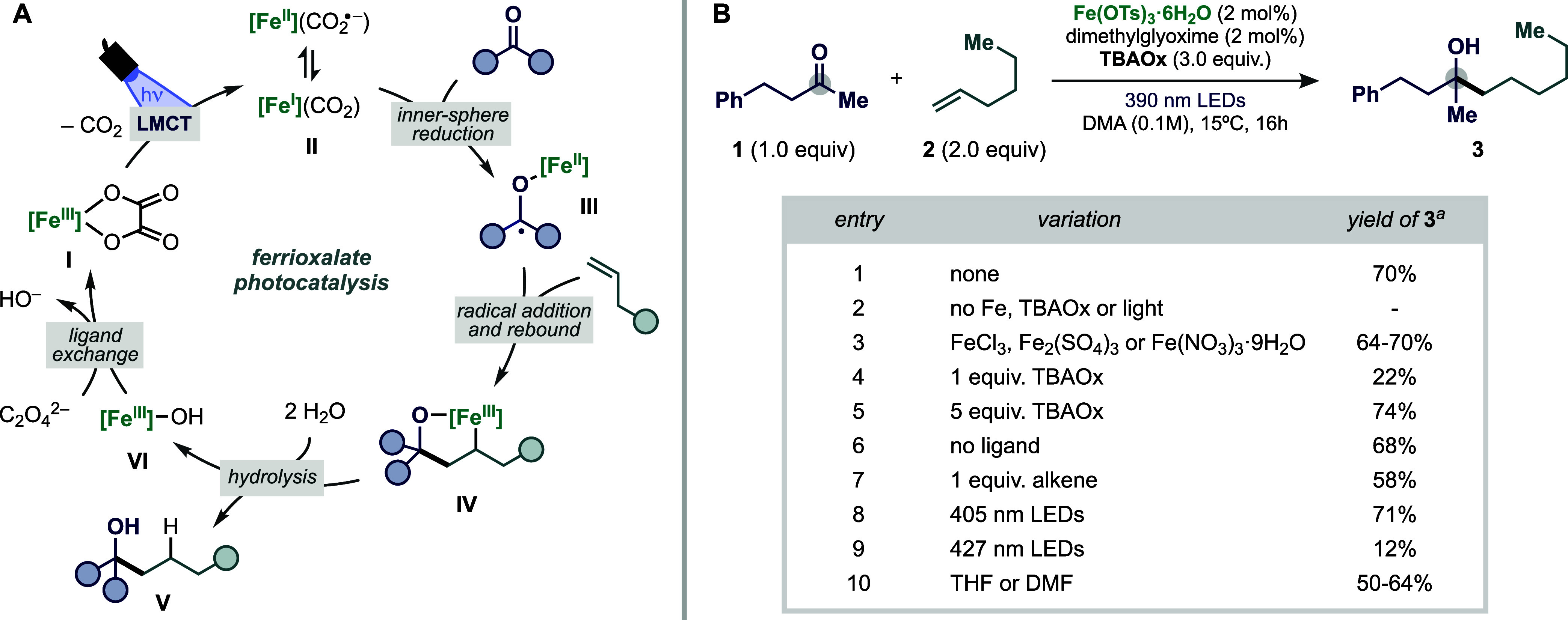
Design and development of the method. (a) Initial mechanistic hypothesis. (b) Optimized conditions and deviations. ^*a*^Yields were determined by HPLC analysis.

To begin our study, we chose ketone **1** and alkene **2** as model compounds for the intermolecular reductive coupling between unbiased substrates en route to tertiary alcohols (Figure 2b). After some optimization, we found this reaction possible in the presence of Fe­(OTs)_3_·6H_2_O as the catalyst, dimethylglyoxime as the ligand, and tetrabutylammonium oxalate (TBAOx) as the stoichiometric reductant under 390 nm irradiation in dimethylacetamide (DMA) solvent. Under these optimized conditions, the desired coupling product **3** was obtained in 70% yield (entry 1). Control experiments confirmed the need for the iron catalyst, oxalate, and light to achieve observable conversion to the product (entry 2). While different Fe­(III) salts can be employed, leading to comparable results (entry 3), we found the use of the nonhygroscopic salt Fe­(OTs)_3_·6H_2_O more practical. The amount of TBAOx (entries 4–5) can be important to achieve effective reactions, and the use of 5 equiv is beneficial in cases where low conversion was found. While the reaction can operate in the absence of added ligands (entry 6), we found the use of dimethylglyoxime gave slightly improved and more reproducible results across different substrates. Although the reaction performs better if the alkene is present in 2-fold excess, it can be carried out using equimolar amounts of both coupling partners with productive yields (entry 7). Finally, other solvents and light sources were found to be valid, albeit in lower yields (entries 8–10). Mechanistic experiments support the inner-sphere reduction of ketones to generate ketyl radicals and the involvement of alkyl Fe intermediates under these conditions in line with our mechanistic hypothesis (see Section 13 in the Supporting Information), representing a conceptually new platform to address this challenging transformation.

The robustness of the protocol was then assessed, presenting low sensitivity to concentration, humidity, temperature, or air exposure, among others (Figure S5). Moreover, the reproducibility of the protocol was independently demonstrated at AstraZeneca’s laboratories, where a model reaction could be reproduced in 70% yield using a different setup, batches of the reagents, and practitioners in comparison with the original reaction (see Section 7.3 in the Supporting Information). In view of the possible benefits of providing a Grignard-free route for tertiary alcohols, the sensitivity of the reaction toward the addition of different additives was evaluated (Figure S6). The model reaction displayed good tolerance to the addition of many common polar and protic functionalities, including >75% of the top-30 functional groups contained in bioactive compounds,^5^ and 80% of the top-10 (hetero)­aromatic motifs found in marketed drugs,^42^ paving the way for the feasible implementation of this method in medicinal chemistry.

With the optimized reactions in hand, we sought to explore the scope of this methodology (Figure 3). A range of different terminal alkenes were first tested, showcasing the performance of the methodology in the presence of a broad range of functionalities such as arene, alcohol, ester, amino acid, carboxylic acid, amine, phenol, or hydantoin (**3–11**). Importantly, the presence of heteroarenes such as pyridine, thiophene, or indole, which are ubiquitous in bioactive compounds, is also tolerated (**12–14**). The tolerance of this method to several reducible functionalities underlines the benefits of inner-sphere activation of ketones over other strategies based on outer-sphere SET reduction^29,33^ (Figure S27). Remarkably, reactions with some electron-rich alkenes such as vinyl ethers and vinyl silanes (**15–16**) are also possible under these reaction conditions despite the strong polarity mismatch. The scope of this transformation can be extended beyond terminal alkenes and was found to be highly effective for 1,1-disubstituted alkenes, a class of olefins that can be readily accessed from ketones via Wittig reactions and is present in many feedstock chemicals and natural products. This aspect significantly broadens the structural diversity of the products accessed with this method, which can accommodate exocyclic alkenes embedded into saturated carbocycles and heterocycles of different ring sizes (**17–22**), including spirocyclic structures such as **23**. Likewise, acyclic disubstituted alkenes displayed good performance (**24–26**) as well as drug derivatives **27** and **28**. These examples further showcase the compatibility with functionalities such as epoxide, thioether, and urea. While some internal alkenes can be engaged, this type of substrate typically displays reduced reactivity under these conditions and generally affords lower yields of the desired products, as shown for **29**. The remaining mass balance in those cases is constituted by unreacted starting material or undesired reduction to the alcohol. The scope of ketones was explored next, showing broad compatibility with different Csp^3^ architectures. Cyclic ketones of different ring sizes in the presence of acetal, alcohol, fluorinated carbons, ether, or carbamates can be used (**30–37**), as well as fused aromatic rings (**38**), bicyclic structures (**39–40**), and the natural product dihydrocarvone, which bears an alkene in its backbone (**41**). Acyclic ketones also work well, as demonstrated in **42–44**. Acetone can also be used for this purpose, as shown for products **45–47**, in which the alkene was used as the limiting reagent. Likewise, the applicability of this method for intramolecular couplings was also demonstrated in the assembly of several cyclic scaffolds (see section 9.4 in the Supporting Information).

**Figure 3:**
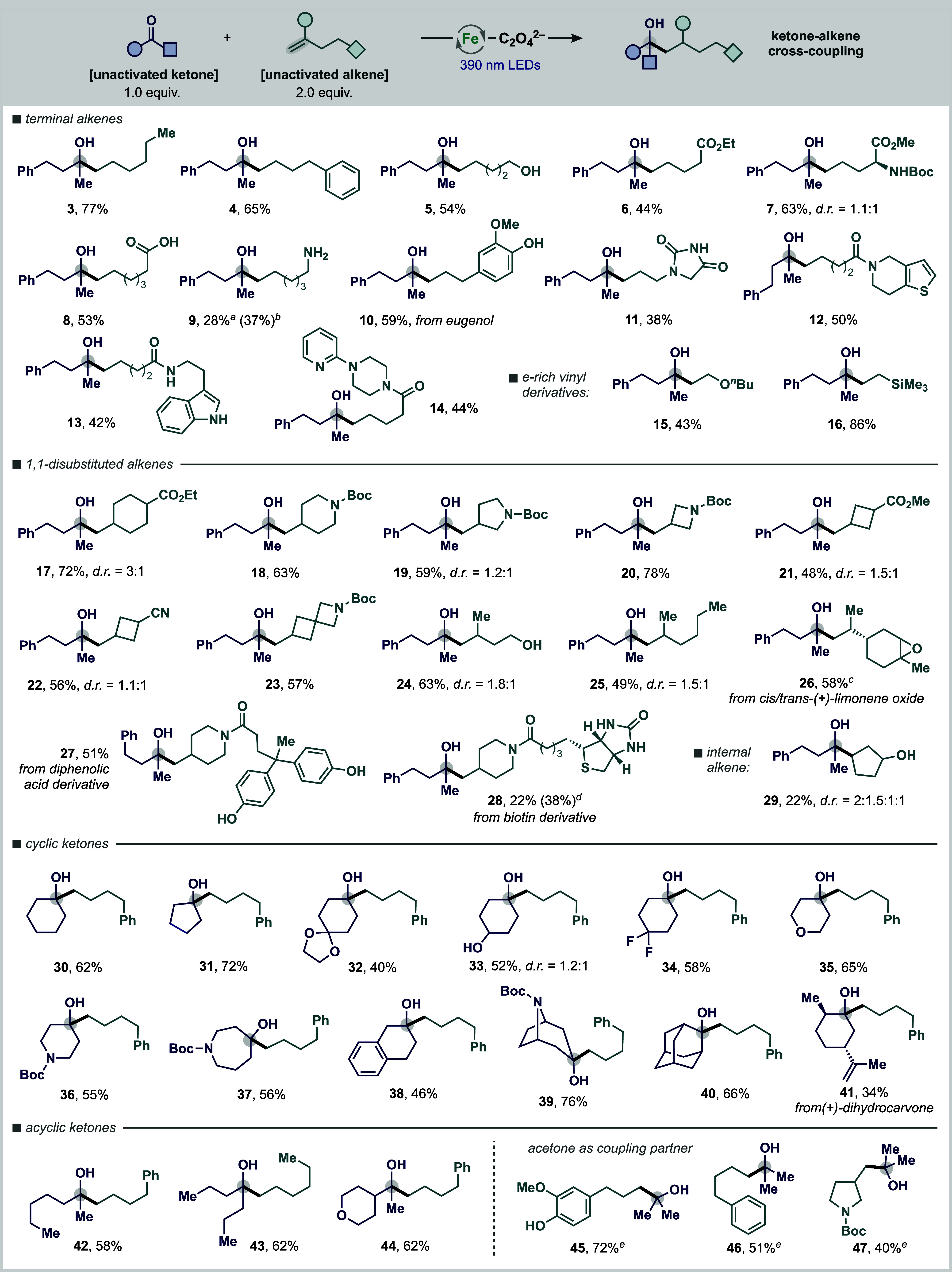
Reaction scope. All yields are isolated. See the SI for detailed information. ^*a*^Isolated after Boc protection. ^*b*^Yield determined by ^1^H NMR analysis. ^*c*^Mixture of diastereomers. ^*d*^Yield determined by HPLC analysis. ^*e*^Alkene was used as limiting reagent.

### Synthetic Applications

In view of the good performance of the method for the radical cross-coupling of alkenes and ketones, we next focused on implementing this strategy in selected applications which might open new synthetic opportunities. Increasing the Fsp^3^ character in drug molecules is a widespread strategy in medicinal chemistry associated with the desirable features offered by three-dimensional scaffolds.^43^ In this context, a method to assemble Csp^3^–Csp^3^ bonds from structurally diverse fragments would constitute a valuable tool to explore 3D chemical space. With this idea in mind, we sought to demonstrate the validity of ferrioxalate catalysis for fragment coupling, creating chemical diversity through “mix and match” of different building blocks (Figure 4a). This blueprint allows exploing the structurally rich pool of terpene chemical feedstocks to introduce sp^3^-rich fragments (**48**). In addition, the coupling between two natural products such as the alkaloid derivative nortropinone and the essential oil eugenol can be carried out in an effective manner (**49**). This chemistry is also suitable for constructing complex polycyclic building blocks that can be further elaborated and would be difficult to access otherwise (**50**). Radical-polar crossover cascades can also be exploited as demonstrated by the radical addition and subsequent lactonization in **51**, which provides an effective route toward γ-lactones, a common chemotype in fragrance chemistry.^44^ Finally, the applicability of this methodology for the late-stage functionalization of pharmaceuticals was demonstrated for the sedative apronalide and the anti-inflammatory drug nabumetone (**52–53**).

**Figure 4:**
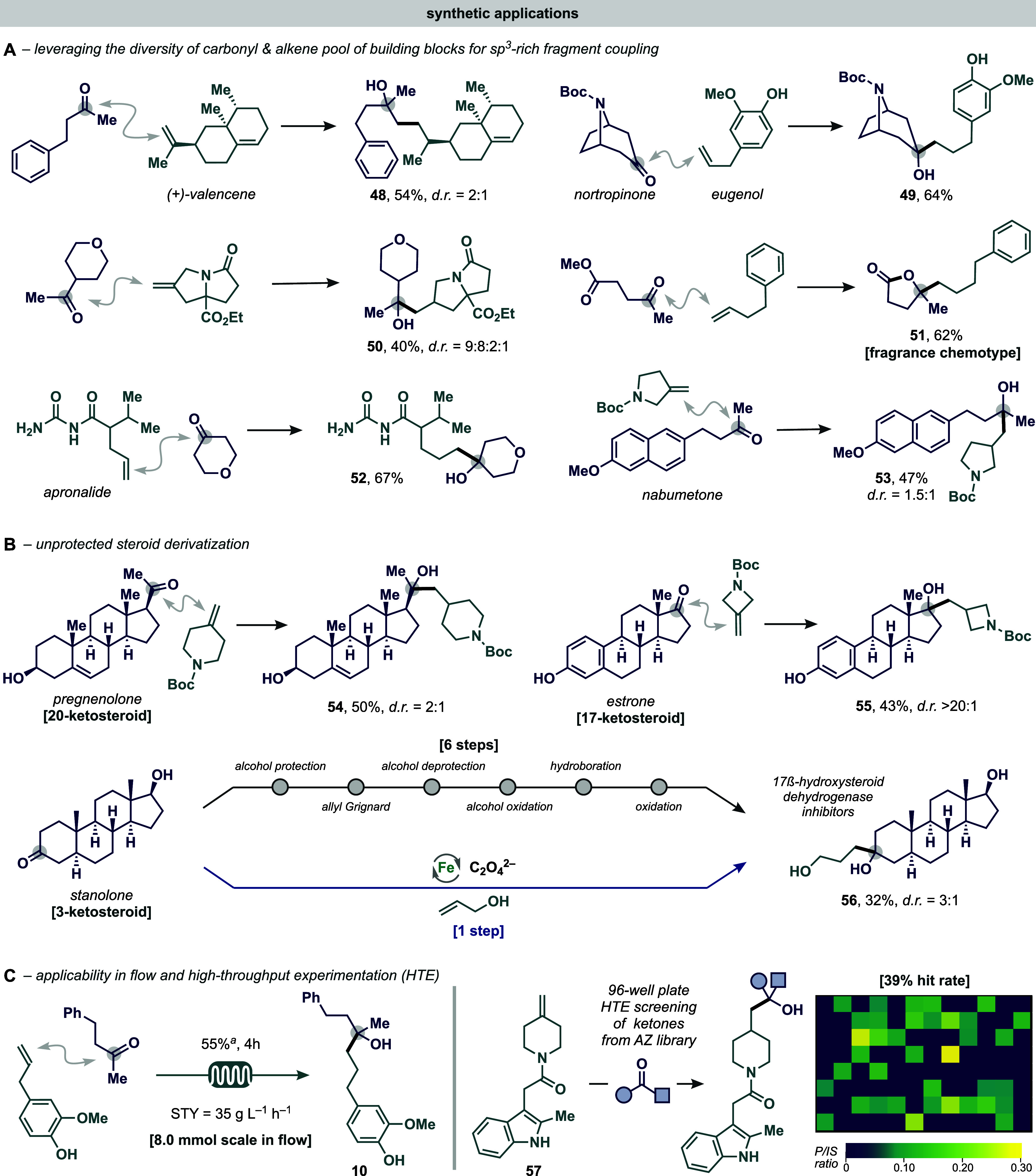
(A–C) Synthetic applications. All yields are isolated. See the SI for detailed information. ^*a*^Yield determined by ^1^H NMR analysis.

Steroids are an important class of bioactive compounds with multiple biological roles and functions.^45^ As such, the derivatization of naturally occurring skeletons has resulted in compounds with pharmacological interest in the treatment of multiple diseases. However, the synthetic elaboration of steroid precursors often suffers from unproductive protection–deprotection sequences. In this regard, we proposed the implementation of our method as a useful way to approach this aim, bypassing the key limitations associated with the use of Grignard reagents such as their incompatibility with functional groups and the lack of diverse organometallic reagents. Thus, we subjected three types of unprotected ketosteroids containing a carbonyl group at position C3 (stanolone), C17 (estrone) and C20 (pregnelone) to our protocol for ketone–alkene coupling (Figure 4b). To our delight, reactions between pregnelone and estrone with alkenes containing N-heterocycles worked well, offering vectors for subsequent derivatization (**54–55**). The synthetic advantage of this strategy can be realized in the synthesis of **56**, an intermediate in the synthesis of inhibitors of Type 3 17β-hydroxysteroid dehydrogenases (17β-HSDs).^46^ While the current route for this target involves 6 steps, associated with protection–deprotection sequences and hydroboration-oxidation to access an alcohol in the Grignard-based fragment, our method provides an expeditious route which enables direct access to this compound in just one step by reaction with allyl alcohol.

The discovery of innovative synthetic methodologies creates new industrial opportunities, particularly when applied in conjunction with advanced technologies such as flow chemistry and high-throughput experimentation (HTE).^47^ Hence, we decided to explore the performance of our method for ketone–alkene cross-coupling in these pharmaceutically relevant setups (Figure 4c). The fully homogeneous conditions of our protocol ease the application of this reaction in flow, which was demonstrated for the coupling of ketone **1** and eugenol in good yields at the gram scale at 35g/L/h (see Section 11 in the Supporting Information). In view of the broad applicability of this tactic for Csp^3^–Csp^3^ bond formation, we posited that this methodology would also be suitable for library synthesis, which is often carried out at microscale on multiwell plates using HTE.^48,49^ Thereby, we used alkene **57** for hit identification with 96 structurally diverse ketones from AstraZeneca’s collection, finding the desired coupling product in at least 39% of the cases (see Section 12 in the Supporting Information). Notably, both flow and HTE were implemented without altering the reaction conditions, underscoring the transformation’s robustness. This operational resilience facilitates straightforward adoption at both large scale and miniaturized formats, which are key tools in pharmaceutical chemistry.

### Case Examples on Applicability for Fast Diversification in Drug Discovery

Considering the benefits of the tertiary alcohol motif in medicinal chemistry, new methods to introduce this functionality are desirable, particularly to overcome the reduced compatibility of current methods with functional groups and their suitability to be implemented at a late stage. In this regard, a method that allows the rapid production of a library of tertiary alcohols from a common intermediate with miscellaneous alkyl fragments would constitute an important advance to streamline the synthesis of drug candidates. This aspect is critical to enable the rapid exploration of structure–activity relationships (SARs), thus accelerating drug discovery campaigns.^50^ For example, the synthetic route to access a family of S1PR2 anticancer antagonists, tested for the treatment of colorectal cancer, involves the addition of Grignard reagents to cyclic ketones such as **58** ^51,52^ (Figure 5a). However, this route is inherently restricted by access to Grignard building blocks, hindering the exploration of structural diversity to optimize the pharmacological parameters. With this idea in mind, we implemented our protocol to derivatize **58** into a wide range of tertiary alcohols (**59–71**), easily introducing in one step assorted Csp^3^-fragments from commercially available alkenes, including cyclic and acyclic structures with different functionalities. This tactic includes many examples of compounds that would be extremely difficult to access via Grignard reactions and delivered synthetically relevant amounts of the desired product in all cases attempted, demonstrating its utility for the fast assembly of libraries of target compounds.

**Figure 5:**
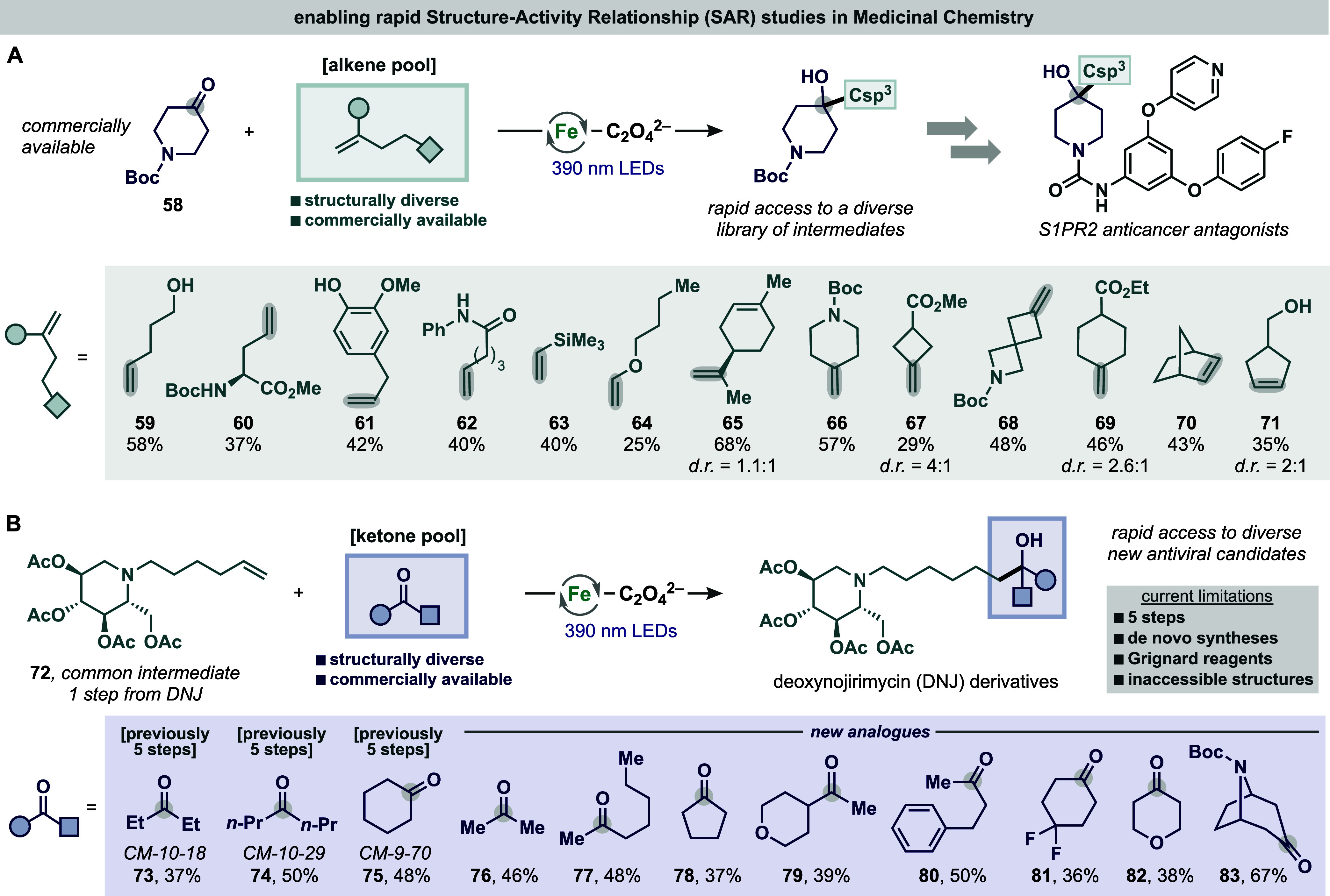
(A, B) Applications in SAR studies for drug discovery. Yields are determined by ^1^H NMR analysis. See the SI for detailed information.

*N*-Alkylated iminosugars constitute a family of compounds that have attracted increasing interest due to their activity as antivirals and in the treatment of many diseases such as cancer, neurodegenerative disorders, or diabetes, among others.^53^ For example, deoxynojirimycin (DNJ) derivatives have shown notable activity against dengue and SARS-CoV-2 virus, some of them incorporating a tertiary alcohol motif in the alkyl chain^54–56^ (Figure 5b). However, the structural modification of the alkyl substituents in the tertiary alcohol requires lengthy de novo syntheses and is intrinsically limited by a double Grignard addition to lactones (Figure S4), which only allows access to symmetrical alkylation. We envisioned the use of ferrioxalate catalysis to enable an unprecedented route for this class of compounds through the late-stage functionalization of *N*-alkylated DNJ derivatives. This blueprint provides direct access to drug candidates in one step from the common alkene intermediate **72** and bypasses the structural restrictions associated with current methods. In this manner, we have streamlined the synthesis of known compounds **73–75**, which can now be prepared in 2 steps from DNJ. Moreover, this approach allows the fast diversification of **72** into 8 new drug candidates (**76–83**), most of them inaccessible with current routes, exploiting the diversity offered by the large pool of commercially available ketones.

## Conclusions

In conclusion, we have developed a strategy utilizing ferrioxalate photocatalysis to enable the reductive cross-coupling of ketones and alkenes. This work introduces a detour from the classical Grignard reaction, using alkenes instead of organometallic reagents in a mild, effective, and sustainable new approach to assemble tertiary alcohols. This strategy capitalizes on abundant, diverse, and readily available building blocks to access intermolecular Csp^3^–Csp^3^ formation, offering new synthetic opportunities in academic and industrial settings. Ferrioxalate photocatalysis provides a unique reactivity platform based on an Earth-abundant metal that is capable of mediating the inner-sphere activation of ketones and their subsequent addition to alkenes, overcoming unfavorable polarity effects. We hope that further exploration of the reactivity modes accessed under this system can promote the discovery of versatile new tools for synthetic chemistry.

## Supplementary Material



## Notes

Experimental details, materials and methods, additional data, mechanistic studies, compound characterization, and spectra (PDF)
