# Supplementary material for: Intermolecular Reductive Coupling of Ketones and Alkenes under Ferrioxalate Photocatalysis
Source: J Am Chem Soc. 2026 Jul 13;148(28):30553–61. doi: 10.1021/jacs.6c10799 (PMC13397557; doi:10.1021/jacs.6c10799)

Intermolecular Reductive Coupling of Ketones and Alkenes under Ferrioxalate Photocatalysis

Dalila Arnaldi[1†], Niccolò Intini[1†], Partha Pratim Sen[1], Sergio Adalid[1], Kristýna Kellovská[1], Michael Guerzoni[2], Thomas D. Svejstrup[2] and Fabio Juliá[1*]

[1]*Facultad de Química, Centro de Investigación Multidisciplinar Pleiades-Vitalis, Universidad de Murcia; Campus de Espinardo, 30100 Murcia, Spain.*

[2]*Early Chemical Development, Pharmaceutical Sciences, R&D, AstraZeneca Pharmaceuticals, Gothenburg 43183, Sweden*

**Corresponding author. Email: fabio.julia@um.es*

†These authors contributed equally to this work.

TABLE OF CONTENTS

1 General Experimental Details ... 2

2 Setup for photochemical reactions ... 2

3 Database search for ketone and alkene commercial building blocks ... 3

4 Availability of Grignard reagents ... 3

5 Description of current routes for *N*-alkylated DNJ antivirals ... 4

6 Reaction conditions and control experiments ... 5

7 Assessment of the robustness of the method ... 7

7.1 Sensitivity to reaction conditions ... 7

7.2 Sensitivity to additives containing diverse functional groups ... 8

7.3 Reproducibility of the reaction in different labs ... 12

8 Starting material syntheses ... 13

9 Ketone-alkene cross-coupling reactions ... 19

9.1 General Procedures ... 19

9.2 Substrate scope ... 21

9.3 Unsuccessful substrates ... 54

9.4 Application on intramolecular reactions ... 55

10 1.0 mmol scale experiment in batch ... 57

11 Experiments under flow conditions ... 58

12 High-throughput experimentation (HTE) ... 63

13 Mechanistic experiments ... 70

13.1 Radical clocks and quenching ... 70

13.2 Light ON/OFF experiments ... 72

13.3 Evidence on inner-sphere reactivity with ketones ... 72

13.4 Comparison between inner-sphere and outer-sphere SET activation ... 73

13.5 Evidence on alkyl Fe intermediate ... 74

13.6 Deuteration labelling studies ... 76

14 Cyclic voltammetry ... 76

15 References ... 83

16 NMR spectra ... 84

1 General Experimental Details

Commercially available reagents were purchased from Sigma Aldrich, Acros Organics, Alfa Aesar, Fluorochem or BLD Pharm and used directly without purification. TBAOx was prepared according to the method described previously, and typically contains 0.5-2 equivalents of water[1]. All air and moisture sensitive reactions were carried out in oven-dried glassware under nitrogen atmosphere using standard Schlenk manifold techniques or inside of a nitrogen-filled MBraun UniLab Pro glovebox. Reaction solvents were bought from Acros Organics as extra dry and +99.5% purity and degassed by nitrogen bubbling. Analytical Thin Layer Chromatography (TLC) was carried out on 0.25 mm Merck pre-coated silica plates (60F-254) and visualized by UV light (λ= 254, 365 nm) as well as potassium permanganate stain. Flash column chromatography was performed using Merck flash silica gel (particle size 0.043-0.063 mm). Dry-loading was used to introduce the sample into the column using Celite® or silica as supporting material. ^{1}H and ^{13}C Nuclear Magnetic Resonance (NMR) spectra were acquired at various field strengths in Bruker instruments. Chemical shifts (δ) are reported in ppm, using the residual solvent peak of $CDCl_3$ (^{1}H = 7.26 and ^{13}C = 77.0 ppm) as reference. Coupling constants, *J*, are reported in hertz and refer to apparent multiplicities and not true coupling constants. Note: the O–H signal on the spectra of some compounds could not be found due to fast proton-deuterium exchange or broadening effects. All ^{13}C NMR spectra were obtained with ^{1}H decoupling. Data is reported as follows: s = singlet, d = doublet, t = triplet, q = quartet, quin = quintet, sext = sextet, sept = septet, m = multiplet, bs = broad singlet. Gas chromatography was performed on an Agilent 5973 mass spectrometer coupled to an Agilent 6890N gas chromatograph. High-resolution mass spectra (HRMS) were determined using an Agilent 1920 Infinity II HPLC module, an Agilent Q-TOF 7250B, and an Agilent Q-TOF 6550 hybrid mass spectrometer with JetStream electrospray + i-Funnel ionization source. UV-vis absorption spectra were obtained using a Horiba Duetta spectrometer and 1 mm High Precision Cells made of quartz from Hellma Analytics.

2 Setup for photochemical reactions

Photochemical reactions were conducted in crimped-cap vials using a homemade photoreactor consisting of 8 reaction-vials slots equipped with independent 390 nm or 405 nm LEDs (LED Engin LZ1-00UB00), where temperature was controlled by liquid recirculation (Fig. S1).

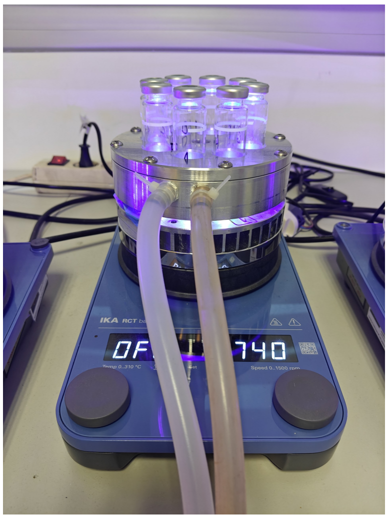

Figure S1. Homemade photoreactor equipped with independent LEDs for temperature-controlled reactions.

3 Database search for ketone and alkene commercial building blocks

The availability of commercial ketone and alkene building blocks was evaluated through searching in the database SciFinder® (Fig. S2).

<u>Search procedure</u>:

• *ketones:* a sub-structure search on the ketone fragment was carried out. Then, the results were refined to remove tautomeric species (e.g. phenols). Finally, commercially available compounds were identified, gathering 7.7 million structures.

• *alkenes:* The search was divided into monosubstituted (terminal) and 1,1-disubstituted alkenes. Sub-structure searches were performed to find 1.8 million commercially available terminal alkenes and 0.66 million 1,1-disubstituted alkenes. Without counting other types of alkenes, this represents over 2.4 million alkenes which are commercially available. *Note: the search based on 1,2-disubstituted and higher-substituted alkenes was discarded due to the inability to refine the compounds to remove aromatic compounds, which would otherwise overestimate the results.*

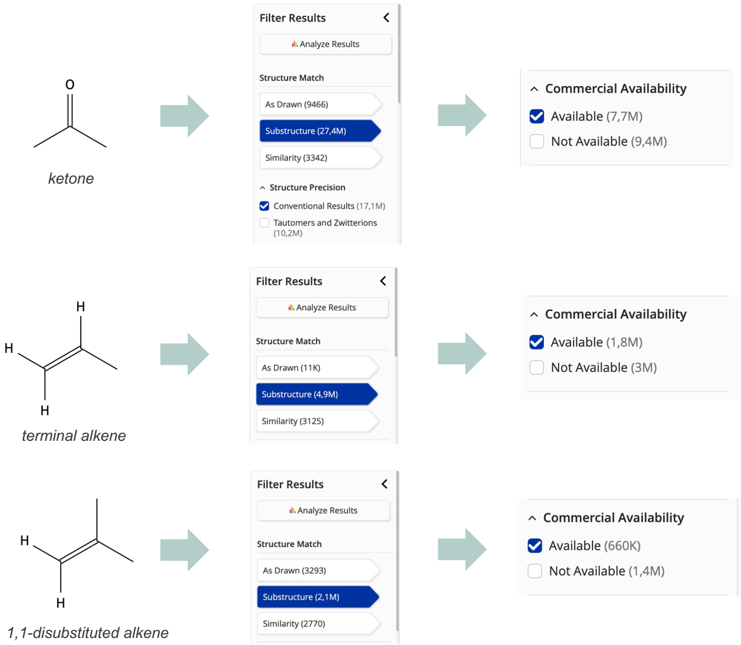

Figure S2. Search in SciFinder® for commercially available ketones and alkenes.

4 Availability of Grignard reagents

The viability of a direct synthetic route to access the tertiary alcohol products shown in this work using Grignard reagents instead of alkenes was evaluated through a database search. For this purpose, we considered all commercially-available alkene building blocks used in our work and performed a search in SciFinder® to identify possible Grignard reagents that would lead to analogous tertiary alcohol products. The criteria we followed was: (i) appearance of the Grignard structure in "Structure Search" or (ii) the involvement of the corresponding alkyl bromide (if reported) on reactions using Mg as reagent to form the Grignard *in situ*. The results are given in Fig. S3:

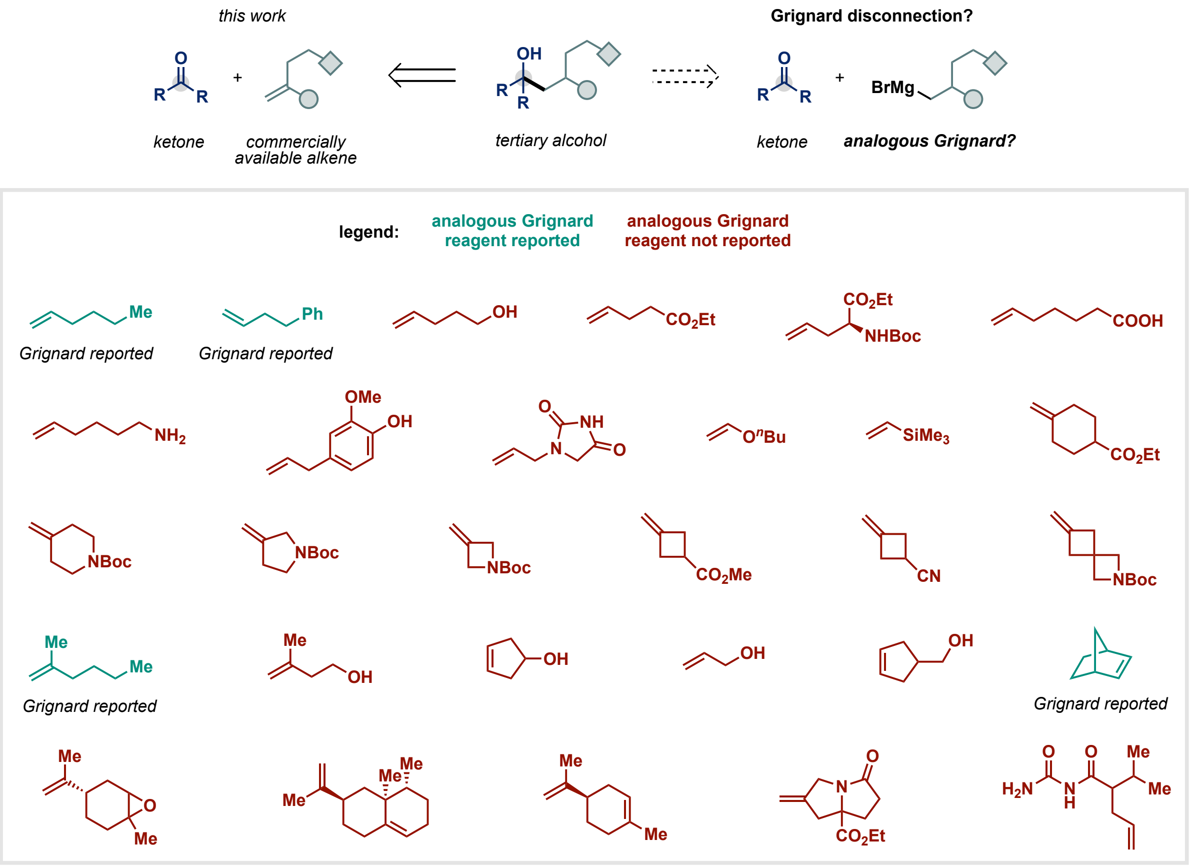

Figure S3. Availability of Grignard reagents required to access the products described in this work.

The results of this search showed that in 90% of the cases (26/29) the analogous Grignard reagents have not been reported. Moreover, in 27% of the cases (8/29) a possible alkyl bromide precursor is not reported either.

5 Description of current routes for *N*-alkylated DNJ antivirals

The reported synthetic route for *N*-alkylated DNJ antivirals containing tertiary alcohols found in publications[2] and patents (US 2009/0042268, WO 2010/027996) is shown in Figure S4. This route entails a double Grignard addition to a 8-membered ring lactone, giving tertiary alcohols which are further elaborated and finally employed for the *N*-alkylation of DNJ. Thus, this route allows symmetrical dialkylation products which must be synthetized de novo from the lactone.

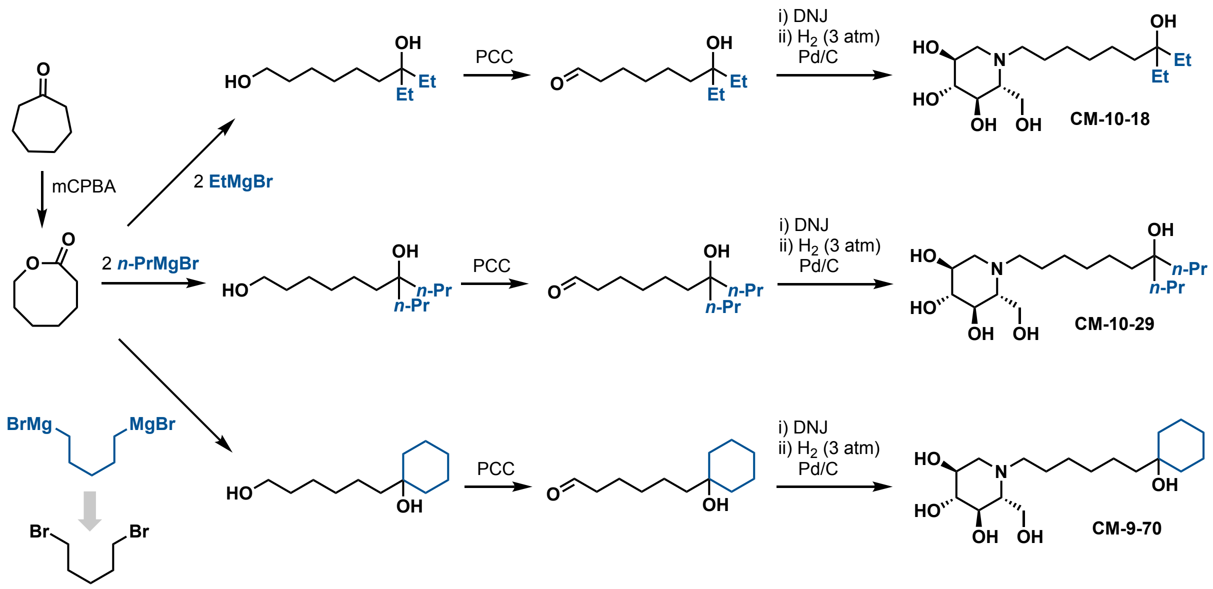

Figure S4. Reported synthesis of DNJ antivirals containing tertiary alcohols

6 Reaction conditions and control experiments

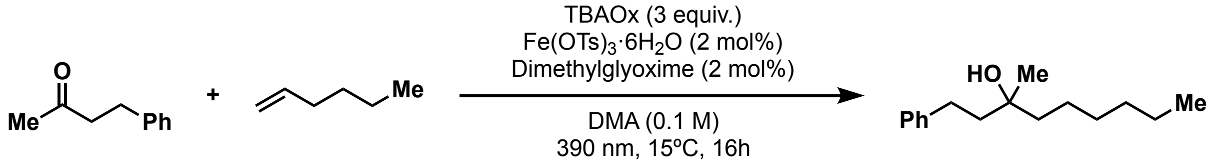

To prepare 10 mL of stock solution containing the Fe and ligand, dimethylglyoxime (2.3 mg, 0.020 mmol, 2 mol%) and $Fe(OTs)_3{\cdot}6H_2O$ (13.55 mg, 0.020 mmol, 2 mol%) were added to a round bottom flask with a stirring bar. The flask was capped with a septum and subjected to three vacuum-nitrogen cycles. 10 mL of dry DMA were added to the flask and the solution was degassed bubbling nitrogen for 5 min. Inside of a N_2-filled glovebox TBAOx (172 mg, 0.3 mmol, 3.0 equiv.) was added to a microwave vial equipped with a stirring bar. The tube was capped with an aluminium crimp cap with PTFE/butyl septum and taken out of the glovebox. Then, 1 mL of the stock solution of iron and ligand was added to this vial via a syringe under a positive N_2 flow, followed by 4-phenyl-2-butenone (15 μL, 0.1 mmol, 1.0 equiv.) and 1-hexene (25 μL, 0.2 mmol, 2.0 equiv). The mixture was stirred under 390 nm light irradiation at 15°C for 16 h. After the reaction is completed, acetophenone (Internal standard for HPLC analysis 11.5 μL, 0.1 mmol, 1.0 equiv) was added. A 50 μL aliquot of the reaction mixture was taken and filtered by a pipette charged with celite, collecting the sample in a GC vial. The resultant sample was analyzed by HPLC to determine the yield of the reaction. Results are reported in Table S1.

Table S1. Variation of reaction conditions

Entry	Variation	Yield (%)[a]
1	none	70%
2	1 equiv. of TBAOx	22%
3	5 equiv. of TBAOx	74%
4	1 equiv. of alkene	58%
5	4 equiv. alkene	59%
6	THF	50%
7	MeCN	17%
8	DMSO	0%
9	EtOAc	14%
10	DMF	64%
11	DCM	0%
12	$FeCl_3 \cdot 6\ H_2O$	62%
13	$Fe_2(SO_4)_3$ hydrate	66%
14	$Fe(NO_3) \cdot 9\ H_2O$	70%
15	$Fe_2(C_2O_4)_3 \cdot 6\ H_2O$	67%
16	$FePO_4 \cdot 4\ H_2O$	68%
17	$FeCl_3$	64%
18	$FeCl_3$ + molecular sieves	28%
19	5 mol% $Fe(OTs)_3 \cdot 6H_2O$	46%
20	10 mol% $Fe(OTs)_3 \cdot 6H_2O$	51%
21	15 mol% $Fe(OTs)_3 \cdot 6H_2O$	37%
22	no ligand	68%
23	salicylaldoxime as ligand	65%
24	4,4'-di-*tert*-butyl-2,2'-bipyridine as ligand	53%
25	+ 10 µL H_2O	68%
26	0.05 M	52%
27	0.2 M	61%
28	0.4 M	63%
29	405 nm	71%
30	Kessil Lamp 427 nm, cooled with a fan	12%
31	Kessil Lamp 390 nm, cooled with a fan	49%
32	Lithium Oxalate	0%
33	Sodium Oxalate	0%
34	Potassium Oxalate	0%
35	Caesium Oxalate	0%
	Control experiments	
36	No Fe	traces
37	No TBAOx	0%
38	No light	0%

[a]Yields were determined by HPLC analysis using 0.1 mmol acetophenone as an internal standard.

7 Assessment of the robustness of the method

7.1 Sensitivity to reaction conditions

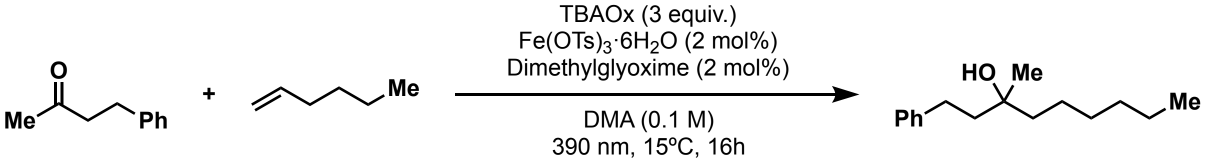

In a microwave vial equipped with a stirring bar were added dimethylglyoxime (0.2 mg, 0.002 mmol, 0.02 equiv.) and $Fe(OTs)_3{\cdot}6H_2O$ (1.4 mg, 0.002 mmol, 0.02 equiv.). The vial has been loaded in the glovebox where TBAOx (172 mg, 0.3 mmol, 3.0 equiv.) was added. The tube was capped with an aluminium crimp cap with PTFE/butyl septum and taken out of the glovebox. An appropriate amount of dry and degassed DMA was added considering the target concentration. Subsequently, 4-phenyl-2-butenone (15 μL, 0.1 mmol, 1.0 equiv.), 1-hexene (25 μL, 0.2 mmol, 2.0 equiv), and eventual water were added via syringe under a positive N_2 flow. The mixture was stirred under 390 nm light irradiation at 15°C for 16 h. After the reaction is completed, acetophenone (Internal standard for HPLC analysis 11.5 μL, 0.1 mmol, 1.0 equiv) was added. A 50 μL aliquot of the reaction mixture was taken and filtered by a pipette charged with celite, collecting the sample in a GC vial. Yields were determined by HPLC analysis. Results are reported in table S2. *Note*: in the case of the reaction under air, the mixture has been exposed to air without aluminium cap for two hours. Then, the reagents were added and finally the vial was crimped again without degassing.

Table S2. Sensitivity to variations on the reaction conditions

Entry	Variation	Yield (%)[a]
1	none	70%
2	0.4 M	63%
3	0.05 M	52%
4	10 μL H_2O	68%
5	20 μL H_2O	48%
6	405 nm	71%
7	25º C	50%
8	45º C	43%
9	Air	52%
10	No ligand	68%[b]
11	1 mmol scale	62%

[a]Yields were determined by HPLC analysis using 0.1 mmol acetophenone as an internal standard.
[b] *Note: although the reaction can also be performed similarly in absence of ligand in most cases, we observed improved reproducibility in presence of ligand for some substrates.*

These results were plotted in a diagram (Fig. S5), displaying a fairly good robustness to changes on reaction conditions:

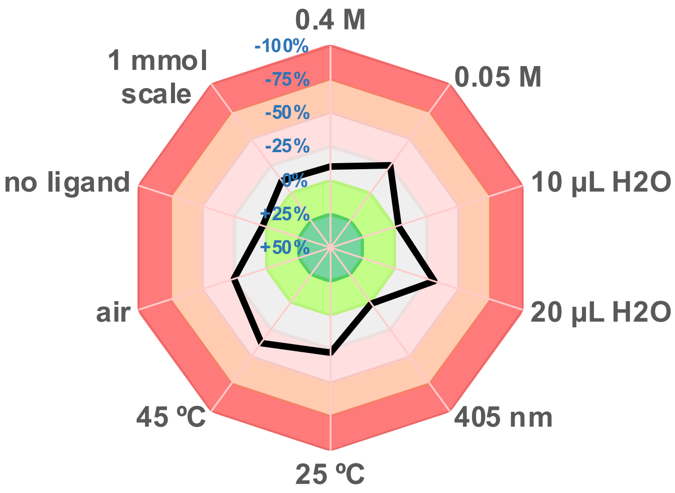

Figure S5. Sensitivity diagram[3] for the variation on reaction conditions for the ketone-alkene cross-coupling protocol.

7.2 Sensitivity to additives containing diverse functional groups

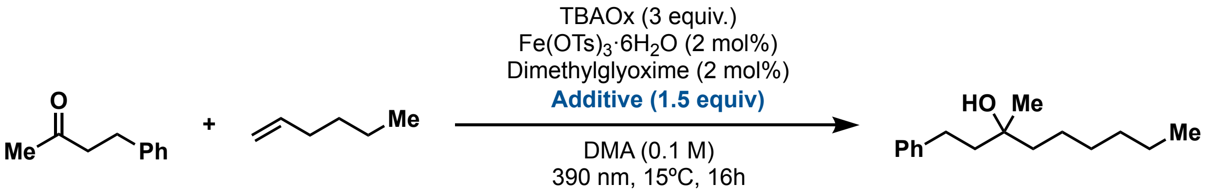

Stock solution method has been employed for the addition of iron and ligand. To prepare 10 mL of stock solution, dimethylglyoxime (2.3 mg, 0.020 mmol, 0.02 equiv.) and $Fe(OTs)_3{\cdot}6H_2O$ (13.55 mg, 0.020 mmol, 0.02 equiv.) were added to a round bottom flask with a stirring bar. The flask has been capped with a septum and three vacuum-nitrogen cycles have been done. 10 mL of dry DMA have been added to the flask and the solution has been degassed bubbling nitrogen for 5 min.

In a microwave vial equipped with a stirring bar were added TBAOx (172 mg, 0.3 mmol, 3.0 equiv.) and the additive if solid (1.5 equiv.). The tube was capped with an aluminium crimp cap with PTFE/butyl septum and taken out of the glovebox. 1 mL of the stock solution of iron and ligand has been added to the vial was then added via a syringe under a positive N_2 flow, followed by 4-phenyl-2-butenone (15 µL, 0.1 mmol, 1.0 equiv.), 1-hexene (25 µL, 0.2 mmol, 2.0 equiv.), and additive if liquid (1.5 equiv.). The mixture was stirred under 390 nm light irradiation at 15°C for 16 h. After the reaction is completed, acetophenone (Internal standard for HPLC analysis 11.5 µL, 0.1 mmol, 1.0 equiv.) was added.

A 50 µL aliquot of the reaction mixture was taken and filtered by a pipette charged with celite, collecting the sample in a GC vial. Yields were determined by HPLC analysis. Results are reported in Table S3.

Table S3. Screening of additive effects

Ranking of occurrence	Functional group	Additive	Yield (%)[a]
-	-	none	70%
Most frequent functional groups in bioactive compounds			
#1	amide	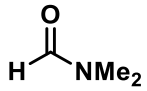	77
#2	ether	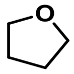	65%
#3	R_3N	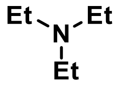	56%
#4	C–F	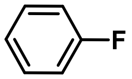	45%
#5	R_2NH	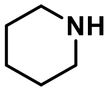	75%
#6	C–Cl	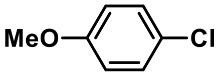	12%
#7	alkOH	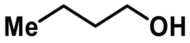	74%
#8	aryl ketone	-	-
#9	$alkCO_2H$	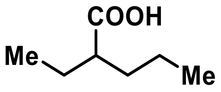	46%
	$ArCO_2H$	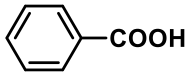	36%
#10	ArOH	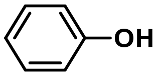	43%
#11	sulfonamide	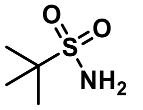	64%
#12	$ArNH_2$	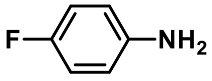	83%
#13	$alkNH_2$	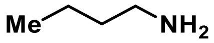	30%
#14	urea	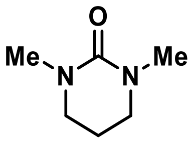	74%
#15	ester	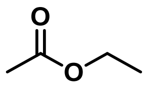	70%
#16	nitrile	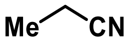	59%

#17	alkyl ketone	-	-
#18	alkene	-	-
#19	thioether	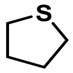	2%
#20	carbamate	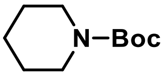	73%
		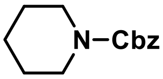	18%
		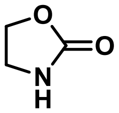	73%
#21	C–Br	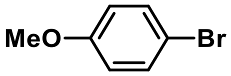	5%
#22	sulfone	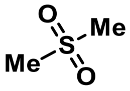	70%
#23	guanidine	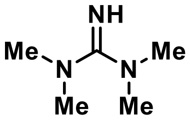	71%
#24	RNO_2	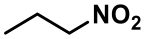	5%
#25	alkyne	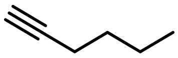	45%
#26	hydroxamic acid	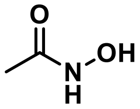	5%
#27	acetal	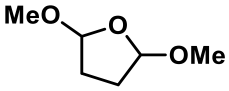	74%
#28	amidine	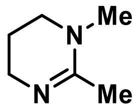	76%
#29	acrylamide	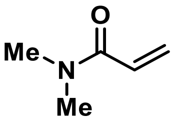	18%
#30	arene	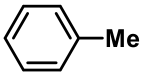	81%
Most frequent (hetero)aryl fragments in marketed drugs			
#1	benzene	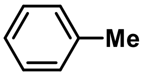	81%
#2	pyridine	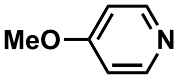	68%

#3	imidazole	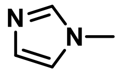	76%
#4	thiazole	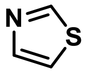	4%
#5	phenothiazine	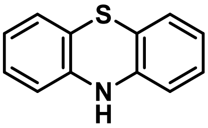	38%
#6	tetrazole	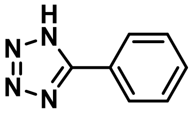	45%
#7	indole	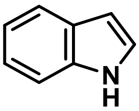	57%
#8	thiophene	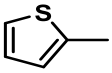	42%
#9	pyrimidine	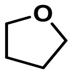	23%
#10	naphthalene	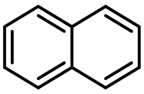	1%
Other common functional groups			
-	pyrrole	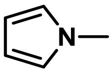	66%
-	furan	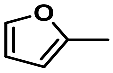	55%
-	enones	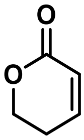	7%
-	e-poor arenes	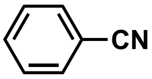	5%
-	thiol	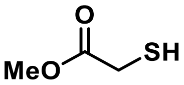	-

[a]Yields were determined by HPLC analysis using 0.1 mmol acetophenone as an internal standard.

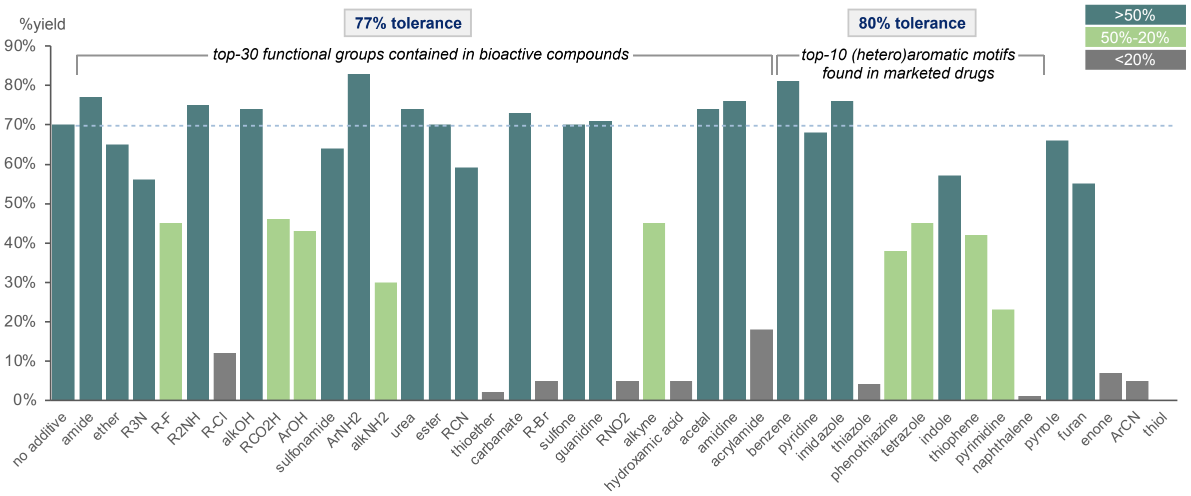

Figure S6. Graphical representation of the results obtained in the additive screening experiments.

<u>Discussion:</u> The presence of additives containing top-30 functionalities found in bioactive compouds (excluding ketones and alkenes) delivered the product in >20% yield in 21/27 cases, which represents a tolerance to 78% of these functional groups. Similarly, the reaction worked with >20% yield in presence of 8/10 heteroarenes, which represents a tolerance to 80% of the top-10 heteroarenes found in marketed drugs. *Note: The aim of this analysis was to obtain a preliminary exploration of functional group compatibility. Some functional groups causing a marked decrease on yield were tolerated nonetheless in effective reactions when present in different structures or after application of slight modifications on reaction conditions (e.g. thioether is present in compound **28**, two phenols are present in compound **27**, and naphthalene is present in compound **53**). Therefore, cases should be evaluated individually to have detailed information of the performance of specific target compounds.*

7.3 Reproducibility of the reaction in different labs

To validate the reproducibility of the method, a model reaction was run at AstraZeneca's labs at their site in Gothenburg involving different batches of all reagents, a different irradiation setup and different practitioners:

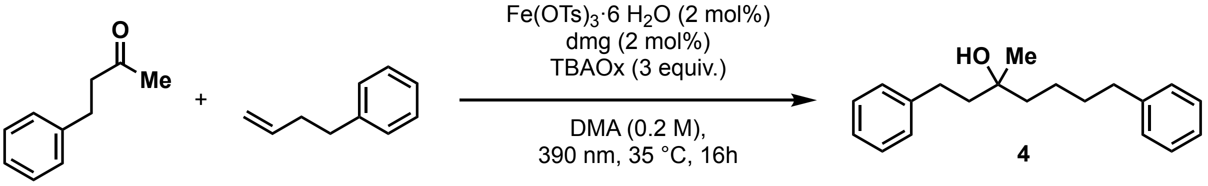

In the glovebox, tetrabutylammonium oxalate (344 mg, 0.60 mmol, 3.0 equiv.) was weighed out in a vial containing the stirring bar. In another vial, $Fe(OTs)_3{\cdot}6H_2O$ (2.71 mg, 4.00 µmol, 0.02 equiv.) and dimethylglyoxime (0.464 mg, 4.00 µmol, 0.02 equiv.) were dissolved in 1 mL of DMA. Then, the solution containing the iron salt and the ligand was added to the vial containing TBAOx followed by the addition of 4-phenylbutan-2-one (0.033 mL, 0.20 mmol, 1.0 equiv.) and 4-phenylbutene (0.060 mL, 0.40 mmol, 2.0 equiv.). The reaction was stirred under 390 nm irradiation provided by two Kessil PR160L lamps for 16h with a fan cooling (see Figure S6). The crude reaction mixture was then diluted with MTBE and washed with NaOH 0.1 M three times. The organic layer was then washed with brine, dried over $MgSO_4$, and evaporated using rotary evaporation to afford a yellow oil that was directly purified with automated

flash column chromatography to afford **4** as a colorless oil (40 mg, 0.142 mmol, 71%). The reaction was repeated a second time, affording 39.3 mg (69% yield) of the product. These results are comparable to that obtained at the labs in Murcia (65% yield for **4**).

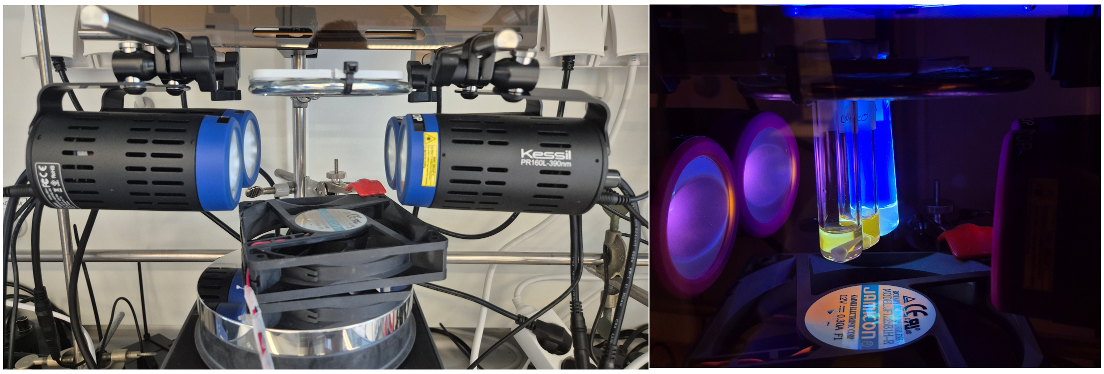

Figure S7. Irradiation setup used at AstraZeneca

8 Starting material syntheses

N-Boc allyl-glycine methyl ester (S1)

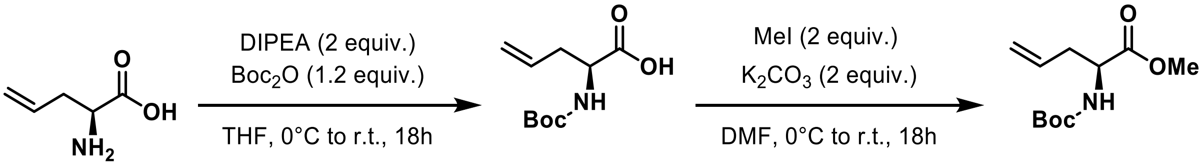

In a round bottom flask, *L*-allylglycine (1.5 g, 13.03 mmol) was added to a solution of THF/H_2O (10:1 ratio, 0.2 M, 65 mL). The solution was cooled at 0°C with an ice bath and Boc_2O (3.37 g, 26.06 mmol, 2.0 equiv.) and Diisopropylethylamine (DIPEA, 3.6 mL, 3.41 g, 15.6 mmol, 1.2 equiv.) were added. The reaction mixture was allowed to warm up at room temperature and stirred for 18h. The reaction mixture has been then acidified using 1 M HCl until pH 2, then the mixture has been extracted three times with EtOAc. The combined organic layers were washed with brine, dried over $MgSO_4$ and concentrated in vacuo. The crude has been then dissolved in DMF (0.5 M, 26 mL) and cooled to 0°C. Then K_2CO_3 (3.59 g, 26.2 mmol, 2.0 equiv.) and iodomethane (1.6 mL, 26.2 mmol, 2.0 equiv.) were then added. The mixture was allowed to warm up at room temperature and stirred for 18 hours.

The reaction mixture has been then diluted with water and extracted three times with EtOAc. The combined organic layers were dried over $MgSO_4$.

The crude has been purified by flash column chromatography (Cyclohexane/EtOAc 6/4 to 3/7 gradient) to afford 2.71 g of product **S1** in a 91% yield as colorless oil.

^{1}H NMR (400 MHz, $CDCl_3$) δ 5.71 (ddt, J = 17.0, 9.7, 7.2 Hz, 1H), 5.19 – 5.10 (m, 2H), 5.05 (d, J = 8.1 Hz, 1H), 4.44 – 4.35 (m, 1H), 3.75 (s, 3H), 2.52 (ddt, J = 27.6, 13.8, 7.1 Hz, 2H), 1.45 (s, 9H).

^{13}C NMR ($CDCl_3$, 101 MHz) δ 172.6, 155.2, 132.3, 119.1, 79.9, 77.2, 52.9, 52.2, 36.8, 28.3.

Data in accordance with literature.[4]

1-(6,7-dihydrothieno[3,2-*c*]pyridin-5(4*H*)-yl)pent-4-en-1-one (S2)

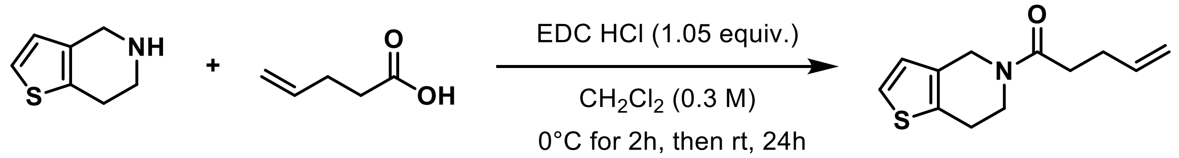

To a solution of the pentenoic acid (991 µL, 1.0g, 10.00 mmol, 1.00 equiv.) in DCM (0.3 M, 33 mL), EDC (2,01 g, 10.5 mmol, 1.05 equiv.) was added at 0°C. After 5 min, the 4,5,6,7-Tetrahydrothieno[3,2-c]pyridine (1.21 mL, 1.39 g, 10.00 mmol, 1.0 eq.) was added and the reaction was stirred overnight at room temperature. The crude was diluted with DCM (15 mL) and HCl 1M (20 mL), then the aqueous layer was extracted with DCM (3x20 mL). The combined organic layers were dried over Na_2SO_4 and the solvent was removed under reduced pressure. The crude has been purified by column chromatography from 6/4 to 3/7 Cyclohexane/ EtOAc to afford the product **S2** as yellow liquid (1.43 g, 64% yield).

^{1}H NMR (400 MHz, $CDCl_3$) δ 7.11 (dd, J = 9.1, 5.1 Hz, 1H), 6.77 (dd, J = 5.2, 3.7 Hz, 1H), 5.94 – 5.78 (m, 1H), 5.11 – 4.93 (m, 2H), 4.64 (t, J = 1.8 Hz, 1H), 4.53 (t, J = 1.7 Hz, 1H), 3.89 (t, J = 5.8 Hz, 1H), 3.76 – 3.69 (m, 1H), 2.92 – 2.85 (m, 1H), 2.82 (td, J = 5.7, 2.9 Hz, 1H), 2.54 – 2.34 (m, 4H).

^{13}C NMR ($CDCl_3$, 101 MHz) δ 171.3, 171.1, 137.4, 134.4, 132.6, 132.1, 131.2, 125.2, 124.4, 123.6, 123.4, 115.3, 115.2, 45.6, 43.4, 42.6, 39.7, 33.1, 32.8, 29.3, 29.2, 25.7, 24.8.

HRMS (EI) calcd for $C_{12}H_{16}NOS$: 222.0952 ($[M+H]^+$), found 222.0952.

***N*-(2-(1*H*-indol-3-yl)ethyl)pent-4-enamide (S3)**

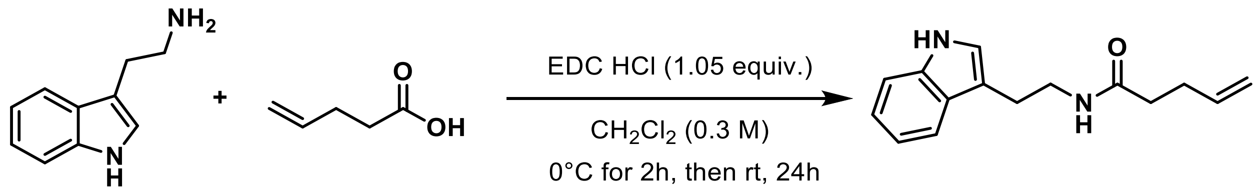

To a solution of the pentenoic acid (991 µL, 1.0g, 10.00 mmol, 1.00 equiv.) in DCM (0.3 M, 33 mL), EDC (2,01 g, 10.5 mmol, 1.05 equiv.) was added at 0°C. After 5 min, the tryptamine (1.6 g, 10.00 mmol, 1.00 eq.) was added and the reaction was stirred overnight at room temperature. The crude was diluted with DCM (15 mL) and HCl 1M (20 mL), then the aqueous layer was extracted with DCM (3x20 mL). The combined organic layers were dried over Na_2SO_4 and the solvent was removed under reduced pressure. The crude has been purified by column chromatography from DCM to DCM/Acetone 8/2 to afford the product **S3** as white powder (1.18g, 49% yield).

^{1}H NMR (400 MHz, $CDCl_3$) δ 8.13 (s, 1H), 7.66 – 7.54 (m, 1H), 7.45 – 7.35 (m, 1H), 7.24 – 7.17 (m, 1H), 7.16 – 7.08 (m, 1H), 7.04 (s, 1H), 5.85 – 5.69 (m, 1H), 5.59 (s, 1H), 5.09 – 4.89 (m, 2H), 3.66 – 3.55 (m, 2H), 2.98 (t, *J* = 6.7 Hz, 2H), 2.43 – 2.27 (m, 2H), 2.21 (t, *J* = 7.6 Hz, 2H).

^{13}C NMR ($CDCl_3$, 101 MHz) δ 172.4, 137.2, 136.6, 127.5, 122.4, 122.2, 119.6, 118.9, 115.7, 113.2, 111.4, 39.8, 36.1, 29.7, 25.5.

Melting point: 64.2 ºC.

HRMS (ESI) calcd for $C_{15}H_{19}N_2O$: ($[M+H]^+$) 243.1492, found 243.1497.

***1*-(4-(pyridin-2-yl)piperazin-1-yl)pent-4-en-1-one (S4)**

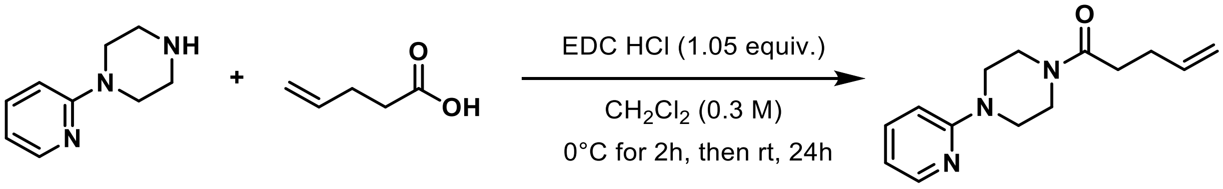

To a solution of the pentenoic acid (991 µL, 1.0g, 10.00 mmol, 1.00 equiv.) in DCM (0.3 M, 33 mL), EDC (2,01 g, 10.5 mmol, 1.05 equiv.) was added at 0°C. After 5 min, the 1-(pyridin-2-yl)piperazine (1.50 mL, 10.00 mmol, 1.00 eq.) was added and the reaction was stirred overnight at room temperature. The crude was diluted with DCM (15 mL) and HCl 1M (20 mL), then the aqueous layer was extracted with DCM (3x20 mL). The combined organic layers were dried over Na_2SO_4 and the solvent was removed under reduced pressure. The crude has been purified by column chromatography from DCM to DCM/Acetone 7/3 to afford the product **S4** as yellow liquid (0.78 g, 40% yield).

^{1}H NMR (400 MHz, CDCl$_3$) δ 8.20 (ddd, *J* = 4.9, 2.0, 0.9 Hz, 1H), 7.52 (dt, 1H), 6.71 – 6.63 (m, 2H), 5.94 – 5.81 (m, 1H), 5.13 – 4.97 (m, 2H), 3.80 – 3.72 (m, 2H), 3.68 – 3.57 (m, 4H), 3.54 – 3.46 (m, 2H), 2.50 – 2.39 (m, 4H).

^{13}C NMR (CDCl$_3$, 101 MHz) δ 171.0, 159.1, 148.0, 148.0, 137.8, 137.4, 115.4, 114.0, 107.3, 53.5, 41.2, 32.6, 29.3

HRMS (EI) calcd for $C_{14}H_{20}N_3O$: 246.1601 ($[M+H]^+$), found 246.1604.

***tert*-butyl 6-methylene-2-azaspiro[3.3]heptane-2-carboxylate (S5)**

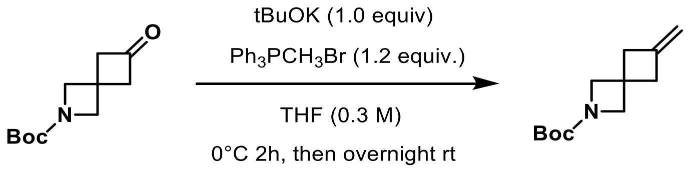

To a two-necked round-bottom flask containing KOt-Bu (612 mg, 5.45 mmol, 1.0 equiv.) was charged with a stir bar. The system was purged with nitrogen, then THF (18 mL, 0.3 M) was added into the flask. Ph_3PCH_3Br (2.34 g, 6.55 mmol, 1.2 equiv.) was added slowly against a flow of argon and the mixture was stirred at 0°C for 2h. A solution of ketone (1.27 g, 6.00 mmol, 1.1 equiv.) in THF (6 mL, 1.0 M) was then added dropwise via syringe, and the mixture was stirred at room temperature overnight. After completion of the reaction, the reaction was taken up in EtOAc and washed with water (2 x 10 mL) and brine (2 x 10 mL). The organic layers were dried over anhydrous Na_2SO_4, concentrated in vacuo. The crude has been purified by column chromatography in Cyclohexane/EtOAc 9/1 to obtain product **S5** as white powder (587 mg, 52% yield).

^{1}H NMR (400 MHz, CDCl$_3$) δ 4.78 (p, *J* = 2.2 Hz, 2H), 3.90 (q, *J* = 1.5 Hz, 4H), 2.82 (q, *J* = 2.4 Hz, 4H), 1.41 (q, *J* = 1.6 Hz, 9H).

^{13}C NMR (101 MHz, CDCl$_3$) δ 156.3, 142.9, 142.8, 107.2, 79.3, 61.2, 42.9, 33.1, 28.4.

Data in accordance with literature[5].

Diphenolic acid derivative (S6)

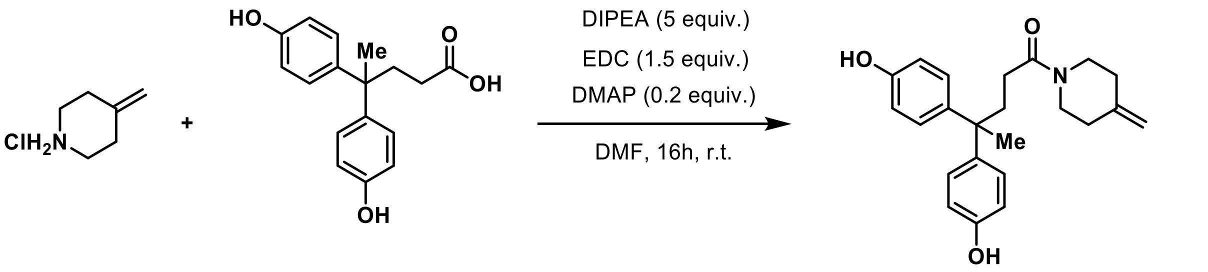

In a round bottom flask under nitrogen atmosphere, diphenolic acid (1.07 g, 3.74 mmol, 1.0 equiv.) and 4-methylenepyperidine hydrochloride (500 mg, 3.74 mmol, 1.0 equiv.) dissolved in DMF (10 mL, 0.36 M). Subsequently DMAP (91.4 mg, 748 μmol, 0.2 equiv.), DIPEA (3.26 mL, 18.7 mmol, 5.0 equiv.), and EDC (1.08 g, 5.61 mmol, 1.5 equiv.) were added, each in one portion. The mixture was stirred overnight at room temperature. The reaction mixture was poured into water and the aqueous phase extracted three times with DCM. The combined organic phases were washed with brine, dried over $MgSO_4$, and concentrated under reduced pressure. The crude was purified by flash column chromatography from Cyclohexane/EtOAc 1/1 to 2/8 afford product **S6** as white powder (602 mg, 45% yield).

^{1}H NMR (400 MHz, DMSO) δ 9.16 (s, 2H), 6.95 (d, J = 8.2 Hz, 4H), 6.65 (d, J = 8.1 Hz, 4H), 4.73 (s, 2H), 3.41 (t, J = 5.9 Hz, 2H), 3.24 – 3.14 (m, 3H), 2.21 (dd, J = 10.9, 5.4 Hz, 2H), 2.11 – 2.00 (m, 6H), 1.49 (s, 3H).

^{13}C NMR (101 MHz, DMSO) δ 171.1, 155.4, 145.4, 140.0, 128.2, 115.1, 109.8, 49.1, 46.6, 44.4, 43.0, 39.6, 39.4, 37.4, 35.0, 34.2, 29.0, 27.6.

Melting point: 186.3 ºC.

HRMS (EI) calcd for $C_{23}H_{27}NO_3$: 365.1991 ([M]$^+$), found 365.1946.

Biotin derivative (S7)

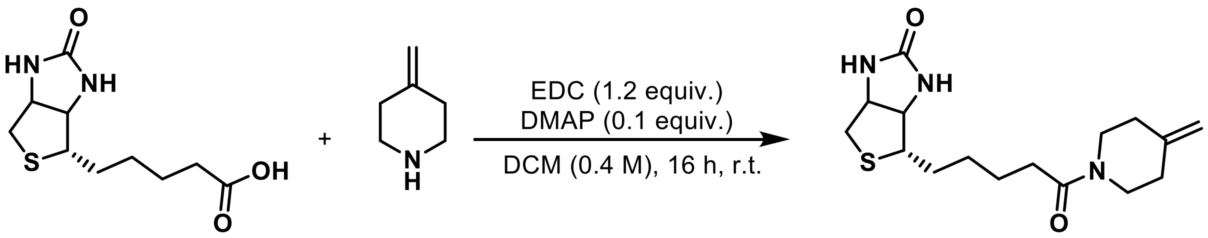

To a dried flask were added EDC (2.60 g, 12.0 mmol, 1.2 equiv.) and DMAP (147 mg, 0.1 mmol, 0.1 equiv.) and (S)-Biotin (2.44 g, 10 mmol, 1.0 equiv.) The flask was evacuated and backfilled with N_2 three times. DCM (30 mL) was then added, and the reaction was cooled to 0ºC. 4-methylenepiperidine (1.7 mL, 13 mmol, 1.3 equiv.) was then added and the reaction mixture was allowed to stir at room temperature for 24 hours. Upon reaction completion, the reaction was quenched with 1 M HCl. The mixture was extracted with DCM three times. Organic layers were combined, washed with $NaHCO_3$ and brine, then dried over anhydrous $MgSO_4$ and concentrated in vacuo. The crude mixture was purified by flash column chromatography on a silica gel column using a mixture of DCM/*i*PrOH 85/15 to provide the product **S7** as white powder (1.40 g, 43%).

^{1}H NMR (400 MHz, MeOD) δ 4.81 (s, 2H), 4.55 – 4.44 (m, 1H), 4.31 (dd, *J* = 7.9, 4.5 Hz, 1H), 3.64 – 3.50 (m, 4H), 3.22 (ddd, *J* = 8.7, 6.0, 4.4 Hz, 1H), 2.93 (dd, *J* = 12.8, 5.0 Hz, 1H), 2.71 (d, *J* = 12.7 Hz,

1H), 2.45 (t, J = 7.5 Hz, 2H), 2.28 (t, J = 5.9 Hz, 2H), 2.21 (t, J = 5.9 Hz, 2H), 1.68 (dtdd, J = 28.8, 21.6, 13.6, 7.1 Hz, 4H), 1.49 (q, J = 7.3 Hz, 2H).

^{13}C NMR (101 MHz, MeOD) δ 173.84, 166.11, 145.96, 110.20, 63.34, 61.62, 57.01, 44.61, 41.05, 36.02, 35.20, 33.82, 29.90, 29.54, 26.49.

Melting point: 111.2 ºC.

HRMS (EI) calcd for $C_{16}H_{25}N_3O_2SNa$: 346.1560 ([M]$^+$), found 346.1562.

***tert*-butyl 4-oxoazepane-1-carboxylate (S8)**

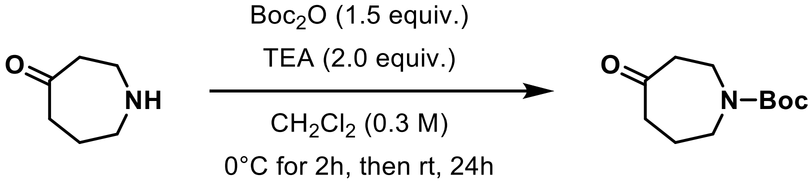

In a round bottom flask, 25 mL of dry DCM were added to azepan-4-one (0.5 g, 4.4 mmol, 1.0 equiv.) under inert atmosphere. The solution was cooled at 0°C with an ice bath and TEA (1.23 mL, 8.8 mmol, 2.0 equiv.) were added and the mixture was stirred for 30 minutes. Then Boc_2O (1.44 g, 6.6 mmol, 1.5 equiv.) were added, and the reaction mixture was allowed to warm up at room temperature and stirred for 18h. The reaction mixture has been treated with 1 M HCl, then the aqueous phase was extracted three times with DCM. The combined organic layers were washed with brine, dried over $MgSO_4$ and concentrated in vacuo. The crude has been purified by column chromatography from DCM to DCM/Acetone 9/1 to afford the product **S8** as colorless oil (0.73 g, 78% yield).

^{1}H NMR (400 MHz, $CDCl_3$) δ 3.63 – 3.46 (m, 4H), 2.70 – 2.55 (m, 4H), 1.78 (s, 2H), 1.45 (s, 9H).

^{13}C NMR ($CDCl_3$, 101 MHz) δ 211.9, 154.5, 80.1, 49.6, 48.9, 43.9, 43.2, 43.0, 28.5, 25.7.

Data in accordance with the literature[6].

***N*-phenylhept-6-enamide (S9)**

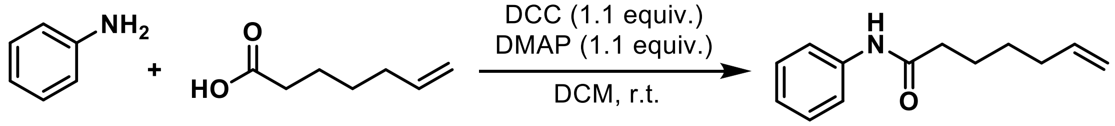

To a dried flask were added N,N′-Dicyclohexylcarbodiimide (2.14 g, 10.4 mmol, 1.3 equiv.) and DMAP (1.37 g, 10.4 mmol, 1.3 equiv.). The flask was evacuated and backfilled with N_2 three times. DCM (30 mL) was then added, and the reaction was cooled to 0ºC. Hept-6-enoic acid (1.1 mL, 8 mmol, 1 equiv.) was then added and the reaction mixture was allowed to stir for 5 min. Aniline (0.9 mL, 9.6 mmol, 1.2 equiv.) was then added and the reaction mixture was allowed to stir at room temperature for 24 hours. Upon reaction completion, the reaction was quenched with 1 M HCl. The mixture was extracted with DCM three times. Organic layers were combined, washed with $NaHCO_3$ and brine, then dried over anhydrous $MgSO_4$ and concentrated in vacuo. The crude mixture was purified by flash column chromatography on a silica gel column using a mixture of Cyclohexane/EtOAc 8/2 to provide **S9** as pale orange powder (1.27 g, 74%).

^{1}H NMR (400 MHz, DMSO) δ 9.85 (s, 1H), 7.65 – 7.57 (m, 2H), 7.28 (t, J = 7.8 Hz, 2H), 7.01 (t, J = 7.4 Hz, 1H), 5.81 (ddt, J = 16.9, 10.3, 6.6 Hz, 1H), 5.11 – 4.86 (m, 2H), 2.32 (t, J = 7.4 Hz, 2H), 2.05 (q, J = 7.0 Hz, 2H), 1.61 (p, J = 7.5 Hz, 2H), 1.41 (p, J = 7.5 Hz, 2H).

^{13}C NMR (101 MHz, DMSO) δ 171.6, 139.8, 139.0, 129.0, 123.3, 119.5, 115.2, 36.7, 33.4, 28.4, 25.1. Data in accordance with literature[7].

1-cyclopropyl-3-phenylpropan-1-one (S10)

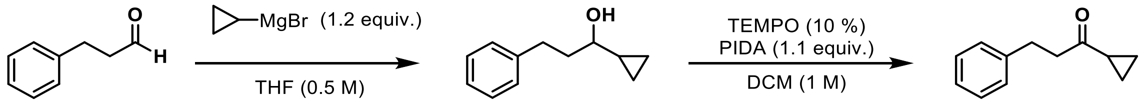

To a solution of hydrocinnamaldehyde (5.55 mL, 42.0 mmol, 1.0 equiv.) in THF (34 mL) at -23 °C was added cyclopropylmagnesium bromide (0.50 M in THF, 100 mL, 50.0 mmol, 1.2 equiv.) dropwise. Following the addition, the reaction was allowed to warm to RT and stirred for 16 h. The mixture was then cooled to 0 °C and quenched by the dropwise addition of sat. aq. NH_4Cl. The layers were separated and the aqueous layer extracted with EtOAc (x3). The combined organic layers were washed with brine, dried over $MgSO_4$ and concentrated in vacuo. Purification by flash column chromatography on a silica gel column using a mixture of Pentane/EtOAc 95/5 to provide 1-cyclopropyl-3-phenylpropan-1-ol as a colourless oil (4.1 g, 50%).
To a solution of 1-cyclopropyl-3-phenylpropan-1-ol (4.1 g, 23.2 mmol) in DCM was added TEMPO (360 mg, 2.3 mmol, 0.1 equiv.) and BAIB (8.9 g, 27.9 mmol, 1.1 equiv.) sequentially. The mixture was stirred for 36 h, after which it was diluted with DCM and washed with $Na_2S_2O_3$. The layers were separated and the aqueous layer extracted with DCM (x4). The combined organic layers were dried over $MgSO_4$ and concentrated in vacuo. Purification by flash column chromatography on a silica gel column using a mixture of Pentane/EtOAc 95/5 to provide the product **S10** as yellow oil (3.7 g, 92%).
^{1}H NMR (400 MHz, CDCl$_3$) δ 7.34 – 7.24 (m, 2H), 7.24 – 7.14 (m, 3H), 3.09 – 2.72 (m, 4H), 1.91 (tt, J = 7.8, 4.6 Hz, 1H), 1.05 – 0.99 (m, 2H), 0.88 – 0.80 (m, 2H).
^{13}C NMR (CDCl$_3$, 101 MHz) δ 210.0, 141.2, 128.5, 128.4, 126.1, 45.0, 30.0, 20.6, 19.0, 10.7.
Data in accordance with literature[8].

(2*R*,3*R*,4*R*,5*S*)-2-(acetoxymethyl)-1-(hex-5-en-1-yl)piperidine-3,4,5-triyl triacetate (72)

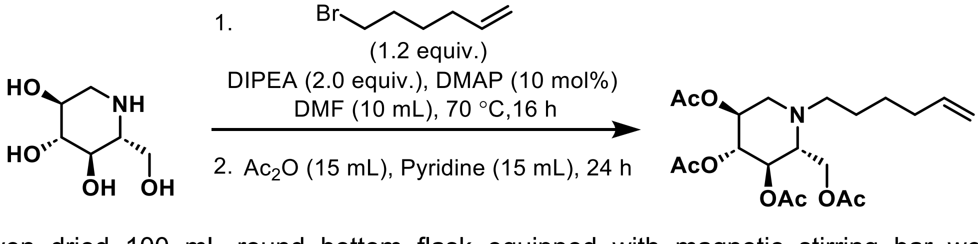

To an oven dried 100 mL round bottom flask equipped with magnetic stirring bar were added (2*R*,3*R*,4*R*,5*S*)-2-(hydroxymethyl)piperidine-3,4,5-triol **DNJ** (326.3 mg, 2 mmol, 1.0 equiv.), 6-bromohex-1-ene (320 µL, 2.4 mmol, 1.2 equiv.), DIPEA (697 µL, 4.0 mmol, 2.0 equiv.), DMAP (24.4 mg, 0.2 mmol, 10 mol%) and 10 mL DMF. The flask was then sealed with rubber septa followed by evacuation and backfilling with N_2 three times. Then the reaction mixture was stirred for 16 h at 70 °C. After 16 h, the reaction mixture was cooled to room temperature followed by the addition of Py/Ac2O (1:1, 30 mL). The solution was further stirred for 24 h, then water (15 mL) was added slowly at 0 °C to quench the reaction. The aqueous phase was extracted twice with DCM (30 mL) and the combined organic layers were washed twice with aqueous 2 M HCl (10 mL) and saturated aqueous $NaHCO_3$ (15 mL), dried over ($MgSO_4$), filtered, and concentrated in rotatory evaporator. The resulting residue was

purified by flash column chromatography on a silica gel column using a mixture of Cyclohexane/EtOAc 9/1 to afford **72** as yellow dense oil (678.0 mg, 82%).

^{1}H NMR (400 MHz, $CDCl_3$) δ 5.82 – 5.69 (m, 1H), 5.09 – 4.91 (m, 5H), 4.14 (d, *J* = 2.6 Hz, 2H), 3.18 (dd, *J* = 11.9, 4.8 Hz, 1H), 2.72 (d, *J* = 6.0 Hz, 1H), 2.63 (d, *J* = 8.6 Hz, 1H), 2.59 – 2.50 (m, 1H), 2.31 (t, *J* = 11.1 Hz, 1H), 2.08 – 2.03 (m, 5H), 2.02 – 1.98 (m, 9H), 1.44 – 1.31 (m, 4H).

^{13}C NMR (101 MHz, $CDCl_3$) δ 171.0, 170.5, 170.1, 169.8, 138.5, 114.9, 74.8, 69.6, 69.5, 61.6, 59.6, 53.0, 51.7, 33.6, 26.5, 24.2, 21.0, 20.9, 20.9, 20.8.

HRMS(ESI) m/z calcd for $C_{20}H_{32}NO_8$ $[M+H]^+$: 414.2122, found: 414.2127.

9 Ketone-alkene cross-coupling reactions

9.1 General Procedures

General Procedure for ketone-alkene coupling (GP1)

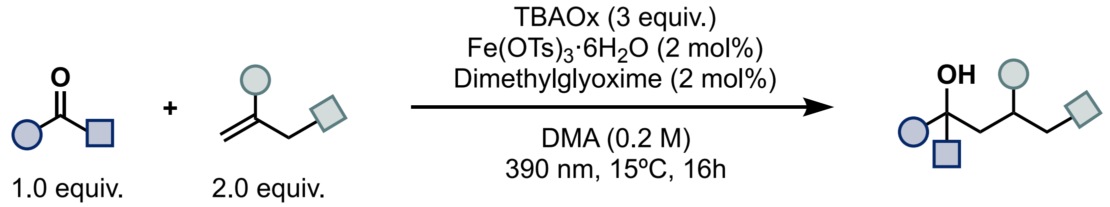

In a microwave vial equipped with a stirring bar were added dimethylglyoxime (0.5 mg, 0.004 mmol, 2 mol%), $Fe(OTs)_3{\cdot}6H_2O$ (2.6 mg, 0.004 mmol, 2 mol%), and the ketone (0.2 mmol, 1.0 equiv.) and/or the alkene (0.4 mmol, 2.0 equiv.) if solid. The vial was transferred into the glovebox and TBAOx (343 mg, 0.6 mmol, 3.0 equiv.) was charged. The tube was then capped with an aluminium crimp cap with PTFE/butyl septum and taken out of the glovebox. 1 mL of dry and degassed DMA was added to the vial via a syringe under a positive N_2 flow, followed by the ketone (0.2 mmol, 1.0 equiv.) and/or the alkene (0.4 mmol, 2.0 equiv) if liquid. The mixture was stirred under 390 nm light irradiation at 15°C for 16 h. After the reaction is completed, the solution was poured into a saturated solution of $NaHCO_3$, which was then extracted three times with EtOAc. The combined organic phases were dried over $MgSO_4$, filtered and the solvent removed under rotatory evaporation. The crude was then purified by flash chromatography on silica.

Note: For convenience, we usually prepare a stock solution of iron and ligand.

General Procedure for alkene coupling with acetone (GP2)

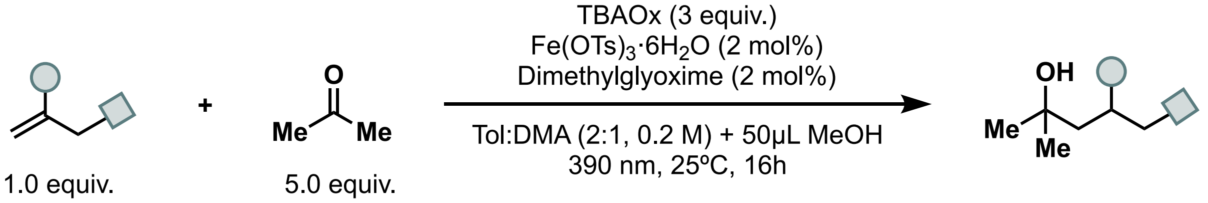

In a microwave vial equipped with a stirring bar were added dimethylglyoxime (0.5 mg, 0.004 mmol, 2 mol%), $Fe(OTs)_3{\cdot}6H_2O$ (2.6 mg, 0.004 mmol, 2 mol%), and the alkene (0.2 mmol, 1.0 equiv.) if solid. The vial was transferred into the glovebox and TBAOx (343 mg, 0.6 mmol, 3.0 equiv.) was charged. The tube was then capped with an aluminium crimp cap with PTFE/butyl septum and taken out of the glovebox. 1 mL of dry and degassed mixture Toluene/DMA (2:1) was added to the vial via a syringe

under a positive N_2 flow, followed by acetone (74 μL, 1.0 mmol, 5.0 equiv.), methanol (50 μL, 1.2 mmol, 12.0 equiv.) and the alkene (0.2 mmol, 1.0 equiv.) if liquid. The mixture was stirred under 390 nm light irradiation at 25°C for 16 h. After the reaction is completed, the solution was poured into a saturated solution of $NaHCO_3$, which was then extracted three times with EtOAc. The combined organic phases were dried over $MgSO_4$, filtered and the solvent removed under rotatory evaporation. The crude was then purified by flash chromatography on silica.

Note: For convenience, we usually prepare a stock solution of iron and ligand.

General Procedure for steroid substrates (GP3)

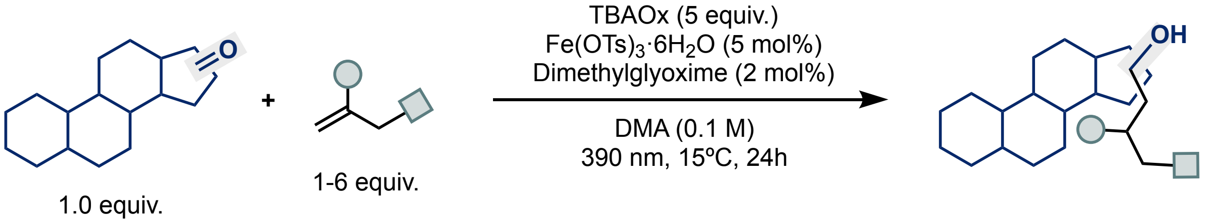

In a microwave vial equipped with a stirring bar were added dimethylglyoxime (0.2 mg, 0.002 mmol, 2 mol%), $Fe(OTs)_3{\cdot}6H_2O$ (3.4 mg, 0.005 mmol, 5 mol%), the steroid (0.1 mmol, 1.0 equiv.) and the alkene (0.1 mmol-0.6 mmol, 1.0-6.0 equiv), if solid. The vial was transferred into the glovebox and TBAOx (286 mg, 0.5 mmol, 5.0 equiv.) was charged. The tube was then capped with an aluminium crimp cap with PTFE/butyl septum and taken out of the glovebox. 1 mL of dry and degassed DMA was added to the vial via a syringe under a positive N_2 flow, followed by the alkene (0.1 mmol-0.6 mmol, 1.0-6.0 equiv) if liquid. The mixture was stirred under 390 nm light irradiation at 15°C for 24 h. After the reaction is completed, the solution was poured into a saturated solution of NH_4Cl, which was then extracted three times with EtOAc. The combined organic phases were dried over $MgSO_4$, filtered and the solvent removed under rotatory evaporation. The crude was then purified by flash chromatography on silica.

General Procedure for library synthesis of *N*-Boc-piperidinone derivatives (GP4)

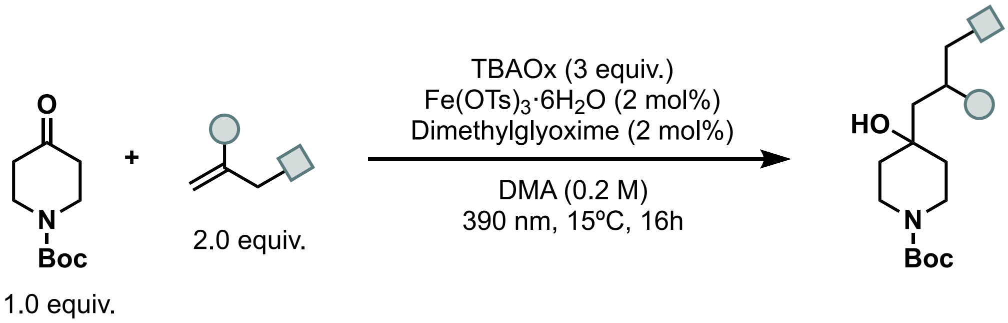

In a microwave vial equipped with a stirring bar were added dimethylglyoxime (0.2 mg, 0.002 mmol, 2 mol%), $Fe(OTs)_3{\cdot}6H_2O$ (1.4 mg, 0.002 mmol, 2 mol%), *tert*-butyl 4-oxopiperidine-1-carboxylate **58** (20 mg, 0.1 mmol, 1.0 equiv.) and the alkene (0.2 mmol, 2.0 equiv.) if solid. The vial was transferred into the glovebox and TBAOx (172 mg, 0.3 mmol, 3.0 equiv.) was charged. The tube was then capped with an aluminium crimp cap with PTFE/butyl septum and taken out of the glovebox. 1 mL of dry and degassed DMA was added to the vial via a syringe under a positive N_2 flow, followed by the alkene (0.2 mmol, 2.0 equiv.) if liquid. The mixture was stirred under 390 nm light irradiation at 15°C for 16 h. After the reaction is completed, the solution was poured into a saturated solution of $NaHCO_3$, which was then

extracted three times with EtOAc. The combined organic phases were dried over $MgSO_4$, filtered and the solvent removed under rotatory evaporation. Then, $CDCl_3$ (~0.5 mL), and CH_2Br_2 (14.0 μL, 0.2 mmol) were added and the mixture was analyzed by ^1H NMR spectroscopy to determine the NMR yield. A purified sample for characterization was obtained after flash chromatography on silica.

General Procedure for library synthesis of *N*-alkylated DNJ derivatives (GP5)

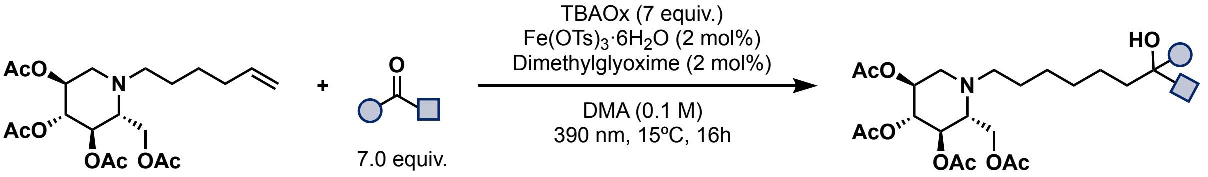

72, 1.0 equiv.

In a microwave vial equipped with a stirring bar were added dimethylglyoxime (0.2 mg, 0.002 mmol, 2 mol%), $Fe(OTs)_3{\cdot}6H_2O$ (1.4 mg, 0.002 mmol, 2 mol%), (2*R*,3*R*,4*R*,5*S*)-2-(acetoxymethyl)-1-(hex-5-en-1-yl)piperidine-3,4,5-triyl triacetate **72** (41.4 mg, 0.1 mmol, 1.0 equiv.) and the ketone (0.7 mmol, 7.0 equiv.) if solid. The vial was transferred into the glovebox and TBAOx (401 mg, 0.7 mmol, 7.0 equiv.) was charged. The tube was then capped with an aluminium crimp cap with PTFE/butyl septum and taken out of the glovebox. 1 mL of dry and degassed DMA was added to the vial via a syringe under a positive N_2 flow, followed by the ketone (0.7 mmol, 7.0 equiv.) if liquid. The mixture was stirred under 390 nm light irradiation at 15°C for 16 h. After the reaction is completed, the solution was poured into a saturated solution of $NaHCO_3$, which was then extracted three times with EtOAc. The combined organic phases were dried over $MgSO_4$, filtered and the solvent removed under rotatory evaporation. Then, $CDCl_3$ (~0.5 mL), and trichloroethylene (9.0 μL, 0.1 mmol) were added and the mixture was analyzed by ^1H NMR spectroscopy to determine the NMR yield. A purified sample for characterization was obtained after flash chromatography on silica.

9.2 Substrate scope

3-methyl-1-phenylnonan-3-ol (3)

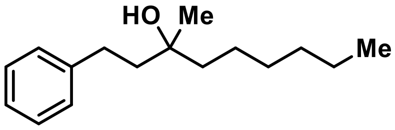

Prepared following general procedure **GP1**, starting from 4-phenylbutan-2-one (30 μL, 0.2 mmol, 1 equiv.) and 1-hexene (50 μL, 0.4 mmol, 2.0 equiv). The crude mixture was purified by flash column chromatography on silica gel using a mixture of Cyclohexane/DCM 1/1 to 1/9 to provide product **3** as a Colorless oil (35.8 mg, 77%).

^1H NMR (400 MHz, $CDCl_3$) δ 7.34 – 7.27 (m, 2H), 7.25 – 7.17 (m, 3H), 2.74 – 2.65 (m, 2H), 1.83 – 1.75 (m, 2H), 1.57 – 1.50 (m, 2H), 1.43 – 1.29 (m, 8H), 1.25 (s, 3H), 0.97 – 0.88 (m, 3H).

^{13}C NMR (101 MHz, $CDCl_3$) δ 142.8, 128.5, 128.4, 125.8, 72.8, 43.8, 42.2, 32.0, 30.5, 30.0, 27.0, 24.1, 22.7, 14.2.

HRMS(ESI) m/z calcd for $C_{16}H_{26}O$ $[M+H]^+$: 235.2051, found: 234.2004.

3-methyl-1,7-diphenylheptan-3-ol (4)

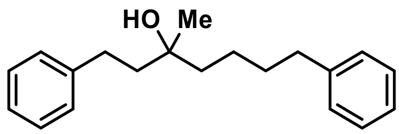

Prepared following general procedure **GP1**, starting from 4-phenylbutan-2-one (30 μL, 0.2 mmol, 1 equiv.) and 1-hexene (60 μL, 0.4 mmol, 2.0 equiv). The crude mixture was purified by flash column chromatography on silica gel using a mixture of Cyclohexane/DCM 1/1 to 1/9 to provide product **4** as a Colorless oil (36.8 mg, 65%).

^{1}H NMR (400 MHz, CDCl$_3$) δ 7.32 – 7.26 (m, 4H), 7.22 – 7.16 (m, 6H), 2.72 – 2.61 (m, 4H), 1.82 – 1.73 (m, 2H), 1.70 – 1.62 (m, 2H), 1.59 – 1.53 (m, 2H), 1.47 – 1.37 (m, 2H), 1.24 (s, 3H).

^{13}C NMR (101 MHz, CDCl$_3$) δ 142.7, 142.7, 128.5, 128.5, 128.5, 128.4, 125.9, 125.8, 72.8, 43.9, 42.0, 36.0, 32.1, 30.5, 27.1, 23.8.

HRMS(EI) m/z calcd for $C_{20}H_{24}$ [M-H$_2$O]$^+$: 264.1878, found: 264.1883.

6-methyl-8-phenyloctane-1,6-diol (5)

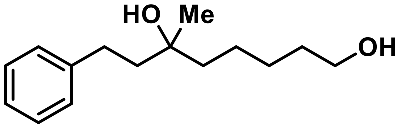

Prepared following general procedure **GP1**, starting from 4-phenylbutan-2-one (30 μL, 0.2 mmol, 1 equiv.) and 4.penten-1-ol (42 μL, 0.4 mmol, 2.0 equiv.). The crude mixture was purified by flash column chromatography on silica gel using a mixture of DCM to DCM/Acetone 7/3 to provide product **5** as a colorless oil (23.5 mg, 54%).

^{1}H NMR (400 MHz, CDCl$_3$) δ 7.36 – 7.24 (m, 2H), 7.24 – 7.15 (m, 3H), 3.63 (t, *J* = 6.6 Hz, 2H), 2.76 – 2.62 (m, 2H), 1.86 – 1.72 (m, 2H), 1.66 (s, 2H), 1.62 – 1.48 (m, 4H), 1.36 (d, *J* = 2.4 Hz, 6H), 1.23 (s, 3H).

^{13}C NMR (101 MHz, CDCl$_3$) δ 142.7, 128.5, 128.4, 72.8, 63.0, 43.8, 42.0, 32.8, 30.5, 30.1, 27.0, 25.8, 24.0.

HRMS(ESI) m/z calcd for $C_{15}H_{24}NaO_2$ [M+Na]$^+$: 259.1674, found: 259.1677.

ethyl 8-(cyclohexa-2,4-dien-1-yl)-6-hydroxy-6-methyloctanoate (6)

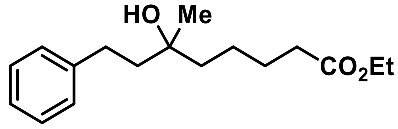

Prepared following general procedure **GP1**, starting from 4-phenylbutan-2-one (30 μL, 0.2 mmol, 1.0 equiv.) and ethyl pent-4-enoate (57 μL, 0.4 mmol, 2.0 equiv.). The crude mixture was purified by flash column chromatography on silica gel using a mixture of DCM to DCM/Acetone 95/5 to provide product **6** as a colorless oil (24.3 mg, 44%).

^{1}H NMR (400 MHz, CDCl$_3$) δ 7.31 – 7.26 (m, 2H), 7.23 – 7.15 (m, 3H), 4.13 (q, *J* = 7.1 Hz, 2H), 2.71 – 2.63 (m, 2H), 2.32 (t, *J* = 7.4 Hz, 2H), 1.79 – 1.72 (m, 2H), 1.65 (q, *J* = 7.5 Hz, 2H), 1.58 – 1.51 (m, 2H), 1.42 – 1.36 (m, 2H), 1.28 – 1.23 (m, 6H).

^{13}C NMR (101 MHz, CDCl$_3$) δ 173.8, 142.6, 128.6, 128.5, 125.9, 72.7, 60.4, 43.9, 41.8, 34.4, 30.5, 27.0, 25.6, 23.6, 14.4.

FTIR υ(cm^{-1}) 3426, 3025, 2936, 2865, 1732, 1716, 1604, 1496, 1455, 1418, 1372, 1351, 1341, 1299.

HRMS(ESI) m/z calcd for $C_{17}H_{26}NaO_3$ [M+Na]$^+$: 301.1780, found: 301.1775.

methyl (2*R*)-2-((*tert*-butoxycarbonyl)amino)-6-hydroxy-6-methyl-8-phenyloctanoate (7)

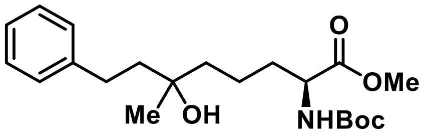

Prepared following general procedure **GP1**, starting from 4-phenylbutan-2-one (30 μL, 0.2 mmol, 1.0 equiv.) and allyl-glycine-*N*-Boc-Methylester (230 mg, 0.4 mmol, 2.0 equiv.). The crude mixture was purified by flash column chromatography on silica gel using a mixture of Cyclohexane/Et_2O from 7/3 to 3/7 to provide product **7** as a colorless oil (44.6 mg, 63%, d.r. 1.1:1).

^{1}H NMR (400 MHz, CDCl$_3$) δ 7.30 – 7.24 (m, 2H), 7.18 (dd, *J* = 7.8, 2.2 Hz, 3H), 5.17 – 5.02 (m, 1H), 4.33 (q, *J* = 7.6 Hz, 1H), 3.73 (s, 3H), 2.70 – 2.62 (m, 2H), 1.80 – 1.71 (m, 3H), 1.62 (dq, *J* = 19.0, 6.6 Hz, 2H), 1.43 (d, *J* = 3.1 Hz, 13H), 1.22 (d, *J* = 3.6 Hz, 3H)..

^{13}C NMR (101 MHz, CDCl$_3$) δ 173.5, 142.6, 142.6, 128.5, 128.4, 125.9, 80.0, 72.5, 72.4, 53.2, 53.1, 52.4, 44.2, 43.8, 41.3, 41.1, 33.4, 33.4, 33.3, 30.4, 29.8, 28.4, 28.0, 27.9, 27.0, 27.0, 26.7, 19.7, 19.7.

FTIR υ(cm^{-1}) 3370, 2973, 2951, 2932, 2868, 1739, 1697, 1508, 1497, 1455, 1438, 1391, 1365, 1249, 1213, 1160, 1122, 1049, 1024, 998, 917, 860, 742, 699.

HRMS(ESI) m/z calcd for $C_{21}H_{33}NNaO_5$ [M+Na]$^+$: 379.2360, found: 379.2316.

8-hydroxy-8-methyl-10-phenyldecanoic acid (8)

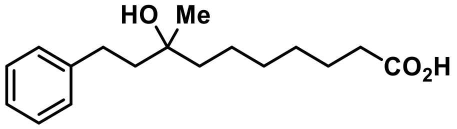

Prepared following general procedure **GP1**, starting from 4-phenylbutan-2-one (30 μL, 0.2 mmol, 1.0 equiv.), TBAOx (573 mg, 1.0 mmol, 5.0 equiv.), and hept-6-enoic acid (54 μL, 0.4 mmol, 2.0 equiv.). The work-up in this case was done using HCl 1 M. The crude mixture was purified by flash column chromatography on silica gel using a mixture of DCM to DCM/Acetone 6/4 to provide product **8** as a yellow oil (29.3 mg, 53%).

^{1}H NMR (400 MHz, CDCl$_3$) δ 7.34 – 7.26 (m, 2H), 7.22 – 7.13 (m, 3H), 6.72 (s, 1H), 2.71 – 2.63 (m, 2H), 2.35 (t, *J* = 7.5 Hz, 2H), 1.81 – 1.73 (m, 2H), 1.65 (t, *J* = 7.2 Hz, 2H), 1.55 – 1.48 (m, 2H), 1.42 – 1.30 (m, 6H), 1.24 (s, 3H).

^{13}C NMR (101 MHz, CDCl$_3$) δ 179.5, 179.4, 142.6, 128.4, 128.4, 125.8, 73.1, 43.7, 41.9, 34.1, 30.3, 29.8, 29.0, 26.8, 24.7, 23.7.

FTIR υ(cm^{-1}) 3388, 3027, 2932, 2856, 1705, 1495, 1455, 1409, 1375, 1355, 1349, 1332, 1269, 1224, 1204, 1113, 916, 742, 699.

HRMS(ESI) m/z calcd for $C_{17}H_{24}ONa$ [M+Na-2H$_2$O]$^+$: 243.1743, found: 243.1746.

9-amino-3-methyl-1-phenylnonan-3-ol (9)

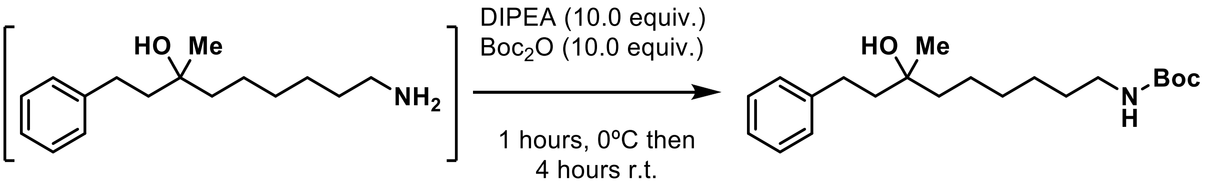

Prepared following general procedure **GP1**, starting from 4-phenylbutan-2-one (60 μL, 0.4 mmol, 2.0 equiv.), TBAOx (573 mg, 1.0 mmol, 5.0 equiv.), and hex-5-en-1-amine (25 μL, 0.2 mmol, 1.0 equiv.). Before purification di-*tert*-butyl dicarbonate (459 μL, 2.0 mmol, 10.0 equiv.), and DIPEA (348 μL, 2.0 mmol, 10.0 equiv.) were added sequentially to the reaction vial, and the mixture was stirred at 0 °C for 1 h then at r.t for another 4 h. The Boc protected target compound was purified by flash column chromatography on silica gel using a mixture of DCM to DCM/Acetone 8/2 to provide product **9** as a yellow oil (19.4 mg, 28%).

^{1}H NMR (400 MHz, $CDCl_3$) δ 7.32 – 7.26 (m, 2H), 7.22 – 7.14 (m, 3H), 4.51 (s, 1H), 3.11 (q, *J* = 6.7 Hz, 2H), 2.70 – 2.63 (m, 2H), 1.80 – 1.72 (m, 2H), 1.57 – 1.46 (m, 4H), 1.44 (s, 9H), 1.39 – 1.25 (m, 6H), 1.23 (s, 3H).

^{13}C NMR (101 MHz, $CDCl_3$) δ 156.1, 142.7, 128.5, 128.5, 125.9, 79.2, 72.8, 43.9, 42.1, 40.7, 30.5, 30.0, 28.6, 27.0, 26.9, 24.0.

FTIR υ(cm^{-1}) 3419, 3343, 2970, 2931, 2860, 1687, 1515, 1496, 1454, 1391, 1365, 1271, 1250, 1167, 742, 699.

HRMS(ESI) m/z calcd for $C_{21}H_{35}NNaO_3$ $[M+Na]^+$: 372.2509, found: 372.2512.

4-(4-hydroxy-4-methyl-6-phenylhexyl)-2-methoxyphenol (10)

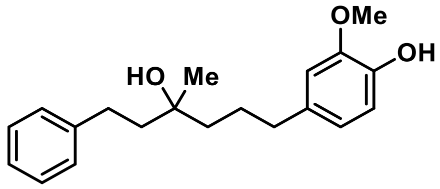

Prepared following general procedure **GP1**, starting from 4-phenylbutan-2-one (30 μL, 0.2 mmol, 1.0 equiv.), TBAOx (573 mg, 1.0 mmol, 5.0 equiv.), and eugenol (124 μL, 0.8 mmol, 4.0 equiv.). The crude mixture was purified by flash column chromatography on silica gel using a mixture of DCM to DCM/Acetone 8/2 to provide product **10** as a yellow oil (37.2 mg, 59%).

^{1}H NMR (400 MHz, $CDCl_3$) δ 7.33 – 7.27 (m, 2H), 7.23 – 7.16 (m, 3H), 6.87 – 6.83 (m, 1H), 6.70 (d, *J* = 6.9 Hz, 2H), 3.88 (s, 3H), 2.70 – 2.63 (m, 2H), 2.58 (t, *J* = 7.4 Hz, 2H), 1.82 – 1.75 (m, 2H), 1.74 – 1.63 (m, 2H), 1.61 – 1.54 (m, 2H), 1.25 (s, 3H).

^{13}C NMR (101 MHz, $CDCl_3$) δ 146.5, 143.7, 134.5, 121.0, 114.3, 111.1, 71.1, 56.0, 43.5, 36.1, 29.4, 26.7.

HRMS(ESI) m/z calcd for $C_{20}H_{26}O_3Na$ $[M+Na]^+$: 337.1774, found: 337.1776.

1-(4-hydroxy-4-methyl-6-phenylhexyl)imidazolidine-2,4-dione (11)

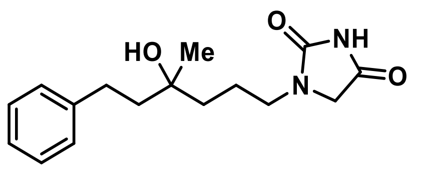

Prepared following general procedure **GP1**, starting from 4-phenylbutan-2-one (30 μL, 0.2 mmol, 1.0 equiv.), TBAOx (573 mg, 1.0 mmol, 5.0 equiv.), and 3-allylimidazolidine-2,4-dione (21 mg, 0.2 mmol, 1.0 equiv.). The crude mixture was purified by flash column chromatography on silica gel using a mixture of DCM to DCM/Acetone 8/2 and 2% MeOH to provide product **11** as a yellow oil (22.0 mg, 38%).

^{1}H NMR (400 MHz, CDCl$_3$) δ 8.81 (s, 1H), 7.31 – 7.25 (m, 2H), 7.21 – 7.13 (m, 3H), 3.89 (s, 2H), 3.37 (t, *J* = 7.1 Hz, 2H), 2.67 (td, *J* = 7.4, 3.4 Hz, 2H), 1.81 – 1.73 (m, 2H), 1.72 – 1.61 (m, 2H), 1.54 – 1.46 (m, 2H), 1.25 (s, 3H).

^{13}C NMR (101 MHz, CDCl$_3$) δ 170.8, 156.7, 142.4, 128.6, 128.4, 126.0, 72.5, 50.9, 44.1, 42.8, 38.5, 30.5, 26.8, 22.3.

HRMS(ESI) m/z calcd for $C_{16}H_{22}N_2NaO_3$ [M+Na]$^+$: 313.1523, found: 313.1524.

1-(6,7-dihydrothieno[3,2-*c*]pyridin-5(4*H*)-yl)-6-hydroxy-6-methyl-8-phenyloctan-1-one (12)

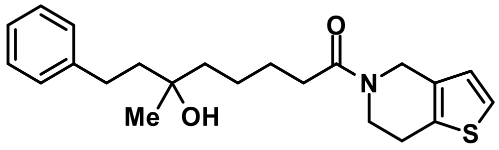

Prepared following general procedure **GP1**, starting from 4-phenylbutan-2-one (30 μL, 0.2 mmol, 1.0 equiv.), TBAOx (573 mg, 1.0 mmol, 5.0 equiv.), and thiophene derivative **S2** (88 mg, 0.8 mmol, 4.0 equiv.). The crude mixture was purified by flash column chromatography on silica gel using a mixture of Cyclohexane/EtOAc 1/1 to 8/2 to provide product **12** as a colorless oil (37.4 mg, 50%).

^{1}H NMR (400 MHz, CDCl$_3$) δ 7.29 (dt, *J* = 6.8, 1.0 Hz, 2H), 7.23 – 7.11 (m, 5H), 6.79 (t, *J* = 4.8 Hz, 1H), 4.67 (s, 1H), 4.55 (s, 1H), 3.92 (d, *J* = 2.0 Hz, 1H), 3.74 (s, 1H), 2.88 (d, *J* = 21.3 Hz, 2H), 2.72 – 2.62 (m, 2H), 2.49 – 2.39 (m, 2H), 1.81 – 1.64 (m, 5H), 1.61 – 1.52 (m, 2H), 1.51 – 1.38 (m, 3H), 1.27 – 1.23 (m, 3H).

^{13}C NMR (101 MHz, CDCl$_3$) δ 172.2, 172.1, 142.7, 134.7, 132.8, 132.2, 131.3, 128.5, 128.5, 125.9, 125.4, 124.6, 123.7, 123.6, 72.7, 45.9, 44.0, 43.6, 42.8, 41.9, 39.9, 33.9, 33.7, 30.5, 29.8, 27.0, 25.9, 25.8, 24.9, 23.9.

HRMS(ESI) m/z calcd for $C_{22}H_{30}NO_2S$ [M+H]$^+$: 372.19918, found: 370.1837.

***N*-(2-(1*H*-indol-2-yl)ethyl)-6-hydroxy-6-methyl-8-phenyloctanamide (13)**

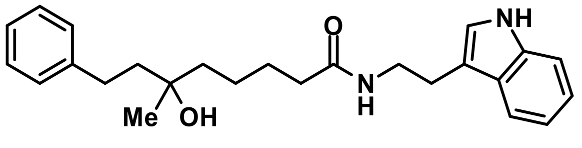

Prepared following general procedure **GP1**, starting from 4-phenylbutan-2-one (30 μL, 0.2 mmol, 1.0 equiv.), TBAOx (573 mg, 1.0 mmol, 5.0 equiv.), and **S3** (97 mg, 0.8 mmol, 4.0 equiv.). The crude mixture was purified by flash column chromatography on silica gel using a mixture of DCM to DCM/Acetone 8/2 and 2% MeOH to provide product **13** as a brown oil (32.9 mg, 42%).

^{1}H NMR (400 MHz, CDCl$_3$) δ 8.44 (s, 1H), 7.66 (d, *J* = 7.8 Hz, 1H), 7.42 (d, *J* = 8.1 Hz, 1H), 7.37 – 7.31 (m, 2H), 7.27 – 7.23 (m, 3H), 7.20 – 7.15 (m, 1H), 7.08 (s, 1H), 5.76 (s, 1H), 3.67 (q, *J* = 6.4 Hz, 2H), 3.05 – 3.00 (m, 2H), 2.75 – 2.66 (m, 2H), 2.19 (t, *J* = 7.4 Hz, 2H), 1.83 – 1.76 (m, 2H), 1.72 – 1.60 (m, 2H), 1.59 – 1.50 (m, 2H), 1.46 – 1.36 (m, 2H), 1.26 (s, 3H).
^{13}C NMR (101 MHz, CDCl$_3$) δ 173.2, 142.7, 136.6, 128.5, 128.4, 127.5, 125.9, 122.3, 122.2, 119.5, 118.8, 112.9, 111.5, 72.7, 43.9, 41.7, 39.8, 36.7, 30.5, 26.9, 26.3, 25.4, 23.6.
HRMS(ESI) m/z calcd for $C_{25}H_{32}N_2NaO_2$ [M+Na]$^+$: 415.2356, found: 415.2362.

6-hydroxy-6-methyl-8-phenyl-1-(4-(pyridin-2-yl)piperazin-1-yl)octan-1-one (14)

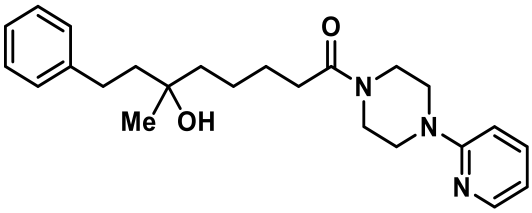

Prepared following general procedure **GP1**, starting from 4-phenylbutan-2-one (30 μL, 0.2 mmol, 1.0 equiv.), TBAOx (803 mg, 1.4 mmol, 7.0 equiv.), and **S4** (98 mg, 0.4 mmol, 2.0 equiv.). In this case, the reaction has been run for 30h. The crude mixture was purified by flash column chromatography on silica gel using a mixture of DCM to DCM/Acetone 6/4 and 2% MeOH to provide product **14** as a yellow oil (34.9 mg, 44%).
^{1}H NMR (400 MHz, CDCl$_3$) δ 8.14 (ddd, *J* = 5.1, 2.0, 0.9 Hz, 1H), 7.52 – 7.44 (m, 1H), 7.25 – 7.19 (m, 2H), 7.15 – 7.04 (m, 3H), 6.66 – 6.58 (m, 2H), 3.69 (t, *J* = 5.2 Hz, 2H), 3.66 – 3.58 (m, 2H), 3.56 – 3.50 (m, 2H), 3.44 (t, *J* = 5.4 Hz, 2H), 2.64 – 2.57 (m, 2H), 2.33 (t, 2H), 1.75 – 1.66 (m, 2H), 1.65 – 1.58 (m, 2H), 1.52 – 1.47 (m, 2H), 1.43 – 1.36 (m, 2H), 1.17 (s, 3H).
^{13}C NMR (101 MHz, CDCl$_3$) δ 171.7, 159.1, 148.0, 142.7, 137.7, 128.4, 128.4, 125.8, 113.9, 107.3, 72.4, 45.4, 45.3, 45.2, 43.9, 41.8, 41.2, 33.3, 30.4, 26.9, 25.8, 23.8.
HRMS(ESI) m/z calcd for $C_{24}H_{34}N_3O_2$ [M+H]$^+$: 396.2646, found: 396.2650.

1-butoxy-3-methyl-5-phenylpentan-3-ol (15)

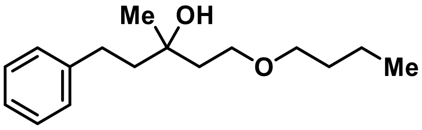

Prepared following general procedure **GP1**, starting from 4-phenylbutan-2-one (30 μL, 0.2 mmol, 1.0 equiv.), TBAOx (573 mg, 1.4 mmol, 5.0 equiv.), and 1-(vinyloxy)butane (89 μL, 0.8 mmol, 4.0 equiv.). The crude mixture was purified by flash column chromatography on silica gel using a mixture of Hexane to Hexane/ EtOAc 8/2 and 10% Acetone to provide product **15** as a colorless oil (21.5 mg, 43%).
^{1}H NMR (400 MHz, CDCl$_3$) δ 7.36 – 7.28 (m, 2H), 7.27 – 7.17 (m, 3H), 3.79 – 3.66 (m, 2H), 3.49 (t, *J* = 6.5 Hz, 2H), 2.84 – 2.66 (m, 2H), 1.95 – 1.75 (m, 4H), 1.66 – 1.55 (m, 2H), 1.48 – 1.36 (m, 2H), 1.32 (s, 3H), 0.96 (t, *J* = 7.4 Hz, 3H).
^{13}C NMR (101 MHz, CDCl$_3$) δ 143.0, 128.5, 125.8, 72.4, 71.4, 68.1, 44.5, 39.9, 31.8, 30.5, 26.6, 19.5, 14.0
HRMS(ESI) m/z calcd for $C_{16}H_{25}O$ [M+H-H$_2$O]$^+$: 233.1900, found: 233.1901.

3-methyl-1-phenyl-5-(trimethylsilyl)pentan-3-ol (16)

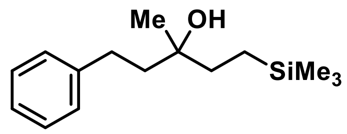

Prepared following general procedure **GP1**, starting from 4-phenylbutan-2-one (30 μL, 0.2 mmol, 1.0 equiv.), TBAOx (573 mg, 1.4 mmol, 5.0 equiv.), and trimethyl(vinyl)silane (117 μL, 0.8 mmol, 4.0 equiv.). The crude mixture was purified by flash column chromatography on silica gel using a mixture of Cyclohexane to Cyclohexane/ EtOAc 8/2 to provide product **16** as a pale-yellow oil (43.2 mg, 86%).

^{1}H NMR (400 MHz, $CDCl_3$) δ 7.31 – 7.22 (m, 2H), 7.16 (m, 3H), 2.64 (m, 2H), 1.79 – 1.70 (m, 2H), 1.50 – 1.42 (m, 2H), 1.20 (s, 3H), 0.54 – 0.40 (m, 2H), -0.01 (s, 9H).

^{13}C NMR (101 MHz, $CDCl_3$) δ 142.8, 128.5, 128.5, 125.9, 73.4, 43.0, 36.1, 30.5, 26.5, 10.3, -1.7.

HRMS(EI) m/z calcd for $C_{15}H_{24}Si$ $[M-H_2O]^+$: 232.1641, found: 232.1637.

ethyl 4-(2-hydroxy-2-methyl-4-phenylbutyl)cyclohexane-1-carboxylate (17)

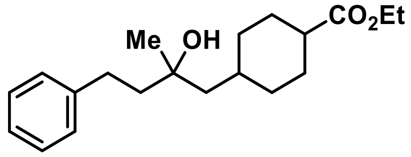

Prepared following general procedure **GP1**, starting from 4-phenylbutan-2-one (30 μL, 0.2 mmol, 1.0 equiv.), and ethyl 4-methylenecyclohexane-1-carboxylate (70 μL, 0.4 mmol, 2.0 equiv.). The crude mixture was purified by flash column chromatography on silica gel using a mixture of DCM to DCM:Acetone 95/5 to provide product **17** as a colorless oil (45.8 mg, 72%, *d.r.* = 3:1).

^{1}H NMR (600 MHz, $CDCl_3$) δ 7.30 – 7.27 (m, 2H), 7.21 – 7.17 (m, 3H), 4.14 (q, J = 7.2 Hz, 0.4H), 4.11 (q, J = 7.1 Hz, 1.6H), 2.73 – 2.62 (m, 2H), 2.47 (tt, J = 5.7, 5.7 Hz, 0.2H), 2.21 (tt, J = 12.3, 3.5 Hz, 0.8H), 1.99 – 1.86 (m, 4H), 1.78 (dd, J = 9.7, 7.6 Hz, 2H), 1.53 – 1.46 (m, 2H), 1.45 – 1.42 (m, 3H), 1.27 – 1.25 (m, 6H), 1.08 – 0.99 (m, 2H).

^{13}C NMR (151 MHz, $CDCl_3$) δ 176.3, 175.7, 142.7, 142.6, 128.6, 128.5, 128.4, 125.9, 125.9, 125.9, 73.5, 73.3, 60.2, 49.3, 44.9, 44.7, 43.3, 34.4, 34.3, 33.3, 31.6, 31.6, 30.6, 29.8, 29.5, 29.2, 27.5, 27.3, 14.4, 14.4.

FTIR υ(cm^{-1}) 3445, 2978, 2929, 2868, 1730, 1692, 1666, 1425, 1365, 1313, 1277, 1262, 1246, 1172, 1154, 1041, 962, 864, 766.

HRMS(ESI) m/z calcd for $C_{20}H_{30}O_3Na$ $[M+Na]^+$: 341.2087, found: 341.2088.

tert-butyl 4-(2-hydroxy-2-methyl-4-phenylbutyl)piperidine-1-carboxylate (18)

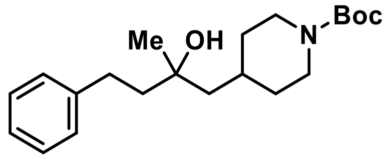

Prepared following general procedure **GP1**, starting from 4-phenylbutan-2-one (30 μL, 0.2 mmol, 1.0 equiv.), and *tert*-butyl 4-methylenepiperidine-1-carboxylate (79 μL, 0.4 mmol, 2.0 equiv.). The crude

mixture was purified by flash column chromatography on silica gel using a mixture of DCM to DCM/Acetone 9/1 to provide product **18** as a colorless oil (43.8 mg, 63%).

^{1}H NMR (400 MHz, CDCl$_3$) δ 7.31 – 7.26 (m, 2H), 7.21 – 7.15 (m, 3H), 4.03 (d, 2H), 2.76 – 2.64 (m, 4H), 1.85 – 1.73 (m, 4H), 1.73 – 1.60 (m, 2H), 1.47 (s, 1H), 1.45 (s, 9H), 1.27 (s, 3H), 1.24 – 1.12 (m, 2H).

^{13}C NMR (101 MHz, CDCl$_3$) δ 155.0, 142.5, 128.6, 128.4, 125.9, 79.3, 73.2, 48.6, 44.9, 44.1, 34.1, 34.0, 32.1, 30.5, 28.6, 27.5.

HRMS(ESI) m/z calcd for $C_{21}H_{33}NNaO_3$ [M+Na]$^+$: 370.2353, found: 370.2355.

***tert*-butyl 3-(2-hydroxy-2-methyl-4-phenylbutyl)pyrrolidine-1-carboxylate (19)**

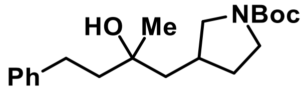

Prepared following general procedure **GP1**, starting from 4-phenylbutan-2-one (30 μL, 0.2 mmol, 1.0 equiv.) and *tert*-butyl 3-methylenepyrrolidine-1-carboxylate (75 μL, 0.4 mmol, 2.0 equiv). The crude mixture was purified by flash column chromatography on silica gel using a mixture of Cyclohexane/EtOAc 8/2 to 1/1 to provide product **19** as a colorless oil (38.2 mg, 59%, *d.r.* = 1.2:1).

^{1}H NMR (400 MHz, CDCl$_3$) δ 7.30 – 7.25 (m, 2H), 7.18 (ddt, *J* = 9.3, 6.4, 1.6 Hz, 3H), 3.65 (ddd, *J* = 11.2, 7.5, 4.2 Hz, 1H), 3.44 (ddd, *J* = 10.6, 8.4, 2.0 Hz, 1H), 3.20 (td, *J* = 10.6, 6.7 Hz, 1H), 2.88 (ddd, *J* = 10.7, 9.6, 3.3 Hz, 1H), 2.72 – 2.62 (m, 2H), 2.35 – 2.21 (m, 1H), 2.04 (ddd, *J* = 9.0, 7.1, 3.6 Hz, 2H), 1.83 – 1.75 (m, 2H), 1.70 – 1.51 (m, 3H), 1.45 (s, 9H), 1.27 (s, 3H).

^{13}C NMR (101 MHz, CDCl$_3$) δ 154.7, 142.4, 142.4, 128.5, 128.4, 125.9, 79.1, 72.6, 52.6, 52.6, 45.5, 45.5, 45.3, 45.2, 44.8, 44.6, 34.7, 34.6, 33.3, 33.3, 30.5, 30.5, 28.7, 27.4, 27.1.

HRMS(ESI) m/z calcd for $C_{20}H_{31}NNaO_3$ [M+Na]$^+$: 356.22016, found: 356.2198.

***tert*-butyl 3-(2-hydroxy-2-methyl-4-phenylbutyl)azetidine-1-carboxylate (20)**

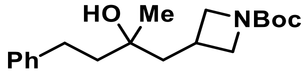

Prepared following general procedure **GP1**, starting from 4-phenylbutan-2-one (30 μL, 0.2 mmol, 1.0 equiv.) and *tert*-butyl 3-methyleneazetidine-1-carboxylate (140 μL, 0.8 mmol, 4.0 equiv). The crude mixture was purified by flash column chromatography on silica gel using a mixture of Cyclohexane/EtOAc from 9/1 to 6/4 to provide product **20** as a colorless oil (50.4 mg, 78%).

^{1}H NMR (400 MHz, CDCl$_3$) δ 7.32 – 7.26 (m, 2H), 7.19 (ddd, *J* = 6.4, 3.2, 1.8 Hz, 3H), 4.04 (td, *J* = 8.4, 2.7 Hz, 2H), 3.66 – 3.59 (m, 2H), 2.86 – 2.73 (m, 1H), 2.72 – 2.63 (m, 2H), 1.81 (dd, *J* = 7.1, 4.7 Hz, 2H), 1.77 – 1.70 (m, 2H), 1.43 (s, 10H), 1.19 (s, 3H).

^{13}C NMR (101 MHz, CDCl$_3$) δ 156.3, 142.2, 128.5, 128.4, 128.3, 125.9, 79.3, 72.7, 55.6, 46.3, 30.3, 28.4, 26.6, 24.9.

HRMS(ESI) m/z calcd for $C_{19}H_{30}NO_3$ [M+H]$^+$: 319.2148, found: 320.2194 .

methyl 3-(2-hydroxy-2-methyl-4-phenylbutyl)cyclobutane-1-carboxylate (21)

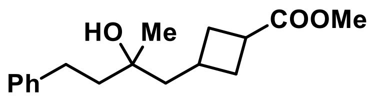

Prepared following general procedure **GP1**, starting from 4-phenylbutan-2-one (30 μL, 0.2 mmol, 1.0 equiv.), TBAOx (573 mg, 1.0 mmol, 5.0 equiv.), and methyl methyl 3-methylenecyclobutane-1-carboxylate (50 μL, 0.4 mmol, 2.0 equiv.). The crude mixture was purified by flash column chromatography on silica gel using a mixture of Cyclohexane/EtOAc 8/2 to 7/3 to provide product **21** as a colorless oil (26.2 mg, 58% *d.r.* = 1.5:1).

^{1}H NMR (400 MHz, $CDCl_3$) δ 7.33 – 7.26 (m, 2H), 7.24 – 7.17 (m, 3H), 3.69 (d, *J* = 15.6 Hz, 3H), 3.11 – 2.96 (m, 1H), 2.69 (dddd, *J* = 9.7, 7.4, 6.1, 1.8 Hz, 2H), 2.49 – 2.33 (m, 2H), 2.09 – 1.94 (m, 2H), 1.77 – 1.65 (m, 4H), 1.27 – 1.19 (m, 4H).

^{13}C NMR (101 MHz, $CDCl_3$) δ 176.9, 175.6, 142.6, 142.2, 128.5, 128.5, 128.4, 128.4, 128.4, 125.9, 125.9, 73.2, 73.0, 67.6, 51.9, 51.7, 49.0, 49.0, 44.4, 44.3, 41.0, 35.4, 35.3, 33.2, 33.1, 32.3, 32.3, 32.1, 30.4, 30.4, 29.0, 27.8, 27.1, 27.0, 23.7.

FTIR υ(cm^{-1}) 3419, 3027, 2966, 2932, 2864, 1721, 1715, 1604, 1495, 1455, 1435, 1371, 1267, 1243, 1198, 1172, 1122, 1068, 1031, 949, 920, 742, 720, 699, 506, 499.

HRMS(ESI) m/z calcd for $C_{17}H_{24}O_3Na$ [M+Na]$^+$: 299.3658, found: 299.1619.

3-(2-hydroxy-2-methyl-4-phenylbutyl)cyclobutane-1-carbonitrile (22)

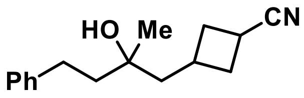

Prepared following general procedure **GP1**, starting from 4-phenylbutan-2-one (30 μL, 0.2 mmol, 1.0 equiv.), TBAOx (573 mg, 1.0 mmol, 5.0 equiv.), and 3-methylenecyclobutane-1-carbonitrile (82 μL, 0.8 mmol, 4.0 equiv.). The crude mixture was purified by flash column chromatography on silica gel using a mixture of Cyclohexane/EtOAc 7/3 to provide product **22** as a colorless oil (28.8 mg, 56%, *d.r.* = 1.1:1).

^{1}H NMR (400 MHz, $CDCl_3$) δ 7.27 (dd, *J* = 13.3, 5.9 Hz, 2H), 7.17 (d, *J* = 7.3 Hz, 3H), 3.09 – 2.86 (m, 2H), 2.69 – 2.59 (m, 2H), 2.13 (dtd, *J* = 15.7, 8.3, 2.8 Hz, 2H), 1.76 – 1.65 (m, 4H), 1.43 (d, *J* = 11.1 Hz, 1H), 1.19 (d, *J* = 4.9 Hz, 3H).

^{13}C NMR (101 MHz, $CDCl_3$) δ 142.2, 128.6, 128.4, 128.4, 128.4, 126.0, 123.6, 122.2, 73.0, 72.9, 48.7, 48.1, 44.5, 44.5, 34.8, 34.6, 33.5, 33.4, 30.4, 30.4, 30.0, 29.8, 27.0, 20.5, 19.2.

FTIR υ(cm^{-1}) 3437, 3027, 2936, 2860, 2234, 1600, 1495, 1453, 1374, 1272, 1207, 1117, 1068, 1030, 921, 743, 702.

HRMS(ESI) m/z calcd for $C_{16}H_{20}NO$ [M-H]$^-$: 242.1550, found: 242.1550.

***tert*-butyl 6-(2-hydroxy-2-methyl-4-phenylbutyl)-2-azaspiro[3.3]heptane-2-carboxylate (23)**

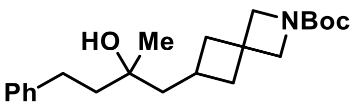

Prepared following general procedure **GP1**, starting from 4-phenylbutan-2-one (30 μL, 0.2 mmol, 1.0 equiv.) and *tert*-butyl 6-methylene-2-azaspiro[3.3]heptane-2-carboxylate (42 mg, 0.4 mmol, 2.0 equiv.).

The crude mixture was purified by flash column chromatography on silica gel using a mixture of Cyclohexane/EtOAc 8/2 to 7/3 to provide product **23** as a colorless oil (36.5 mg, 57%).

^{1}H NMR (400 MHz, CDCl$_3$) δ 7.27 (d, *J* = 6.1 Hz, 2H), 7.18 (d, *J* = 7.1 Hz, 3H), 3.94 (s, 2H), 3.76 (s, 2H), 2.66 (dd, *J* = 11.4, 6.1 Hz, 2H), 2.30 (q, *J* = 7.5 Hz, 3H), 1.83 (dd, *J* = 7.6, 4.6 Hz, 2H), 1.76 – 1.67 (m, 3H), 1.62 (d, *J* = 5.7 Hz, 2H), 1.42 (s, 9H), 1.18 (s, 3H).

^{13}C NMR (101 MHz, CDCl$_3$) δ 156.4, 142.5, 128.5, 128.4, 125.9, 79.3, 73.0, 62.6, 60.4, 49.0, 44.3, 40.9, 40.7, 34.9, 30.4, 28.5, 27.0, 26.0.

HRMS(ESI) m/z calcd for $C_{22}H_{34}NO_3$ $[M+H]^+$: 359.2463, found: 382.2355.

4,6-dimethyl-8-phenyloctane-1,6-diol (24)

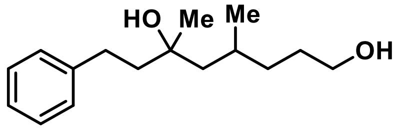

Prepared following general procedure **GP1**, starting from 4-phenylbutan-2-one (30 μL, 0.2 mmol, 1.0 equiv.) and 3-methylbut-3-en-1-ol (81 μL, 0.8 mmol, 4.0 equiv.). The crude mixture was purified by flash column chromatography on silica gel using a mixture of DCM/EtOAc from 6/4 to 1/1 to provide product **24** as a colorless oil (31.5 mg, 63%, *d.r.* = 1.8:1).

^{1}H NMR (400 MHz, CDCl$_3$) δ 7.28 (ddt, *J* = 6.9, 5.6, 1.0 Hz, 2H), 7.23 – 7.15 (m, 3H), 3.80 – 3.63 (m, 2H), 2.74 – 2.61 (m, 2H), 2.19 (s, 2H), 1.97 – 1.85 (m, 1H), 1.80 (tdd, *J* = 10.6, 6.5, 1.9 Hz, 2H), 1.74 – 1.60 (m, 1H), 1.56 (q, *J* = 6.4 Hz, 2H), 1.40 (dd, *J* = 14.5, 6.0 Hz, 1H), 1.27 (d, *J* = 6.7 Hz, 5H), 1.02 (dd, *J* = 6.8, 2.1 Hz, 3H).

^{13}C NMR (101 MHz, CDCl$_3$) δ 142.6, 142.6, 128.6, 128.6, 128.5, 128.4, 125.9, 125.9, 73.5, 73.5, 60.7, 60.6, 48.1, 47.2, 45.9, 43.9, 41.1, 40.8, 30.7, 30.5, 29.8, 28.0, 26.7, 25.2, 25.0, 23.5, 23.0.

HRMS(ESI): molecular weight peak not found despite extensive efforts.

3,5-dimethyl-1-phenylnonan-3-ol (25)

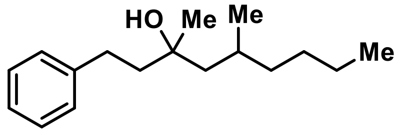

Prepared following general procedure **GP1**, starting from 4-phenylbutan-2-one (30 μL, 0.2 mmol, 1.0 equiv.) and 2-methylhex-1-ene (82 μL, 0.8 mmol, 4.0 equiv.). The crude mixture was purified by flash column chromatography on silica gel using a mixture of Cyclohexane to Cyclohexane/EtOAc 9/1 to provide product **25** as a colorless oil (24.1 mg, 49%, *d.r.* = 1.5:1).

^{1}H NMR (400 MHz, CDCl$_3$) δ 7.33 – 7.27 (m, 2H), 7.24 – 7.17 (m, 3H), 2.78 – 2.63 (m, 2H), 1.84 – 1.75 (m, 2H), 1.65 (dddd, *J* = 11.5, 6.7, 4.4, 3.3 Hz, 1H), 1.57 (ddd, *J* = 14.3, 4.0, 1.6 Hz, 1H), 1.38 (ddd, *J* = 14.2, 7.1, 2.5 Hz, 2H), 1.34 – 1.29 (m, 3H), 1.28 (s, 3H), 1.26 – 1.15 (m, 2H), 1.00 (dd, *J* = 6.6, 2.4 Hz, 3H), 0.94 – 0.89 (m, 3H).

^{13}C NMR (101 MHz, CDCl$_3$) δ 142.8, 142.8, 128.5, 128.5, 125.9, 73.5, 73.5, 49.2, 49.2, 45.1, 44.5, 38.9, 38.8, 30.6, 30.5, 29.5, 29.5, 29.0, 29.0, 27.6, 27.2, 23.1, 22.1, 22.1, 14.3.

HRMS(ESI) m/z calcd for $C_{17}H_{29}O$ $[M+H]^+$: 249.2213, found: 249.2211.

3-methyl-5-(6-methyl-7-oxabicyclo[4.1.0]heptan-3-yl)-1-phenylhexan-3-ol (26)

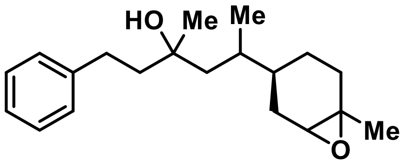

Prepared following general procedure **GP1**, starting from 4-phenylbutan-2-one (30 μL, 0.2 mmol, 1.0 equiv.) and limonene oxide cis-trans mixture (132 μL, 0.8 mmol, 4.0 equiv.). The crude mixture was purified by flash column chromatography on silica gel using a mixture of Cyclohexane/EtOAc from 9/1 to 7/3 to provide product **26** as a colorless oil (35.0 mg, 58%, *d.r.* = 2.4:2:1.4:1).

^{1}H NMR (400 MHz, CDCl$_3$) δ 7.28 (dd, *J* = 8.2, 6.8 Hz, 2H), 7.22 – 7.15 (m, 3H), 3.01 (ddd, *J* = 23.9, 4.7, 1.8 Hz, 1H), 2.69 (tt, *J* = 8.8, 5.9 Hz, 2H), 2.02 (ddt, *J* = 14.5, 11.6, 2.7 Hz, 1H), 1.95 – 1.71 (m, 4H), 1.69 – 1.50 (m, 3H), 1.49 – 1.36 (m, 1H), 1.30 (s, 3H), 1.28 – 1.15 (m, 7H), 0.93 (td, *J* = 7.0, 3.8 Hz, 3H).

^{13}C NMR (101 MHz, CDCl$_3$) δ 142.7, 142.7, 142.6, 128.5, 128.5, 128.4, 125.9, 73.3, 73.3, 73.3, 73.3, 73.3, 73.2, 73.2, 67.6, 61.3, 61.2, 59.8, 59.8, 59.7, 58.1, 58.0, 57.8, 57.8, 46.6, 46.3, 46.2, 46.0, 46.0, 45.9, 45.8, 45.2, 45.1, 44.9, 44.6, 44.6, 44.5, 44.5, 41.0, 39.7, 39.6, 35.6, 35.6, 35.5, 35.5, 32.9, 32.8, 32.7, 32.6, 32.5, 32.5, 32.3, 32.3, 32.2, 31.2, 31.0, 30.6, 30.5, 30.5, 29.6, 29.6, 29.6, 29.4, 29.4, 28.2, 28.1, 28.0, 27.9, 27.6, 27.5, 27.4, 27.2, 27.1, 27.0, 26.8, 26.7, 25.9, 25.9, 24.6, 24.6, 23.9, 23.8, 23.2, 23.2, 23.0, 22.9, 21.6, 21.5, 18.6, 18.5, 18.5, 18.4, 18.4, 18.4, 18.2.

HRMS(ESI) m/z calcd for $C_{20}H_{30}O_2Na$ [M+Na]$^+$: 325.2143, found: 325.2141.

1-(4-(2-hydroxy-2-methyl-4-phenylbutyl)piperidin-1-yl)-4,4-bis(4-hydroxyphenyl)pentan-1-one (27)

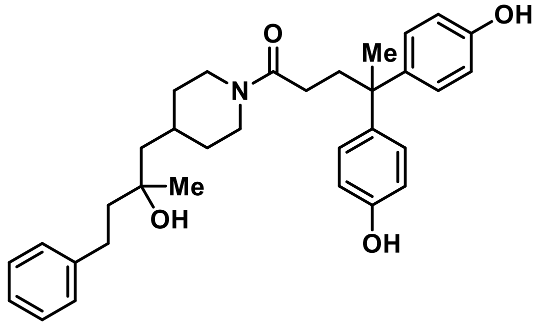

Prepared following general procedure **GP1**, starting from 4-phenylbutan-2-one (30 μL, 0.2 mmol, 1.0 equiv.). TBAOx (573 mg, 1.0 mmol, 5.0 equiv.) and diphenolic acid derivative **S6** (73 mg, 0.4 mmol, 2.0 equiv.). The crude mixture was purified by flash column chromatography on silica gel using a mixture of DCM/Acetone from 8/2 to 6/4 to provide product **27** as a colorless oil (52.7 mg, 51%).

^{1}H NMR (400 MHz, DMSO-*d$_6$*) δ 9.17 (s, 2H), 7.25 (t, *J* = 7.5 Hz, 2H), 7.21 – 7.10 (m, 3H), 6.96 (d, *J* = 8.4 Hz, 4H), 6.67 (d, *J* = 8.3 Hz, 4H), 4.29 (d, *J* = 12.9 Hz, 1H), 2.85 (t, *J* = 12.8 Hz, 1H), 2.59 (td, *J* = 7.5, 4.8 Hz, 2H), 2.54 – 2.43 (m, 1H), 2.26 – 2.15 (m, 2H), 2.10 – 1.95 (m, 3H), 1.92 (s, 3H), 1.78 – 1.59 (m, 4H), 1.49 (s, 3H), 1.35 – 1.29 (m, 2H), 1.25 – 1.14 (m, 1H), 1.13 (s, 3H), 0.97 (t, *J* = 11.3 Hz, 2H).

^{13}C NMR (101 MHz, DMSO-*d$_6$*) δ 172.0, 170.4, 155.0, 143.0, 139.6, 128.3, 128.2, 127.8, 125.5, 114.7, 71.1, 59.8, 47.8, 45.2, 44.8, 44.7, 44.0, 41.4, 37.1, 34.4, 34.3, 33.6, 33.6, 31.5, 30.0, 28.6, 28.6, 27.2, 27.2, 27.1, 21.1, 20.8, 14.1.

HRMS(ESI) m/z calcd for $C_{33}H_{42}NO_4$ $[M+H]^+$: 515.3043, found: 516.3116.

(4*S*)-4-(4-(4-(2-hydroxy-2-methyl-4-phenylbutyl)piperidin-1-yl)-4-oxobutyl)tetrahydro-1*H*-thieno[3,4-*d*]imidazol-2(3*H*)-one (28)

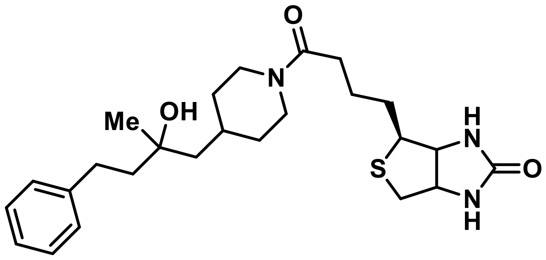

Prepared following general procedure **GP1**, starting from 4-phenylbutan-2-one (180 μL, 1.2 mmol, 6.0 equiv.), TBAOx (573 mg, 1.0 mmol, 5.0 equiv.), and Biotine derivative **S7** (62 mg, 0.2 mmol, 1.0 equiv.). The crude mixture was directly purified by flash column chromatography without workup on silica gel using a mixture of 2% to 10% MeOH in DCM to provide product **28** as a colorless oil (20.3 mg, 22%).

^{1}H NMR (400 MHz, MeOH-*d4*) δ [7.23 – 7.10 (m, 2H), 7.12 – 7.04 (m, 2H), 7.08 – 6.97 (m, 1H), 4.43 – 4.36 (m, 1H), 4.33 (d, J = 12.5 Hz, 1H), 4.20 (dd, J = 7.9, 4.5 Hz, 1H), 3.85 – 3.76 (m, 1H), 3.18 – 2.93 (m, 2H), 2.83 (dd, J = 12.7, 5.0 Hz, 1H), 2.79 – 2.66 (m, 1H), 2.64 – 2.48 (m, 3H), 2.39 – 2.28 (m, 1H), 2.32 – 2.22 (m, 1H), 1.93 – 1.44 (m, 7H), 1.37 (d, J = 5.8 Hz, 3H), 1.32 (dd, J = 14.8, 7.4 Hz, 1H), 1.16 (s, 3H), 1.16 – 1.00 (m, 1H).

^{13}C NMR (101 MHz, MeOH-*d4*) δ 173.6, 166.1, 163.8, 144.1, 129.4, 129.4, 129.3, 127.0, 126.7, 73.6, 63.3, 61.6, 60.7, 57.0, 46.2, 46.1, 43.3, 41.1, 35.9, 35.8, 35.1, 35.0, 33.8, 33.8, 33.2, 31.5, 30.8, 29.9, 29.9, 29.5, 29.5, 27.1, 27.0, 26.5, 24.8, 13.9.

HRMS(EI) m/z calcd for $C_{26}H_{40}N_3O_2S$ $[M+H]^+$: 474.2792, found: 474.2790.

3-(2-hydroxy-4-phenylbutan-2-yl)cyclopentan-1-ol (29)

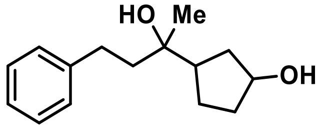

Prepared following general procedure **GP1**, starting from 4-phenylbutan-2-one (30 μL, 0.2 mmol, 1.0 equiv.), TBAOx (573 mg, 1.0 mmol, 5.0 equiv.) and cyclopent-3-en-1-ol (95 μL, 1.2 mmol, 6.0 equiv.). In this case, the reaction has been run for 72h. The crude mixture was purified by flash column chromatography on silica gel using a mixture of DCM to DCM/Acetone 6/4 to provide product **29** as a pale yellow oil (10.4 mg, 22%, *d.r.* = 2:1.5:1:1).

^{1}H NMR (400 MHz, $CDCl_3$) δ δ 7.35 – 7.26 (m, 2H), 7.25 – 7.14 (m, 3H), 4.46 – 4.31 (m, 0.35H), 4.29 – 4.22 (m, 0.5H), 2.77 – 2.61 (m, 2H), 2.46 – 2.32 (m, 0.3H), 2.24 – 2.12 (m, 0.7H), 1.91 – 1.62 (m, 9H), 1.29 – 1.21 (m, 3H).

^{13}C NMR (101 MHz, $CDCl_3$) δ δ 142.7, 142.6, 128.6, 128.5, 128.5, 126.0, 125.9, 125.9, 125.9, 74.0, 73.8, 73.5, 73.4, 73.3, 46.7, 46.7, 46.4, 44.5, 43.7, 43.6, 43.4, 37.0, 36.5, 36.3, 36.2, 36.2, 35.9, 35.4, 35.4, 31.0, 30.9, 30.6, 30.5, 26.3, 25.1, 24.9, 24.8, 24.8, 24.3, 24.2, 23.8.

HRMS(EI) m/z calcd for $C_{15}H_{20}O$ $[M]^+$: 216.1517, found: 216.1512.

1-(4-phenylbutyl)cyclohexan-1-ol (30)

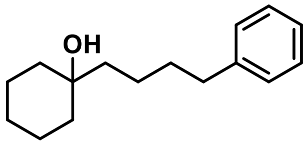

Prepared following general procedure **GP1**, starting from cyclohexanone (21 μL, 0.2 mmol, 1.0 equiv.) and 4-phenyl-1-butene (60 μL, 0.4 mmol, 2.0 equiv.). The crude mixture was purified by flash column chromatography on silica gel using a mixture of Cyclohexane/EtOAc 9/1 to 8/2 to provide product **30** as a colorless oil (23.5 mg, 62%).

^{1}H NMR (400 MHz, CDCl$_3$) δ 7.20 (t, *J* = 7.6 Hz, 2H), 7.10 (d, *J* = 7.2 Hz, 3H), 2.55 (t, *J* = 7.8 Hz, 2H), 1.52 – 1.28 (m, 15H).

^{13}C NMR (101 MHz, CDCl$_3$) δ 142.8, 128.5, 128.5, 128.4, 125.7, 71.5, 42.4, 37.5, 36.1, 32.2, 26.0, 22.7, 22.4.

HRMS(EI) m/z calcd for $C_{16}H_{22}$ $[M+H-H_2O]^+$: 214.1719, found: 214.1724.

1-(4-phenylbutyl)cyclopentan-1-ol (31)

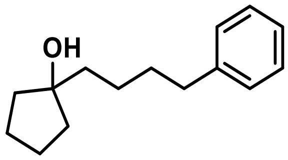

Prepared following general procedure **GP1**, starting from pentanone (19 μL, 0.2 mmol, 1.0 equiv.), TBAOx (573 mg, 1.0 mmol, 5.0 equiv.), and 4-phenyl-1-butene (180 μL, 1.2 mmol, 6.0 equiv.). In this case, the reaction has been run for 48h. The crude mixture was purified by flash column chromatography on silica gel using a mixture of Cyclohexane/EtOAc 95/5 to 7/3 to provide product **31** as a colorless oil (31.4 mg, 72%).

^{1}H NMR (400 MHz, CDCl$_3$) δ 7.30 – 7.24 (m, 2H), 7.19 – 7.14 (m, 3H), 2.67 – 2.60 (m, 2H), 1.87 – 1.73 (m, 2H), 1.69 – 1.51 (m, 9H), 1.51 – 1.43 (m, 3H).

^{13}C NMR (101 MHz, CDCl$_3$) δ 142.8, 128.5, 128.4, 125.8, 82.7, 41.5, 39.8, 36.1, 32.2, 24.6, 23.9.

HRMS(EI) m/z calcd for $C_{15}H_{22}O$ $[M+H]^+$: 218.1626, found: 218.1631.

8-(4-phenylbutyl)-1,4-dioxaspiro[4.5]decan-8-ol (32)

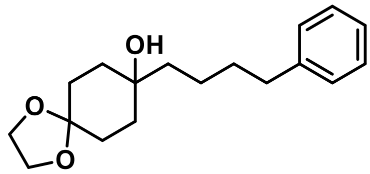

Prepared following general procedure **GP1**, starting from 1,4-dioxaspiro[4.5]decan-8-one (31 mg, 0.2 mmol, 1.0 equiv.), and 4-phenyl-1-butene (60 μL, 0.4 mmol, 2.0 equiv.). The crude mixture was purified by flash column chromatography on silica gel using a mixture of Cyclohexane/EtOAc 95/5 to 8/2 to provide product **32** as a colorless oil (23.2 mg, 40%).

^{1}H NMR (400 MHz, CDCl$_3$) δ 7.30 – 7.24 (m, 2H), 7.21 – 7.13 (m, 3H), 4.04 – 3.87 (m, 4H), 2.62 (t, *J* = 7.7 Hz, 2H), 1.89 (ddd, *J* = 12.3, 10.3, 5.9 Hz, 2H), 1.72 – 1.54 (m, 8H), 1.54 – 1.46 (m, 2H), 1.46 – 1.38 (m, 2H).

^{13}C NMR (101 MHz, CDCl$_3$) δ 142.7, 128.5, 128.4, 125.8, 109.0, 70.6, 64.4, 64.3, 42.6, 36.0, 34.8, 32.1, 30.6, 23.0.

HRMS(ESI) m/z calcd for $C_{18}H_{25}O_2$ [M+H-H_2O]$^+$: 273.1849, found:273.1850.

1-(4-phenylbutyl)cyclohexane-1,4-diol (33)

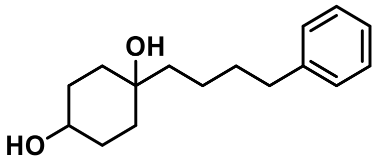

Prepared following general procedure **GP1**, starting from 4-hydroxycyclohexanone (21 μL, 0.2 mmol, 1.0 equiv.) and 4-phenyl-1-butene (60 μL, 0.4 mmol, 2.0 equiv.). The crude mixture was purified by flash column chromatography on silica gel using a mixture of DCM/Acetone 9/1 to 6/4 to provide product **33** (diastereoisomer 1: 10.5 mg, diastereoisomer 2: 12.7 mg, 52%).

Diastereoisomer 1: yellow oil

^{1}H NMR (400 MHz, CDCl$_3$) δ 7.23 – 7.17 (m, 2H), 7.13 – 7.07 (m, 3H), 3.85 (t, *J* = 3.4 Hz, 1H), 2.56 (t, *J* = 7.7 Hz, 2H), 1.80 (ddd, *J* = 13.2, 8.8, 4.2 Hz, 2H), 1.72 – 1.63 (m, 2H), 1.62 – 1.52 (m, 2H), 1.50 – 1.28 (m, 10H)..

^{13}C NMR (101 MHz, CDCl$_3$) δ 142.7, 128.5, 128.4, 125.8, 71.4, 67.6, 41.5, 36.1, 32.8, 32.1, 29.9, 29.8, 22.7.

HRMS(EI) m/z calcd for $C_{16}H_{20}$ [M-2H_2O]$^+$: 212.1554, found: 212.1560.

Diastereoisomer 2: yellow waxy solid

^{1}H NMR (400 MHz, CDCl$_3$) δ 7.24 – 7.17 (m, 2H), 7.10 (t, *J* = 6.4 Hz, 3H), 3.51 (dt, *J* = 10.1, 5.4 Hz, 1H), 2.55 (t, *J* = 7.7 Hz, 2H), 1.74 – 1.67 (m, 2H), 1.63 – 1.50 (m, 6H), 1.35 (dddd, *J* = 22.1, 17.1, 10.7, 6.3 Hz, 6H).

^{13}C NMR (101 MHz, CDCl$_3$) δ 142.5, 128.4, 128.3, 125.7, 70.3, 70.2, 43.0, 35.9, 35.0, 32.0, 30.9, 22.9.

Melting point: 58.0 ºC.

HRMS(EI) m/z calcd for $C_{16}H_{20}$ [M-2H_2O]$^+$: 212.1560, found: 212.1565.

4,4-difluoro-1-(4-phenylbutyl)cyclohexan-1-ol (34)

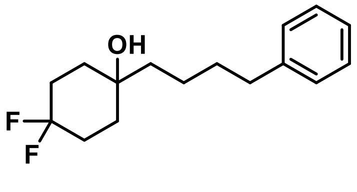

Prepared following general procedure **GP1**, starting from 4,4-difluorocyclohexanone (27 mg, 0.2 mmol, 1.0 equiv.) and 4-phenyl-1-butene (60 μL, 0.4 mmol, 2.0 equiv.). The crude mixture was purified by flash column chromatography on silica gel using a mixture of Cyclohexane/Et_2O 9/1 to 6/4 to provide product **34** as a colorless oil (31.5 mg, 58%).

^{1}H NMR (400 MHz, CDCl$_3$) δ 7.29 (dd, *J* = 8.3, 6.5 Hz, 2H), 7.18 (d, *J* = 7.6 Hz, 3H), 2.64 (t, *J* = 7.7 Hz, 2H), 2.09 (ddt, *J* = 32.7, 10.9, 5.0 Hz, 3H), 1.92 (dtd, *J* = 15.3, 7.6, 3.6 Hz, 2H), 1.71 – 1.60 (m, 6H), 1.51 (dt, *J* = 7.5, 5.3 Hz, 2H), 1.42 (tdd, *J* = 12.5, 7.9, 5.1 Hz, 2H).

^{13}C NMR (101 MHz, CDCl$_3$) δ 142.4, 128.5, 128.4, 125.9, 70.1, 70.1, 42.7, 42.7, 35.9, 33.6, 33.6, 31.9, 29.9, 29.7, 29.7, 29.5, 22.9.
^{19}F NMR (377 MHz, CDCl$_3$) δ -92.45 (d, *J* = 235 Hz), -103.93 (d, *J* = 235 Hz).
HRMS(EI) m/z calcd for $C_{16}H_{20}F_2$ [M-H$_2$O]$^+$: 250.1532, found: 250.1537.

4-(4-phenylbutyl)tetrahydro-2H-pyran-4-ol (35)

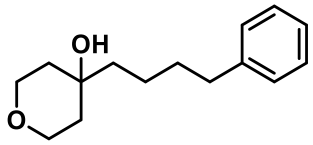

Prepared following general procedure **GP1**, starting from tetrahydro-4H-pyran-4-one (19 μL, 0.2 mmol, 1.0 equiv.) and 4-phenyl-1-butene (60 μL, 0.4 mmol, 2.0 equiv.). The crude mixture was purified by flash column chromatography on silica gel using a mixture of Cyclohexane/Acetone 9/1 to 6/4 to provide product **35** as a colorless oil (29.1 mg, 65%).
^{1}H NMR (400 MHz, CDCl$_3$) δ 7.47 – 7.40 (m, 2H), 7.34 (dd, *J* = 7.6, 5.4 Hz, 3H), 3.90 (dt, *J* = 8.8, 2.4 Hz, 4H), 2.80 (t, *J* = 7.7 Hz, 2H), 1.89 – 1.75 (m, 4H), 1.62 (ttd, *J* = 17.2, 6.9, 2.7 Hz, 6H).
^{13}C NMR (101 MHz, CDCl$_3$) δ 142.5, 128.5, 128.4, 125.8, 69.0, 64.0, 43.3, 37.7, 36.0, 32.0, 22.2.
HRMS(EI) m/z calcd for $C_{15}H_{20}O$ [M-H$_2$O]$^+$: 216.1513, found: 216.1518.

***tert*-butyl 4-hydroxy-4-(4-phenylbutyl)piperidine-1-carboxylate (36)**

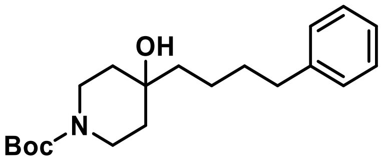

Prepared following general procedure **GP1**, starting from tert-butyl 4-oxopiperidine-1-carboxylate (40 mg, 0.2 mmol, 1.0 equiv.), and 4-phenyl-1-butene (60 μL, 0.4 mmol, 2.0 equiv.). The crude mixture was purified by flash column chromatography on silica gel using a mixture of Cyclohexane to Cyclohexane/EtOAc 6/4 to provide product **36** as a colorless oil (37.4 mg, 56%).
^{1}H NMR (400 MHz, CDCl$_3$) δ 7.30 – 7.26 (m, 2H), 7.20 – 7.15 (m, 3H), 3.79 (dt, *J* = 13.5, 4.0 Hz, 2H), 3.15 (ddd, *J* = 13.4, 9.4, 5.5 Hz, 2H), 2.63 (d, *J* = 7.6 Hz, 2H), 1.72 – 1.57 (m, 4H), 1.52 – 1.48 (m, 4H), 1.46 (s, 9H), 1.42 – 1.36 (m, 2H).
^{13}C NMR (101 MHz, CDCl$_3$) δ 155.0, 142.5, 128.5, 128.4, 125.8, 79.5, 69.9, 43.1, 36.8, 36.0, 32.0, 28.6, 22.5.
HRMS (EI) m/z calcd for $C_{20}H_{31}NO_3$ [M+]$^+$: 333.2293, found: 333.2287.

***tert*-butyl 4-hydroxy-4-(4-phenylbutyl)azepane-1-carboxylate (37)**

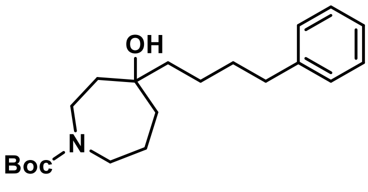

Prepared following general procedure **GP1**, starting from **S8** (43 mg, 0.2 mmol, 1.0 equiv.), TBAOx (573 mg, 1.0 mmol, 5.0 equiv.) and 4-phenyl-1-butene (60 μL, 0.4 mmol, 2.0 equiv.). The crude mixture was purified by flash column chromatography on silica gel using a mixture of DCM to DCM/Acetone 9/1 to provide product **37** as a colorless oil (39.2 mg, 56%).

^{1}H NMR (400 MHz, $CDCl_3$) δ 7.29 – 7.22 (m, 2H), 7.19 – 7.11 (m, 3H), 3.69 – 3.35 (m, 2H), 3.31 – 3.15 (m, 2H), 2.61 (t, *J* = 7.7 Hz, 2H), 2.02 – 1.89 (m, 1H), 1.73 – 1.52 (m, 7H), 1.52 – 1.32 (m, 13H).

^{13}C NMR (101 MHz, $CDCl_3$) δ 155.8, 142.6, 128.5, 128.4, 125.8, 79.3, 73.2, 46.7, 45.7, 43.9, 40.8, 40.5, 40.1, 38.3, 38.0, 36.0, 32.1, 28.6, 23.2, 21.5, 21.1.

HRMS(ESI) molecular weight peak not found despite extensive efforts.

2-(4-phenylbutyl)-1,2,3,4-tetrahydronaphthalen-2-ol (38)

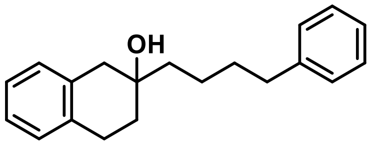

Prepared following general procedure **GP1**, starting from β-tetralone (27 μL, 0.2 mmol, 1.0 equiv.), TBAOx (573 mg, 1.0 mmol, 5.0 equiv.) and 4-phenyl-1-butene (60 μL, 0.4 mmol, 2.0 equiv.). The crude mixture was purified by flash column chromatography on silica gel using a mixture of Cyclohexane/ EtOAc 9/1 to 6/4 to provide product **38** as a yellow oil (22.0 mg, 46%).

^{1}H NMR (400 MHz, $CDCl_3$) δ 7.29 (t, *J* = 7.5 Hz, 2H), 7.23 – 7.17 (m, 3H), 7.10 (m, 4H), 3.01 (ddd, *J* = 16.2, 9.3, 6.4 Hz, 1H), 2.91 – 2.74 (m, 3H), 2.66 (t, *J* = 7.7 Hz, 2H), 1.91 – 1.73 (m, 2H), 1.74 – 1.59 (m, 4H), 1.52 (dd, *J* = 7.2, 3.1 Hz, 2H).

^{13}C NMR (101 MHz, $CDCl_3$) δ 142.7, 135.7, 134.6, 129.9, 128.8, 128.5, 128.4, 126.1, 126.0, 125.8, 71.0, 42.2, 41.4, 36.1, 33.9, 32.1, 26.3, 23.0.

HRMS(EI) m/z calcd for $C_{20}H_{22}$ $[M-H_2O]^+$: 262.1723, found: 262.1729.

***tert*-butyl 3-hydroxy-3-(4-phenylbutyl)-8-azabicyclo[3.2.1]octane-8-carboxylate (39)**

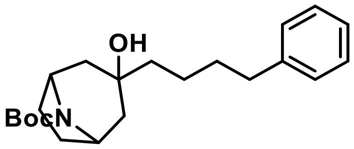

Prepared following general procedure **GP1**, starting from *tert*-Butyl 3-oxo-8-azabicyclo[3.2.1]octane-8-carboxylate (45 mg, 0.2 mmol, 1.0 equiv.) and 4-phenyl-1-butene (60 μL, 0.4 mmol, 2.0 equiv.). The crude mixture was purified by flash column chromatography on silica gel using a mixture of Cyclohexane/EtOAc 9/1 to 6/4 to provide product **39** as a colorless oil (54.5 mg, 76%).

^{1}H NMR (400 MHz, $CDCl_3$) δ 7.30 – 7.24 (m, 2H), 7.20 – 7.12 (m, 3H), 4.29 – 4.07 (m, 2H), 2.59 (dd, *J* = 8.6, 6.7 Hz, 2H), 2.12 (s, 2H), 1.87 (d, *J* = 8.6 Hz, 5H), 1.65 – 1.54 (m, 4H), 1.46 (s, 9H), 1.35 (q, *J* = 2.8 Hz, 3H).

^{13}C NMR (101 MHz, $CDCl_3$) δ 153.6, 142.5, 128.4, 128.4, 125.8, 79.2, 71.4, 53.4, 52.6, 46.9, 42.8, 42.1, 36.0, 31.9, 28.6, 28.5, 28.2, 27.6, 22.5.

HRMS(ESI) m/z calcd for $C_{22}H_{33}NNaO_3$ $[M+Na]^+$: 382.2358, found: 382.2354.

(1*r*,3*r*,5*r*,7*r*)-2-(4-phenylbutyl)adamantan-2-ol (40)

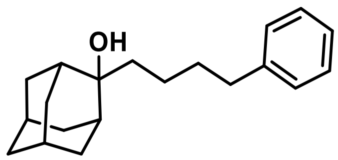

Prepared following general procedure **GP1**, starting from (1*r*,3*r*,5*r*,7*r*)-adamantan-2-one (30 mg, 0.2 mmol, 1.0 equiv.), TBAOx (573 mg, 1.0 mmol, 5.0 equiv.), and 4-phenyl-1-butene (180 μL, 1.2 mmol, 6.0 equiv.). In this case, the reaction was run for 48h. The crude mixture was purified by flash column chromatography on silica gel using a mixture of Cyclohexane/DCM 8/2 to DCM to provide product **40** as a colorless oil (37.1 mg, 66%).

^{1}H NMR (400 MHz, CDCl$_3$) δ 7.28 (dd, *J* = 7.9, 1.1 Hz, 2H), 7.20 – 7.14 (m, 3H), 2.68 – 2.60 (m, 2H), 2.16 (d, *J* = 11.7 Hz, 2H), 1.87 – 1.79 (m, 4H), 1.74 – 1.64 (m, 9H), 1.55 (d, *J* = 11.8 Hz, 3H), 1.41 (dddd, *J* = 14.6, 11.7, 6.0, 3.0 Hz, 2H).

^{13}C NMR (101 MHz, CDCl$_3$) δ 142.9, 128.5, 128.4, 125.7, 75.2, 38.5, 38.3, 37.1, 36.2, 34.7, 33.1, 32.3, 27.6, 27.4, 22.0.

HRMS(ESI) m/z calcd for $C_{20}H_{27}$ $[M+H-H_2O]^+$: 267.2107, found:267.2108.

(2*R*,5*R*)-2-methyl-1-(4-phenylbutyl)-5-(prop-1-en-2-yl)cyclohexan-1-ol (41)

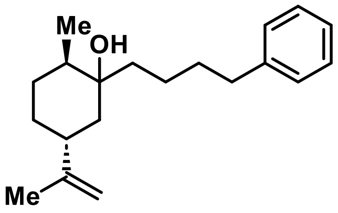

Prepared following general procedure **GP1**, starting from (+)-dihydrocarvone (31 μL, 0.2 mmol, 1.0 equiv.), and 4-phenyl-1-butene (60 μL, 0.4 mmol, 2.0 equiv.). The crude mixture was purified by flash column chromatography on silica gel using a mixture of DCM to DCM/Acetone 9/1 to provide product **41** as a colorless oil (19.5 mg, 34%).

^{1}H NMR (400 MHz, CDCl$_3$) δ 7.31 – 7.21 (m, 2H), 7.21 – 7.10 (m, 3H), 4.67 (d, *J* = 2.1 Hz, 2H), 2.61 (t, *J* = 7.8 Hz, 2H), 2.23 (tt, *J* = 12.6, 3.5 Hz, 1H), 1.70 (s, 4H), 1.65 – 1.57 (m, 2H), 1.54 – 1.44 (m, 3H), 1.43 – 1.29 (m, 4H), 1.27 – 1.07 (m, 3H), 0.85 (d, *J* = 5.9 Hz, 3H).

^{13}C NMR (101 MHz, CDCl$_3$) δ 150.5, 142.7, 128.5, 128.4, 125.8, 108.5, 73.5, 41.7, 41.4, 40.2, 37.9, 36.0, 32.3, 31.5, 30.7, 23.6, 21.2, 14.8.

HRMS(EI) m/z calcd for $C_{20}H_{30}O$ $[M]^+$: 286.2297, found:286.2291.

5-methyl-1-phenyldecan-5-ol (42)

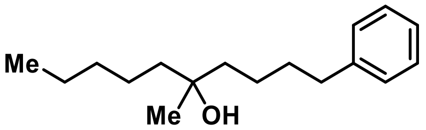

Prepared following general procedure **GP1**, starting from 2-heptenone (29 μL, 0.2 mmol, 1.0 equiv.) and 4-phenyl-1-butene (60 μL, 0.4 mmol, 2.0 equiv.). The crude mixture was purified by flash column

chromatography on silica gel using a mixture of Cyclohexane/DCM 1/1 to 7/3 to provide product **42** as a yellow oil (23.5 mg, 58%).

^{1}H NMR (400 MHz, $CDCl_3$) δ 7.29 (t, *J* = 7.5 Hz, 2H), 7.19 (d, *J* = 7.3 Hz, 3H), 2.69 – 2.61 (m, 2H), 1.65 (quin, *J* = 7.5 Hz, 2H), 1.53 – 1.40 (m, 6H), 1.35 – 1.25 (m, 6H), 1.16 (s, 3H), 0.91 (t, *J* = 6.9 Hz, 3H).

^{13}C NMR (101 MHz, $CDCl_3$) δ 142.7, 128.5, 128.5, 128.4, 125.7, 72.9, 42.0, 41.8, 36.0, 32.5, 32.2, 27.0, 23.7, 22.8, 14.2.

HRMS(ESI) m/z calcd for $C_{17}H_{28}O$ $[M]^+$: 248.2140, found: 248.2138.

4-propyldecan-4-ol (43)

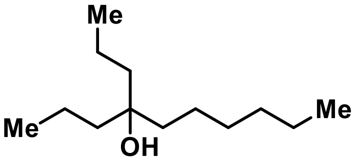

Prepared following general procedure **GP1**, starting from 4-heptenone (28 μL, 0.2 mmol, 1.0 equiv.) and 1-hexene (50 μL, 0.4 mmol, 2.0 equiv.). The crude mixture was purified by flash column chromatography on silica gel using a mixture of cyclohexane to Cyclohexane/EtOAc 9/1 to provide product **43** as a colorless oil (24.8 mg, 62%).

^{1}H NMR (400 MHz, $CDCl_3$) δ 1.43 – 1.22 (m, 18H), 0.89 (dt, *J* = 11.2, 7.0 Hz, 9H).

^{13}C NMR (101 MHz, $CDCl_3$) δ 74.7, 41.8, 39.4, 32.0, 30.1, 27.0, 23.6, 22.8, 16.9, 16.9, 14.9, 14.2.

HRMS(EI) m/z calcd for $C_{15}H_{20}O$ $[M-H_2O]^+$: 216.1513, found: 216.1518.

6-phenyl-2-(tetrahydro-2*H*-pyran-4-yl)hexan-2-ol (44)

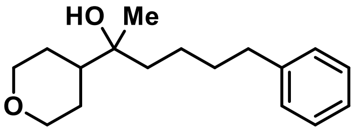

Prepared following general procedure **GP1**, starting from 1-(tetrahydro-2H-pyran-4-yl)ethan-1-one (25 μL, 0.2 mmol, 1.0 equiv.) and 4-phenyl-1-butene (120 μL, 0.8 mmol, 4.0 equiv.). The crude mixture was purified by flash column chromatography on silica gel using a mixture of DCM to DCM/Acetone 9/1 to provide product **44** as a colorless oil (32.7 mg, 62%).

^{1}H NMR (400 MHz, $CDCl_3$) δ 7.30 – 7.26 (m, 2H), 7.23 – 7.13 (m, 3H), 4.03 (dt, *J* = 11.1, 5.5 Hz, 2H), 3.35 (t, *J* = 11.6 Hz, 2H), 2.64 (t, *J* = 7.7 Hz, 2H), 1.65 – 1.38 (m, 11H), 1.10 (s, 3H).

^{13}C NMR (101 MHz, $CDCl_3$) δ 142.6, 128.4, 128.3, 125.8, 73.8, 68.5, 68.3, 44.7, 39.5, 36.0, 32.1, 27.6, 27.0, 23.8, 23.0.

HRMS(ESI) m/z calcd for $C_{17}H_{25}O$ $[M+H-H_2O]^+$: 245.1900, found: 245.1900.

4-(4-hydroxy-4-methylpentyl)-2-methoxyphenol (45)

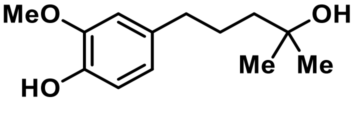

Prepared following general procedure **GP2**, starting from eugenol (31 μL, 0.2 mmol, 1.0 equiv.). The crude mixture was purified by flash column chromatography on silica gel using a mixture of DCM to DCM/Acetone 9/1 with 1% MeOH to provide product **45** as a pale yellow oil (32.4 mg, 72%).

^{1}H NMR (400 MHz, CDCl$_3$) δ 6.83 (d, *J* = 7.8 Hz, 1H), 6.68 (d, *J* = 8.5 Hz, 2H), 3.87 (s, 3H), 2.55 (t, *J* = 7.6 Hz, 2H), 1.71 – 1.62 (m, 2H), 1.54 – 1.47 (m, 2H), 1.21 (s, 6H).

^{13}C NMR (101 MHz, CDCl$_3$) δ 146.5, 143.7, 134.5, 121.0, 114.3, 111.1, 71.1, 56.0, 43.5, 36.1, 29.4, 26.7.

HRMS(ESI) m/z calcd for $C_{13}H_{20}O_3Na$ $[M+Na]^+$: 247.1305, found: 247.1311.

2-methyl-6-phenylhexan-2-ol (46)

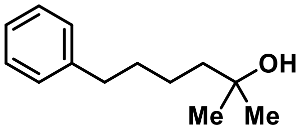

Prepared following general procedure **GP2**, starting from 4-phenylbutene (30 μL, 0.2 mmol, 1.0 equiv.). The crude mixture was purified by flash column chromatography on silica gel using a mixture of DCM to DCM/Acetone 95/5 to provide product **46** as a pale yellow oil (20.0 mg, 51%).

^{1}H NMR (400 MHz, CDCl$_3$) δ 7.31 – 7.25 (m, 2H), 7.23 – 7.13 (m, 3H), 2.68 – 2.59 (m, 2H), 1.70 – 1.58 (m, 2H), 1.54 – 1.47 (m, 2H), 1.46 – 1.38 (m, 2H), 1.38 – 1.31 (m, 1H), 1.21 (s, 6H).

^{13}C NMR (101 MHz, CDCl$_3$) δ 142.8, 128.5, 128.4, 125.8, 71.2, 43.9, 36.1, 32.2, 29.4, 24.2.

HRMS(ESI) m/z calcd for $C_{13}H_{19}$ $[M+H-H_2O]^+$: 175.1481, found: 175.1482.

***tert*-butyl 3-(2-hydroxy-2-methylpropyl)pyrrolidine-1-carboxylate (47)**

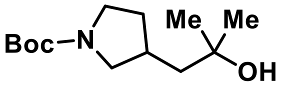

Prepared following general procedure **GP2**, starting from *tert*-butyl 3-methylenepyrrolidine-1-carboxylate (37 μL, 0.2 mmol, 1.0 equiv.) and acetone (148 μL, 2.0 mmol, 10.0 equiv.). The crude mixture was purified by flash column chromatography on silica gel using a mixture of DCM to DCM/Acetone 7/3 to provide product **47** as a pale yellow oil (19.3 mg, 40%).

^{1}H NMR (400 MHz, CDCl$_3$) δ 3.63 (dd, *J* = 10.7, 7.5 Hz, 1H), 3.42 (ddd, *J* = 10.6, 8.4, 2.0 Hz, 1H), 3.19 (td, *J* = 10.5, 6.6 Hz, 1H), 2.84 (dd, *J* = 10.7, 9.5 Hz, 1H), 2.34 – 2.17 (m, 1H), 1.67 – 1.49 (m, 4H), 1.44 (s, 9H), 1.23 (s, 6H).

^{13}C NMR (101 MHz, CDCl$_3$) δ 154.7, 79.1, 71.0, 52.6, 47.1, 45.5, 35.1, 33.3, 30.0, 28.7.

HRMS(ESI) m/z calcd for $C_{13}H_{25}NNaO_3$ $[M+Na]^+$: 266.1727, found: 266.1729.

(5R)-5-((2R,8R,8aS)-8,8a-dimethyl-1,2,3,4,6,7,8,8a-octahydronaphthalen-2-yl)-3-methyl-1-phenylhexan-3-ol (48)

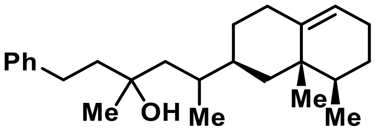

Prepared following general procedure **GP1**, starting from 4-phenylbutan-2-one (30 μL, 0.2 mmol, 1 equiv.) and (+)-valencene (70% of purity, 30% excess has been employed, 130 μL, 0.4 mmol, 2.0 equiv.). The crude mixture was purified by flash column chromatography on silica gel using a mixture of Cyclohexane/EtOAc from 9/1 to 7/3 to provide product **48** as a colorless oil (34.6 mg, 54%, *d.r.* = 1.9:1).

^{1}H NMR (400 MHz, $CDCl_3$) δ 7.32 – 7.26 (m, 2H), 7.24 – 7.16 (m, 3H), 5.32 (dt, *J* = 4.7, 2.2 Hz, 1H), 2.75 – 2.66 (m, 2H), 2.31 – 2.19 (m, 1H), 2.08 – 1.91 (m, 3H), 1.84 – 1.71 (m, 4H), 1.60 (dddd, *J* = 19.8, 9.7, 5.5, 2.8 Hz, 4H), 1.45 – 1.39 (m, 3H), 1.27 (d, *J* = 2.7 Hz, 3H), 0.99 – 0.84 (m, 10H).

^{13}C NMR (101 MHz, $CDCl_3$) δ 143.9, 142.8, 142.8, 142.7, 128.5, 128.5, 125.9, 125.9, 120.0, 119.9, 73.5, 73.5, 73.5, 46.7, 46.6, 46.5, 45.1, 44.4, 44.3, 43.6, 41.8, 41.3, 41.3, 39.9, 39.8, 39.8, 38.0, 37.8, 33.6, 33.5, 33.1, 33.0, 31.7, 30.6, 30.5, 30.0, 29.9, 27.6, 27.6, 27.3, 27.3, 27.1, 26.0, 18.7, 18.7, 18.6, 18.5, 15.9, 15.8.

HRMS(ESI) m/z calcd for $C_{25}H_{39}O$ $[M+H]^+$: 355.2995, found: 355.2998.

***tert*-butyl 3-hydroxy-3-(3-(4-hydroxy-3-methoxyphenyl)propyl)-8-azabicyclo[3.2.1]octane-8-carboxylate (49)**

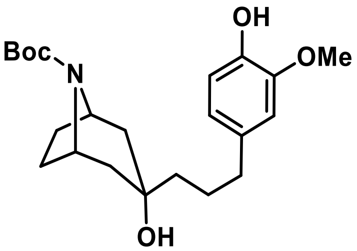

Prepared following general procedure **GP1** and starting from *tert*-butyl (1*R*,5*S*)-3-oxo-8-azabicyclo[3.2.1]octane-8-carboxylate (45 mg, 0.2 mmol, 1.0 equiv.), eugenol (124 μL, 0.8 mmol, 4.0 equiv.) and TBAOx (573 mg, 1.0 mmol, 5.0 equiv.). The crude mixture was purified by flash column chromatography on silica gel using a mixture of Cyclohexane/EtOAc 9/1 to Cyclohexane/EtOAc 4/1 to provide product **49** as a yellow oil (50.1 mg, 64%).

^{1}H NMR (400 MHz, $CDCl_3$) δ 6.80 (d, *J* = 8.1 Hz, 1H), 6.64 – 6.61 (m, 2H), 5.62 (s, 1H), 4.17 (s, 2H), 3.85 (s, 3H), 2.49 (t, *J* = 7.6 Hz, 2H), 2.10 (d, *J* = 7.1 Hz, 2H), 1.87 (dt, *J* = 9.9, 3.0 Hz, 4H), 1.67 – 1.54 (m, 4H), 1.44 (s, 9H), 1.38 – 1.31 (m, 2H).

^{13}C NMR (101 MHz, $CDCl_3$) δ 153.6, 146.5, 143.8, 134.2, 121.0, 114.3, 111.0, 79.2, 71.4, 56.0, 53.0, 46.5, 42.5, 35.9, 28.6, 27.9, 27.0, 24.9.

HRMS(ESI) m/z calcd for $C_{22}H_{33}NNaO_5$ $[M+Na]^+$: 414.2251, found: 414.2257.

ethyl 2-(2-hydroxy-2-(tetrahydro-2H-pyran-4-yl)propyl)-5-oxotetrahydro-1H-pyrrolizine-7a(5*H*)-carboxylate (50)

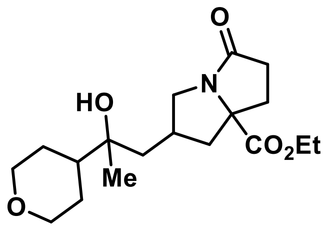

Prepared following general procedure **GP1**, starting from 1-(tetrahydro-2*H*-pyran-4-yl)ethan-1-one (25 μL, 0.2 mmol, 1.0 equiv.), ethyl 2-methylene-5-oxotetrahydro-1*H*-pyrrolizine-7a(5*H*)-carboxylate (167 mg, 0.8 mmol, 4.0 equiv.) and TBAOx (573 mg, 1.0 mmol, 5.0 equiv.). The crude mixture was purified by flash column chromatography on silica gel using a mixture of DCM to DCM/Acetone 7/3 to provide product **50** as a pale yellow oil (27.4 mg, 40 %, *d.r.* = 9:8:2:1).

^{1}H NMR (400 MHz, $CDCl_3$) δ 4.25 – 4.10 (m, 2H), 4.08 – 3.97 (m, 2H), 3.41 – 3.29 (m, 2H), 2.73 – 2.63 (m, 2H), 2.38 – 2.24 (m, 2H), 2.12 – 1.89 (m, 2H), 1.63 – 1.35 (m, 9H), 1.31 – 1.27 (m, 3H), 1.13 – 1.10 (m, 3H).

^{13}C NMR (101 MHz, $CDCl_3$) δ 177.7, 177.7, 176.2, 174.4, 172.5, 172.1, 142.1, 142.0, 127.6, 127.5, 123.1, 123.1, 74.0, 73.8, 73.8, 73.6, 73.3, 72.0, 68.4, 68.2, 62.2, 61.8, 61.8, 53.6, 50.1, 47.8, 46.2, 45.9, 45.8, 45.6, 45.3, 45.2, 44.7, 44.4, 43.7, 42.5, 39.2, 39.1, 35.6, 33.5, 33.0, 32.9, 32.9, 32.8, 28.6, 28.5, 27.9, 27.8, 27.0, 24.2, 24.0, 24.0, 23.8, 23.6, 14.3.

FTIR υ(cm^{-1}) 3400, 3208, 2924, 2849, 1732, 1686, 1671, 1456, 1446, 1436, 1404, 1393, 1378, 1340, 1312, 1258, 1242, 1196, 1160, 1117, 1092, 1035, 1017, 984, 933, 825, 792, 783, 779, 753, 745, 742, 645.

HRMS(ESI) m/z calcd for $C_{18}H_{29}NNaO_5$ $[M+Na]^+$: 362.1938, found: 362.1939.

5-methyl-5-(4-phenylbutyl)dihydrofuran-2(3H)-one (51)

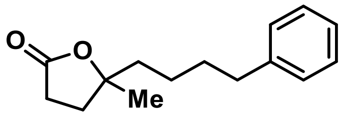

Prepared following general procedure, starting from methyl levulinate (25 μL, 0.2 mmol, 1.0 equiv.) and 4-phenyl-1-butene (60 μL, 0.4 mmol, 2.0 equiv.). The crude mixture was purified by flash column chromatography on silica gel using a mixture of Cyclohexane/Et_2O 7/3 to 4/6 to provide product **51** as a colorless oil (24.1 mg, 62%).

Mixture of rotamers.

^{1}H NMR (400 MHz, $CDCl_3$) δ 7.32 – 7.27 (m, 2H), 7.22 – 7.15 (m, 3H), 2.68 – 2.56 (m, 4H), 2.11 – 1.92 (m, 2H), 1.73 – 1.61 (m, 4H), 1.48 – 1.34 (m, 5H).

^{13}C NMR (101 MHz, $CDCl_3$) δ 178.1, 176.9, 142.6, 142.4, 142.2, 128.4, 128.4, 128.4, 128.4, 128.3,125.8, 86.9, 40.8, 38.7,35.9, 35.8, 35.7, 34.6, 33.5, 33.0, 31.7, 31.6, 29.7, 29.2, 26.1, 25.6, 23.5.

HRMS(ESI) m/z calcd for $C_{15}H_{20}O_2Na$ $[M+Na]^+$: 255.13610, found: 255.1357.

N-carbamoyl-5-(4-hydroxytetrahydro-2H-pyran-4-yl)-2-isopropylpentanamide (52)

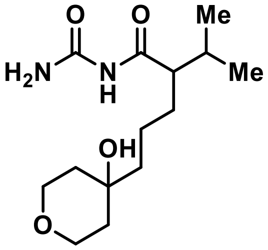

Prepared following general procedure, starting from tetrahydro-4H-pyran-4-one (80 μL, 1.0 mmol, 5.0 equiv.), TBAOx (573 mg, 1.0 mmol, 5.0 equiv.), and apronal (37 mg, 0.1 mmol, 1.0 equiv.). The crude

mixture was purified by flash column chromatography on silica gel using a mixture of DCM to 20% MeOH in DCM to provide product **52** as a yellowish oil (36.4 mg, 67%).

^{1}H NMR (400 MHz, MeOD-*d₄*) δ 3.80 – 3.61 (m, 4H), 2.16 – 2.09 (m, 1H), 1.82 (dp, *J* = 7.9, 6.6 Hz, 1H), 1.61 (dddd, *J* = 16.4, 11.1, 5.0, 2.9 Hz, 3H), 1.55 – 1.44 (m, 4H), 1.41 – 1.30 (m, 3H), 0.95 (dd, *J* = 9.4, 6.7 Hz, 6H).

^{13}C NMR (101 MHz, MeOD-*d₄*) δ 179.7, 156.7, 69.3, 64.9, 55.5, 44.2, 38.4, 38.1, 32.1, 31.2, 21.7, 21.0, 20.4.

HRMS(EI) m/z calcd for $C_{14}H_{26}N_2O_4Na$ [M+Na]$^+$: 309.1785, found: 309.1787.

***tert*-butyl 3-(2-hydroxy-4-(6-methoxynaphthalen-2-yl)-2-methylbutyl)pyrrolidine-1-carboxylate (53)**

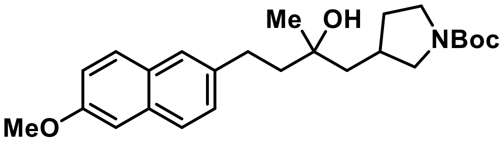

Prepared following general procedure, starting from nabumetone (45 mg, 0.2 mmol, 1.0 equiv.) and *tert*-butyl 3-methylenepyrrolidine-1-carboxylate (150 μL, 0.8 mmol, 4.0 equiv.). The crude mixture was purified by flash column chromatography on silica gel using a mixture of Cyclohexane/EtOAc 9/1 to 6/4 to provide product **53** as a brown oil (39.0 mg, 47%, *d.r.* = 1.5:1).

^{1}H NMR (400 MHz, CDCl₃) δ 7.66 (dd, *J* = 8.6, 2.3 Hz, 2H), 7.62 – 7.50 (m, 1H), 7.29 (dd, *J* = 8.4, 1.8 Hz, 1H), 7.18 – 7.08 (m, 2H), 3.89 (s, 3H), 3.67 (ddd, *J* = 11.0, 7.7, 3.4 Hz, 1H), 3.49 – 3.41 (m, 1H), 3.21 (dt, *J* = 10.7, 5.3 Hz, 1H), 2.89 (td, *J* = 10.2, 3.3 Hz, 1H), 2.84 – 2.76 (m, 2H), 2.31 (s, 1H), 1.91 – 1.81 (m, 2H), 1.73 – 1.56 (m, 3H), 1.46 (s, 10H), 1.29 (s, 3H).

^{13}C NMR (101 MHz, CDCl₃) δ 157.3, 154.7, 137.5, 137.5, 133.0, 129.2, 128.9, 127.8, 127.0, 126.1, 118.8, 105.7, 79.1, 72.7, 60.5, 55.4, 52.7, 52.6, 45.6, 45.5, 45.3, 45.3, 44.8, 44.5, 34.8, 34.6, 33.3, 30.5, 30.4, 28.7, 27.4, 27.1, 14.3.

HRMS(ESI) m/z calcd for $C_{25}H_{35}NNaO_4$ [M+Na]$^+$: 436.24638, found: 436.2462.

***tert*-butyl-4-(2-hydroxy-2-((10R,13S)-3-hydroxy-10,13-dimethyl-2,3,4,7,8,9,10,11,12,13,14,15,16,17-tetradecahydro-1H-cyclopenta[a]phenanthren-17-yl)propyl)piperidine-1-carboxylate (54)**

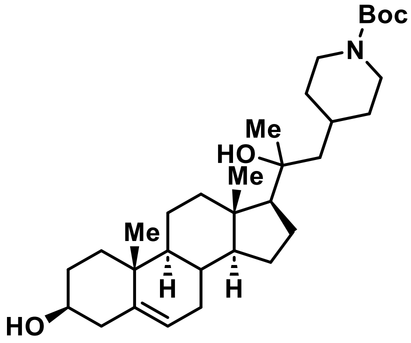

Prepared following general procedure **GP3**, starting from pregnenolone (32 mg, 0.1 mmol, 1.0 equiv.) and *tert*-butyl 4-methylenepiperidine-1-carboxylate (80 μL, 0.4 mmol, 4.0 equiv.). The crude mixture was

purified by flash column chromatography on silica gel using a mixture of DCM to DCM/Acetone 85/15 to provide product **54** as a white powder (26.3 mg, 50%, *d.r.* = 2:1)

^{1}H NMR (500 MHz, CDCl$_3$) δ 5.34 (d, J = 4.9 Hz, 1H), 4.01 (s, 2H), 3.51 (tt, J = 10.9, 4.6 Hz, 1H), 2.70 (s, 2H), 2.38 – 2.19 (m, 2H), 2.10 (dt, J = 12.6, 3.4 Hz, 1H), 2.02 – 1.92 (m, 1H), 1.89 – 1.79 (m, 3H), 1.76 – 1.47 (m, 14H), 1.46 – 1.41 (m, 11H), 1.38 – 1.22 (m, 7H), 1.22 – 1.03 (m, 7H), 1.00 (s, 3H), 0.92 (td, J = 11.3, 5.3 Hz, 1H), 0.85 (s, 3H).

^{13}C NMR (126 MHz, CDCl$_3$) δ 155.0, 140.9, 121.7, 79.3, 75.9, 71.9, 59.4, 57.0, 57.0, 50.1, 49.9, 42.9, 42.4, 40.3, 37.4, 36.6, 34.4, 34.2, 32.5, 32.05, 31.9, 31.8, 31.4, 28.6, 26.9, 23.9, 22.8, 21.0, 19.5, 13.7.

Melting point: 172.6 ºC.

HRMS(ESI) m/z calcd for $C_{32}H_{53}NNaO_4$ [M+Na]$^+$: 538.3872, found: 538.3868.

***tert*-butyl 3-((3,17-dihydroxy-13-methyl-7,8,9,11,12,13,14,15,16,17-decahydro-6H-cyclopenta[a]phenanthren-17-yl)methyl)azetidine-1-carboxylate (55)**

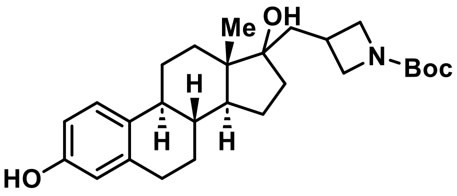

Prepared following general procedure **GP3**, starting from estrone (27 mg, 0.1 mmol, 1.0 equiv.), and *tert*-butyl 3-methyleneazetidine-1-carboxylate (120 µL, 0.6 mmol, 6.0 equiv.). The crude mixture was purified by flash column chromatography on silica gel using a mixture of DCM to DCM/Acetone 7/3 to provide product **55** as a white powder (19.0 mg, 43%) as a single diastereoisomer.

^{1}H NMR (500 MHz, CDCl$_3$) δ 7.08 (d, J = 8.4 Hz, 1H), 6.57 (dd, J = 8.4, 2.8 Hz, 1H), 6.50 (d, J = 2.7 Hz, 1H), 5.58 (s, 1H), 4.07 – 3.93 (m, 2H), 3.62 – 3.51 (m, 2H), 2.84 – 2.74 (m, 2H), 2.73 – 2.64 (m, 1H), 2.44 – 2.33 (m, 1H), 2.23 – 2.13 (m, 1H), 2.09 – 1.99 (m, 1H), 1.82 – 1.68 (m, 1H), 1.64 – 1.56 (m, 5H), 1.54 – 1.44 (m, 4H), 1.37 (s, 9H), 1.31 – 1.24 (m, 2H), 0.89 (s, 3H).

^{13}C NMR (126 MHz, CDCl$_3$) δ 156.5, 153.7, 138.4, 132.1, 127.1, 115.4, 112.9, 86.5, 79.4, 47.7, 47.4, 38.7, 37.6, 35.8, 34.2, 31.1, 30.4, 28.4, 28.3, 27.4, 25.4, 20.7, 16.4.

Melting point: 185.2 ºC.

HRMS(ESI) m/z calcd for $C_{27}H_{40}NO_4$ [M+H]$^+$: 442.2957, found: 442.2960.

(10S,13S)-3-(3-hydroxypropyl)-10,13-dimethylhexadecahydro-1H-cyclopenta[a]phenanthrene-3,17-diol (56)

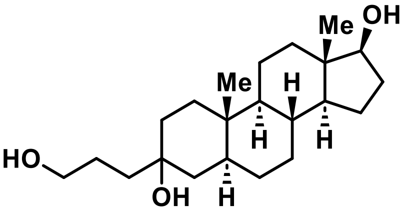

Prepared following general procedure **GP3**, starting from stanolone (29 mg, 0.1 mmol, 1.0 equiv.), and allyl alcohol (10 µL, 0.1 mmol, 1.0 equiv.). The crude mixture was purified by flash column chromatography on silica gel using a mixture of Cyclohexane/EtOAc 1/1 to Cyclohexane/EtOAc 1/9.

After the chromatography column, the afforded powder was washed with cold Et_2O to provide product **56** as a white powder (11.4 mg, 32%, *d.r.* = 3:1).

^{1}H NMR (500 MHz, MeOD-*d4*) δ 3.57 – 3.43 (m, 3H), 2.02 – 1.87 (m, 1H), 1.86 – 1.74 (m, 1H), 1.75 – 1.60 (m, 1H), 1.65 – 1.45 (m, 9H), 1.47 – 1.35 (m, 3H), 1.36 – 1.05 (m, 6H), 1.07 – 0.84 (m, 5H), 0.83 (s, 2H), 0.71 (s, 1H), 0.68 (s, 3H).

^{13}C NMR (126 MHz, MeOD-*d4*) δ 81.1, 71.8, 62.3, 54.8, 51.0, 43.5, 42.7, 40.2, 36.7, 35.9, 35.8, 35.6, 33.8, 33.2, 31.5, 31.3, 29.2, 28.4, 25.8, 25.6, 22.9, 20.5, 20.4, 11.2, 10.3, 10.1.

Melting point: 158.8 ºC.

HRMS(ESI) m/z calcd for $C_{22}H_{37}O_2$ $[M+H-H_2O]^+$ 333.2793, found: 333.2791.

***tert*-butyl 4-hydroxy-4-(5-hydroxypentyl)piperidine-1-carboxylate (59)**

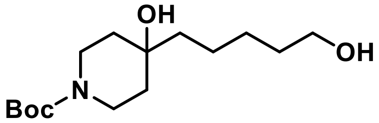

Prepared following general procedure **GP4**, starting from **58** (20 mg, 0.1 mmol, 1.0 equiv.), TBAOx (287 mg, 0.5 mmol, 5.0 equiv.) and pent-4-en-1-ol (41 µL, 0.4 mmol, 4.0 equiv.), affording **59** in 58% NMR yield. Purification by column chromatography using a mixture of Cyclohexane/EtOAc 1/3 provided an analytically pure sample for characterization.

^{1}H NMR (400 MHz, $CDCl_3$) δ 3.81 – 3.73 (m, 2H), 3.62 (t, *J* = 6.5 Hz, 2H), 3.19 – 3.09 (m, 2H), 1.99 (bs, 2H), 1.61 – 1.53 (m, 2H), 1.51 – 1.47 (m, 4H), 1.43 (s, 9H), 1.41 – 1.32 (m, 4H).

^{13}C NMR (101 MHz, $CDCl_3$) δ 155.0, 79.5, 69.8, 62.8, 43.1, 39.9, 36.7, 32.7, 28.6, 26.4, 22.6.

HRMS (ESI) m/z calcd for $C_{15}H_{29}NNaO_4$ $[M+Na]^+$: 310.1989, found 310.1990.

***tert*-butyl (*S*)-4-(4-((*tert*-butoxycarbonyl)amino)-5-methoxy-5-oxopentyl)-4-hydroxypiperidine-1-carboxylate (60)**

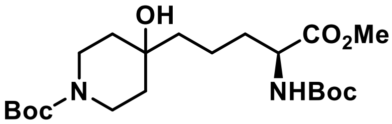

Prepared following general procedure **GP4**, starting from **58** (20 mg, 0.1 mmol, 1.0 equiv.) and *N*-(Boc)-allylglycine methyl ester (46 mg, 0.2 mmol, 2.0 equiv.) affording **60** in 37% NMR yield. Purification by column chromatography using a mixture of DCM to DCM/Acetone 85/15. A second purification was needed using a mixture of Cyclohexane/EtOAc 2/3 provided an analytically pure sample for characterization.

^{1}H NMR (400 MHz, $CDCl_3$) δ 5.07 (d, *J* = 7.8 Hz, 1H), 4.38 – 4.26 (m, 1H), 3.81 – 3.74 (m, 2H), 3.73 (s, 3H), 3.21 – 3.09 (m, 2H), 1.83 – 1.73 (m, 1H), 1.70 – 1.56 (m, 4H), 1.52 – 1.47 (m, 5H), 1.45 (s, 9H), 1.43 (s, 9H).

^{13}C NMR (101 MHz, $CDCl_3$) δ 173.5, 155.7, 155.0, 80.2, 79.5, 69.6, 53.0, 52.5, 42.1, 39.9, 37.2, 36.5, 33.4, 28.6, 28.5, 18.5.

HRMS (ESI) m/z calcd for $C_{21}H_{38}N_2NaO_7$ $[M+Na]^+$: 453.2571, found 453.2576.

***tert*-butyl 4-hydroxy-4-(3-(4-hydroxy-3-methoxyphenyl)propyl)piperidine-1-carboxylate (61)**

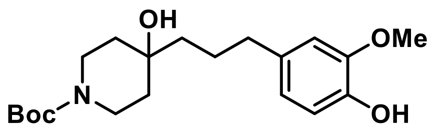

Prepared following general procedure **GP4**, starting from **58** (20 mg, 0.1 mmol, 1.0 equiv.), TBAOx (287 mg, 0.5 mmol, 5.0 equiv.) and eugenol (62 µL, 0.4 mmol, 4.0 equiv.) affording **61** in 42% NMR yield. Purification by column chromatography using a mixture of DCM to DCM/Acetone 85/15 provided an analytically pure sample for characterization.

^{1}H NMR (400 MHz, $CDCl_3$) δ 6.84 – 6.79 (m, 1H), 6.68 – 6.63 (m, 2H), 3.87 (s, 3H), 3.79 (t, *J* = 3.6 Hz, 1H), 3.76 (t, *J* = 3.4 Hz, 1H), 3.19 – 3.09 (m, 2H), 2.55 (t, *J* = 7.5 Hz, 2H), 1.72 – 1.62 (m, 2H), 1.52 – 1.47 (m, 6H), 1.45 (s, 9H).

^{13}C NMR (101 MHz, $CDCl_3$) δ 155.0, 146.5, 143.8, 134.2, 121.0, 114.3, 111.0, 79.5, 69.9, 56.0, 42.7, 39.9, 36.8, 36.0, 28.6, 25.0.

HRMS (ESI) m/z calcd for $C_{20}H_{31}NNaO_5$ $[M+Na]^+$: 388.2094, found 388.2096.

***tert*-butyl 4-hydroxy-4-(7-oxo-7-(phenylamino)heptyl)piperidine-1-carboxylate (62)**

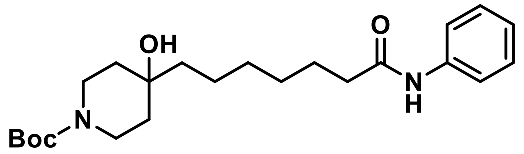

Prepared following general procedure **GP4**, starting from **58** (20 mg, 0.1 mmol, 1.0 equiv.) and *N*-phenylhept-6-enamide (41 mg, 0.2 mmol, 2.0 equiv.) affording **62** in 40% NMR yield. Purification by column chromatography using a mixture of DCM to DCM/Acetone 8/2 provided an analytically pure sample for characterization.

^{1}H NMR (400 MHz, $CDCl_3$) δ 7.51 (d, *J* = 7.6 Hz, 2H), 7.31 (t, *J* = 7.9 Hz, 2H), 7.23 (bs, 1H), 7.10 (t, *J* = 7.3 Hz, 1H), 3.83 – 3.74 (m, 2H), 3.19 – 3.10 (m, 2H), 2.35 (t, *J* = 7.1 Hz, 2H), 1.73 (quin, *J* = 7.0 Hz, 2H), 1.52 – 1.47 (m, 4H), 1.45 (s, 9H), 1.42 – 1.31 (m, 8H).

^{13}C NMR (101 MHz, $CDCl_3$) δ 171.4, 155.0, 138.0, 129.1, 124.3, 119.9, 79.5, 69.9, 43.1, 39.9, 37.9, 36.8, 29.9, 29.3, 28.6, 25.5, 22.6.

HRMS (ESI) m/z calcd for $C_{23}H_{36}N_2NaO_4$ $[M+Na]^+$: 427.2567, found 427.2570.

***tert*-butyl 4-hydroxy-4-(2-(trimethylsilyl)ethyl)piperidine-1-carboxylate (63)**

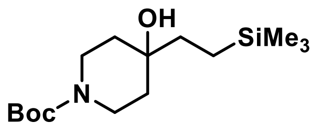

Prepared following general procedure **GP4**, starting from **58** (20 mg, 0.1 mmol, 1.0 equiv.), TBAOx (287 mg, 0.5 mmol, 5.0 equiv.) and trimethyl(vinyl)silane (59 µL, 0.6 mmol, 6.0 equiv.) affording **63** in 40% NMR yield. Purification by column chromatography using a mixture of Cyclohexane/EtOAc 3/1. A second purification was needed using a mixture of DCM/Acetone 98/2 provided an analytically pure sample for characterization.

^{1}H NMR (400 MHz, $CDCl_3$) δ 3.87 – 3.77 (m, 2H), 3.20 – 3.09 (m, 2H), 1.52 – 1.47 (m, 4H), 1.45 (s, 9H), 1.44 – 1.38 (m, 2H), 0.51 – 0.44 (m, 2H), -0.01 (s, 9H).

^{13}C NMR (101 MHz, CDCl$_3$) δ 155.0, 79.4, 70.3, 40.0, 37.2, 36.4, 28.6, 8.7, -1.7.
HRMS (ESI) m/z calcd for $C_{15}H_{31}NNaO_3Si$ [M+Na]$^+$: 324.1965, found 324.1966.

***tert*-butyl 4-(2-butoxyethyl)-4-hydroxypiperidine-1-carboxylate (64)**

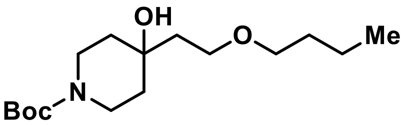

Prepared following general procedure **GP4**, starting from **58** (20 mg, 0.1 mmol, 1.0 equiv.), TBAOx (287 mg, 0.5 mmol, 5.0 equiv.) and butyl vinyl ether (52 µL, 0.4 mmol, 4.0 equiv.) affording **64** in 25% NMR yield. Purification by column chromatography using a mixture of Cyclohexane/EtOAc 3/2. A second purification was needed using a mixture of DCM/Acetone 95/5 provided an analytically pure sample for characterization.
^{1}H NMR (400 MHz, CDCl$_3$) δ 3.81 (t, J = 3.6 Hz, 1H), 3.77 (t, J = 3.5 Hz, 1H), 3.67 (t, J = 5.7 Hz, 2H), 3.43 (t, J = 6.5 Hz, 2H), 3.24 – 3.15 (m, 2H), 1.73 (t, J = 5.8 Hz, 2H), 1.65 – 1.52 (m, 4H), 1.53 – 1.46 (m, 2H), 1.45 (s, 9H), 1.39 – 1.31 (m, 2H), 0.91 (t, J = 7.4 Hz, 3H).
^{13}C NMR (101 MHz, CDCl$_3$) δ 155.1, 79.3, 71.5, 69.6, 67.4, 40.4, 39.9, 37.1, 31.8, 28.6, 19.5, 14.0.
HRMS (ESI) m/z calcd for $C_{16}H_{31}NNaO_4$ [M+Na]$^+$: 324.2145, found 324.2147.

***tert*-butyl 4-hydroxy-4-(2-(4-methylcyclohex-3-en-1-yl)propyl)piperidine-1-carboxylate (65)**

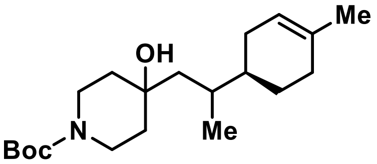

Prepared following general procedure **GP4**, starting from **58** (20 mg, 0.1 mmol, 1.0 equiv.) and *R*-(+)-limonene (118 µL, 0.8 mmol, 8.0 equiv.) affording **65** in 68% NMR yield. Purification by column chromatography using a mixture of DCM to DCM/Acetone 95/5 provided an analytically pure sample for characterization. (*d.r.* = 1.1:1).
^{1}H NMR (400 MHz, CDCl$_3$) δ 5.36 (s, 1H), 3.86 – 3.76 (m, 2H), 3.19 – 3.08 (m, 2H), 2.02 – 1.87 (m, 3H), 1.81 – 1.67 (m, 2H), 1.63 (s, 3H), 1.61 – 1.55 (m, 2H), 1.54 – 1.49 (m, 4H), 1.45 (s, 9H), 1.31 – 1.21 (m, 3H), 0.98 – 0.93 (m, 3H).
^{13}C NMR (101 MHz, CDCl$_3$) δ 155.0, 134.2, 134.2, 121.0, 120.9, 79.5, 70.6, 47.6, 47.6, 40.3, 40.2, 39.9, 39.9, 37.7, 37.6, 37.1, 37.0, 32.3, 31.9, 31.2, 30.9, 29.0, 28.6, 28.0, 26.9, 25.5, 23.6, 23.6, 19.3, 19.0.
HRMS (ESI) m/z calcd for $C_{20}H_{35}NNaO_3$ [M+Na]$^+$: 360.2509, found 360.2510.

***tert*-butyl 4-((1-(*tert*-butoxycarbonyl)piperidin-4-yl)methyl)-4-hydroxypiperidine-1-carboxylate (66)**

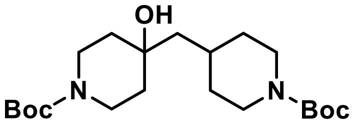

Prepared following general procedure **GP4,** starting from **58** (20 mg, 0.1 mmol, 1.0 equiv.) and *tert*-butyl 4-methylenepiperidine-1-carboxylate (40 µL, 0.2 mmol, 2.0 equiv.) affording **66** in 57% NMR yield.

Purification by column chromatography using a mixture of DCM to DCM/Acetone 9/1 provided an analytically pure sample for characterization.

^{1}H NMR (400 MHz, CDCl$_3$) δ 4.07 – 3.98 (m, 2H), 3.83 – 3.75 (m, 2H), 3.20 – 3.10 (m, 2H), 2.70 (dt, *J* = 12.9, 2.1 Hz, 2H), 1.75 – 1.69 (m, 2H), 1.68 – 1.62 (m, 1H), 1.57 – 1.51 (m, 4H), 1.45 (s, 9H), 1.44 (s, 9H), 1.40 (d, *J* = 5.5 Hz, 2H), 1.23 – 1.12 (m, 2H).

^{13}C NMR (101 MHz, CDCl$_3$) δ 155.0, 155.0, 79.6, 79.4, 70.5, 49.8, 44.1, 39.8, 37.4, 34.2, 31.3, 28.6.

HRMS (ESI) m/z calcd for $C_{21}H_{38}N_2NaO_5$ $[M+Na]^+$: 421.2673, found 421.2675.

***tert*-butyl 4-hydroxy-4-((3-(methoxycarbonyl)cyclobutyl)methyl)piperidine-1-carboxylate (67)**

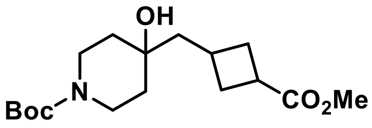

Prepared following general procedure **GP4**, starting from **58** (20 mg, 0.1 mmol, 1.0 equiv.) and methyl 3-methylenecyclobutane-1-carboxylate (25.0 µL, 0.2 mmol, 2.0 equiv.) affording **67** in 29% NMR yield. Purification by column chromatography using a mixture of Cyclohexane/EtOAc 1/1. A second purification was needed using a mixture of DCM/Acetone 4/1 provided an analytically pure sample for characterization. (*d.r.* = 4:1)

^{1}H NMR (400 MHz, CDCl$_3$) δ 3.82 – 3.75 (m, 2H), 3.69 (s, 0.6H), 3.65 (s, 2.3H), 3.16 – 3.06 (m, 2H), 3.04 – 2.95 (m, 1H), 2.51 – 2.41 (m, 1H), 2.39 – 2.30 (m, 2H), 2.01 – 1.91 (m, 2H), 1.64 (d, *J* = 7.1 Hz, 0.5H), 1.60 (d, *J* = 6.8 Hz, 1.6H), 1.53 – 1.46 (m, 4H), 1.46 (s, 9H).

^{13}C NMR (101 MHz, CDCl$_3$) δ 176.7, 175.6, 155.0, 79.6, 70.5, 70.4, 51.9, 51.8, 50.3, 50.2, 39.8, 37.0, 35.5, 35.4, 33.1, 32.3, 28.6, 28.2, 27.0.

HRMS (ESI) m/z calcd for $C_{17}H_{29}NNaO_5$ $[M+Na]^+$: 350.1938, found 350.1939.

***tert*-butyl 6-((1-(*tert*-butoxycarbonyl)-4-hydroxypiperidin-4-yl)methyl)-2-azaspiro[3.3]heptane-2-carboxylate (68)**

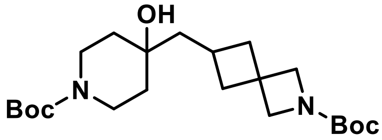

Prepared following general procedure **GP4**, starting from **58** (20 mg, 0.1 mmol, 1.0 equiv.) and **S5 (**42 mg, 0.2 mmol, 2.0 equiv.) affording **68** in 48% NMR yield. Purification by column chromatography using a mixture of Cyclohexane/EtOAc 1/1. A second purification was needed using a mixture of DCM/Acetone 4/1 provided an analytically pure sample for characterization.

^{1}H NMR (400 MHz, CDCl$_3$) δ 3.92 (s, 2H), 3.82 – 3.76 (m, 2H), 3.75 (s, 2H), 3.15 – 3.06 (m, 2H), 2.40 – 2.25 (m, 3H), 1.85 – 1.78 (m, 2H), 1.55 (d, *J* = 6.6 Hz, 2H), 1.52 – 1.46 (m, 4H), 1.44 (s, 9H), 1.41 (s, 9H).

^{13}C NMR (101 MHz, CDCl$_3$) δ 156.4, 154.9, 79.5, 79.4, 70.3, 62.5, 60.5, 50.2, 40.8, 39.8, 36.9, 35.1, 28.6, 28.5, 25.2.

HRMS (ESI) m/z calcd for $C_{22}H_{38}N_2NaO_5$ $[M+Na]^+$: 433.2673, found 433.2675.

***tert*-butyl 4-((4-(ethoxycarbonyl)cyclohexyl)methyl)-4-hydroxypiperidine-1-carboxylate (69)**

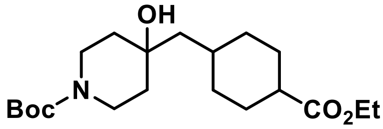

Prepared following general procedure **GP4**, starting from **58** (20 mg, 0.1 mmol, 1.0 equiv.) and ethyl 4-methylenecyclohexane-1-carboxylate (35 µL, 0.2 mmol, 2.0 equiv.) affording **69** in 46% NMR yield. Purification by column chromatography using a mixture of DCM to DCM/Acetone 9/1 provided an analytically pure sample for characterization. (*d.r.* = 2.6:1)

^{1}H NMR (400 MHz, $CDCl_3$) δ 4.15 – 4.08 (m, 2H), 3.83 – 3.73 (m, 2H), 3.20 – 3.10 (m, 2H), 2.23 – 2.14 (m, 1H), 1.98 – 1.83 (m, 4H), 1.80 – 1.63 (m, 2H), 1.62 – 1.57 (m, 1H), 1.55 – 1.50 (m, 4H), 1.45 (s, 9H), 1.37 (d, J = 5.8 Hz, 2H), 1.26 – 1.22 (m, 3H), 1.08 – 0.96 (dq, *J* = 13.2, 3.4 Hz, 2H).

^{13}C NMR (101 MHz, $CDCl_3$) δ 176.2, 175.6, 155.0, 79.5, 70.7, 70.6, 60.3, 50.5, 43.2, 39.9, 37.4, 37.3, 34.4, 32.3, 31.7, 29.8, 29.2, 28.6, 14.4.

HRMS (ESI) m/z calcd for $C_{20}H_{35}NNaO_5$ $[M+Na]^+$: 392.2407, found 392.2407.

***tert*-butyl 4-(bicyclo[2.2.1]heptan-2-yl)-4-hydroxypiperidine-1-carboxylate (70)**

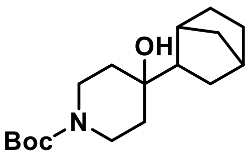

Prepared following general procedure **GP4**, starting from **58** (20 mg, 0.1 mmol, 1.0 equiv.) and norbornene (57 mg, 0.6 mmol, 6.0 equiv.) affording **70** in 43% NMR yield. Purification by column chromatography using a mixture of Cyclohexane/EtOAc 5/2. A second purification was needed using a mixture of DCM/Acetone 97/3 provided an analytically pure sample for characterization.

^{1}H NMR (400 MHz, $CDCl_3$) δ 3.92 – 3.82 (m, 2H), 3.12 – 3.00 (m, 2H), 2.27 – 2.21 (m, 2H), 1.52 – 1.47 (m, 4H), 1.45 (s, 9H), 1.43 – 1.37 (m, 2H), 1.37 – 1.32 (m, 3H), 1.31 – 1.29 (m, 1H), 1.17 – 1.11 (m, 2H), 1.10 – 1.05 (m, 1H).

^{13}C NMR (101 MHz, $CDCl_3$) δ 155.0, 79.4, 71.9, 53.1, 39.8, 39.7, 37.2, 37.0, 36.2, 35.6, 34.5, 33.2, 31.9, 28.6, 28.1.

HRMS (ESI) m/z calcd for $C_{17}H_{29}NNaO_3$ $[M+Na]^+$: 318.2040, found 318.2043.

***tert*-butyl 4-hydroxy-4-(3-(hydroxymethyl)cyclopentyl)piperidine-1-carboxylate (71)**

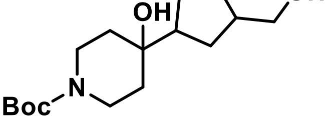

Prepared following general procedure **GP4**, starting from **58** (20 mg, 0.1 mmol, 1.0 equiv.) and cyclopent-3-en-1-ylmethanol (60 µL, 0.6 mmol, 6.0 equiv.) affording **71** in 35% NMR yield. Purification by column chromatography using a mixture of Cyclohexane/EtOAc 1/4 provided an analytically pure sample for characterization. (*d.r.* = 2:1)

^{1}H NMR (400 MHz, $CDCl_3$) δ 3.89 – 3.80 (m, 2H), 3.59 – 3.45 (m, 2H), 3.14 – 3.03 (m, 2H), 2.17 – 2.07 (m, 1H), 1.97 – 1.79 (m, 4H), 1.76 – 1.68 (m, 1H), 1.67 – 1.59 (m, 1H), 1.54 – 1.47 (m, 4H), 1.44 (s, 9H), 1.27 – 1.13 (m, 1H).

^{13}C NMR (101 MHz, CDCl$_3$) δ 155.0, 79.5, 70.9, 70.7, 67.2, 50.5, 49.5, 41.7, 41.7, 39.8, 35.9, 35.9, 35.8, 35.6, 29.6, 29.4, 28.6, 28.5, 28.5, 26.4, 24.9.

HRMS (ESI) m/z calcd for $C_{16}H_{29}NNaO_4$ [M+Na]$^+$: 322.1989, found 322.1991.

(2*R*,3*R*,4*R*,5*S*)-2-(acetoxymethyl)-1-(7-ethyl-7-hydroxynonyl)piperidine-3,4,5-triyl triacetate (73)

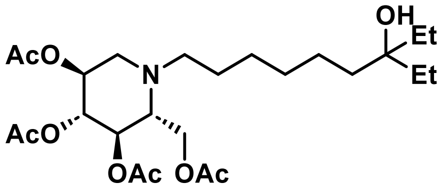

Prepared following general procedure **GP5** and starting from **72** (41 mg, 0.1 mmol, 1.0 equiv.) and pentan-3-one (74 µL, 0.7 mmol, 7.0 equiv.) affording **73** in 37% NMR yield. Purification by column chromatography on a silica gel column using a mixture of Cyclohexane/EtOAc 4/1 provided an analytically pure sample for characterization.

^{1}H NMR (400 MHz, CDCl$_3$) δ 5.05 (dt, *J* = 18.0, 9.1 Hz, 2H), 4.96 (td, *J* = 9.5, 4.8 Hz, 1H), 4.15 (t, *J* = 2.9 Hz, 2H), 3.19 (dd, *J* = 11.4, 4.9 Hz, 1H), 2.72 (ddd, *J* = 13.6, 9.7, 6.1 Hz, 1H), 2.63 (d, *J* = 8.8 Hz, 1H), 2.56 (td, *J* = 9.2, 4.7 Hz, 1H), 2.32 (t, *J* = 10.7 Hz, 1H), 2.07 (s, 3H), 2.02 (s, 6H), 2.00 (s, 3H), 1.49 – 1.35 (m, 7H), 1.30 – 1.23 (m, 7H), 0.85 (t, *J* = 7.5 Hz, 6H).

^{13}C NMR (101 MHz, CDCl$_3$) δ 171.1, 170.5, 170.2, 169.9, 74.8, 74.7, 69.6, 69.6, 61.6, 59.6, 53.0, 51.9, 38.3, 31.1, 30.2, 27.3, 24.7, 23.4, 21.0, 21.0, 20.9, 20.8, 7.9.

HRMS(ESI) m/z calcd for $C_{25}H_{44}NO_9$ [M+H]$^+$: 502.3011, found: 502.3015.

(2*R*,3*R*,4*R*,5*S*)-2-(acetoxymethyl)-1-(7-hydroxy-7-propyldecyl)piperidine-3,4,5-triyl triacetate (74)

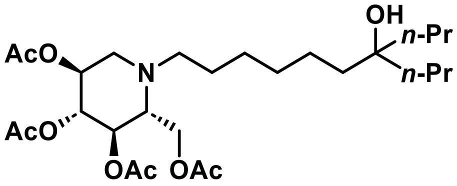

Prepared following general procedure **GP5** and starting from **72** (41 mg, 0.1 mmol, 1.0 equiv.) and heptan-4-one (98 µL, 0.7 mmol, 7.0 equiv.) affording **74** in 50% NMR yield. Purification by flash column chromatography on a silica gel column using a mixture of Cyclohexane/EtOAc 4/1 provided an analytically pure sample for characterization.

^{1}H NMR (400 MHz, CDCl$_3$) δ 5.12 – 5.00 (m, 2H), 4.97 (dd, *J* = 9.8, 4.8 Hz, 1H), 4.15 (t, *J* = 2.9 Hz, 2H), 3.19 (dd, *J* = 11.4, 5.0 Hz, 1H), 2.72 (ddd, *J* = 14.8, 9.7, 6.0 Hz, 1H), 2.63 (d, *J* = 8.9 Hz, 1H), 2.56 (td, *J* = 9.3, 4.6 Hz, 1H), 2.32 (t, *J* = 10.7 Hz, 1H), 2.07 (s, 3H), 2.02 (s, 6H), 2.00 (s, 3H), 1.42 – 1.36 (m, 8H), 1.31 – 1.23 (m, 10H), 0.91 (t, *J* = 7.1 Hz, 6H).

^{13}C NMR (101 MHz, CDCl$_3$) δ 171.1, 170.5, 170.2, 169.9, 74.8, 74.5, 69.7, 69.6, 61.6, 59.6, 53.0, 51.9, 41.8, 39.4, 30.2, 27.3, 24.7, 23.5, 21.0, 21.0, 20.9, 20.8, 16.9, 14.9.

HRMS(ESI) m/z calcd for $C_{27}H_{48}NO_9$ [M+H]$^+$: 530.3324, found: 530.3326.

(2*R*,3*R*,4*R*,5*S*)-2-(acetoxymethyl)-1-(6-(1-hydroxycyclohexyl)hexyl)piperidine-3,4,5-triyl triacetate (75)

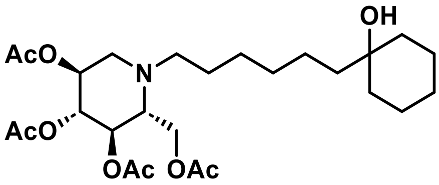

Prepared following general procedure **GP5** and starting from **72** (41 mg, 0.1 mmol, 1.0 equiv.) and cyclohexanone (72 µL, 0.7 mmol, 7.0 equiv.) affording **75** in 48% NMR yield. Purification by flash column chromatography on a silica gel column using a mixture of Cyclohexane/EtOAc 4/1 provided an analytically pure sample for characterization.

^{1}H NMR (400 MHz, CDCl$_3$) δ 5.11 – 4.99 (m, 2H), 4.96 (dt, *J* = 9.3, 4.6 Hz, 1H), 4.20 – 4.08 (m, 2H), 3.18 (dd, *J* = 11.4, 5.0 Hz, 1H), 2.71 (ddd, *J* = 13.6, 9.7, 6.1 Hz, 1H), 2.62 (d, *J* = 8.7 Hz, 1H), 2.54 (ddd, *J* = 13.9, 9.6, 4.7 Hz, 1H), 2.31 (t, *J* = 10.8 Hz, 1H), 2.06 (s, 3H), 2.01 (s, 6H), 1.99 (s, 3H), 1.59 – 1.39 (m, 10H), 1.39 – 1.22 (m, 10H).

^{13}C NMR (101 MHz, CDCl$_3$) δ 171.1, 170.5, 170.2, 169.9, 74.8, 71.5, 69.6, 69.6, 61.5, 59.6, 53.0, 51.9, 42.4, 37.6, 37.5, 30.1, 27.3, 26.0, 24.6, 22.9, 22.4, 21.0, 21.0, 20.9, 20.8.

HRMS(ESI) m/z calcd for $C_{26}H_{44}NO_9$ $[M+H]^+$: 514.3011, found: 514.3013.

(2*R*,3*R*,4*R*,5*S*)-2-(acetoxymethyl)-1-(7-hydroxy-7-methyloctyl)piperidine-3,4,5-triyl triacetate (76)

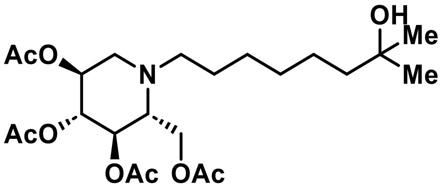

Prepared following general procedure **GP5** and starting from **72** (41 mg, 0.1 mmol, 1.0 equiv.) and acetone (52 µL, 0.7 mmol, 7.0 equiv.) affording **76** in 46% NMR yield. Purification by flash column chromatography on a silica gel column using a mixture of Cyclohexane/EtOAc 4/1 provided an analytically pure sample for characterization.

^{1}H NMR (400 MHz, CDCl$_3$) δ 5.09 – 4.98 (m, 2H), 4.97 – 4.89 (m, 1H), 4.13 (dd, *J* = 4.5, 2.7 Hz, 2H), 3.18 (dd, *J* = 11.4, 4.9 Hz, 1H), 2.75 – 2.66 (m, 1H), 2.63 – 2.59 (m, 1H), 2.53 (td, *J* = 9.2, 4.6 Hz, 1H), 2.33 – 2.25 (m, 1H), 2.05 (s, 3H), 2.00 (s, 6H), 1.98 (s, 3H), 1.45 – 1.35 (m, 4H), 1.35 – 1.21 (m, 6H), 1.18 (s, 6H).

^{13}C NMR (101 MHz, CDCl$_3$) δ 171.0, 170.5, 170.2, 169.8, 74.8, 71.0, 69.6, 69.5, 61.6, 59.6, 53.0, 51.8, 43.9, 30.0, 29.3, 29.3, 27.2, 24.7, 24.3, 21.0, 20.9, 20.8, 20.8.

HRMS(ESI) m/z calcd for $C_{23}H_{40}NO_9$ $[M+H]^+$: 474.2698, found: 474.2700.

(2*R*,3*R*,4*R*,5*S*)-2-(acetoxymethyl)-1-(7-hydroxy-7-methyldodecyl)piperidine-3,4,5-triyl triacetate (77)

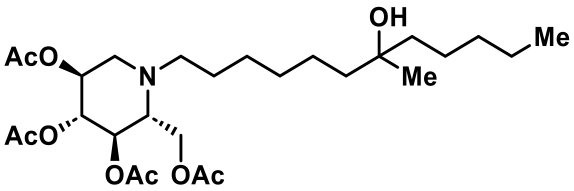

Prepared following general procedure **GP5** and starting from **72** (41 mg, 0.1 mmol, 1.0 equiv.) and heptan-2-one (100 μL, 0.7 mmol, 7.0 equiv.) affording **77** in 48% NMR yield. Purification by flash column chromatography on a silica gel column using a mixture of Cyclohexane/EtOAc 4/1 provided an analytically pure sample for characterization.

^{1}H NMR (400 MHz, CDCl$_3$) δ 5.08 – 4.98 (m, 2H), 4.97 – 4.88 (m, 1H), 4.12 (t, *J* = 3.1 Hz, 2H), 3.17 (dd, *J* = 11.4, 5.1 Hz, 1H), 2.74 – 2.65 (m, 1H), 2.60 (dt, *J* = 9.4, 2.7 Hz, 1H), 2.56 – 2.48 (m, 1H), 2.29 (dd, *J* = 11.4, 10.1 Hz, 1H), 2.05 (s, 3H), 1.99 (s, 6H), 1.98 (s, 3H), 1.43 – 1.34 (m, 6H), 1.33 – 1.20 (m, 12H), 1.11 (s, 3H), 0.92 – 0.79 (m, 3H).

^{13}C NMR (101 MHz, CDCl$_3$) δ 171.0, 170.5, 170.1, 169.8, 74.8, 72.8, 69.6, 69.5, 61.5, 59.6, 53.0, 51.8, 42.0, 41.9, 41.8, 32.5, 30.1, 27.2, 27.0, 27.0, 24.7, 23.9, 23.7, 22.7, 20.9, 20.9, 20.8, 20.8, 14.2.

HRMS(ESI) m/z calcd for $C_{27}H_{48}NO_9$ [M+H]$^+$: 530.3324, found: 530.3343.

(2*R*,3*R*,4*R*,5*S*)-2-(acetoxymethyl)-1-(6-(1-hydroxycyclopentyl)hexyl)piperidine-3,4,5-triyl triacetate (78)

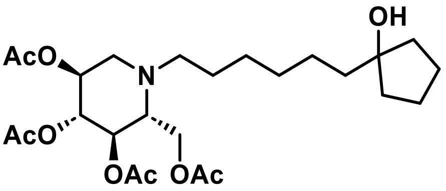

Prepared following general procedure **GP5** and starting from **72** (41 mg, 0.1 mmol, 1.0 equiv.) and cyclopentanone (62 μL, 0.7 mmol, 7.0 equiv.) affording **78** in 37% NMR yield. Purification by flash column chromatography on a silica gel column using a mixture of Cyclohexane/EtOAc 4/1 provided an analytically pure sample for characterization.

^{1}H NMR (400 MHz, CDCl$_3$) δ 5.11 – 5.00 (m, 2H), 4.95 (td, *J* = 9.6, 5.0 Hz, 1H), 4.20 – 4.09 (m, 2H), 3.19 (dd, *J* = 11.4, 5.0 Hz, 1H), 2.77 – 2.68 (m, 1H), 2.63 (d, *J* = 8.8 Hz, 1H), 2.55 (td, *J* = 9.2, 4.5 Hz, 1H), 2.35 – 2.28 (m, 1H), 2.07 (s, 3H), 2.01 (s, 6H), 2.00 (s, 3H), 1.82 – 1.75 (m, 2H), 1.65 – 1.51 (m, 8H), 1.44 – 1.35 (m, 4H), 1.33 – 1.22 (m, 4H).

^{13}C NMR (101 MHz, CDCl$_3$) δ 171.1, 170.5, 170.2, 169.9, 82.6, 74.8, 69.7, 69.6, 61.6, 59.6, 53.0, 51.9, 41.6, 39.8, 39.8, 30.1, 27.2, 24.7, 24.7, 23.9, 21.0, 21.0, 20.9, 20.8.

HRMS(ESI) m/z calcd for $C_{25}H_{42}NO_9$ [M+H]$^+$: 500.2854, found: 500.2862.

(2*R*,3*R*,4*R*,5*S*)-2-(acetoxymethyl)-1-(7-hydroxy-7-(tetrahydro-2H-pyran-4-yl)octyl)piperidine-3,4,5-triyl triacetate (79)

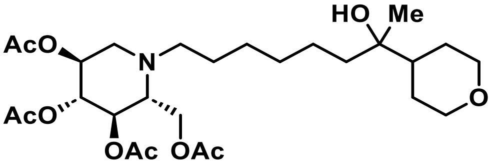

Prepared following general procedure **GP5** and starting from **72** (41.3 mg, 0.1 mmol, 1.0 equiv.) and 1-(tetrahydro-2*H*-pyran-4-yl)ethan-1-one (88 μL, 0.7 mmol, 7.0 equiv.) affording **79** in 39% NMR yield. Purification by flash column chromatography on a silica gel column using a mixture of Cyclohexane/EtOAc 4/1 provided an analytically pure sample for characterization.

^{1}H NMR (400 MHz, CDCl$_3$) δ 5.12 – 5.00 (m, 2H), 4.96 (dd, *J* = 9.8, 4.9 Hz, 1H), 4.20 – 4.09 (m, 2H), 4.06 – 3.98 (m, 2H), 3.35 (td, *J* = 11.3, 2.1 Hz, 2H), 3.19 (dd, *J* = 11.4, 5.0 Hz, 1H), 2.72 (ddd, *J* = 15.7, 9.5, 6.1 Hz, 1H), 2.63 (d, *J* = 8.8 Hz, 1H), 2.58 – 2.50 (m, 1H), 2.30 (t, *J* = 10.8 Hz, 1H), 2.07 (s, 3H), 2.01 (s, 6H), 2.00 (s, 3H), 1.66 – 1.44 (m, 6H), 1.41 – 1.24 (m, 9H), 1.10 (s, 3H).
^{13}C NMR (101 MHz, CDCl$_3$) δ 171.1, 170.5, 170.2, 169.9, 74.8, 73.8, 69.6, 69.6, 68.5, 68.4, 61.6, 61.6, 59.6, 53.0, 51.8, 44.8, 44.8, 39.6, 30.1, 27.7, 27.2, 27.0, 24.8, 24.8, 23.8, 23.3, 21.0, 21.0, 20.9, 20.8.
HRMS(ESI) m/z calcd for $C_{27}H_{46}NO_{10}$ $[M+H]^+$: 544.3116, found: 544.3131.

(2*R*,3*R*,4*R*,5*S*)-2-(acetoxymethyl)-1-(7-hydroxy-7-methyl-9-phenylnonyl)piperidine-3,4,5-triyl triacetate (80)

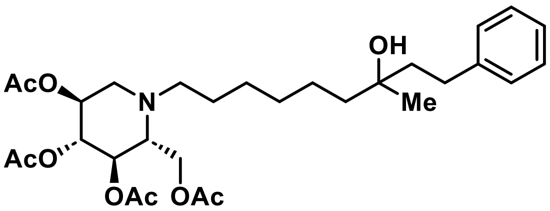

Prepared following general procedure **GP5** and starting from **72** (41 mg, 0.1 mmol, 1.0 equiv.), 4-phenylbutan-2-one (75 µL, 0.5 mmol, 5.0 equiv.) and TBAOx (287 mg, 0.5 mmol, 5.0 equiv.) affording **80** in 50% NMR yield. Purification by flash column chromatography on a silica gel column using a mixture of Cyclohexane/EtOAc 4/1 provided an analytically pure sample for characterization.
^{1}H NMR (400 MHz, CDCl$_3$) δ 7.30 – 7.24 (m, 2H), 7.21 – 7.14 (m, 3H), 5.11 – 5.00 (m, 2H), 4.99 – 4.91 (m, 1H), 4.15 (dd, *J* = 4.8, 2.8 Hz, 2H), 3.19 (dd, *J* = 11.4, 5.0 Hz, 1H), 2.77 – 2.70 (m, 1H), 2.69 – 2.60 (m, 1H), 2.55 (ddd, *J* = 13.9, 9.4, 4.6 Hz, 3H), 2.34 – 2.27 (m, 1H), 2.06 (s, 3H), 2.01 (s, 3H), 2.01 (s, 3H), 2.00 (s, 3H), 1.78 – 1.72 (m, 2H), 1.52 – 1.47 (m, 2H), 1.46 – 1.39 (m, 2H), 1.38 – 1.24 (m, 6H), 1.22 (s, 3H).
^{13}C NMR (101 MHz, CDCl$_3$) δ 171.1, 170.5, 170.2, 169.9, 142.7, 128.5, 128.4, 125.8, 74.8, 72.7, 69.6, 69.6, 61.6, 59.6, 53.0, 51.8, 43.9, 43.9, 42.1, 30.5, 30.1, 27.2, 27.0, 27.0, 24.7, 23.9, 21.0, 20.9, 20.9, 20.8.
HRMS(ESI) m/z calcd for $C_{30}H_{46}NO_9$ $[M+H]^+$: 564.3167, found: 564.3170.

(2*R*,3*R*,4*R*,5*S*)-2-(acetoxymethyl)-1-(6-(4,4-difluoro-1-hydroxycyclohexyl)hexyl)piperidine-3,4,5-triyl triacetate (81)

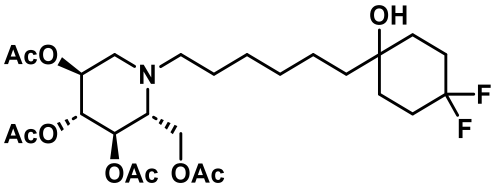

Prepared following general procedure **GP5** and starting from **72** (41 mg, 0.1 mmol, 1.0 equiv.), 4,4-difluorocyclohexan-1-one (67 mg, 0.5 mmol, 5.0 equiv.) and TBAOx (287 mg, 0.5 mmol, 5.0 equiv.) affording **81** in 36% NMR yield. Purification by flash column chromatography on a silica gel column using a mixture of Cyclohexane/EtOAc 4/1 provided an analytically pure sample for characterization.
^{1}H NMR (400 MHz, CDCl$_3$) δ 5.11 – 5.00 (m, 2H), 4.97 – 4.89 (m, 1H), 4.20 – 4.06 (m, 2H), 3.19 (dd, *J* = 11.4, 5.1 Hz, 1H), 2.76 – 2.67 (m, 1H), 2.64 – 2.59 (m, 1H), 2.51 (ddd, *J* = 13.7, 9.2, 4.4 Hz, 1H), 2.28

(dd, J = 11.4, 10.2 Hz, 1H), 2.06 (s, 3H), 2.01 (s, 6H), 2.00 (s, 3H), 1.93 – 1.83 (m, 3H), 1.70 – 1.59 (m, 4H), 1.52 – 1.38 (m, 4H), 1.37 – 1.25 (m, 7H).

^{13}C NMR (101 MHz, CDCl$_3$) δ 171.1, 170.5, 170.3, 169.9, 124.0 (t, J_{CF} = 242 Hz), 74.8, 70.0, 70.0, 69.7, 69.6, 61.7, 59.7, 52.9, 51.7, 42.8, 42.8, 33.60 (dd, J_{CF} = 9.1, 5.3 Hz), 30.0, 29.8, 29.7, 29.7, 29.5, 27.0, 24.7, 23.1, 21.0, 21.0, 20.9, 20.8.

^{19}F NMR (377 MHz, CDCl$_3$) δ -92.54 (d, J = 234 Hz), -104.02 (d, J = 234 Hz).

HRMS(ESI) m/z calcd for $C_{26}H_{42}F_2NO_9$ [M+H]$^+$: 550.2822, found: 550.2830.

(2*R*,3*R*,4*R*,5*S*)-2-(acetoxymethyl)-1-(6-(4-hydroxytetrahydro-2H-pyran-4-yl)hexyl)piperidine-3,4,5-triyl triacetate (82)

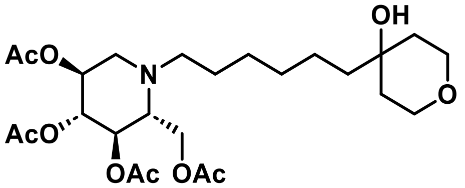

Prepared following general procedure **GP5** and starting from **72** (41 mg, 0.1 mmol, 1.0 equiv.), tetrahydro-4H-pyran-4-one (46 µL, 0.5 mmol, 5.0 equiv.) and TBAOx (287 mg, 0.5 mmol, 5.0 equiv.) affording **82** in 38% NMR yield. Purification by flash column chromatography on a silica gel column using a mixture of Cyclohexane/EtOAc 6/1 to Cyclohexane/EtOAc 3/2 provided an analytically pure sample for characterization.

^{1}H NMR (400 MHz, CDCl$_3$) δ 5.10 – 5.00 (m, 2H), 4.96 (dd, J = 10.0, 5.1 Hz, 1H), 4.22 – 4.07 (m, 2H), 3.80 – 3.70 (m, 4H), 3.19 (dd, J = 11.4, 5.1 Hz, 1H), 2.72 (ddd, J = 13.8, 9.6, 6.3 Hz, 1H), 2.62 (d, J = 8.7 Hz, 1H), 2.53 (ddd, J = 13.7, 9.2, 4.6 Hz, 1H), 2.29 (dd, J = 11.4, 10.2 Hz, 1H), 2.07 (s, 3H), 2.01 (s, 6H), 2.00 (s, 3H), 1.71 – 1.62 (m, 3H), 1.49 – 1.40 (m, 5H), 1.39 – 1.22 (m, 6H).

^{13}C NMR (101 MHz, CDCl$_3$) δ 171.1, 170.5, 170.3, 169.9, 74.8, 69.7, 69.6, 69.0, 64.0, 61.7, 59.6, 52.9, 51.7, 43.4, 37.8, 37.7, 29.9, 27.1, 24.7, 22.4, 21.0, 21.0, 20.9, 20.8.

HRMS(ESI) m/z calcd for $C_{25}H_{42}NO_{10}$ [M+H]$^+$: 516.2803, found: 516.2818.

(2*R*,3*R*,4*R*,5*S*)-2-(acetoxymethyl)-1-(6-(-8-(*tert*-butoxycarbonyl)-3-hydroxy-8-azabicyclo[3.2.1]octan-3-yl)hexyl)piperidine-3,4,5-triyl triacetate (83)

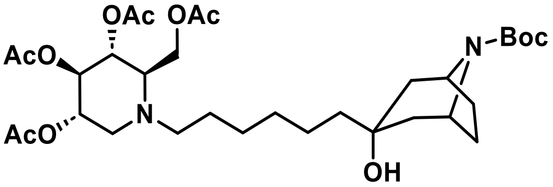

Prepared following general procedure **GP5** and starting from **72** (41 mg, 0.1 mmol, 1.0 equiv.) and *tert*-butyl (1*R*,5*S*)-3-oxo-8-azabicyclo[3.2.1]octane-8-carboxylate (113 mg, 0.5 mmol, 5.0 equiv.) affording **83** in 67% NMR yield. Purification by flash column chromatography on a silica gel column using a mixture of Cyclohexane/EtOAc 6/1 to Cyclohexane/EtOAc 3/2 provided an analytically pure sample for characterization.

^{1}H NMR (400 MHz, CDCl$_3$) δ 5.02 (q, J = 8.9 Hz, 2H), 4.93 (dq, J = 9.8, 5.7, 5.2 Hz, 1H), 4.22 – 4.06 (m, 4H), 3.22 – 3.12 (m, 1H), 2.75 – 2.64 (m, 1H), 2.64 – 2.57 (m, 1H), 2.51 (d, J = 11.3 Hz, 1H), 2.27

(t, J = 10.8 Hz, 1H), 2.13 – 2.05 (m, 2H), 2.04 (s, 3H), 1.99 (s, 6H), 1.98 (s, 3H), 1.95 – 1.73 (m, 5H), 1.64 – 1.53 (m, 2H), 1.43 (s, 9H), 1.38 – 1.18 (m, 9H).

^{13}C NMR (101 MHz, CDCl$_3$) δ 171.0, 170.4, 170.2, 169.8, 153.6, 79.7, 79.1, 74.7, 73.5, 71.3, 69.6, 61.6, 59.6, 53.4, 52.9, 52.6, 51.7, 46.9, 42.9, 42.1, 38.0, 31.7, 29.7, 28.6, 28.2, 27.6, 27.0, 24.6, 22.6, 21.0, 20.9, 20.8, 20.8.

HRMS(ESI) m/z calcd for $C_{32}H_{53}N_2O_{11}$ [M+H]$^+$: 641.3644, found: 641.3652.

9.3 Unsuccessful substrates

Highly hindered molecule shown lower reactivity and easier reduction to the corresponding secondary alcohol:

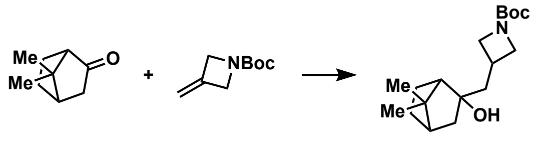

10% isolated yield

The presence of leaving group in α- led to an extremelly fast ring opening *via* Spin-Centered Shift (SCS) and no product formation:

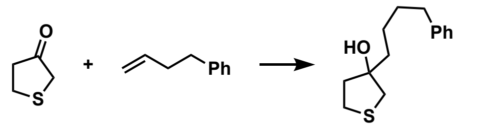

Not found

Competitive alcohol or pinacol formation on aromatic ketones:

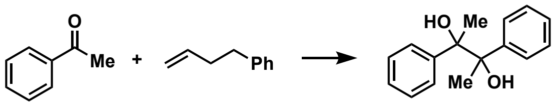

37%, major product

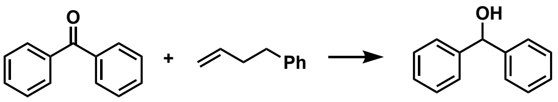

50%, major product

Electron deficient arenes can slow down the reduction of ketones to ketyl radicals in some cases:

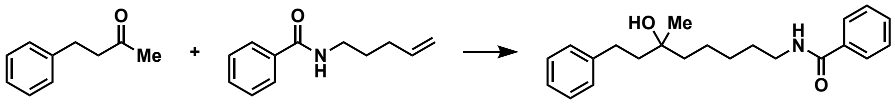

Electron deficient arene

Not found

Internal alkenes, styrenes and e-deficient alkenes are typically underperforming:

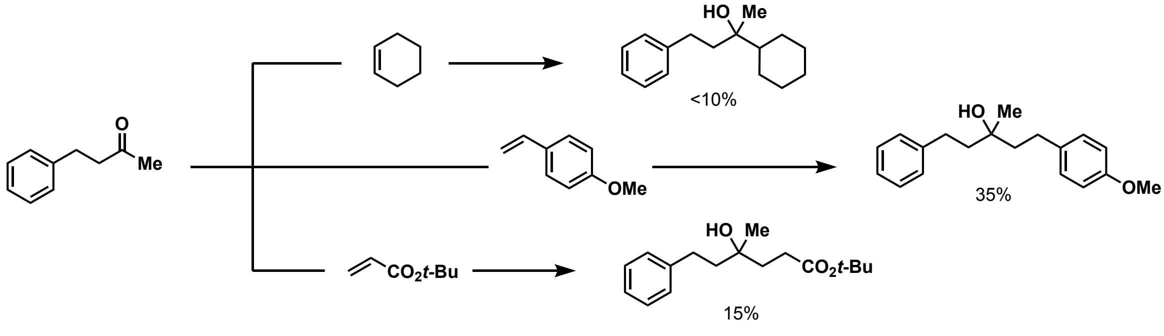

9.4 Application on intramolecular reactions

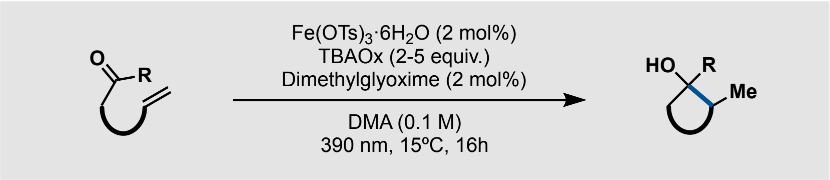

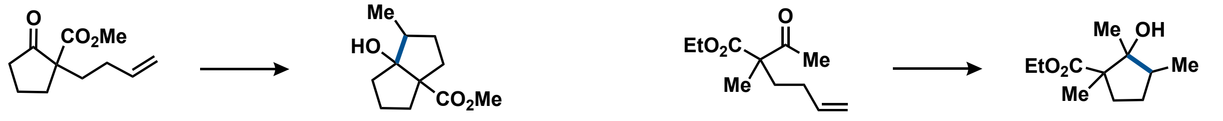

S11, 55%, *d.r* = 2.3:1 **S12**, 50%, *d.r* = 6:3:1

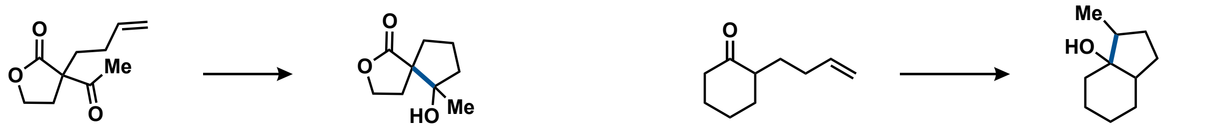

S13, 53%, *d.r* = 8:2:1 **S14**, 52%, *d.r* = 8:2:1

methyl 6a-hydroxy-1-methylhexahydropentalene-3a(1H)-carboxylate (S11)

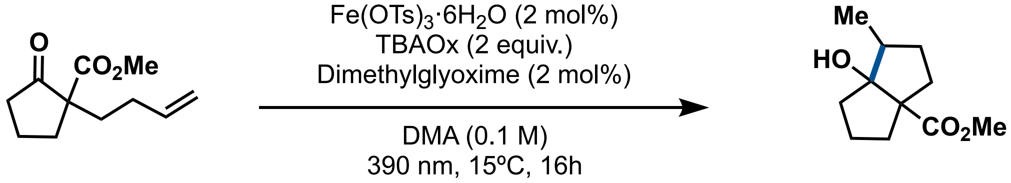

S11, 55%, *d.r* = 2.3:1

Methyl 1-(but-3-en-1-yl)-2-oxocyclopentane-1-carboxylate was prepared according to a previously reported procedure. [9] In a vial, methyl 1-(but-3-en-1-yl)-2-oxocyclopentane-1-carboxylate (39.6 mg, 0.2 mmol, 1.0 equiv.), $Fe(OTs)_3·6H_2O$ (2.4 mg, 0.002 mmol, 2 mol%), and dimethylglyoxime (0.8 mg, 0.002 mmol, 2 mol%) were added. The vial has been then loaded in the glovebox where tetrabutylammonium oxalate (342 mg, 0.4 mmol, 2 equiv.) was added. The vial has been then removed from the glovebox dry and degassed DMA (2 mL, 0.1 M) was added. The reaction mixture has been irradiated at 390 nm at 15ºC for 16h. After this time, 15 mL of saturated solution of $NaHCO_3$ were added and extracted with EtOAc (10 mL x 3). The combined organic phases have been dried over $MgSO_4$ and concentrated. The crude has been purified by column chromatography (2:8 Cyclohexane in EtOAc) to afford the product (**S11**) as yellow oil (55%, 22.2 mg, *d.r.* = 2.3:1).

^{1}H NMR (400 MHz, $CDCl_3$) δ 3.64 (s, 3H), 2.57 – 2.35 (m, 2H), 2.29 – 2.22 (m, 0.3H), 2.22 – 2.09 (m, 1H), 1.98 – 1.88 (m, 1H), 1.76 – 1.63 (m, 2H), 1.62 – 1.49 (m, 3H), 1.45 (ddd, J = 13.2, 7.5, 1.2 Hz, 1H), 1.40 – 1.29 (m, 1H), 1.20 – 1.06 (m, 1H), 0.94 (d, J = 6.8 Hz, 3H).

^{13}C NMR (101 MHz, $CDCl_3$) δ 177.3, 94.0, 62.0, 52.0, 45.2, 38.0, 35.5, 35.3, 30.6, 24.9, 13.2.

HRMS(ESI) m/z calcd for $C_{11}H_{18}O_3$ $[M]^+$ 198.1256, found 198.1247.

Ethyl 2-hydroxy-1,2,3-trimethylcyclopentanecarboxylate (S12)

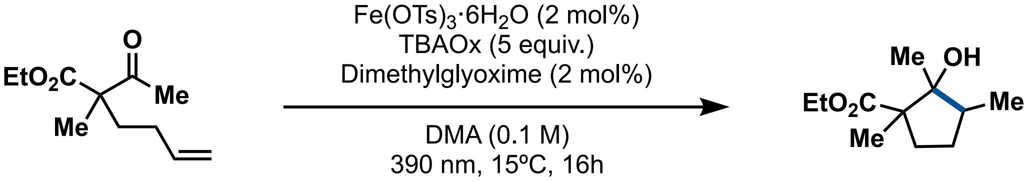

S12, 50%, *d.r* = 8:4:1

Ethyl 2-acetyl-2-methyl-5-hexenoate was prepared according to a previously reported procedure.[10] In a microwave vial equipped with a stirring bar were added ethyl 2-acetyl-2-methyl-5-hexenoate (39.7 mg, 0.2 mmol, 1.0 equiv.), dimethylglyoxime (0.2 mg, 0.002 mmol, 2 mol%) and $Fe(OTs)_3{\cdot}6H_2O$ (1.4 mg, 0.002 mmol, 2 mol%). The vial was transferred into the glovebox and TBAOx (573 mg, 1.0 mmol, 5.0 equiv.) was charged. The tube was then capped with an aluminium crimp cap with PTFE/butyl septum and taken out of the glovebox. 2 mL of dry and degassed DMA were added to the vial via a syringe under a positive N_2 flow. The mixture was stirred under 390 nm light irradiation at 15°C for 16 h. After the reaction is completed, the solution was poured into a saturated solution of $NaHCO_3$, which was then extracted three times with EtOAc. The combined organic phases were dried over $MgSO_4$, filtered and the solvent removed under rotatory evaporation. The crude was then purified by flash chromatography on silica using a mixture of Hexane to Hexane/EtOAc 8/2 to provide product **S12** as a colorless oil (20.4 mg, 50%, *d.r.* = 6:3:1).

^{1}H NMR (400 MHz, $CDCl_3$) δ 4.16 (q, *J* = 7.4 Hz, 1.4H), 4.09 (q, *J* = 7.1 Hz, 0.6H), 2.35 – 2.27 (m, 0.6H), 2.22 – 2.15 (m, 0.5H), 2.1 – 1.83 (m, 2H), 1.59 – 1.34 (m, 2H), 1.29 – 1.23 (m, 4.1H), 1.20 (s, 1.9H), 1.11 (s, 1.1H), 1.07 (s, 1.9H), 0.98 – 0.93 (m, 3H).

^{13}C NMR (101 MHz, $CDCl_3$) δ 178.5, 177.3, 177.0, 82.4, 82.2, 80.4, 60.9, 60.5, 57.7, 55.5, 55.2, 43.2, 42.3, 39.3, 34.0, 32.9, 30.3, 30.2, 28.5, 26.5, 22.0, 21.3, 20.0, 19.6, 18.6, 18.2, 15.7, 14.4, 14.3, 13.2.

HRMS(ESI): molecular weight peak not found despite extensive efforts.

6-Hydroxy-6,7-dimethyl-2-oxaspiro[4.4]nonan-1-one (S13)

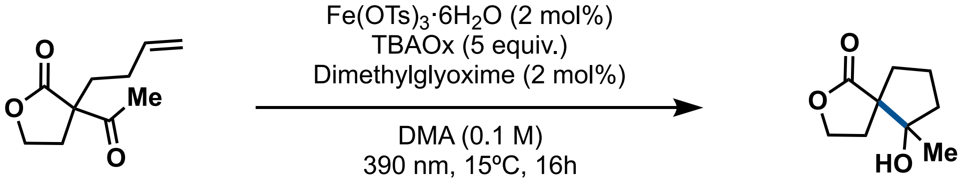

S13, 53%, *d.r* = 7:2:1

3-acetyl-3-(3-buten-1-yl)dihydro-2(3*H*)-furanone was prepared according to a previously reported procedure.[10] In a microwave vial equipped with a stirring bar were added 3-acetyl-3-(3-buten-1-yl)dihydro-2(3*H*)-furanone (36.4 mg, 0.2 mmol, 1.0 equiv.), dimethylglyoxime (0.2 mg, 0.002 mmol, 2 mol%) and $Fe(OTs)_3{\cdot}6H_2O$ (1.4 mg, 0.002 mmol, 2 mol%). The vial was transferred into the glovebox and TBAOx (573 mg, 1.0 mmol, 5.0 equiv.) was charged. The tube was then capped with an aluminium crimp cap with PTFE/butyl septum and taken out of the glovebox. 2 mL of dry and degassed DMA were added to the vial via a syringe under a positive N_2 flow. The mixture was stirred under 390 nm light irradiation at 15°C for 16 h. After the reaction is completed, the solution was poured into a saturated solution of $NaHCO_3$, which was then extracted three times with EtOAc. The combined organic phases were dried over $MgSO_4$, filtered and the solvent removed under rotatory evaporation. The crude was

then purified by flash chromatography on silica using a mixture of Hexane/EtOAc 8/2 to 6/4 to provide product **S13** as a colorless oil (19.5 mg, 53%, *d.r.* = 8:2:1).

^{1}H NMR (400 MHz, CDCl$_3$) δ 4.35 – 4.14 (m, 2H), 2.78 (ddd, *J* = 10.3, 7.2, 3.1 Hz, 0.1H), 2.68 (ddd, *J* = 13.4, 8.3, 7.2 Hz, 0.7H), 2.64 – 2.56 (m, 0.1H), 2.54 – 2.42 (m, 0.7H), 2.26 – 2.16 (m, 0.1H), 2.15 – 2.05 (m, 1.7H), 2.04 – 2.09 (m, 1H), 1.88 – 1.76 (m, 0.7H), 1.70 – 1.59 (m, 0.3H), 1.52 – 1.42 (m, 0.8H), 1.37 – 1.26 (m, 0.2H), 1.20 (s, 2.1H), 1.19 (s, 0.5H), 1.05 (s, 0.3H), 0.98 – 0.94 (m, 3H).

^{13}C NMR (101 MHz, CDCl$_3$) δ 181.4, 181.1, 180.4, 83.3, 82.0, 81.8, 66.4, 65.8, 65.8, 57.3, 55.7, 55.6, 43.4, 42.5, 41.9, 34.5, 33.9, 33.1, 32.4, 31.8, 31.2, 29.8, 28.8, 27.9, 20.8, 19.5, 17.7, 14.3, 13.8, 12.3.

HRMS(ESI): molecular weight peak not found despite extensive efforts.

3-methyloctahydro-3aH-inden-3a-ol (S14)

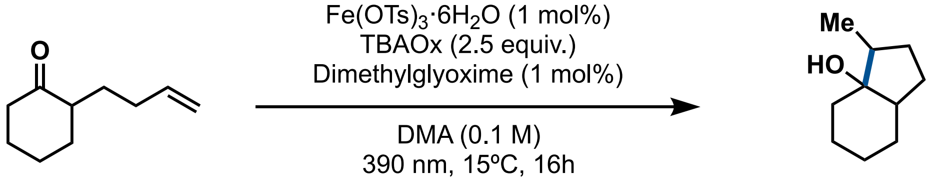

S14, 52%, *d.r* = xx : 1

2-(but-3-en-1-yl)cyclohexan-1-one was prepared according to a previously reported procedure.[11] In a vial, 2-(but-3-en-1-yl)cyclohexan-1-one (60.8 mg, 0.4 mmol, 1.0 equiv.), Fe(OTs)$_3$ · 6 H_2O (2.4 mg, 0.004 mmol, 1 mol%), and dimethylglyoxime (0.8 mg, 0.004 mmol, 1 mol%) were added. The vial has been then loaded in the glovebox where tetrabutylammonium oxalate (573 mg, 1 mmol, 2.5 equiv.) was added. The vial has been then removed from the glovebox dry and degassed DMA (2 mL, 0.2 M) was added. The reaction mixture has been irradiated at 390 nm at 15ºC for 16h. After this time, 15 mL of saturated solution of $NaHCO_3$ were added and extracted with EtOAc (10 mL x 3). The combined organic phases have been dried over $MgSO_4$ and concentrated. The crude has been purified by column chromatography (pure DCM to 9:1 DCM in Acetone) to afford the product (S14) as yellow oil (52%, 32.2mg, *d.r.* = 8:2:1).

^{1}H NMR (400 MHz, CDCl$_3$) δ 1.93 – 1.80 (m, 2H), 1.71 – 1.26 (m, 10H), 1.24 – 1.10 (m, 2H), 0.92 – 0.81 (m, 3H).

^{13}C NMR (101 MHz, CDCl$_3$) δ 78.4, 45.6, 44.9, 32.1, 29.9, 29.8, 28.4, 27.8, 24.3, 23.6, 22.8, 21.3, 20.5, 14.3, 12.8.

HRMS(ESI): molecular weight peak not found despite extensive efforts.

10 1.0 mmol scale experiment in batch

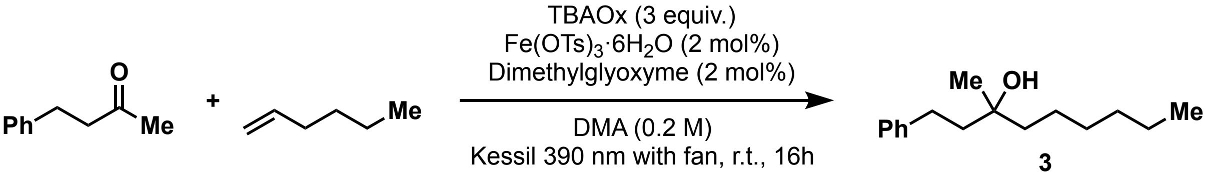

In a microwave vial equipped with a stirring bar were added dimethylglyoxime (2.3 mg, 0.02 mmol, 0.02 equiv.), Fe(OTs)$_3$·6H_2O (13.6 mg, 0.02 mmol, 0.02 equiv.). The vial has been charged in glovebox where we added TBAOx (1.72 g, 3.0 mmol, 3.0 equiv.). The tube was capped with an aluminium crimp cap

with PTFE/butyl septum and taken out of the glovebox. 5 mL of dry and degassed DMA has been added to the vial was then added via a syringe under a positive N_2 flow, followed by the 4-phenyl-2-butanone (150 μL, 1.0 mmol, 1.0 equiv.) and 1-hexene (125 μL, 4.0 mmol, 2.0 equiv.). The mixture was stirred under 390 nm light irradiation using two Kessil Lamps PR160L-390 nm placed at 6 cm of distance from the reaction vial. Two fans alimented with USB-adaptor were used to cool down the reaction mixture (Fig. S7). The reaction mixture was analyzed then by HPLC to check the conversion, which resulted full after 16 h with 62 % formation of the desired product. Then solution was treated with saturated $NaHCO_3$ (40 mL), and the aqueous phase was extracted three times with EtOAc (3 x 40 mL). The combined organic phases have been dried over $MgSO_4$, filtered and concentrated. The crude has been purified by flash chromatography starting from Cyclohexane to Cyclohexane/EtOAc 9/1, giving product **3** as colorless oil in 58 % (136.3 mg) isolated yield.

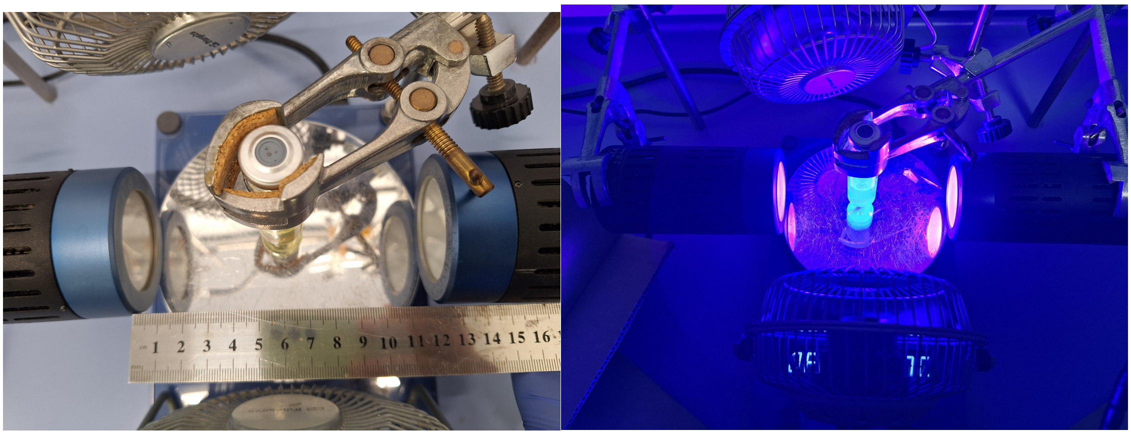

Figure S8. Setup used for the reactions on 1 mmol scale.

11 Experiments under flow conditions

Experimental details on the setup: The reaction in flow was carried out using a Vapourtec SF-10 laboratory pump fitted with a high power 385 nm led lamp on 8 mmol scale. IR measurements were carried out using an iC IR from Mettler-Toledo with a silicon-fitted probe. The overall aim was to evaluate whether the methodology presented herein would be amenable for scale up in flow. Therefore, the conditions employed were not optimised further from the standard reaction conditions and were just translated as is into the flow experiment.

Experimental protocol:

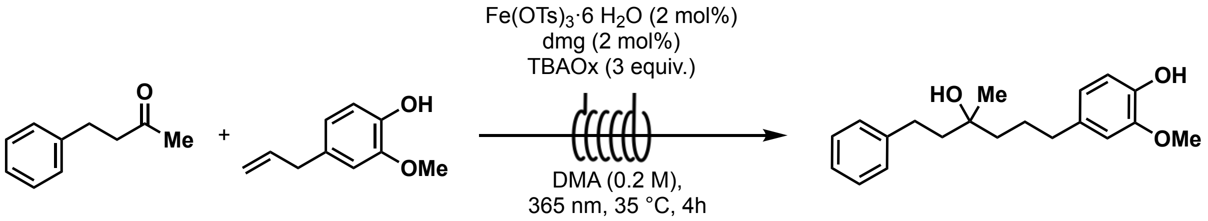

In the glovebox, tetrabutylammonium oxalate (13.75 g, 24.00 mmol, 3.0 equiv.) was weighted inside of a 3-necked 100 ml round-bottom flask containing a stirring bar, capped and taken out of the glovebox (Figure S9a). Outside the box, $Fe(OTs)_3·6H_2O$ (108.4 mg, 0.16 mmol, 0.02 equiv.) and dimethylglyoxime (18.58 mg, 0.16 mmol, 0.02 equiv.) were added in a sample vial (Figure S9b) and dissolved in 1 mL of dry and oxygen-free DMA (Figure S9c). Then, dry and oxygen-free DMA was added to the oxalate until

fully dissolved, followed by the addition of more solvent until a final volume of 40 mL was reached (Figure S9d). For the IR measurements, the IR probe was initially fitted in another flask (not showed) containing only DMA to measure the background IR spectra of the solvent. After the black acquisition, the probe was quickly put on the reaction flask so then another background spectra consisting in DMA+TBAOx could be measured (Figure S9e). Thereafter, IR spectra were recorded continuously while the remaining reaction components were added sequentially. The solution containing the iron catalyst and ligand was next added to the reaction flask, followed by 4-phenylbutan-2-one (1.186 g, 1.20 mL, 8.00 mmol, 1.0 equiv.) and eugenol (2.627 g, 2.48 mL, 16.00 mmol, 2.0 equiv.), which were both added neat (Figure S9f). Once all the reagents were added, the flask was equipped with two needles: one which would draw the reaction mixture from the flask and pump it into the reactor coil and another which would recirculate the reaction mixture back into the flask (Figure S9g). The flow was set at 1mL/min through a 10 mL reactor coil (Figure S8h) under 385 nm irradiation at 35 ºC. Cooling was achieved by using compressed air blowing onto its surface.

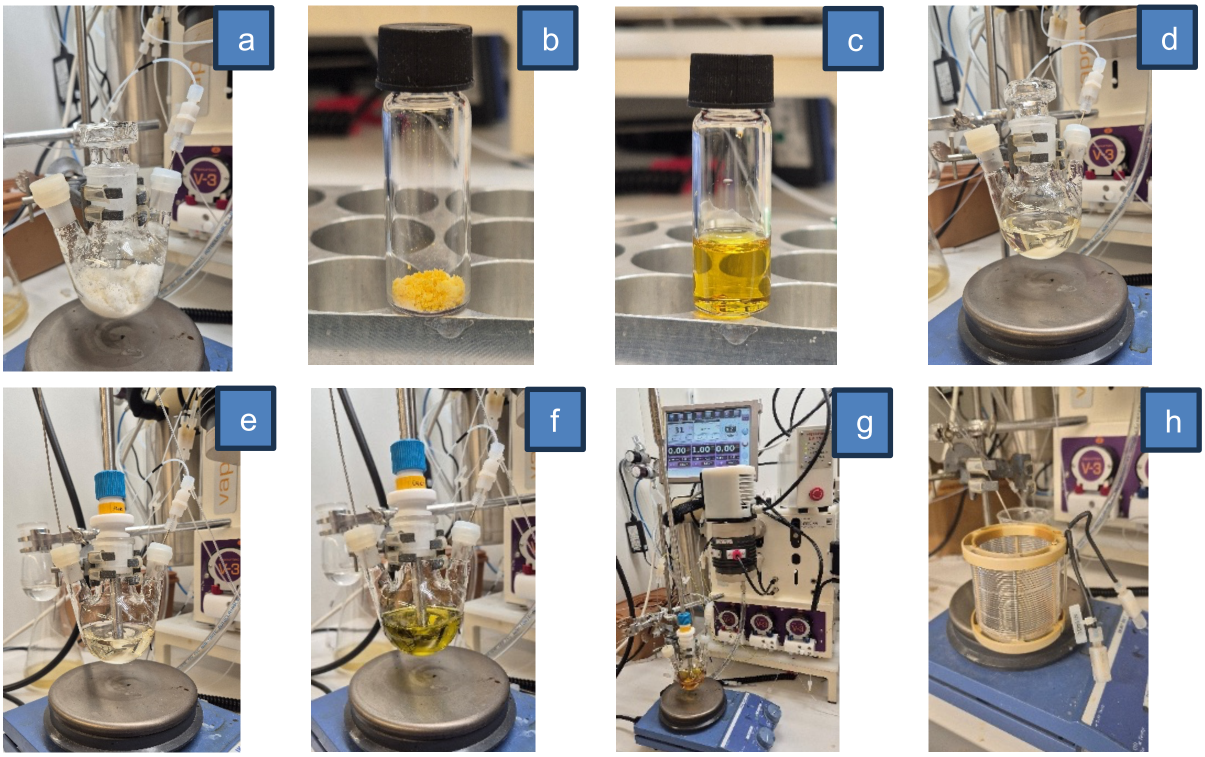

Figure S9. Graphical description of the experiment in flow.

After 4 hours of reaction, an aliquot of 1 mL was collected, transferred to a separatory funnel and diluted with EtOAc. Subsequently, 1 mL of a 0.2 M stock solution of 1,3-dinitrobenzene (DNB) in EtOAc was also added. The organics were treated three times with HCl 0.1M, brine, and then dried over $MgSO_4$. The solvent was removed using rotary evaporation to afford a yellow oil which was dissolved in $CDCl_3$ and subjected to NMR yield assay (Figure S10), compared with pure samples of the product and starting materials. Considering the dilution effect due to the addition of eugenol (2.5 mL), ketone (1.2 mL) and the stock solution containing $Fe(OTs)_3$ and DMG (~1 mL) to the reaction mixture, the concentration of the 1 mL drawn from the reaction mixture was assumed to be 0.18 M. Hence, by integrating for 1 the two protons of 1,3-DNB at ~8.5 ppm the integration for the CH_2 peaks belonging to the desired product at approx. 1.75 ppm gives the NMR yield, which is 55.6% after 4h. Additionally, 16.6% of ketone RSM

was also detected. This value would correspond to a theoretical yield of 1.4g for 8mmol reaction over 4h, or 8.4 g per day. Considering a reactor volume of 10 ml, the space time yield (STY) would be of 35 $g \cdot L^{-1} \cdot h^{-1}$.

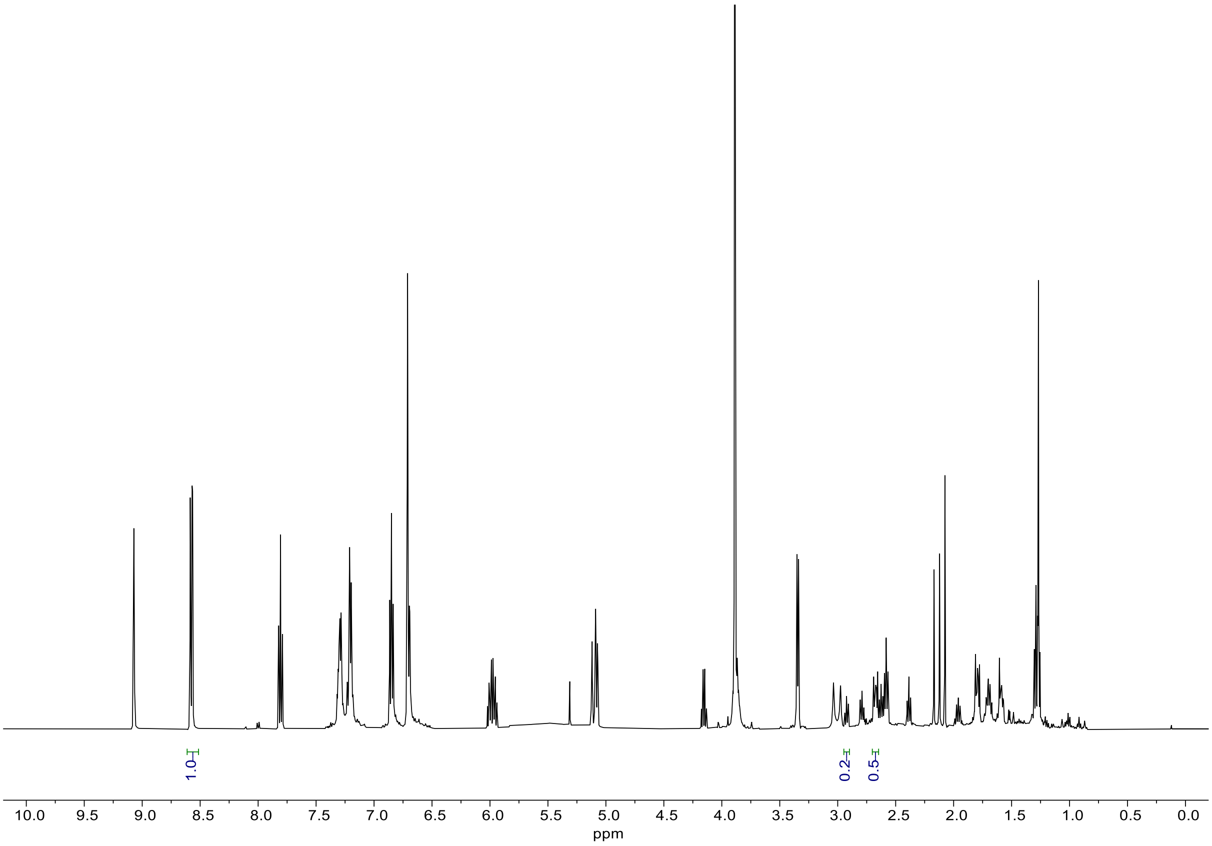

Figure S10. ^{1}H NMR analysis of the reaction mixture after 4h.

IR analysis:

The initial plan was to measure the progress of the reaction based on the carbonyl stretching. However, DMA hid all the possible peaks of interests, except for one peak at ca. 1540 cm-1 that we attribute deriving from TBAOx.

IR measurement of DMA (background):

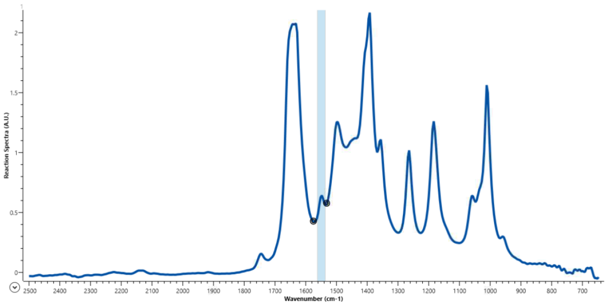

IR measurement with DMA+TBAOx. A clear increase in intensity of peak at ca. 1540 cm^{-1} was detected upon measuring the IR spectrum of the solution containing DMA and TBAOx compared to the blank solvent (representes as dotted green profile). For this reason, it was decided to track its variation over time:

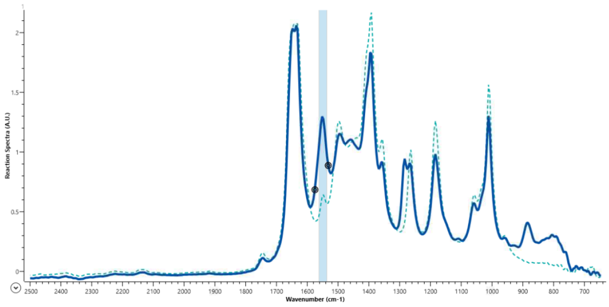

IR measurement with $Fe(OTs)_3{\cdot}6H_2O$+dmg. Upon addition of the solution containing $Fe(OTs)_3$ and DMG, no clear variations in the spectra were detected:

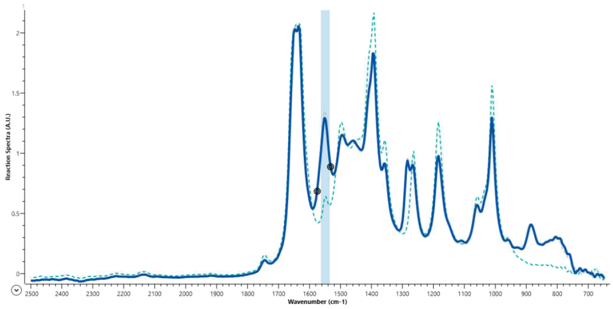

IR measurement after adding the ketone. Upon addition of the neat ketone, no clear variations in the spectra were detected:

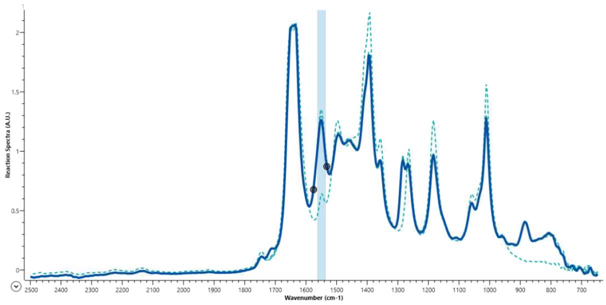

IR measurement after adding the eugenol. Upon addition of neat eugenol, no clear variations in the spectra were detected, except for a decrease in intensity of the peak at 1540 cm^{-1}. We attribute this phenomenon to an interaction between the acidic proton of eugenol with TBAOx:

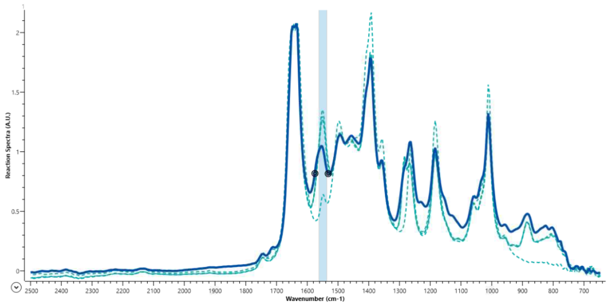

IR measurement after t=3h: At this time point we could detect a marked decrease of the peak 1540 cm^{-1}, which we attributed to the consumption of TBAOx:

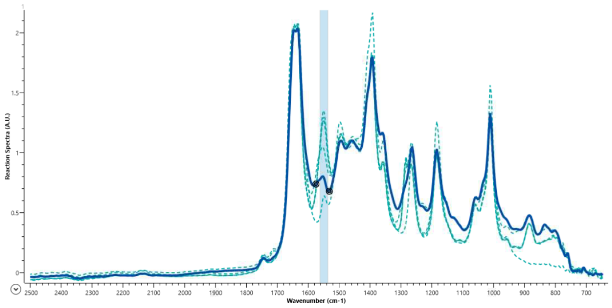

We therefore decided to plot the intensity of the peak at 1540 cm^{-1} over time, to get a rough estimation of the kinetic of the reaction:

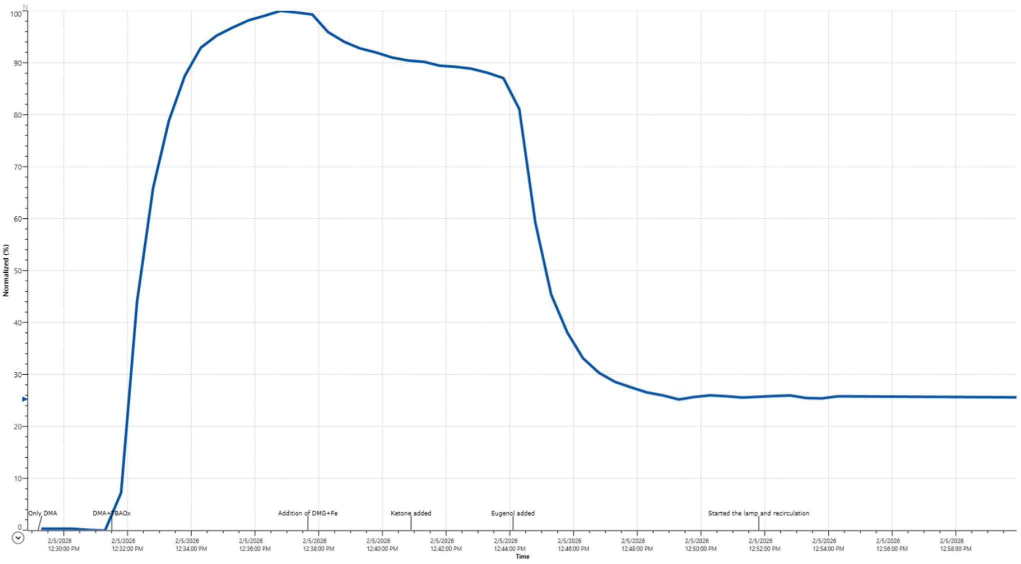

As noted earlier, during the first 30 minutes –when all reaction components were added sequentially– the peak attributed to TBAOx rose to a relative maximum and then dropped upon addition of eugenol. During the first few hours of recirculation, a steady consumption of the oxalate was observed with its corresponding peak reaching a relative minimum at 4 hours:

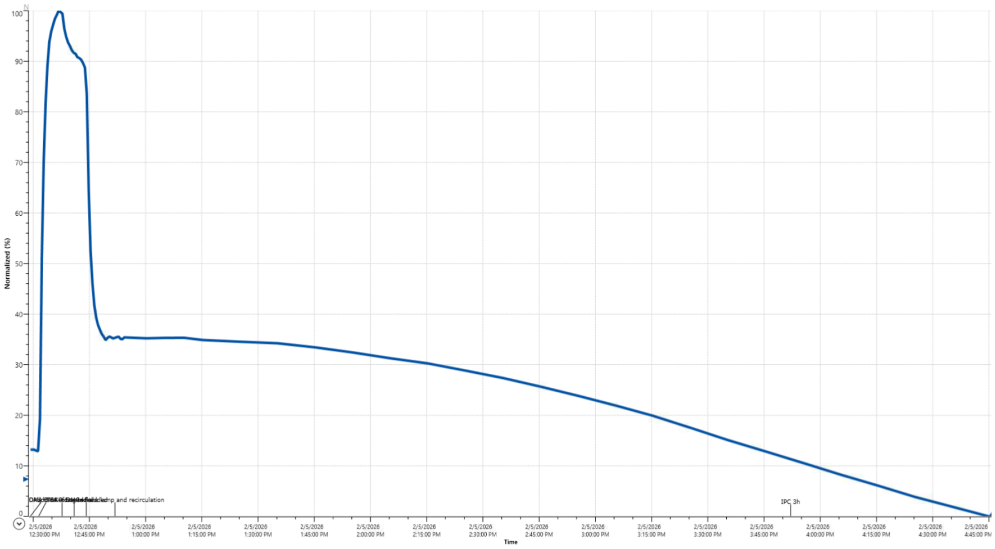

12 High-throughput experimentation (HTE)

General experimental details: All commercially available reagents and solvents were purchased from standard vendors and used without further purification unless otherwise stated. The equipment used for photochemistry was a Lumidox®II 48-position LED Array for TCR with 9mm Spaced 385nm LEDs and a Standard 48-Position Photoredox, TCR 6 Rows of 8 with 9mm Spacing. LC-MS analyses were performed on a Waters Acquity Classic with QDa masspec and PDA detector. For analyses at pH10 a BEH C18 column (1.7 µm, 50 x 2.1 mm) was used. Basic mobile phase A (pH10): 100% water + 4 mL/L basic stock (110 g H_2O, 128.5 g NH_4CO_3, 761.4 g ammonium hydroxide solution (25-30% NH_3). Basic mobile phase B (pH10): 95% MeCN + 5% water + 4 mL/L basic stock. Data was analysed using the software Masslynx.

Alkene substrate synthesis:

2-(2-methyl-1*H*-indol-3-yl)-1-(4-methylenepiperidin-1-yl)ethan-1-one (57)

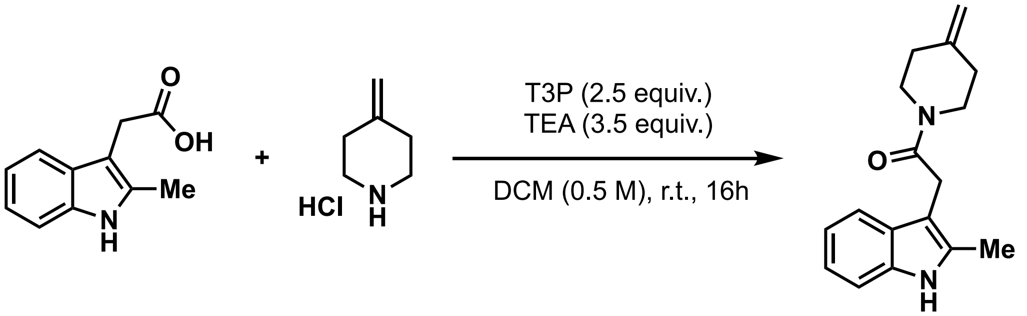

Under nitrogen atmosphere, 2-(2-methyl-1H-indol-3-yl)acetic acid (3.520 g, 18.60 mmol, 1.0 equiv.) 4-methylenepiperidine hydrochloride (2.983 g, 22.32 mmol, 1.20 equiv.) were added to a flask and suspended in DCM (37 mL). Then, triethylamine (6.589 g, 9.08 mL, 65.11 mmol, 3.5 Eq) was added in one portion, followed by 2,4,6-tripropyl-1,3,5,2,4,6-trioxatriphosphinane 2,4,6-trioxide (29.60 g, 27.69 mL, 46.51 mmol, 2.5 equiv.). The reaction was left to stir at room temperature overnight, after which it was diluted with DCM and washed 3 times with NaOH 0.1 M. The organics were then dried over $MgSO_4$, evaporated using rotary evaporation to afford **57** as an off-white powder which was used without further purification (4.67 g, 17.4 mmol, 93.5%).

^{1}H NMR ($CDCl_3$, 500 MHz): δ 7.85 (1H, br s), 7.54 (1H, d, *J* = 7.6 Hz), 7.26 (1H, m), 7.15 – 7.04 (2H, m), 4.77–4.58 (2H, m), 3.80 (2H, s), 3.62 (2H, s), 3.42 (2H, s), 2.42 (3H, s), 2.14 (2H, s), 1.85 (2H, s).

^{13}C NMR ($CDCl_3$, 126 MHz): δ 170.1, 144.9, 135.3, 132.0, 128.4, 121.4, 119.8, 118.1, 110.4, 109.6, 105.4, 47.6, 43.8, 34.8, 34.5, 31.2, 12.1.

HRMS(ESI+) m/z calcd for $C_{17}H_{21}N_2O$ $[M+H]^+$: 269.1654, found: 269.1741.

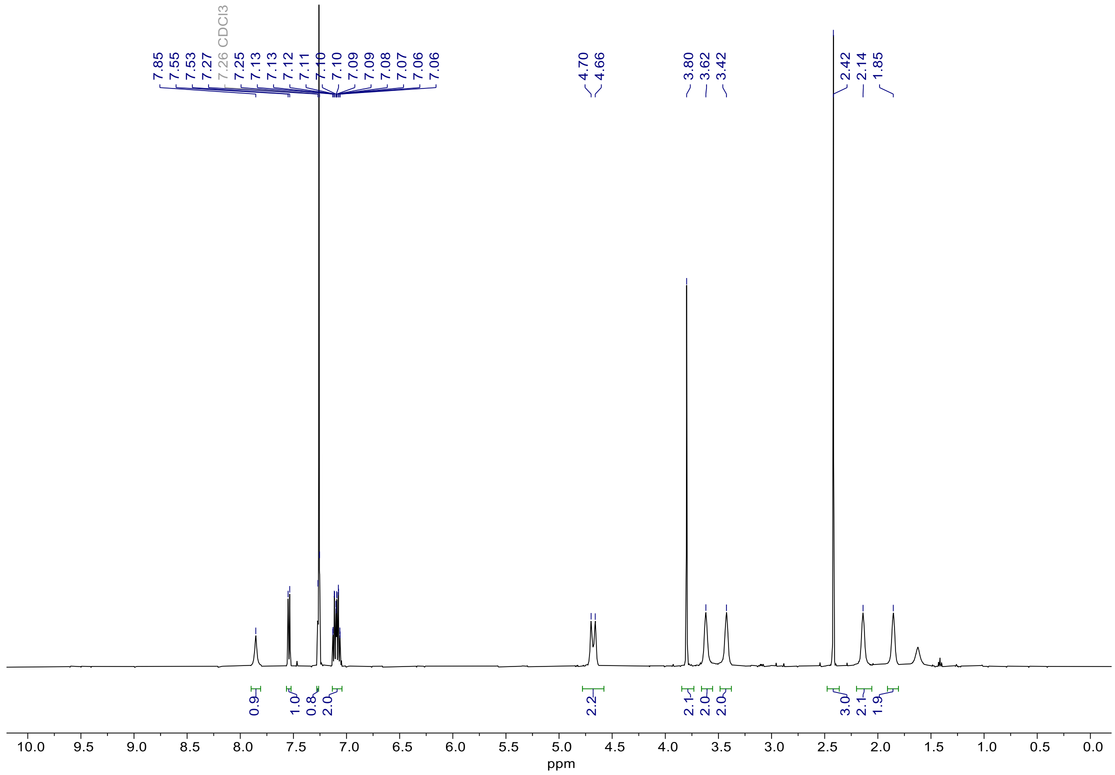

Figure S11. ^{1}H NMR spectrum of **57**.

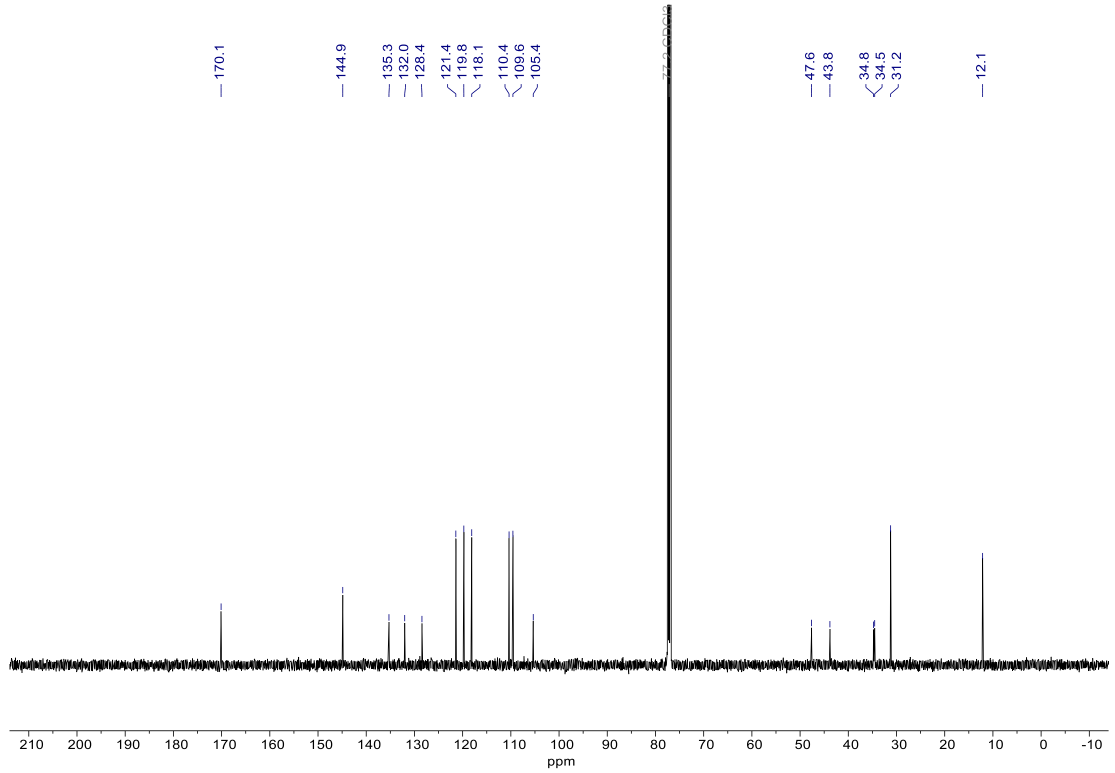

Figure S12. ^{13}C NMR spectrum of **57**.

Test of the alkene substrate in a model reaction:

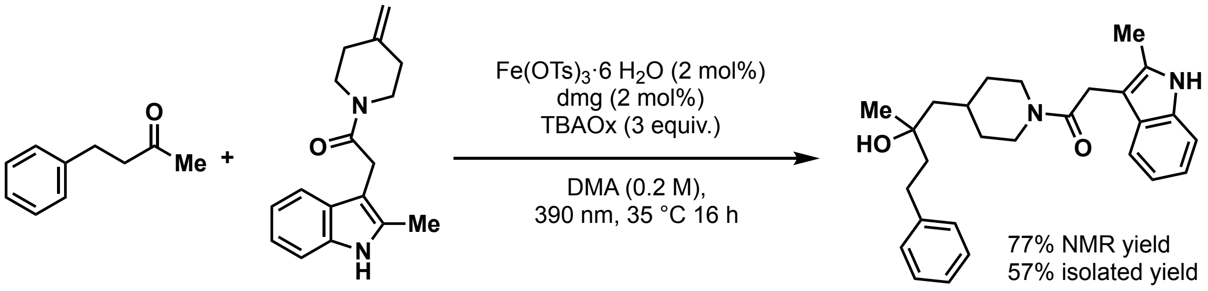

57 **S15**

In the glovebox, tetrabutylammonium oxalate (344 mg, 0.600 mmol, 3.0 equiv.) was added in a vial. Outside the box, $Fe(OTs)_3·6H_2O$ (2.71 mg, 4 µmol, 0.02 equiv.), dimethylglyoxime (464 µg, 4.00 µmol, 0.02 equiv.) and **57** (107 mg, 0.4 mmol, 2 equiv.) were added in another vial and subjected to three cycles of vacuum/N_2. The oxalate was then dissolved in dry and degassed DMA (1.00 mL) and transferred to the vial containing the other solids. Finally, 4-phenylbutan-2-one (29.6 mg, 30.0 µL, 0.200 mmol, 1.0 equiv.) was added neat to the mixture. The flask was then irradiated for 16 h with Kessil lamps (390 nm), using a fan for cooling (approx. temperature 35 °C). After the set amount of time, 1 mL of a 0.2 M stock solution of 1,3-dinitrobenzene in EtOAc was added to the crude reaction mixture. The crude reaction mixture was further diluted with EtOAc, and washed with NaOH 0.1M. The organics were then dried over $MgSO_4$, evaporated using rotary evaporation to afford an oil that was dissolved in MeOD to measure the ^{1}H-NMR yield of **S15** (77%):

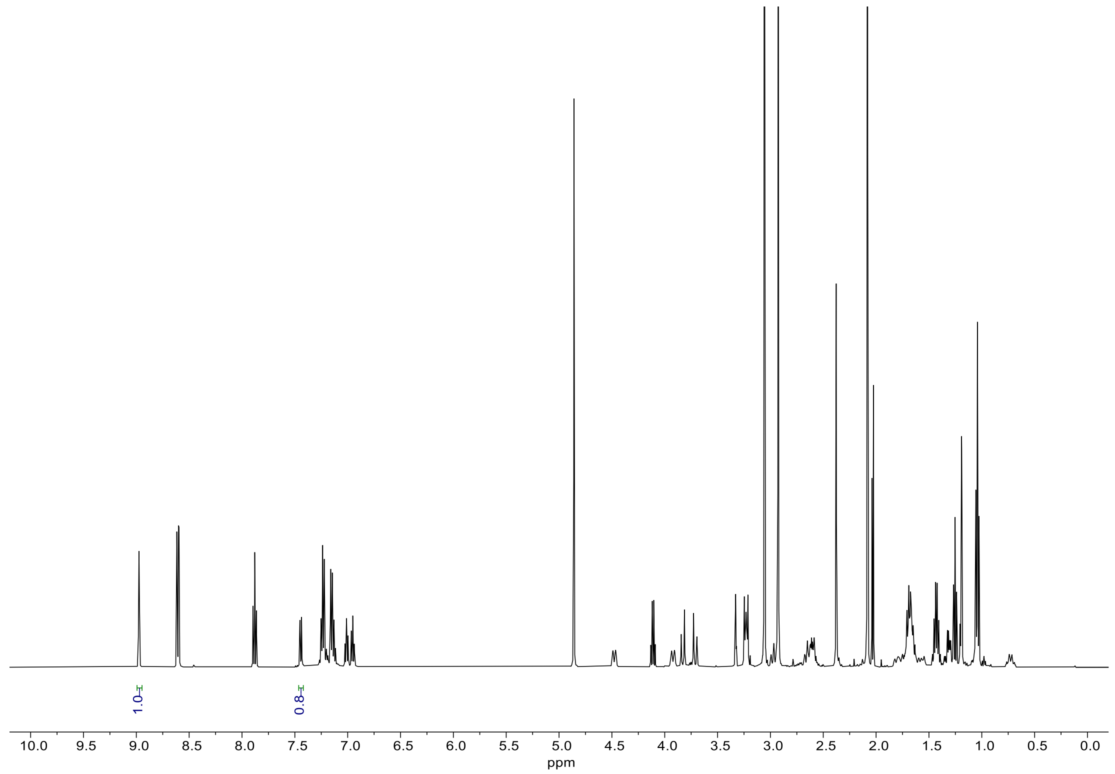

Figure S13. ^{1}H NMR spectrum of the crude reaction.

The crude mixture was then purified via basic preparative HPLC to afford the pure product **S15** as an oil (47.8 mg, 0.114 mmol, 57%).

^{1}H NMR (MeOD, 500 MHz): δ 7.44 (1H, dd, J = 7.9, 1.0 Hz), 7.26 – 7.19 (3H, m), 7.17 – 7.08 (3H, m), 7.01 (1H, ddd, J = 8.2, 7.0, 1.2 Hz), 6.95 (1H, ddd, J = 8.1, 7.0, 1.1 Hz), 4.50 – 4.42 (1H, m), 3.89 (1H,

d, J = 12.9 Hz), 3.81 (1H, d, J = 15.5 Hz), 3.69 (1H, d, J = 15.5 Hz), 2.98 – 2.88 (1H, m), 2.60 (3H, ttd, J = 15.1, 7.3, 4.4 Hz), 2.36 (3H, s), 1.81 – 1.71 (1H, m), 1.67 (3H, t, J = 8.6 Hz), 1.61 – 1.48 (1H, m), 1.35 – 1.20 (2H, m), 1.17 (3H, d, J = 1.3 Hz), 1.09 – 0.97 (1H, m), 0.75 – 0.63 (1H, m).

^{13}C NMR (MeOD, 126 MHz): δ 172.6, 144.1, 137.0, 133.4, 129.5, 129.4, 129.3, 126.6, 121.6, 119.7, 118.6, 111.4, 104.9, 73.5, 47.6, 46.0, 43.7, 35.1, 35.0, 33.0, 31.9, 31.4, 27.0, 11.6.

HRMS(ESI+) m/z calcd for $C_{27}H_{35}N_2O_2$ [M+H]$^+$: 419.2699, found: 419.2733.

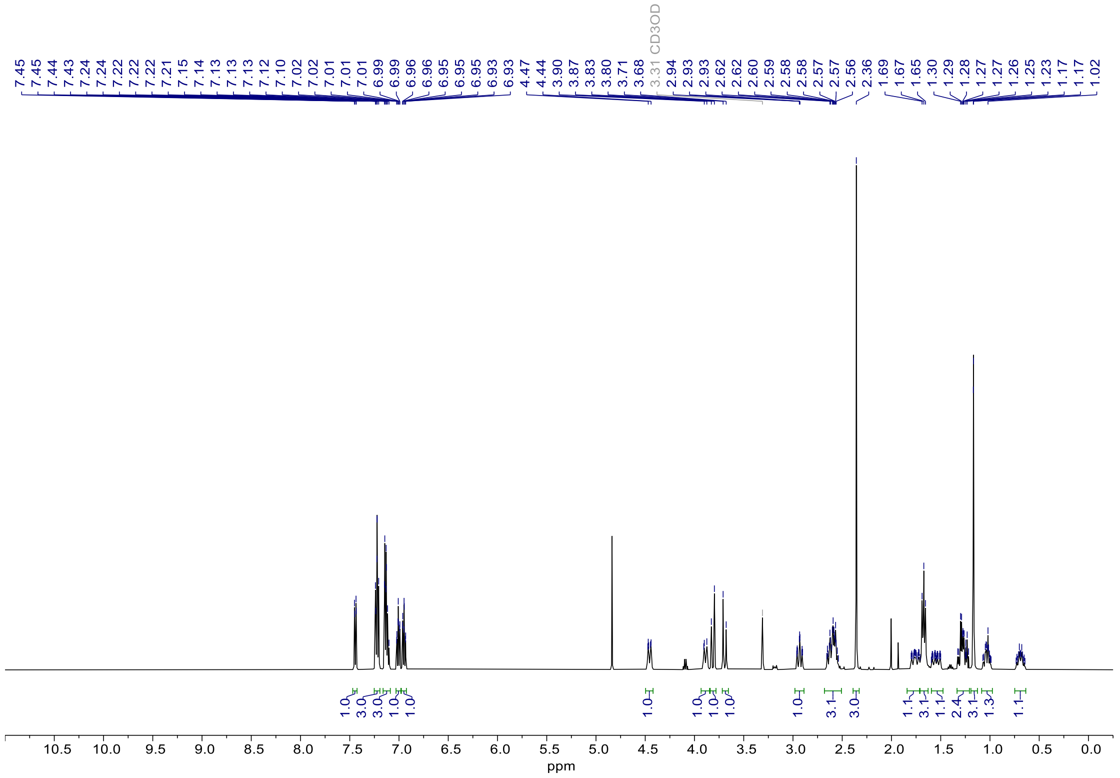

Figure S14. ^{1}H NMR spectrum of **S15**.

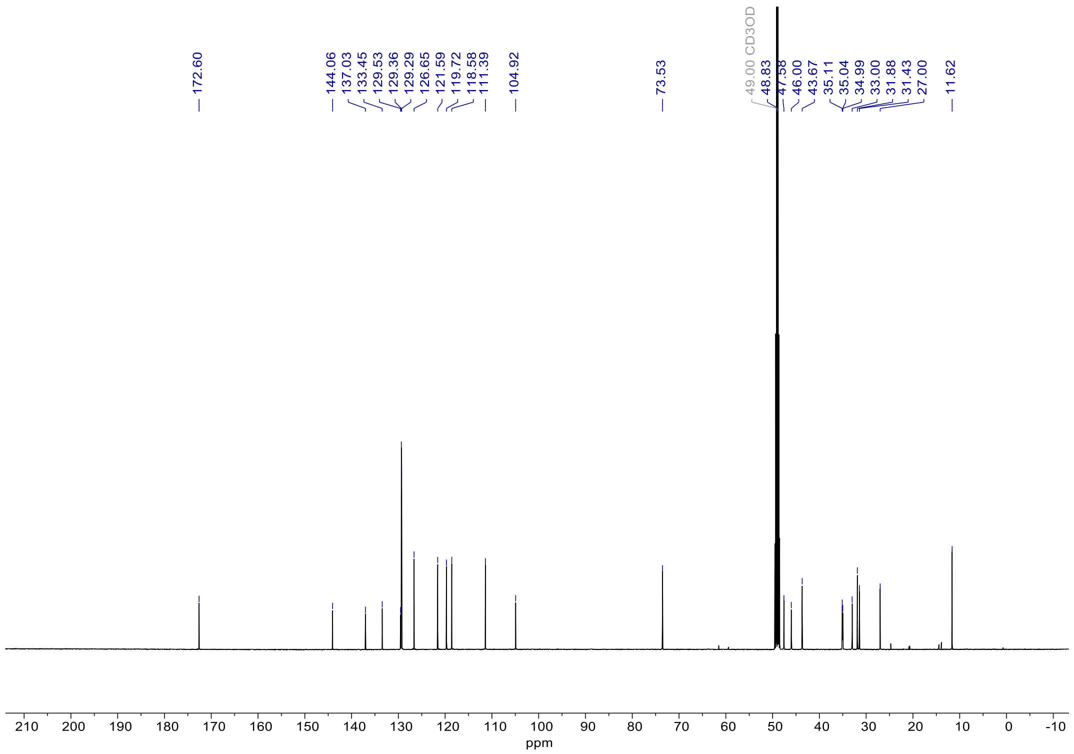

Figure S15. ^{13}C NMR spectrum of **S15**.

HTE library screening:

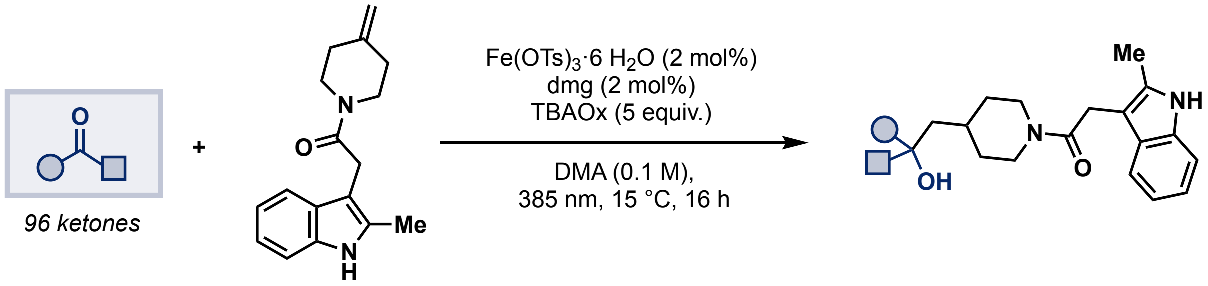

In the glovebox, tetrabutylammonium oxalate (6.876 g, 0.25x48 mmol, 5x48 equiv.), $Fe(OTs)_3·6H_2O$ (330 mg, 1.00x48 μmol, 0.02x48 equiv.), dimethylglyoxime (5.57 mg, 1.00x48 μmol, 0.02x48 equiv.) and 2-(2-methyl-1H-indol-3-yl)-1-(4-methylenepiperidin-1-yl)ethan-1-one (1.286 g, 0.1x48 mmol, 2x48 equiv.) were weighed out in a vial. To the vial, was added DMA until the total volume would be exactly 24 ml. Then, 500 μL were dispensed in each well containing the ketone building blocks (BBs) (*vide infra* for further details). The plate was sealed, transferred outside the glovebox and put on an array of 48 purple leds. The reaction was then irradiated for 16 h at 15 °C. After the set amount of time, the lid was removed and into each well were dispensed 100 μL of a 0.5 M stock solution of m-terphenyl in DMSO (=0.05 mmol). 5 μL of crude reaction mixture were aliquoted, transferred to an analytical plate and diluted with 190 μL of DMSO. The mixture was then filtered and analysed directly via LCMS. For the library screening, the data was obtained in two runs accounting for a total of 96 building blocks (BBs) derived from AZ's internal compound collection, generating the following list (Figure S16):

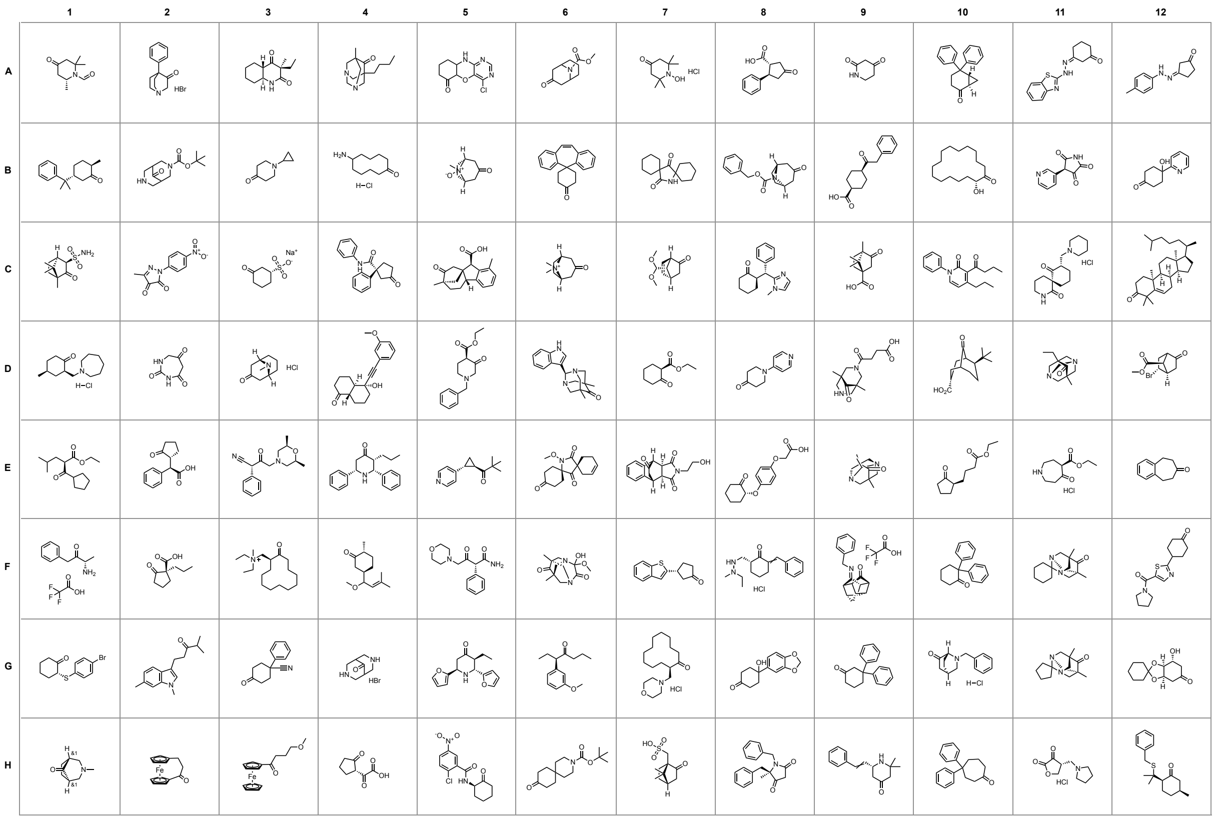

Figure S16. Ketone building blocks used in this study

Protocol description: The overall aim of the screening was to identify potential hits and determine whether the methodology presented herein would be amenable for miniaturisation and plate screening.

Therefore, the conditions employed were not optimised further from the standard reaction conditions and were just translated as is into a plate format. For us, it was important to determine whether it would be feasible to dispense all the reactants as a single stock solution, if the usage of a different LED setup would still form the desired product, and the high-throughput analysis via LCMS would be possible. The BBs were delivered pre-weighted in 0.1 mmol amounts in high-recovery vials (Figure S17, left). Subsequently, 0.5 mL of DMF or DMA were added to each vial, and the solid was left to fully dissolve either by shaking or sonicating the vial (*note: for compounds that were not fully soluble, additional 0.3 ml of DMSO were added*). Then, 0.25 mL (or 0.4 if DMSO was added to the vial) was transferred into a 1 mL flat bottom vial contained in a 96 well plate para-dox golden plate so the amount transferred for each reaction would be of 50 µmol (Figure S17, right).

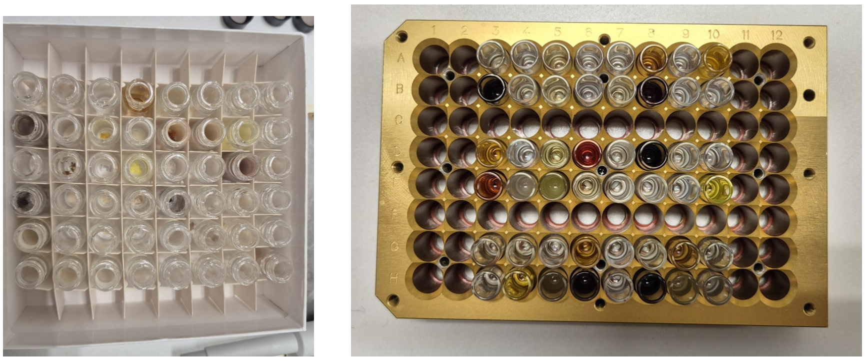

Figure S17. BBs delivered as pre-weighted in 0.1 mmol amounts (left). 50 µmol of material were dispensed into each vial (right).

Subsequently, the solvent was removed overnight using a Genevac evaporator. To the solvent-free vials containing the ketone BBs, were then added PTFE coated magnetic stirring bars and the plate was brought inside the glovebox (Figure S18, left). Inside the glovebox, to a vial containing all the remaining solids (5x48 equiv of TBAOx, 2x48 mol% of $Fe(OTs)_3{\cdot}6H_2O$, 2x48 mol% of dmg and 2x48 equiv. of the alkene partner) (Figure S18, right) was added dry and degassed DMA until the overall volume would be 24 mL for a 48 well reaction.

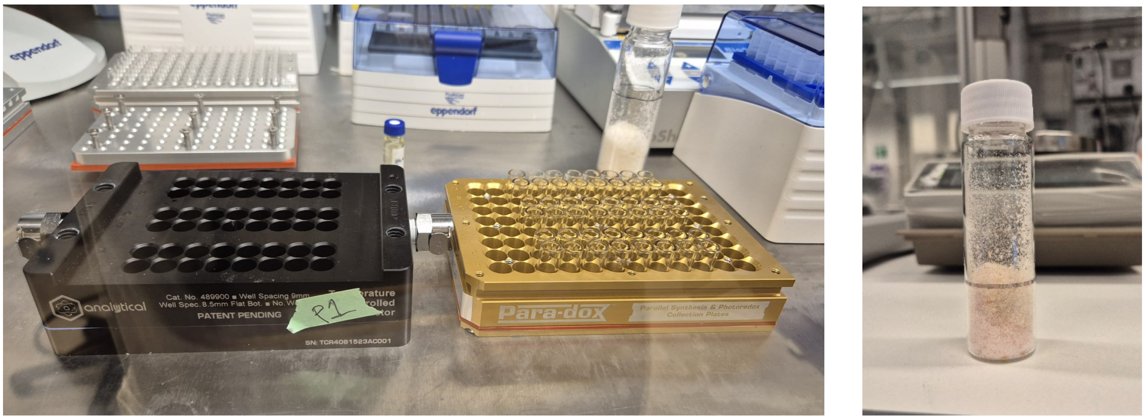

Figure S18. 48-well Para-dox TCR and and Para-dox I Gen plates inside a N_2-filled glovebox (left). Vial containing TBAOx, $Fe(OTs)_3{\cdot}6H_2O$ and dmg (right)

At this point, the vials containing the BBs were transferred over the temperature controlled reactor (TCR) plate, and 0.5 mL of the freshly made stock solution was added into each vial so the overall concentration would be 0.1 M with respect of the ketone (Figure S19).

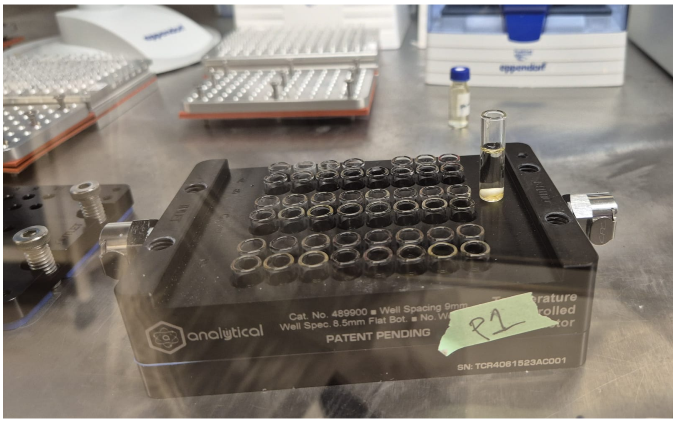

Figure S19. Para-dox TCR plate with all the vials filled with the desired reaction mixture.

The plate was then sealed, taken outside the box and placed over a 385 nm Lumidox®II 48-Position LED Arrays, which was itself placed over a Thermal Transfer Deck (Figure S20).

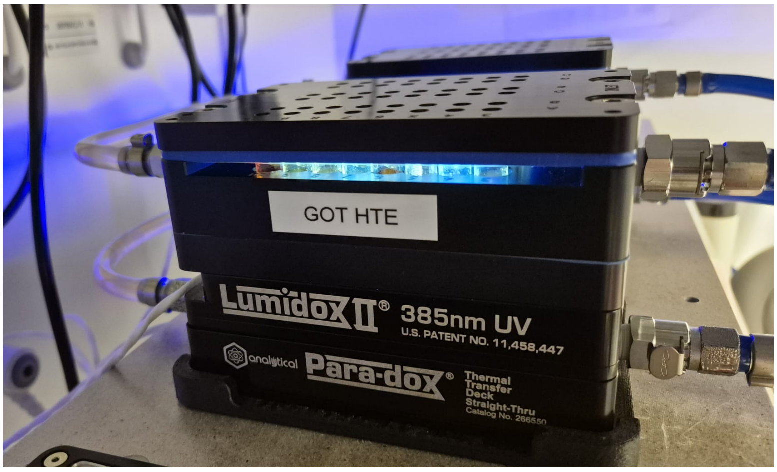

Figure S20. Stacked set-up for the photochemical library screening. From top to bottom: TCR, LED array, Thermal Transfer Deck. Two tubes connect the TCR to a chiller which recirculates a coolant at 15 °C. The bottom LED array is instead connected to a second chiller set at 0 °C.

After completion of the reaction, the TCR was detached from the chiller, opened and the vials were visually inspected to assess if precipitate had occurred, which became fully soluble upon addition of a 100 µL 0.5 M stock solution of m-terphenyl internal standard (IS) in DMSO (Figure S21).

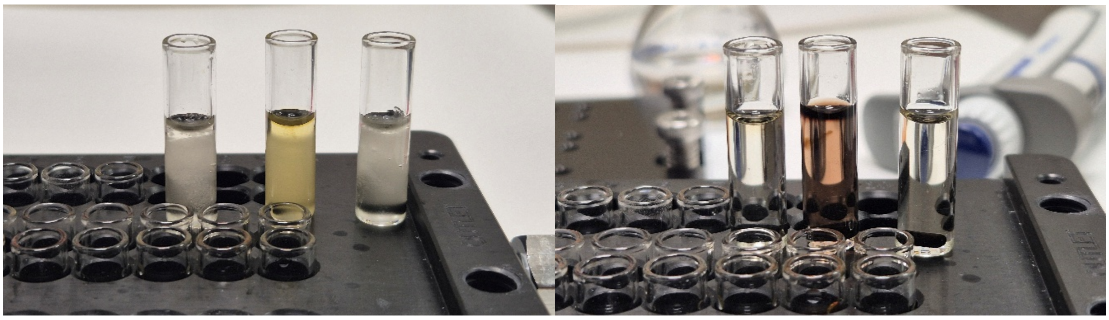

Figure S21. a) crude reaction mixtures (a) after reaction; (b) after addition of IS in DMSO.

From these solutions, 5 µL were taken out and transferred over a 96-well V-shaped microplate, diluted with 190 µL of DMSO, filtered and directly analysed via LCMS (Figure S22).

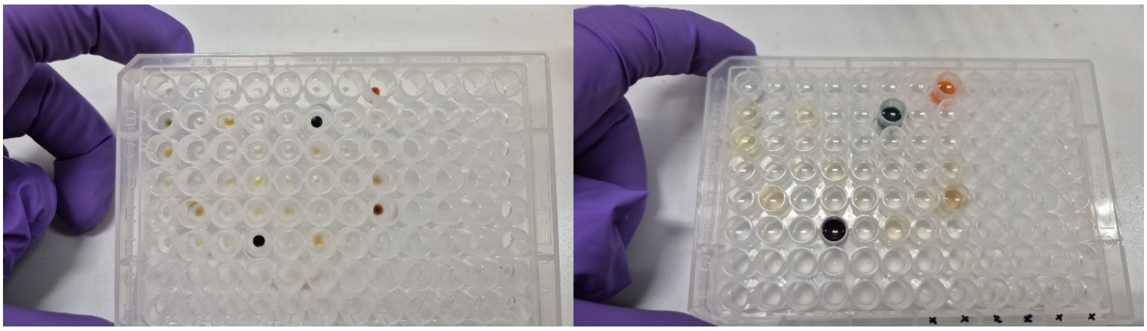

Figure S22. a) 5 µL added directly into the 96-well microplate. b) Samples diluted and filtered.

The analysis of the plate results was carried out, identifying hits when (1) the desired product was detected by MS and (2) a clear DAD absorption for this product was observed, allowing to integrate the corresponding signal against that of the IS to obtain a P/IS ratio. Notably, in 24 cases we detected a clear ion signal for the putative product without a corresponding UV absorbance; these entries were also treated as negative hits. In other cases, where new clear peaks were observed but the mass fragmentation could not be unambiguously ascribed to the desired product, where also labeled as negative hits. With these criteria in hand, we identified significant product formation in 36 cases, accounting for a hit rate of 37/96 = 39%. *Note: The presence of tetrabutylammonium salts rendered the ionization and the detection of some compounds challenging. We would like to note that this aspect, together with the known issues associated to the detection of tertiary alcohols under mass spectrometry, might have been hiding some positive results across the plate.*

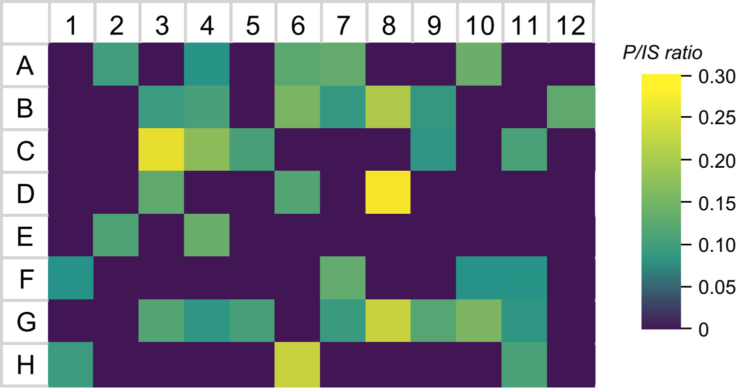

Figure S23. HTE results

13 Mechanistic experiments

13.1 Radical clocks and quenching

<u>TEMPO quenching</u>

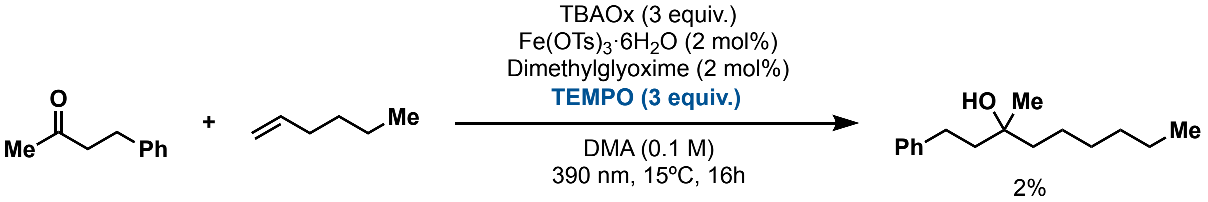

In a microwave vial equipped with a stirring bar were added dimethylglyoxime (0.5 mg, 0.004 mmol, 2 mol%), $Fe(OTs)_3 \cdot 6H_2O$ (2.6 mg, 0.002 mmol, 0.02 equiv.), and TEMPO (93.8 mg, 0.6 mmol, 3 equiv.). The vial was transferred into the glovebox and TBAOx (344 mg, 0.6 mmol, 3.0 equiv.) was charged. The tube was then capped with an aluminium crimp cap with PTFE/butyl septum and taken out of the glovebox. 1 mL of dry and degassed DMA was added to the vial via a syringe under a positive N_2 flow, followed by the 4-phenyl-2-butanone (30 μL, 0.2 mmol, 1.0 equiv.) and 1-hexene (50 μL, 0.4 mmol, 2.0 equiv). The mixture was stirred under 390 nm light irradiation at 15°C for 16 h. After the reaction is completed, the reaction mixture was analyzed by GC-MS and HPLC. HPLC analysis showed the formation of 2% of product, 10% of ketone reduction to secondary alcohol, and 87% of starting material.

Radical clocks

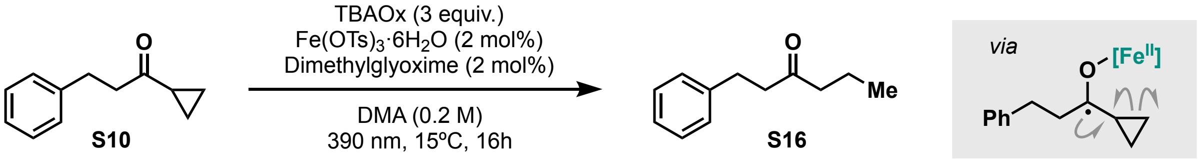

Prepared following general procedure **GP1**, starting from **S10** (34.8 μL, 0.2 mmol, 1 equiv.). The crude mixture was purified by flash column chromatography on silica gel using a mixture of Cyclohexane to Cyclohexane/ EtOAc 9/1 to provide product **S16** as a yellow oil (24.8 mg, 62 %).

^{1}H NMR (400 MHz, $CDCl_3$) δ 7.31 – 7.27 (m, 3H), 7.22 – 7.16 (m, 2H), 2.90 (dd, J = 8.3, 6.9 Hz, 2H), 2.80 – 2.65 (m, 2H), 2.37 (t, J = 7.3 Hz, 2H), 1.60 (h, J = 7.4 Hz, 2H), 0.90 (t, J = 7.4 Hz, 3H).

^{13}C NMR (101 MHz, $CDCl_3$) δ 210.3, 141.3, 128.6, 128.4, 126.2, 45.1, 44.4, 29.9, 17.4, 13.9.

HRMS(EI) m/z calcd for $C_{12}H_{16}O$ $[M+]^+$: 176.1201, found: 176.1189.

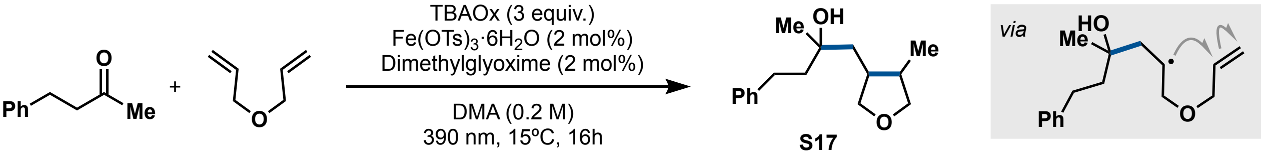

Prepared following general procedure **GP1**, starting from 4-phenyl-2-butanone (30 μL, 0.2 mmol, 1.0 equiv.) and diallyl ether (98 μL, 0.8 mmol, 4.0 equiv.). The crude mixture was purified by flash column chromatography on a silica gel column using a mixture of Cyclohexane to Cyclohexane/EtOAc (8:2) to provide the desired product **S17** as a pale-yellow oil (19.8 mg, 40%, d.r. 4:1).

^{1}H NMR (400 MHz, $CDCl_3$) δ 7.31 – 7.26 (m, 2H), 7.22 – 7.16 (m, 3H), 4.18 (dd, J = 8.6, 6.7 Hz, 0.2H), 4.03 (dt, 0.8H), 3.98 – 3.93 (m, 0.3H), 3.92 – 3.85 (m, .0.9H), 3.53 – 3.42 (m, 2H), 3.33 – 3.24 (m, 0.2H), 2.74 – 2.63 (m, 1.9H), 2.44 – 2.33 (m, 1H), 2.33 – 2.21 (m, 1H), 1.82 – 1.77 (m, 2H) 1.76 – 1.74 (m, 0.4H), 1.52 – 1.39 (m, 2H), 1.27 (s, 2H), 1.26 (s, 1H), 1.04 (d, J = 6.1 Hz, 1H), 0.95 (dd, J = 7.1, 1.9 Hz, 2H).

^{13}C NMR (101 MHz, $CDCl_3$) δ 142.4, 142.4, 128.6, 128.4, 126.0, 126.0, 75.2, 75.2, 75.2, 74.3, 74.2, 72.7, 72.7, 72.5, 72.5, 45.4, 45.3, 44.4, 44.3, 44.2, 44.0, 43.4, 43.1, 40.9, 40.9, 39.7, 39.7, 38.1, 38.0, 37.1, 37.1, 30.7, 30.6, 30.5, 30.4, 28.0, 27.8, 26.8, 15.6, 15.6, 13.8, 13.8.

HRMS(ESI) m/z calcd for $C_{16}H_{24}O_2$ $[M+H]^+$:271.1669, found: 271.1671.

13.2 Light ON/OFF experiments

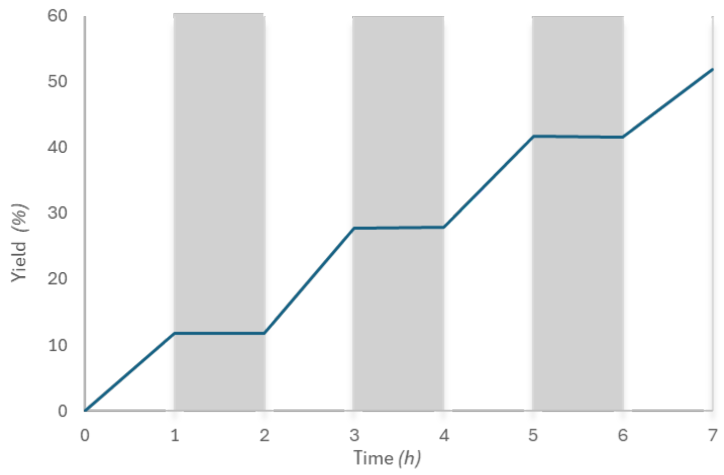

Figure S24. ON/OFF experiment

Conclusion: The reaction needs continuous irradiation to proceed.

13.3 Evidence on inner-sphere reactivity with ketones

Competition experiments between different ketone substrates were performed to assess the influence of their reduction potential, measured by cyclic voltammetry, on their relative reactivity. The obtained ratios were determined by HPLC analysis using authentic samples. The obtained results are given below:

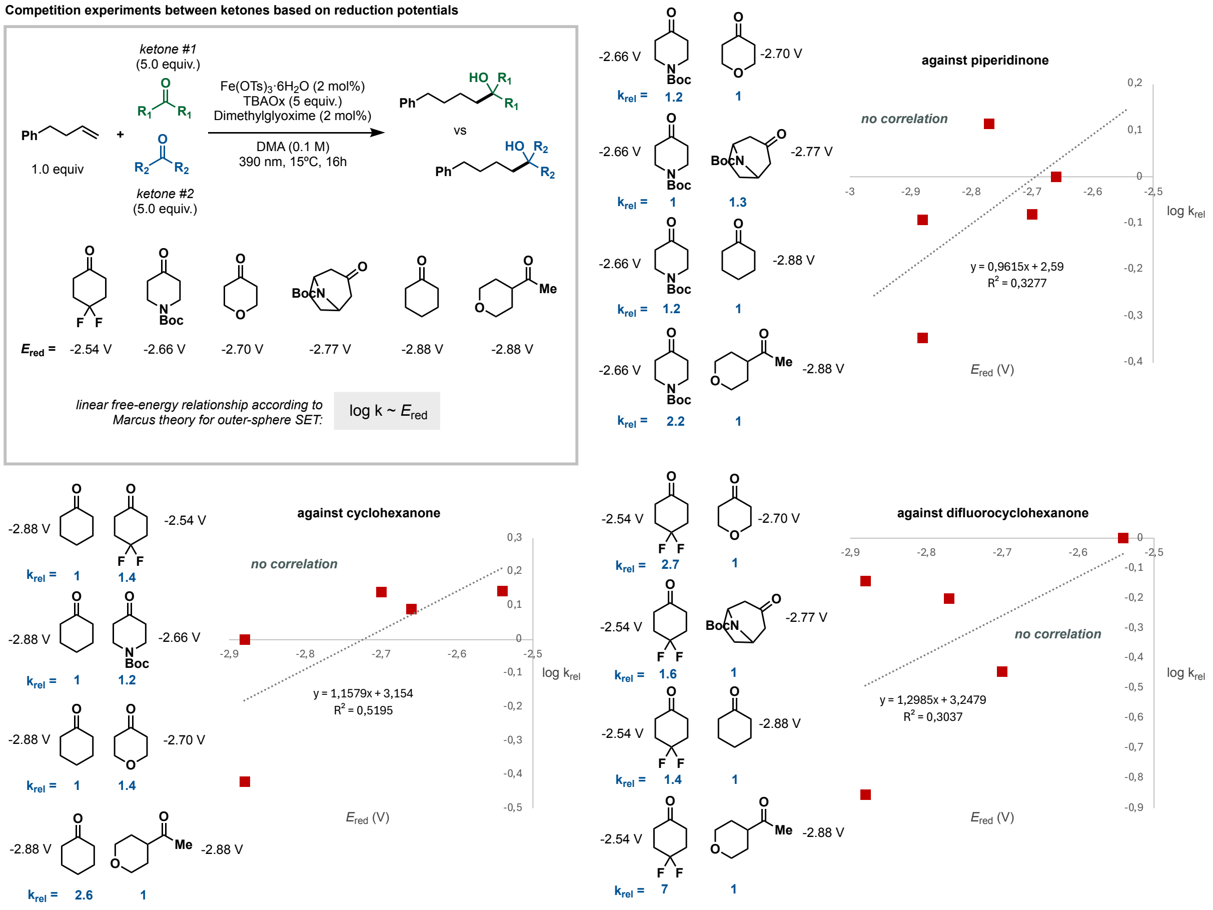

Figure S25. Reactivity of ketones according to redox potentials

The results shown in Figure S25 display a lack of correlation between the reactivity of ketones according to their reduction potentials, which differs from the expected behaviour for outer-sphere SET.[12]

Similarly, competition of ketones involving sterically hindered substrates was interrogated:

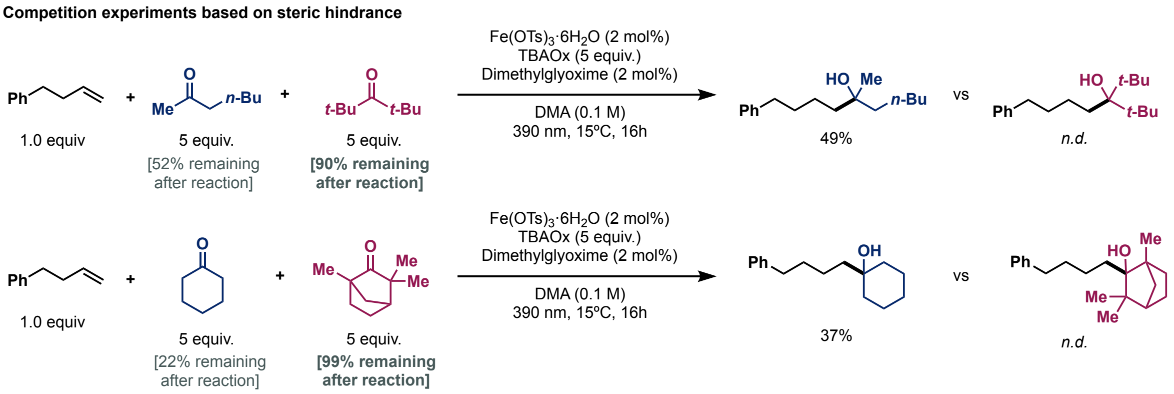

Figure S26. Reactivity of ketones according to redox potentials

The results in Figure S26 show a substantial lack of reactivity displayed by sterically hindered ketones, which could be recovered unreacted after the reaction, for both cyclic and acyclic substrates.

Conclusion: The lack of correlation on the reactivity of ketones with their redox potential, together with the marked sensitivity to steric hindrance, collectively support an inner-sphere mechanism for the activation of carbonyls to produce ketyl radicals under our reaction conditions.

13.4 Comparison between inner-sphere and outer-sphere SET activation

With the aim of assessing the synthetic divergencies arising from different mechanism on ketyl radical generation, we performed a direct comparison of our work based on inner-sphere activation with Xia's method (*Org. Lett.* **2026**, *28*, 2213-2218, cited on the main text as reference 29) based on outer-sphere SET via photoredox catalysis. The results are shown below:

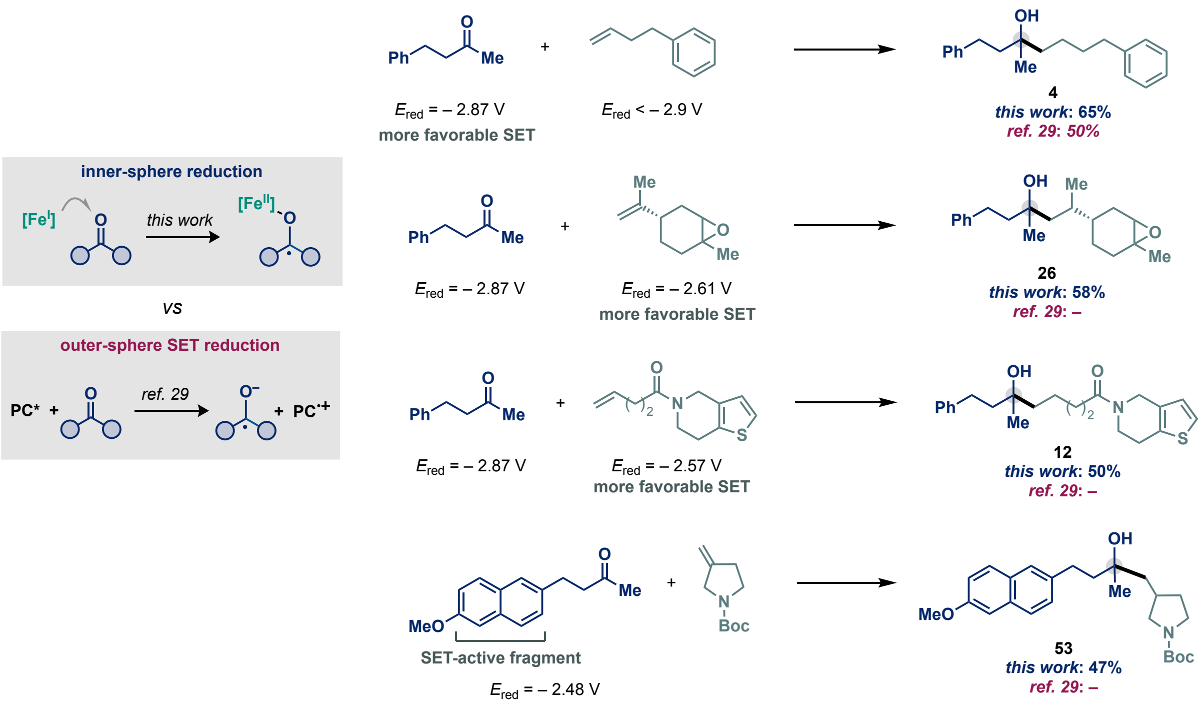

Figure S27. Comparison of this work with Xia's method

As can be appreciated in Figure S27, we were able to nicely reproduce the reactivity of this alternative method for substrate **4**, showing that both inner-sphere and outer-sphere strategies can work effectively when other redox-active species are not present. However, the presence of other functionalities displaying more accessible reduction potential than the ketone (**26**, **12**, **53**) failed to deliver product under ref. 29's conditions. Considering the challenging reduction of inactivated ketones, requiring extremely negative redox potentials, this aspect can result an issue for strategies that generate ketyl radical via outer-sphere SET reduction. On the other hand, the broad compatibility of our method to different functionalities is a direct consequence of the inner-sphere activation of ketones, which makes possible to produce ketyl radicals from ketones in presence of reducible functionalities with more accessible redox potential.

13.5 Evidence on alkyl Fe intermediate

A) parasitic β-hydride elimination product

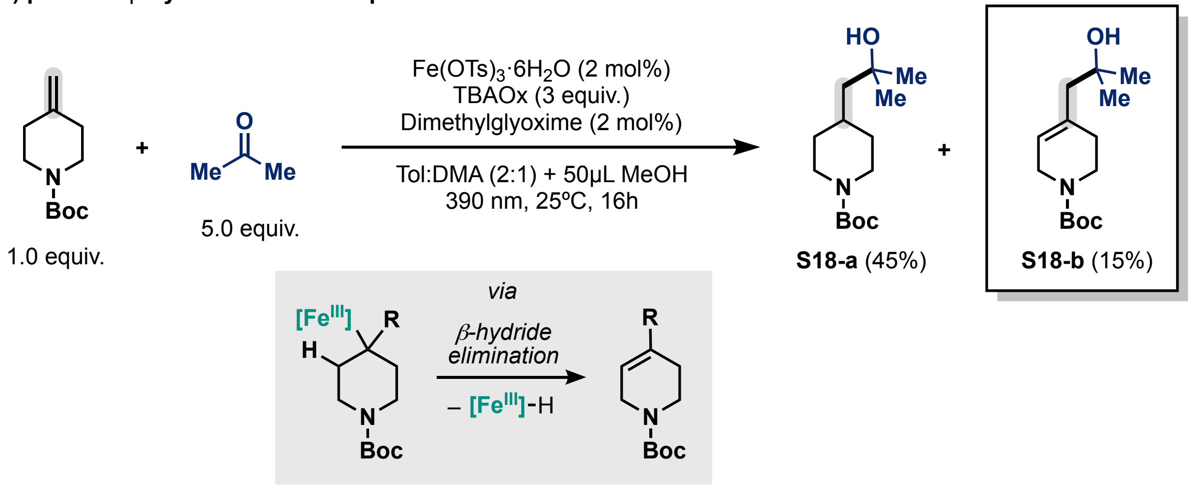

Prepared following general procedure **GP2**, starting from tert-butyl 4-methylenepiperidine-1-carboxylate (79 mg, 0.4 mmol, 1.0 equiv.) and acetone (148 µL, 2.0 mmol, 5.0 equiv.). The crude mixture was purified by flash column chromatography on a silica gel column using a mixture DCM/Acetone (9:1) + 5% V/V toluene to provide the desired product **S18-a** as a yellow oil (46.3 mg, 45%) and **S18-b** as a pale-yellow oil (33.0 mg, 15%).

***tert*-butyl 4-(2-hydroxy-2-methylpropyl)piperidine-1-carboxylate (S18-a)**

^{1}H NMR (400 MHz, $CDCl_3$) δ 4.02 (d, J = 12.4 Hz, 2H), 2.71 (t, J = 12.8 Hz, 2H), 1.78 – 1.71 (m, 2H), 1.66 – 1.57 (m, 1H), 1.44 (s, 9H), 1.41 (d, J = 5.7 Hz, 2H), 1.23 (s, 6H), 1.21 – 1.10 (m, 2H).

^{13}C NMR (101 MHz, $CDCl_3$) δ 155.0, 79.3, 71.6, 50.2, 44.1, 34.0, 32.5, 30.3, 28.6.

HRMS (ESI) m/z calcd for $C_{14}H_{27}NO_3$ $[M]^+$: 257.1991, found 257.1979.

***tert*-butyl 4-(2-hydroxy-2-methylpropyl)-3,6-dihydropyridine-1(2*H*)-carboxylate (S18-b)**

^{1}H NMR (400 MHz, $CDCl_3$) δ 5.45 (s, 1H), 3.89 (s, 2H), 3.46 (t, J = 5.6 Hz, 2H), 2.18 (s, 4H), 1.47 (s, 9H), 1.22 (s, 6H).

^{13}C NMR (101 MHz, $CDCl_3$) δ 155.1, 134.1, 122.7, 79.7, 71.7, 51.2, 43.6, 39.9, 31.0, 29.9, 28.6.

HRMS (ESI) m/z calcd for $C_{14}H_{25}NO_3$ $[M]^+$: 255.1834, found 255.1821.

B) polar elimination in presence of a α-leaving group

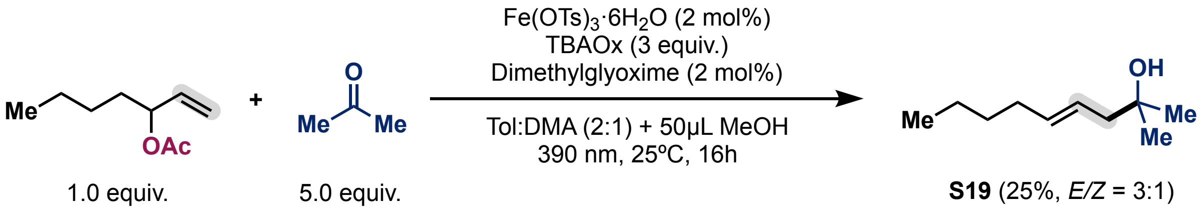

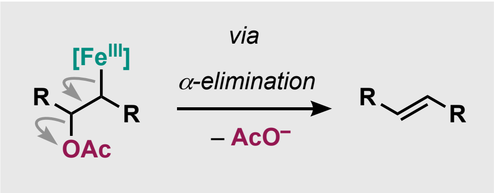

Prepared following general procedure **GP2**, starting from oct-1-en-3-yl acetate (60 µL, 0.3 mmol, 1.0 equiv.) and acetone (150 µL, 1.5 mmol, 5.0 equiv.). The crude mixture was purified by flash column chromatography on a silica gel column using a mixture Pentane/Ether (7:3) to provide the desired product **S19** as a pale-yellow oil (12.8 mg, 25%).

^{1}H NMR (400 MHz, $CDCl_3$) δ 5.63 – 5.27 (m, 2H), 2.29 – 2.05 (m, 2H), 2.04 – 1.90 (m, 2H), 1.33 – 1.18 (m, 6H), 1.21-1.13 (m, 3H), 0.86 – 0.74 (m, 3H).

^{13}C NMR (101 MHz, $CDCl_3$) δ 135.5, 133.8, 125.1, 124.4, 71.1, 70.4, 46.9, 41.2, 32.7, 31.6, 31.4, 29.3, 29.2, 29.1, 29.0, 27.3, 22.6, 22.5, 14.1, 14.1.

Conclusion: Both reactivities observed are in line with the intermediacy of an alkyl Fe intermediate under these reaction conditions

13.6 Deuteration labelling studies

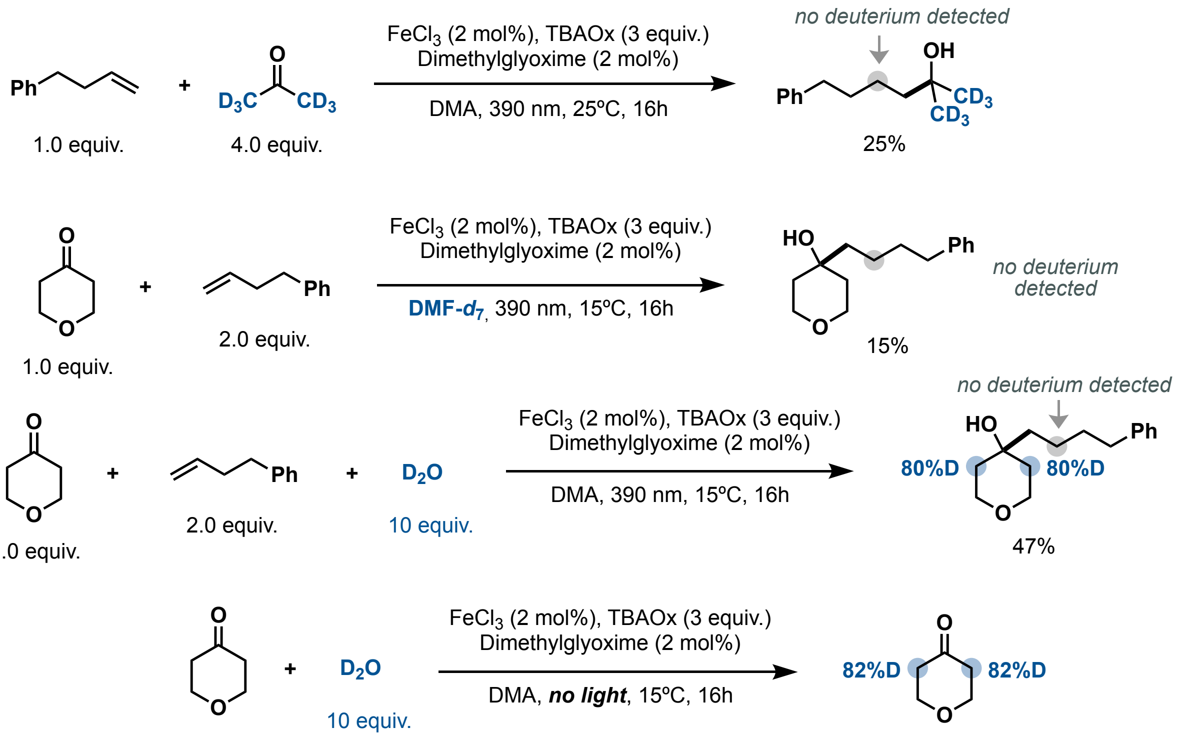

We did not observe deuterium incorporation across the alkene neither from the ketone nor from the solvent. However, when using D_2O deuteration was not observed either, which could be due to the ineffective removal of residual H_2O on TBAOx. Furthermore, as can be noticed from the results above, D-incorporation was taking place in the ketone via enolization, which further complicates the analysis.

The lack of suitable labelling experiments, which we ascribe to the ineffective removal of residual H_2O on TBAOx, precluded us from demonstrating unequivocally the proton source. However, in view of the previous studies on H-incorporation from H_2O to alkyl radical intermediates under ferrioxalate photocatalysis (*Science* **2026**, *678*, 84-89), together with the markedly detrimental effect of molecular sieves on this reaction (Table S1, entry 18), we consider water as the most plausible source of hydrogen in this reaction.

14 Cyclic voltammetry

Cyclic voltammetry was conducted on an EmStat (PalmSens) potentiostat using a 3-electrode cell configuration. A glassy carbon working electrode was employed alongside a platinum wire counter electrode and a Ag/AgCl reference electrode. All the solutions were degassed by bubbling N_2 prior to measurements. The solutions of the desired compounds were freshly prepared in dry acetonitrile or in DMF along with 0.1 M of tetrabutylammonium hexafluorophosphate as supporting electrolyte and were examined at a scan rate of 0.1 V s^{-1}. Ferrocene ($E_{1/2}$ = +0.42 V vs SCE)[13] was added at the end of the measurements as an internal standard to determine the precise potential scale. Potential values are given versus the saturated calomel electrode (SCE). Irreversible waves were obtained in all cases; therefore, the potentials were estimated at half the maximum current, as previously described by Nicewicz.[14]

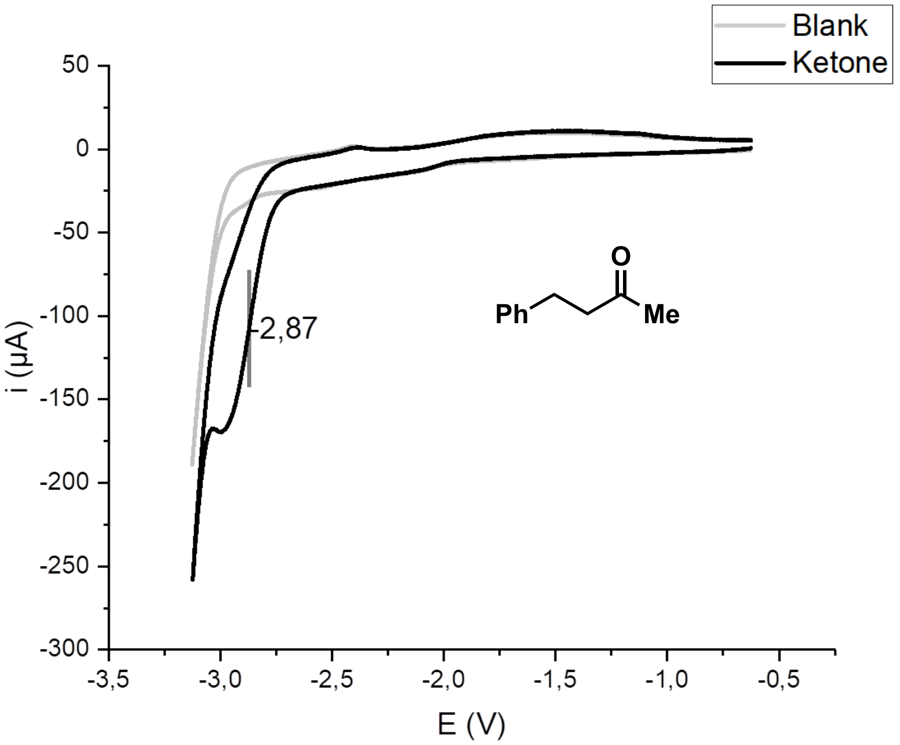

Blank
Ketone
-2,87
Ph
O
Me
i (µA)
E (V)

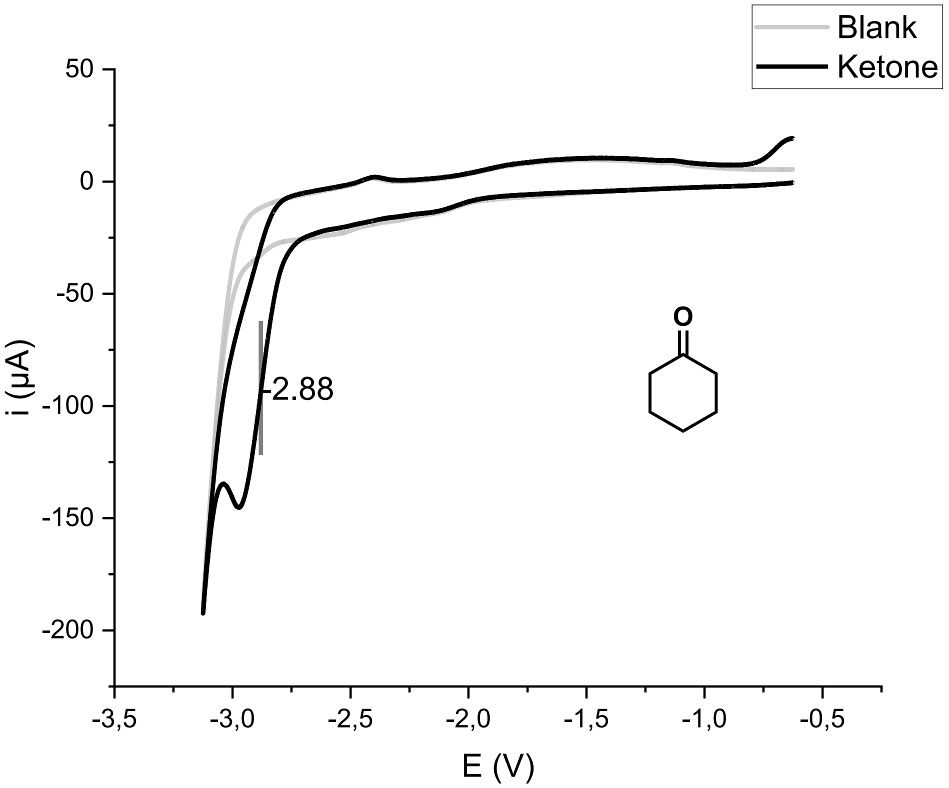

Blank
Ketone
-2.88
O
i (µA)
E (V)

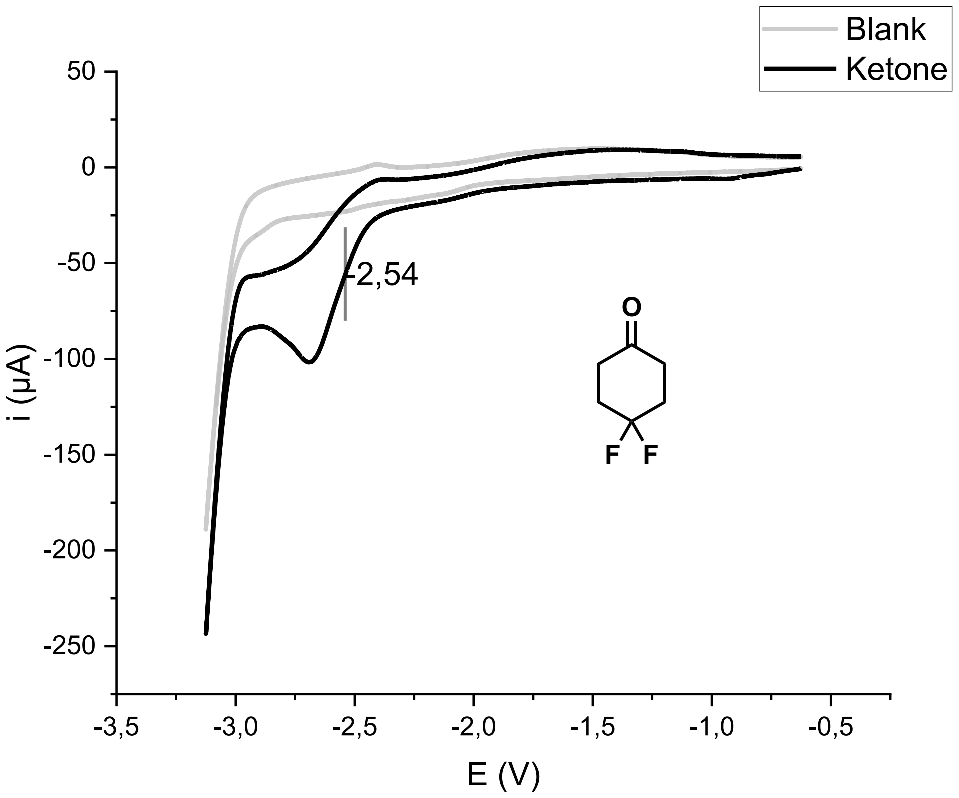
Blank
Ketone
-2,54
i (µA)
E (V)
O
F F

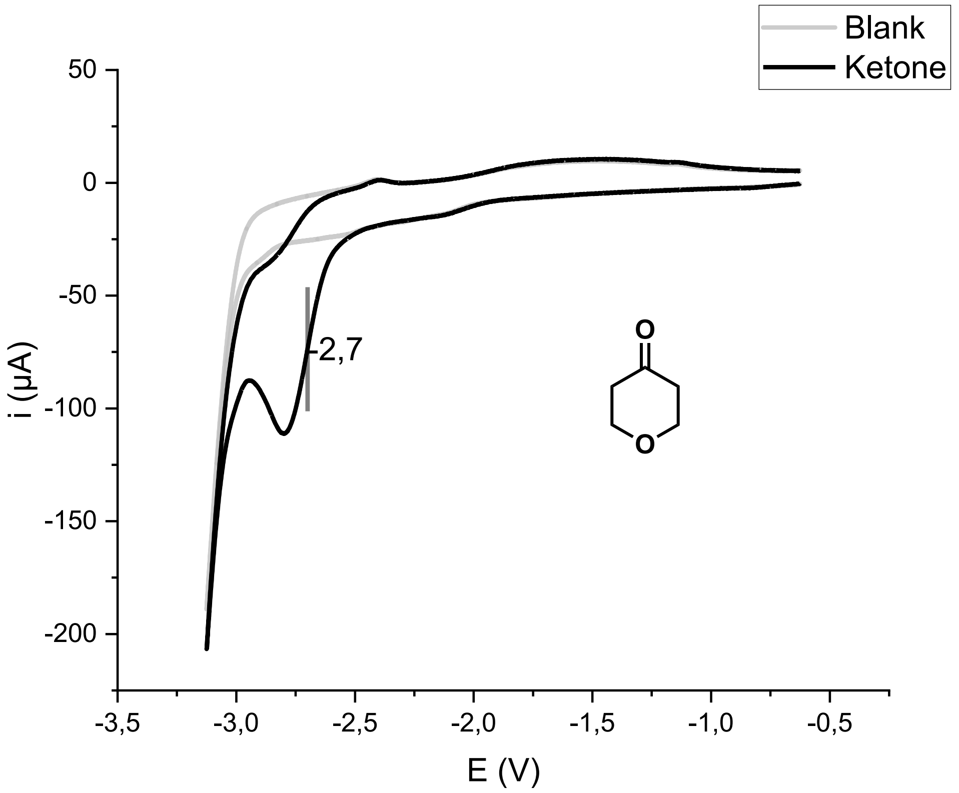
Blank
Ketone
-2,7
i (µA)
E (V)
O
O

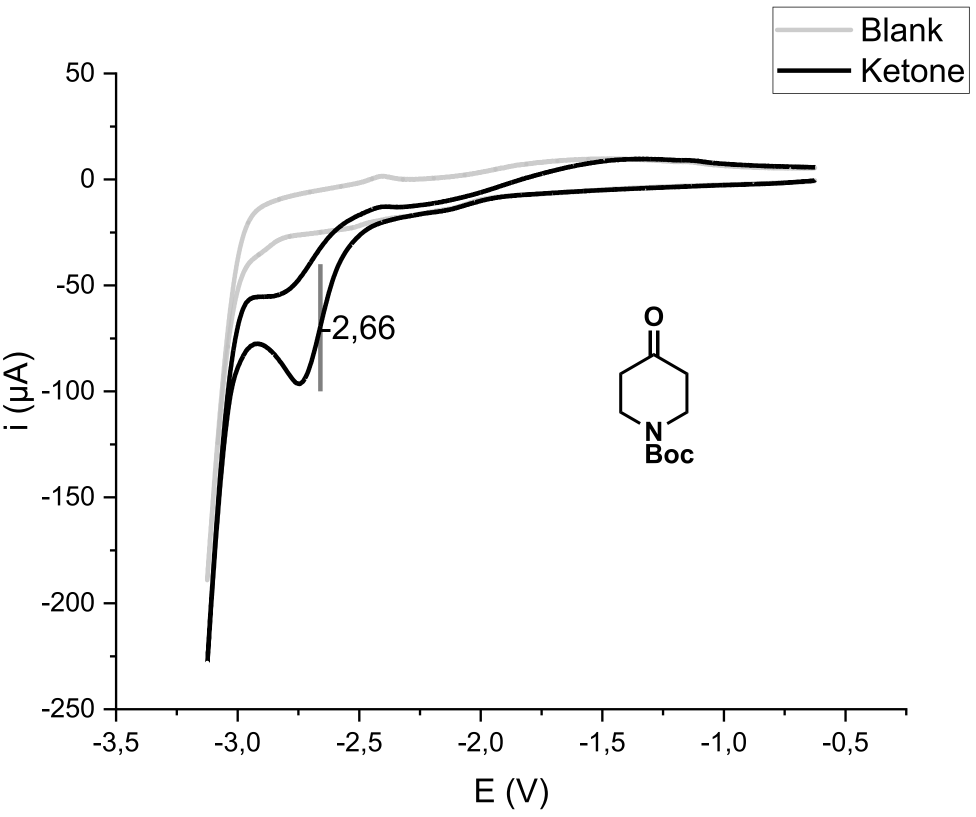
Blank
Ketone
50
0
-50
-100
-150
-200
-250
i (µA)
-3,5
-3,0
-2,5
-2,0
-1,5
-1,0
-0,5
E (V)
-2,66
O
N
Boc

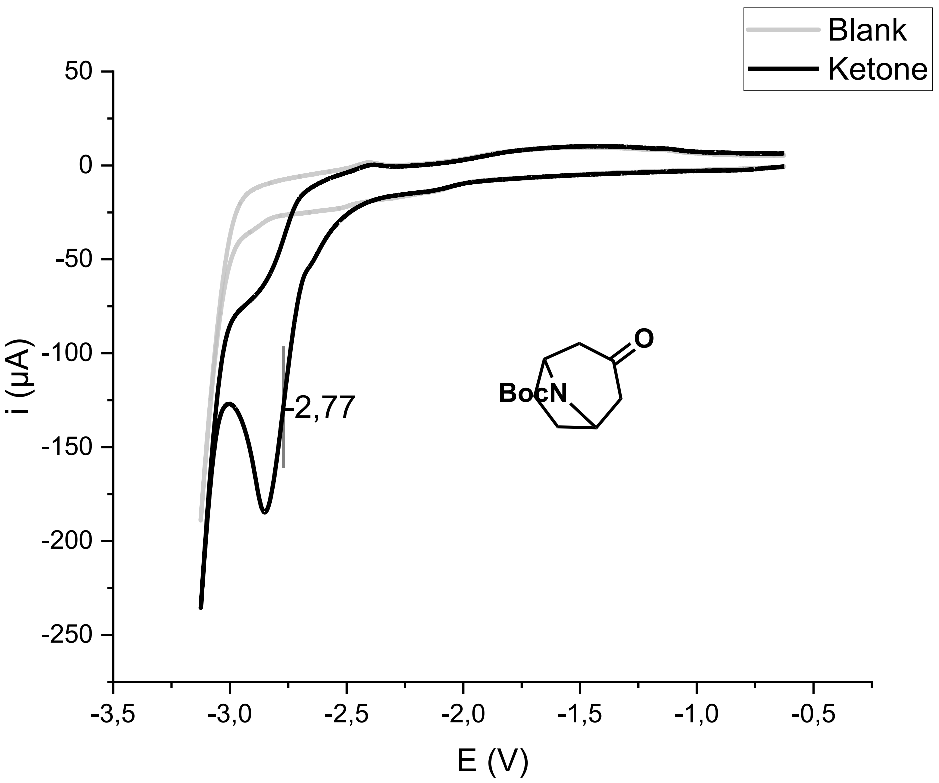
Blank
Ketone
50
0
-50
-100
-150
-200
-250
i (µA)
-3,5
-3,0
-2,5
-2,0
-1,5
-1,0
-0,5
E (V)
-2,77
O
BocN

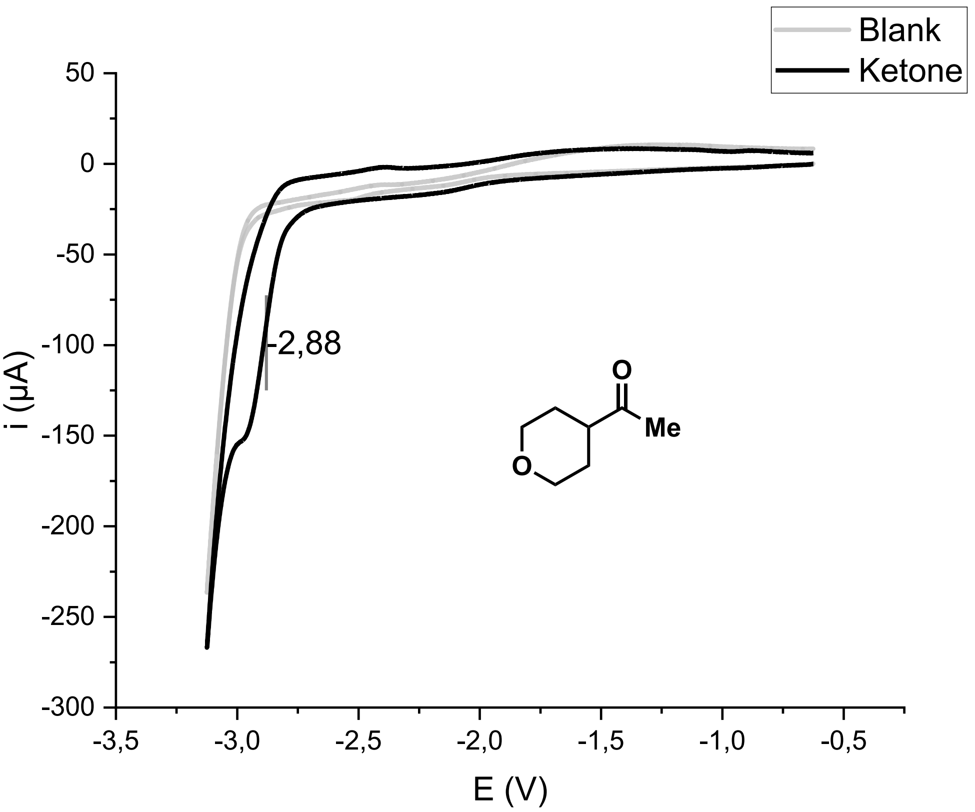
Blank
Ketone
50
0
-50
-100
-150
-200
-250
-300
i (µA)
-3,5
-3,0
-2,5
-2,0
-1,5
-1,0
-0,5
E (V)
-2,88
O
Me
O

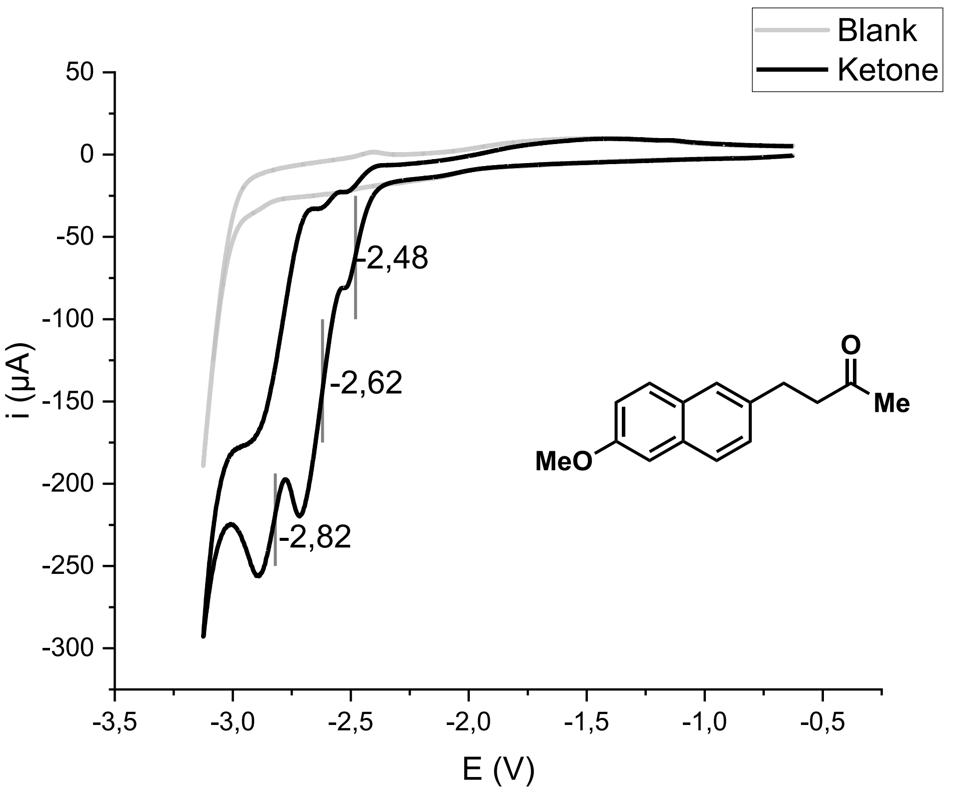
Blank
Ketone
50
0
-50
-100
-150
-200
-250
-300
i (µA)
-3,5
-3,0
-2,5
-2,0
-1,5
-1,0
-0,5
E (V)
-2,48
-2,62
-2,82
O
Me
MeO

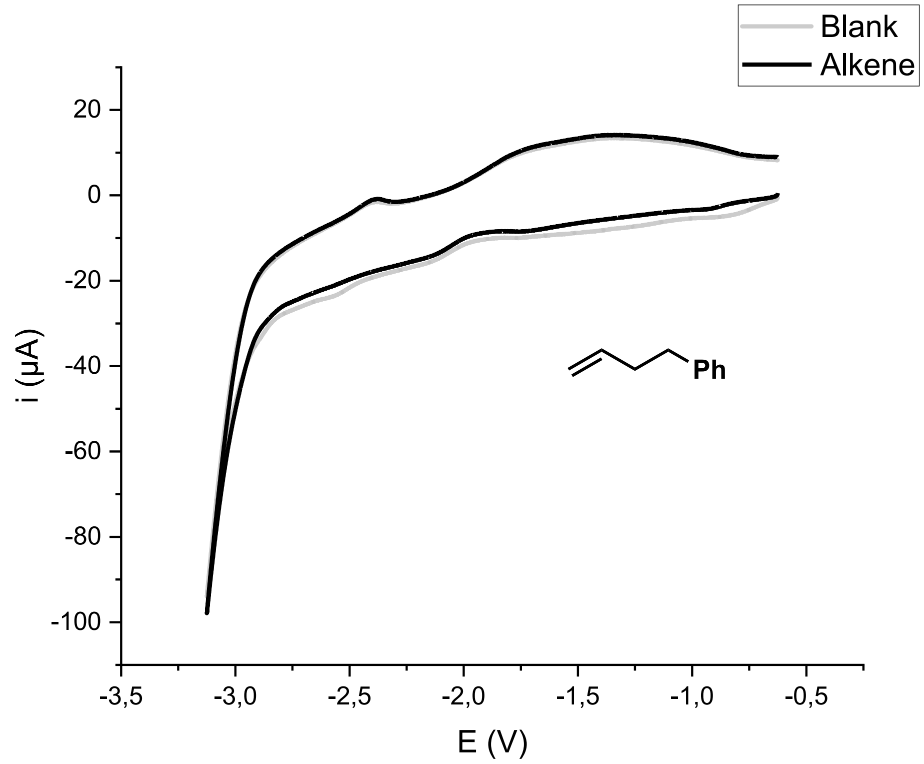
Blank
Alkene
20
0
-20
-40
-60
-80
-100
i (µA)
-3,5
-3,0
-2,5
-2,0
-1,5
-1,0
-0,5
E (V)
Ph

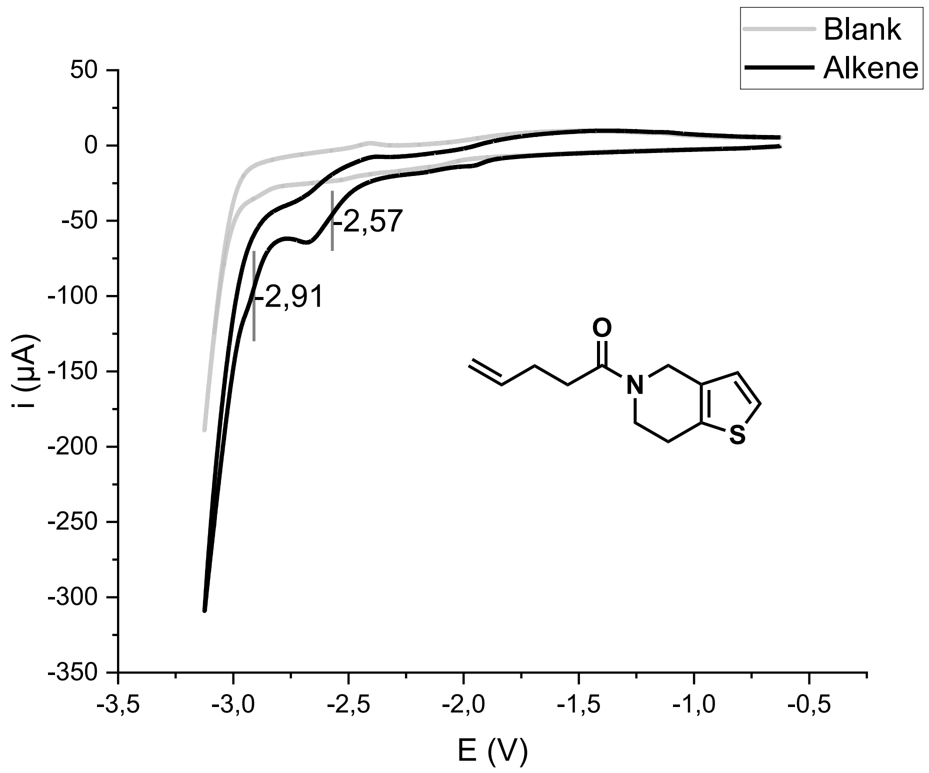
Blank
Alkene
50
0
-50
-100
-150
-200
-250
-300
-350
i (µA)
-2,57
-2,91
-3,5
-3,0
-2,5
-2,0
-1,5
-1,0
-0,5
E (V)
O
N
S

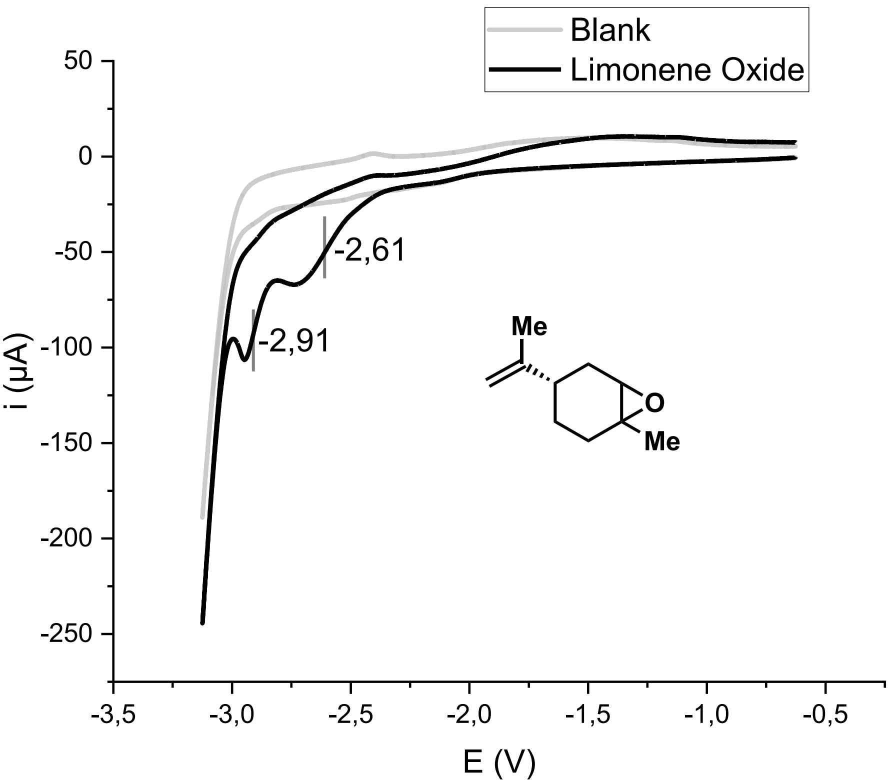
Blank
Limonene Oxide
-2,61
-2,91
Me
O
Me
i (µA)
E (V)
50
0
-50
-100
-150
-200
-250
-3,5
-3,0
-2,5
-2,0
-1,5
-1,0
-0,5

15 References

[1] C. Bernabeu, S. Adalid, S. Colombo, N. Cironis, P. P. Sen, K. Okuno, F. Juliá, "Ferrioxalate photocatalysis: A multitasking platform for reductive iron catalysis" *Science* **2026**, *391*, 84–89.

[2] W. Yu, T. Gill, L. Wang, Y. Du, H. Ye, X. Qu, J.-T. Guo, A. Cuconati, K. Zhao, T. M. Block, X. Xu, J. Chang, "Design, Synthesis, and Biological Evaluation of *N* -Alkylated Deoxynojirimycin (DNJ) Derivatives for the Treatment of Dengue Virus Infection" *J. Med. Chem.* **2012**, *55*, 6061–6075.

[3] L. Pitzer, F. Schäfers, F. Glorius, "Rapid Assessment of the Reaction-Condition-Based Sensitivity of Chemical Transformations" *Angew. Chem. Int. Ed.* **2019**, *58*, 8572–8576.

[4] N.-H. Yeh, R. Krueger, K. D. Moeller, "Microelectrode Arrays, Dihydroxylation, and the Development of an Orthogonal Safety-Catch Linker" *Org. Lett.* **2021**, *23*, 5440–5444.

[5] F. Cong, G.-Q. Sun, S.-H. Ye, R. Hu, W. Rao, M. J. Koh, "A Bimolecular Homolytic Substitution-Enabled Platform for Multicomponent Cross-Coupling of Unactivated Alkenes" *J. Am. Chem. Soc.* **2024**, *146*, 10274–10280.

[6] D. M. Schultz, F. Lévesque, D. A. DiRocco, M. Reibarkh, Y. Ji, L. A. Joyce, J. F. Dropinski, H. Sheng, B. D. Sherry, I. W. Davies, "Oxyfunctionalization of the Remote C−H Bonds of Aliphatic Amines by Decatungstate Photocatalysis" *Angew. Chem. Int. Ed.* **2017**, *56*, 15274–15278.

[7] S. N. Alektiar, J. Han, Y. Dang, C. Z. Rubel, Z. K. Wickens, "Radical Hydrocarboxylation of Unactivated Alkenes via Photocatalytic Formate Activation" *J. Am. Chem. Soc.* **2023**, *145*, 10991–10997.

[8] R. Dawes, B. Jiang, H. Guo, "UV Absorption Spectrum and Photodissociation Channels of the Simplest Criegee Intermediate (CH_2 OO)" *J. Am. Chem. Soc.* **2015**, *137*, 50–53.

[9] B. D. Kelly, J. M. Allen, R. E. Tundel, T. H. Lamber, "Multicatalytic Synthesis of Complex Tetrahydrofurans Involving Bismuth(III) Triflate Catalyzed Intramolecular Hydroalkoxylation of Unactivated Olefins" *Org. Lett.* **2009**, *11 (6)*, 1381-1383.

[10] G. A. Molander, C. Kenny, "Intramolecular Reductive Coupling Reactions Promoted by Samarium Diiodide", *J. Am. Chem. Soc.,* **1989**, *111* (21), 8236−8246.

[11] D. W. Hansen, Jr., A. K. Awasthi, T. J. Hagen, E. Ann Hallinan, S. Metz, B. S. Pitzele, A. E. Moormann, "Heterocyclic and Tricyclic Nitric Oxide Synthase Inhibitors" WO Pat. 99/64426, 1999.

[12] C. L. Wong, J. K. Kochi, "Electron transfer with organometals. Steric effects as probes for outer-sphere and inner-sphere oxidations of homoleptic alkylmetals with iron(III) and iridate(IV) complexes" *J. Am. Chem. Soc.* **1979**, *101*, 5593–5603.

[13] V. V. Pavlishchuk, A. W. Addison, "Conversion constants for redox potentials measured versus different reference electrodes in acetonitrile solutions at 25°C" *Inorganica Chim. Acta* **2000**, *298*, 97–102.

[14] H. Roth, N. Romero, D. Nicewicz, "Experimental and Calculated Electrochemical Potentials of Common Organic Molecules for Applications to Single-Electron Redox Chemistry" *Synlett* **2015**, *27*, 714–723.

16 NMR Spectra

3 – ^{1}H NMR (400 MHz, CDCl$_3$)

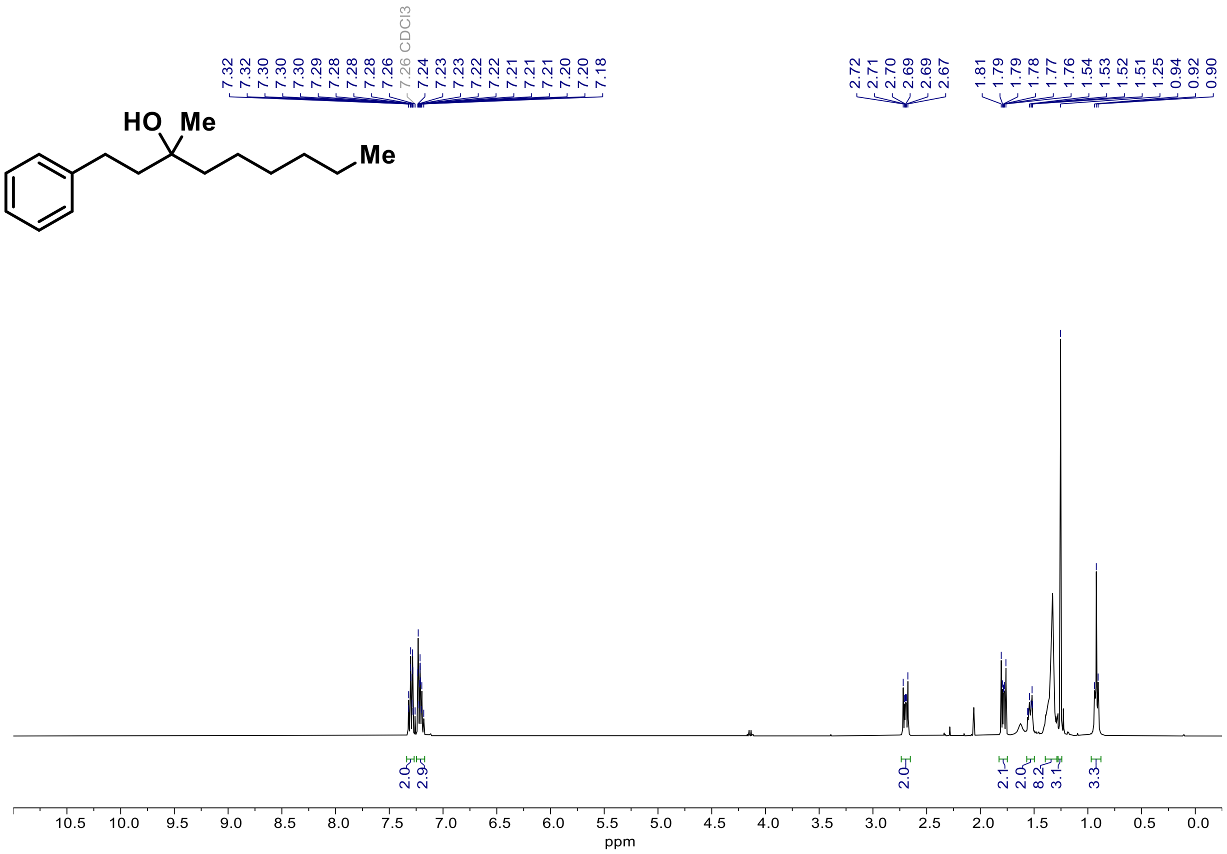

3 – ^{13}C NMR (101 MHz, CDCl$_3$)

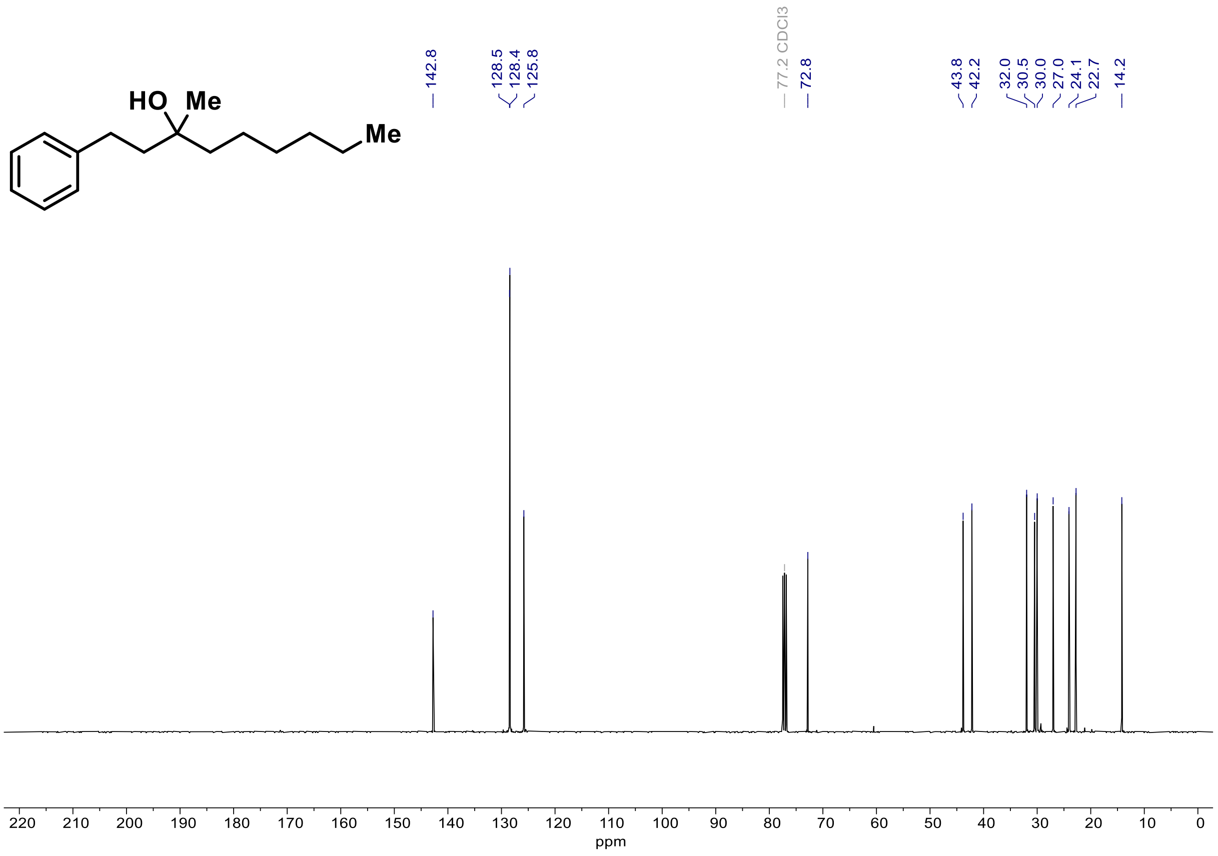

4 – ^{1}H NMR (400 MHz, CDCl$_3$)

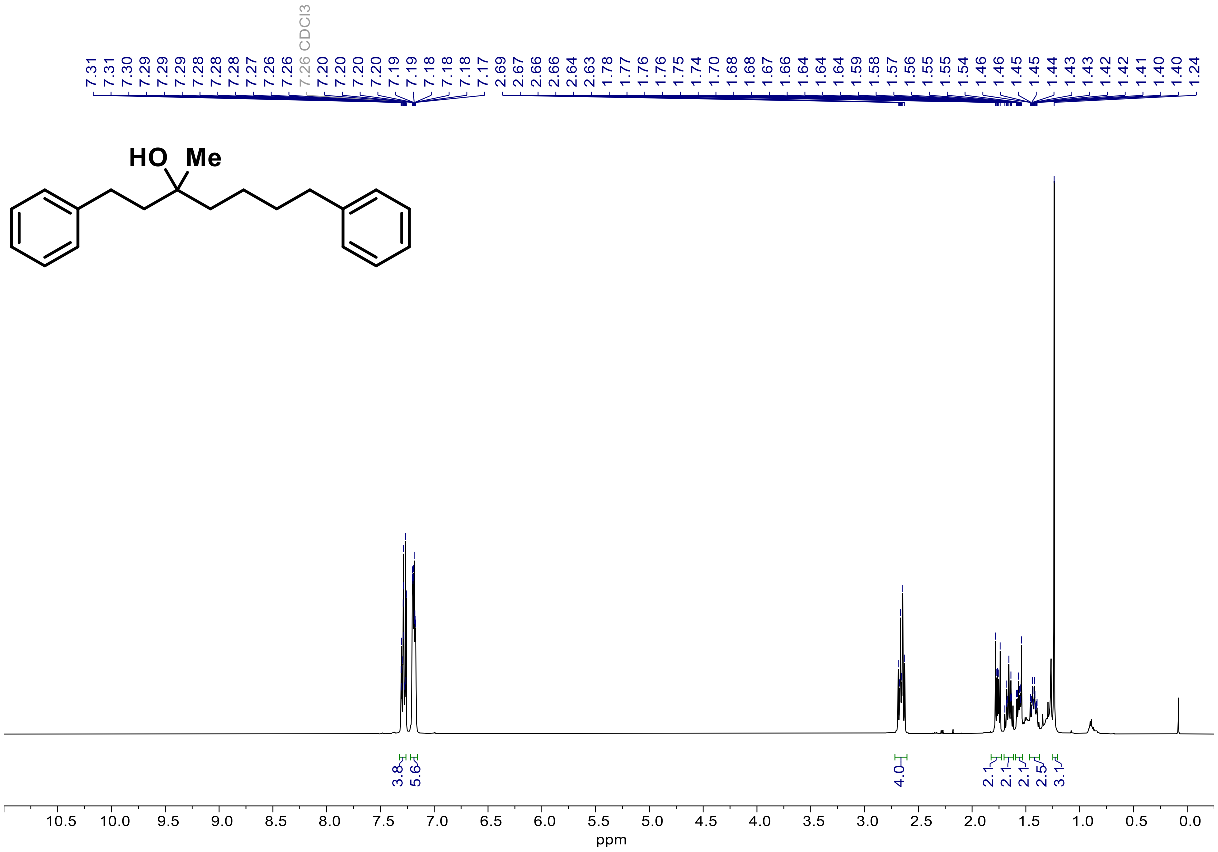

4 – ^{13}C NMR (101 MHz, CDCl$_3$)

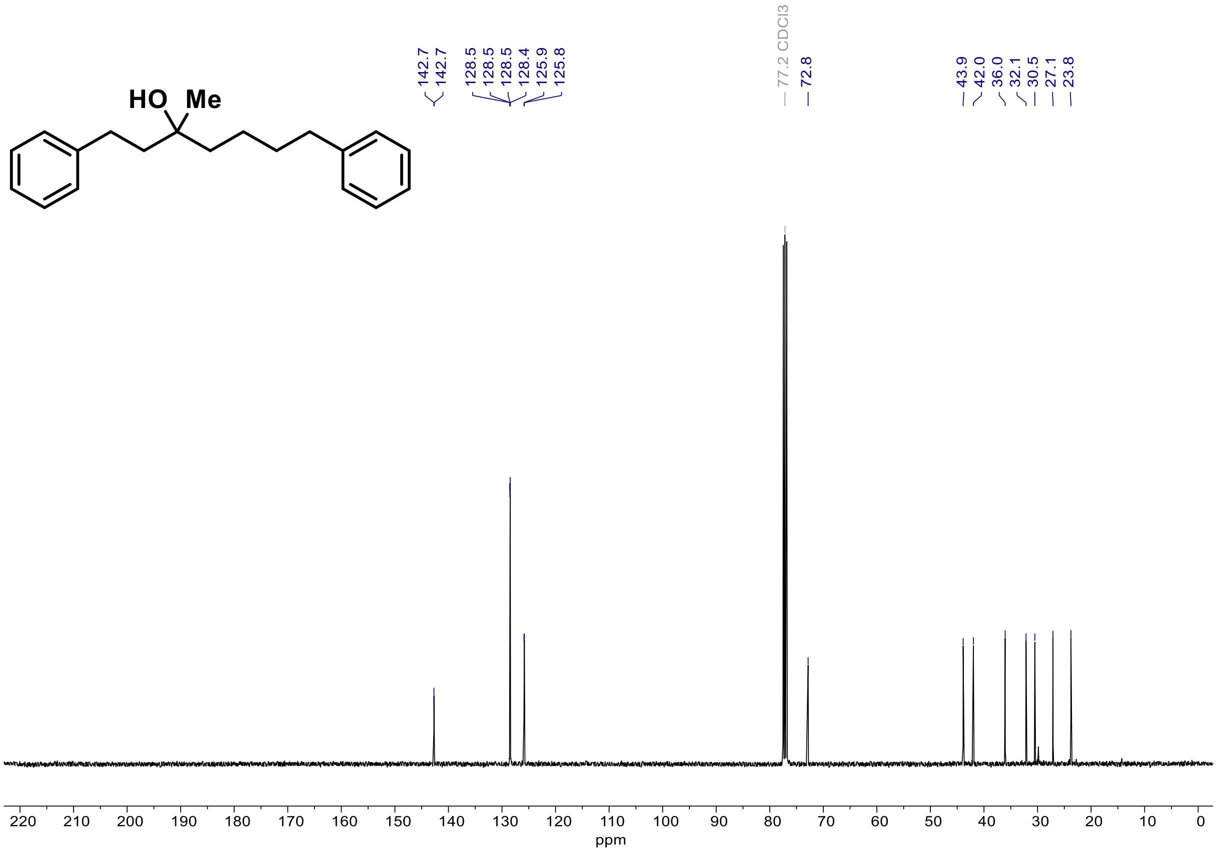

5 – ^{1}H NMR (400 MHz, $CDCl_3$)

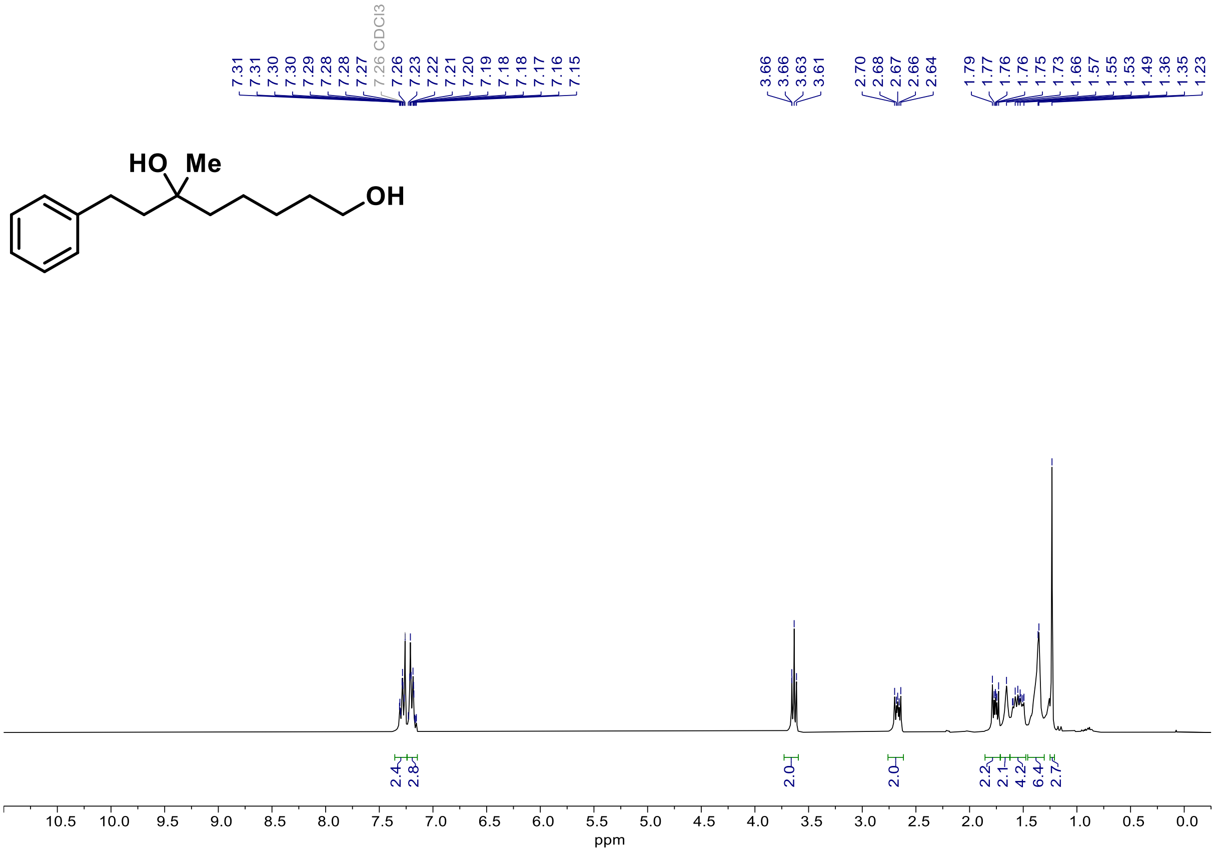

5 – ^{13}C NMR (101 MHz, $CDCl_3$)

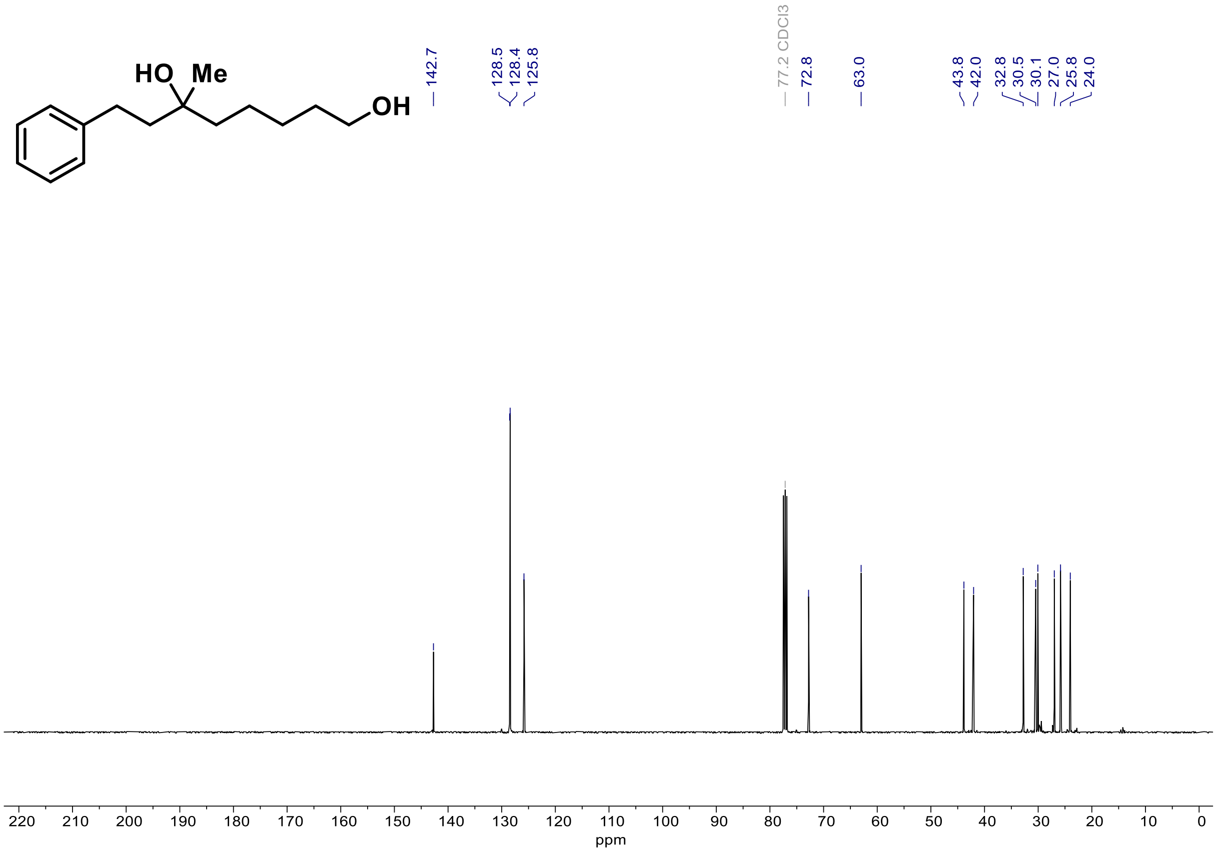

6 – ^{1}H NMR (400 MHz, $CDCl_3$)

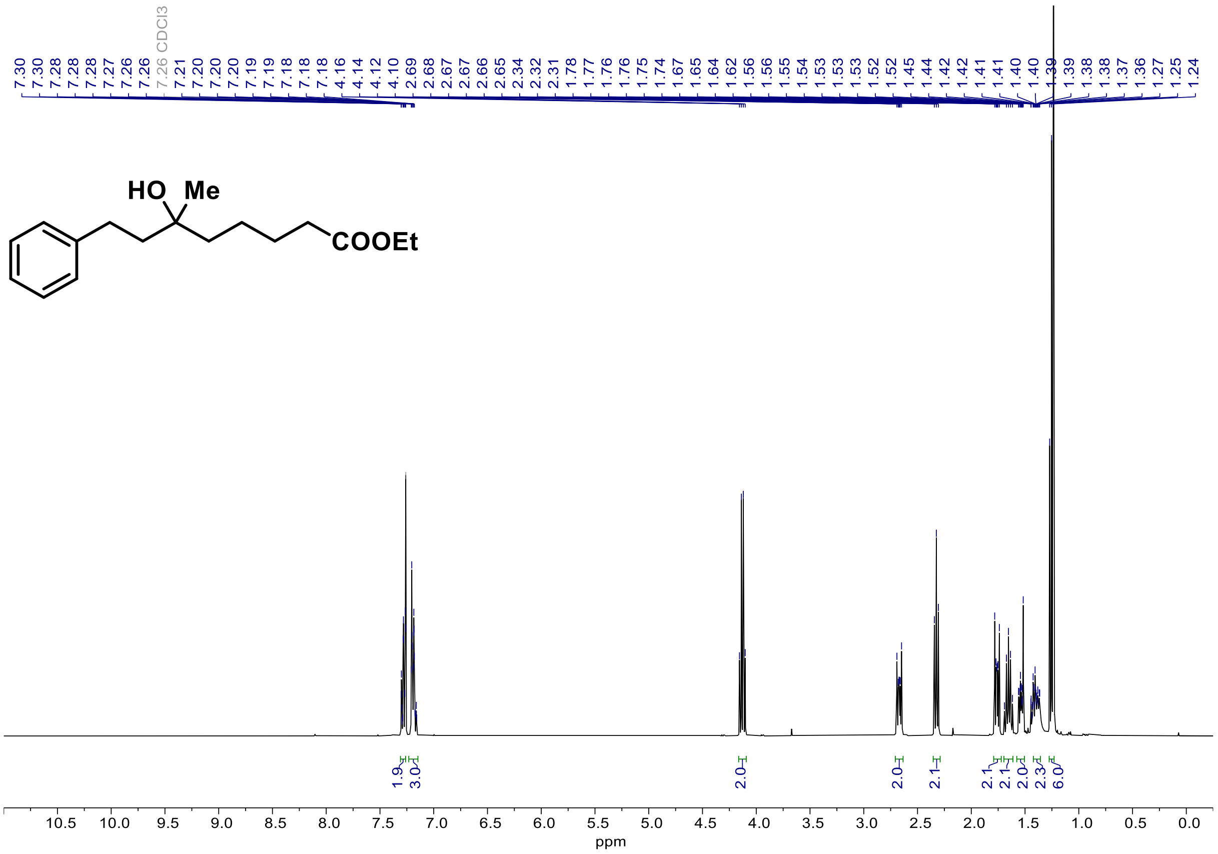

6 – ^{13}C NMR (101 MHz, $CDCl_3$)

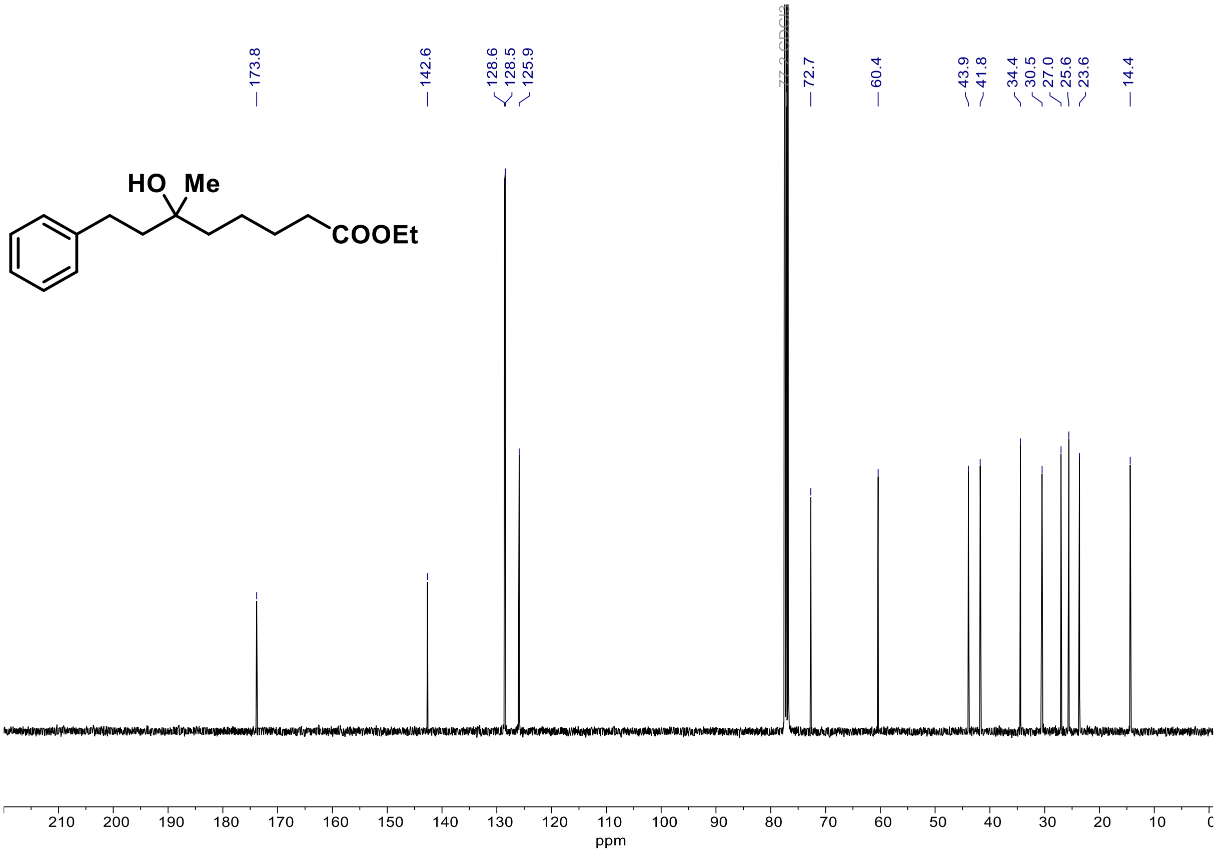

7 – ^{1}H NMR (400 MHz, CDCl$_3$)

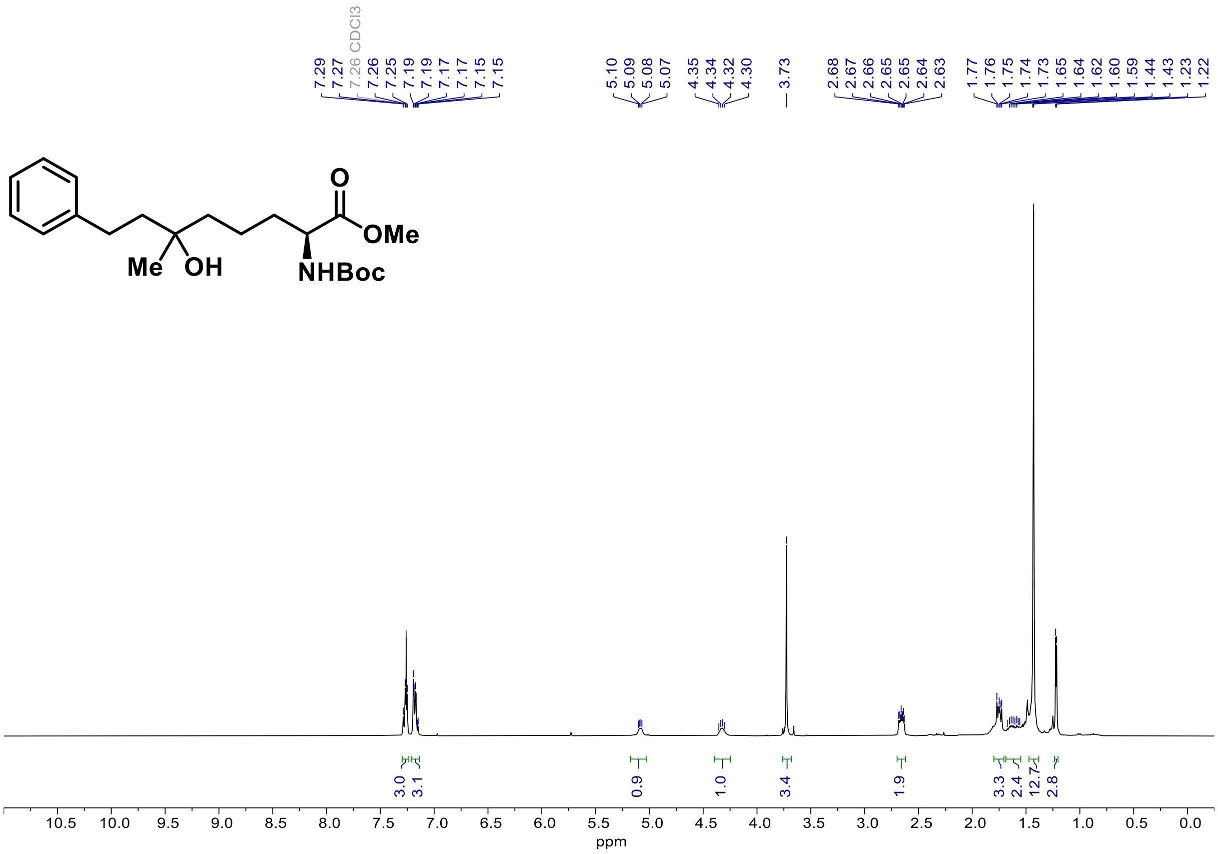

7 – ^{13}C NMR (101 MHz, CDCl$_3$)

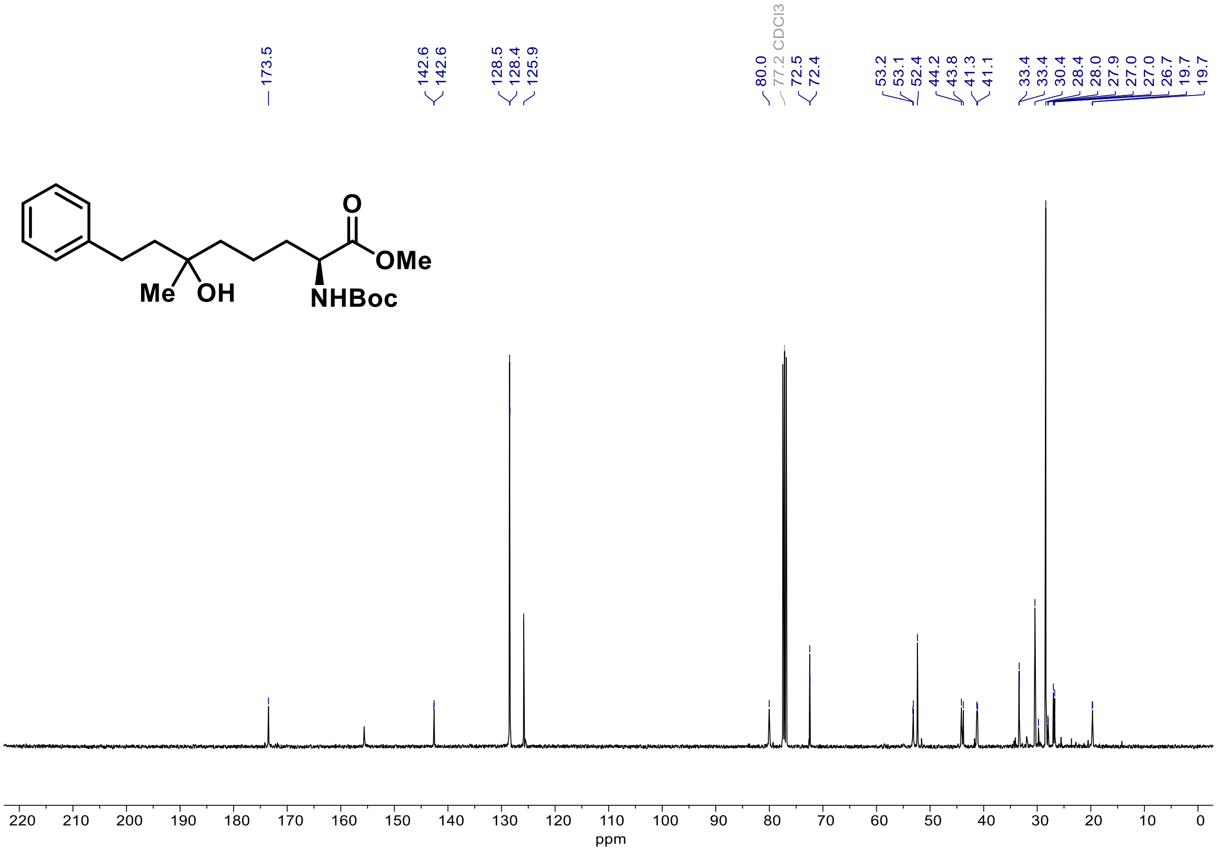

8 – ^{1}H NMR (400 MHz, $CDCl_3$)

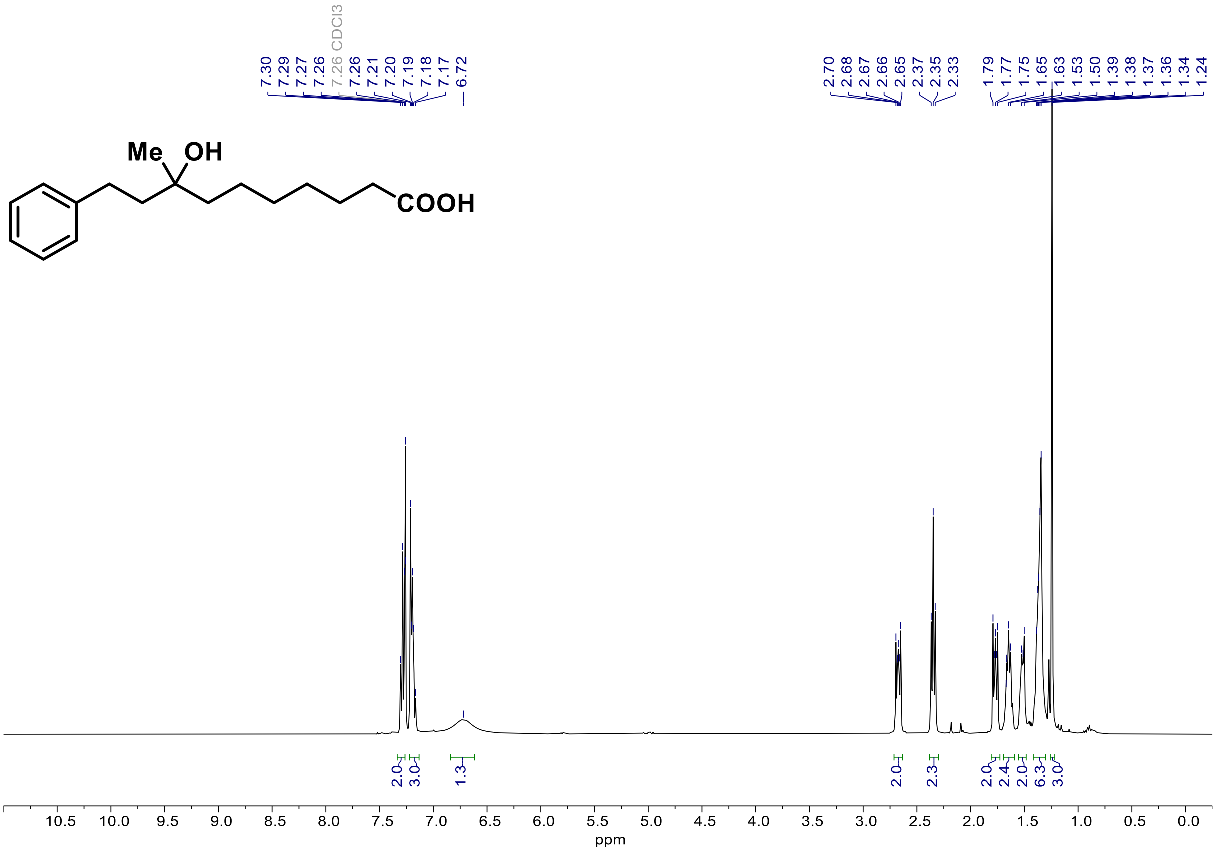

8 – ^{13}C NMR (101 MHz, $CDCl_3$)

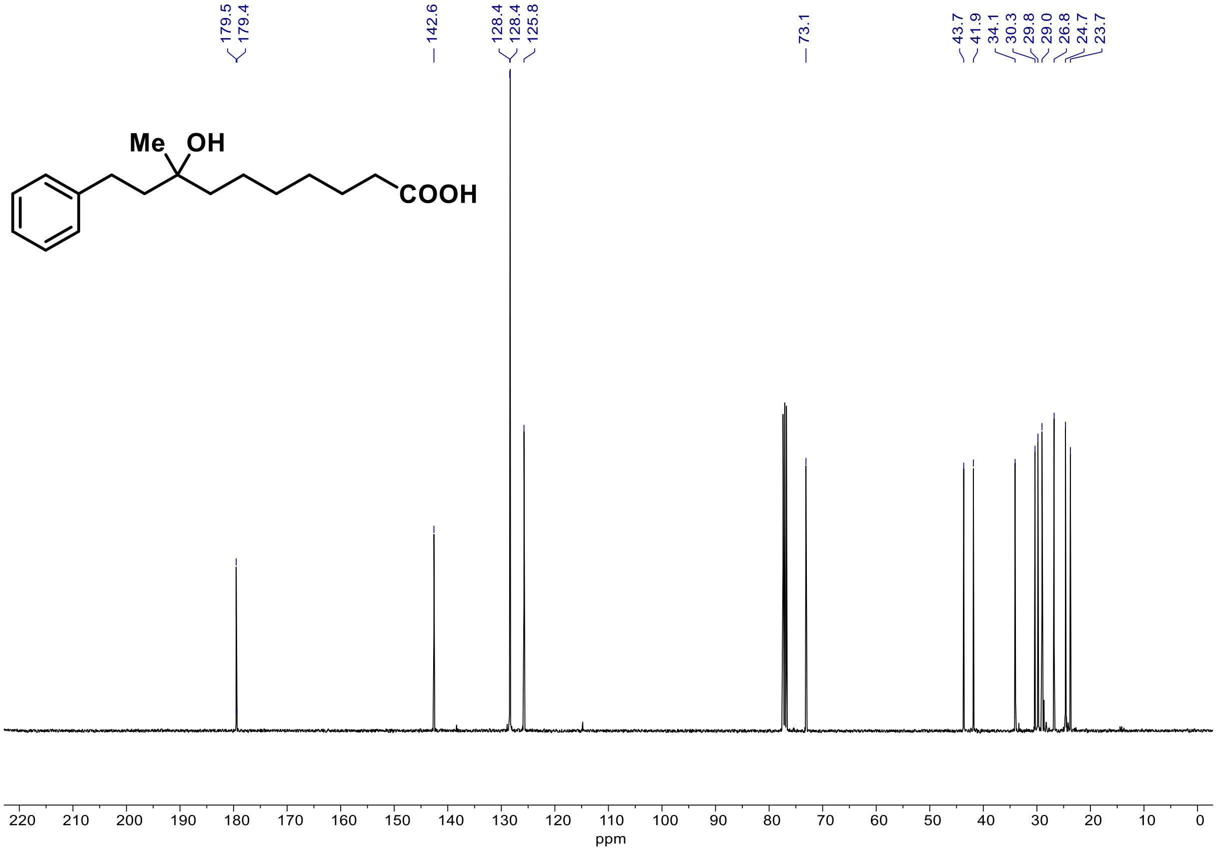

9 – ^{1}H NMR (400 MHz, $CDCl_3$)

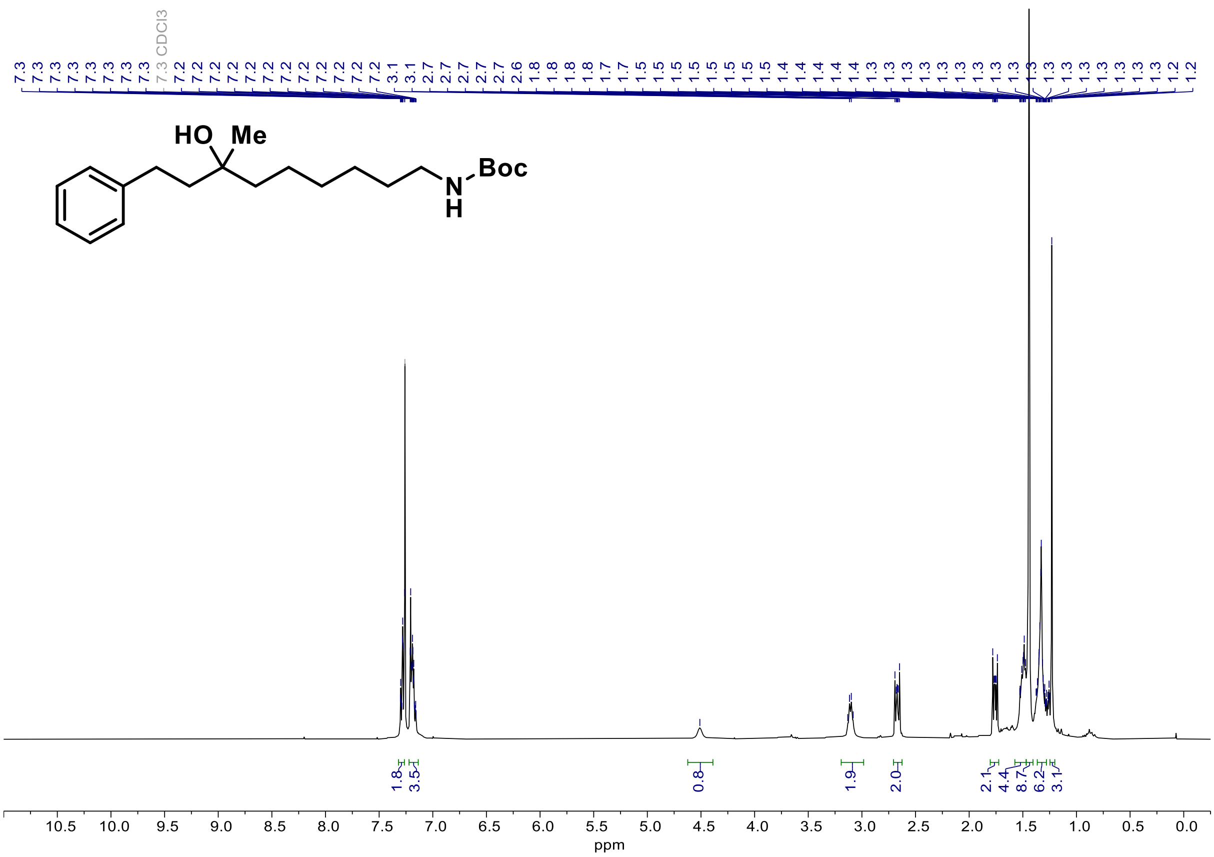

9 – ^{13}C NMR (101 MHz, $CDCl_3$)

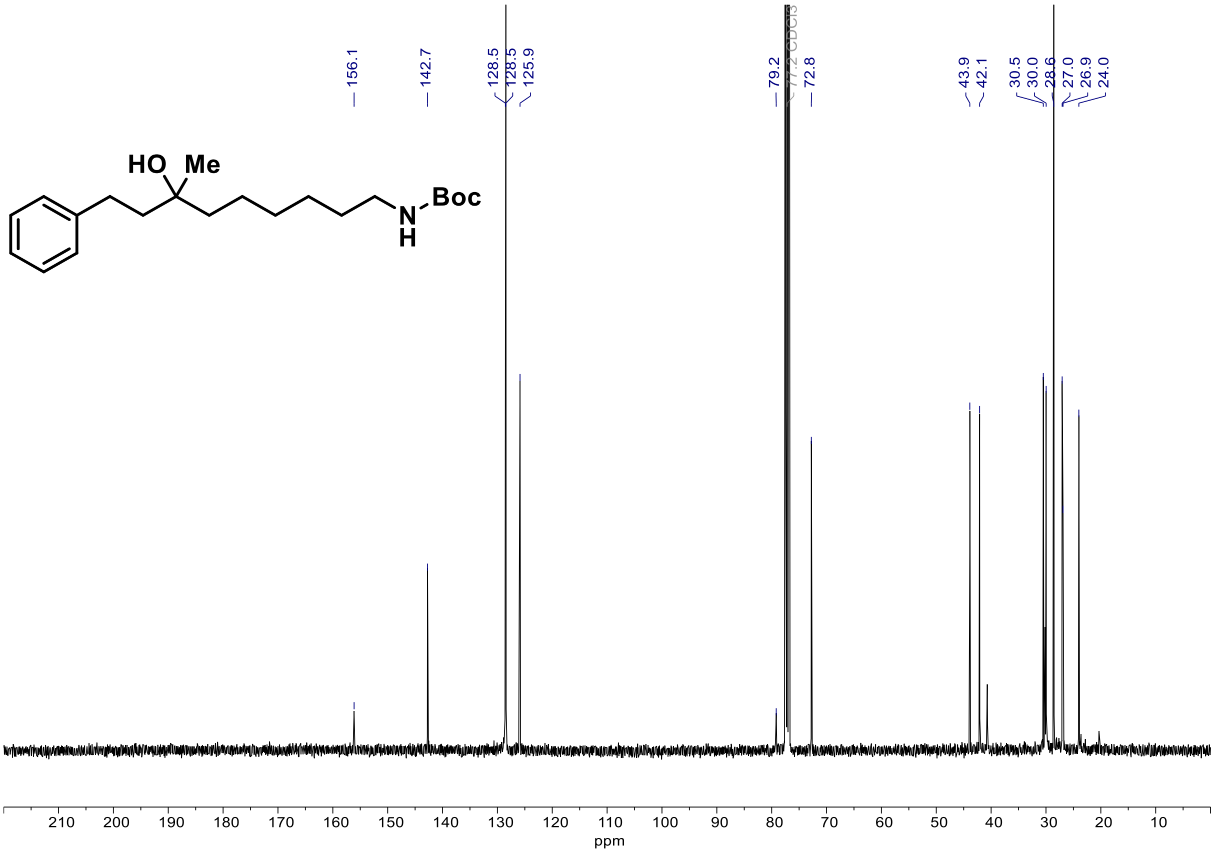

10 – ^{1}H NMR (400 MHz, $CDCl_3$)

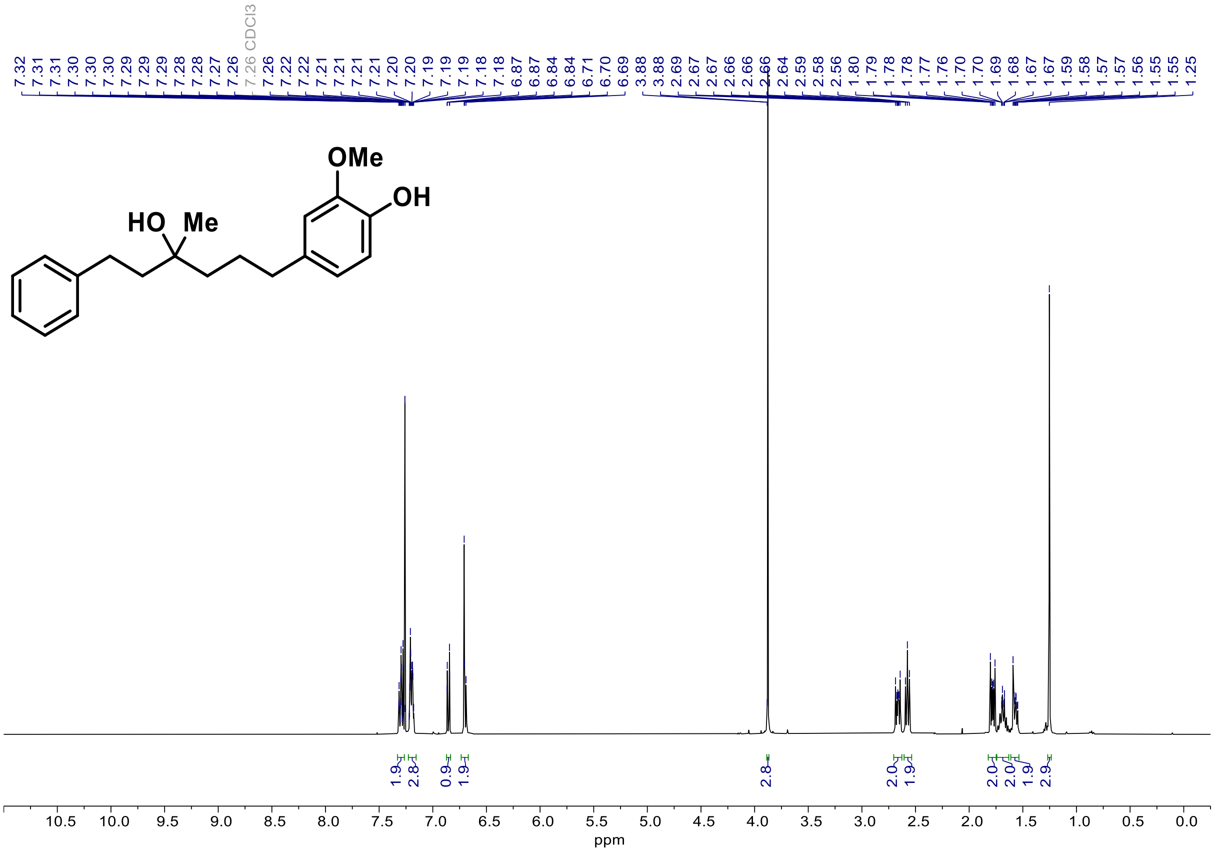

10 – ^{13}C NMR (101 MHz, $CDCl_3$)

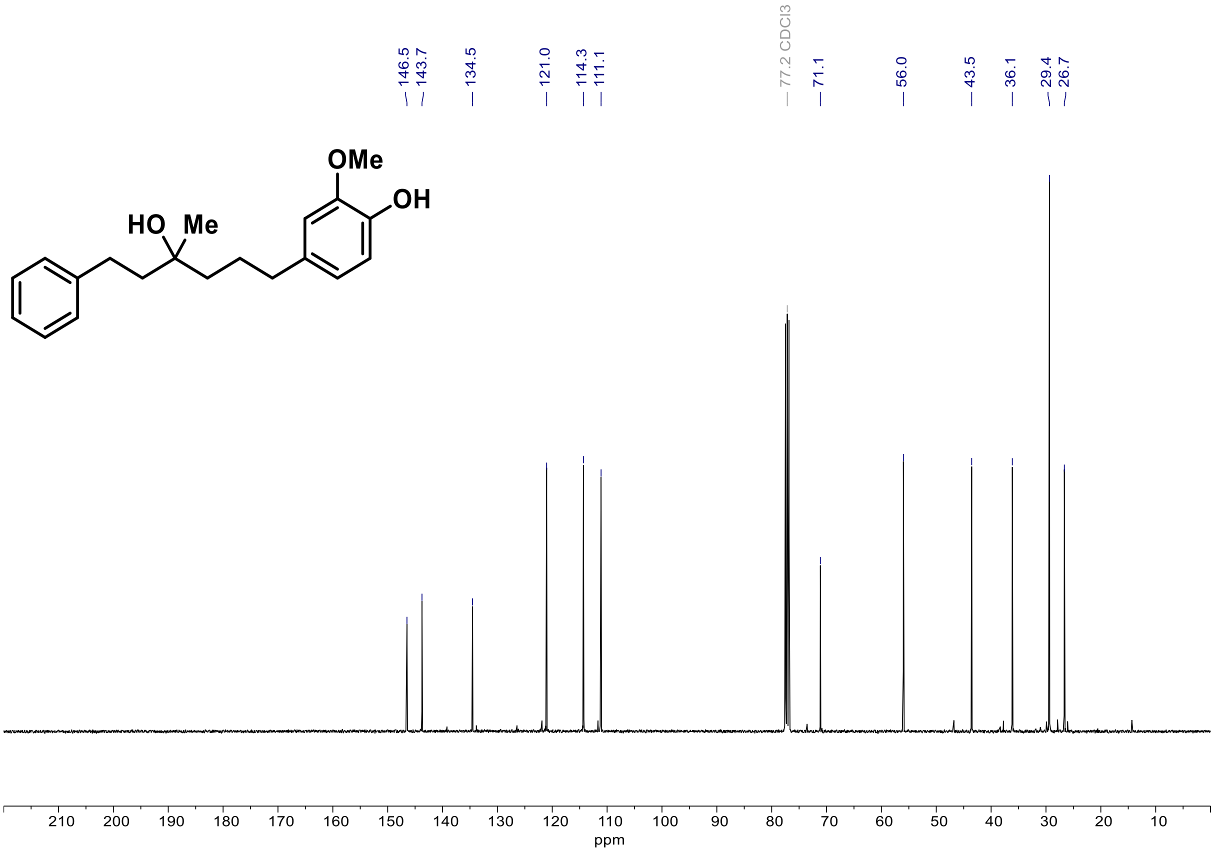

11 – ^{1}H NMR (400 MHz, $CDCl_3$)

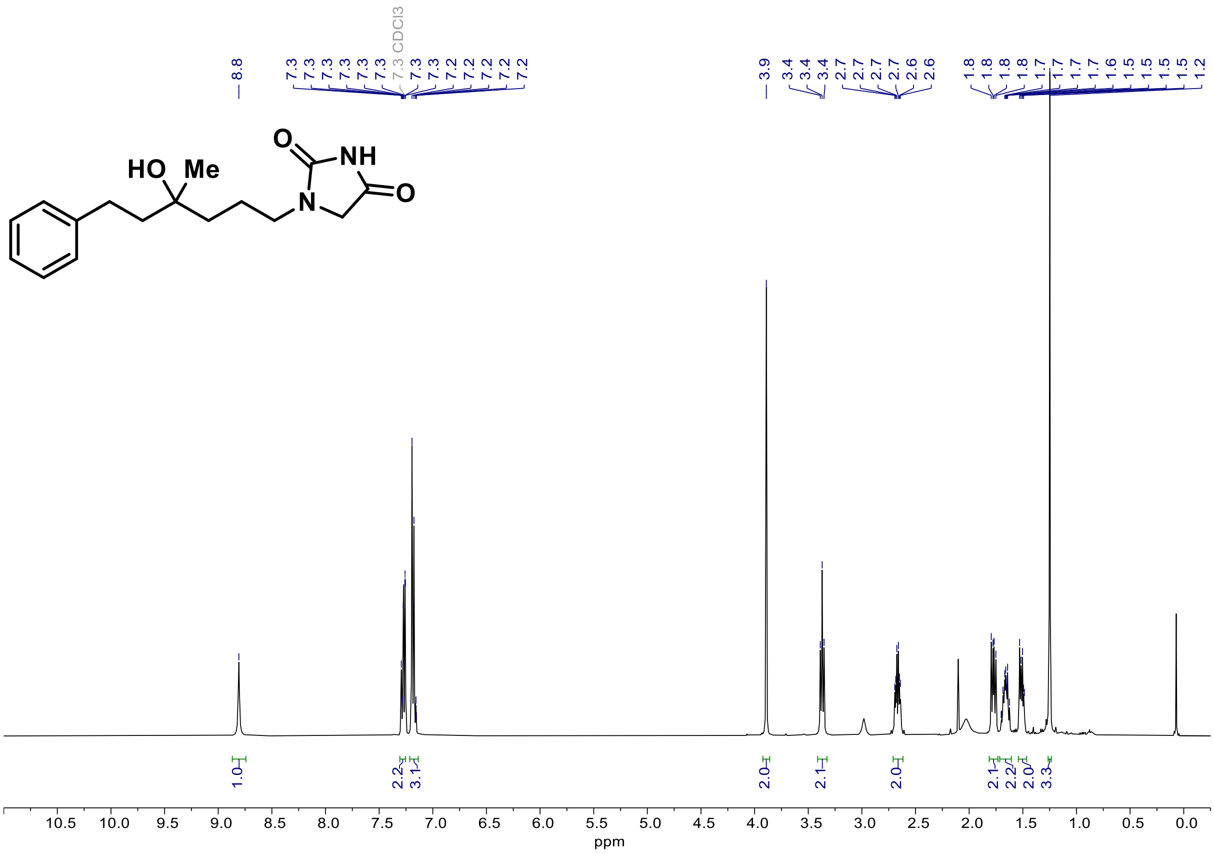

11 – ^{13}C NMR (101 MHz, $CDCl_3$)

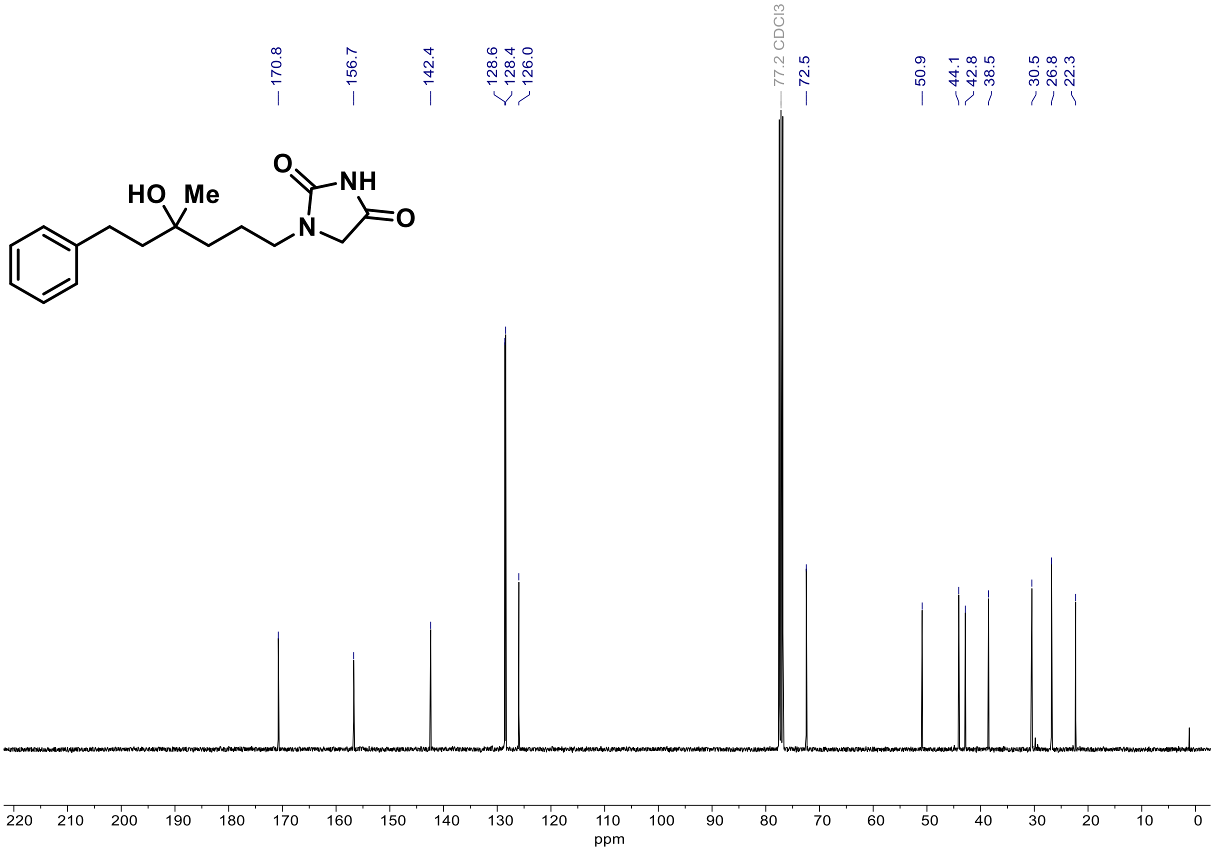

12 – ^{1}H NMR (400 MHz, $CDCl_3$)

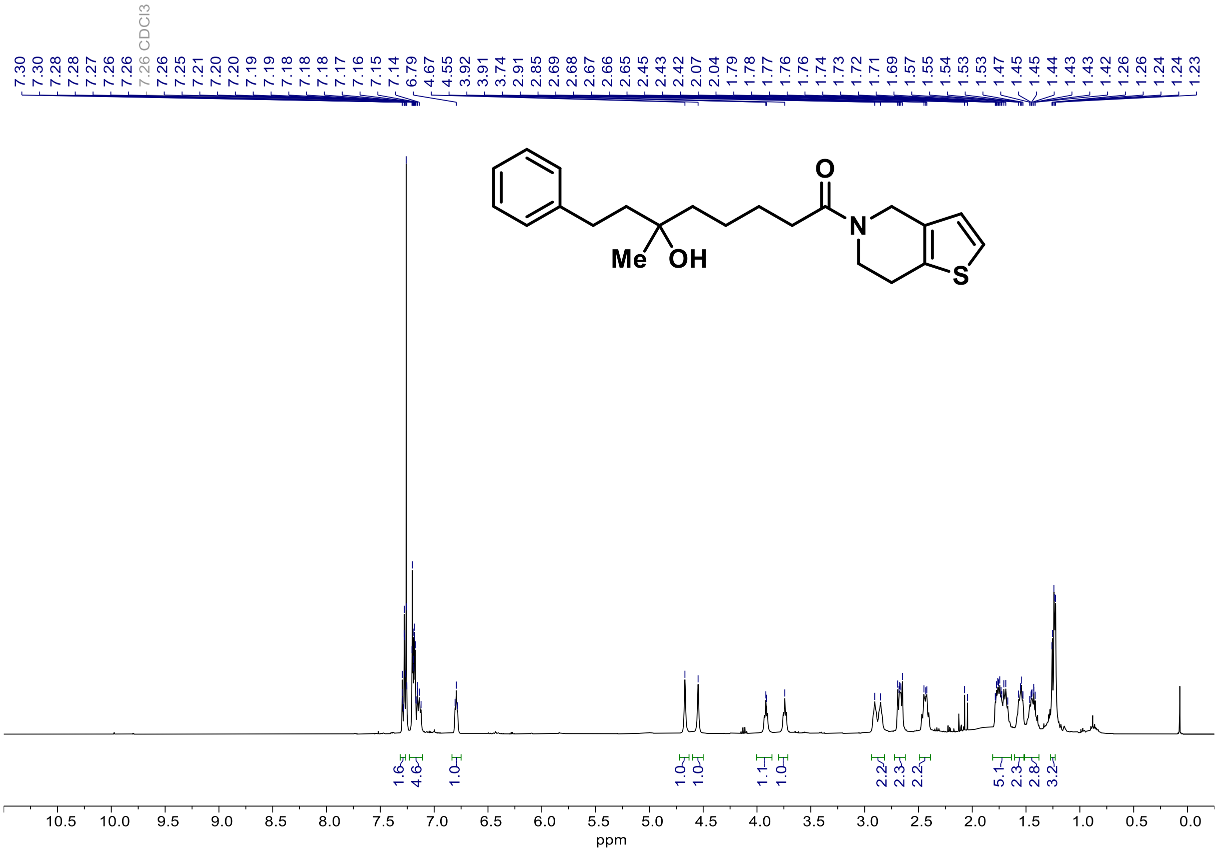

12 – ^{13}C NMR (101 MHz, $CDCl_3$)

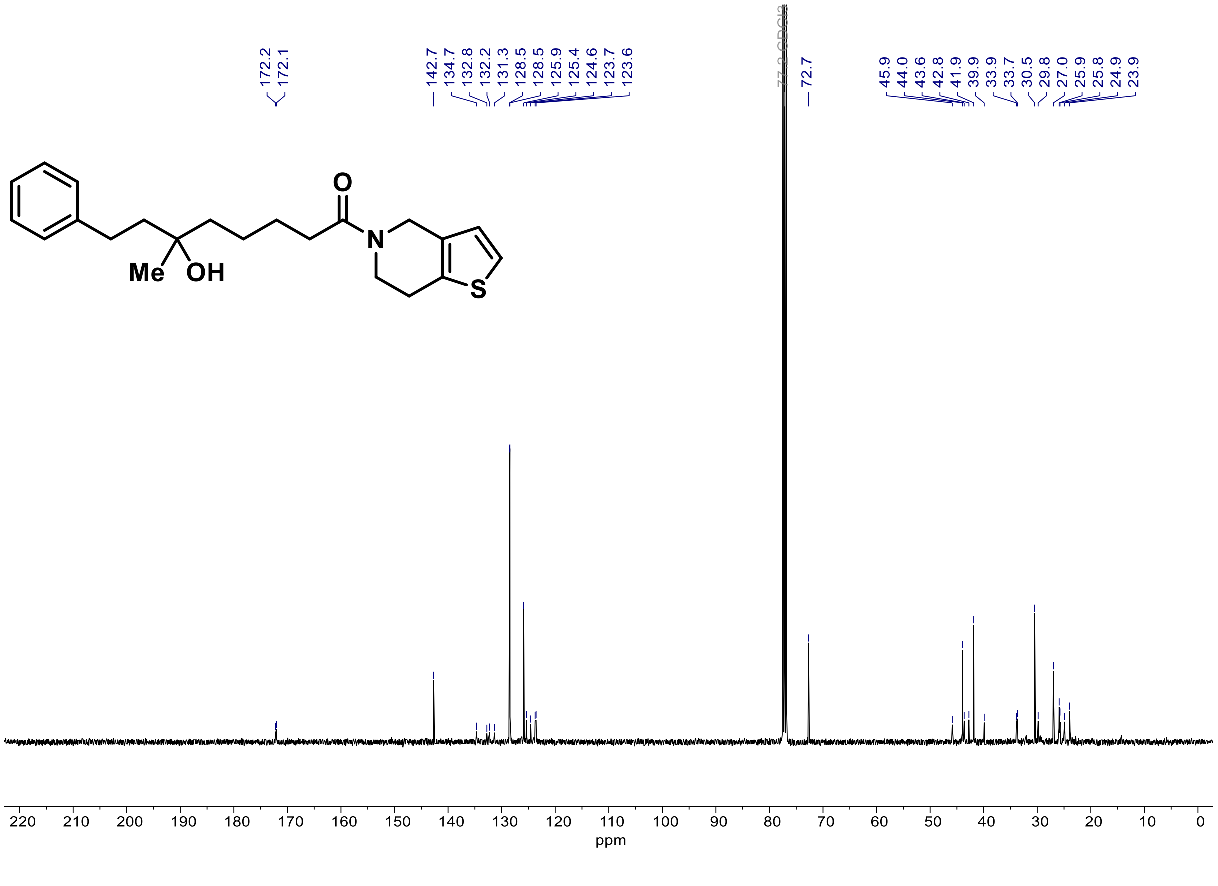

13 – ^{1}H NMR (400 MHz, $CDCl_3$)

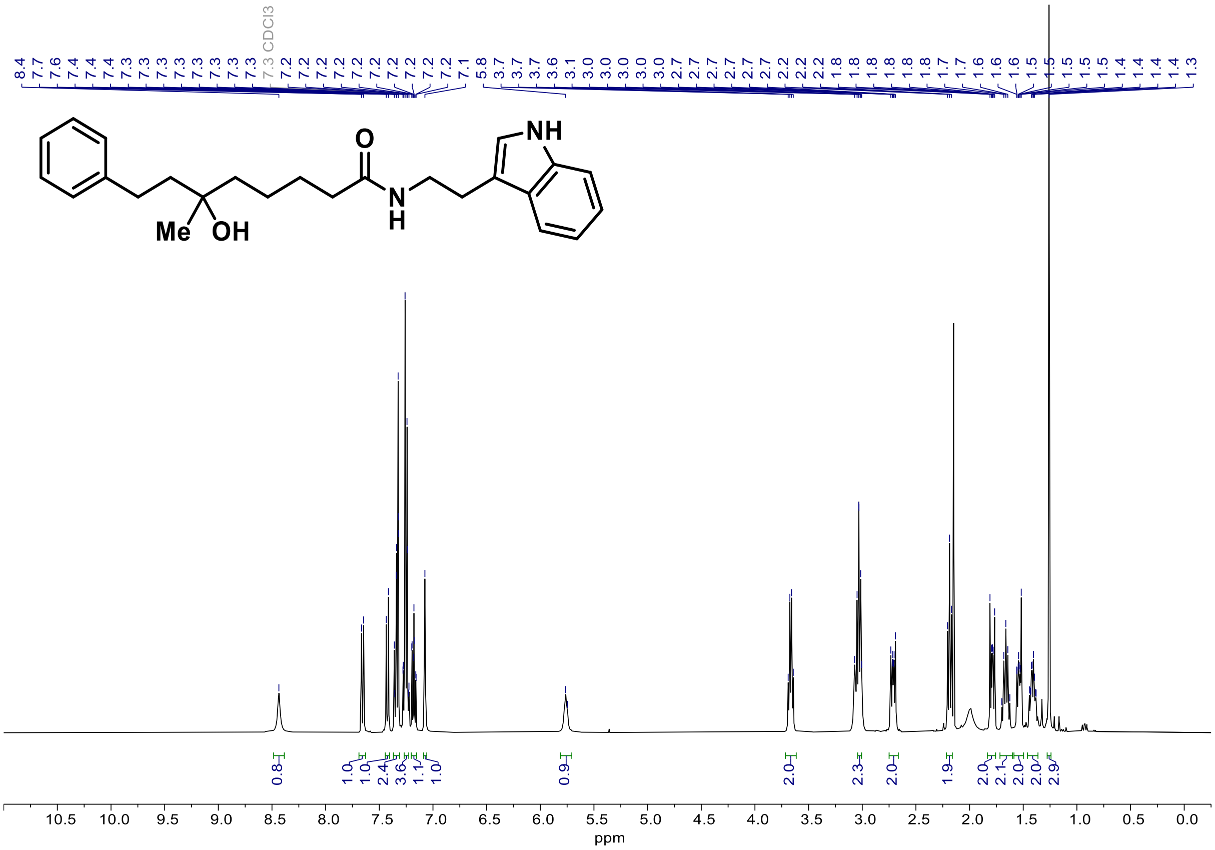

13 – ^{13}C NMR (101 MHz, $CDCl_3$)

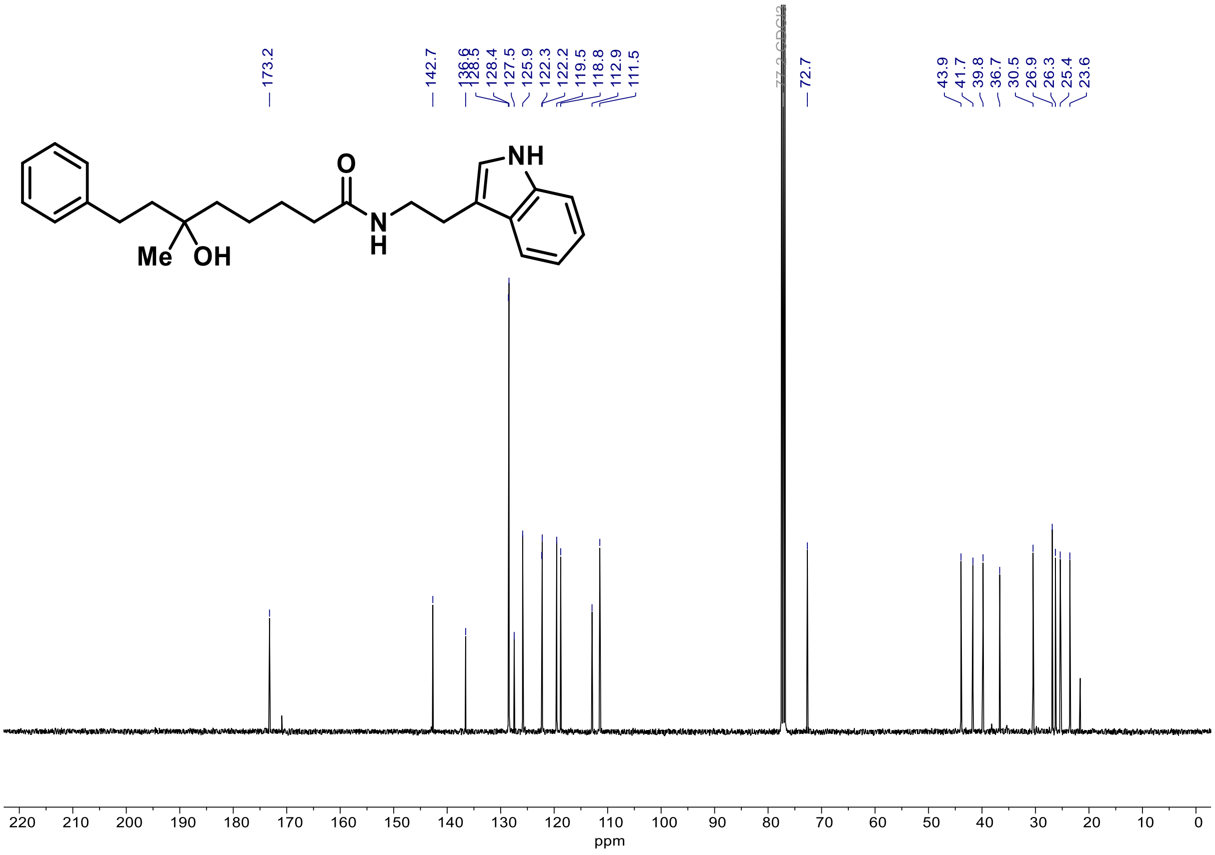

14 – ^{1}H NMR (400 MHz, $CDCl_3$)

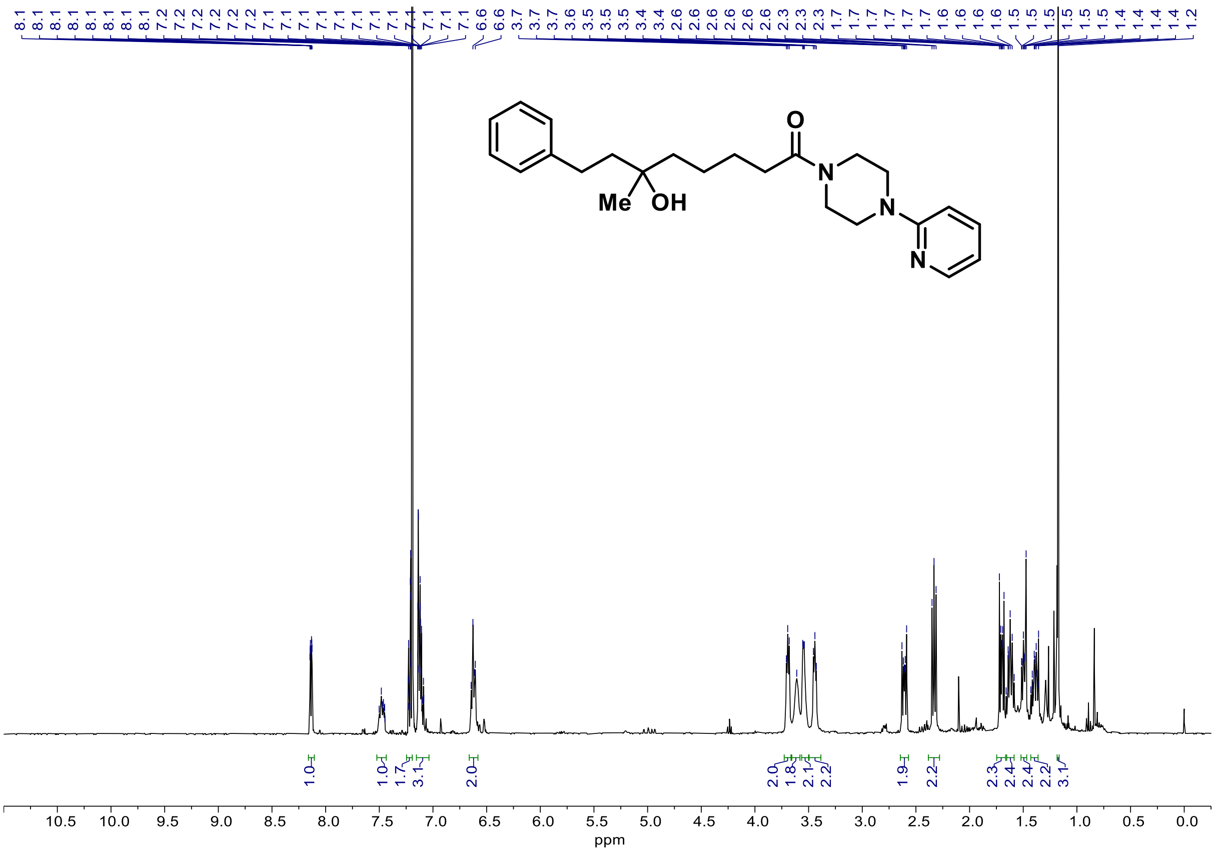

14 – ^{13}C NMR (101 MHz, $CDCl_3$)

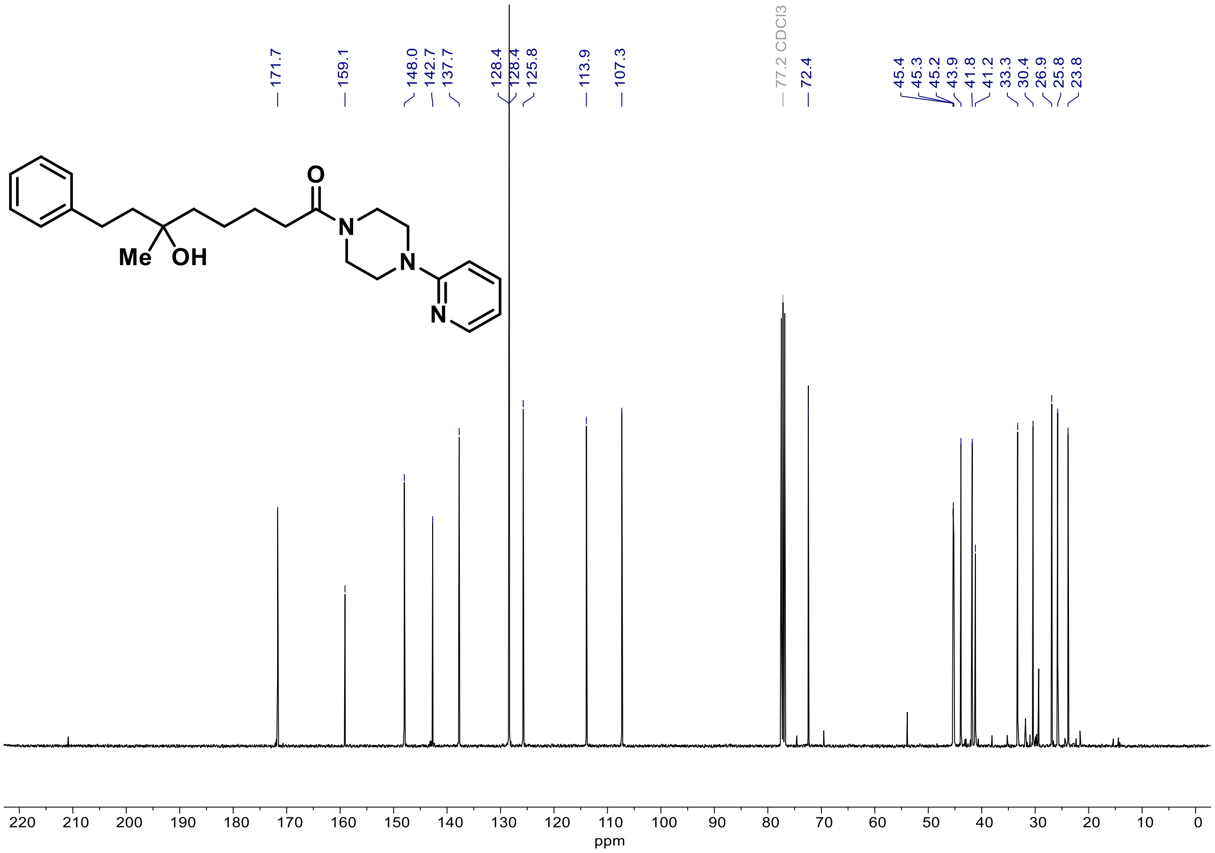

15 – ^{1}H NMR (400 MHz, $CDCl_3$)

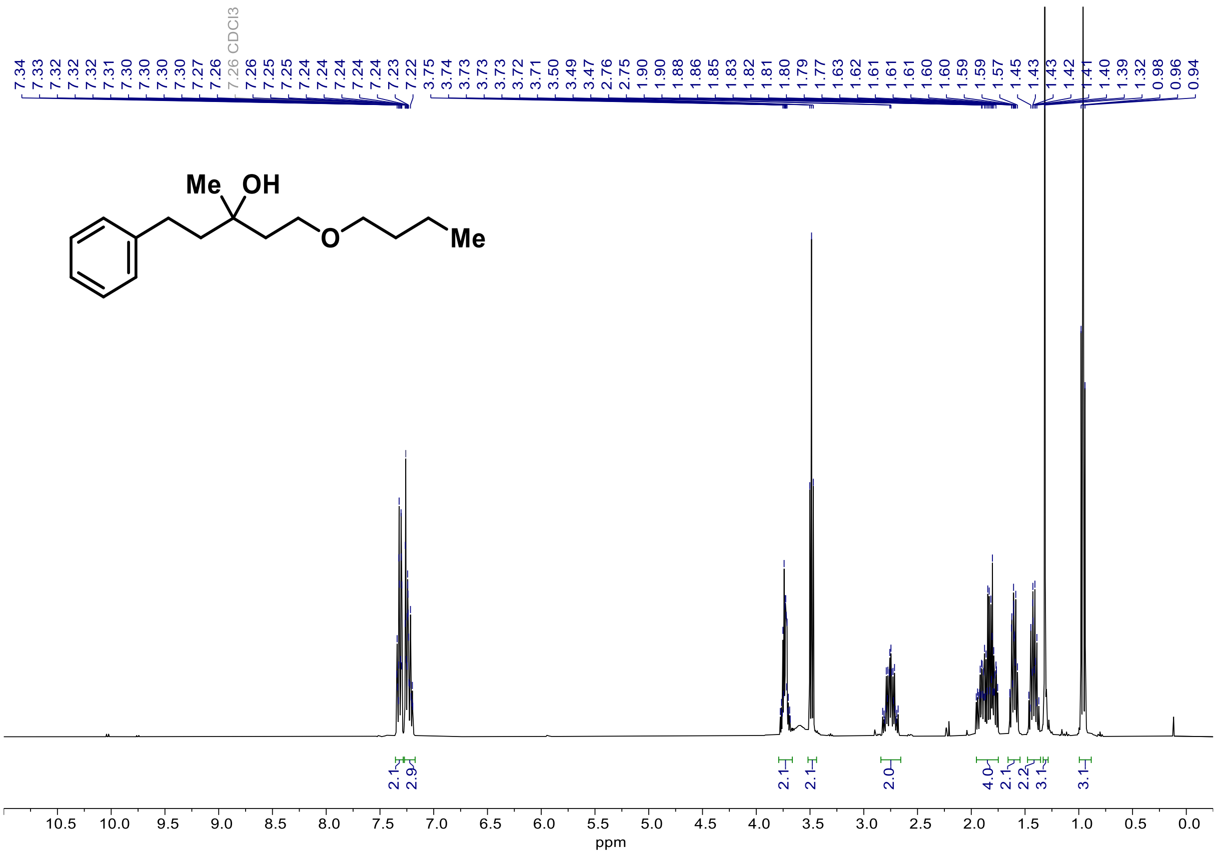

15 – ^{13}C NMR (101 MHz, $CDCl_3$)

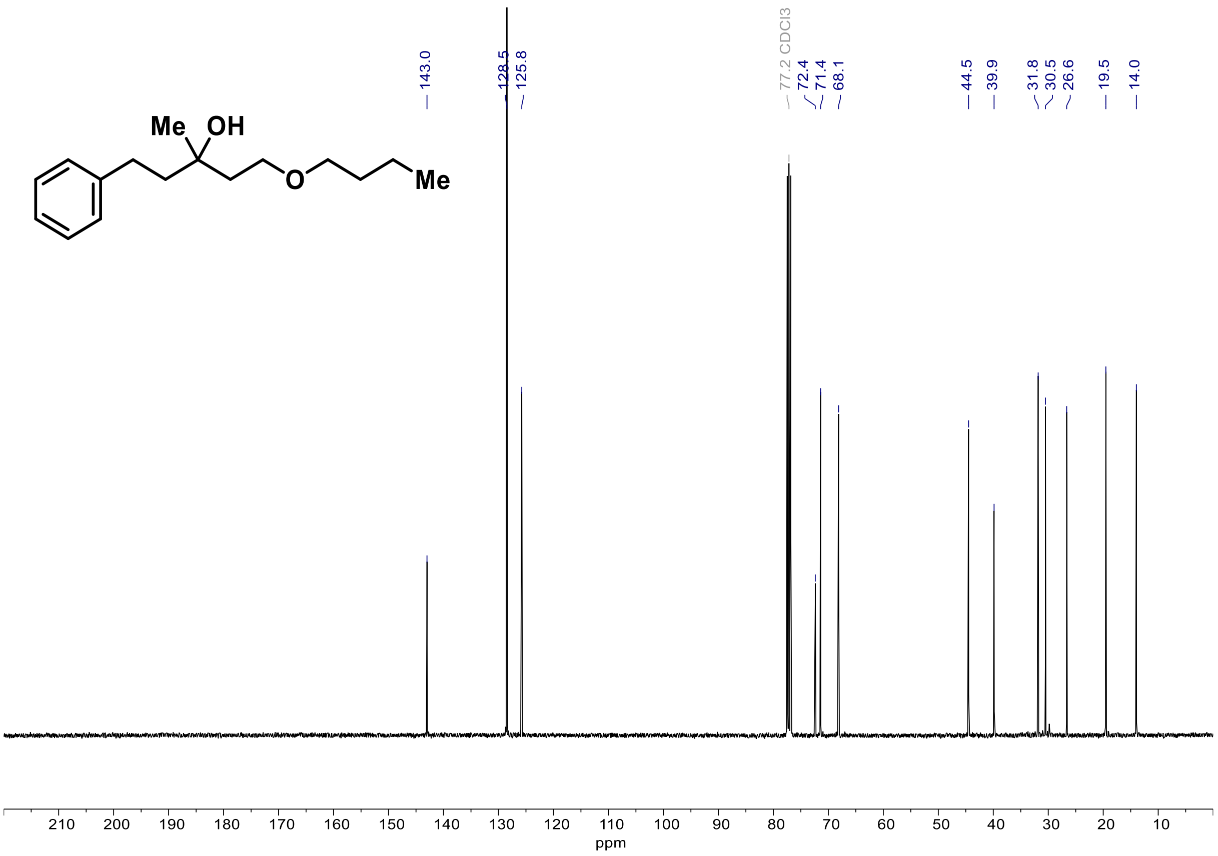

16 – ^{1}H NMR (400 MHz, $CDCl_3$)

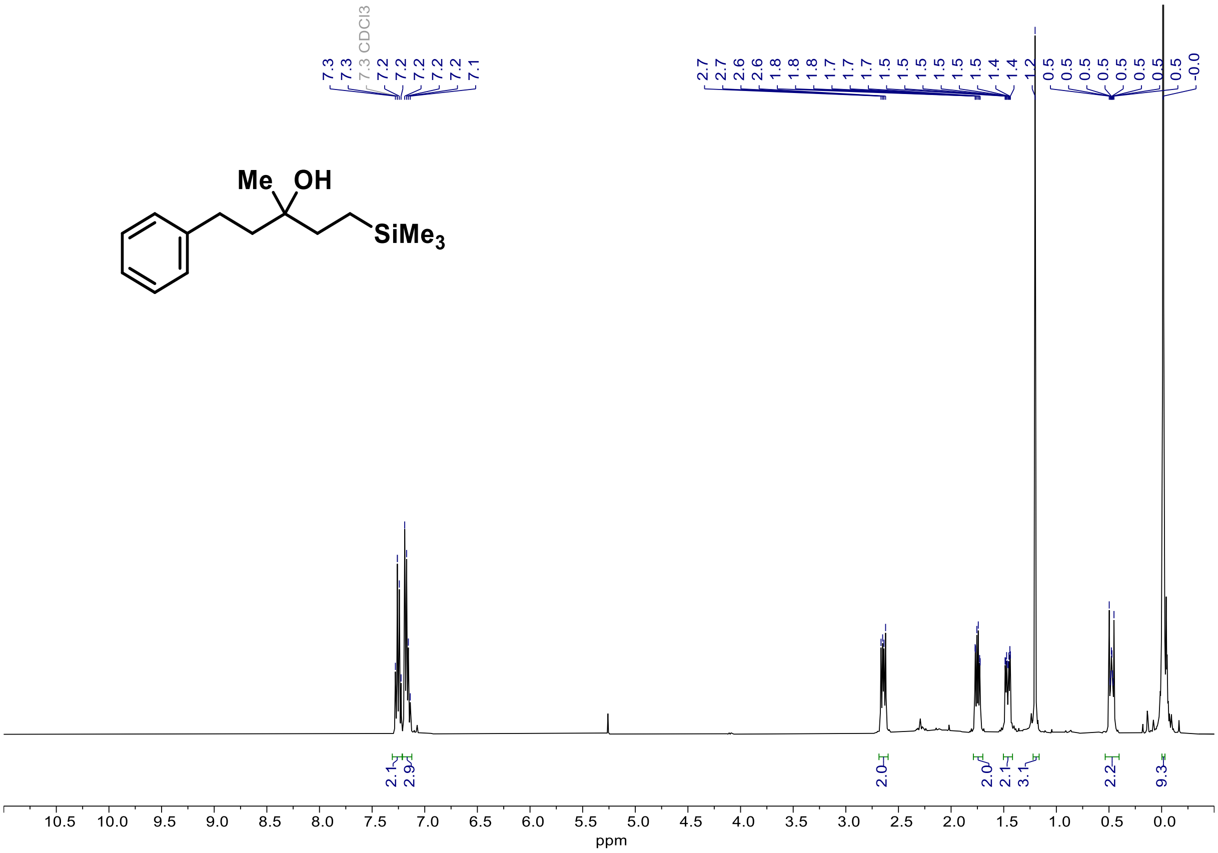

16 – ^{13}C NMR (101 MHz, $CDCl_3$)

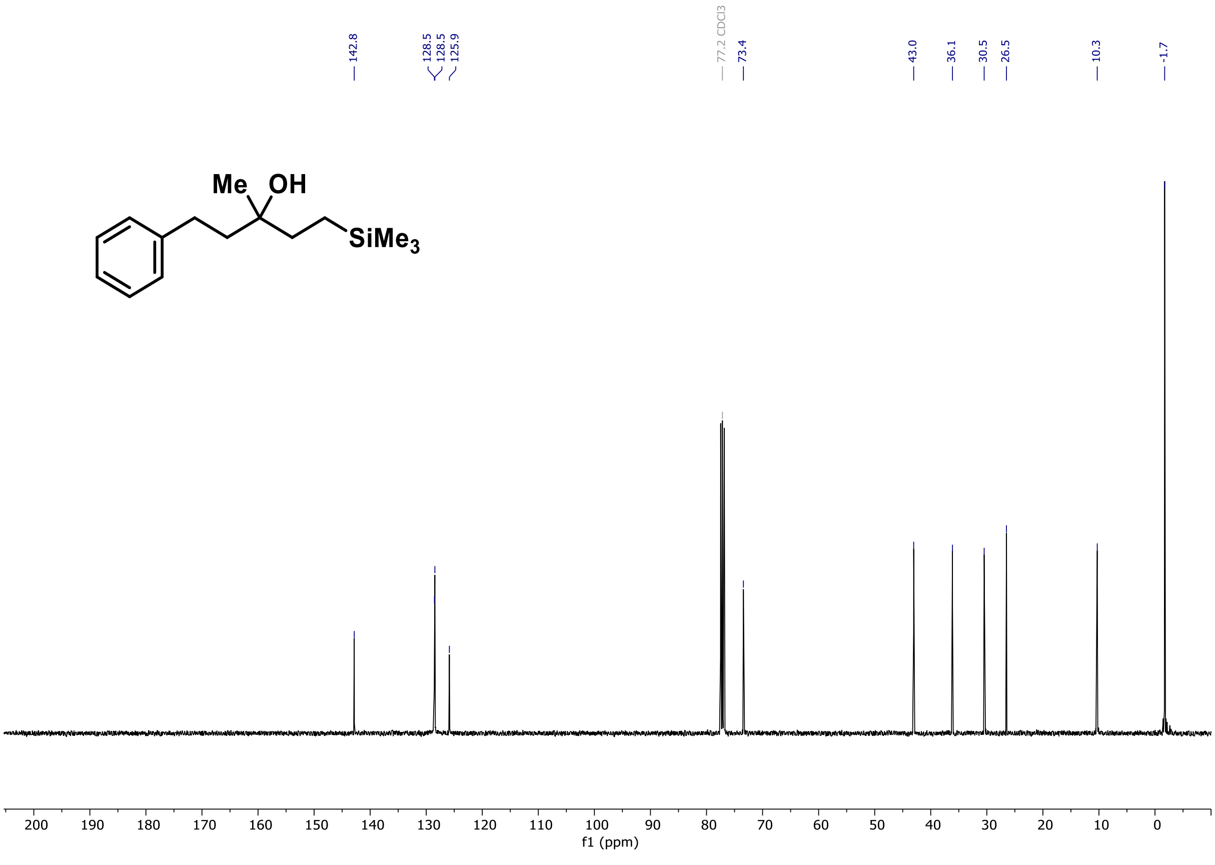

17 – ^{1}H NMR (400 MHz, $CDCl_3$)

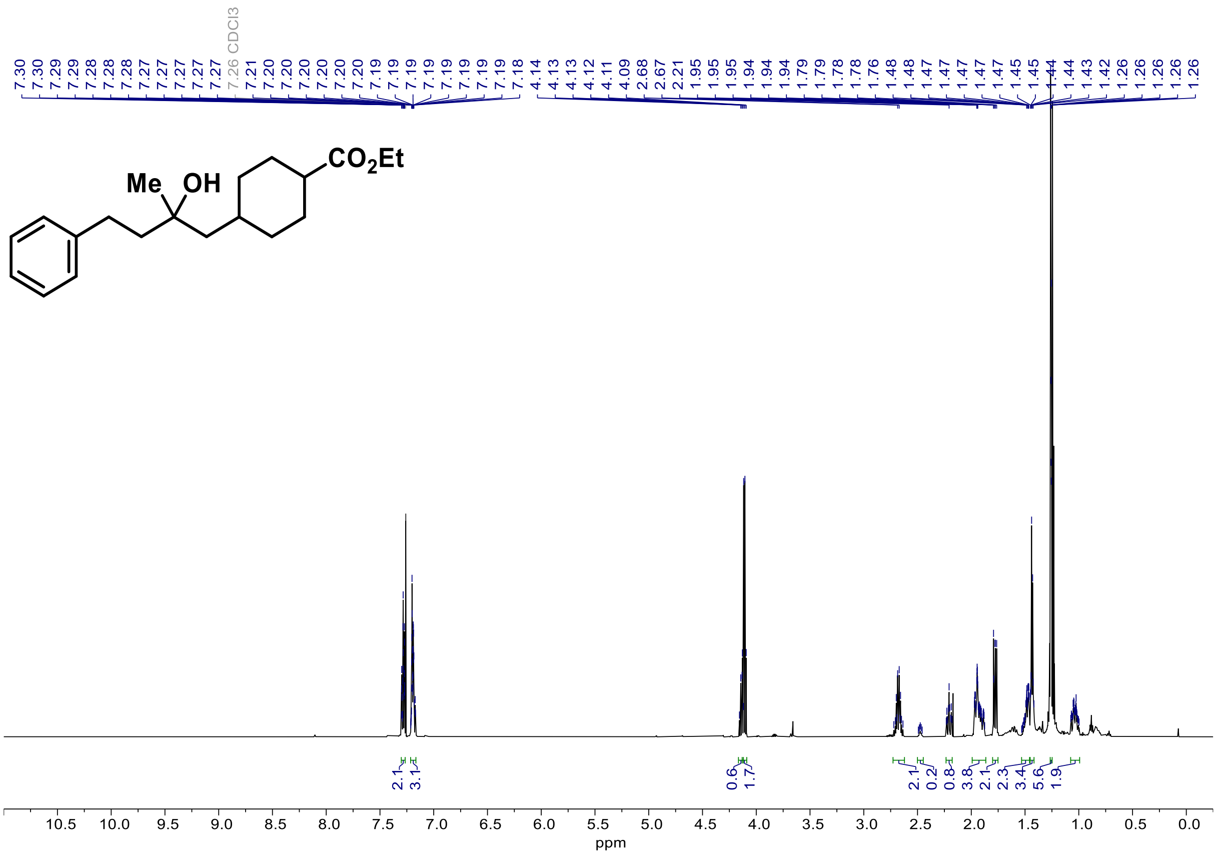

17 – ^{13}C NMR (101 MHz, $CDCl_3$)

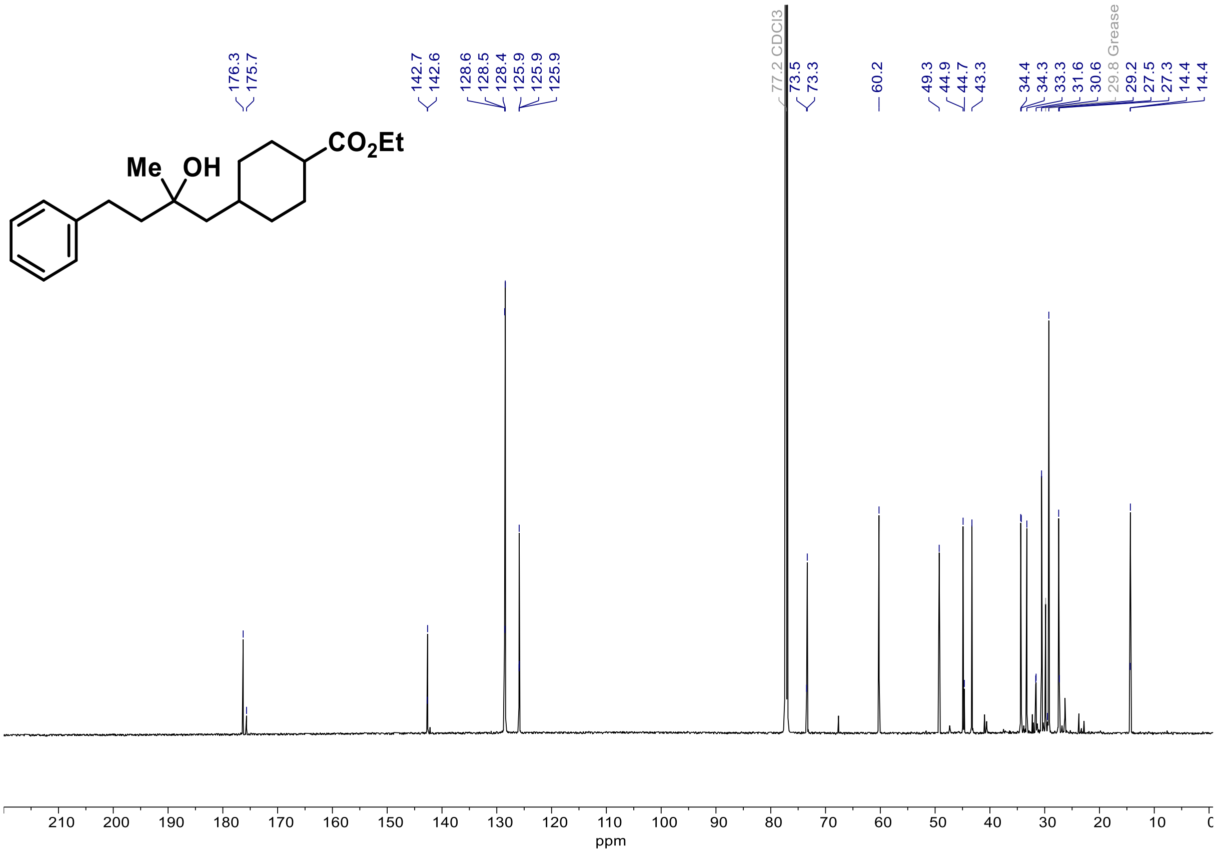

18 – ^{1}H NMR (400 MHz, $CDCl_3$)

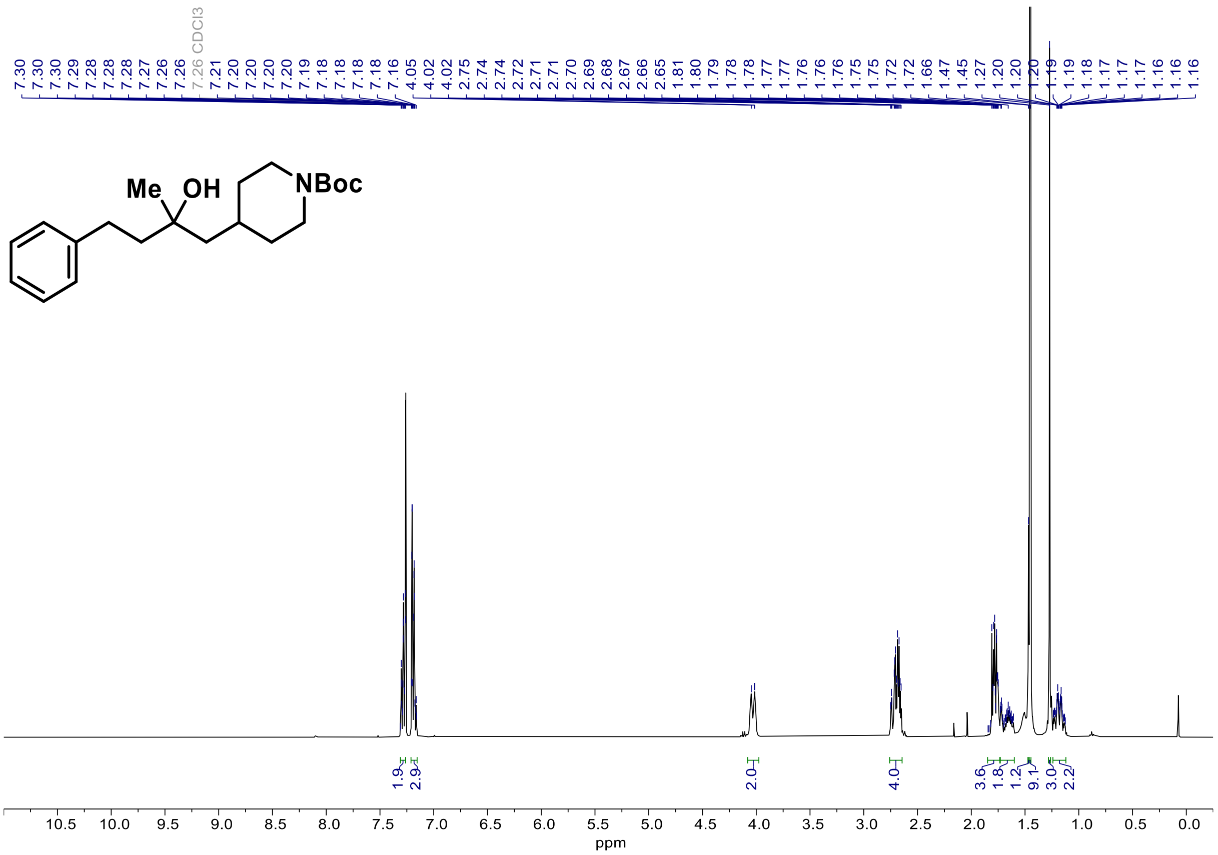

18 – ^{13}C NMR (101 MHz, $CDCl_3$)

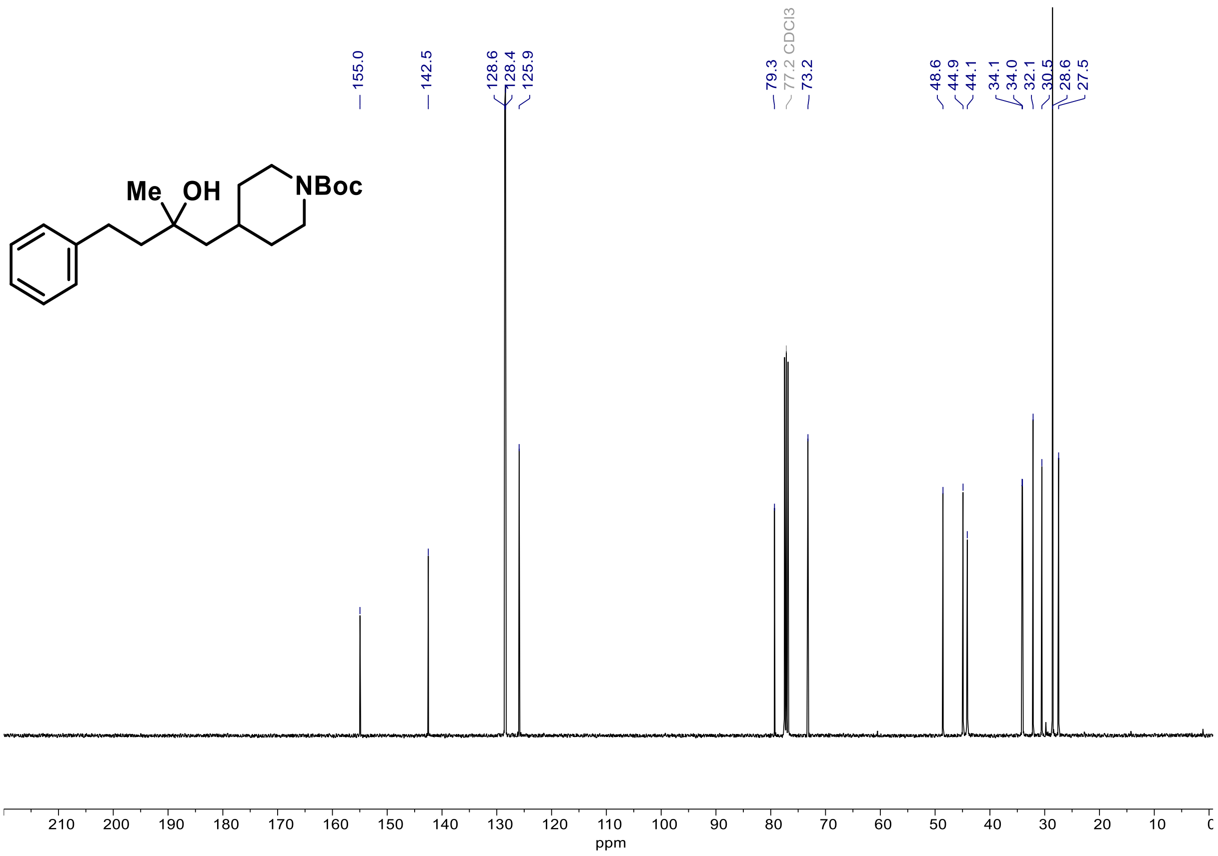

19 – ^{1}H NMR (400 MHz, $CDCl_3$)

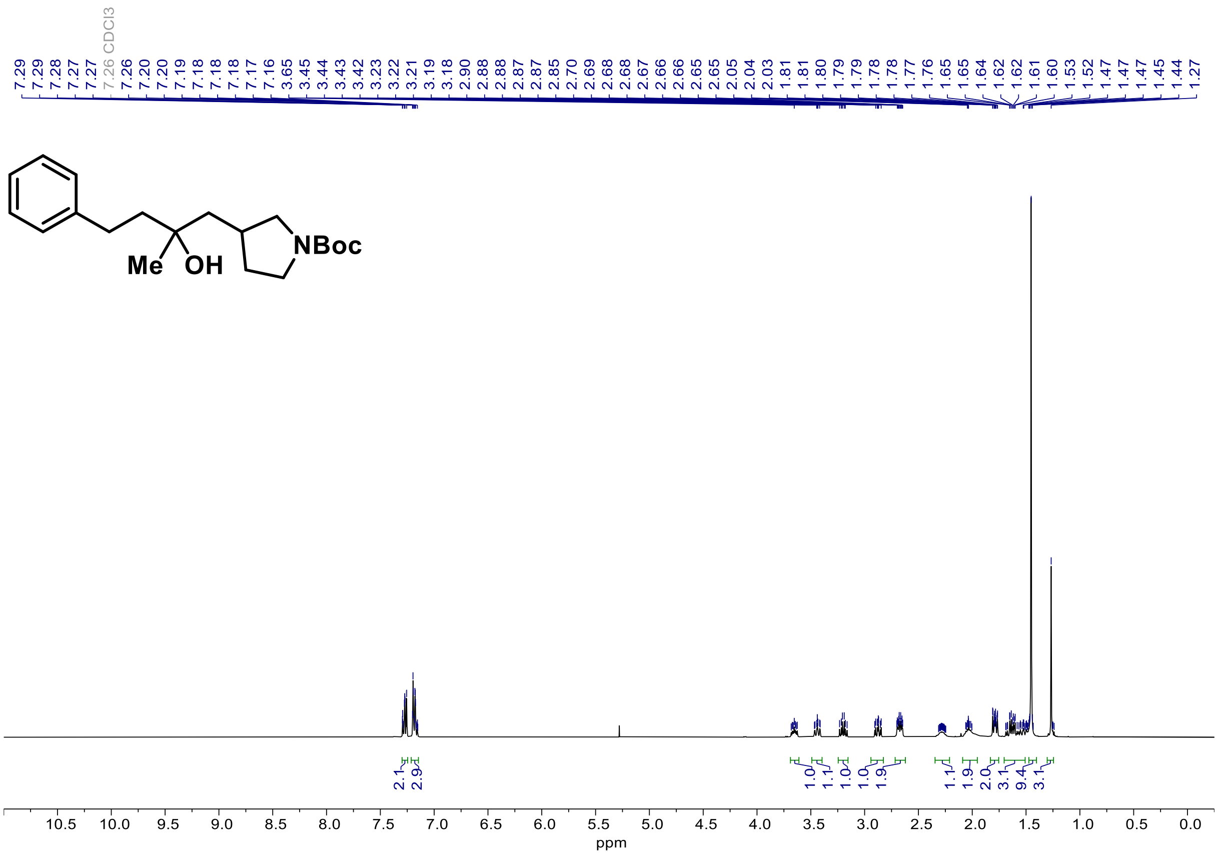

19 – ^{13}C NMR (101 MHz, $CDCl_3$)

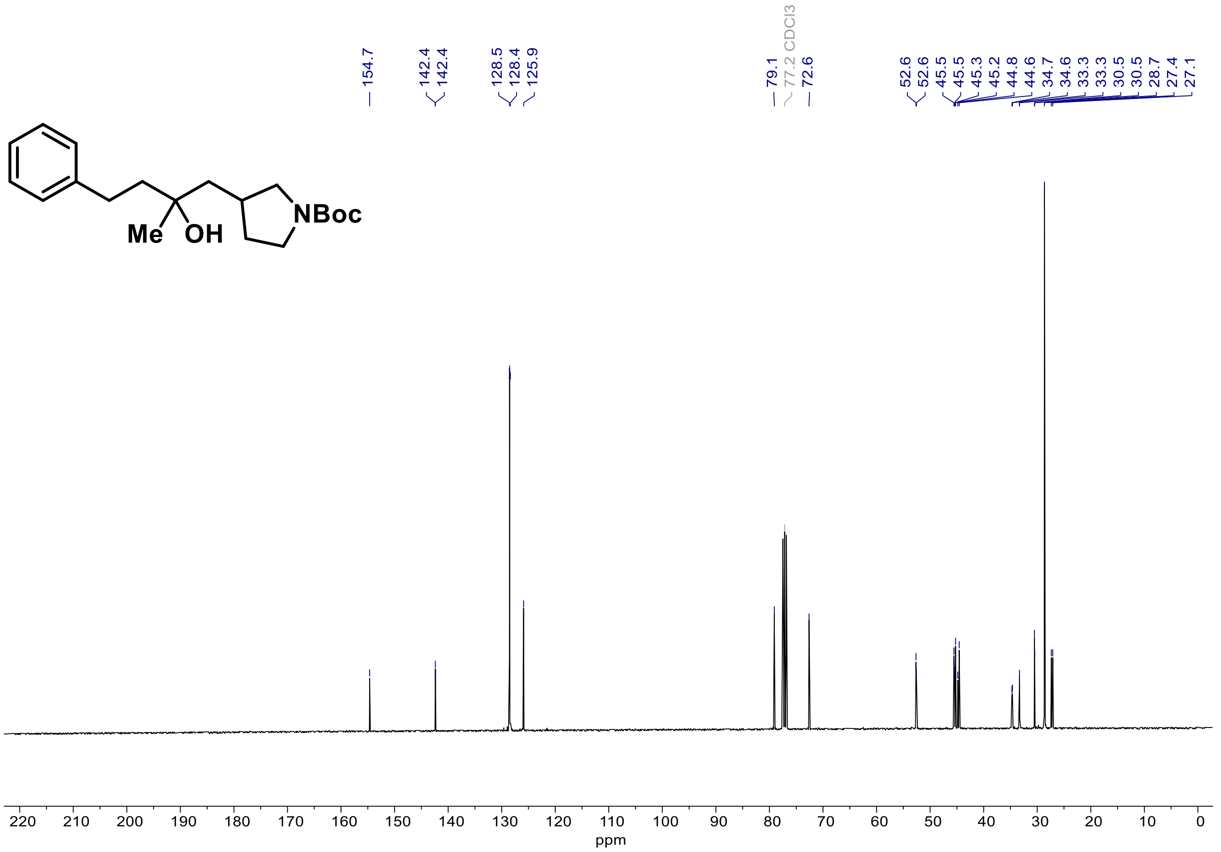

20 – ^{1}H NMR (400 MHz, $CDCl_3$)

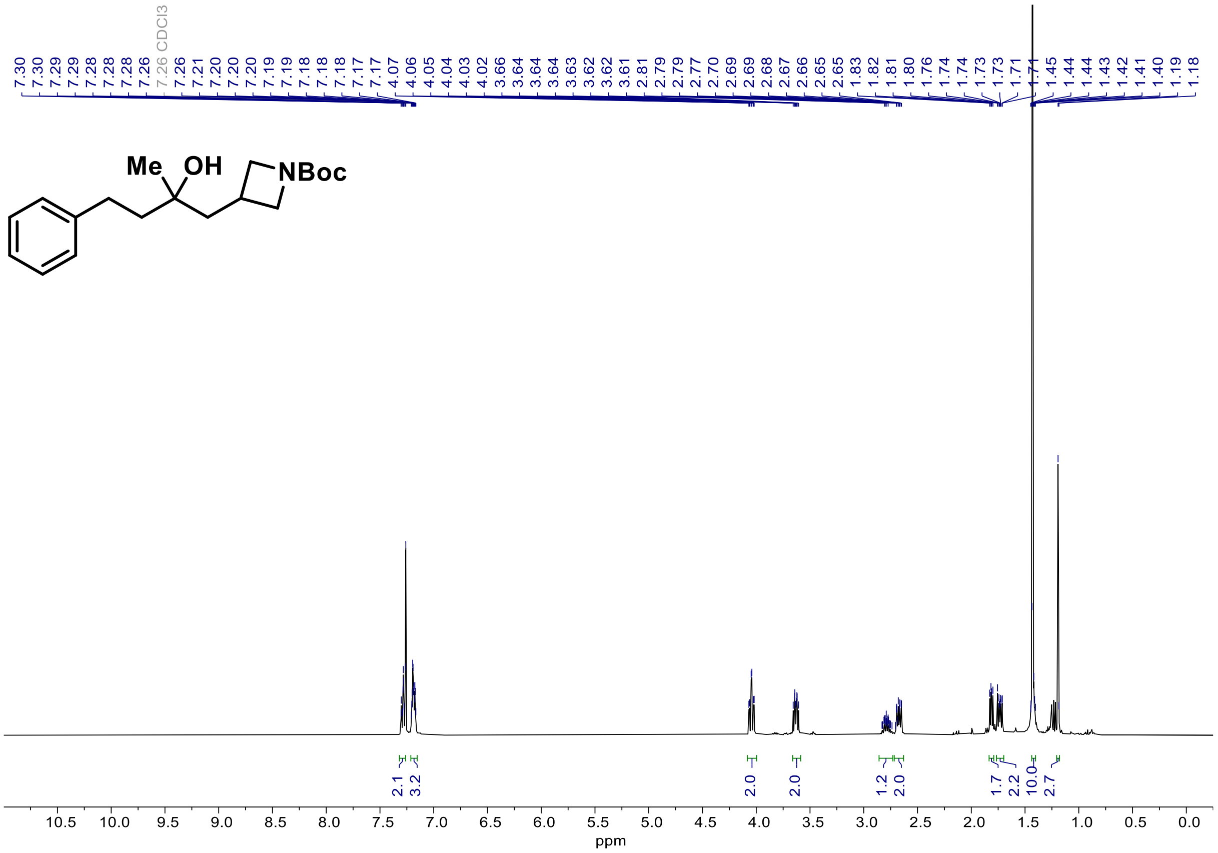

20 – ^{13}C NMR (101 MHz, $CDCl_3$)

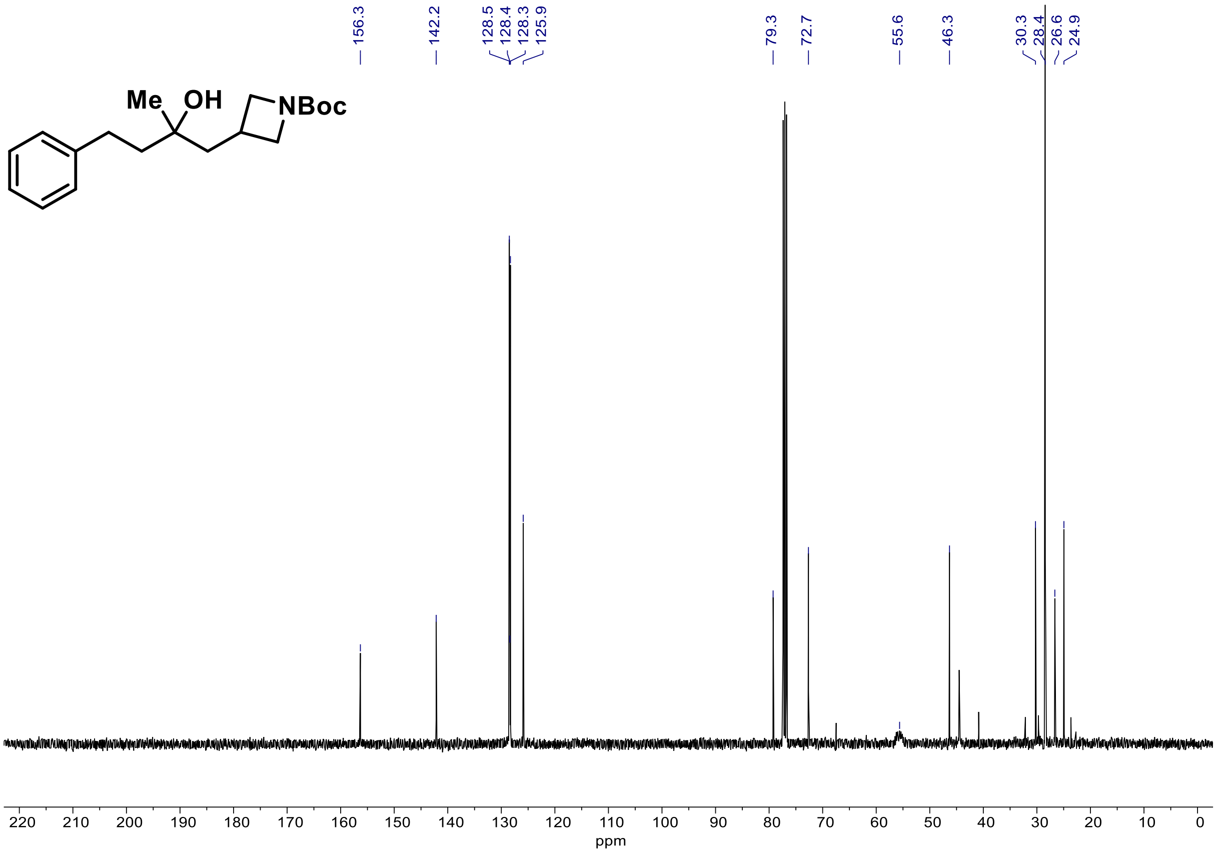

21 – ^{1}H NMR (400 MHz, $CDCl_3$)

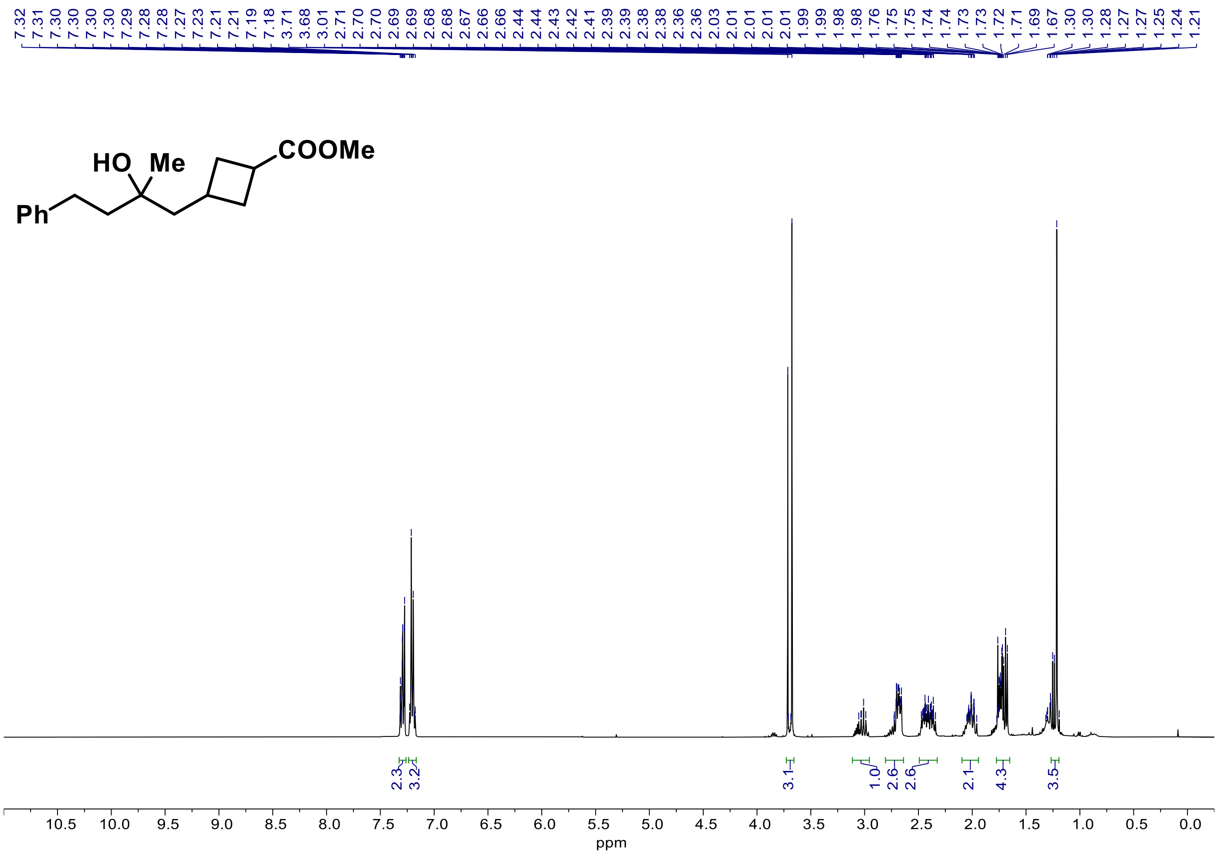

21 – ^{13}C NMR (101 MHz, $CDCl_3$)

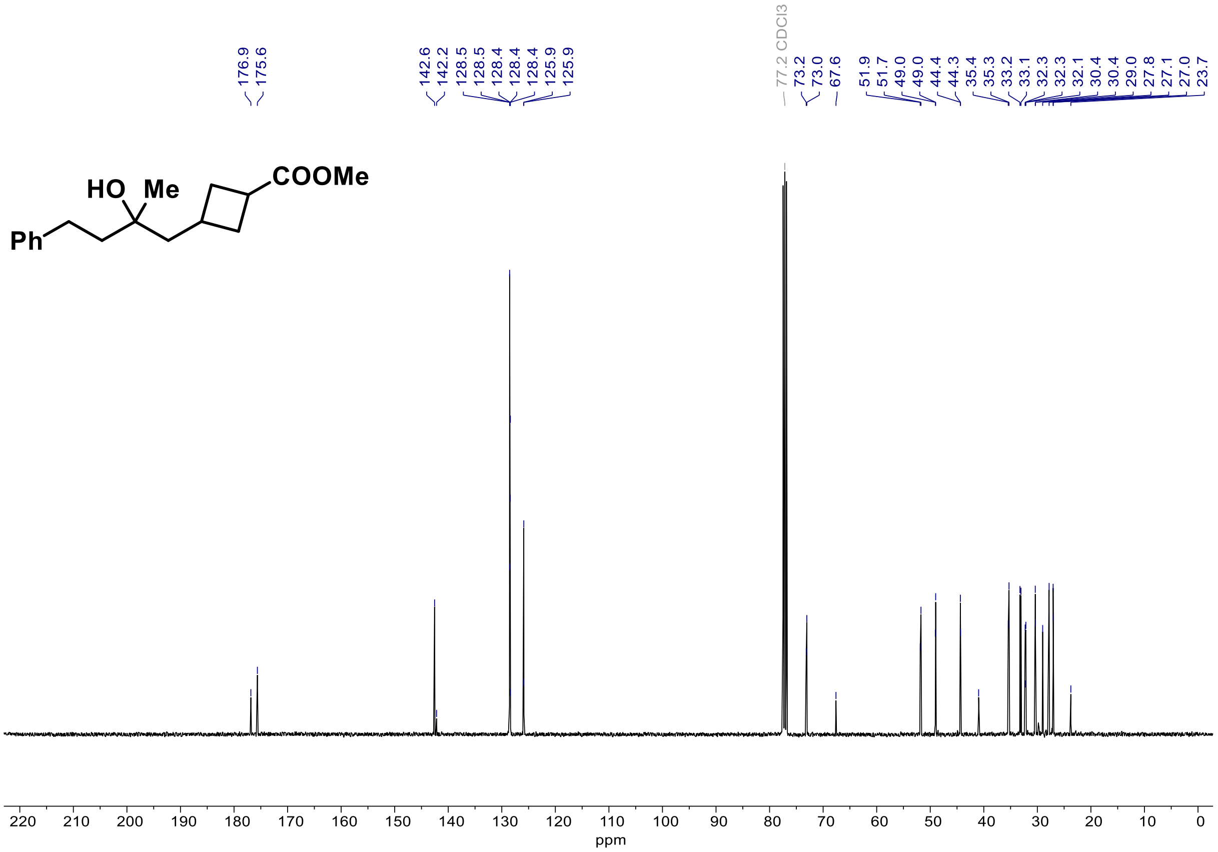

22 – ^{1}H NMR (400 MHz, CDCl$_3$)

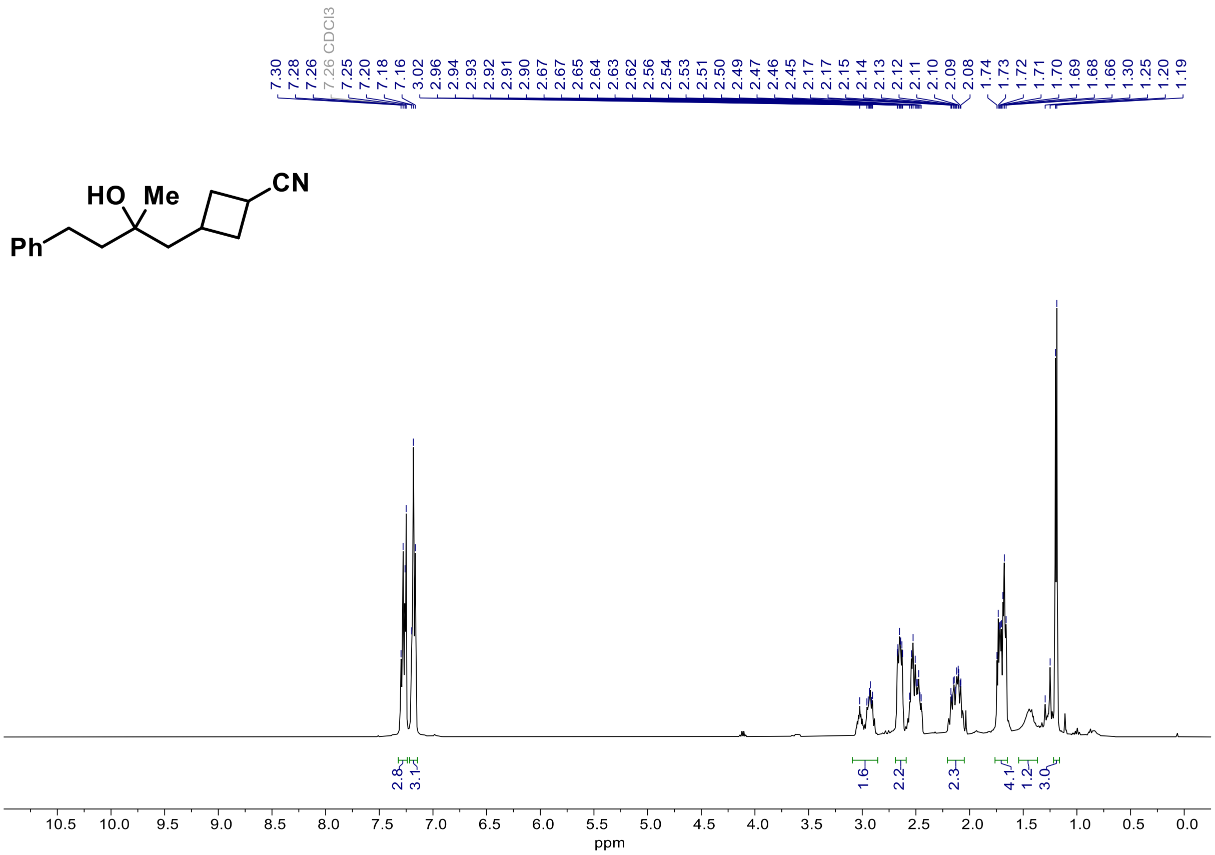

22 – ^{13}C NMR (101 MHz, CDCl$_3$)

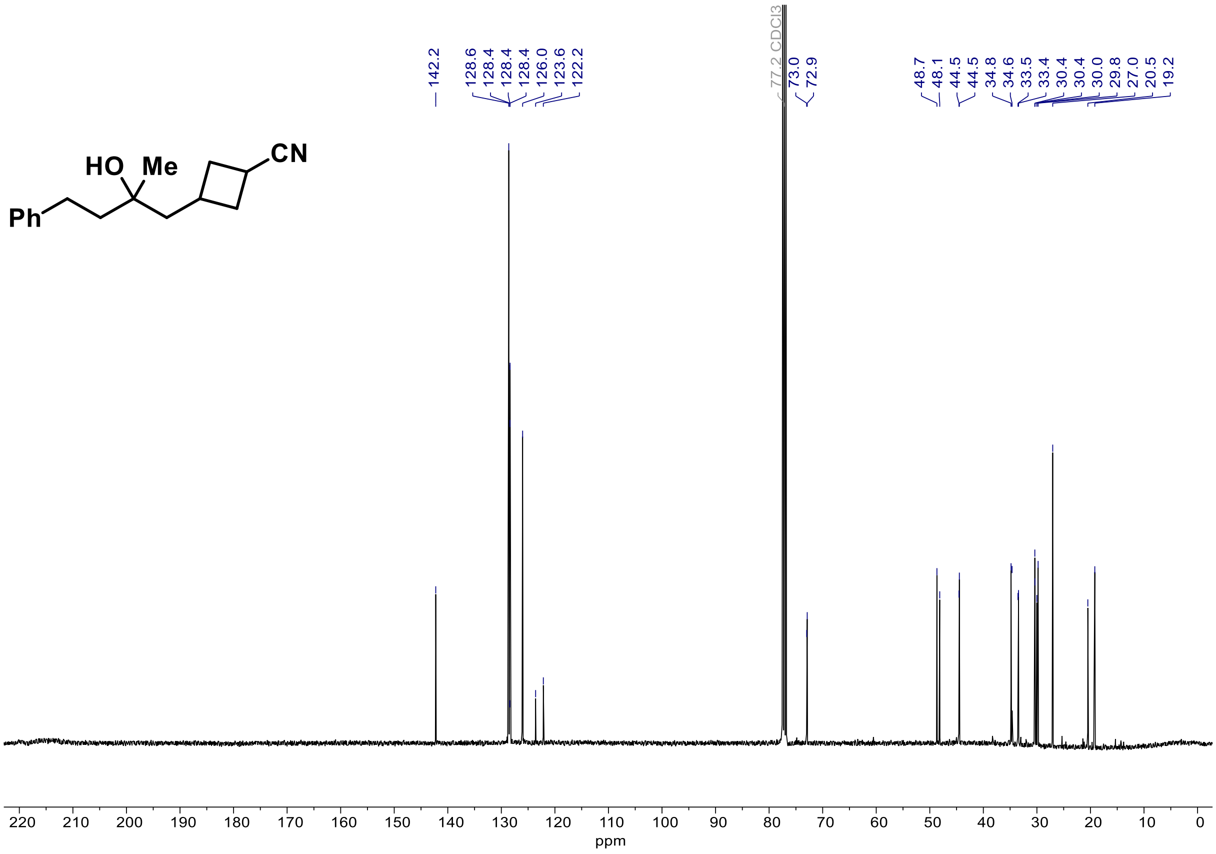

23 – ^{1}H NMR (400 MHz, $CDCl_3$)

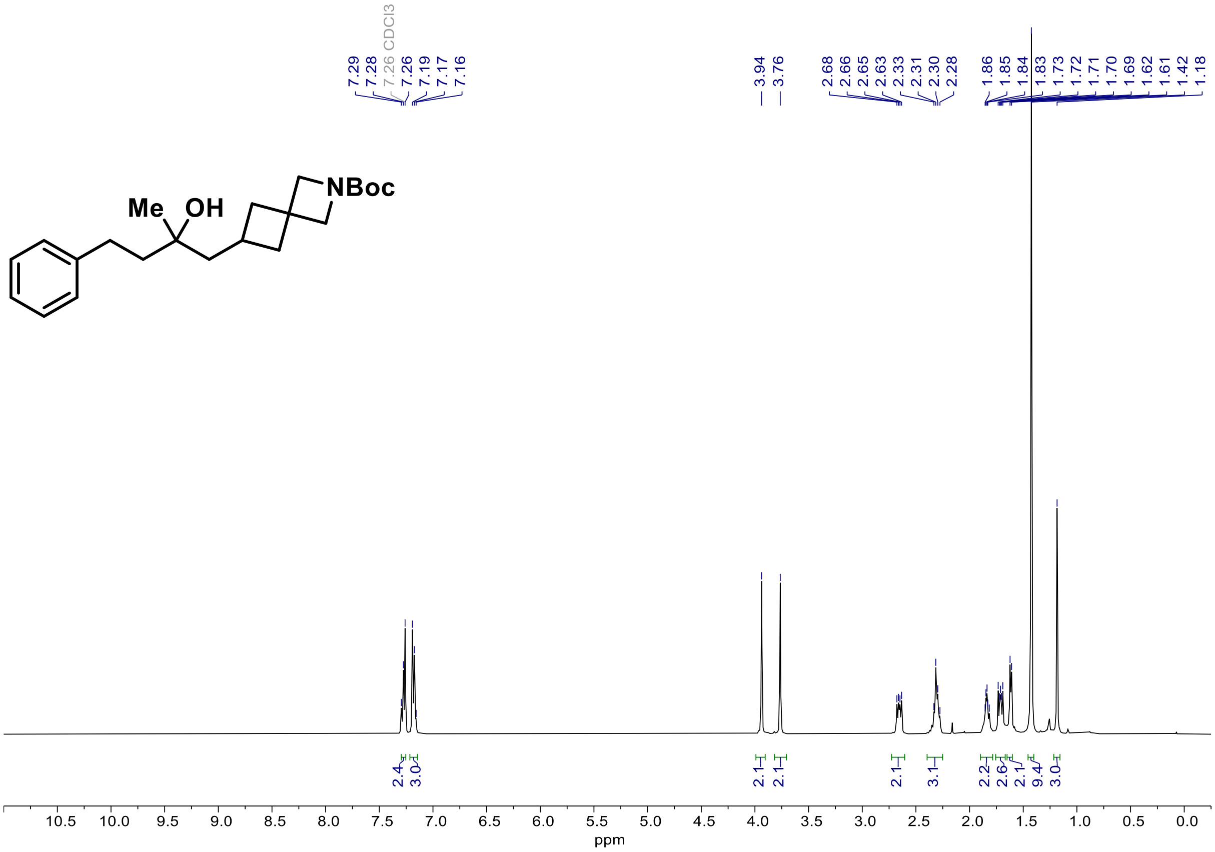

23 – ^{13}C NMR (101 MHz, $CDCl_3$)

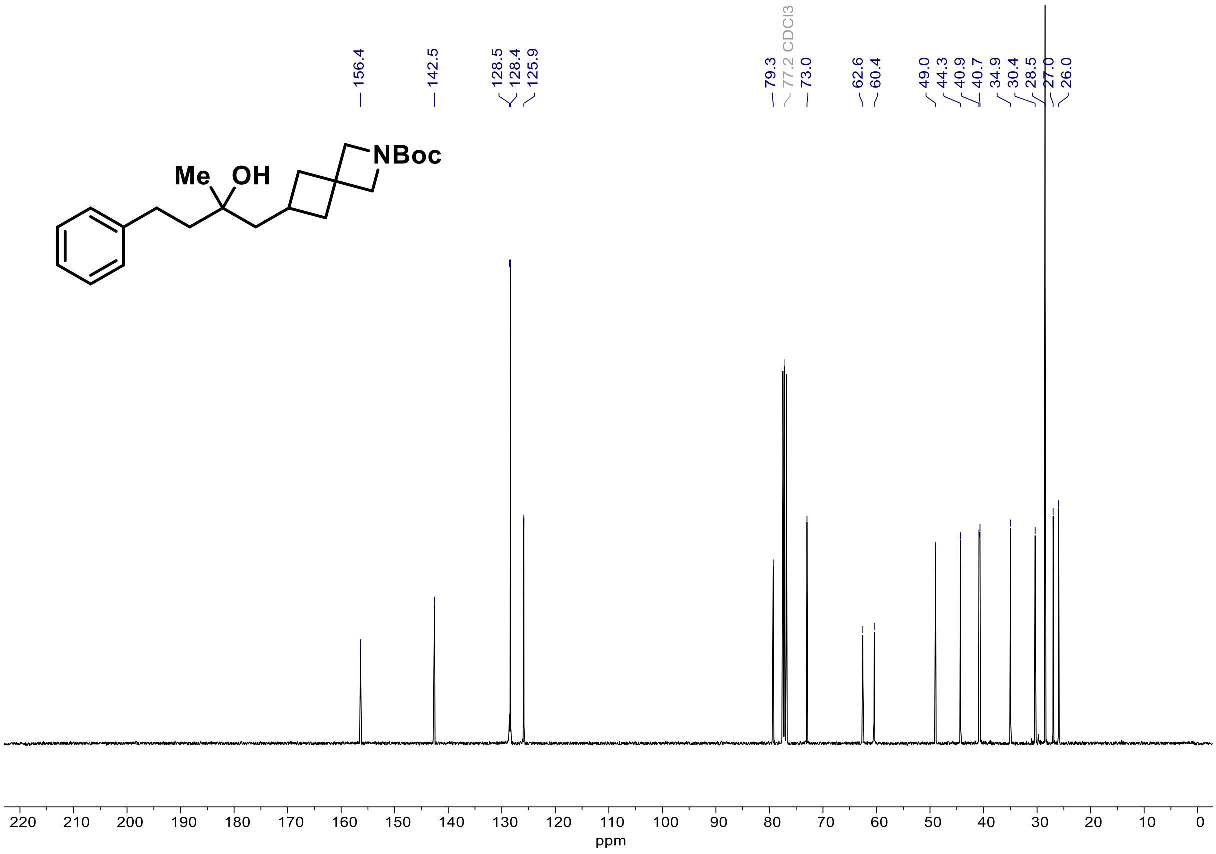

24 – ^{1}H NMR (400 MHz, $CDCl_3$)

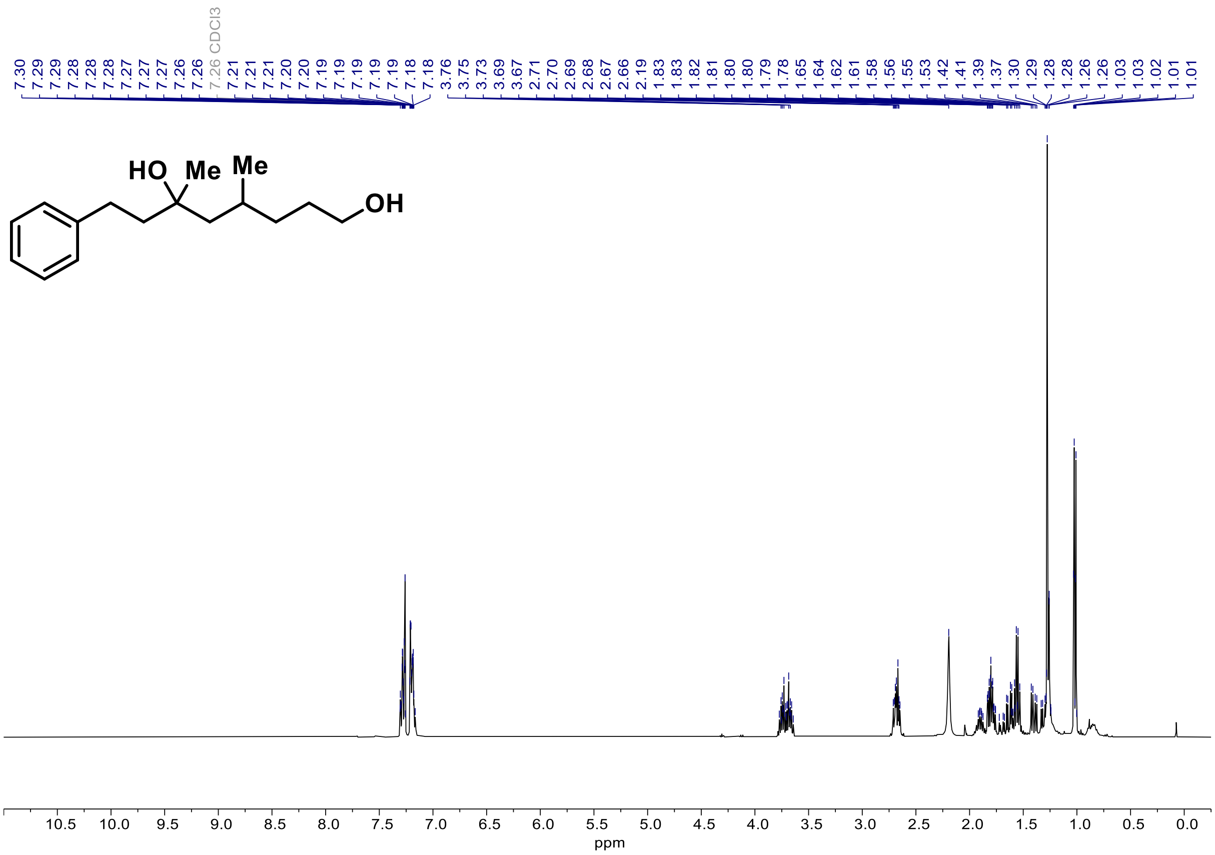

24 – ^{13}C NMR (101 MHz, $CDCl_3$)

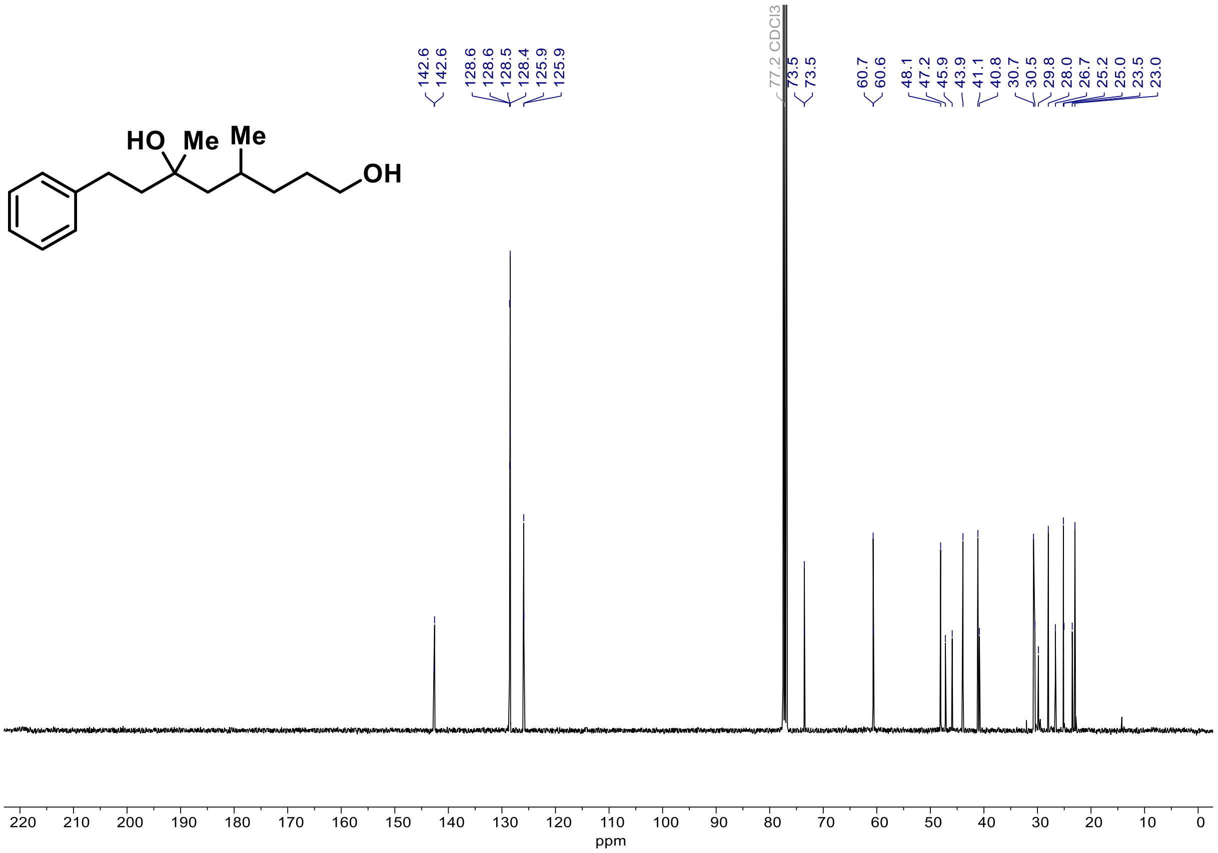

25 – ^{1}H NMR (400 MHz, $CDCl_3$)

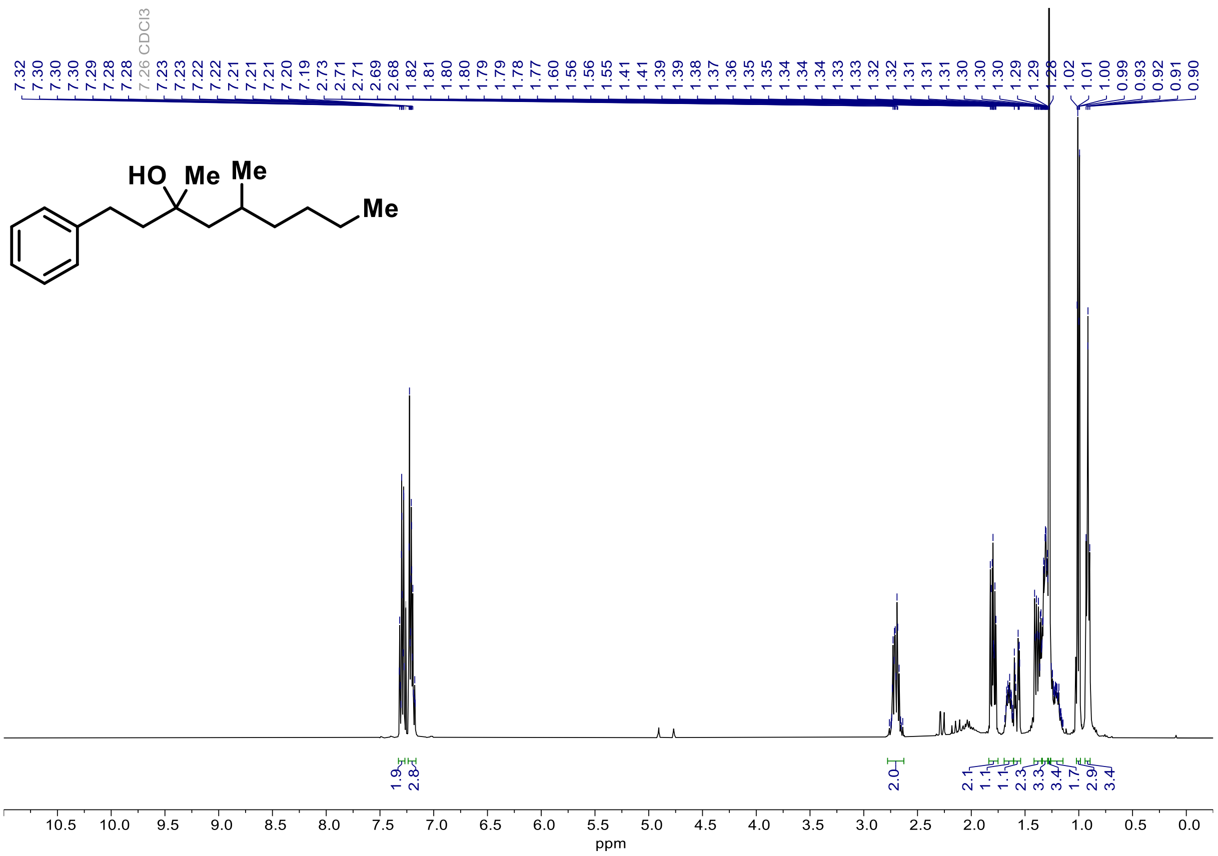

25 – ^{13}C NMR (101 MHz, $CDCl_3$)

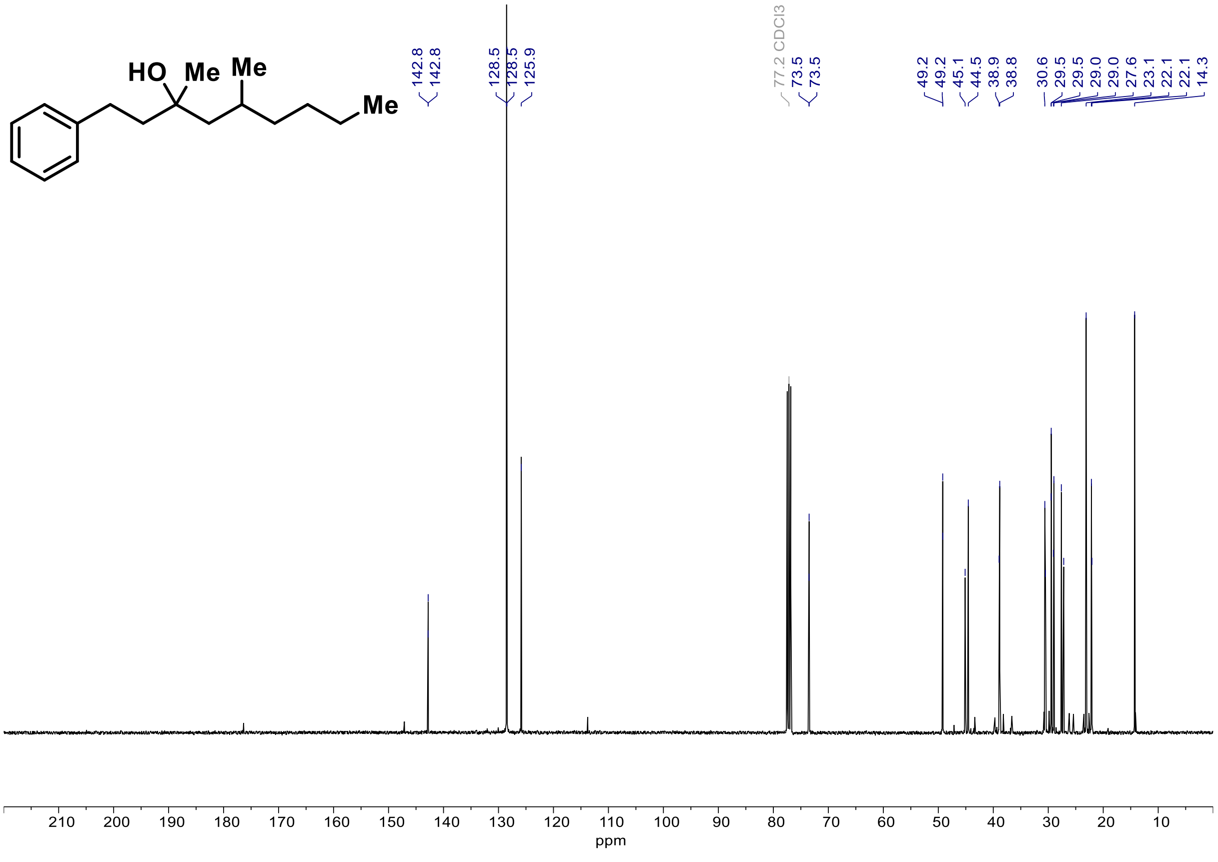

26 – ^{1}H NMR (400 MHz, $CDCl_3$)

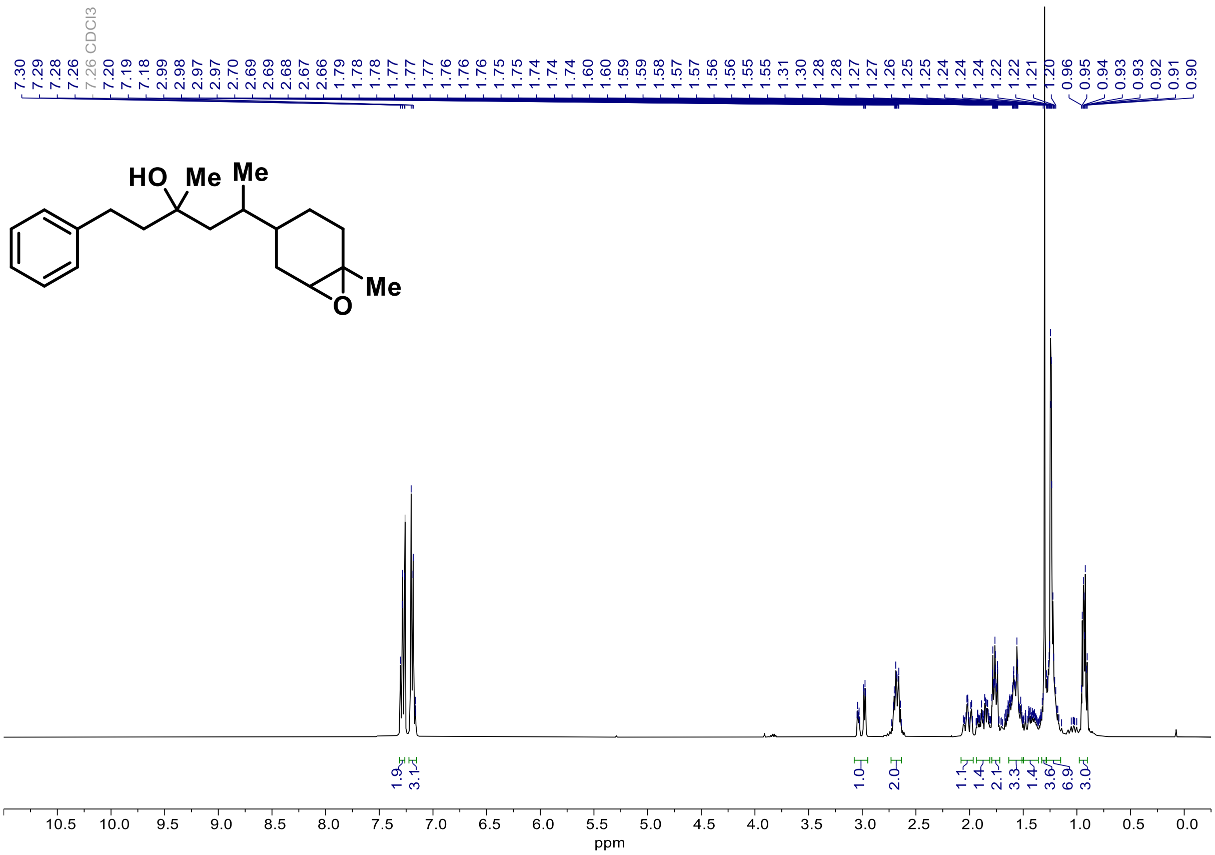

26 – ^{13}C NMR (101 MHz, $CDCl_3$)

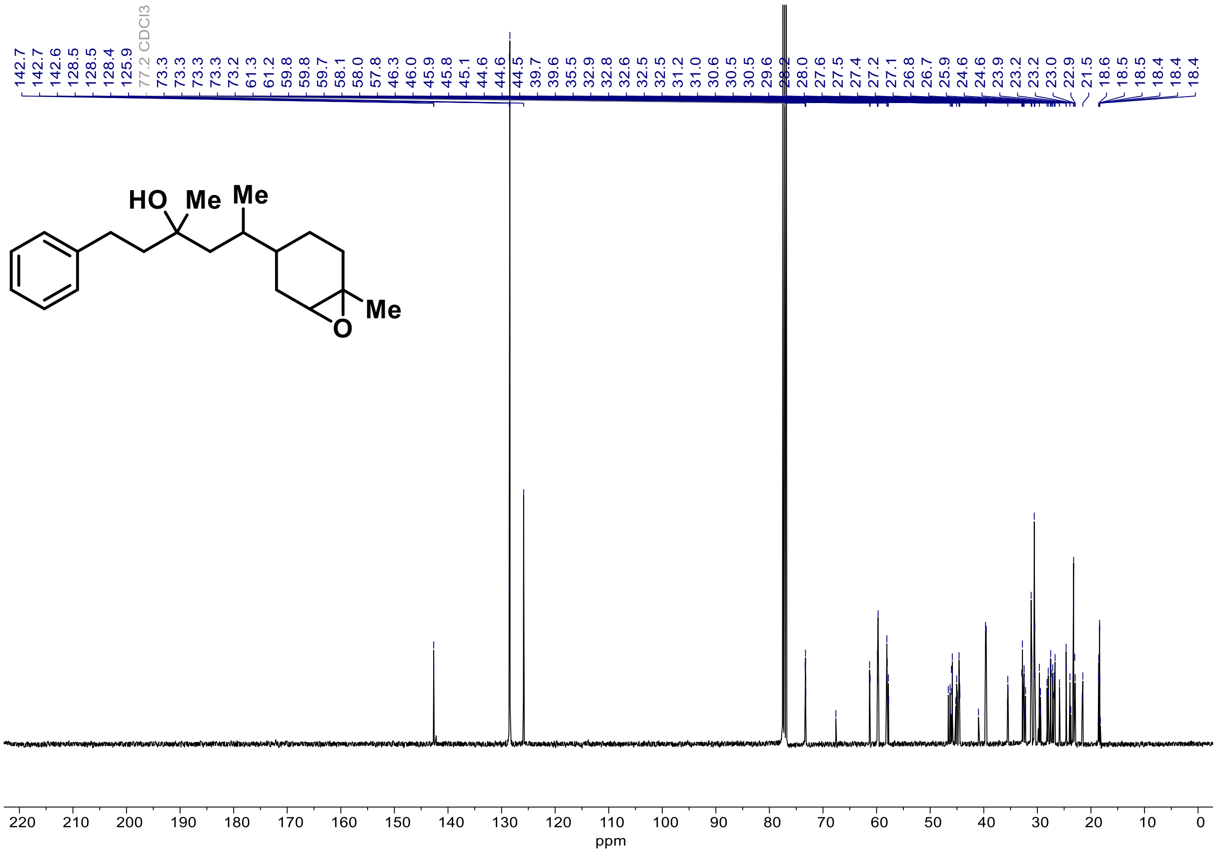

27 – ^{1}H NMR (400 MHz, DMSO-*d6*)

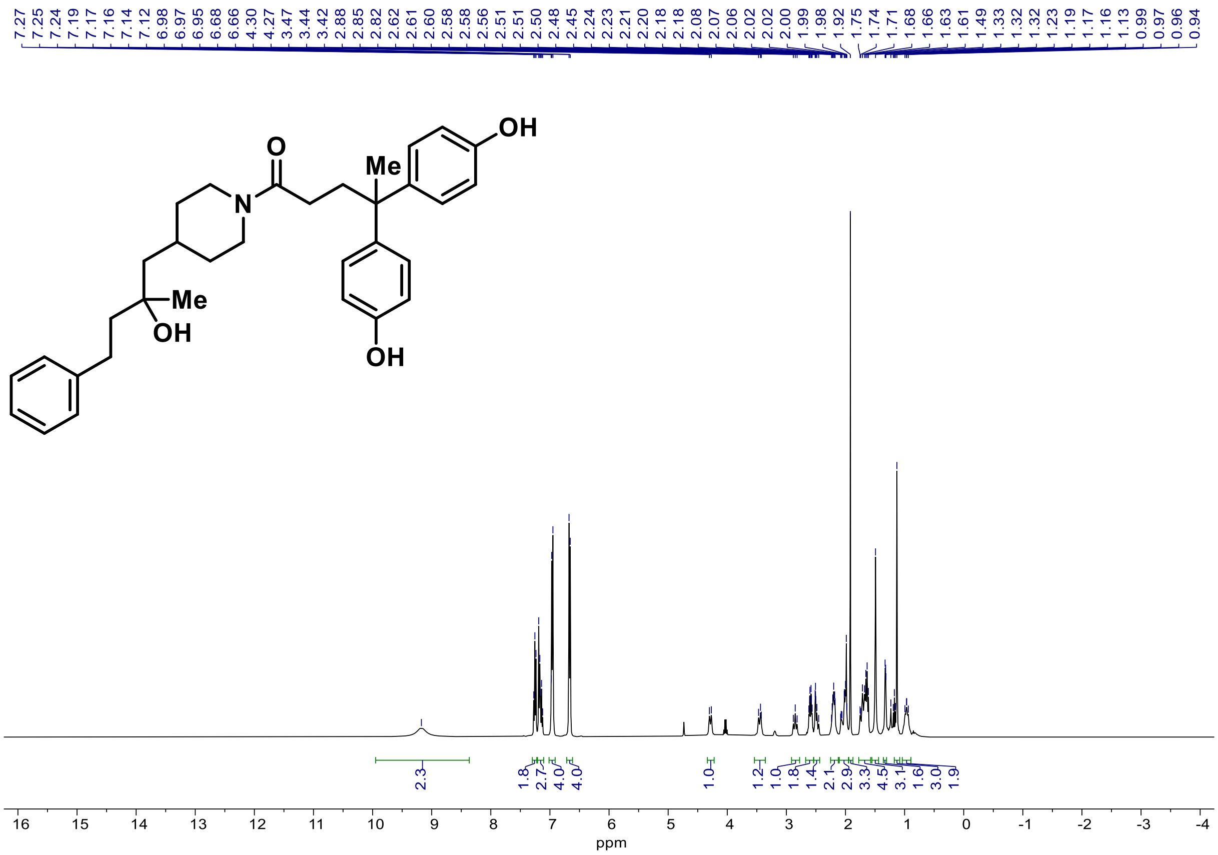

27 – ^{13}C NMR (101 MHz, DMSO-*d6*)

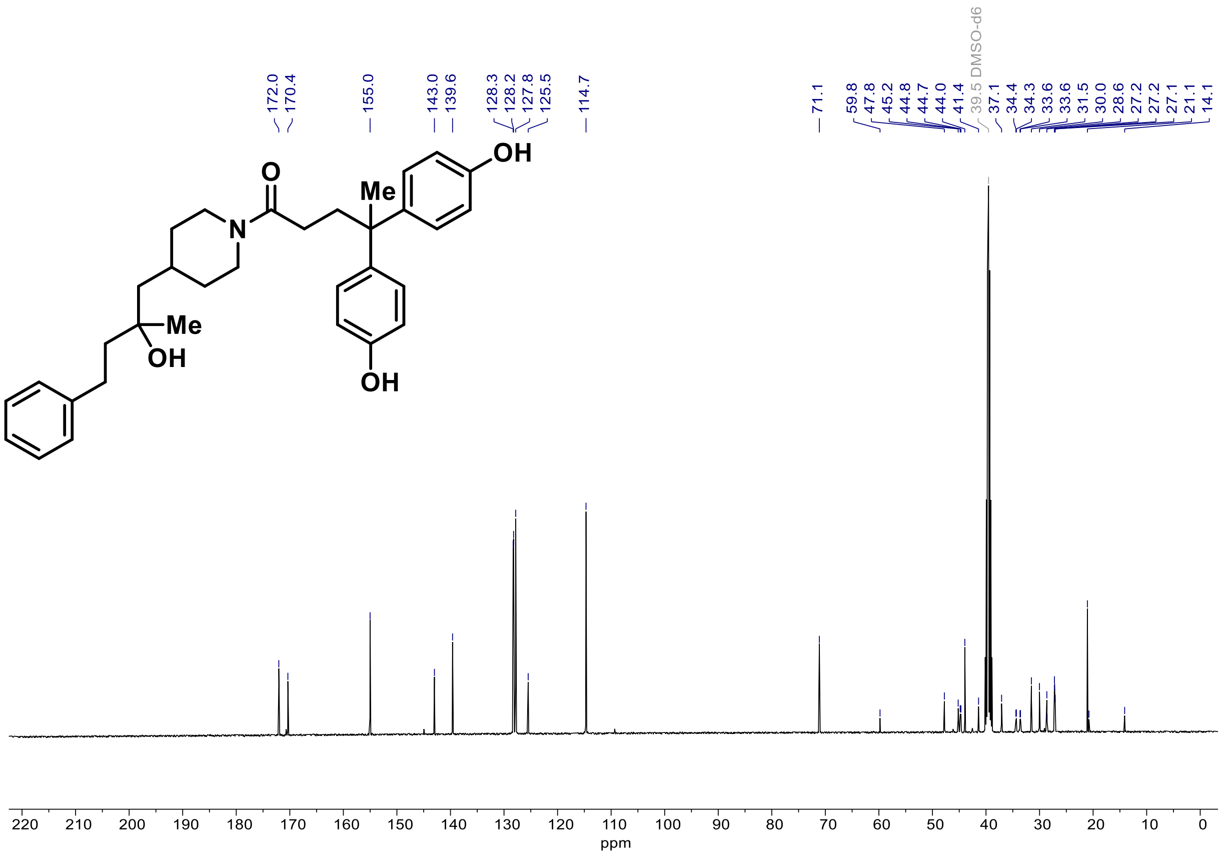

28 – ^{1}H NMR (400 MHz, MeOH-*d4*)

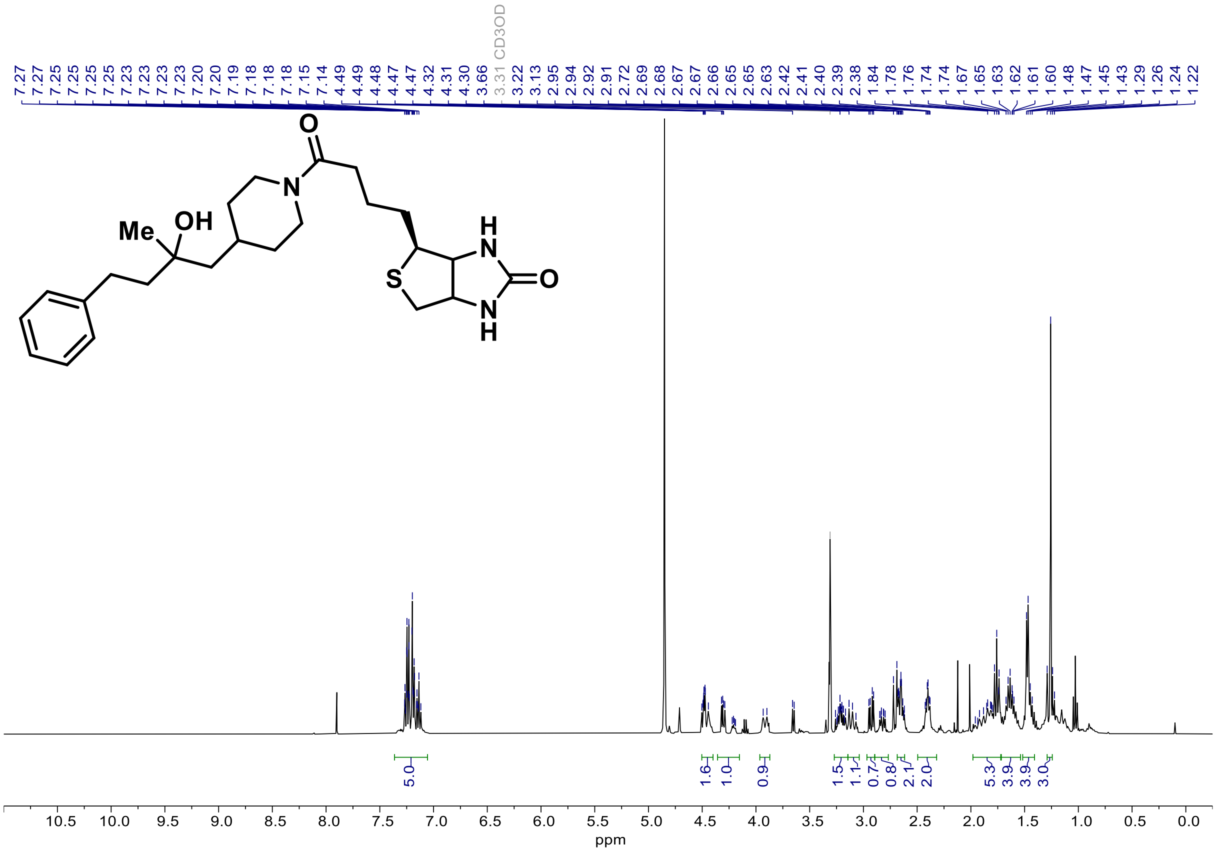

28 – ^{13}C NMR (101 MHz, MeOH-*d4*)

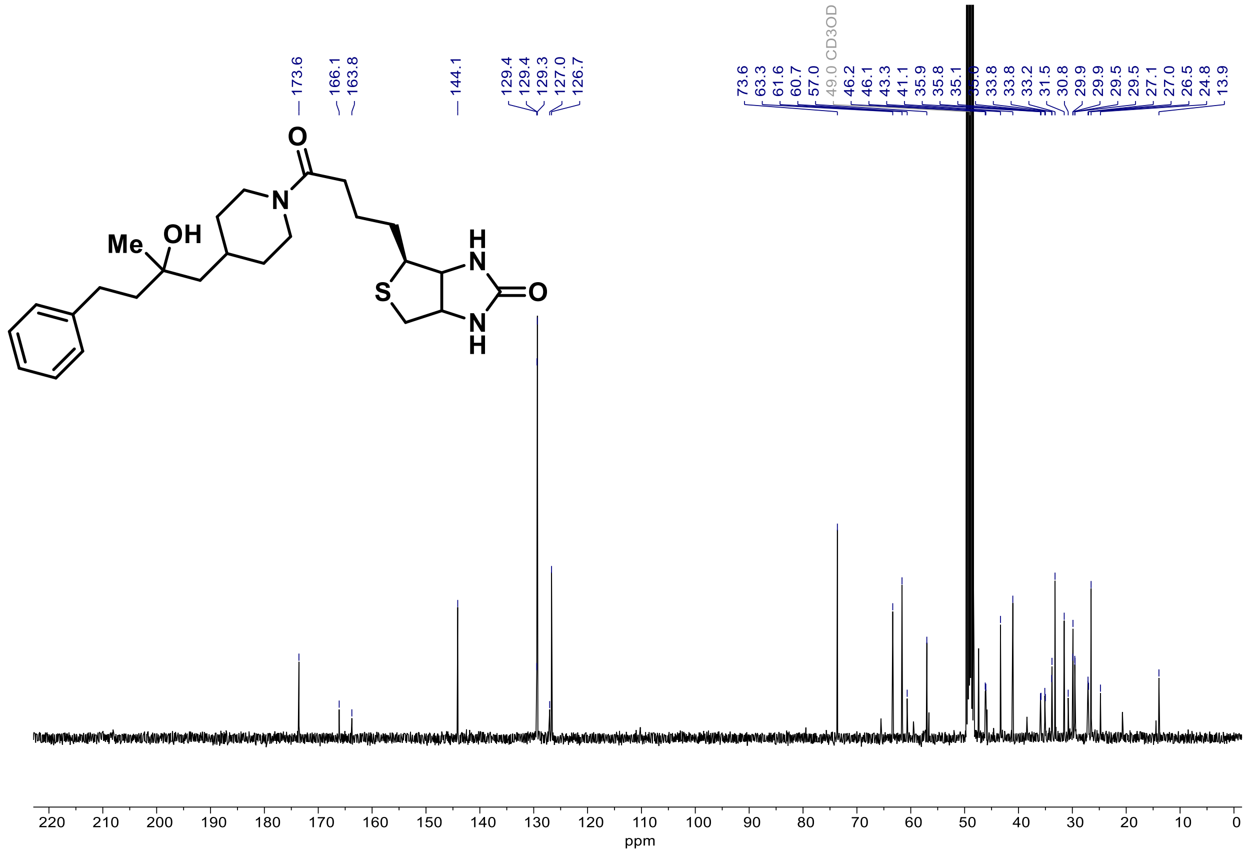

29 – ^{1}H NMR (400 MHz, CDCl3)

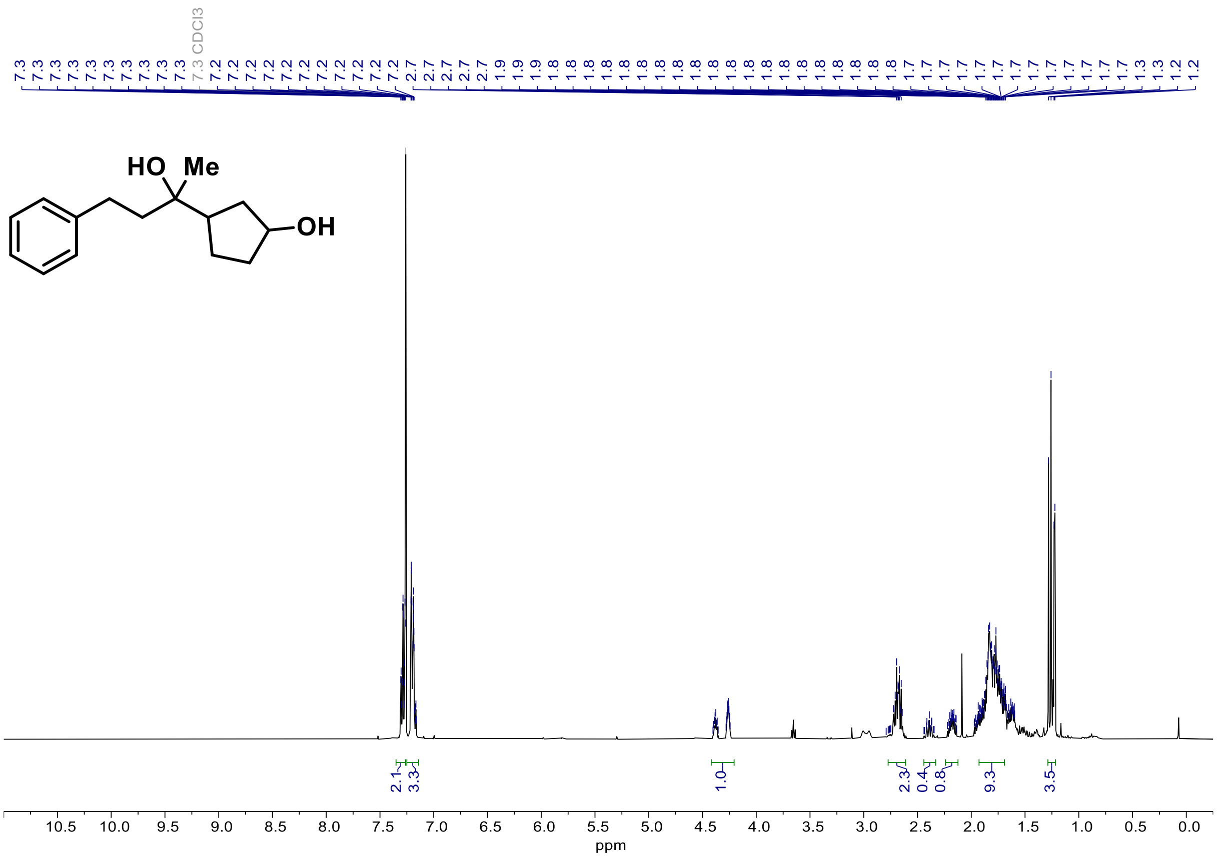

29 – ^{13}C NMR (101 MHz, CDCl3)

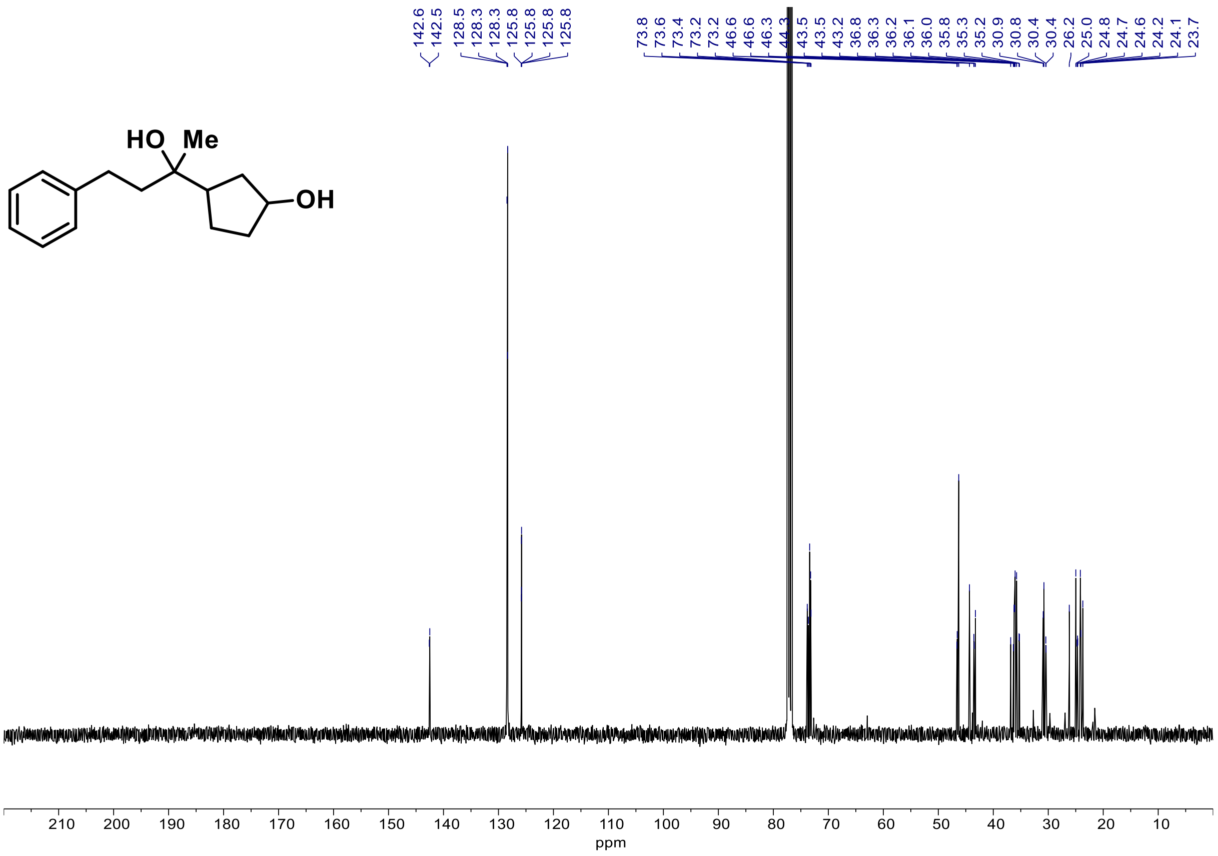

30 – ^{1}H NMR (400 MHz, CDCl$_3$)

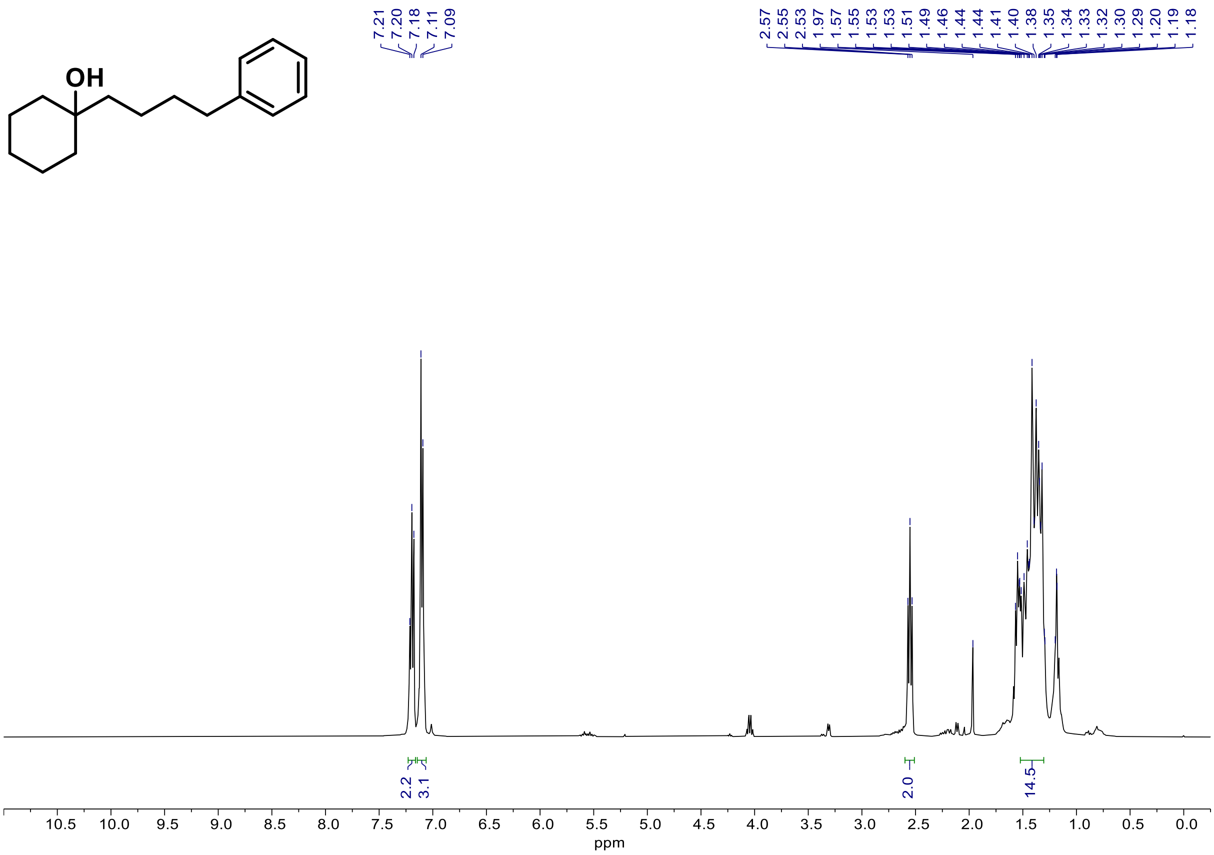

30 – ^{13}C NMR (101 MHz, CDCl$_3$)

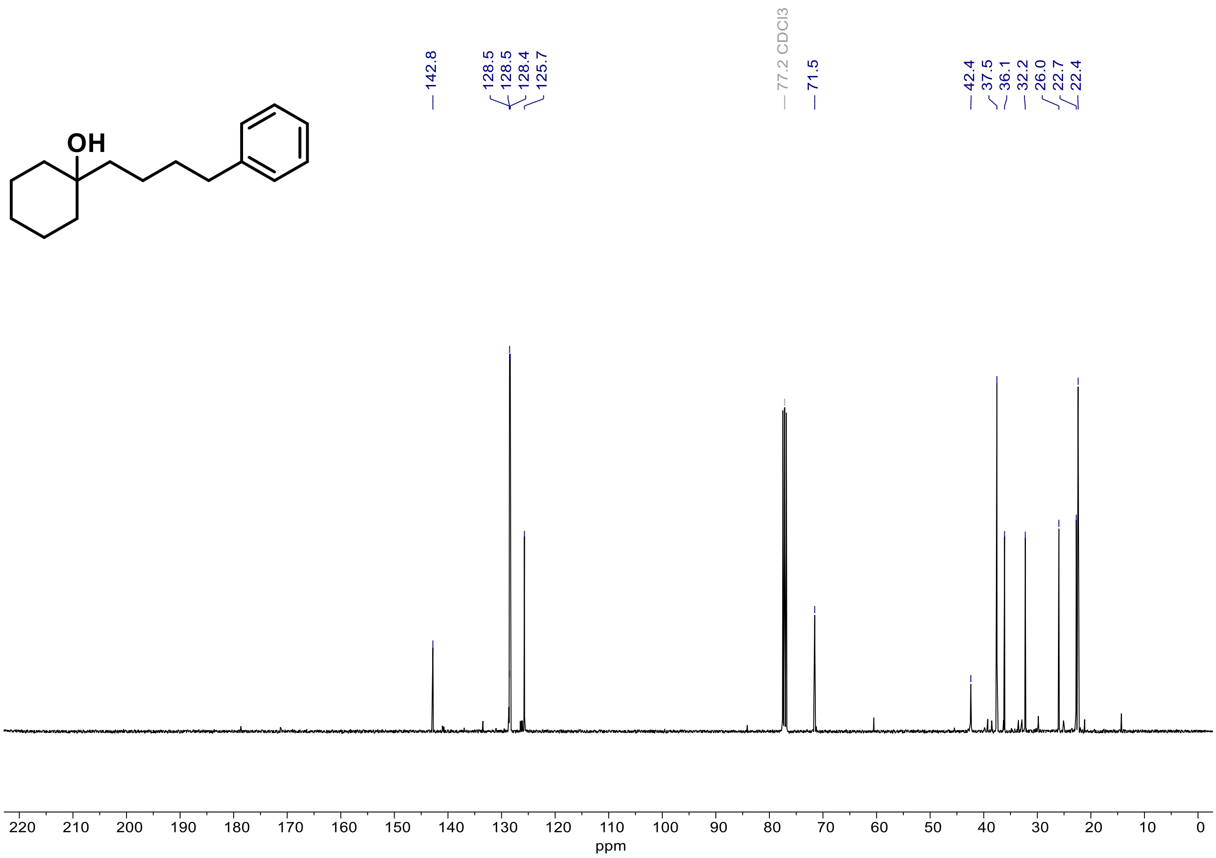

31 – ^{1}H NMR (400 MHz, $CDCl_3$)

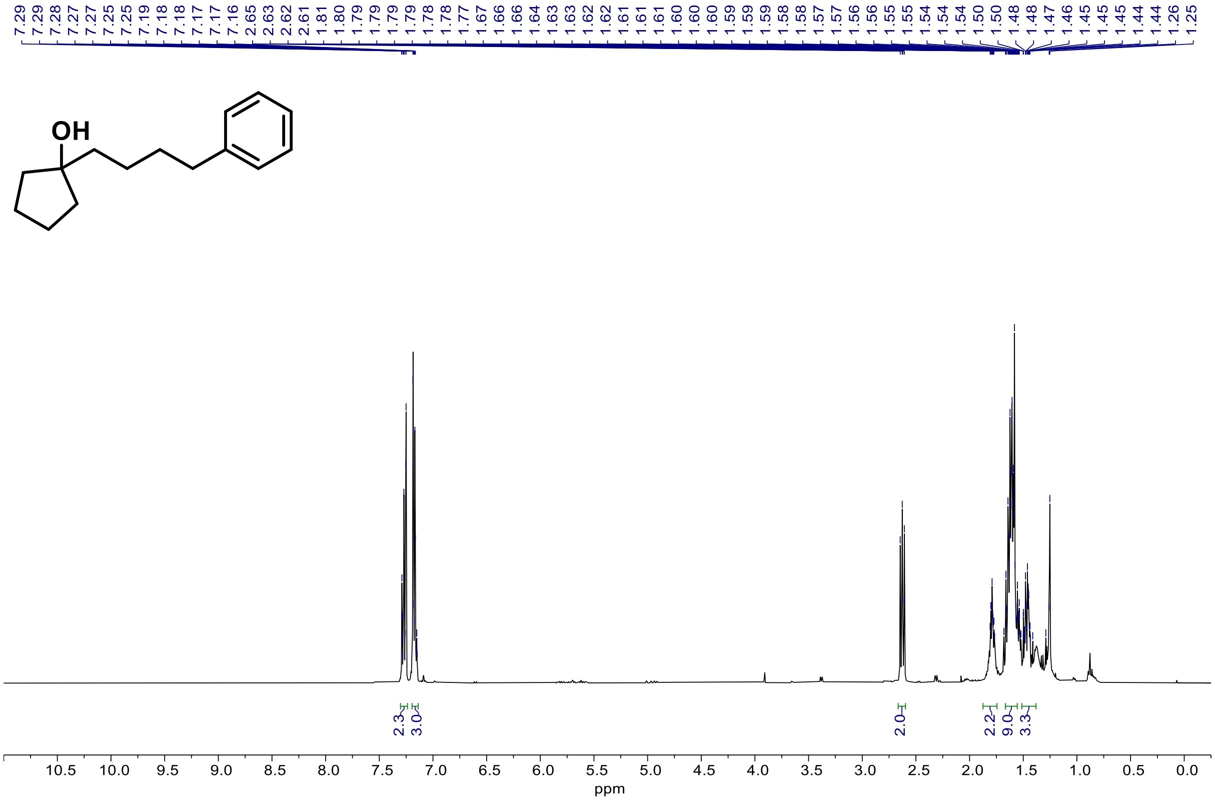

31 – ^{13}C NMR (101 MHz, $CDCl_3$)

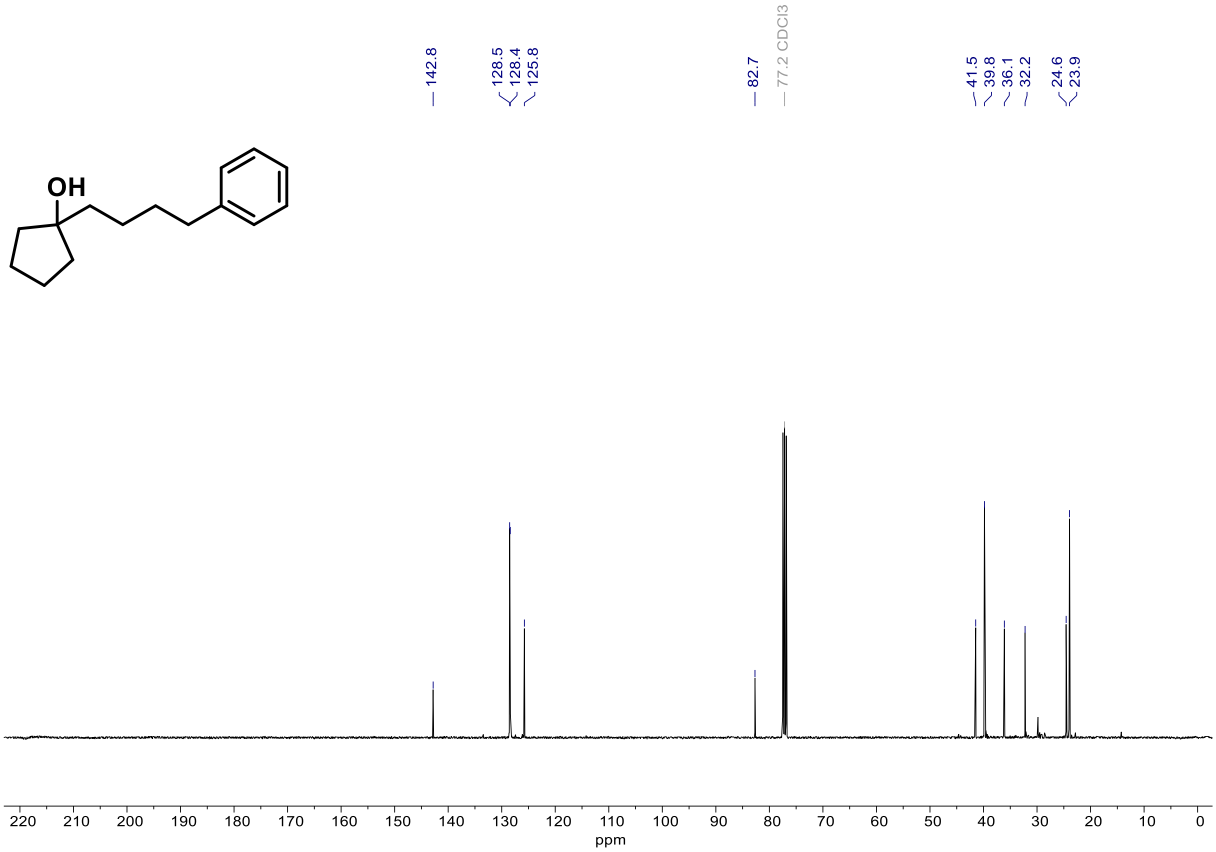

32 – ^{1}H NMR (400 MHz, $CDCl_3$)

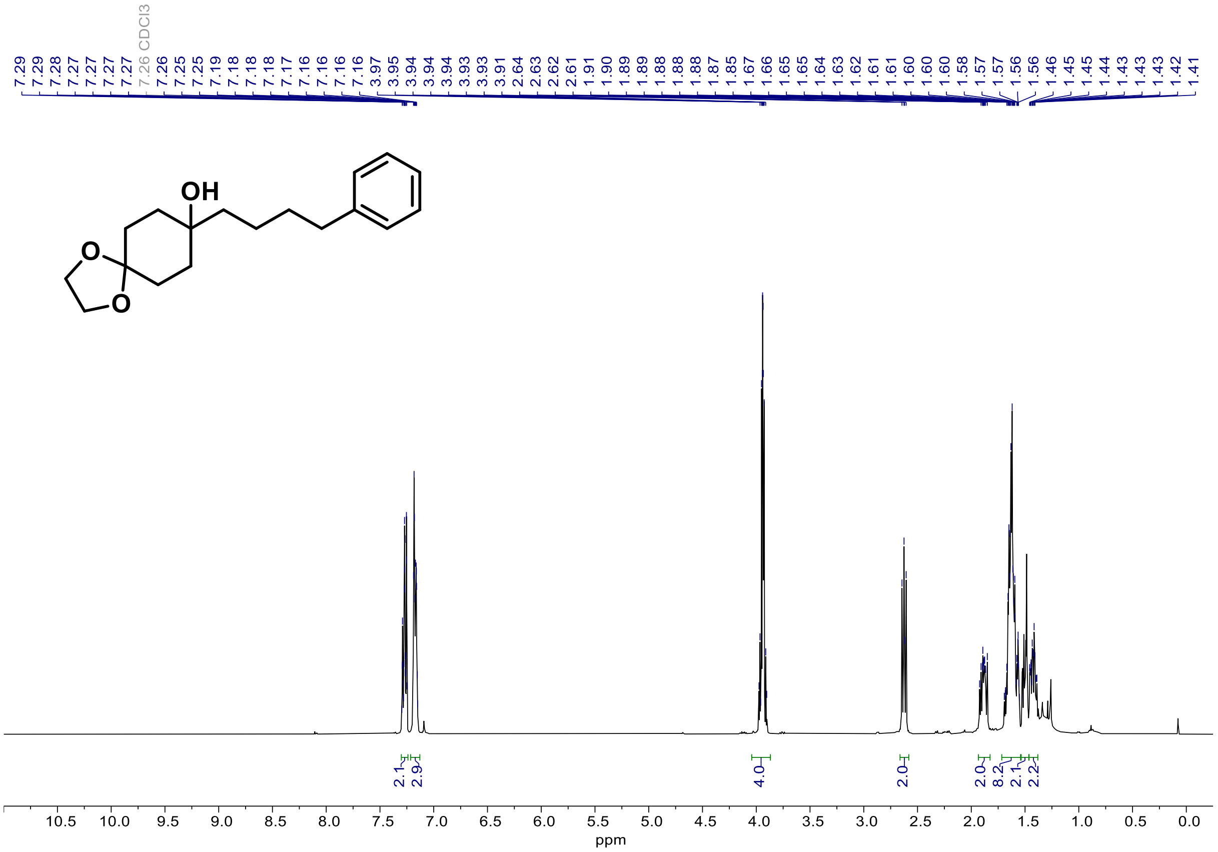

32 – ^{13}C NMR (101 MHz, $CDCl_3$)

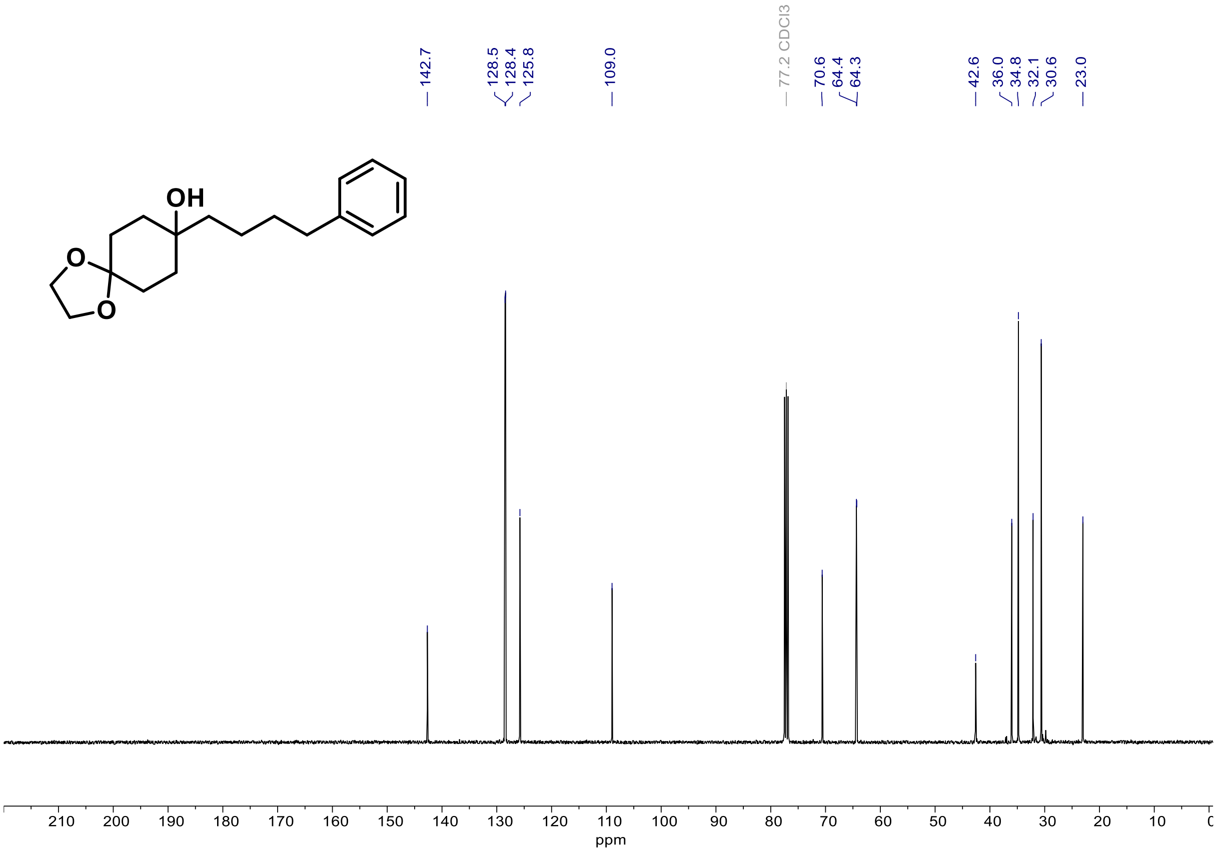

33 – ^{1}H NMR (400 MHz, $CDCl_3$) diastereoisomer 1

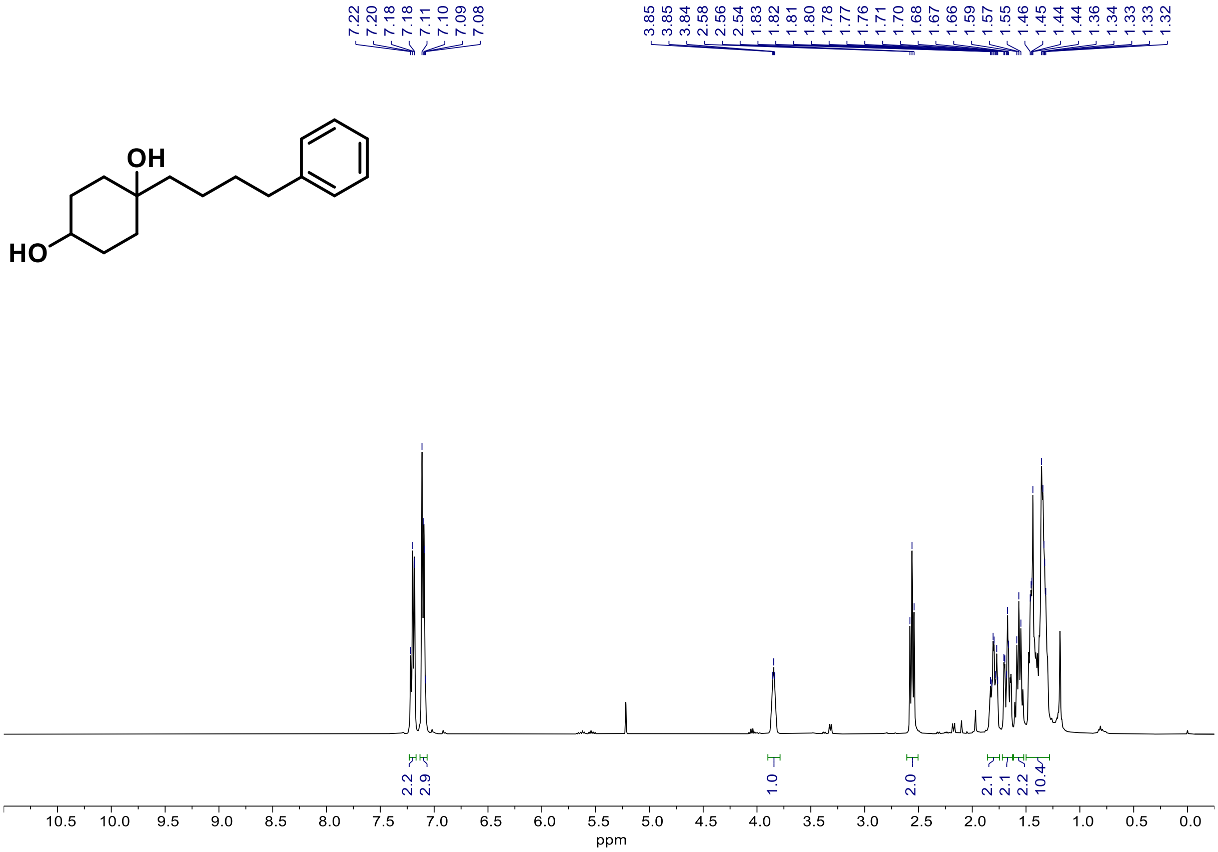

33 – ^{13}C NMR (101 MHz, $CDCl_3$) diastereoisomer 1

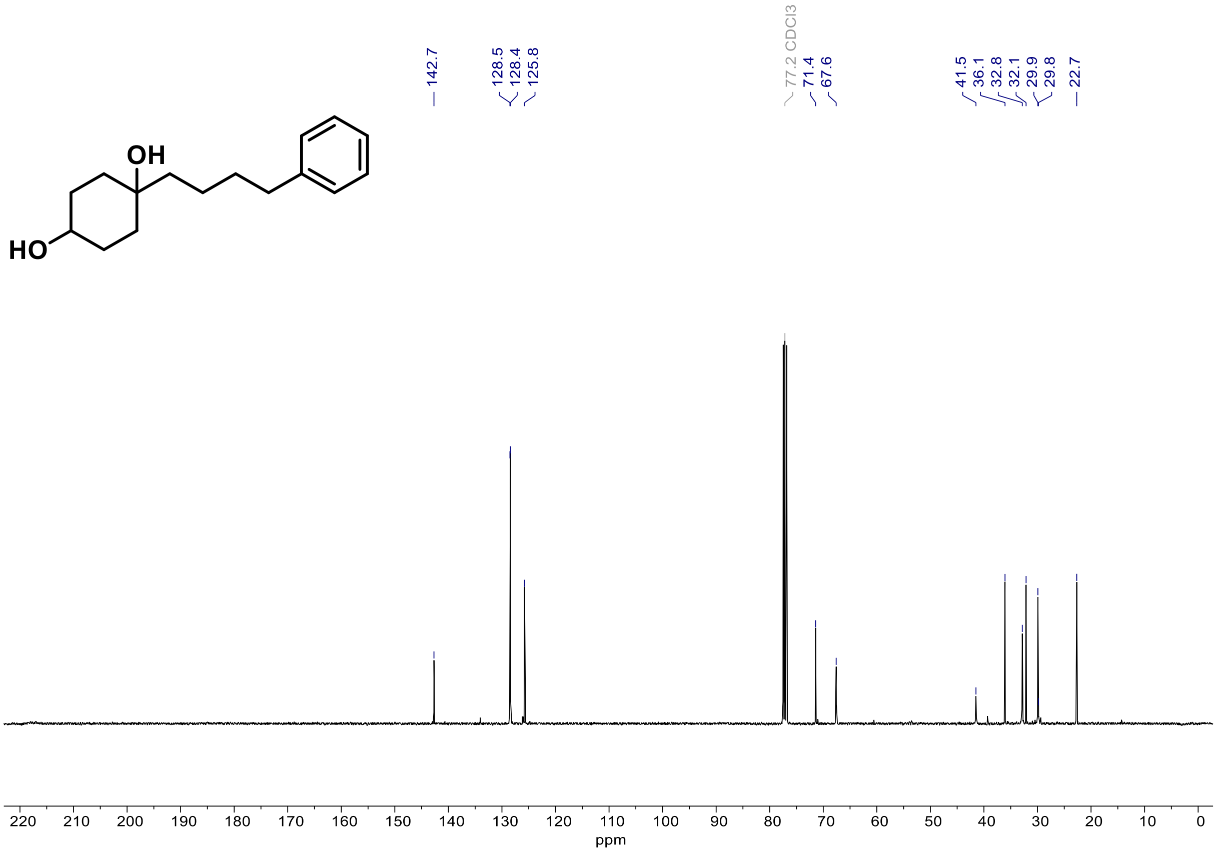

33 – ^{1}H NMR (400 MHz, $CDCl_3$) diastereoisomer 2

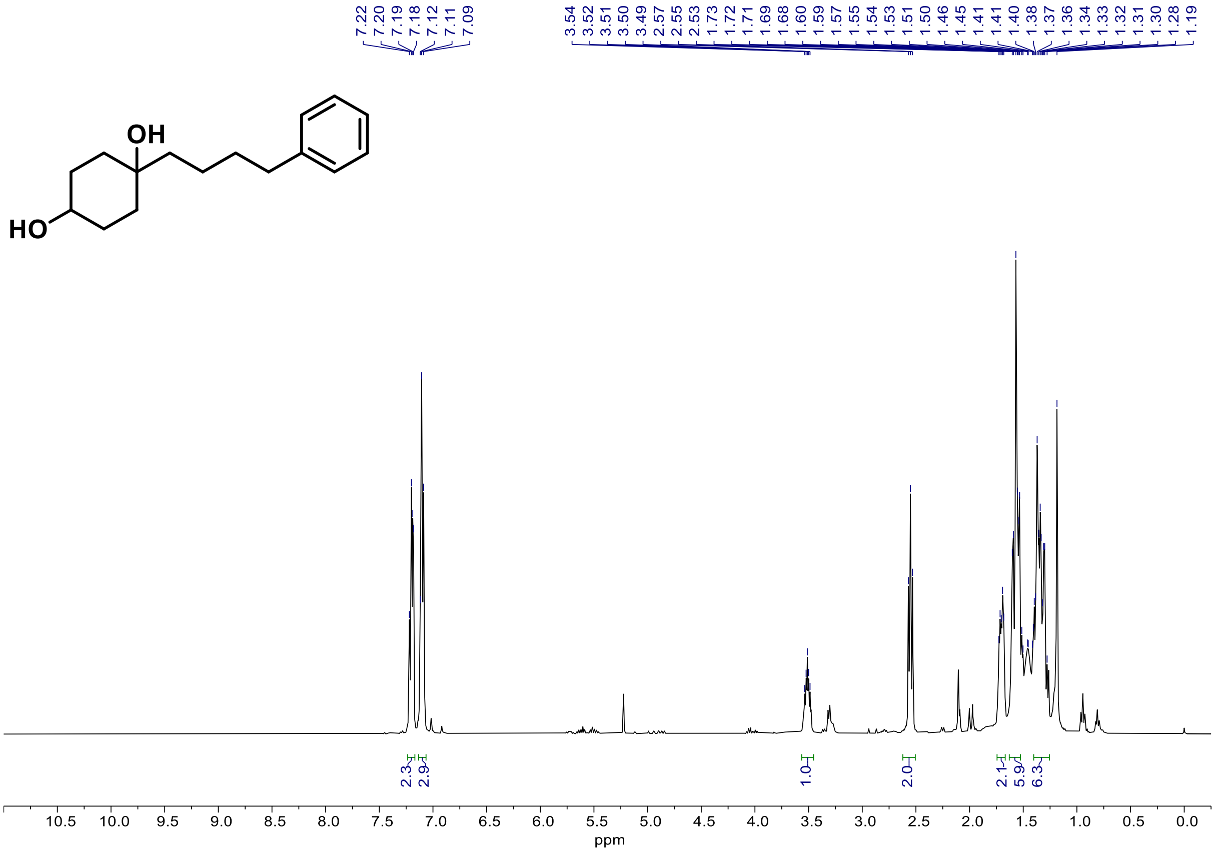

33 – ^{13}C NMR (101 MHz, $CDCl_3$) diastereoisomer 2

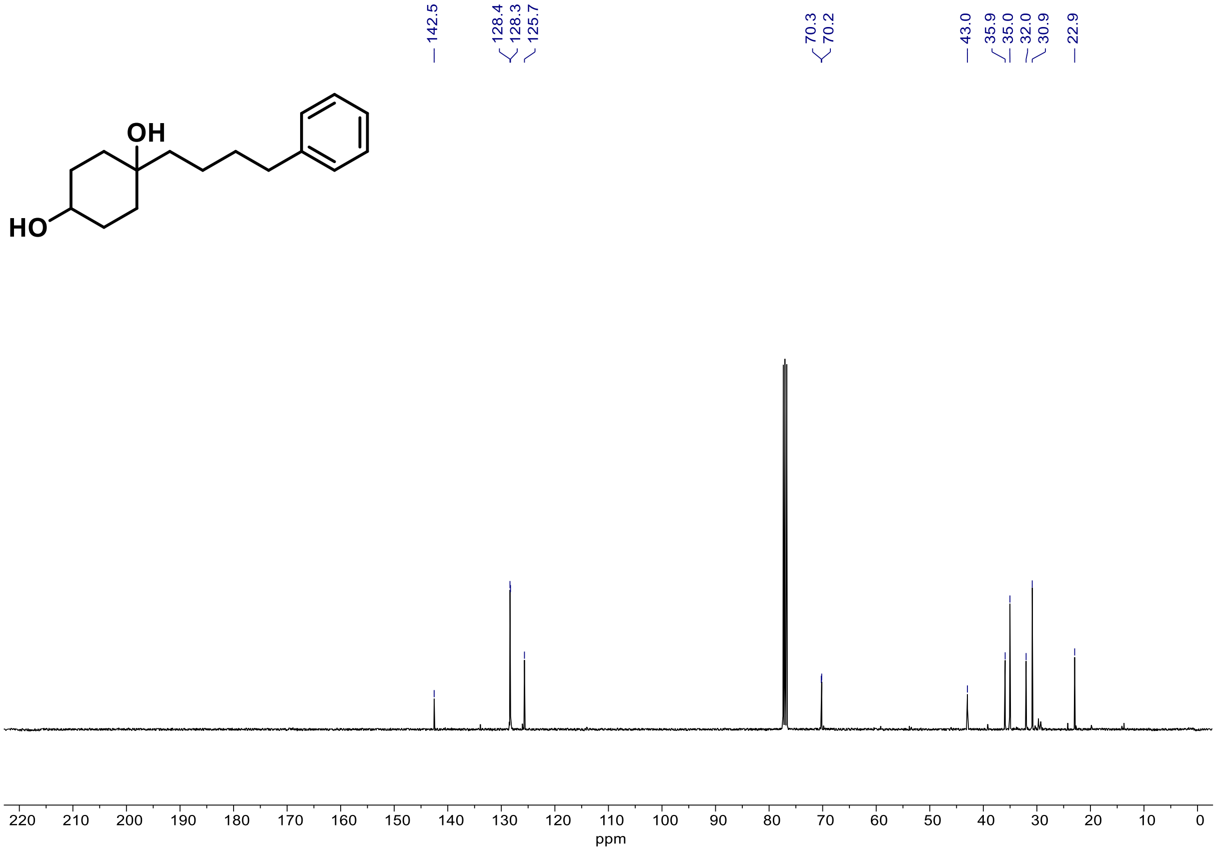

34 – ^{1}H NMR (400 MHz, $CDCl_3$)

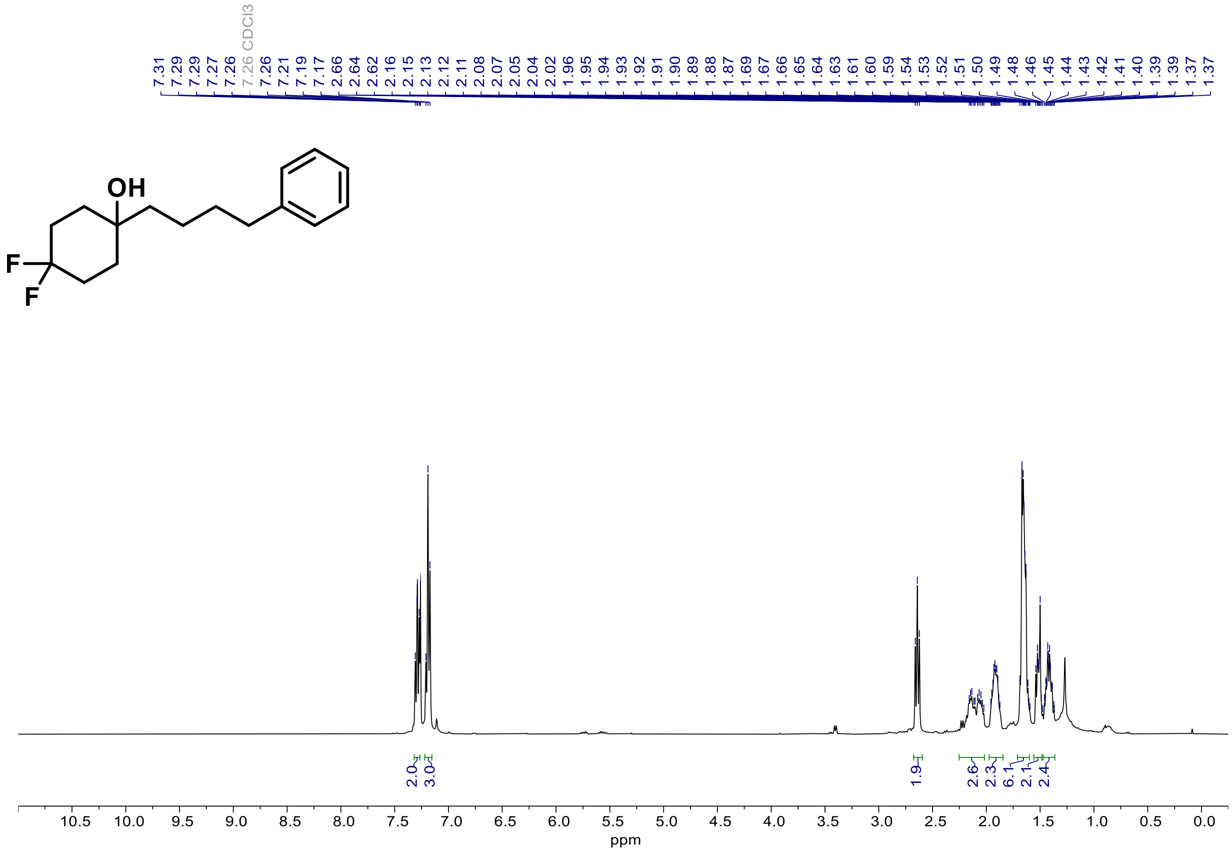

34 – ^{13}C NMR (101 MHz, $CDCl_3$)

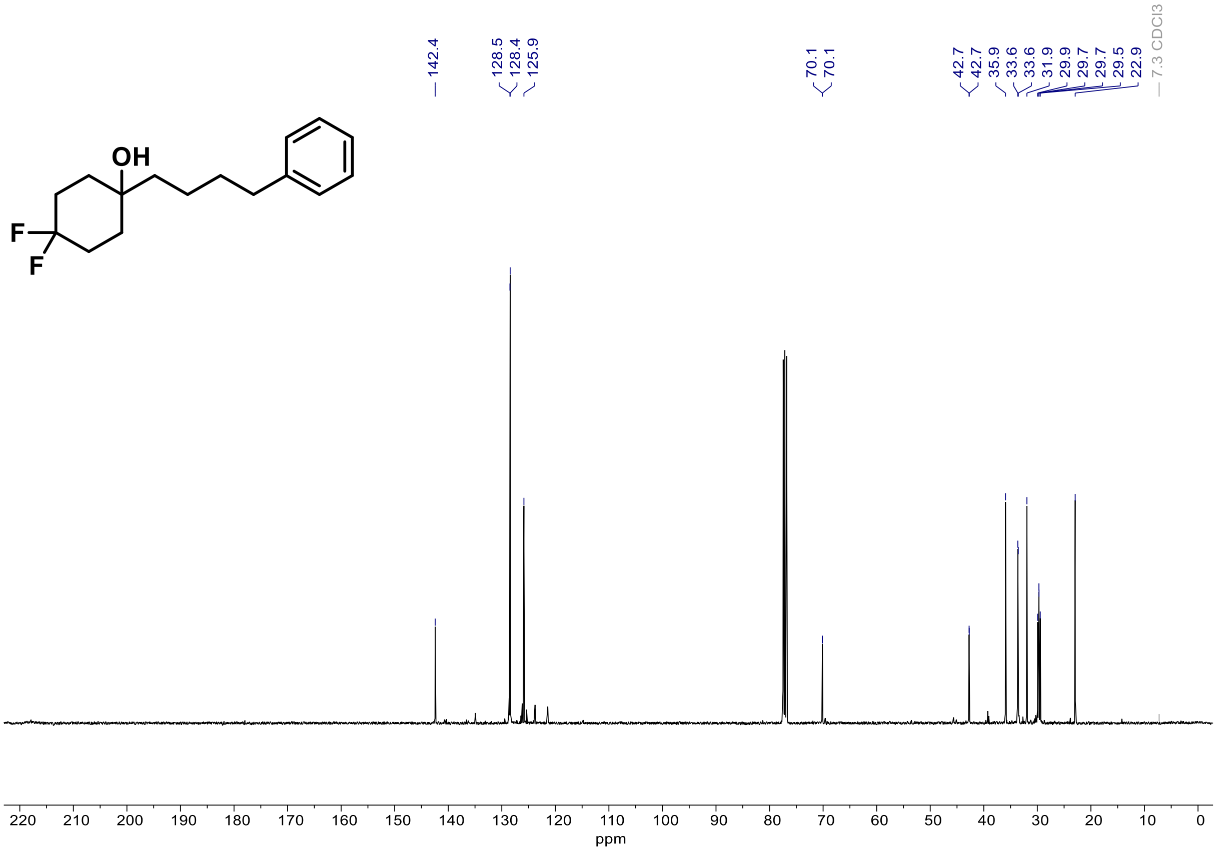

34 – ^{19}F NMR (377 MHz, $CDCl_3$)

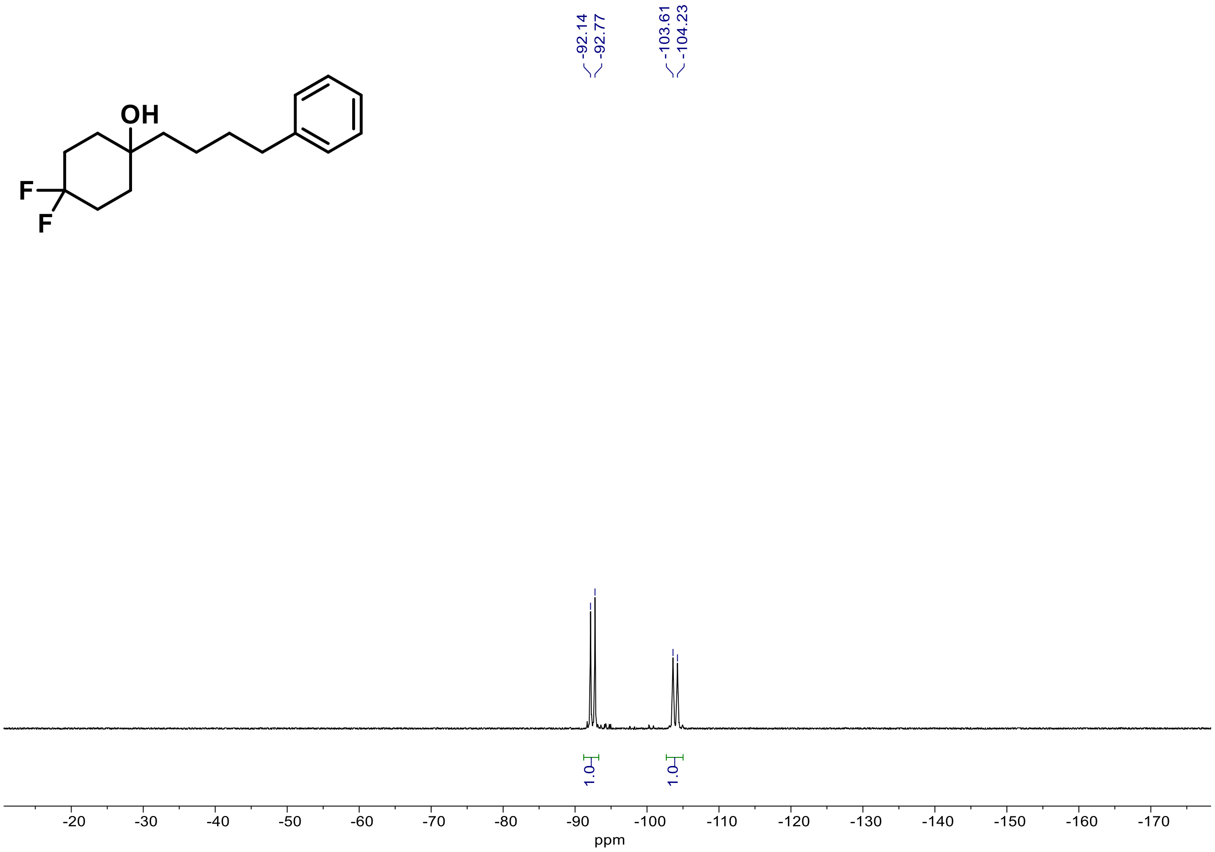

35 – ^{1}H NMR (400 MHz, $CDCl_3$)

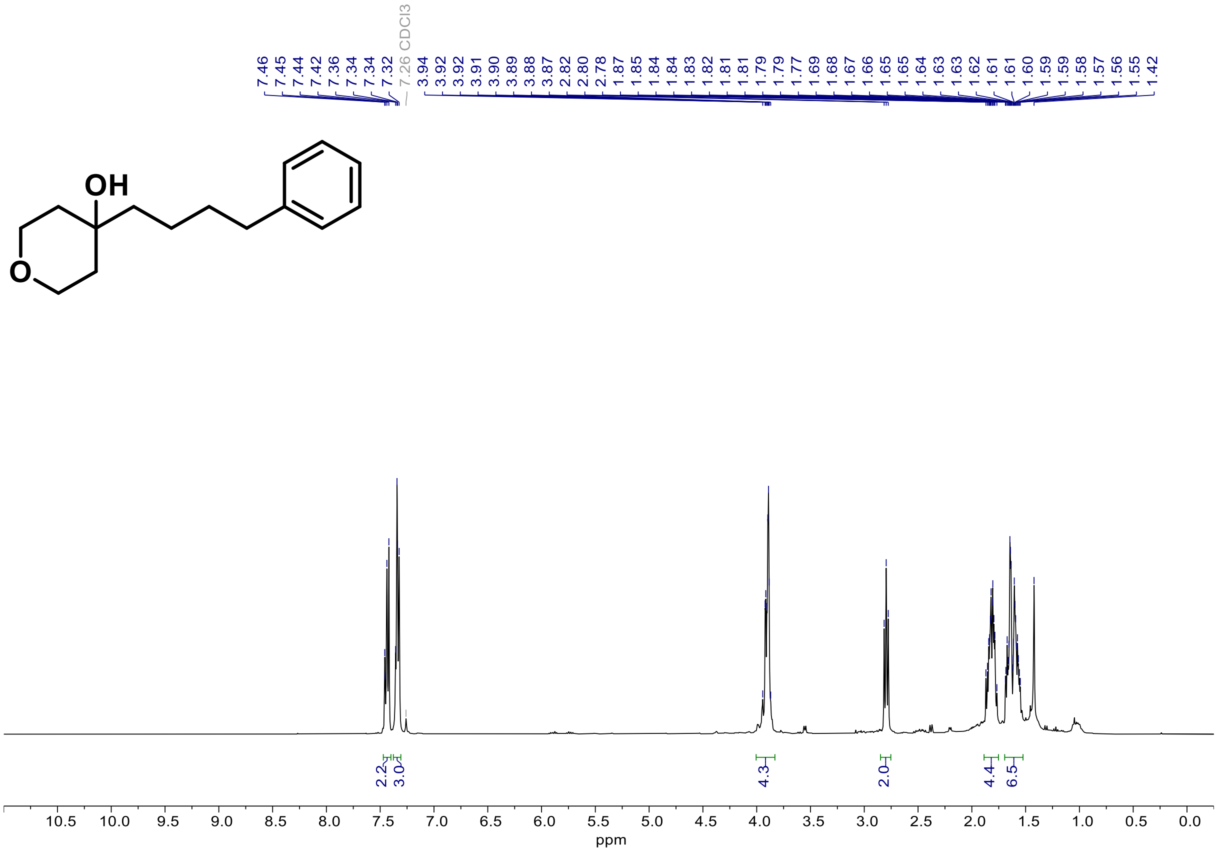

35 – ^{13}C NMR (101 MHz, $CDCl_3$)

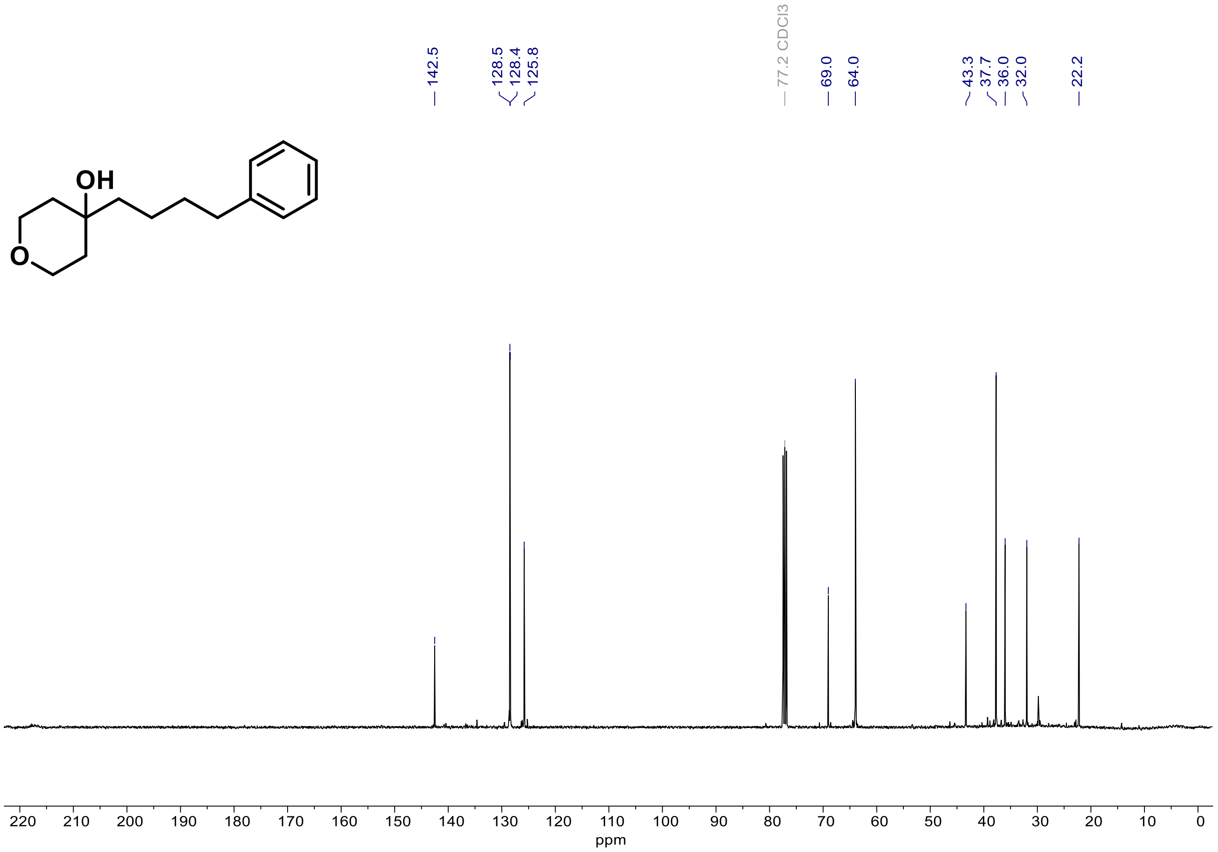

36 – ^{1}H NMR (400 MHz, $CDCl_3$)

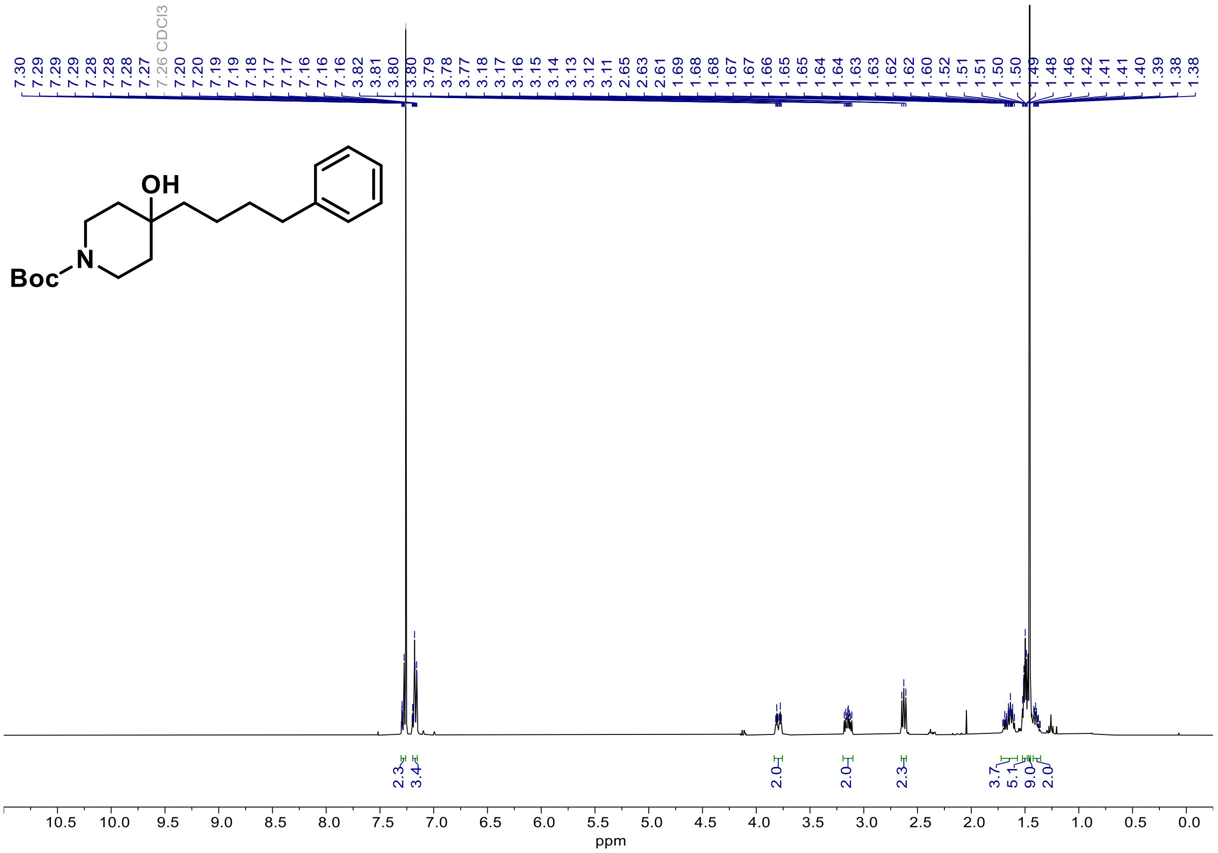

36 – ^{13}C NMR (101 MHz, $CDCl_3$)

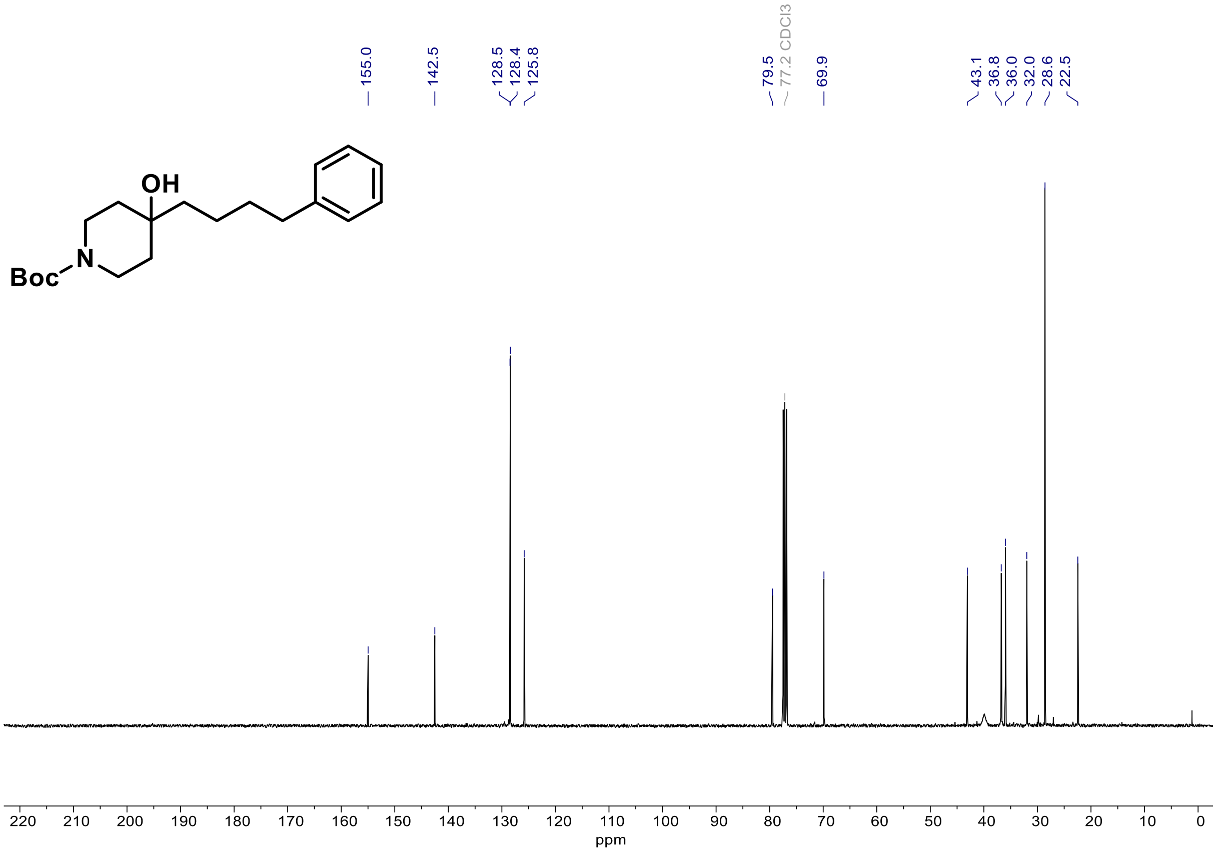

37 – ^{1}H NMR (400 MHz, $CDCl_3$)

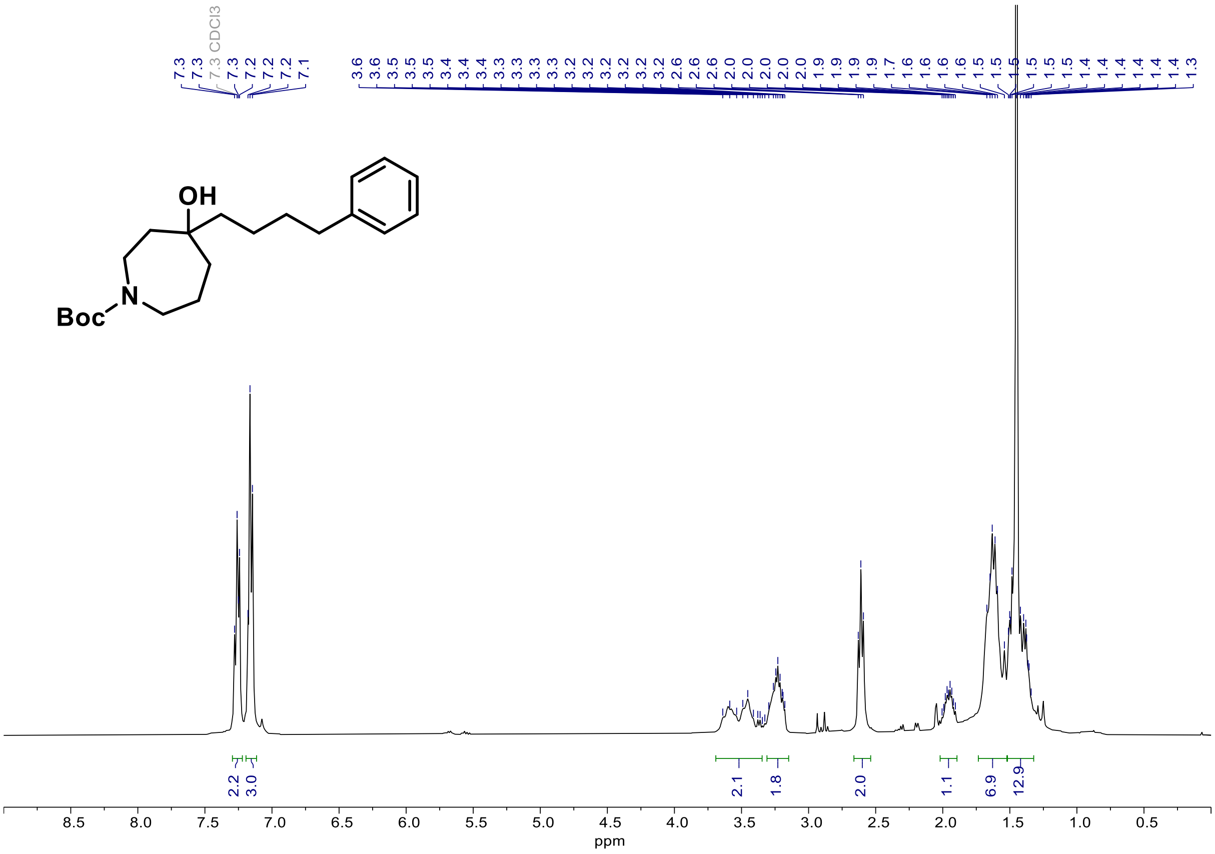

37 – ^{13}C NMR (101 MHz, $CDCl_3$)

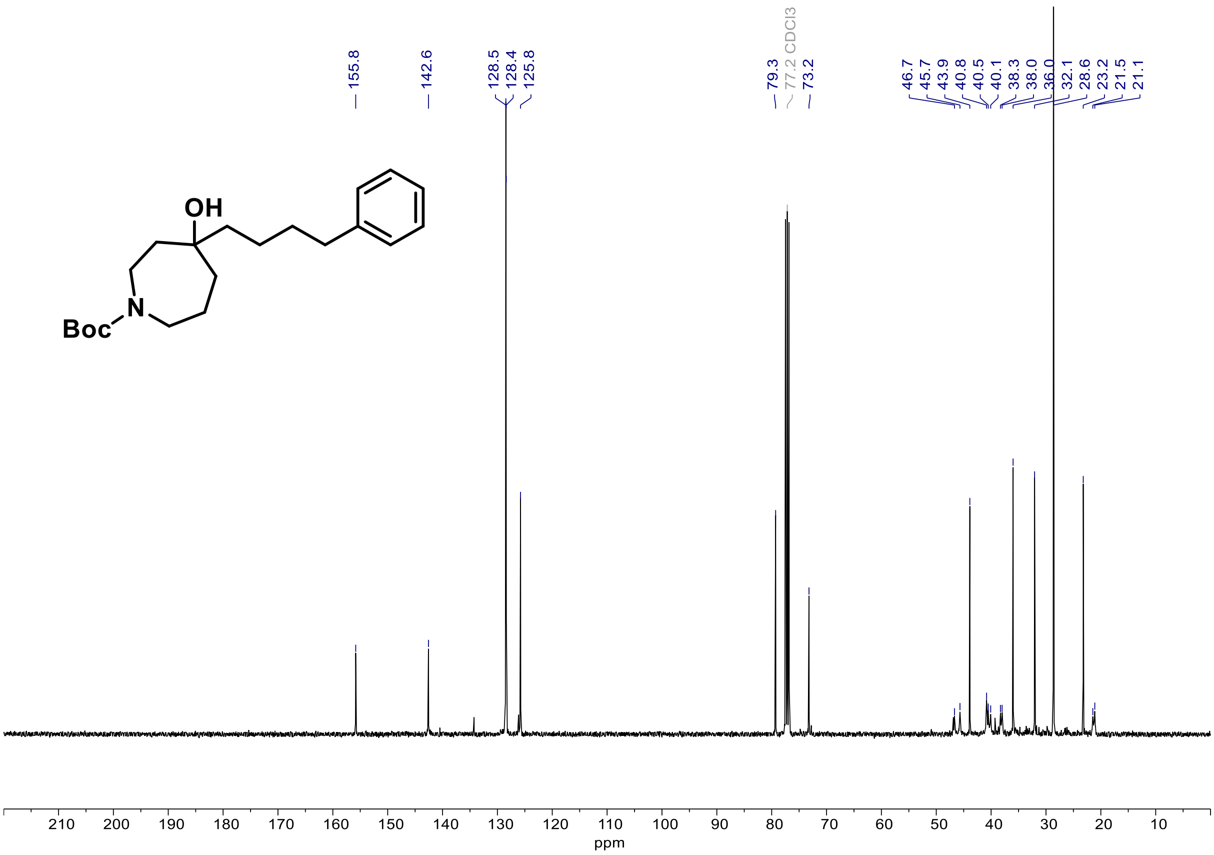

38 – ^{1}H NMR (400 MHz, $CDCl_3$)

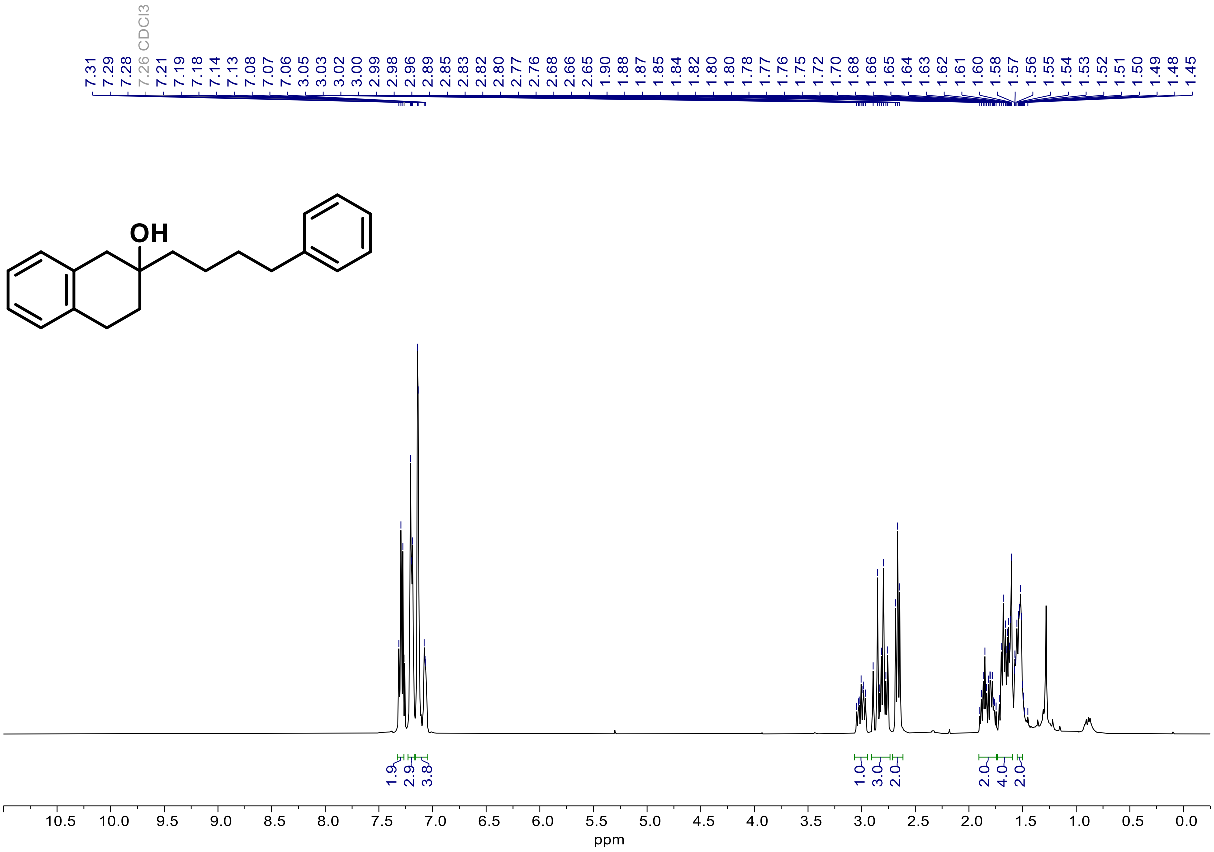

38 – ^{13}C NMR (101 MHz, $CDCl_3$)

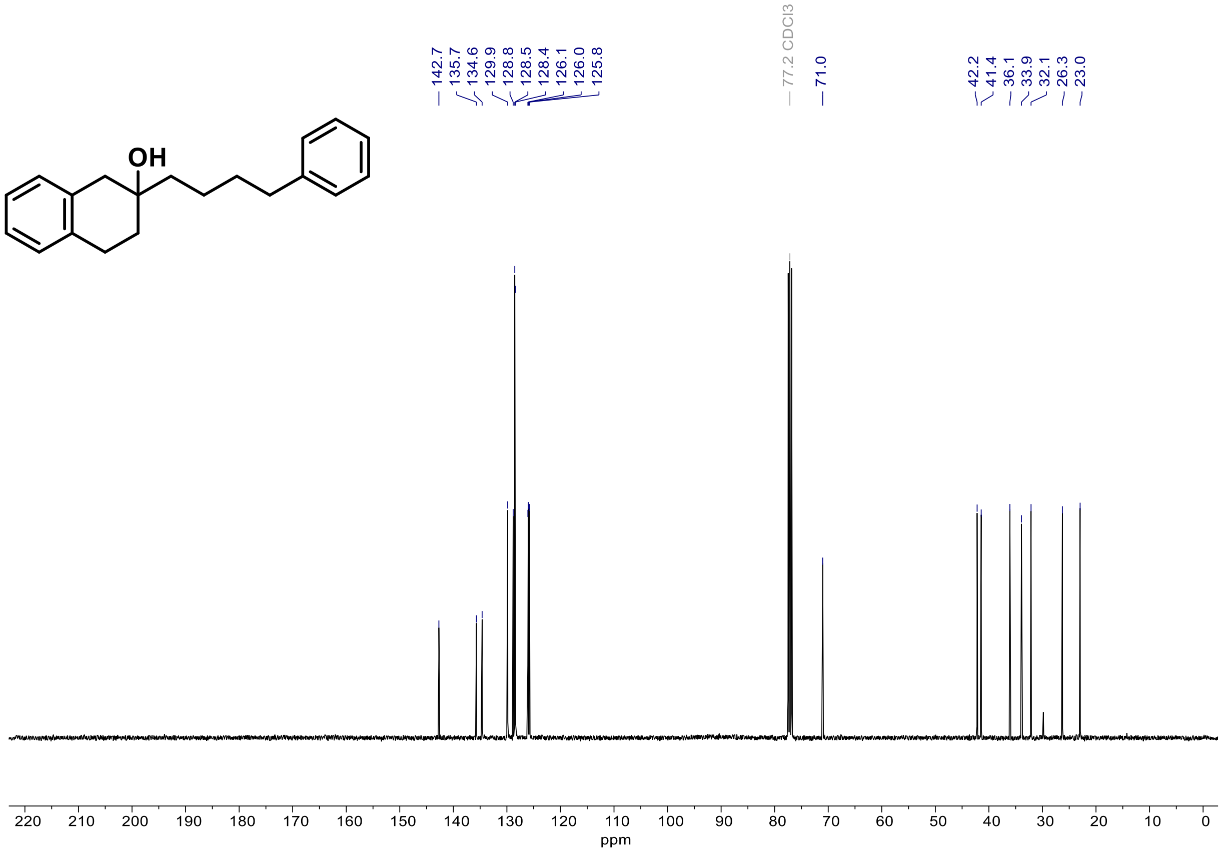

39 – ^{1}H NMR (400 MHz, $CDCl_3$)

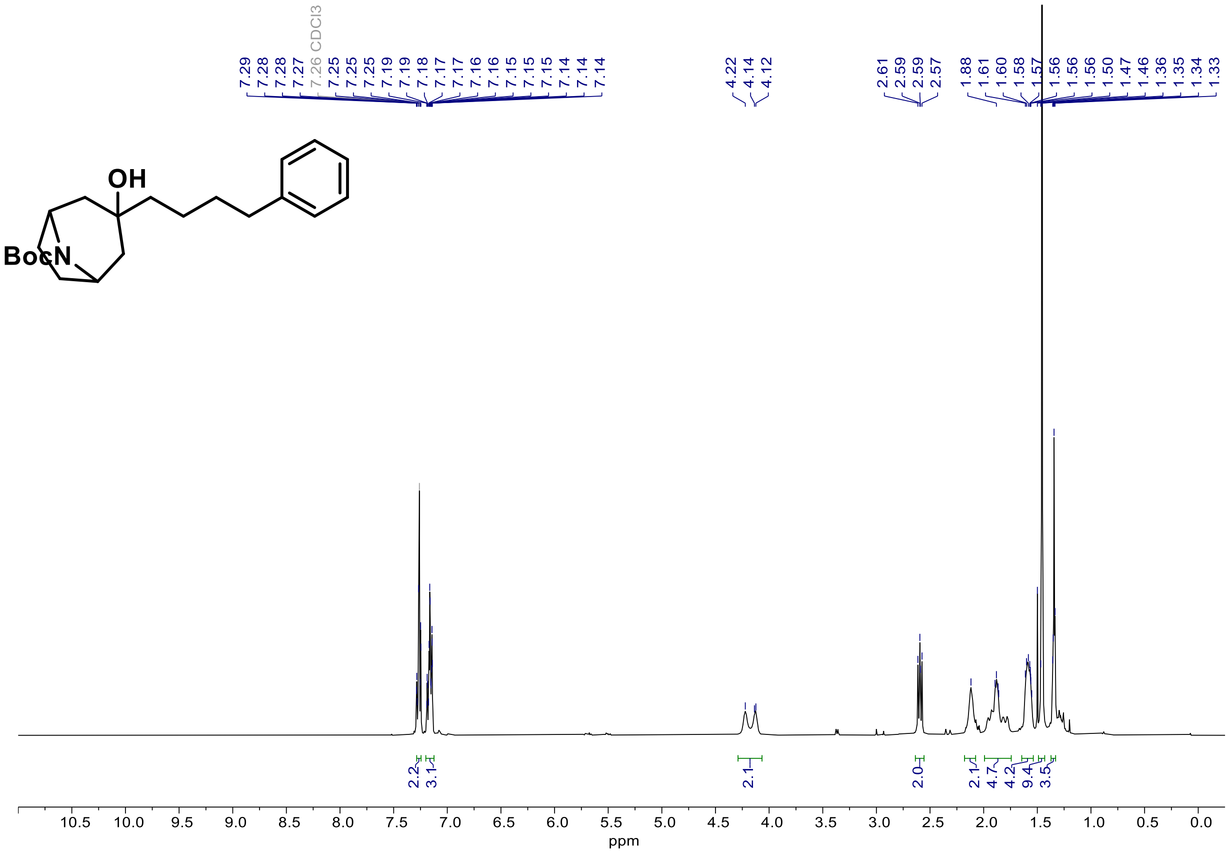

39 – ^{13}C NMR (101 MHz, $CDCl_3$)

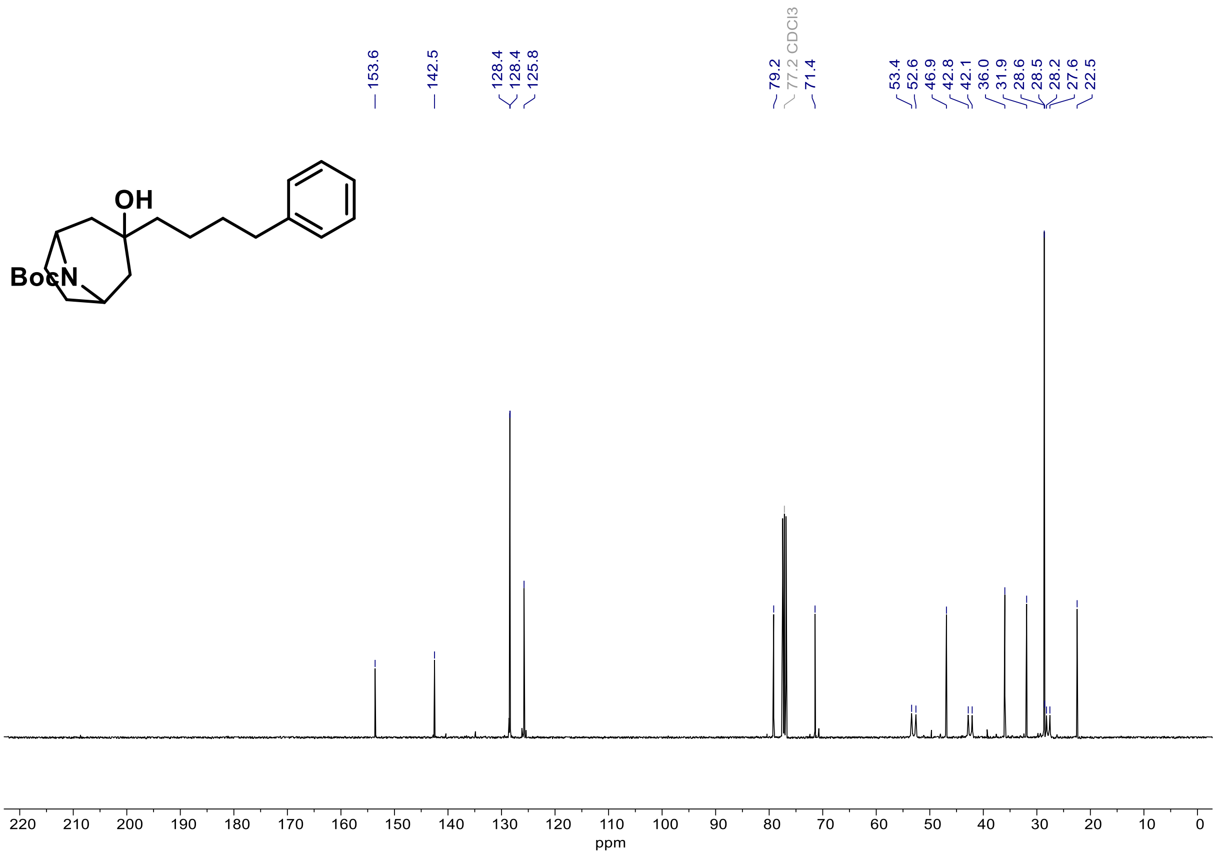

40 – ^{1}H NMR (400 MHz, $CDCl_3$)

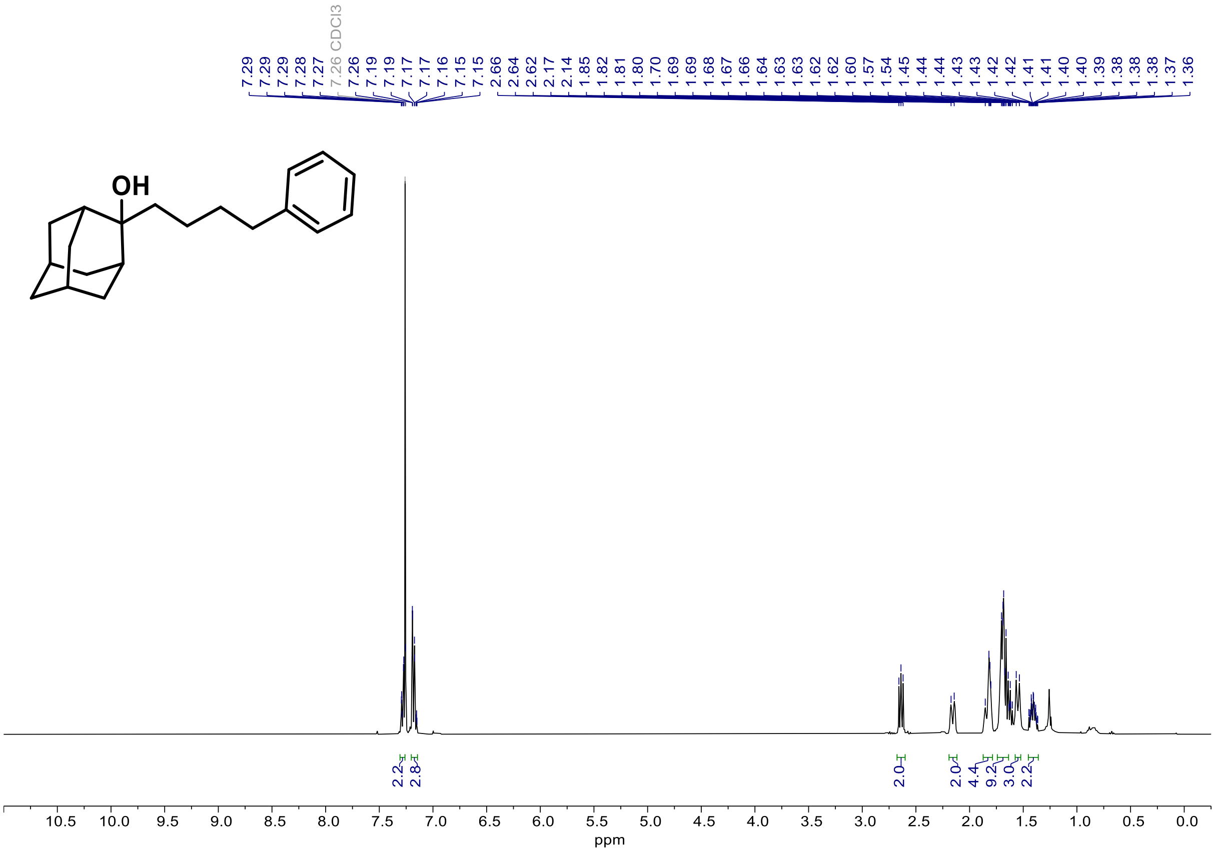

40 – ^{13}C NMR (101 MHz, $CDCl_3$)

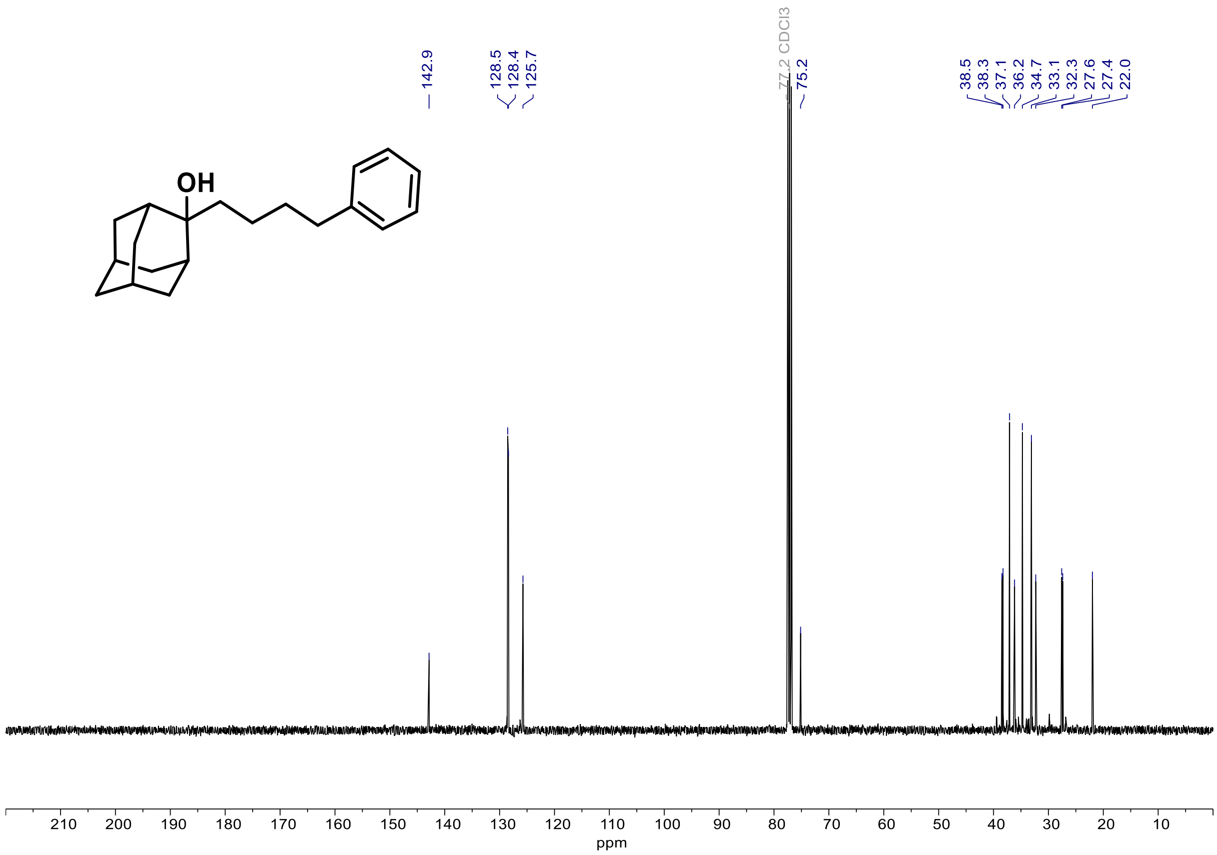

41 – ^{1}H NMR (400 MHz, $CDCl_3$)

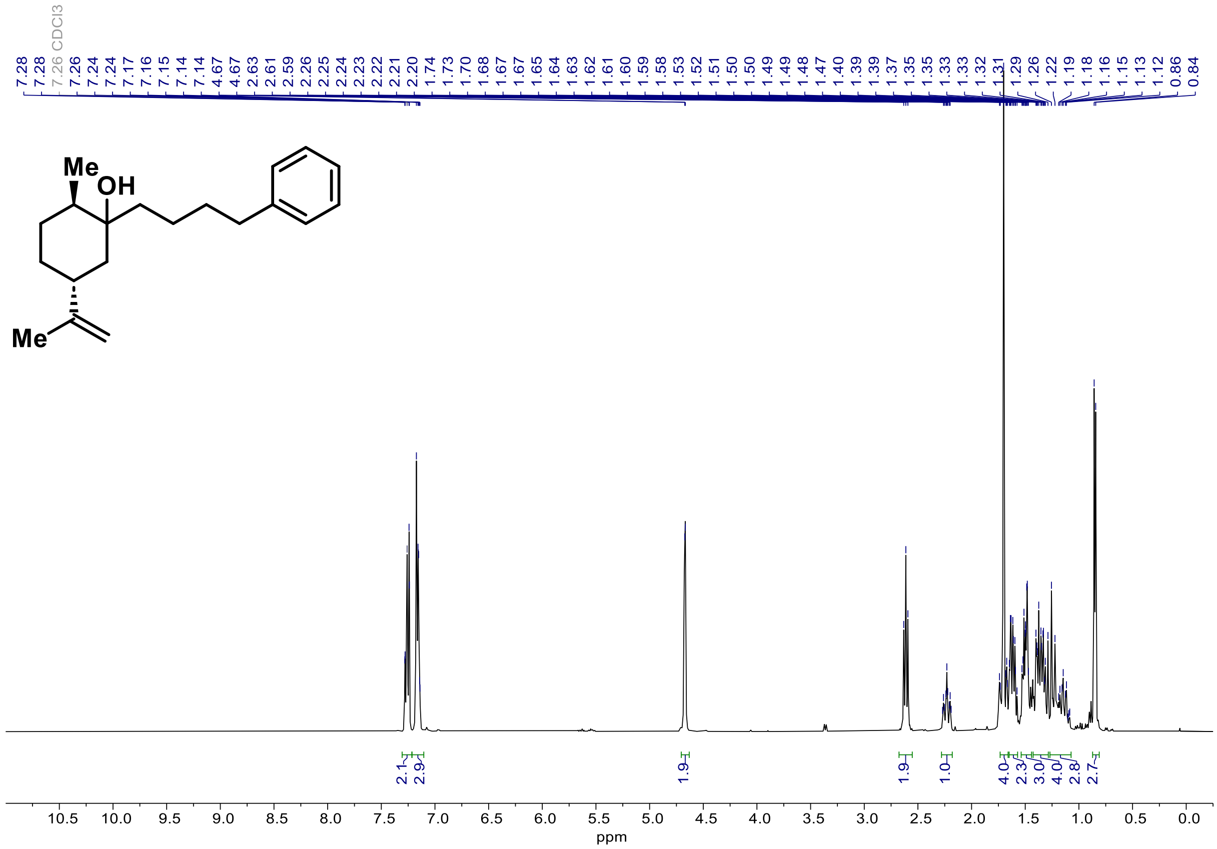

41 – ^{13}C NMR (101 MHz, $CDCl_3$)

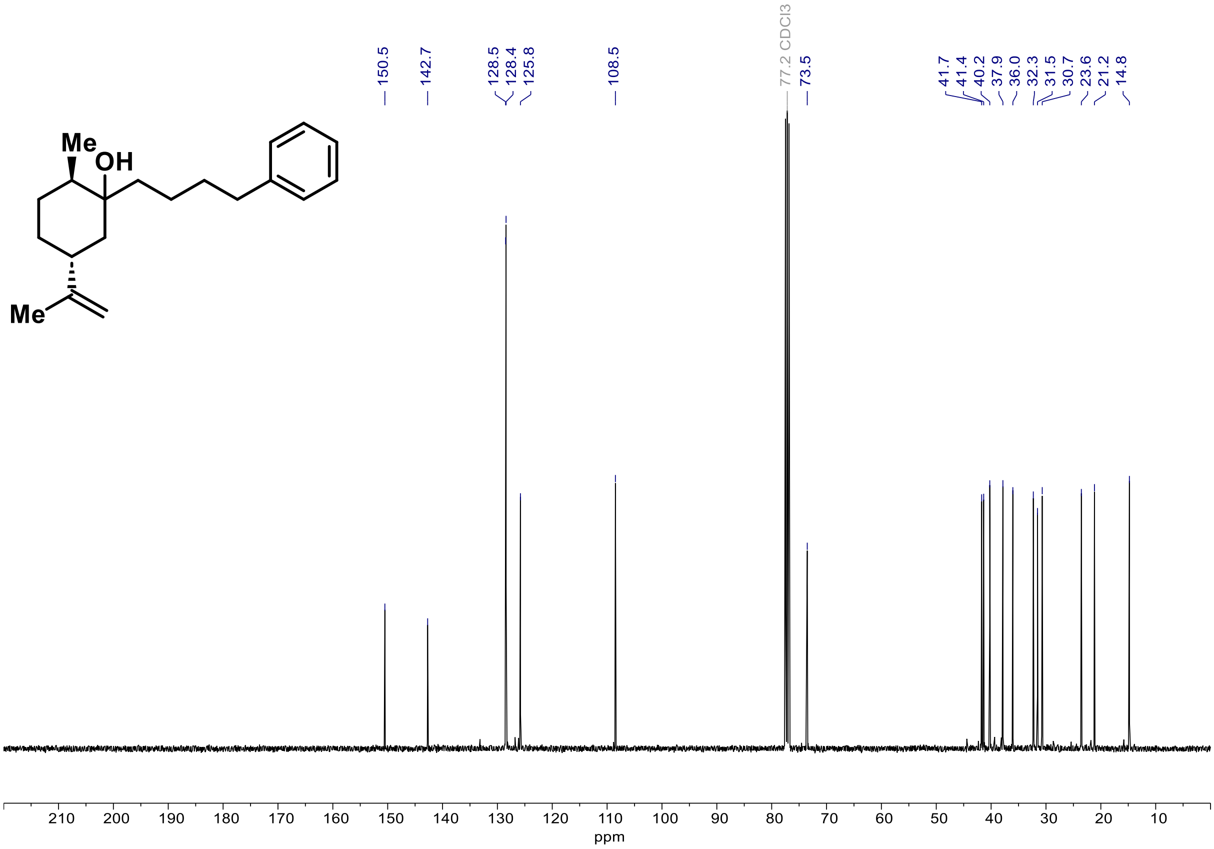

42 – ^{1}H NMR (400 MHz, $CDCl_3$)

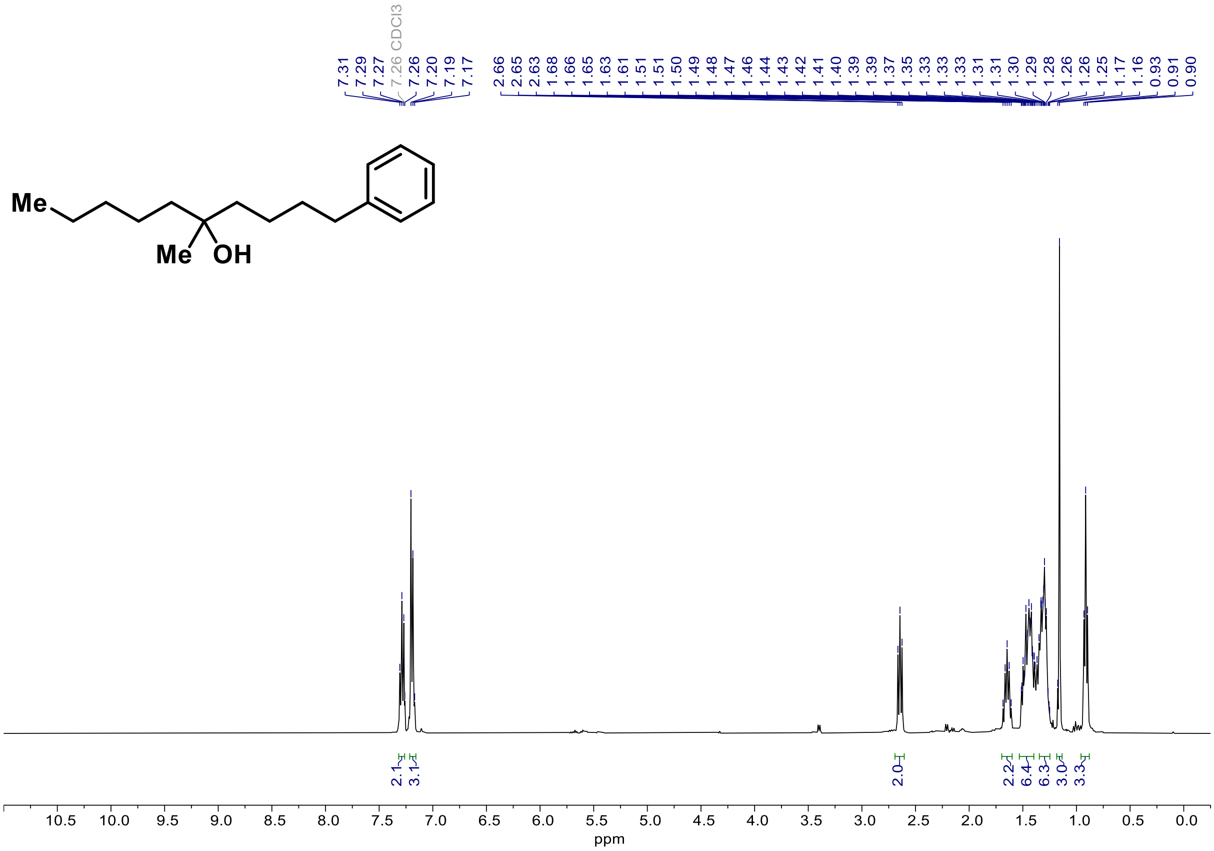

42 – ^{13}C NMR (101 MHz, $CDCl_3$)

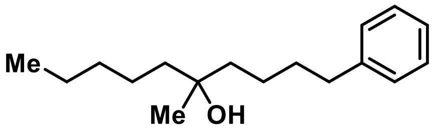

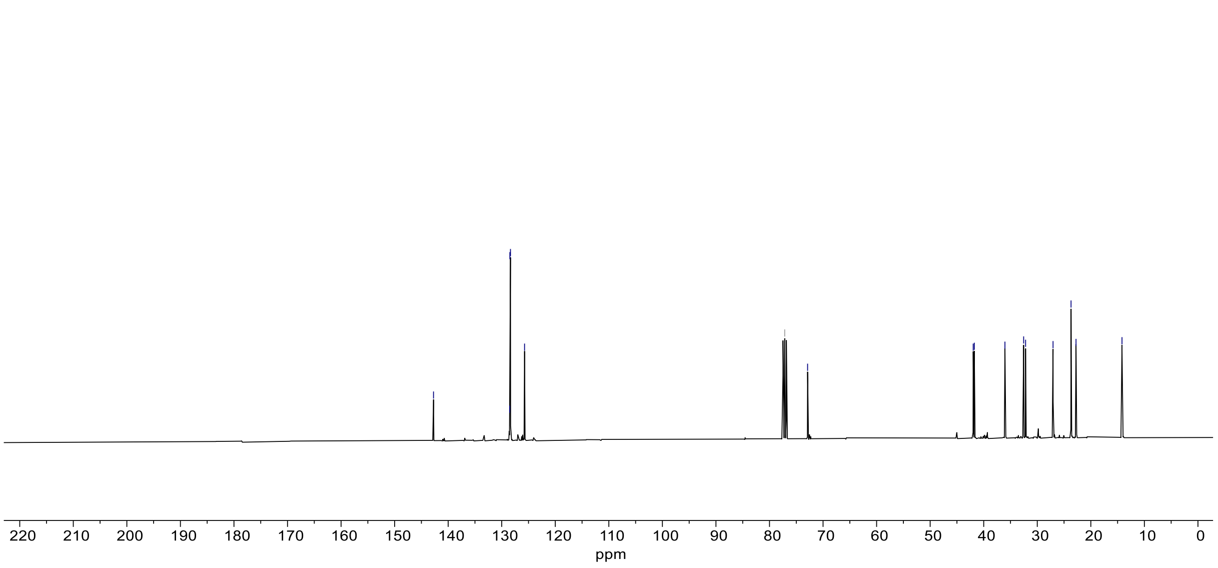

43 – ^{1}H NMR (400 MHz, $CDCl_3$)

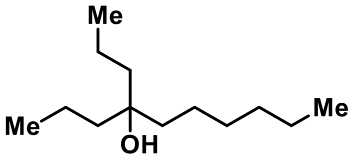

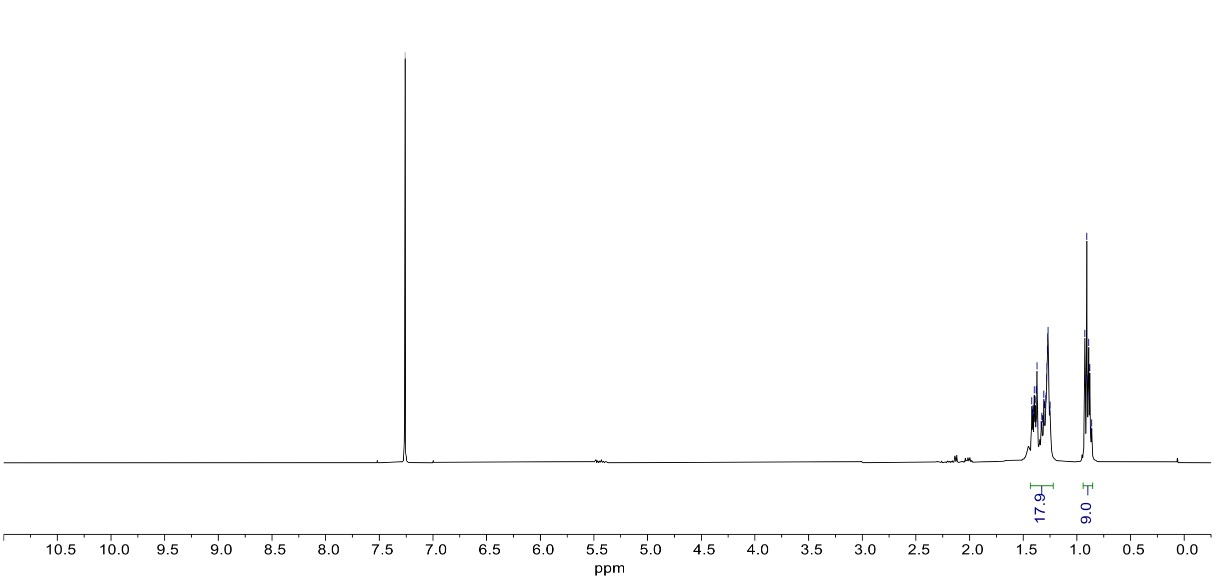

43 – ^{13}C NMR (101 MHz, $CDCl_3$)

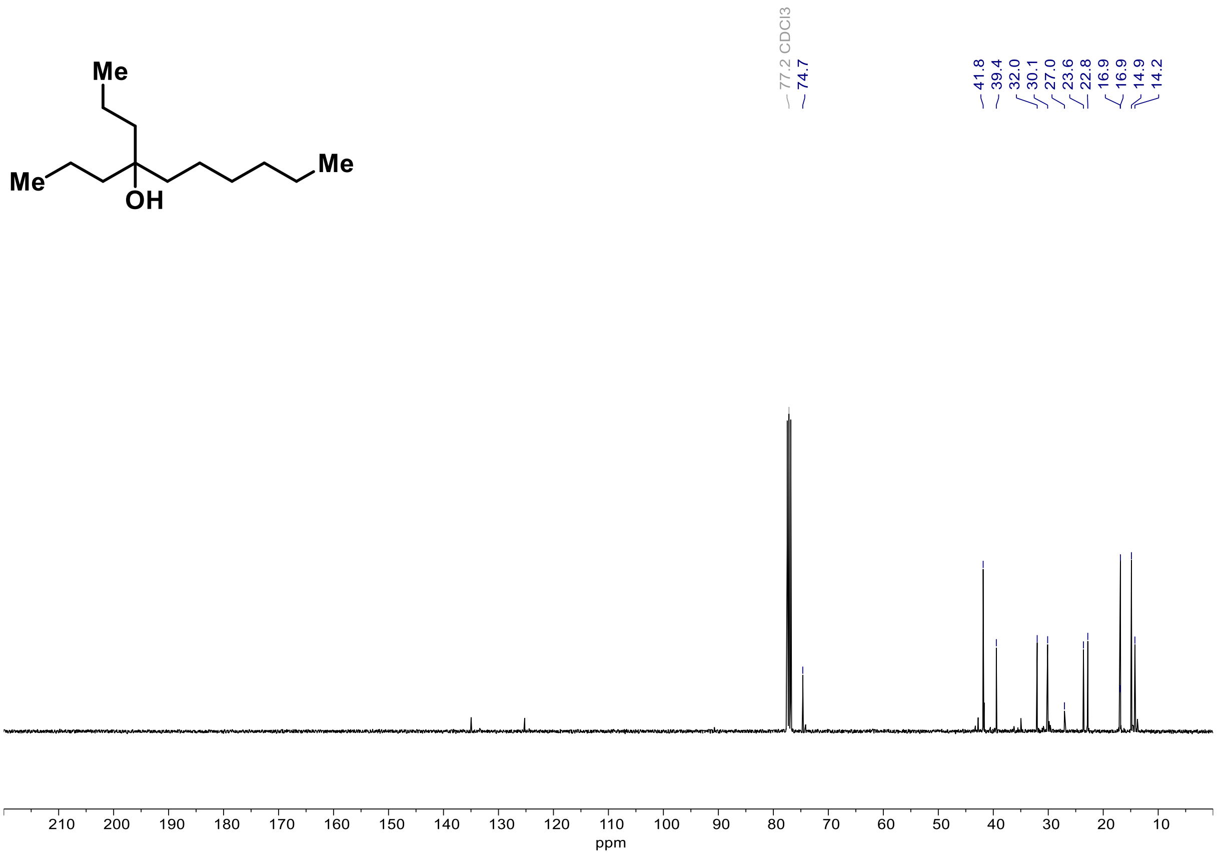

44 – ^{1}H NMR (400 MHz, $CDCl_3$)

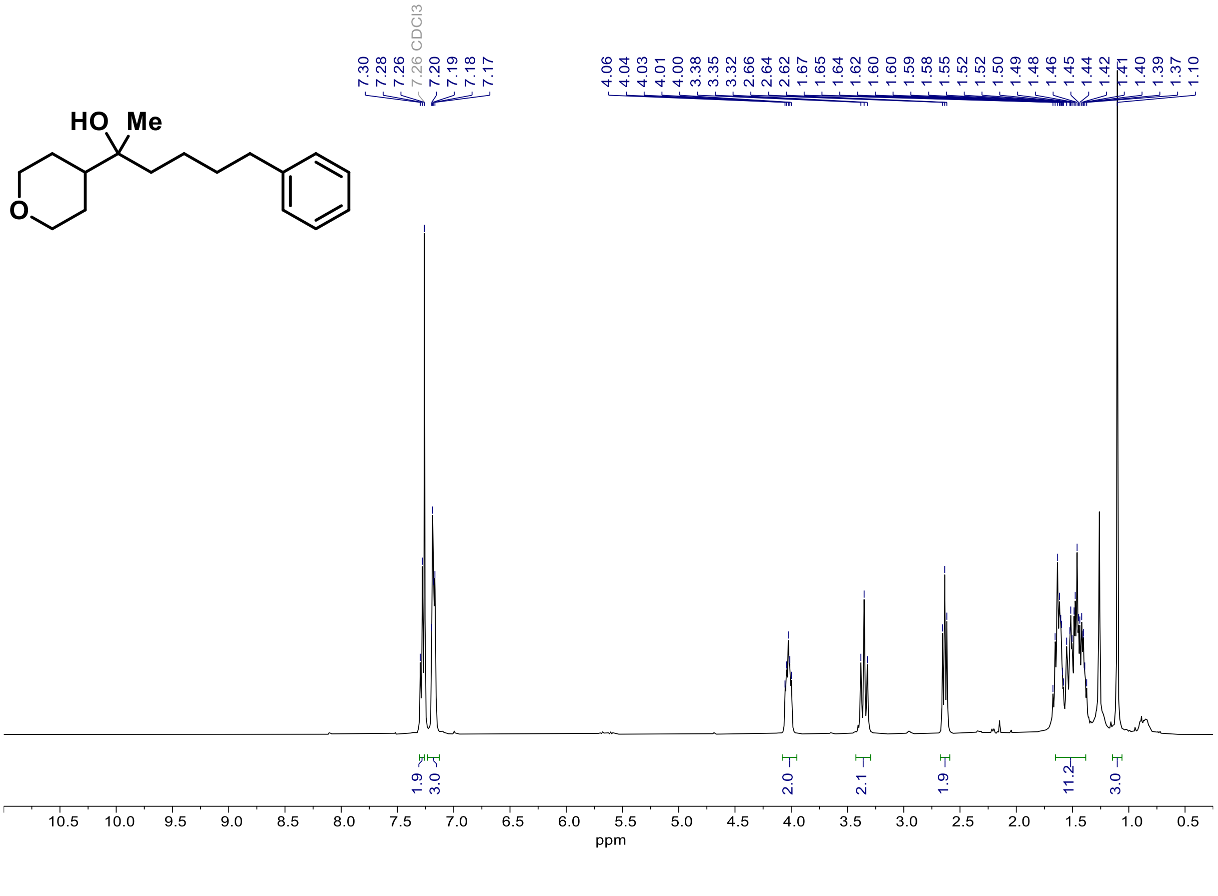

44 – ^{13}C NMR (101 MHz, $CDCl_3$)

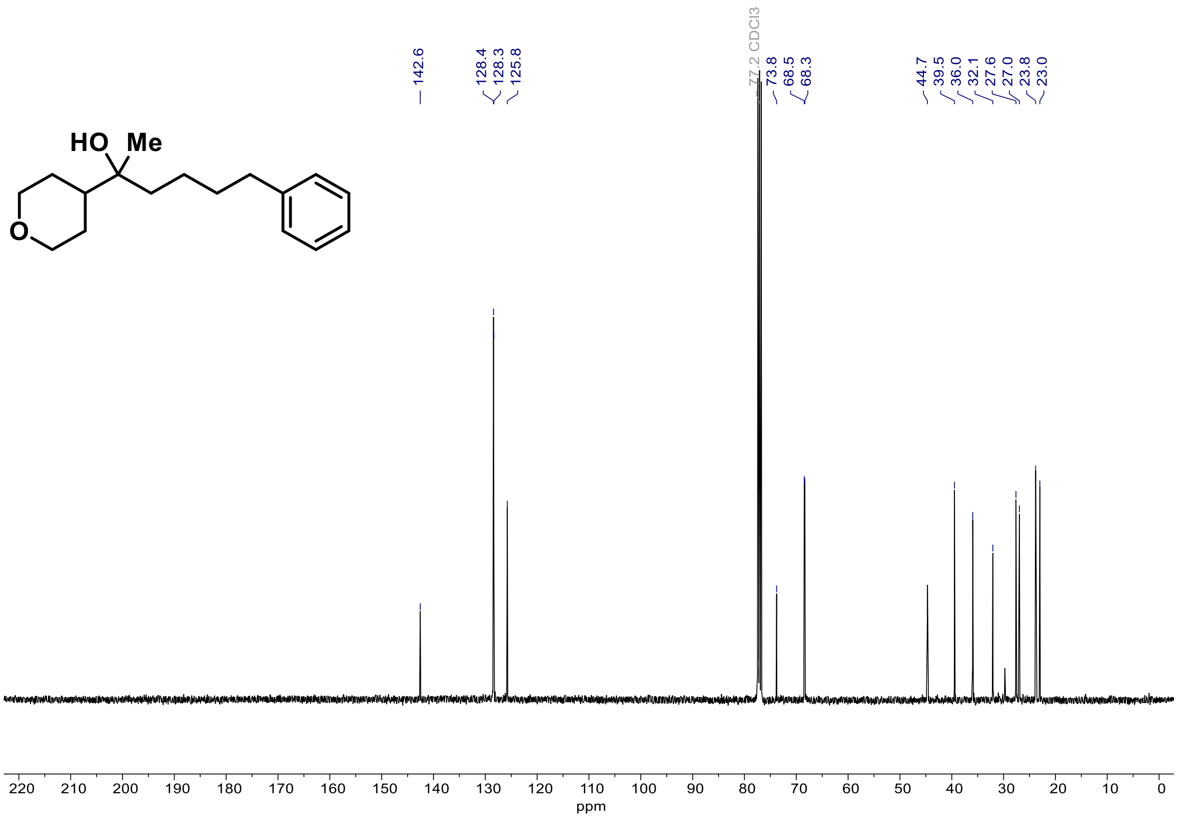

45 – ^{1}H NMR (400 MHz, $CDCl_3$)

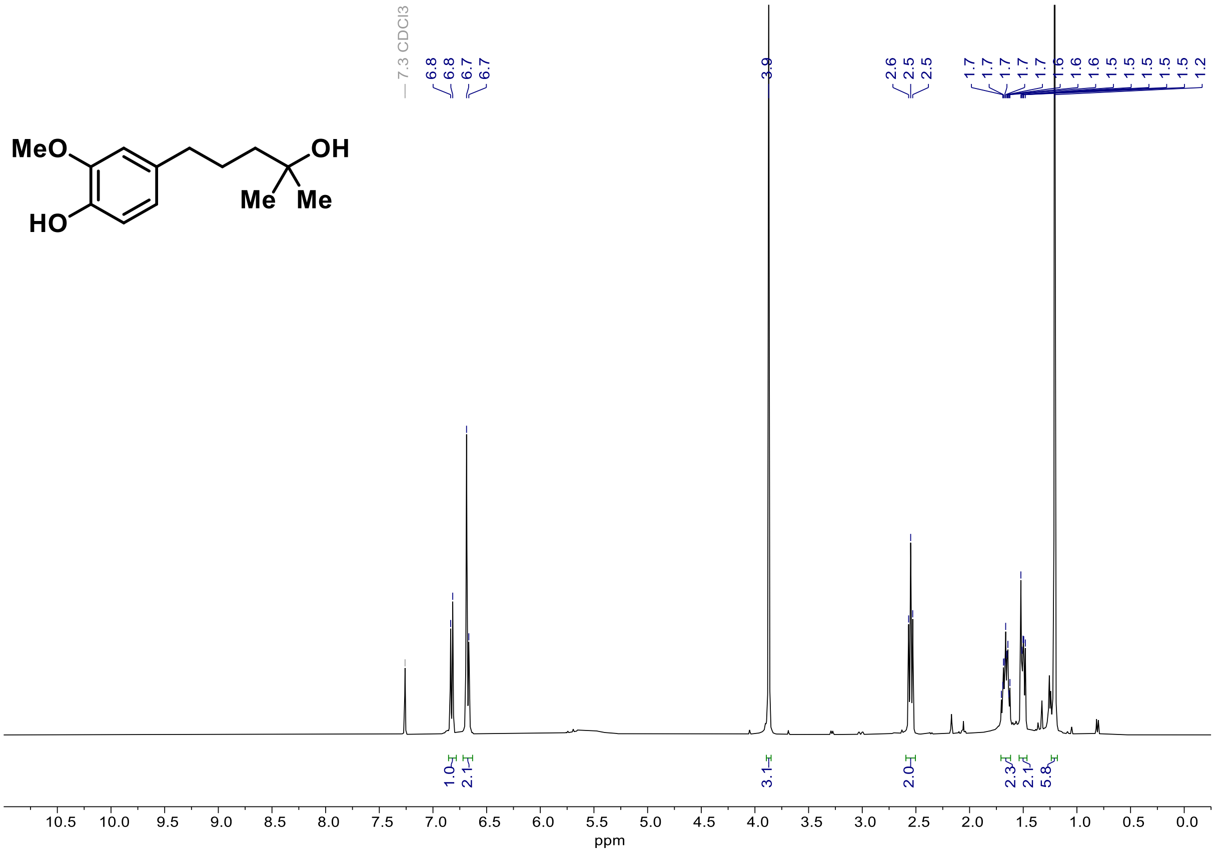

45 – ^{13}C NMR (101 MHz, $CDCl_3$)

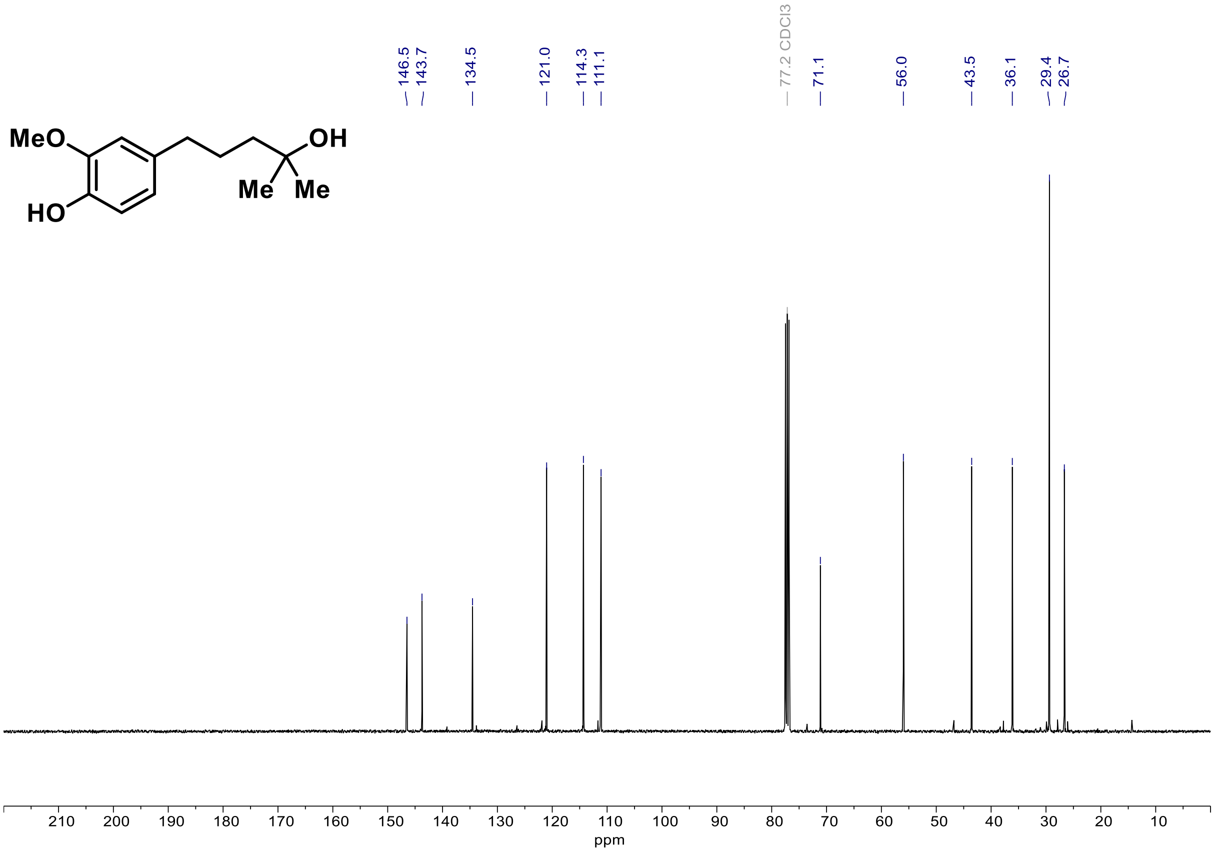

46 – ^{1}H NMR (400 MHz, $CDCl_3$)

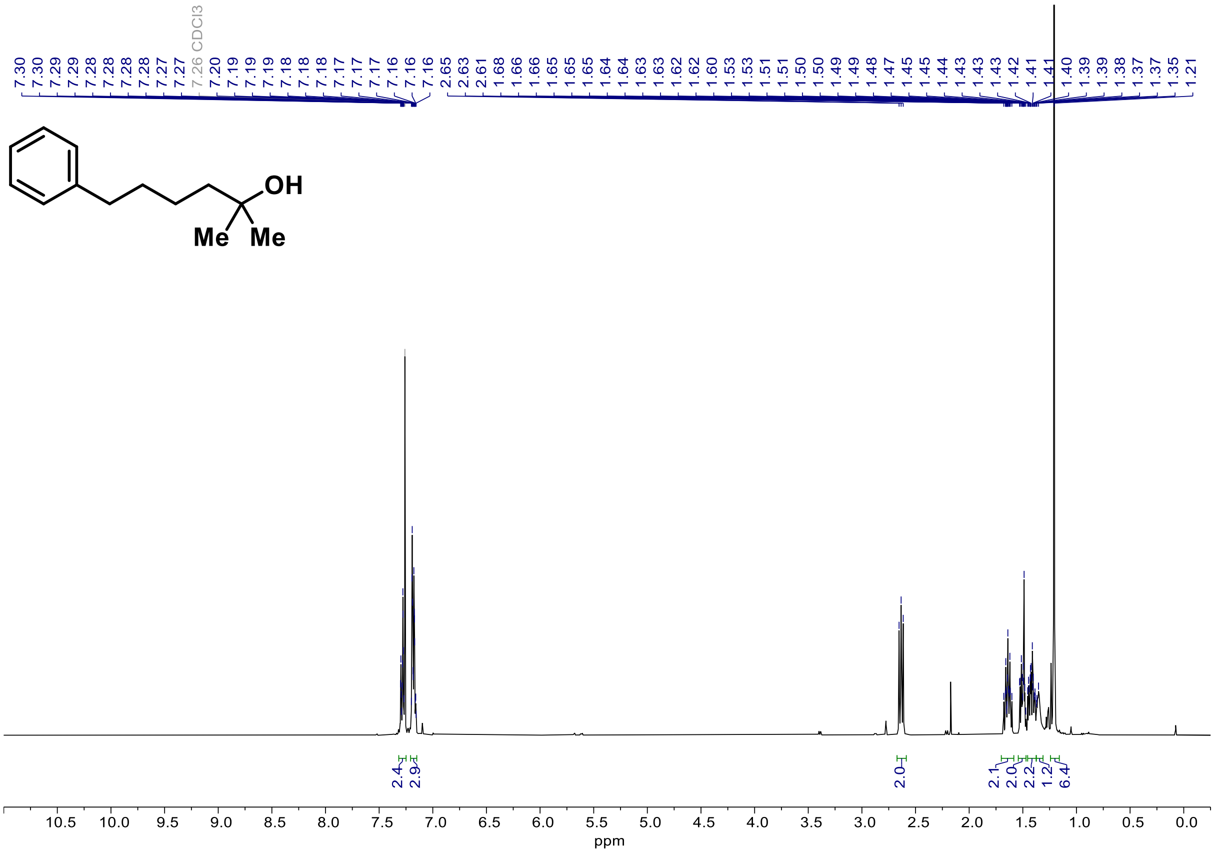

46 – ^{13}C NMR (101 MHz, $CDCl_3$)

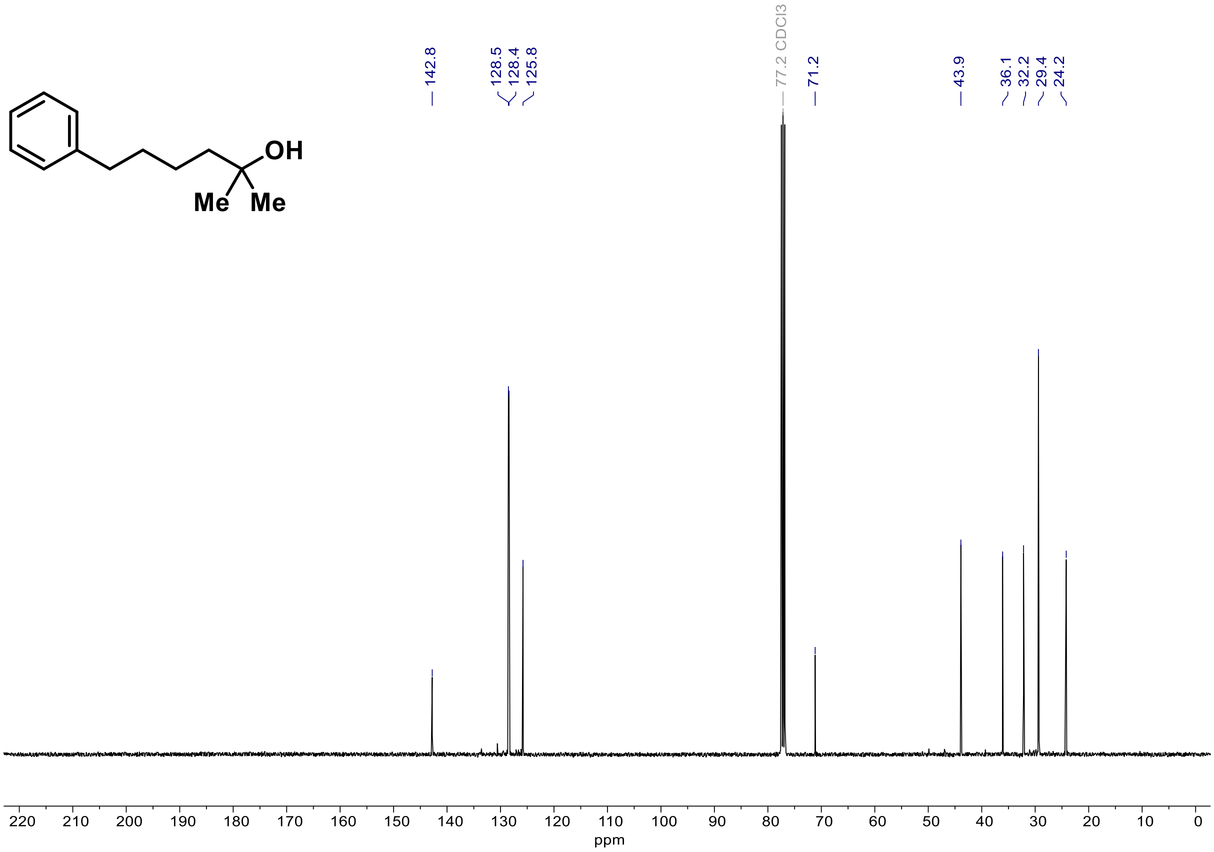

47 – ^{1}H NMR (400 MHz, $CDCl_3$)

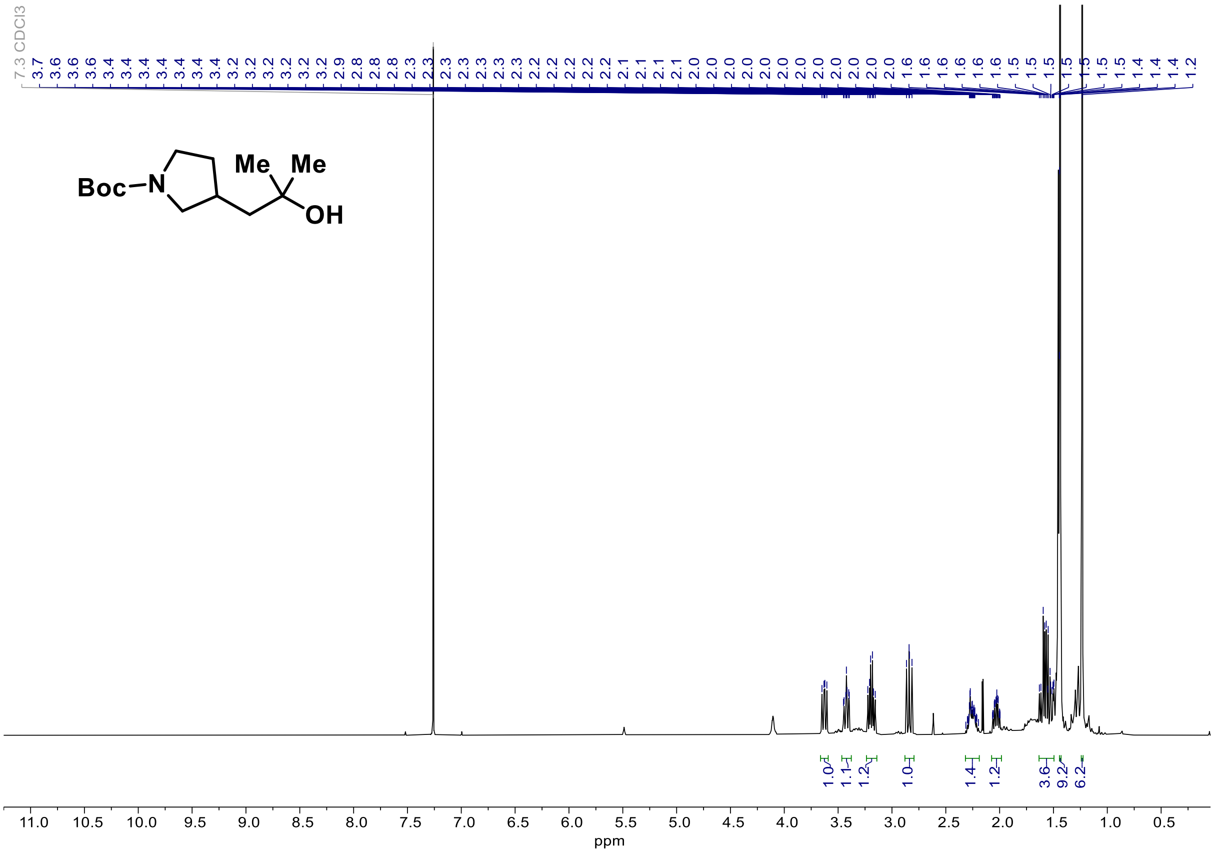

47 – ^{13}C NMR (101 MHz, CDCl$_3$)

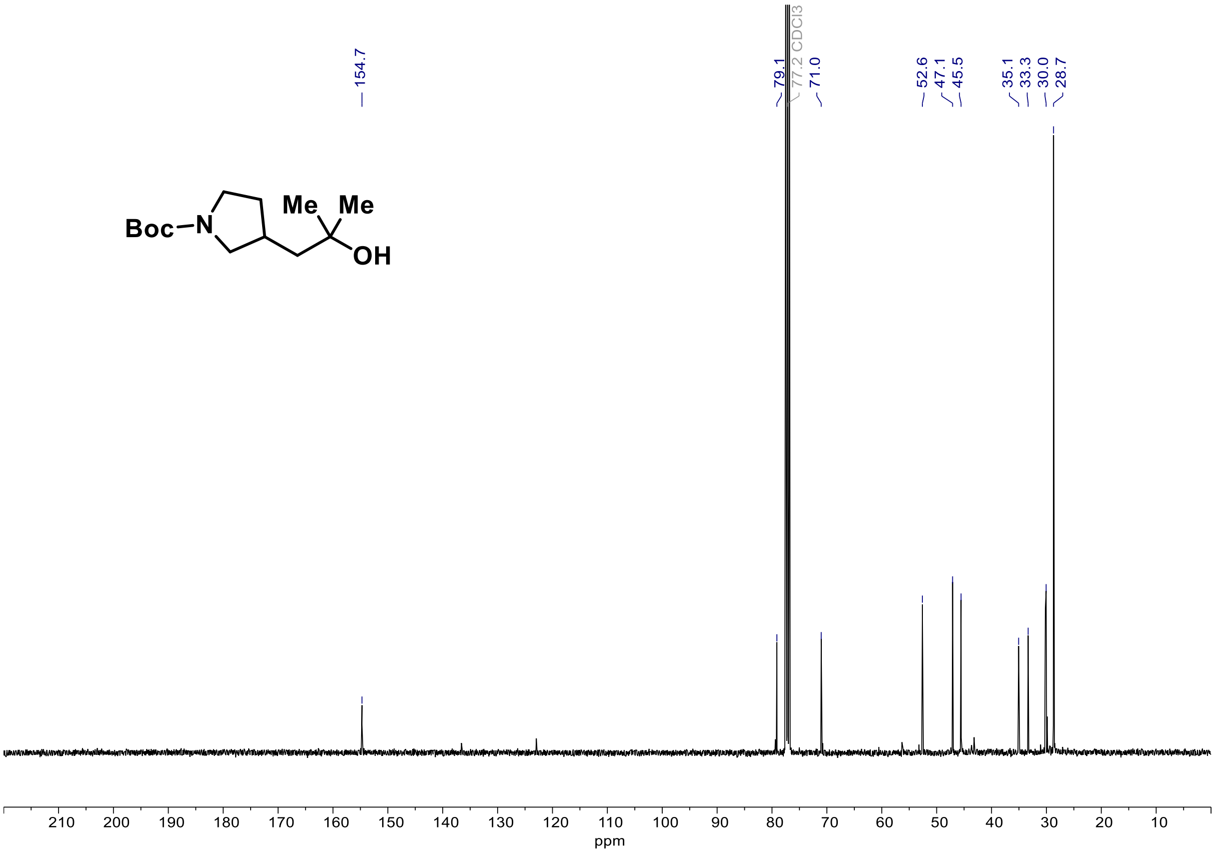

48 – ^{1}H NMR (400 MHz, CDCl$_3$)

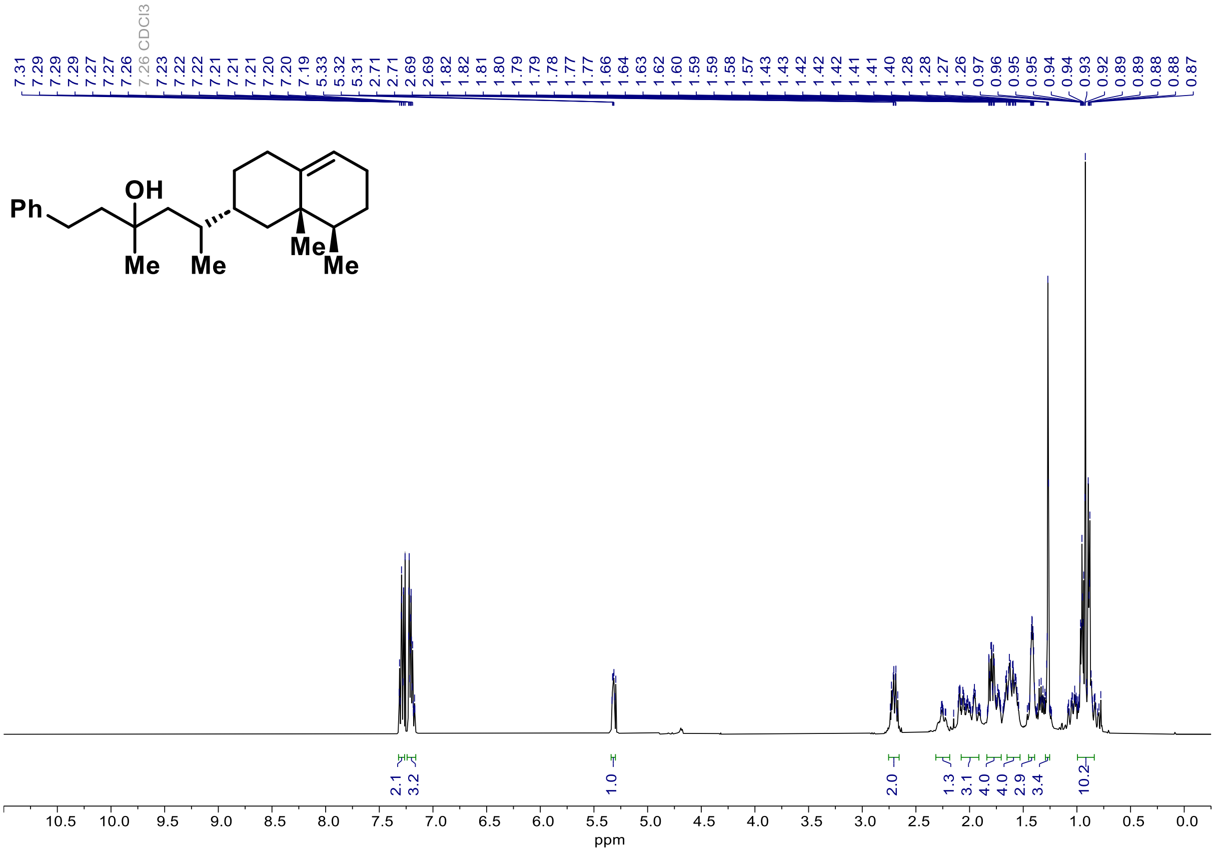

48 – ^{13}C NMR (101 MHz, $CDCl_3$)

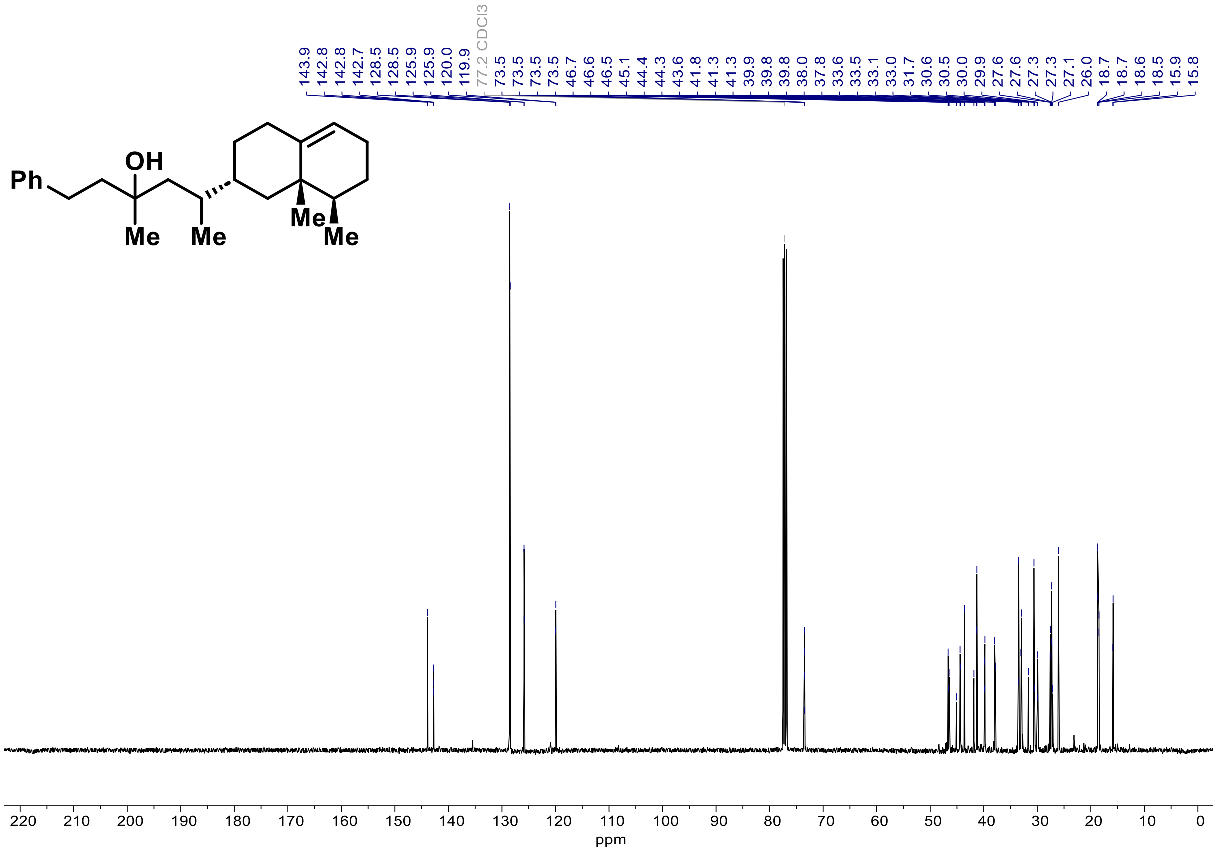

49 – ^{1}H NMR (400 MHz, $CDCl_3$)

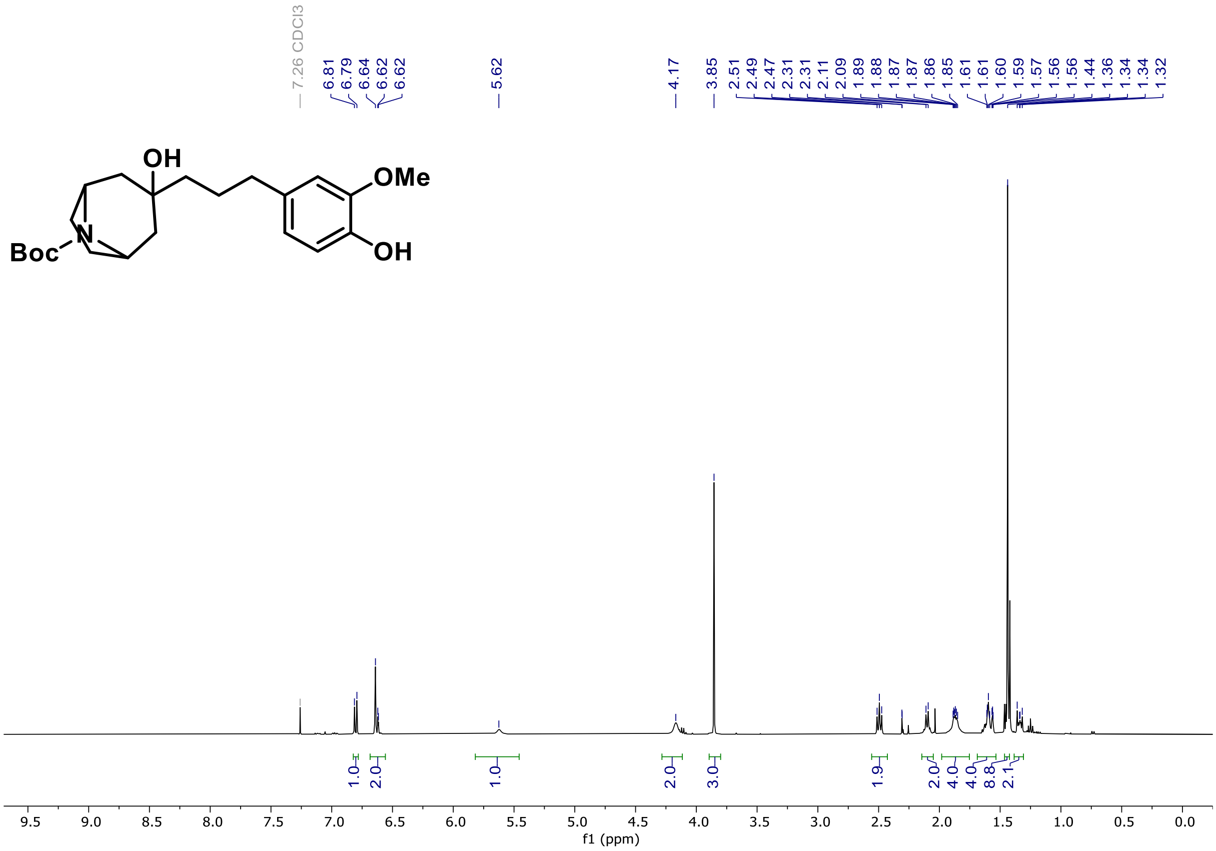

49 – ^{13}C NMR (101 MHz, $CDCl_3$)

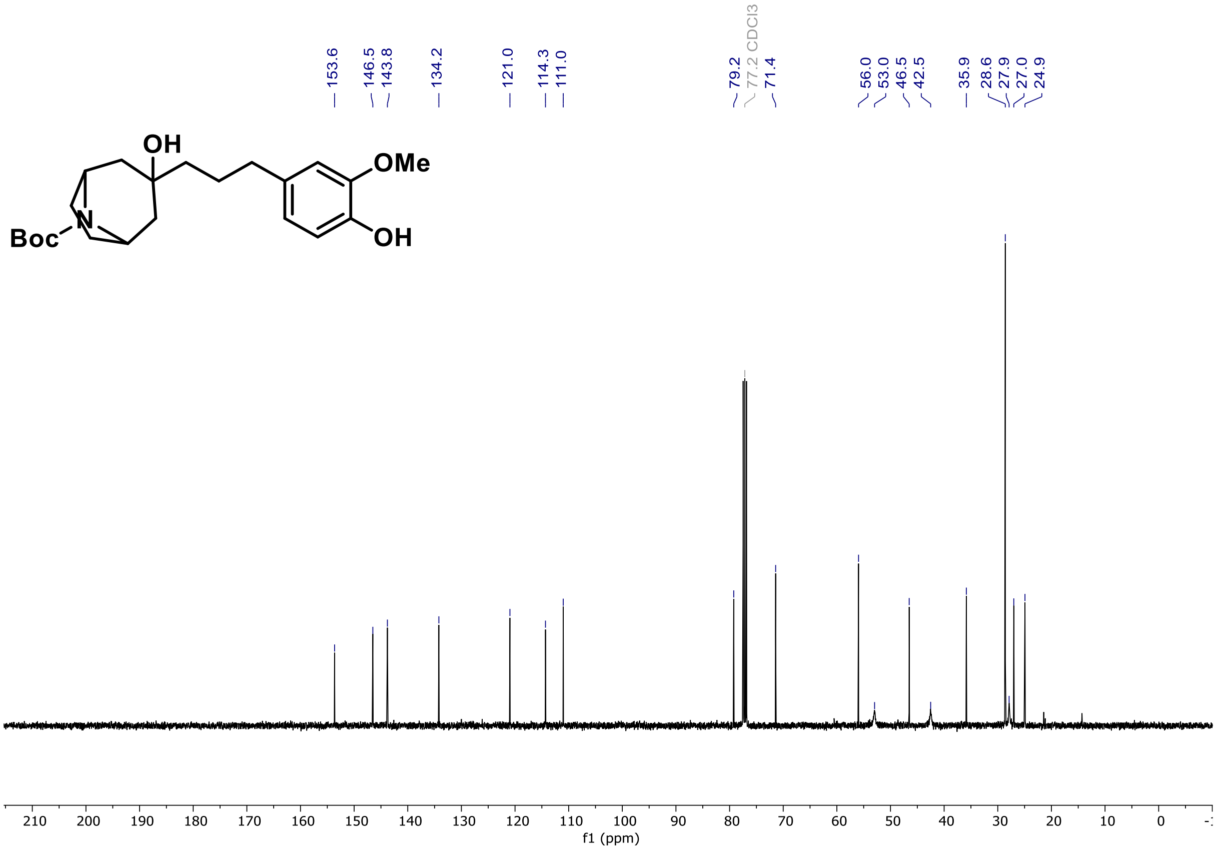

50 – ^{1}H NMR (400 MHz, $CDCl_3$)

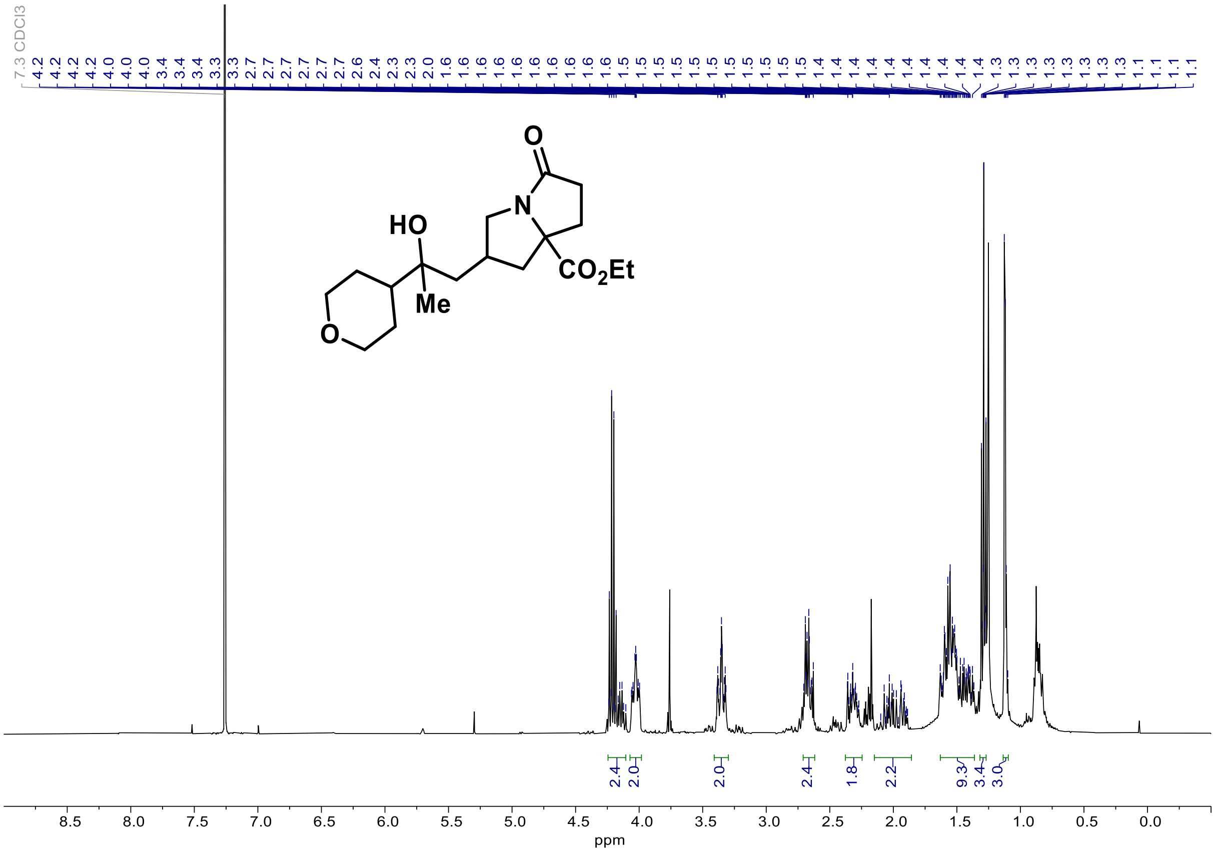

50 – ^{13}C NMR (101 MHz, $CDCl_3$)

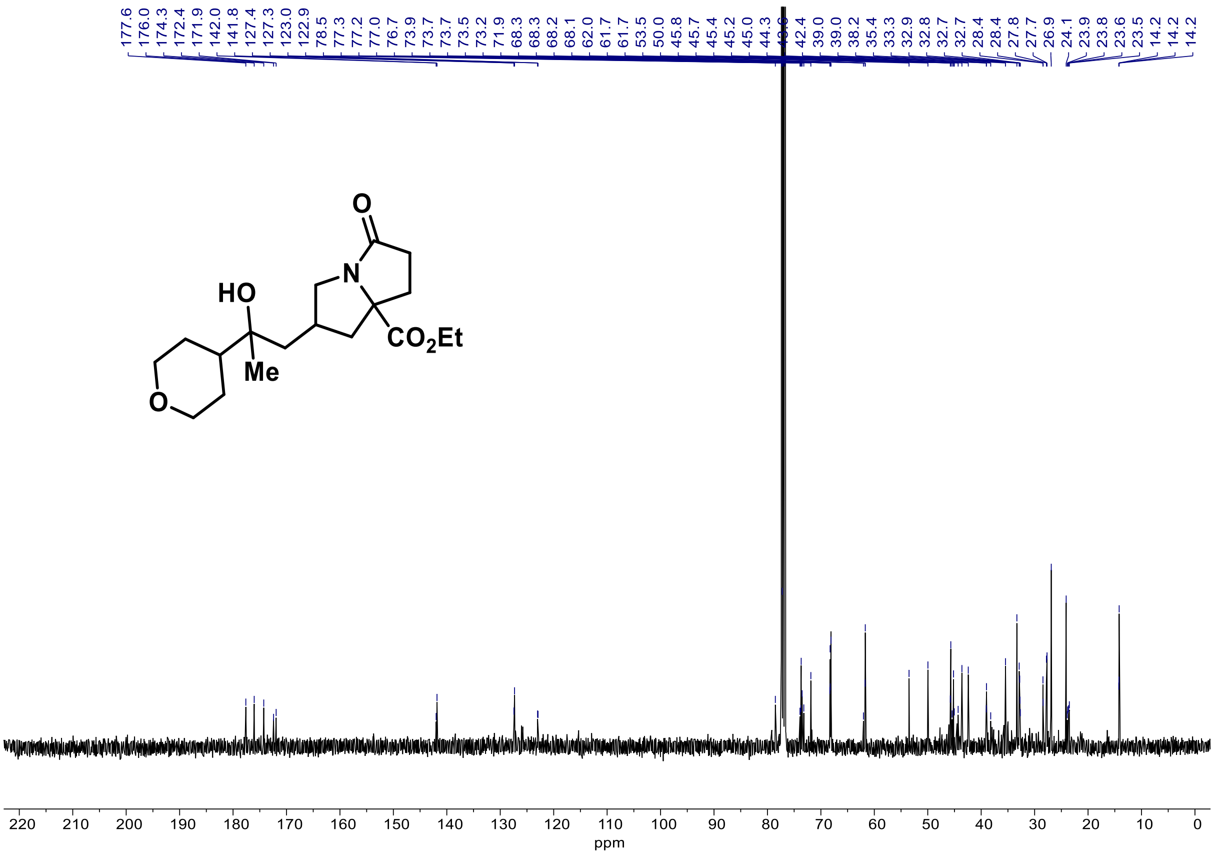

51 – ^{1}H NMR (400 MHz, $CDCl_3$)

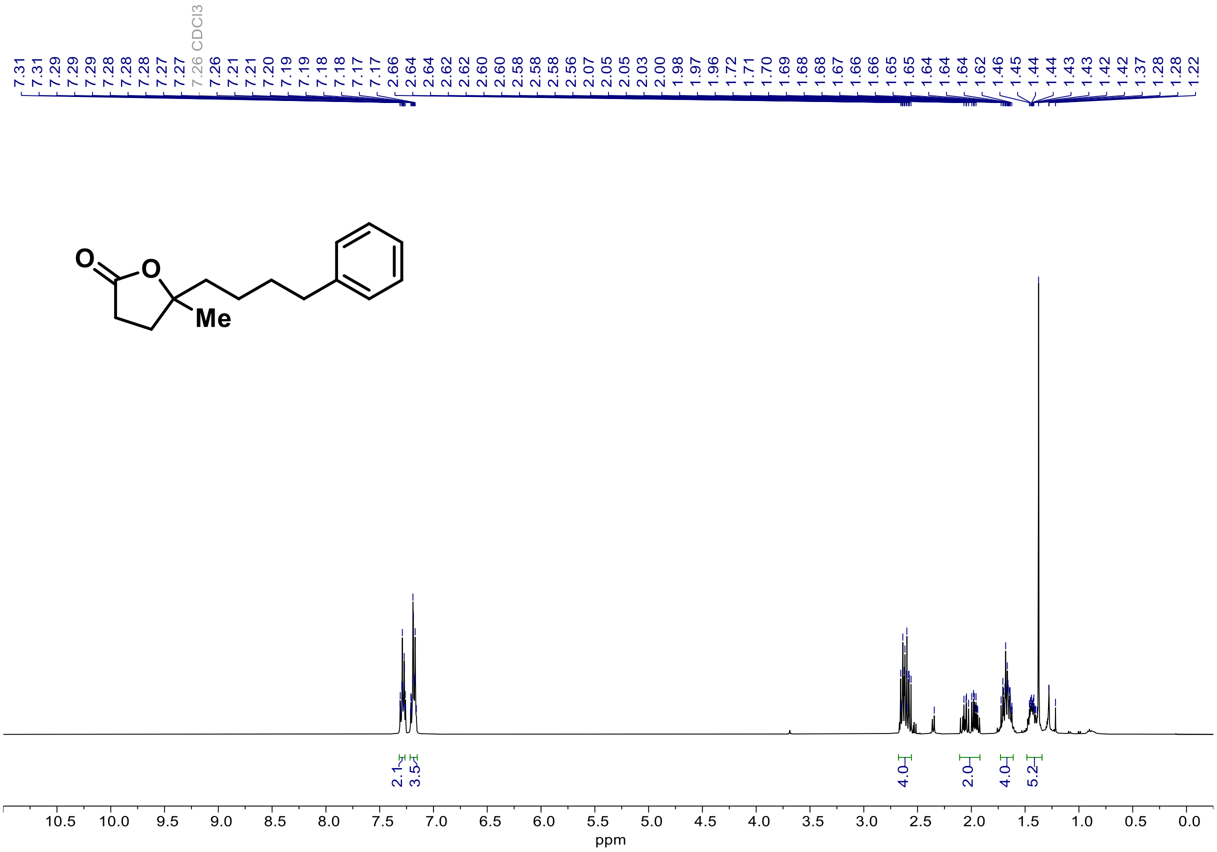

51 – ^{13}C NMR (101 MHz, $CDCl_3$)

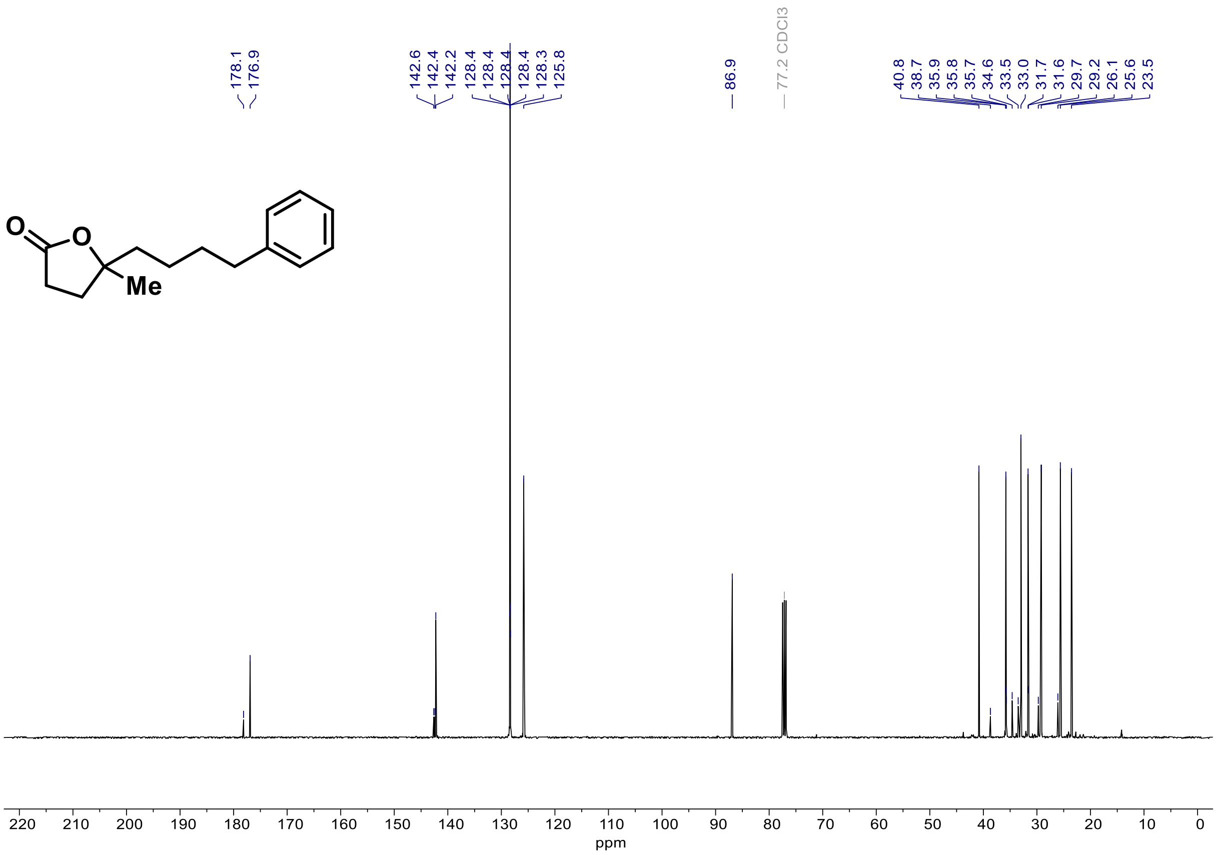

52 – ^{1}H NMR (400 MHz, MeOH-*d4*)

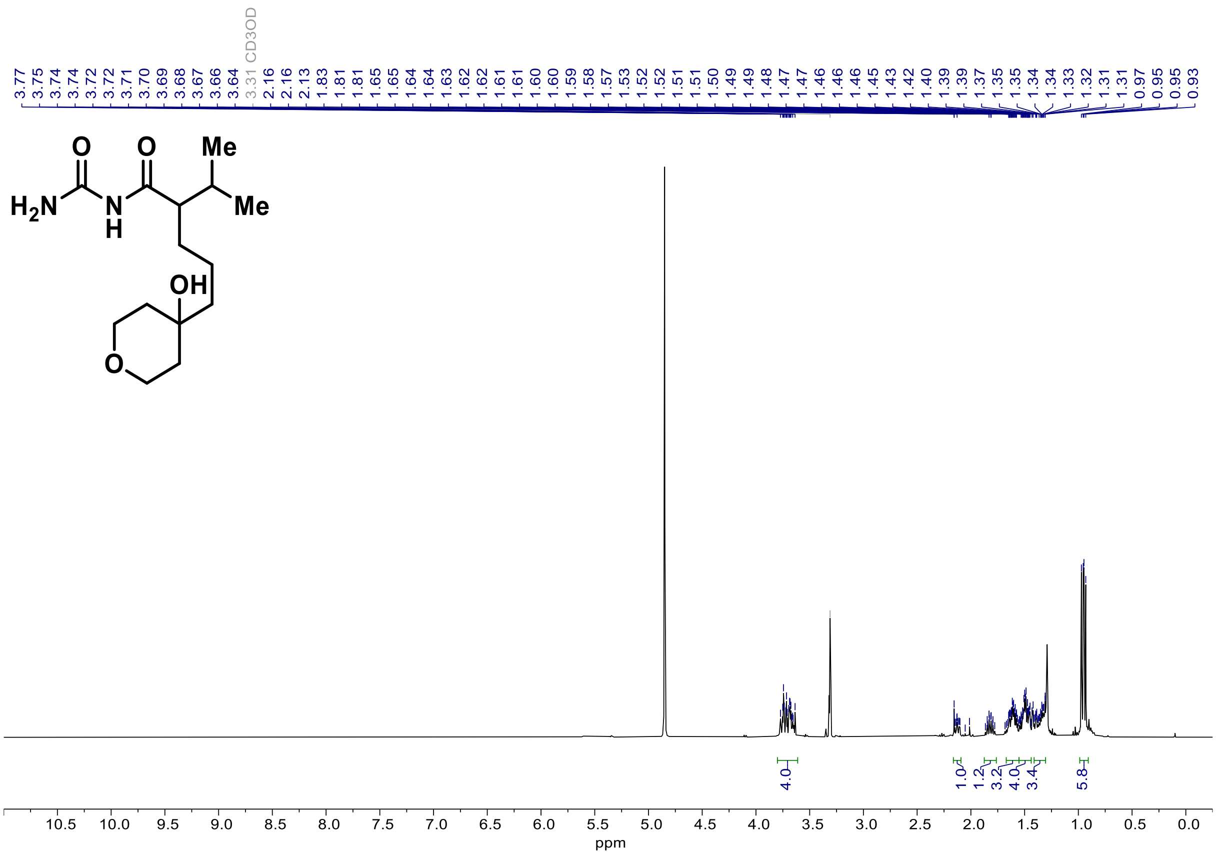

52 – ^{13}C NMR (101 MHz, MeOH-*d4*)

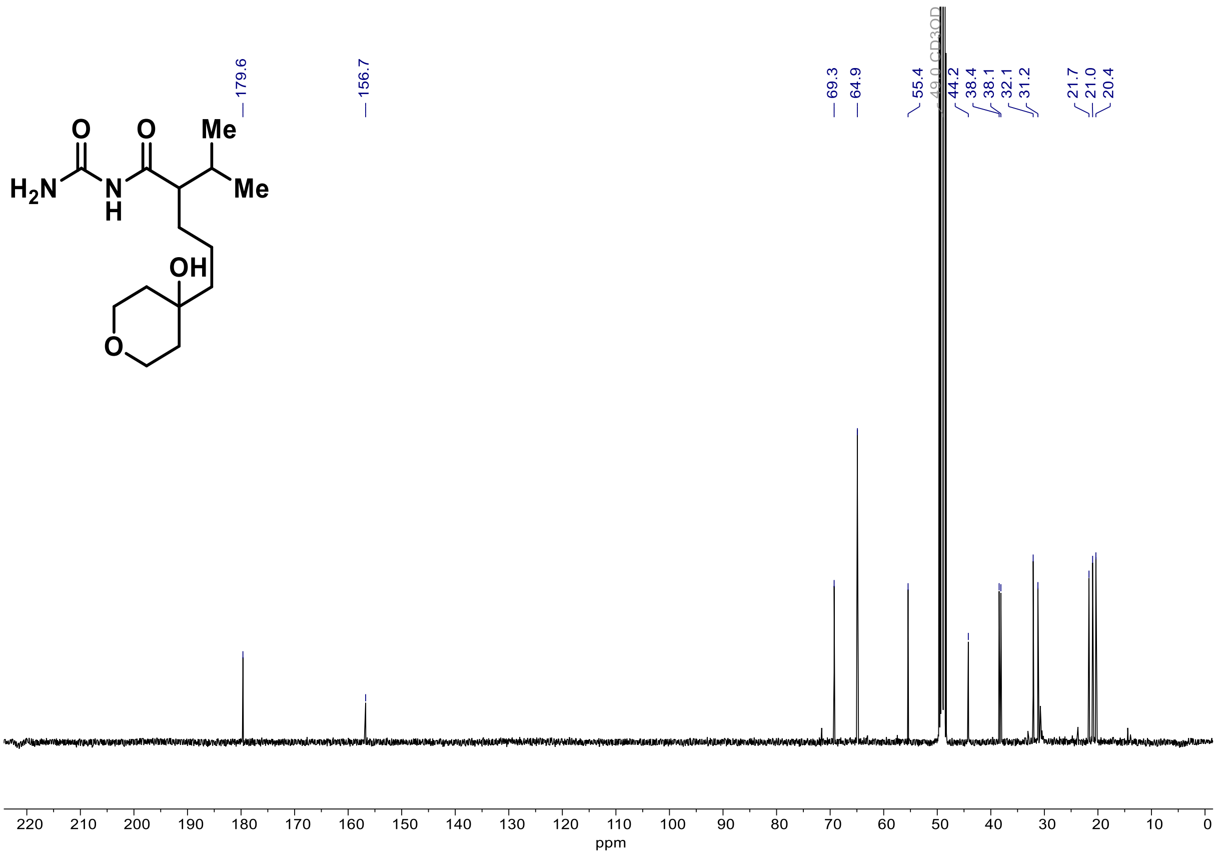

53 – ^{1}H NMR (400 MHz, CDCl$_3$)

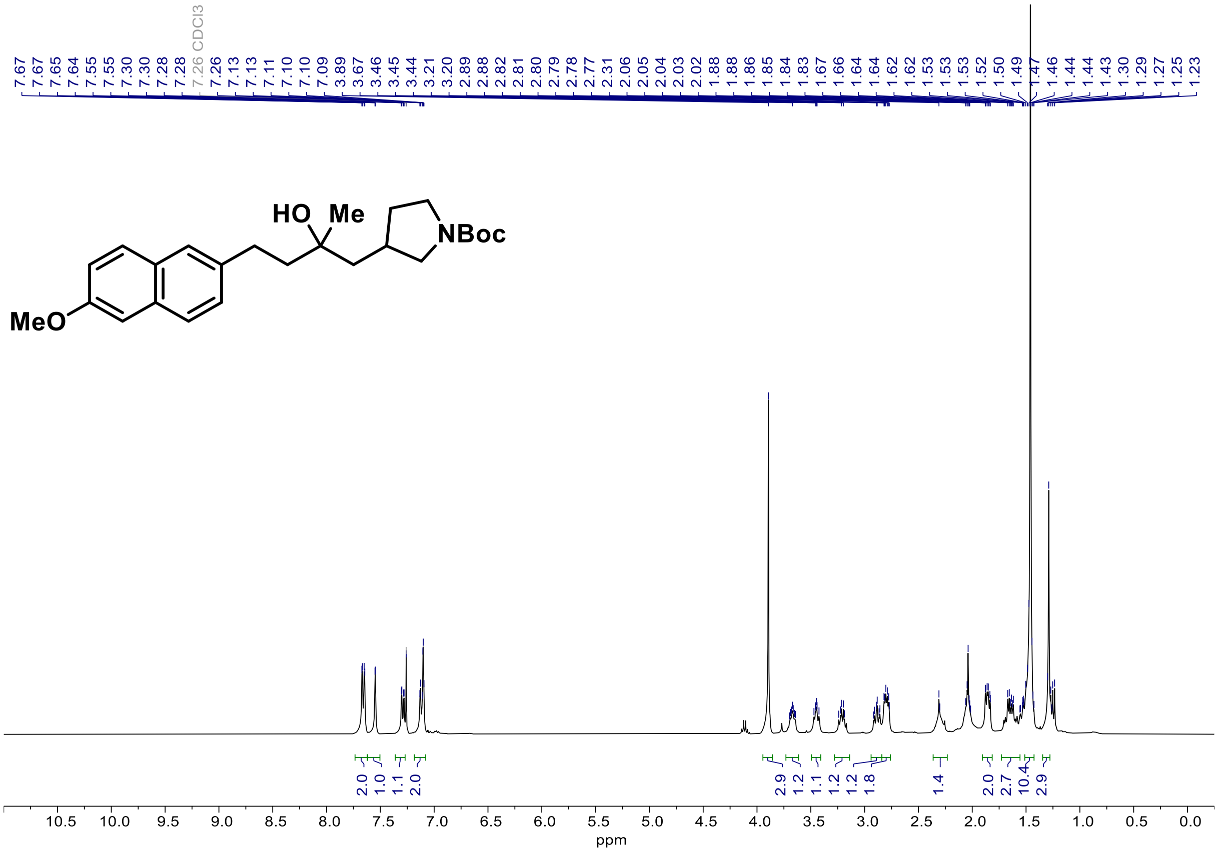

53 – ^{13}C NMR (101 MHz, CDCl$_3$)

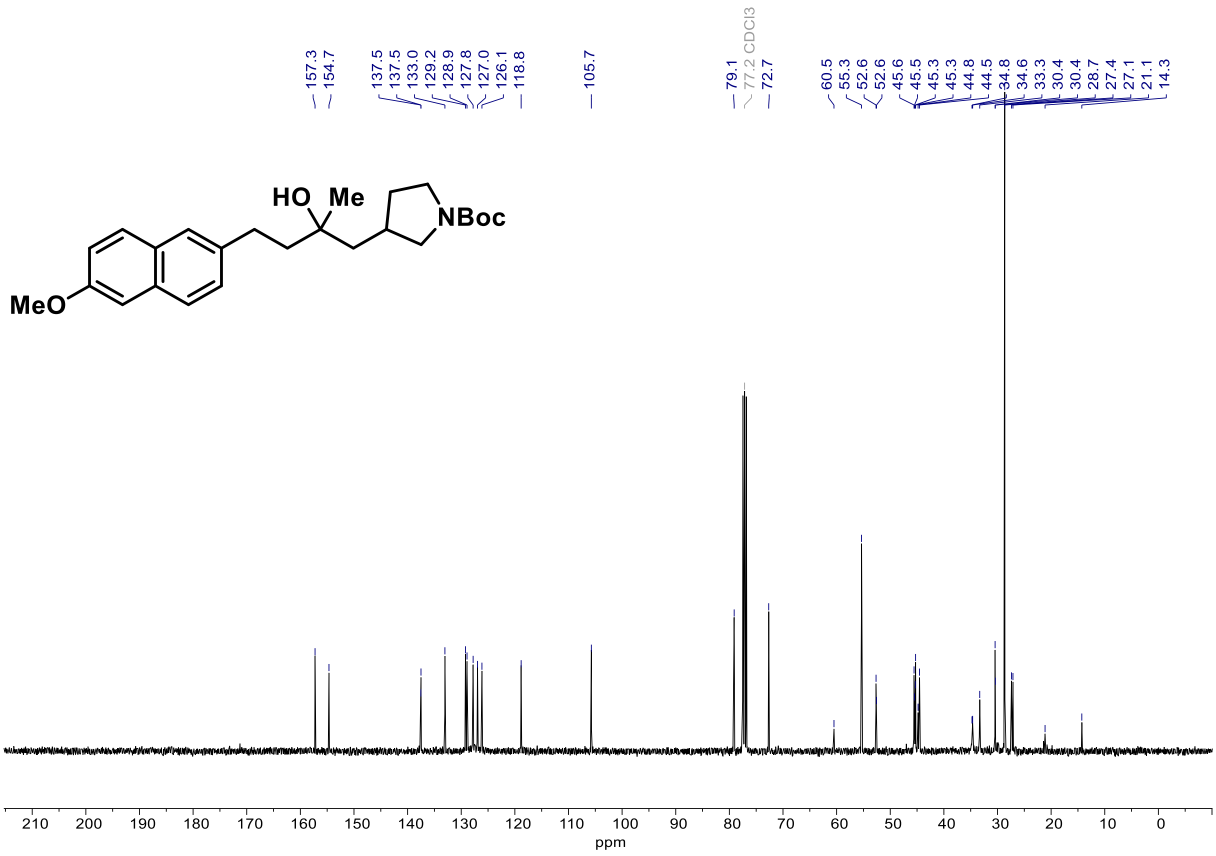

54 – ^{1}H NMR (500 MHz, CDCl$_3$)

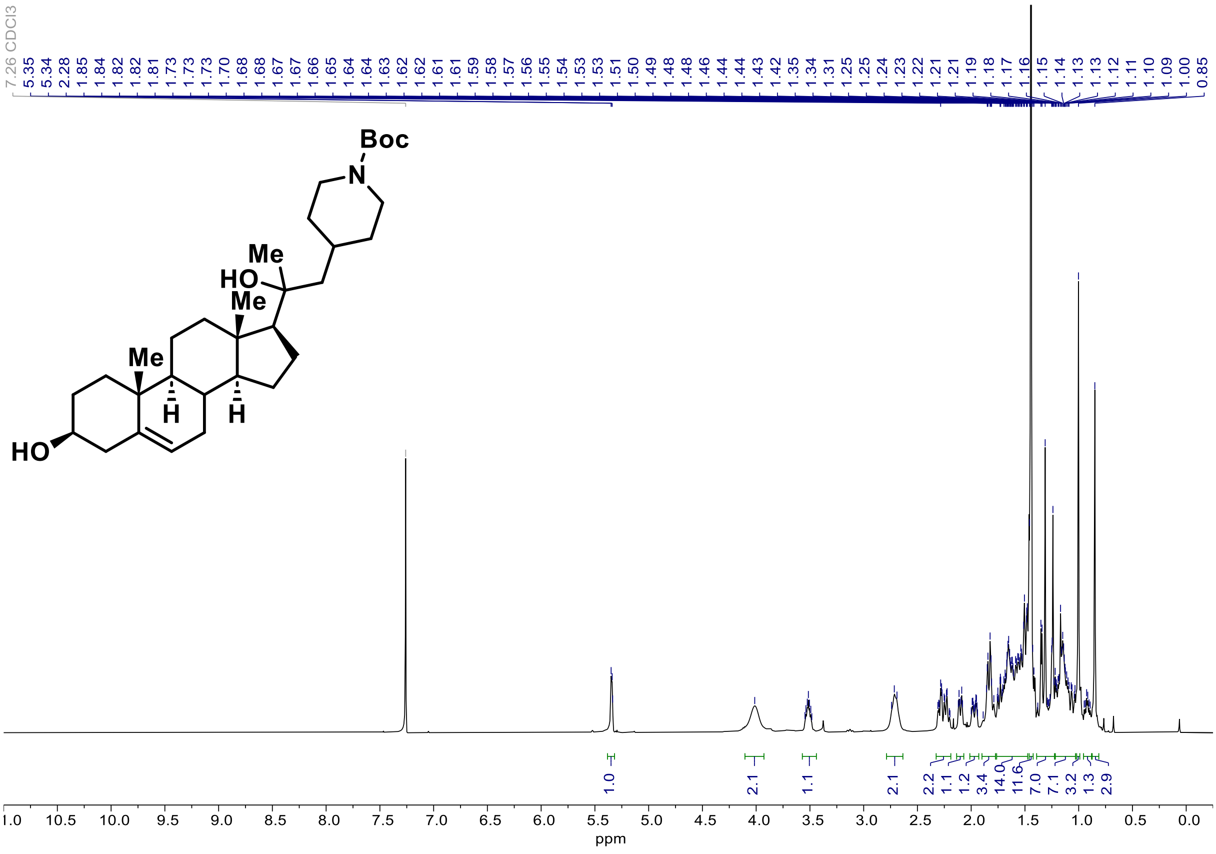

54 – ^{13}C NMR (126 MHz, CDCl$_3$)

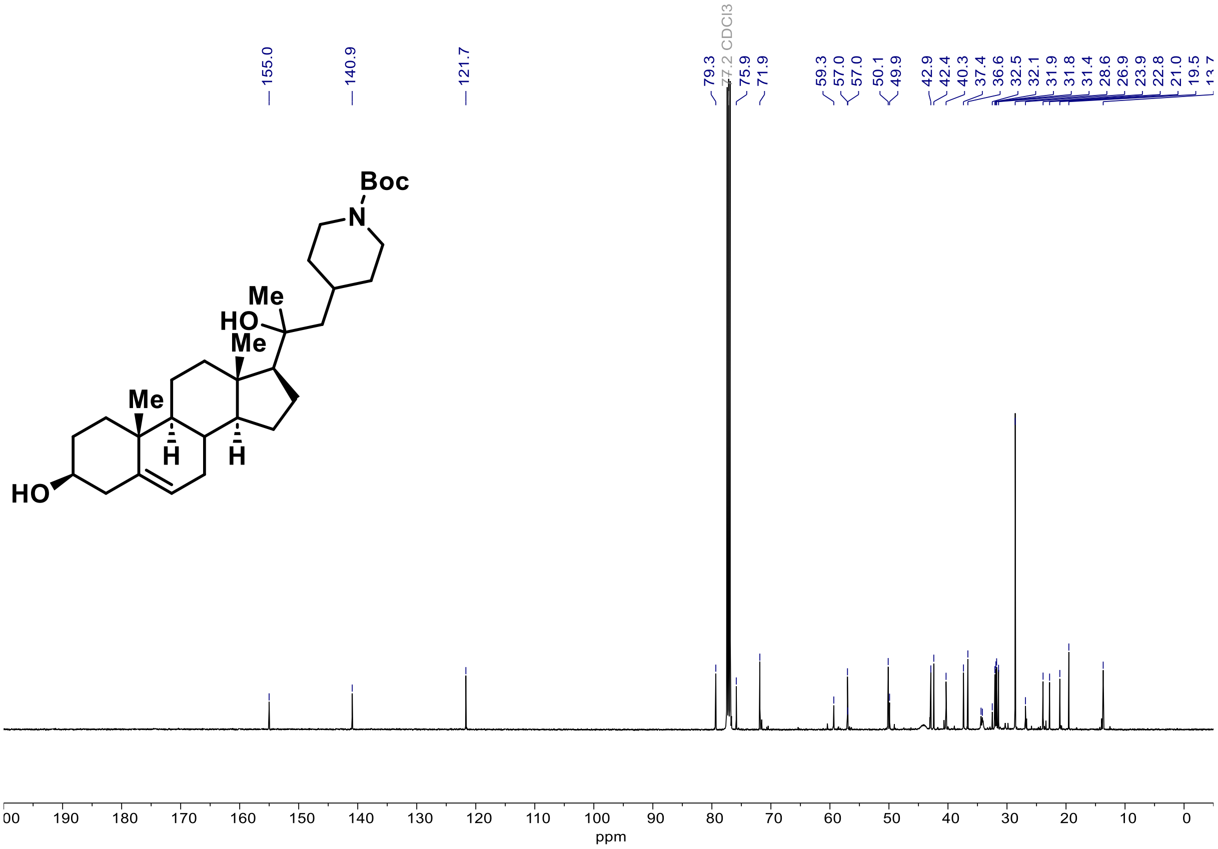

55 – ^{1}H NMR (500 MHz, CDCl$_3$)

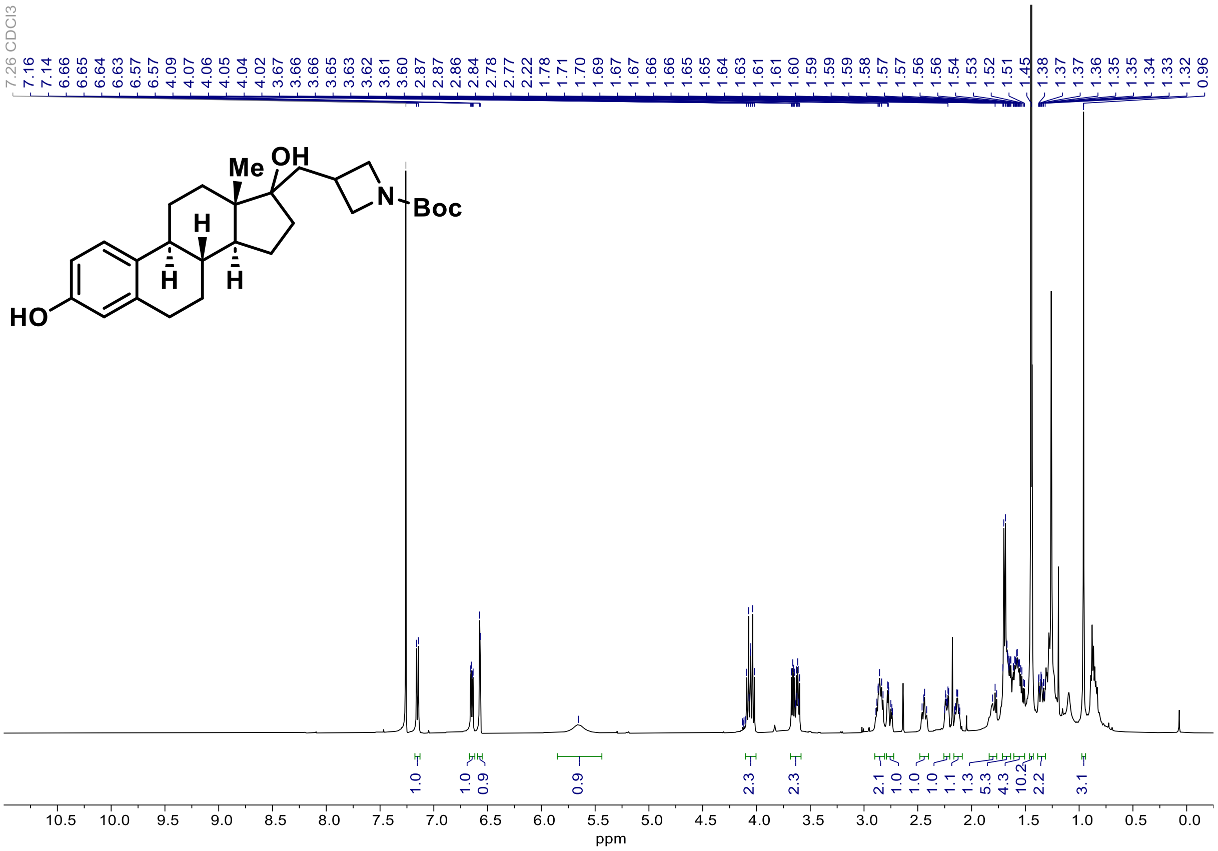

55 – ^{13}C NMR (126 MHz, CDCl$_3$)

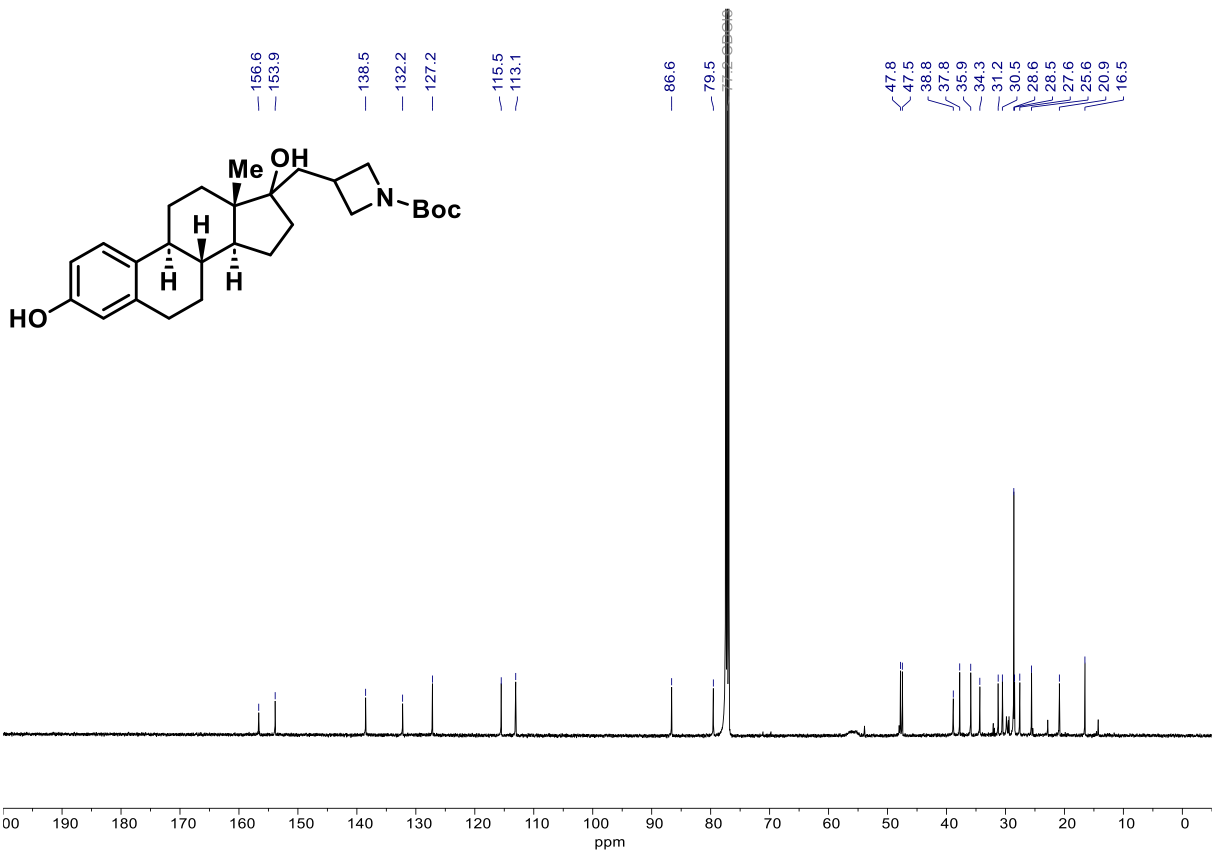

56 – ^{1}H NMR (500 MHz, MeOH-d^4)

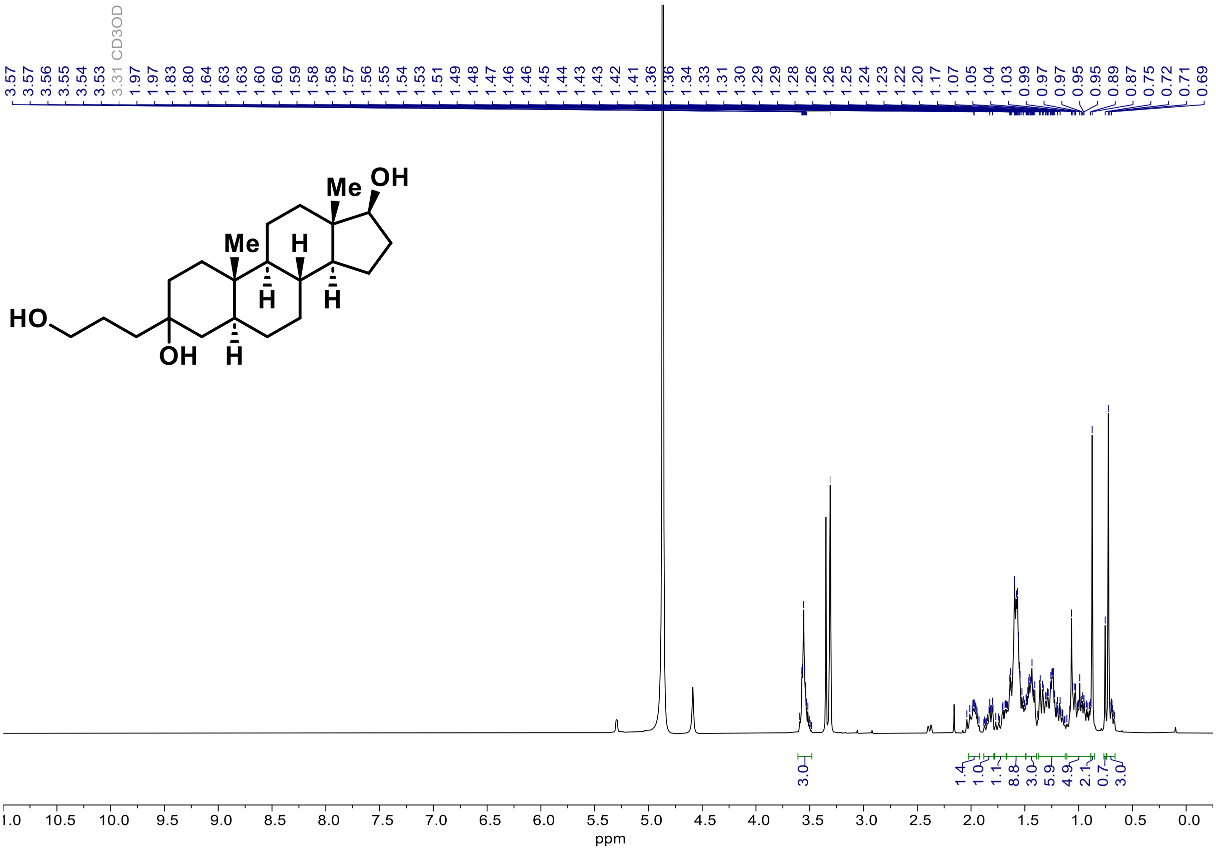

56 – ^{13}C NMR (126 MHz, MeOH-d^4)

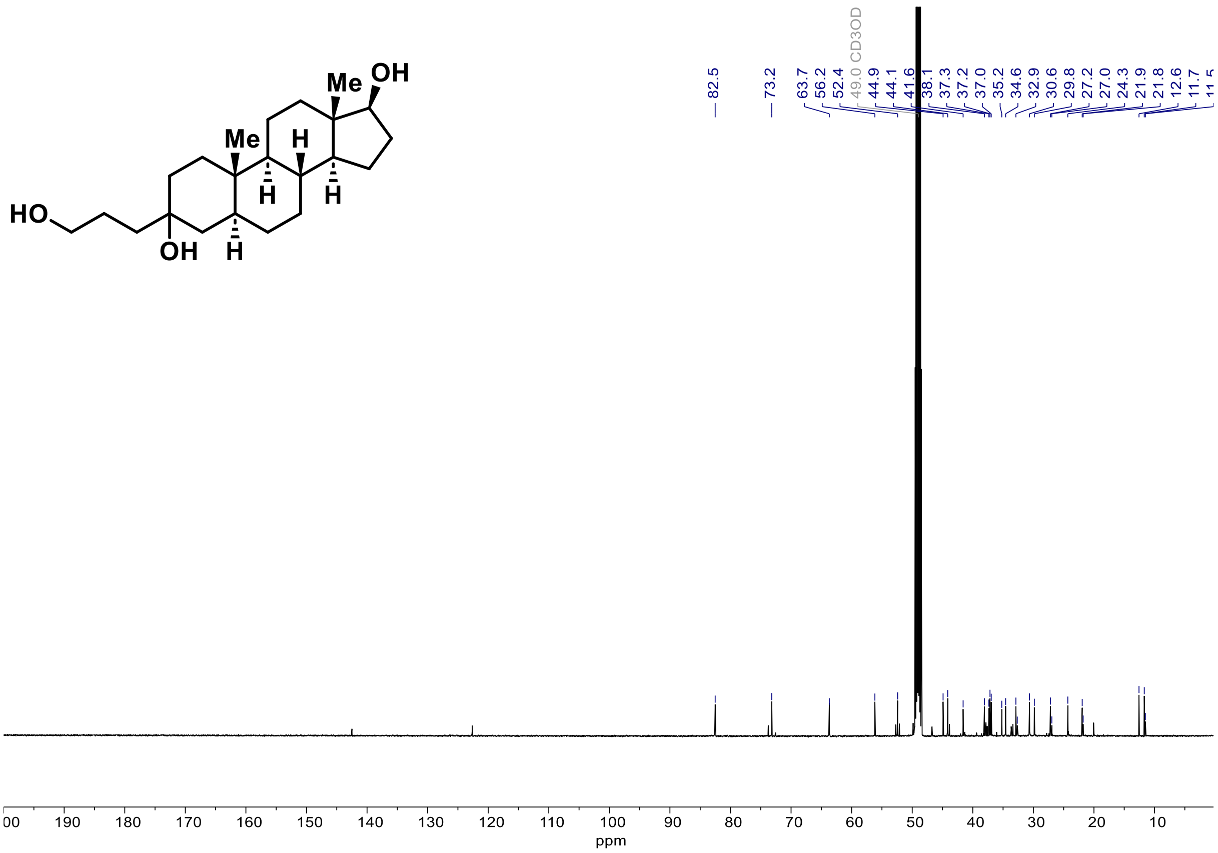

59 – ^{1}H NMR (400 MHz, $CDCl_3$)

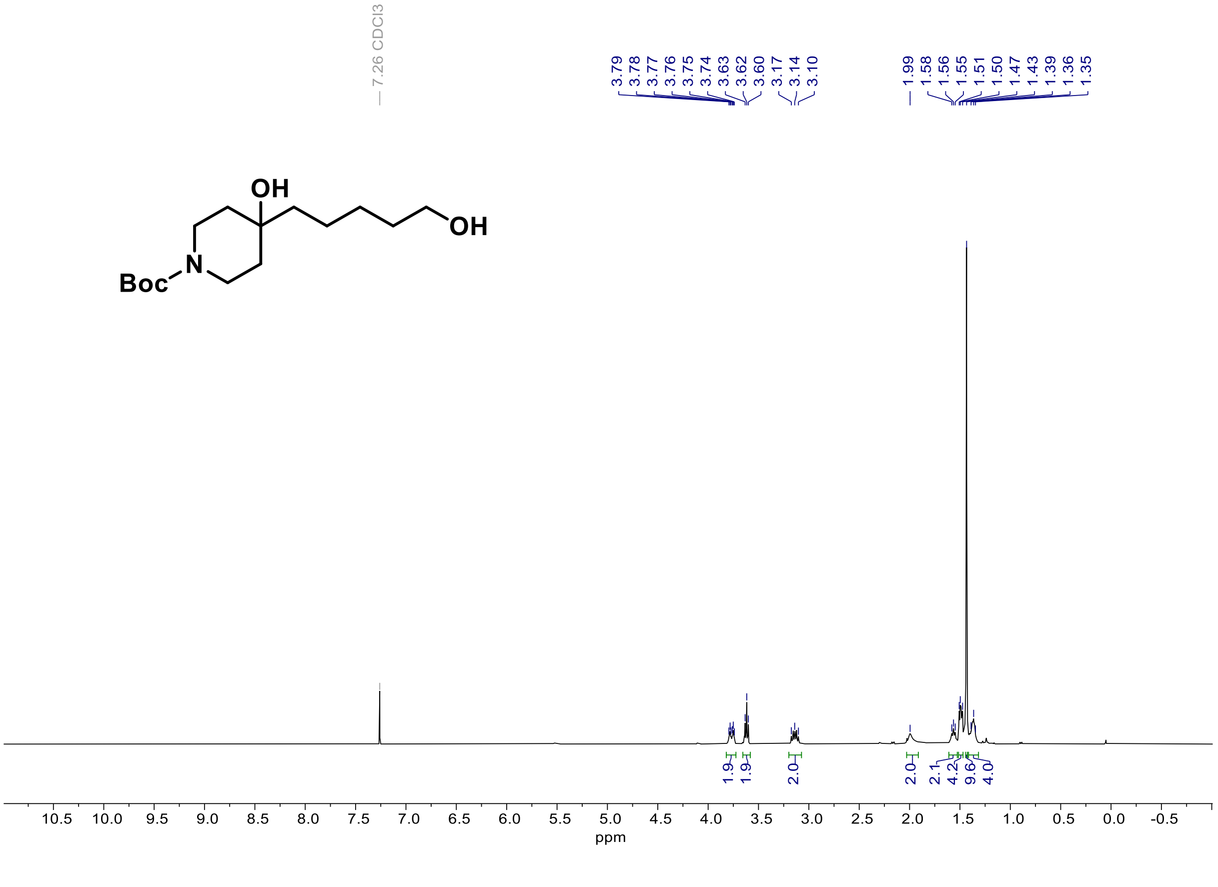

59 – ^{13}C NMR (101 MHz, $CDCl_3$)

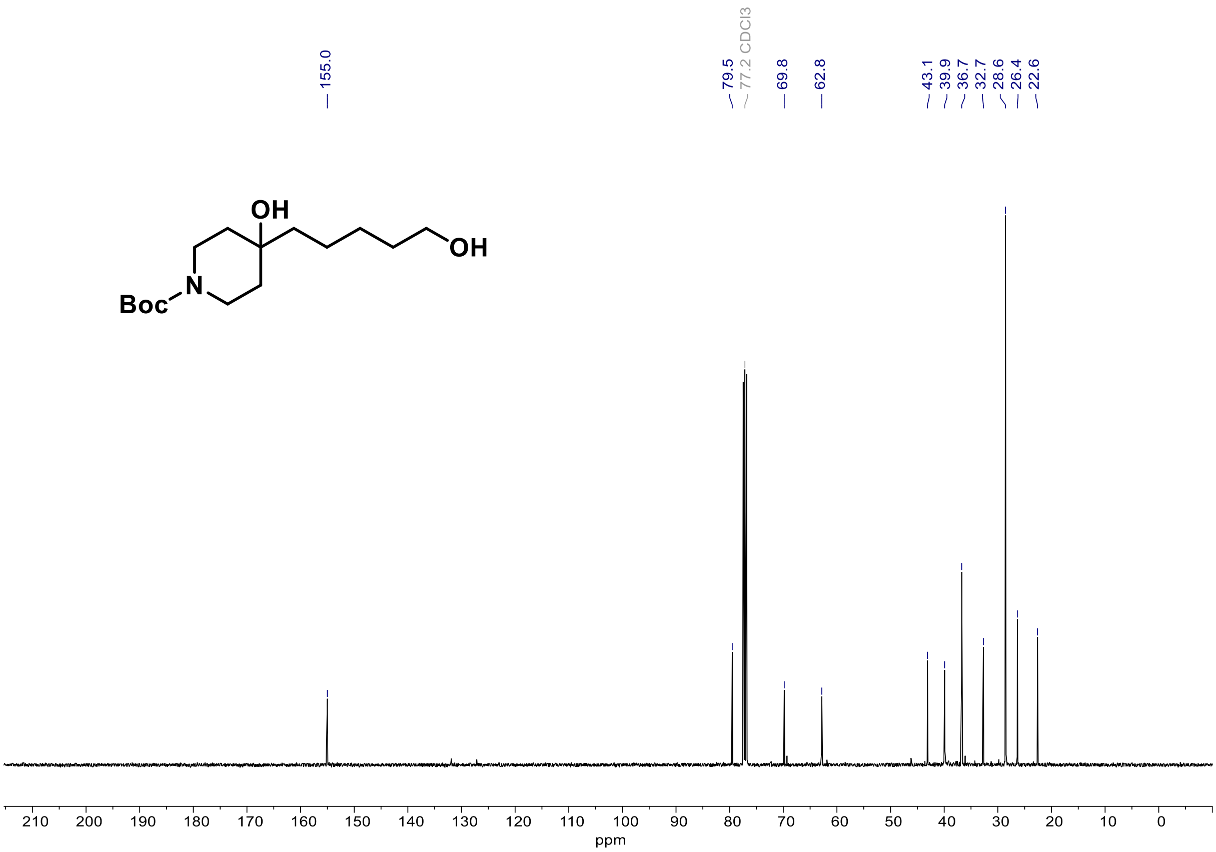

60 – ^{1}H NMR (400 MHz, $CDCl_3$)

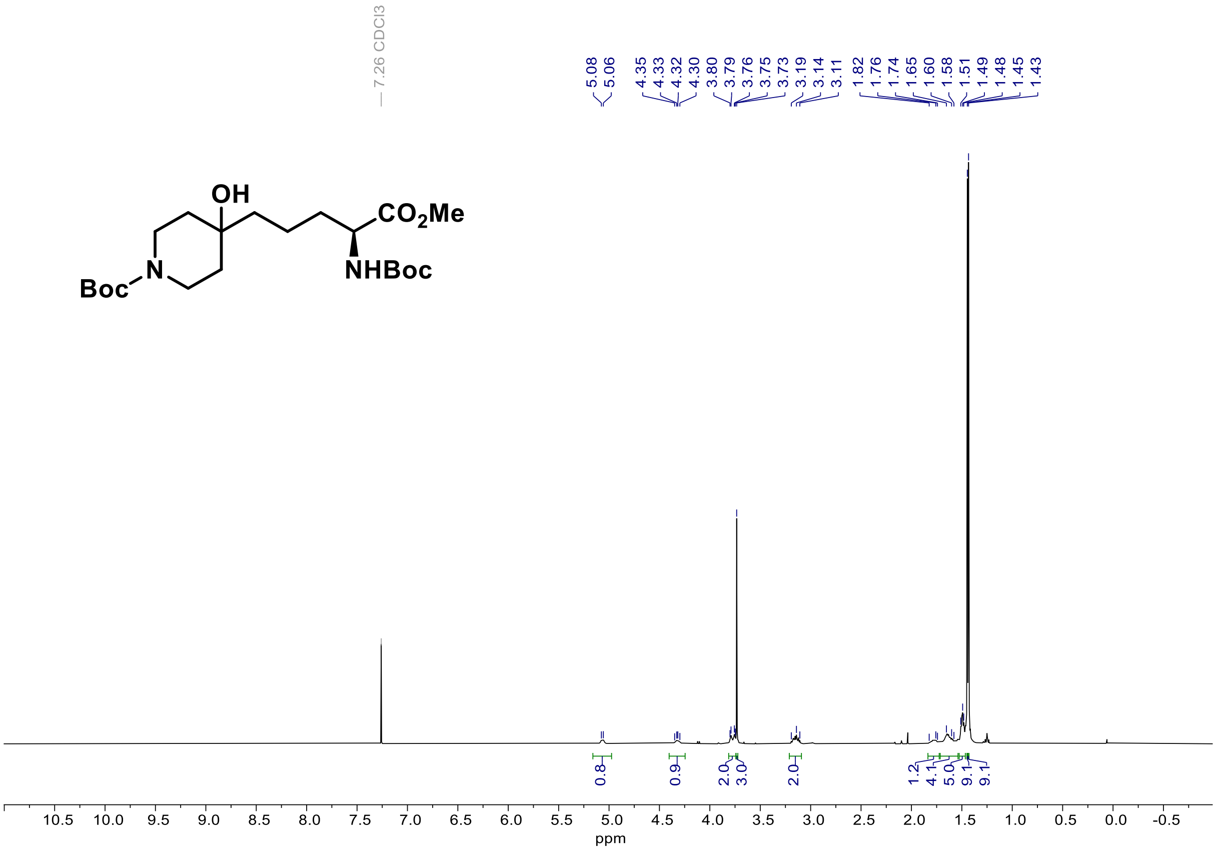

60 – ^{13}C NMR (101 MHz, CDCl$_3$)

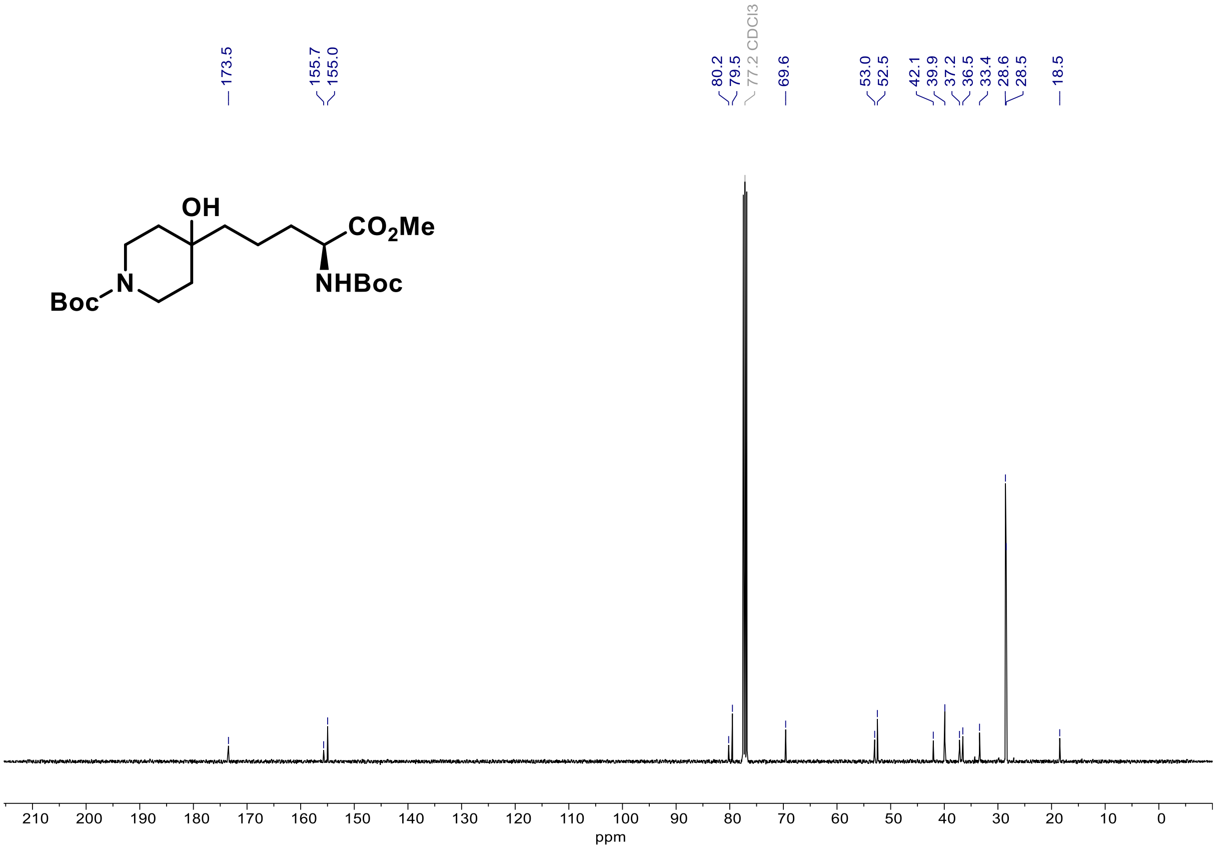

61 – ^{1}H NMR (400 MHz, CDCl$_3$)

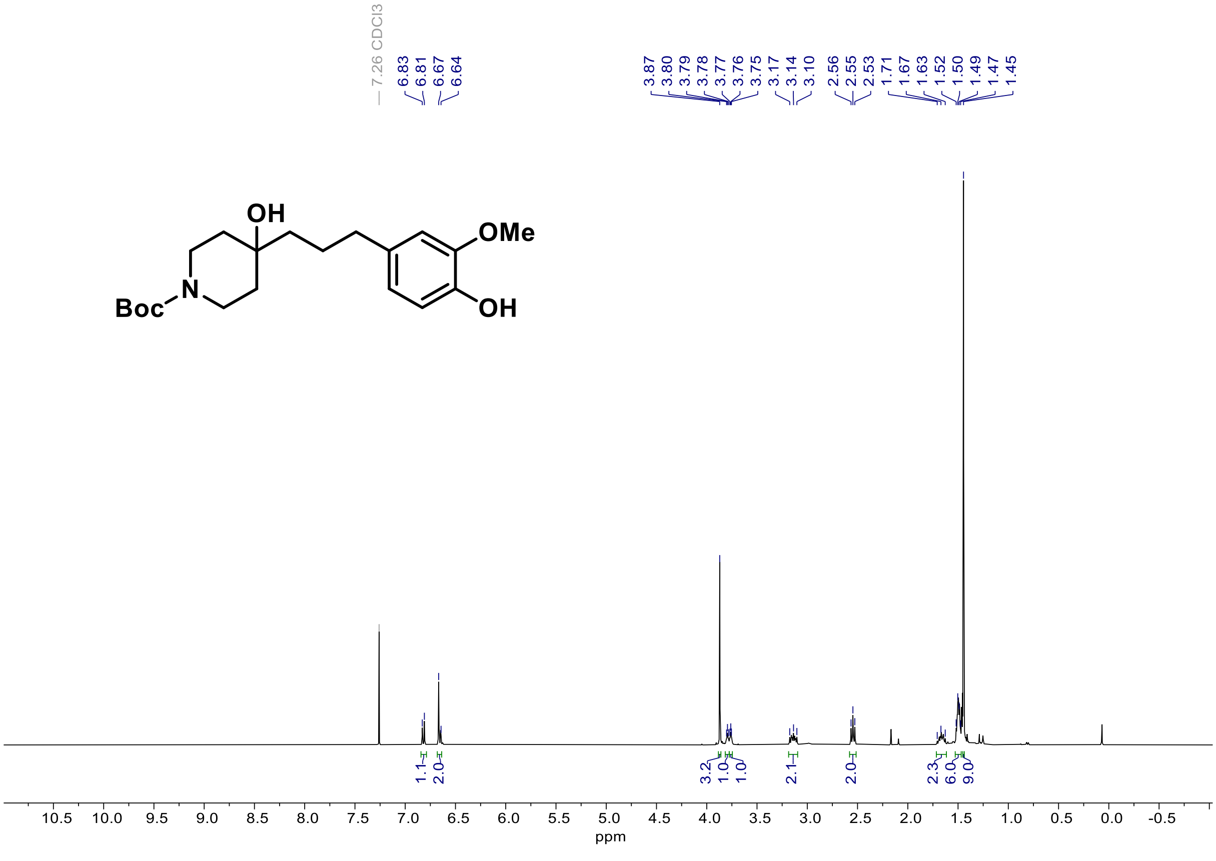

61 – ^{13}C NMR (101 MHz, CDCl$_3$)

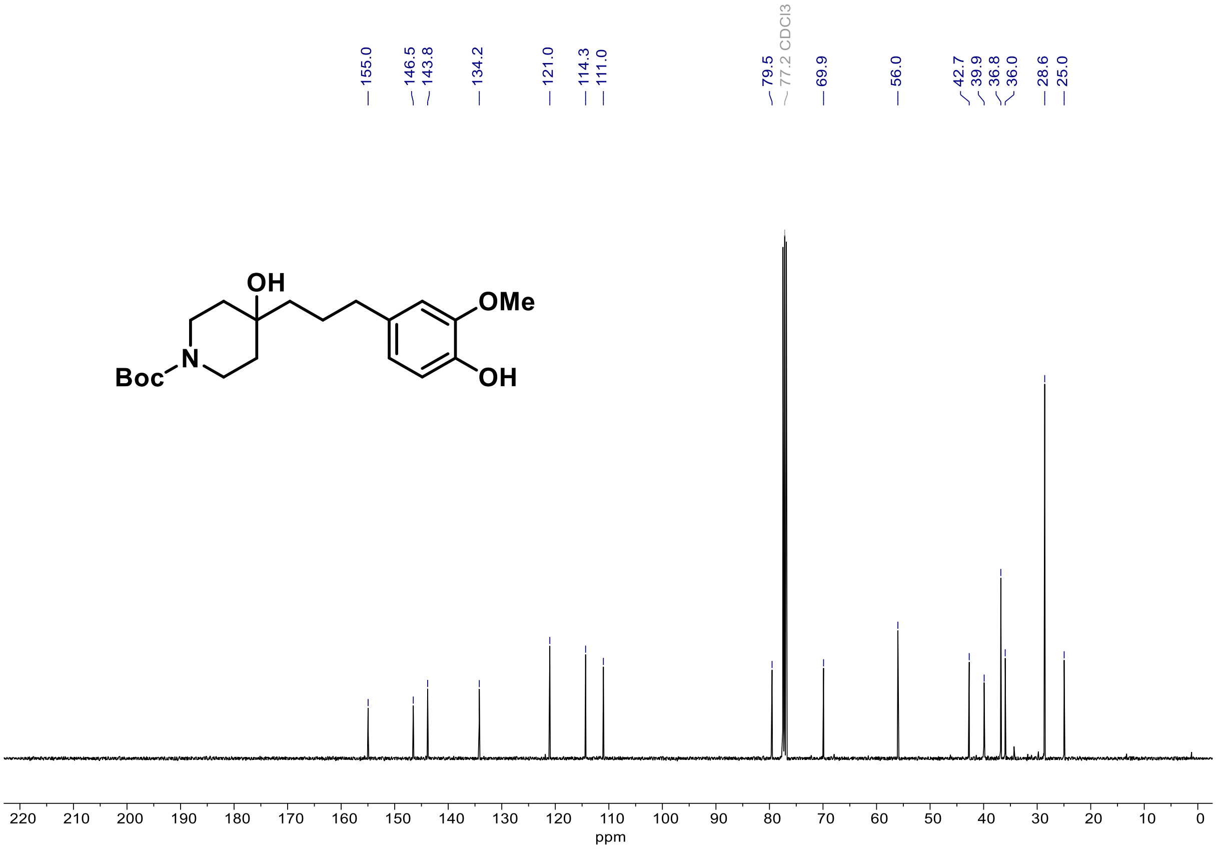

62 – ^{1}H NMR (400 MHz, CDCl$_3$)

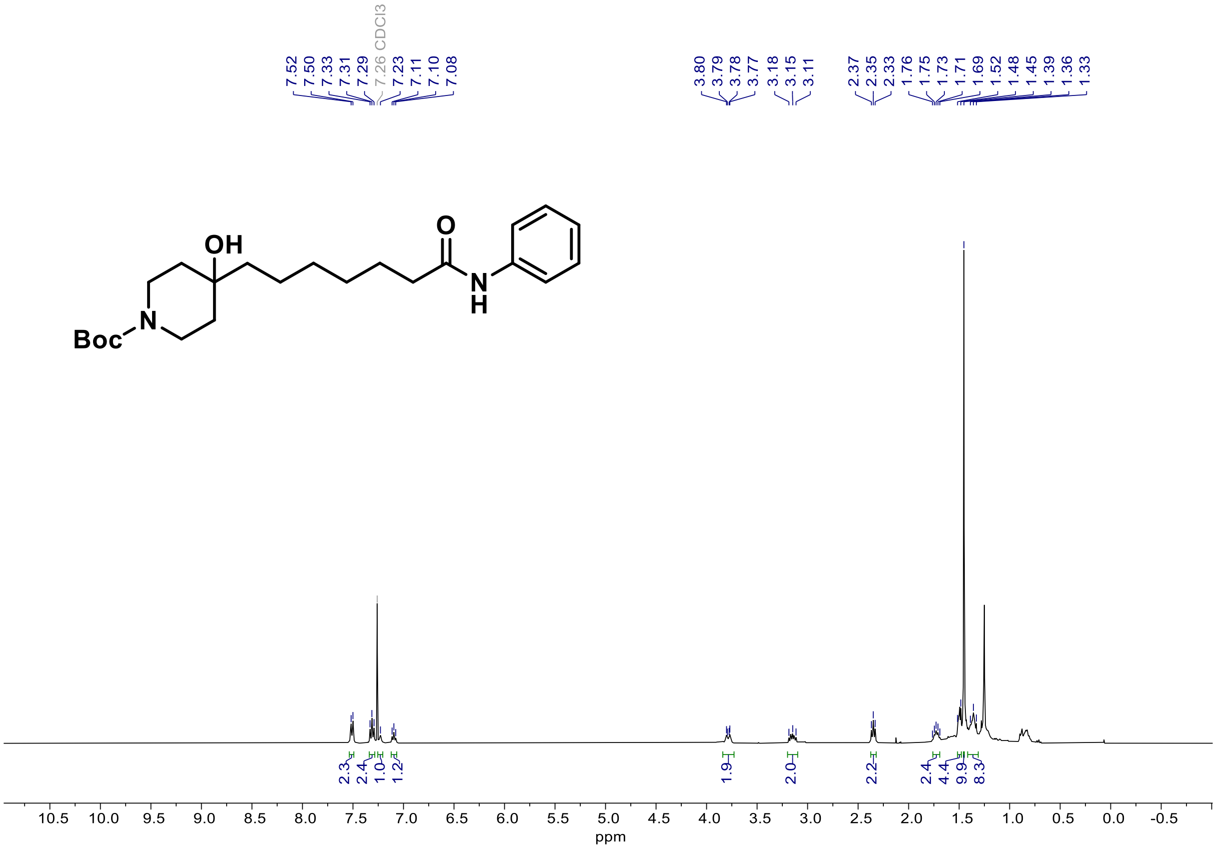

62 – ^{13}C NMR (101 MHz, $CDCl_3$)

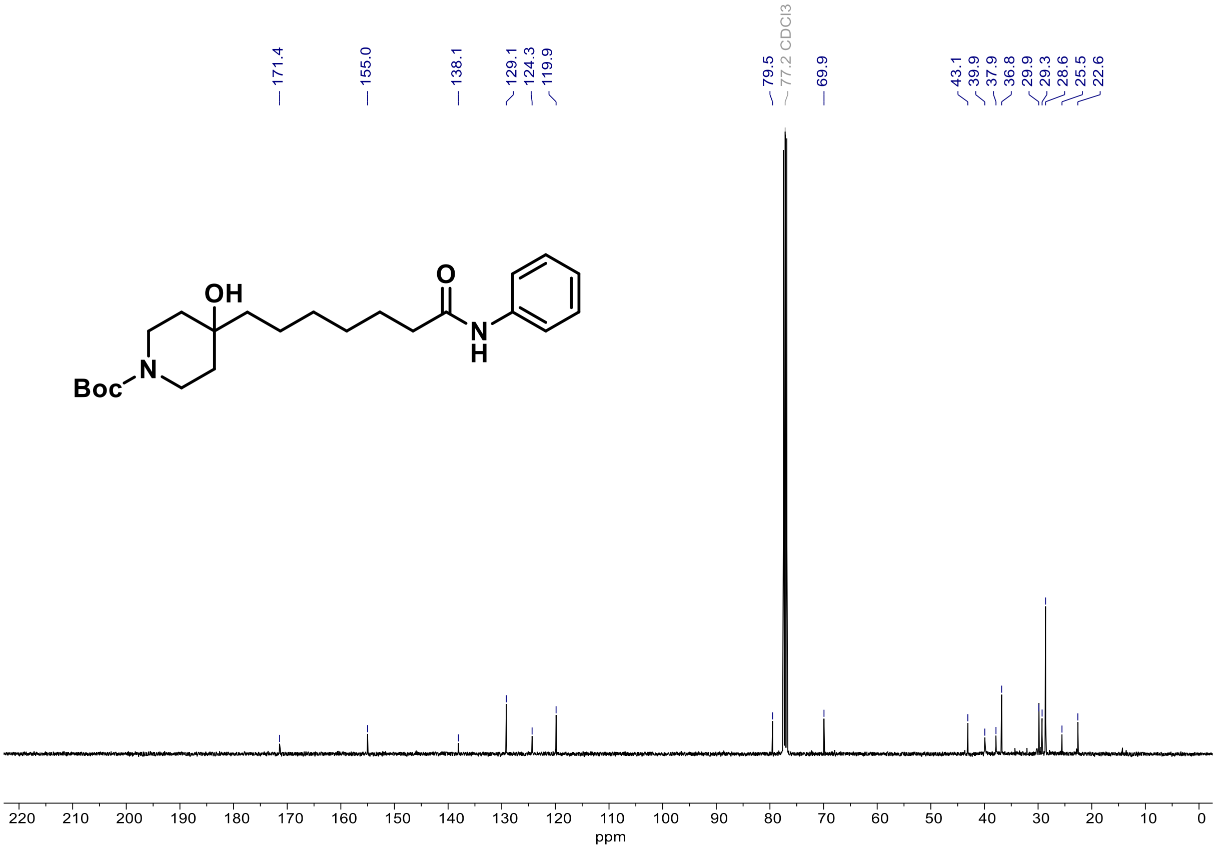

63 – ^{1}H NMR (400 MHz, $CDCl_3$)

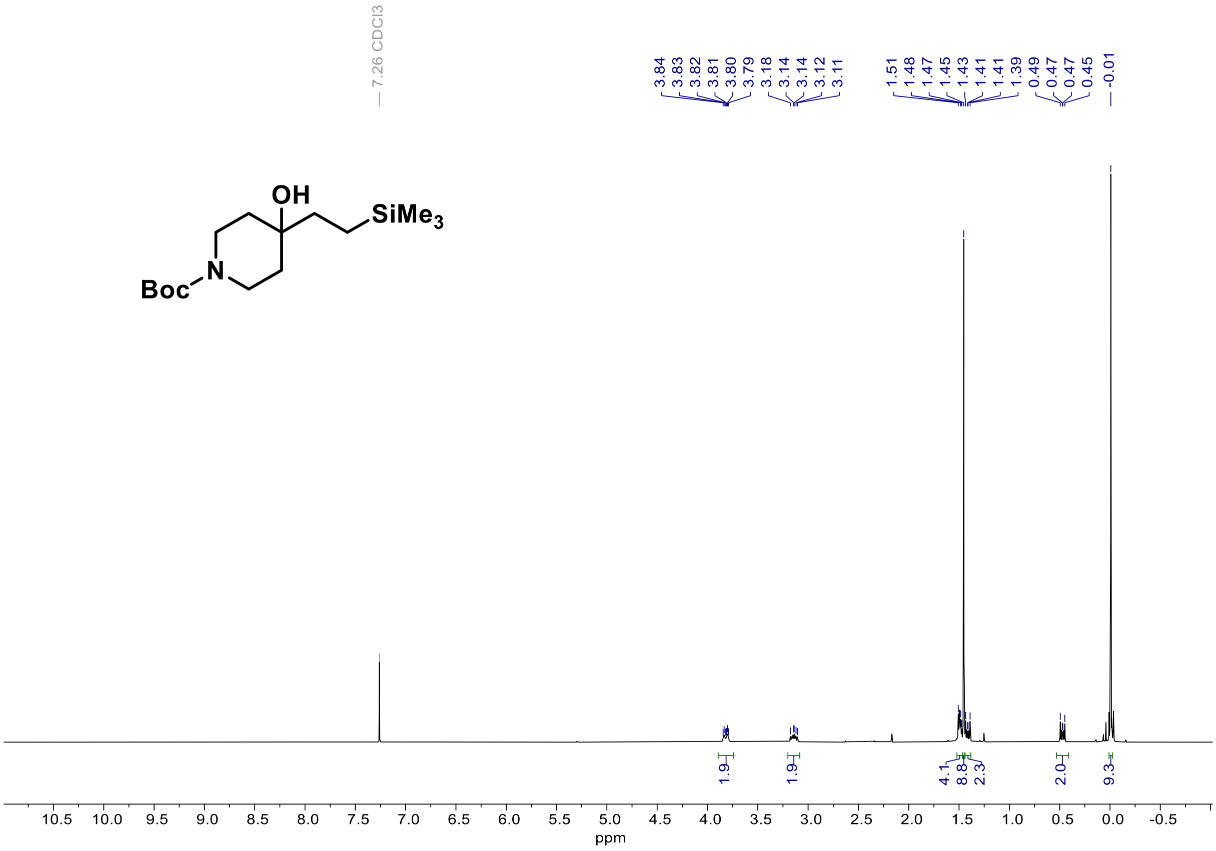

63 – ^{13}C NMR (101 MHz, $CDCl_3$)

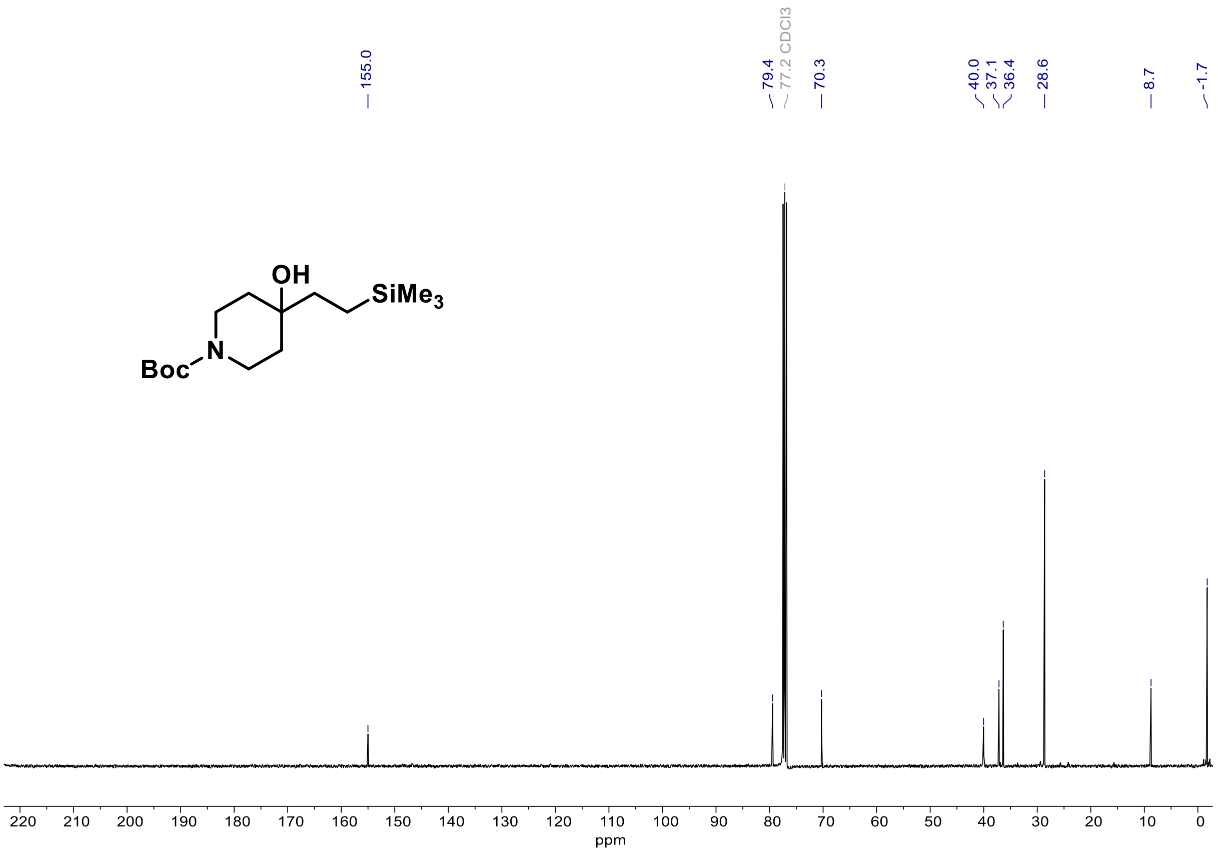

64 – ^{1}H NMR (400 MHz, $CDCl_3$)

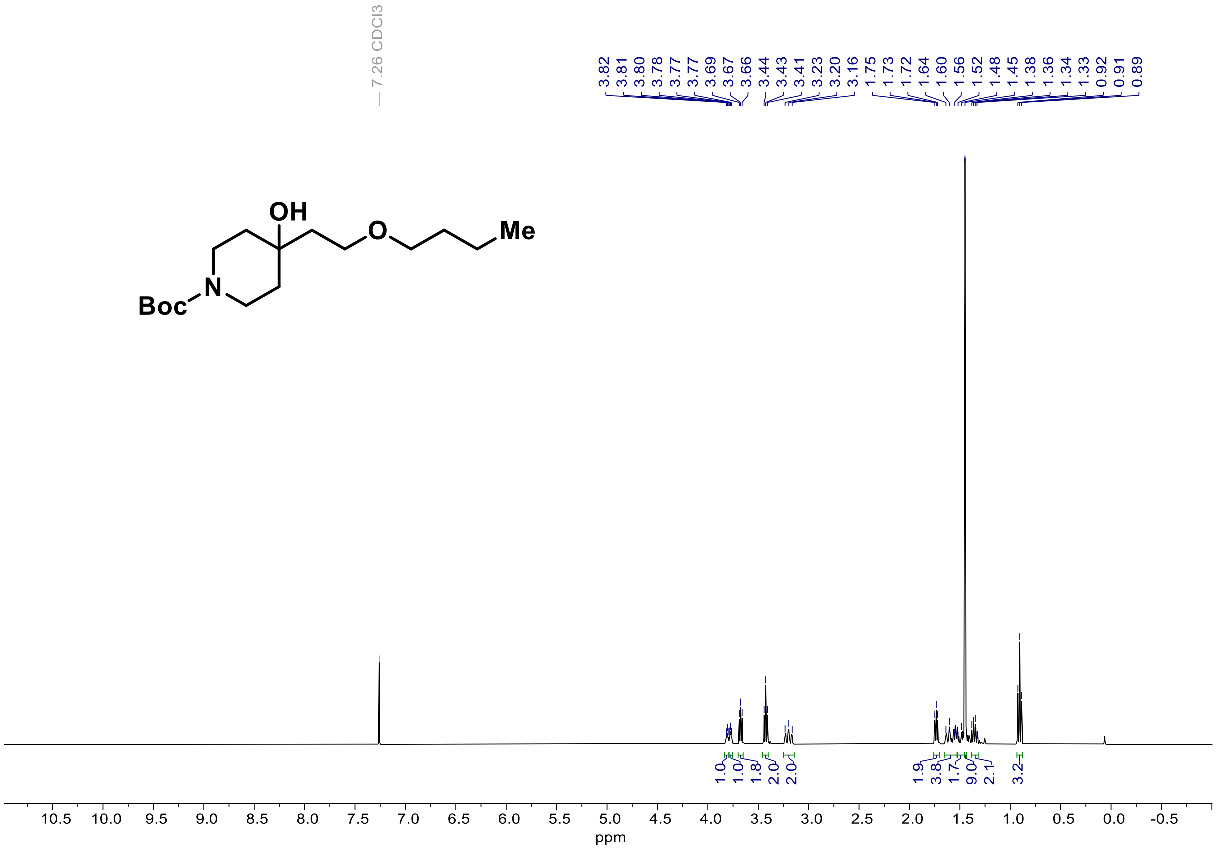

64 – ^{13}C NMR (101 MHz, $CDCl_3$)

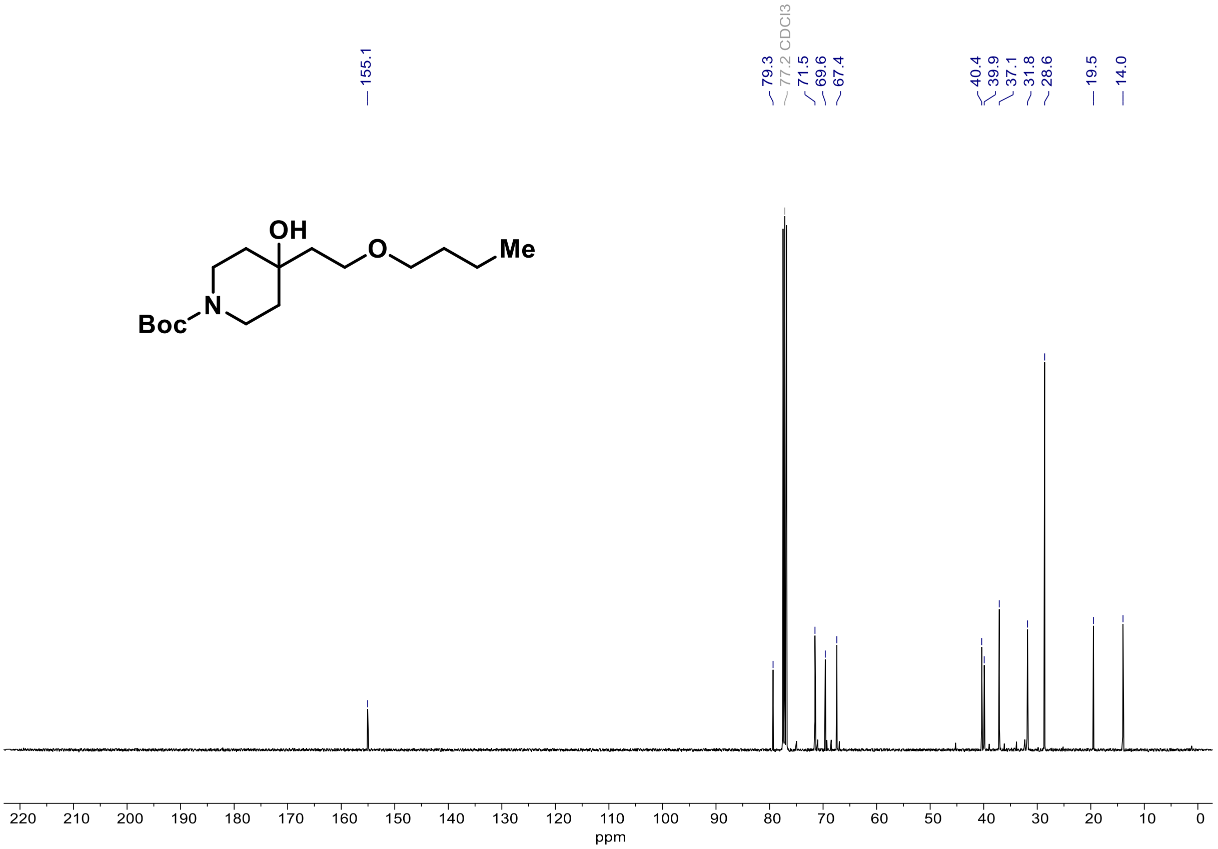

65 – ^{1}H NMR (400 MHz, $CDCl_3$)

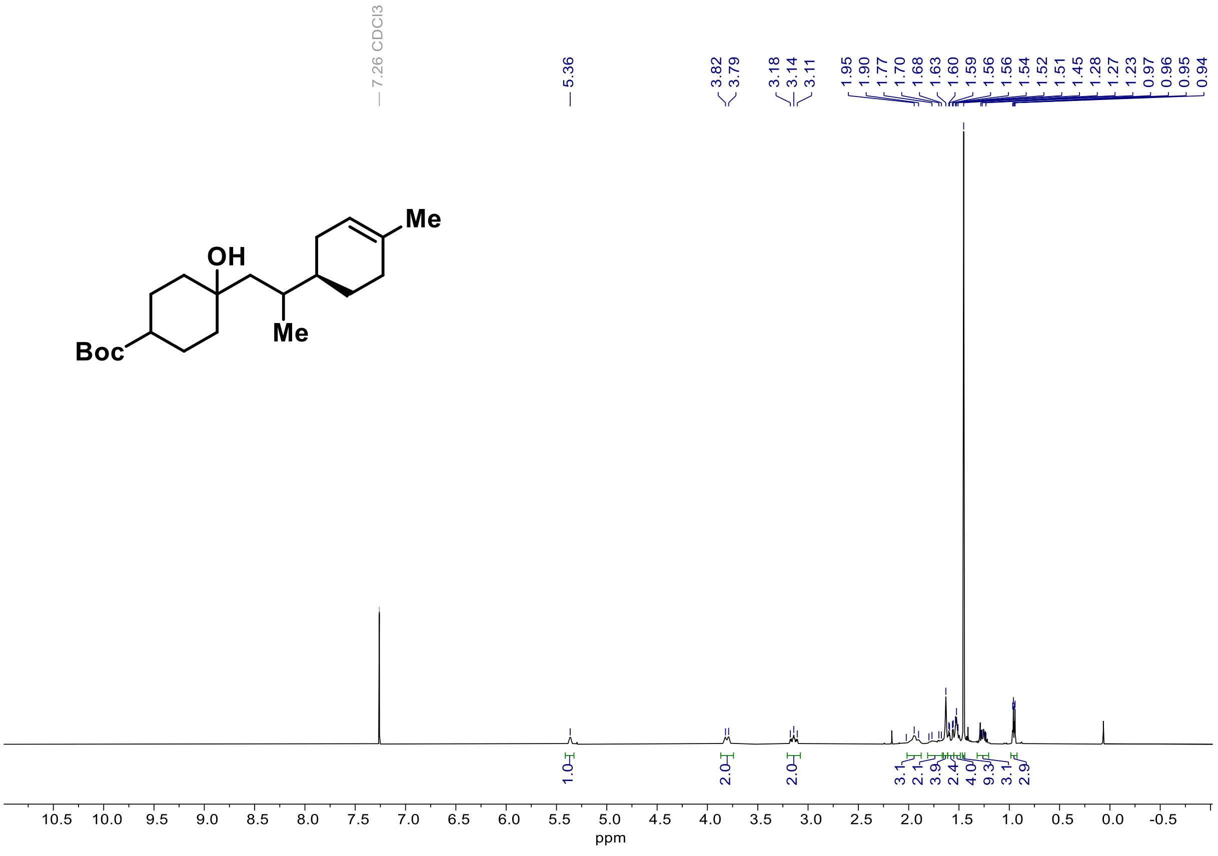

65 – ^{13}C NMR (101 MHz, CDCl$_3$)

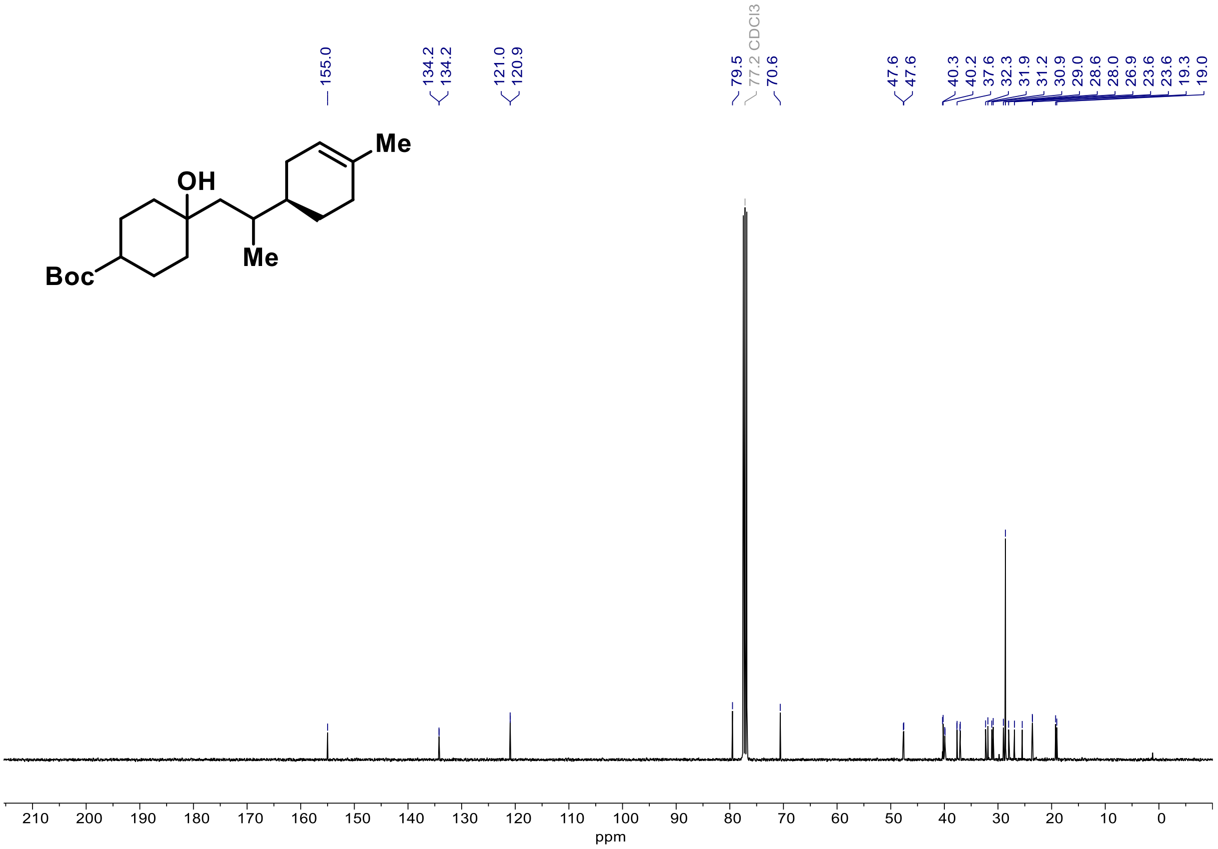

66 – ^{1}H NMR (400 MHz, CDCl$_3$)

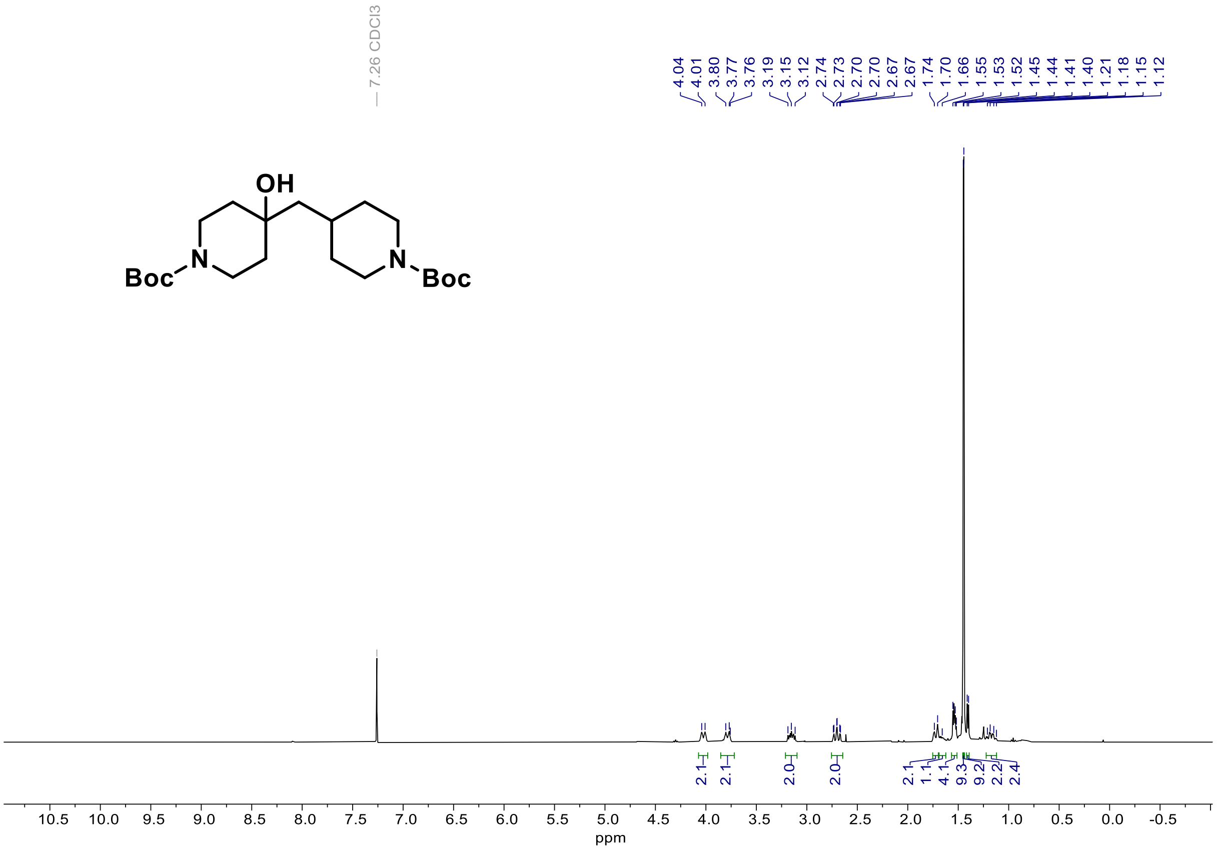

66 – ^{13}C NMR (101 MHz, $CDCl_3$)

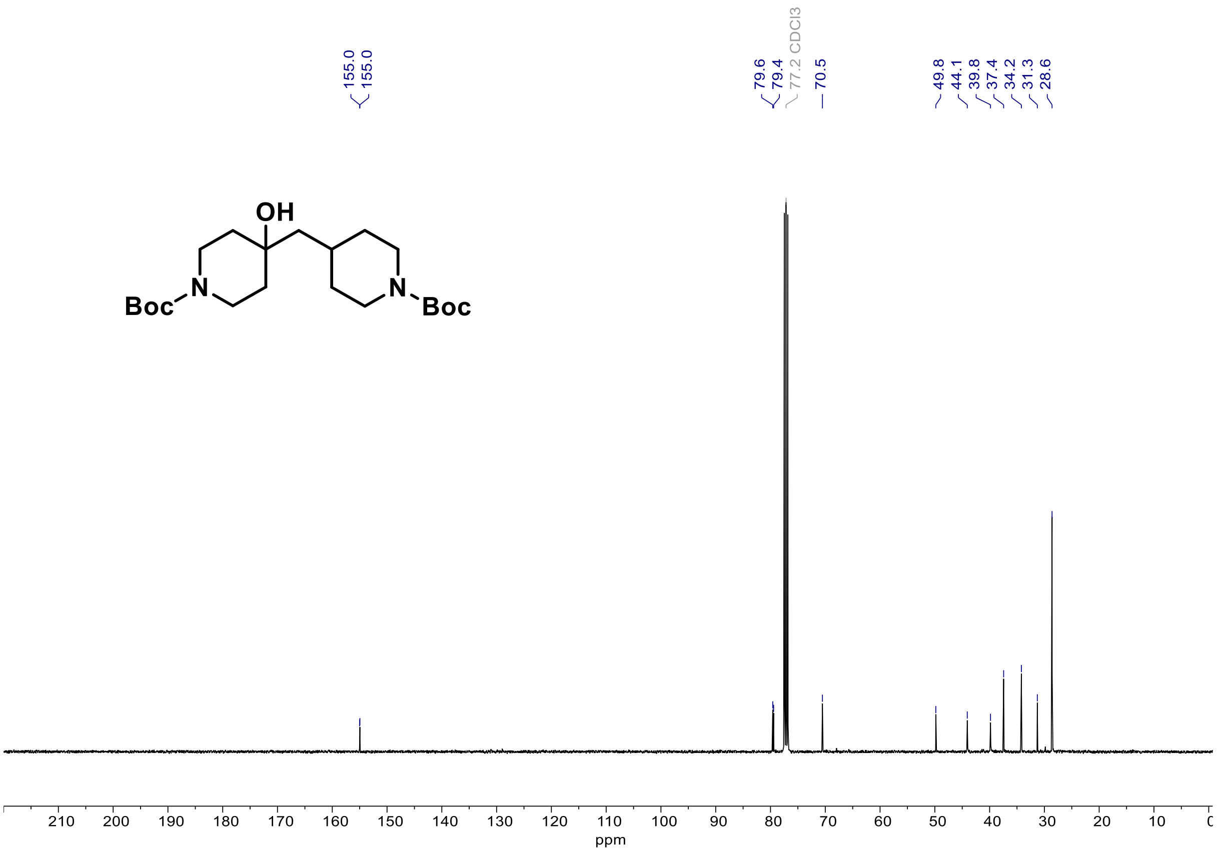

67 – ^{1}H NMR (400 MHz, $CDCl_3$)

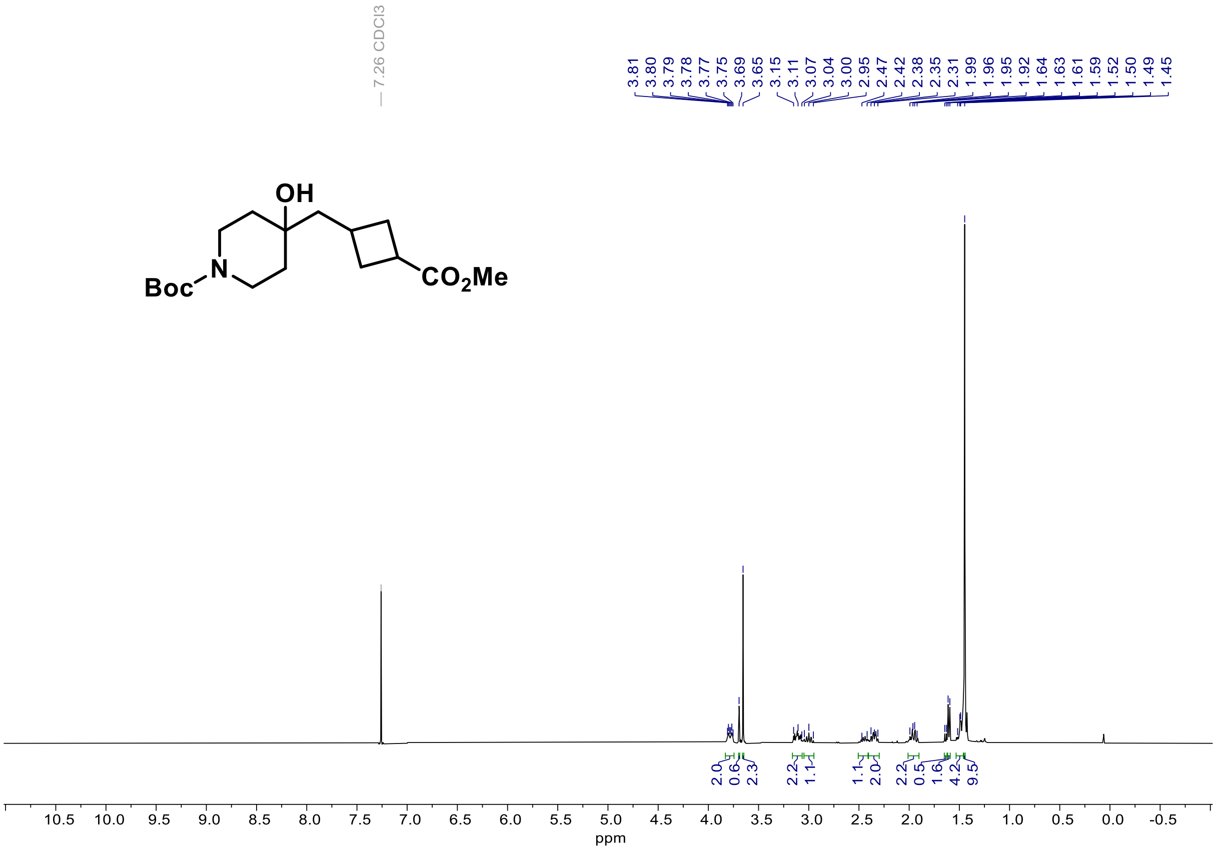

67 – ^{13}C NMR (101 MHz, $CDCl_3$)

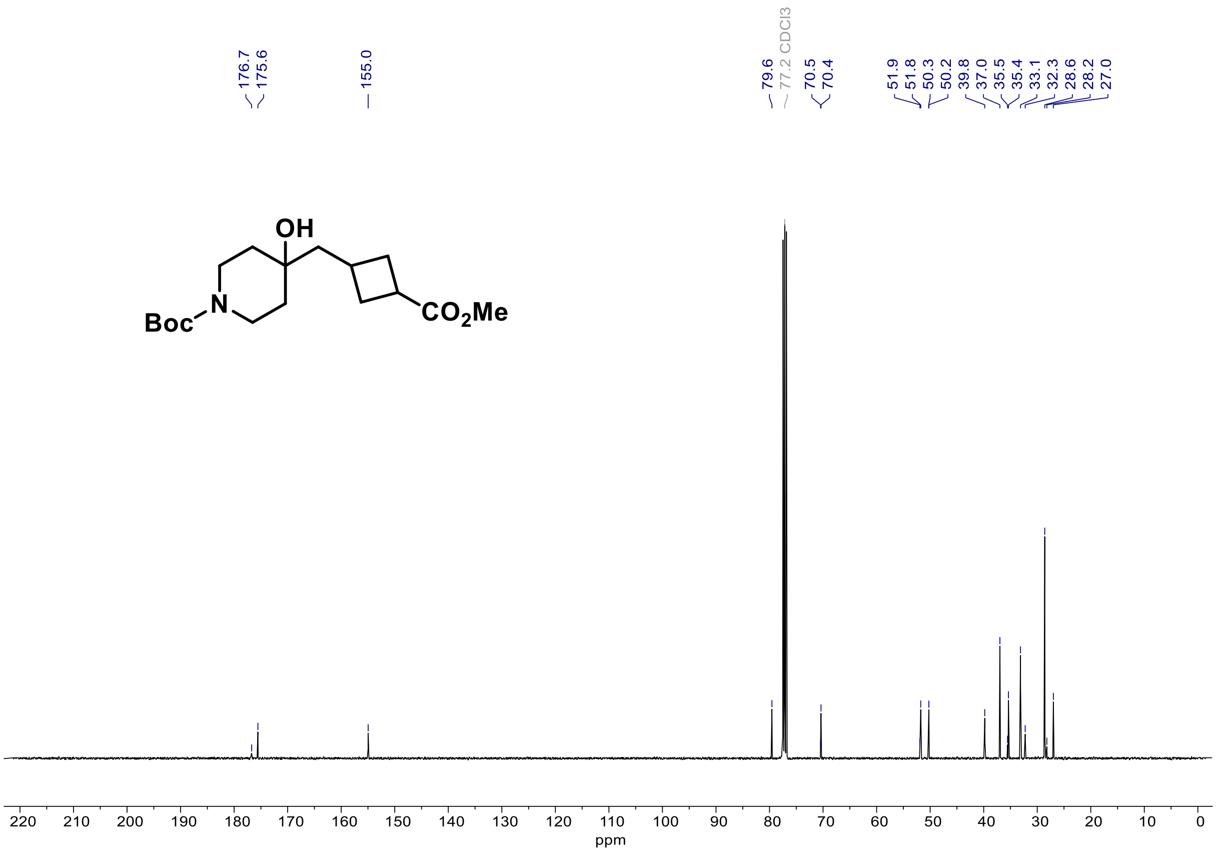

68 – ^{1}H NMR (400 MHz, $CDCl_3$)

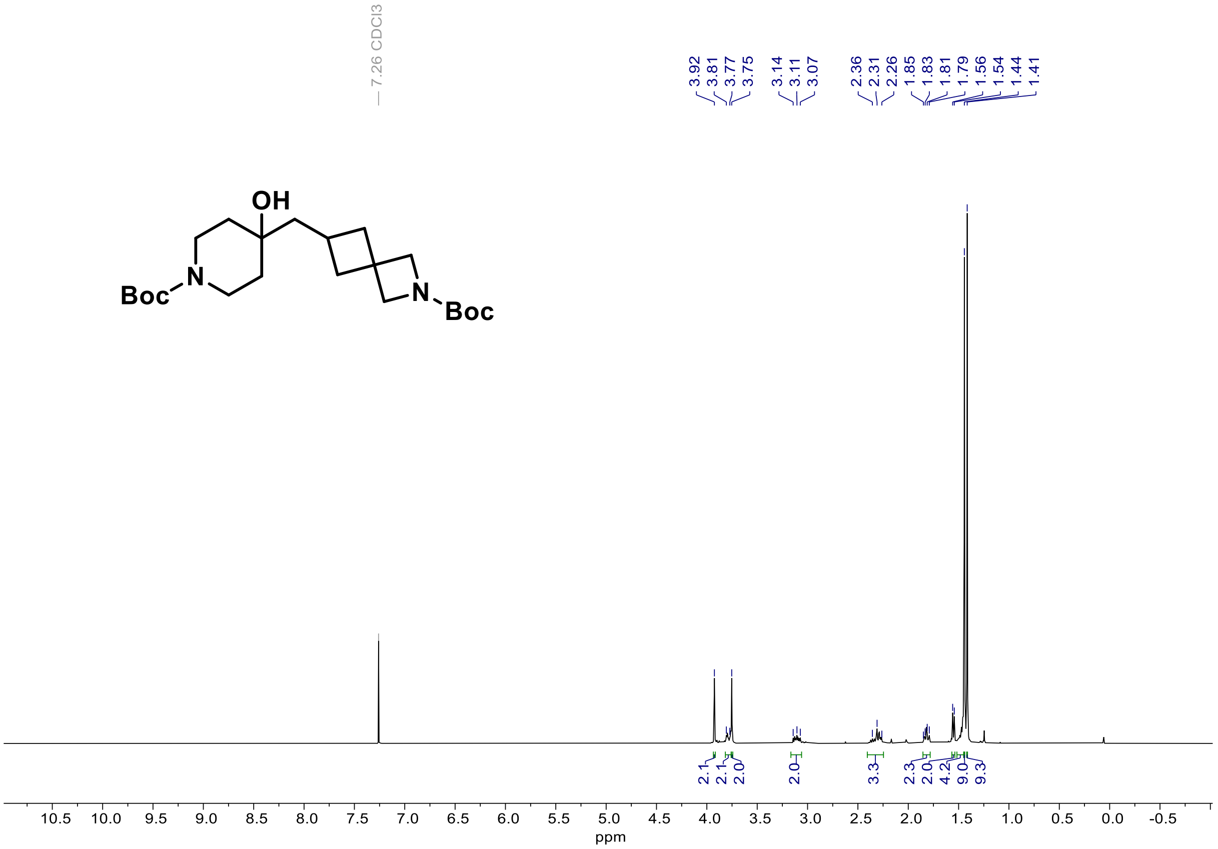

68 – ^{13}C NMR (101 MHz, $CDCl_3$)

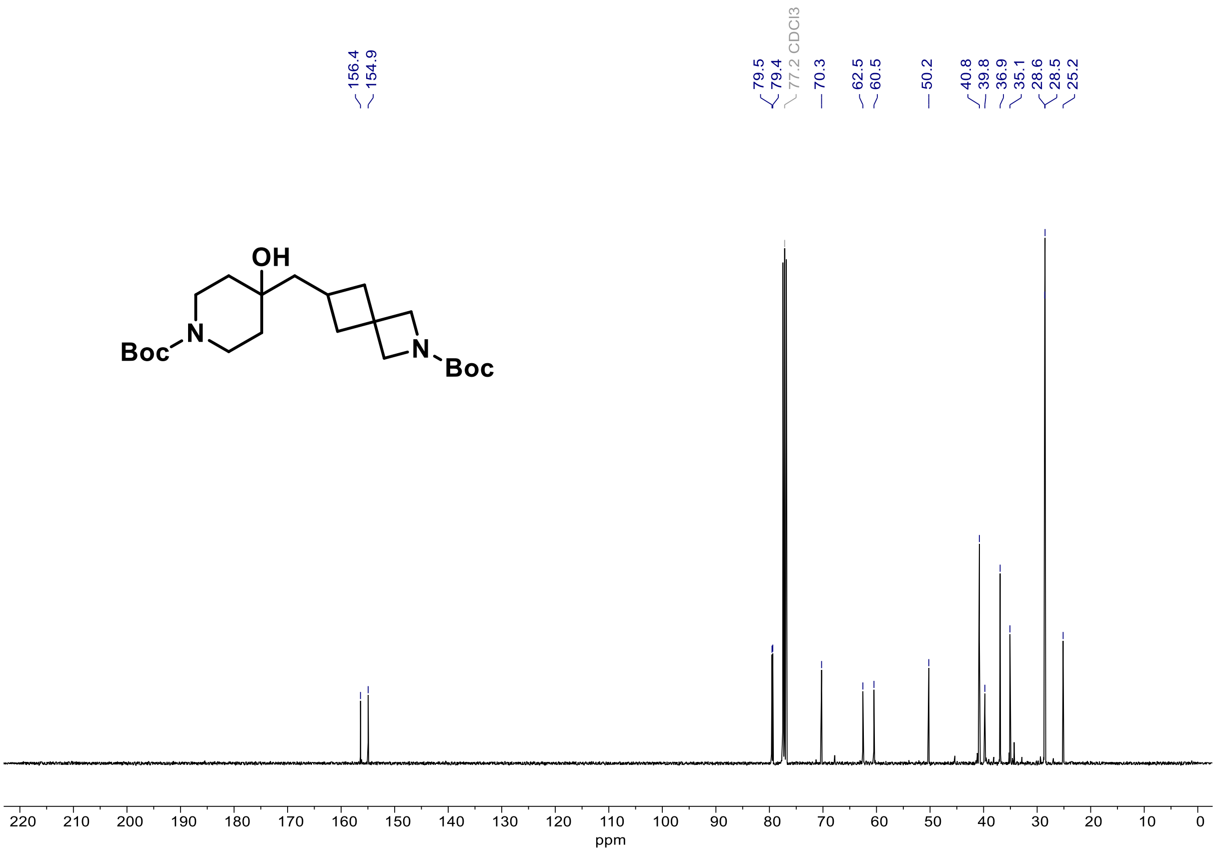

69 – ^{1}H NMR (400 MHz, $CDCl_3$)

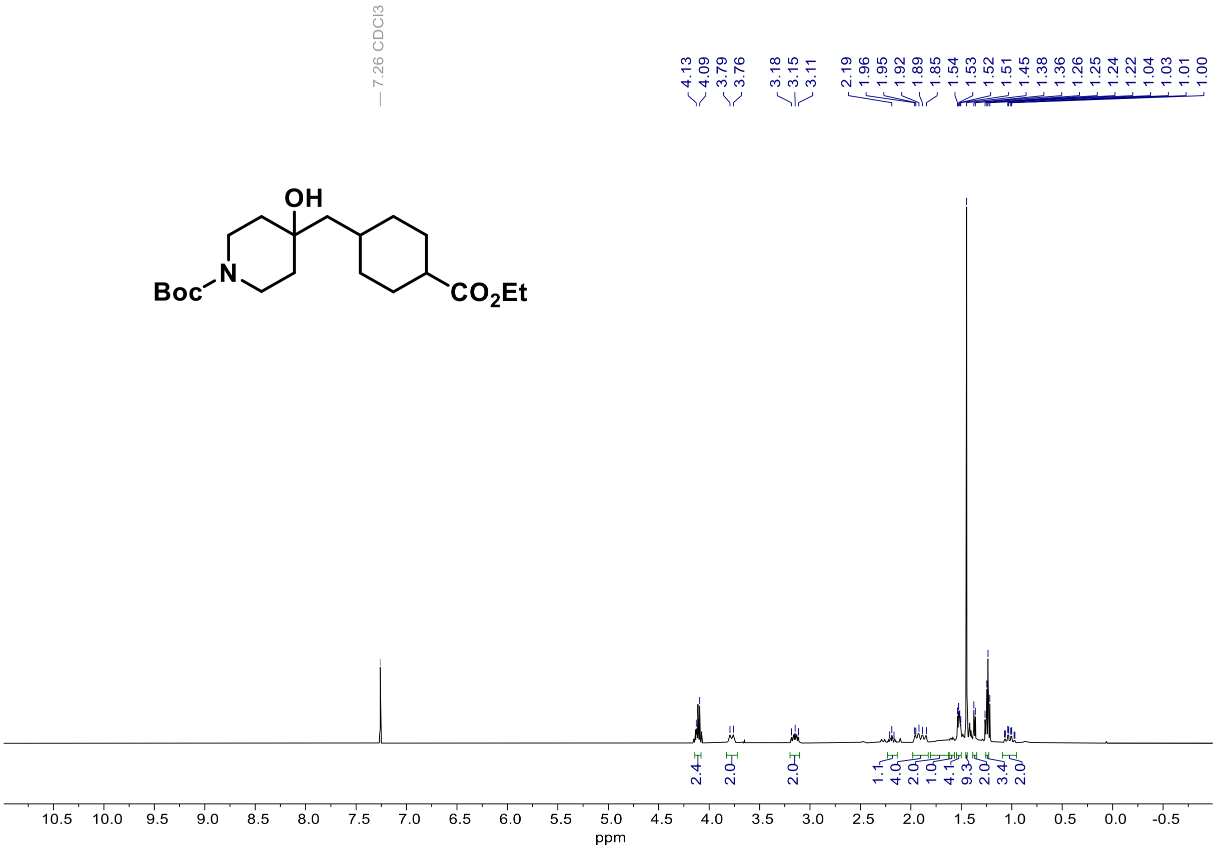

69 – ^{13}C NMR (101 MHz, CDCl$_3$)

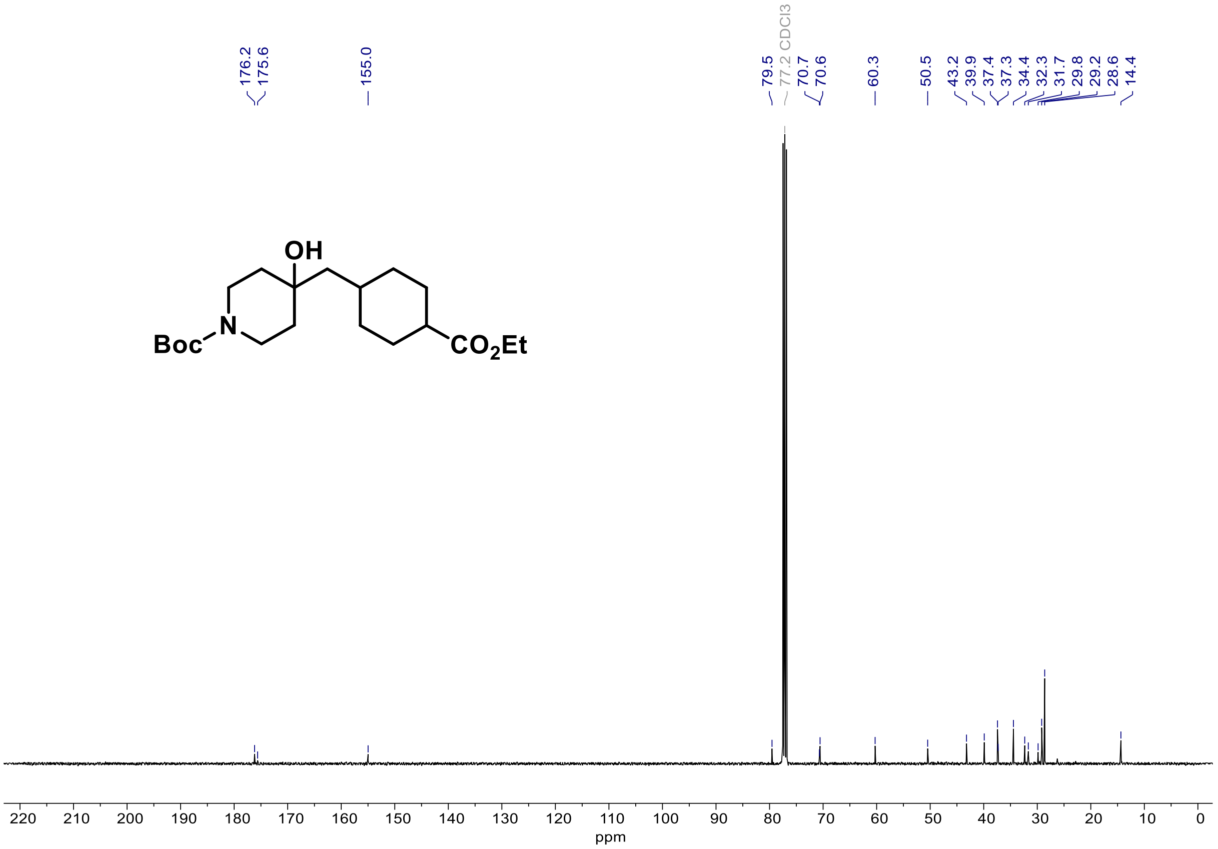

70 – ^{1}H NMR (400 MHz, CDCl$_3$)

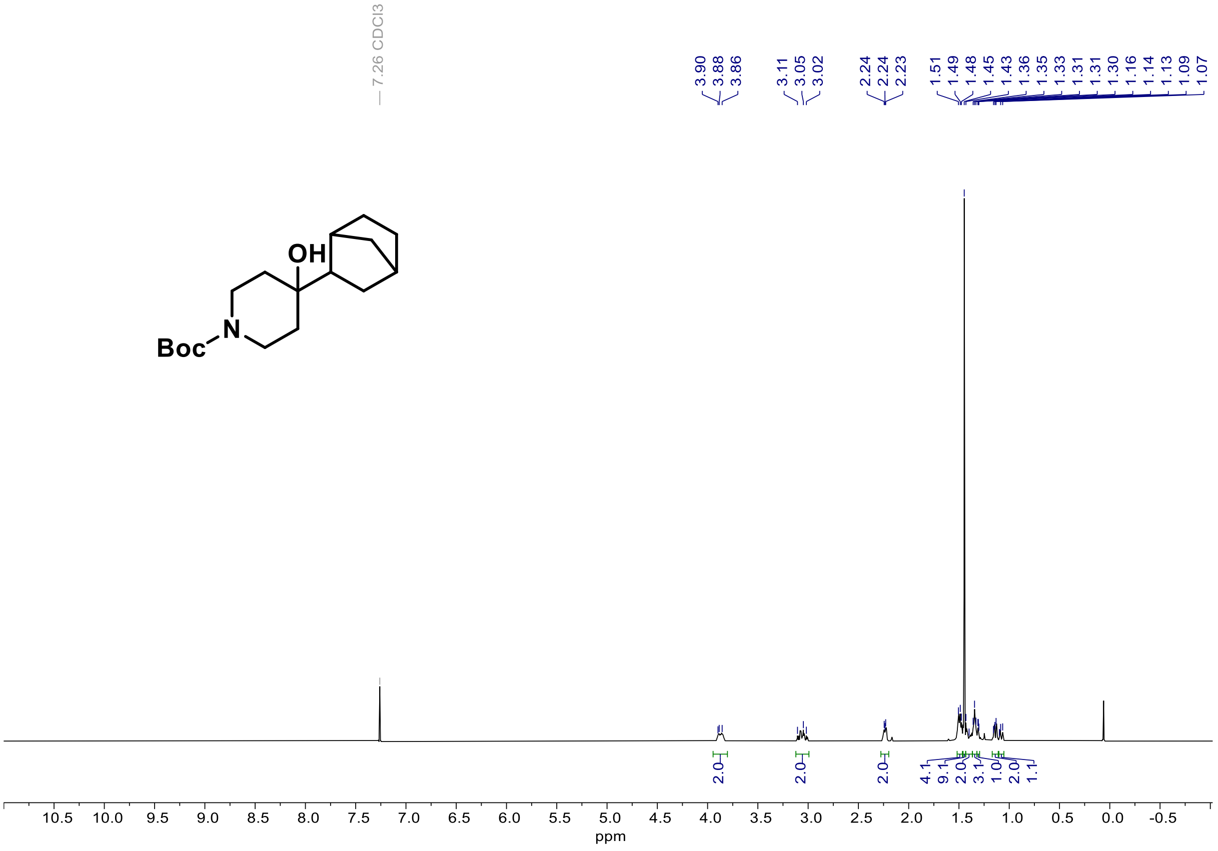

70 – ^{13}C NMR (101 MHz, $CDCl_3$)

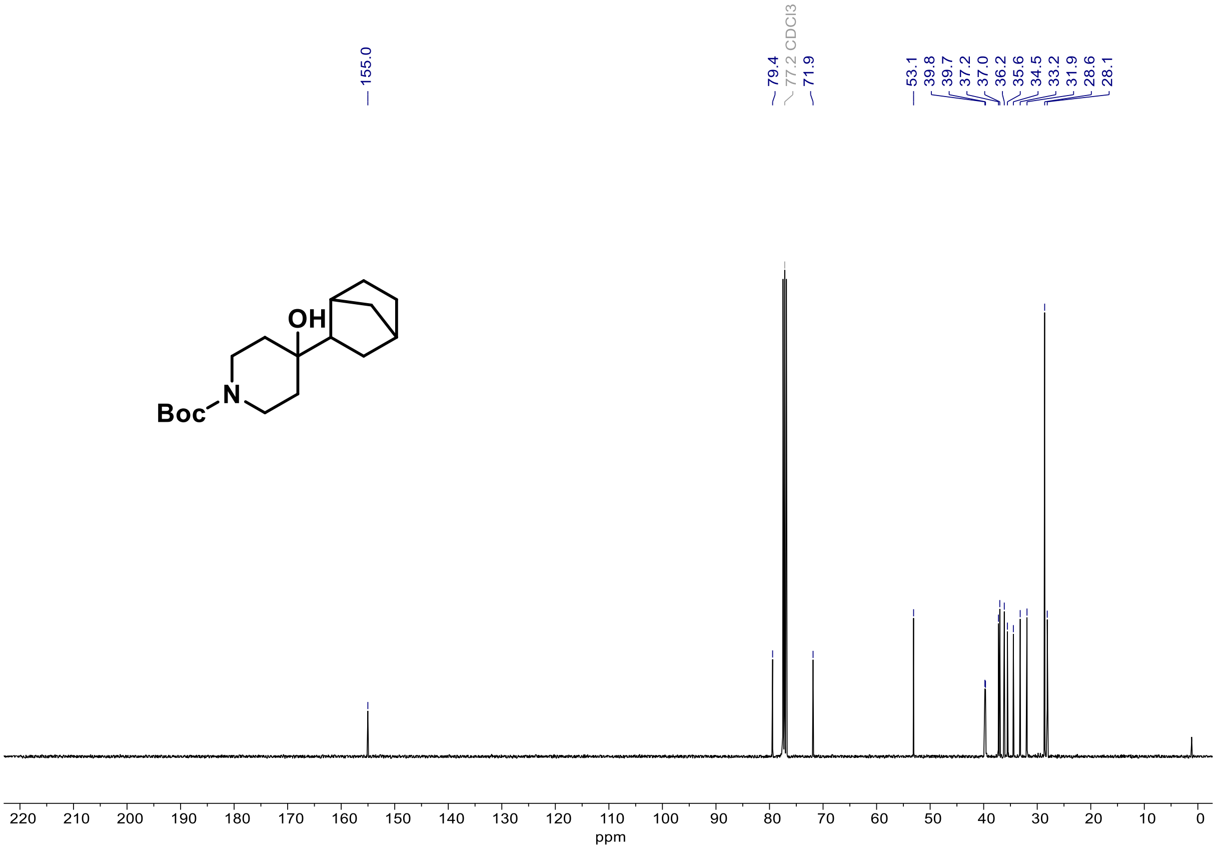

71 – ^{1}H NMR (400 MHz, $CDCl_3$)

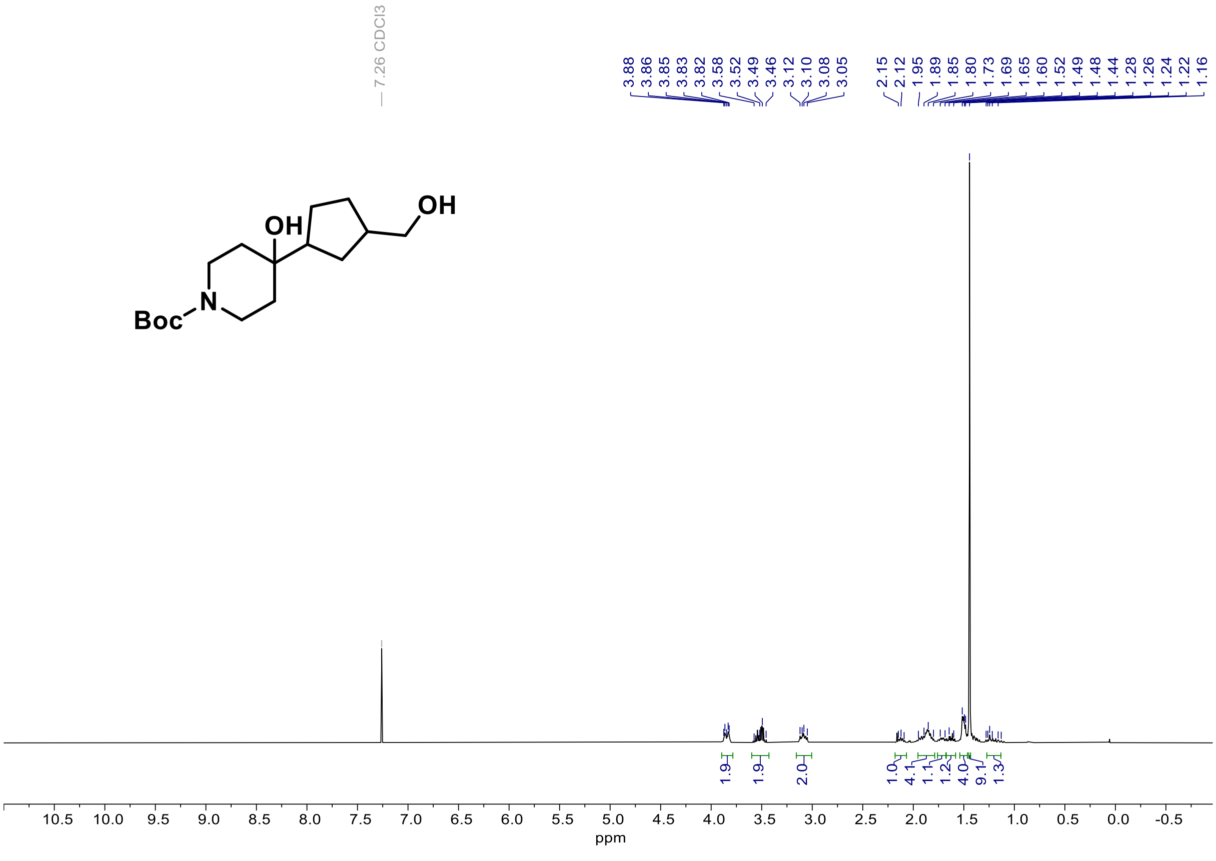

71 – ^{13}C NMR (101 MHz, $CDCl_3$)

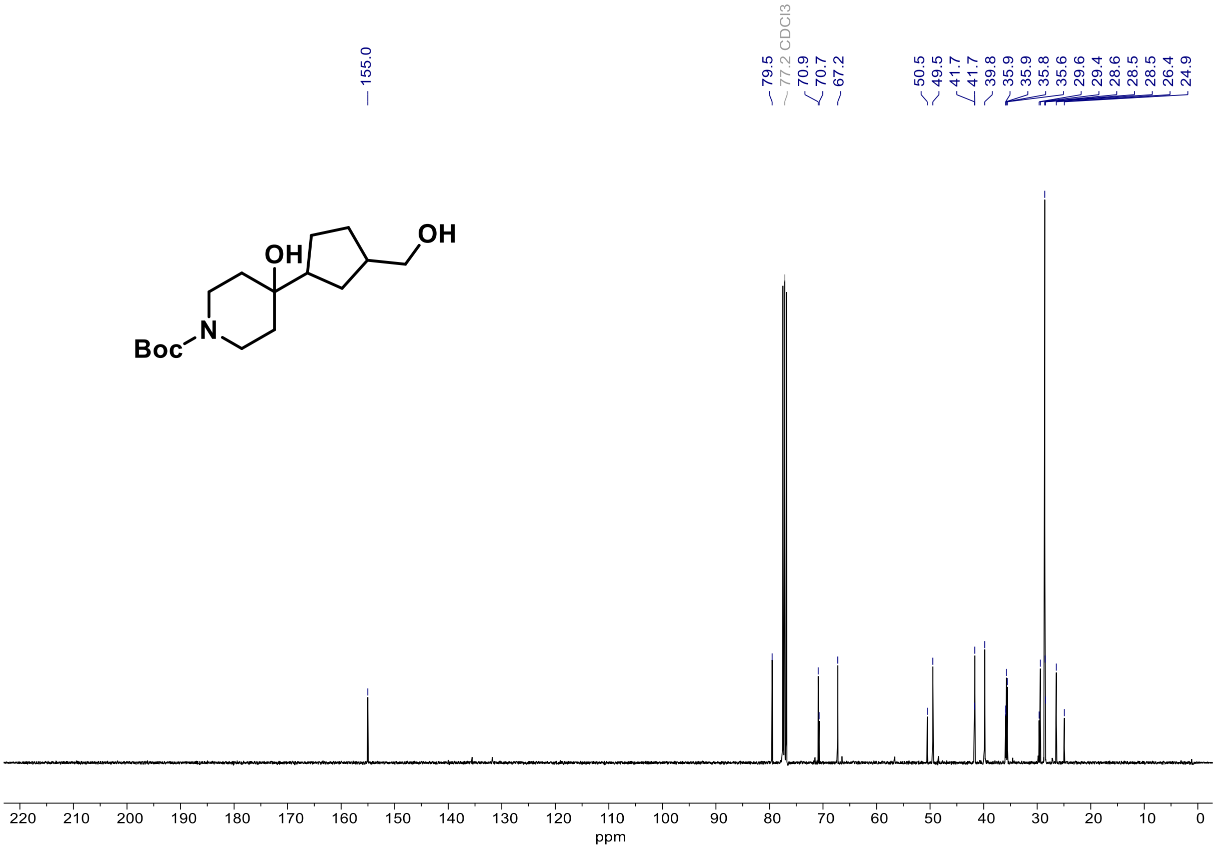

72 – ^{1}H NMR (400 MHz, $CDCl_3$)

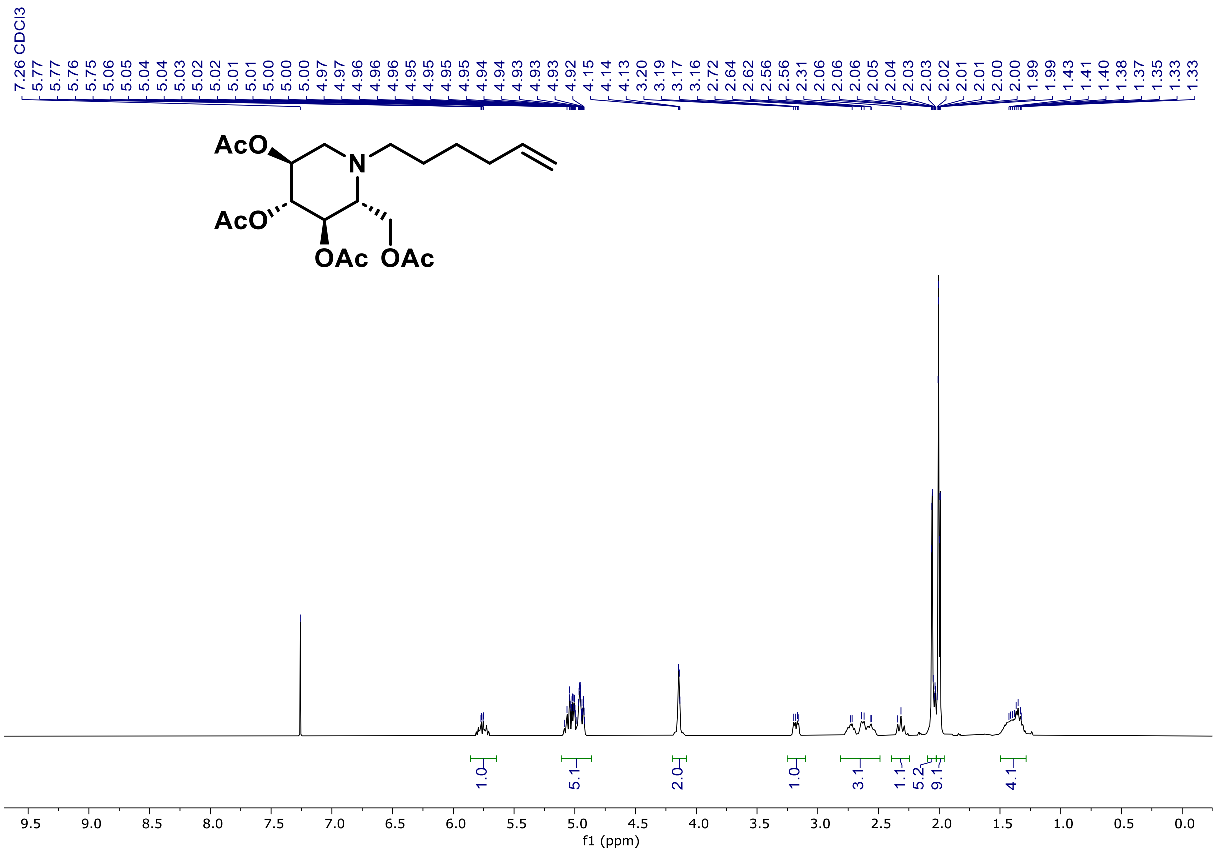

72 – ^{13}C NMR (101 MHz, $CDCl_3$)

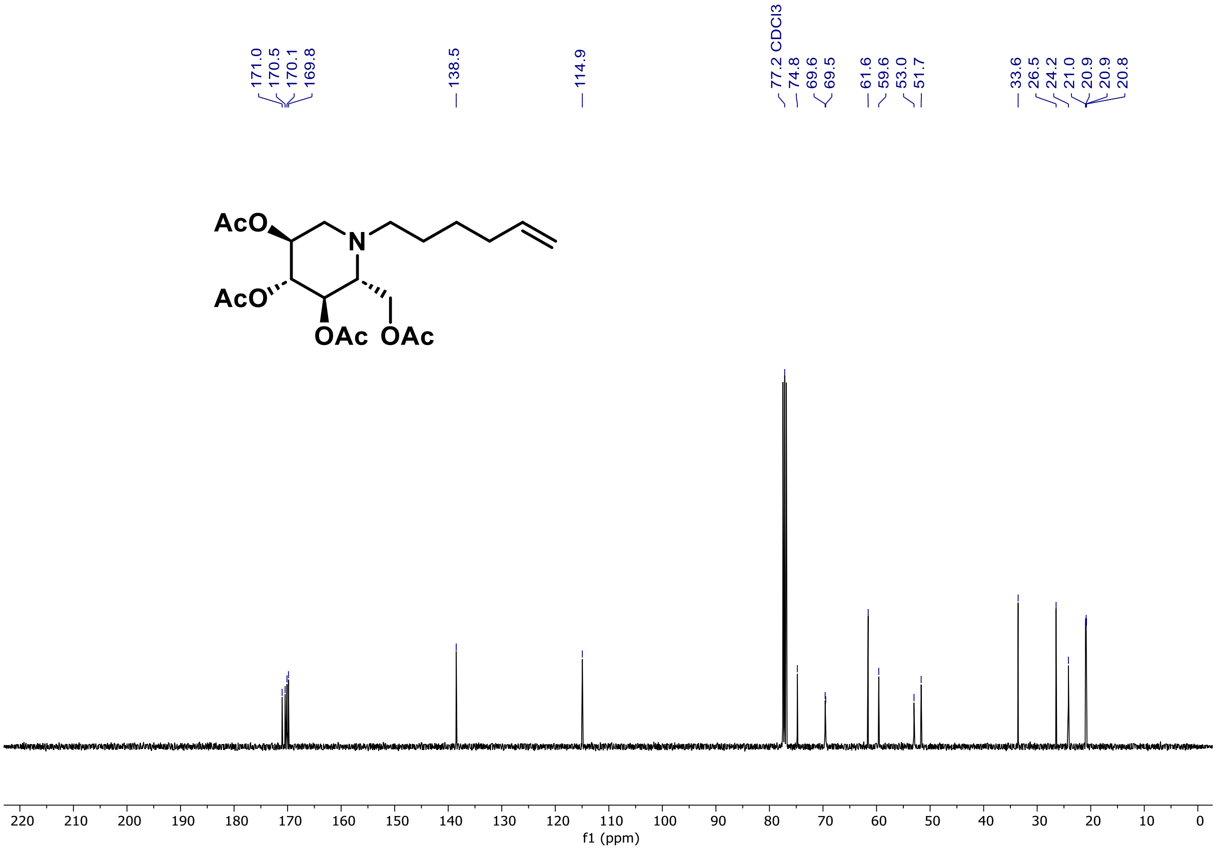

73 – ^{1}H NMR (400 MHz, $CDCl_3$)

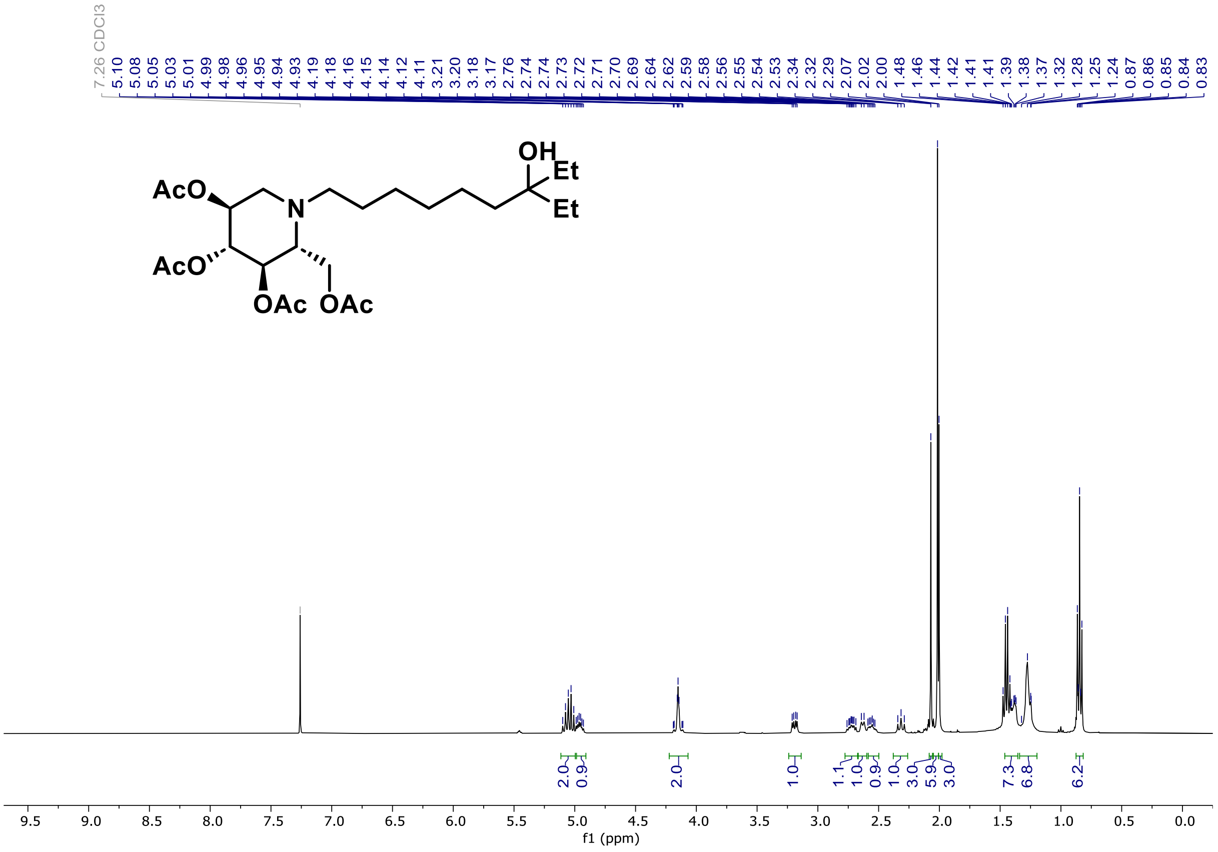

73 – ^{13}C NMR (101 MHz, $CDCl_3$)

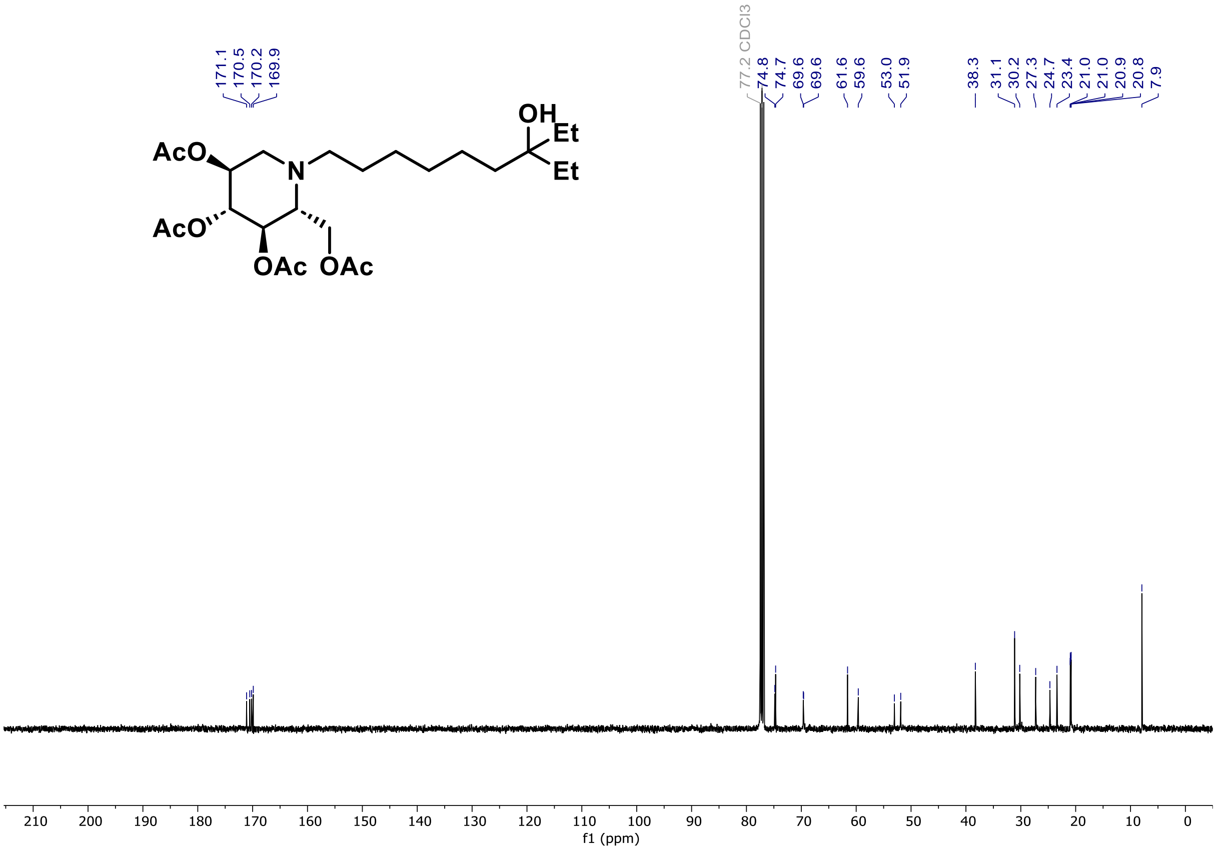

74 – ^{1}H NMR (400 MHz, $CDCl_3$)

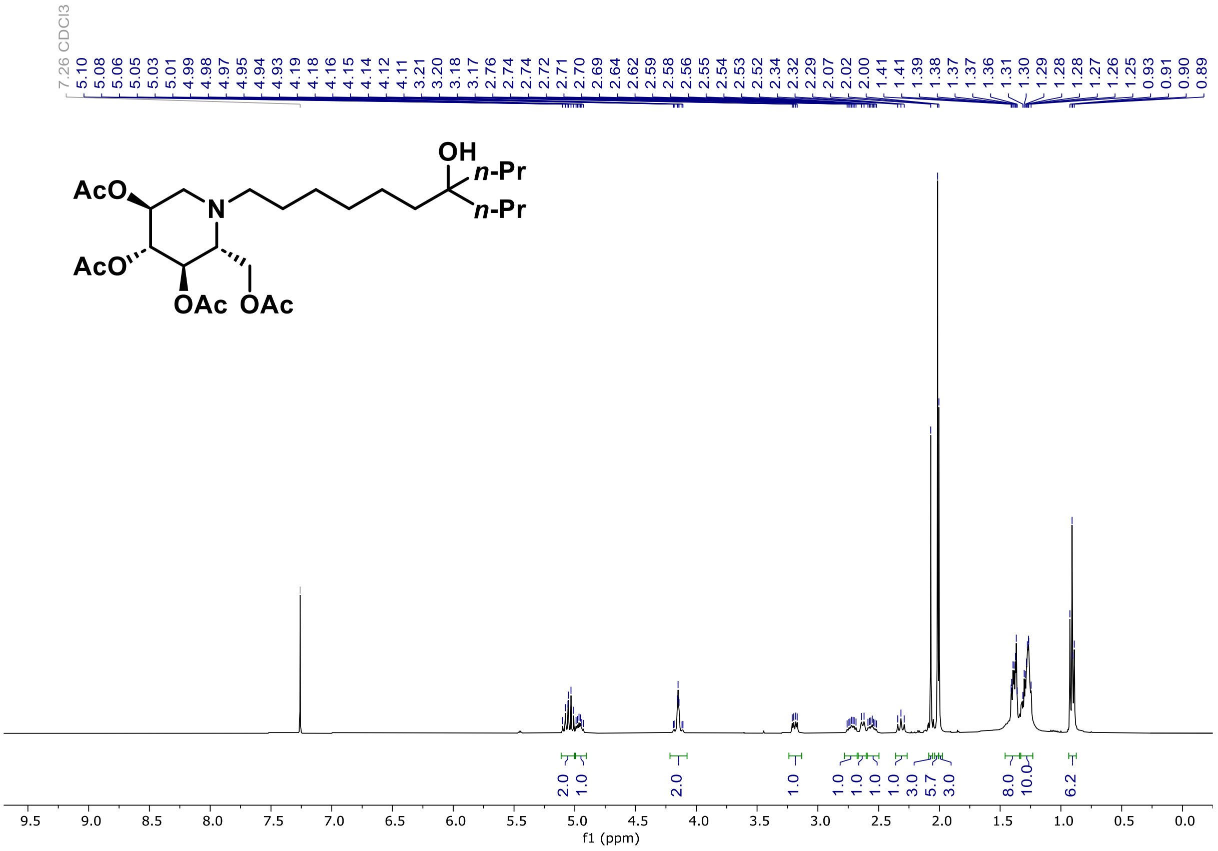

74 – ^{13}C NMR (101 MHz, CDCl$_3$)

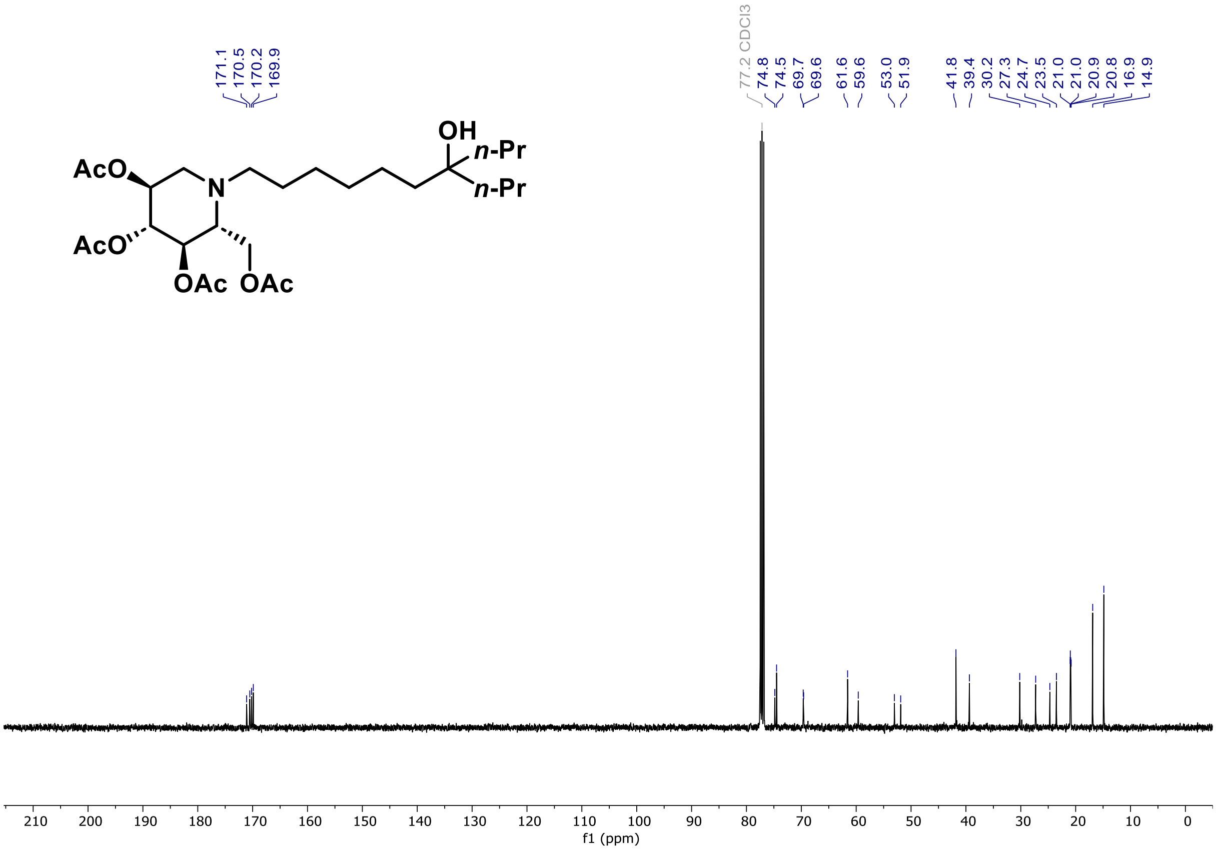

75 – ^{1}H NMR (400 MHz, CDCl$_3$)

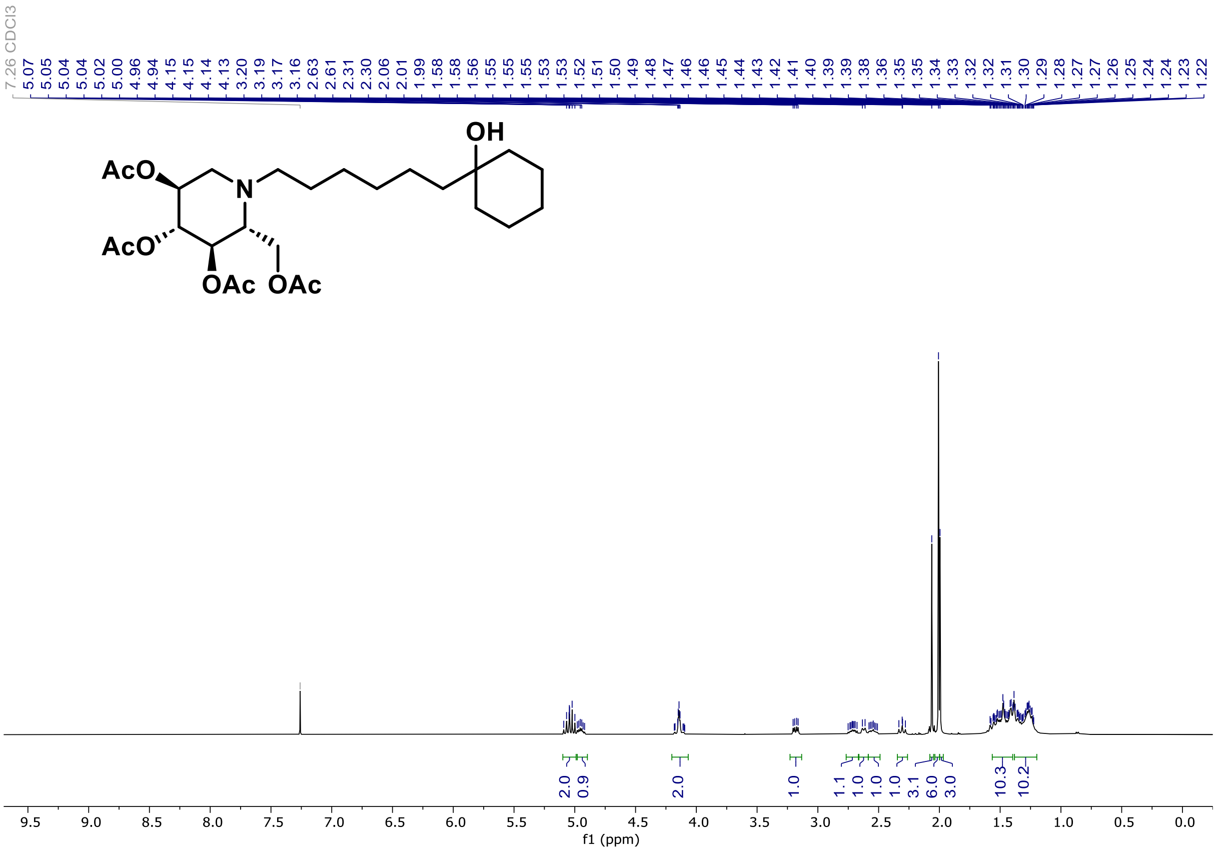

75 – ^{13}C NMR (101 MHz, CDCl$_3$)

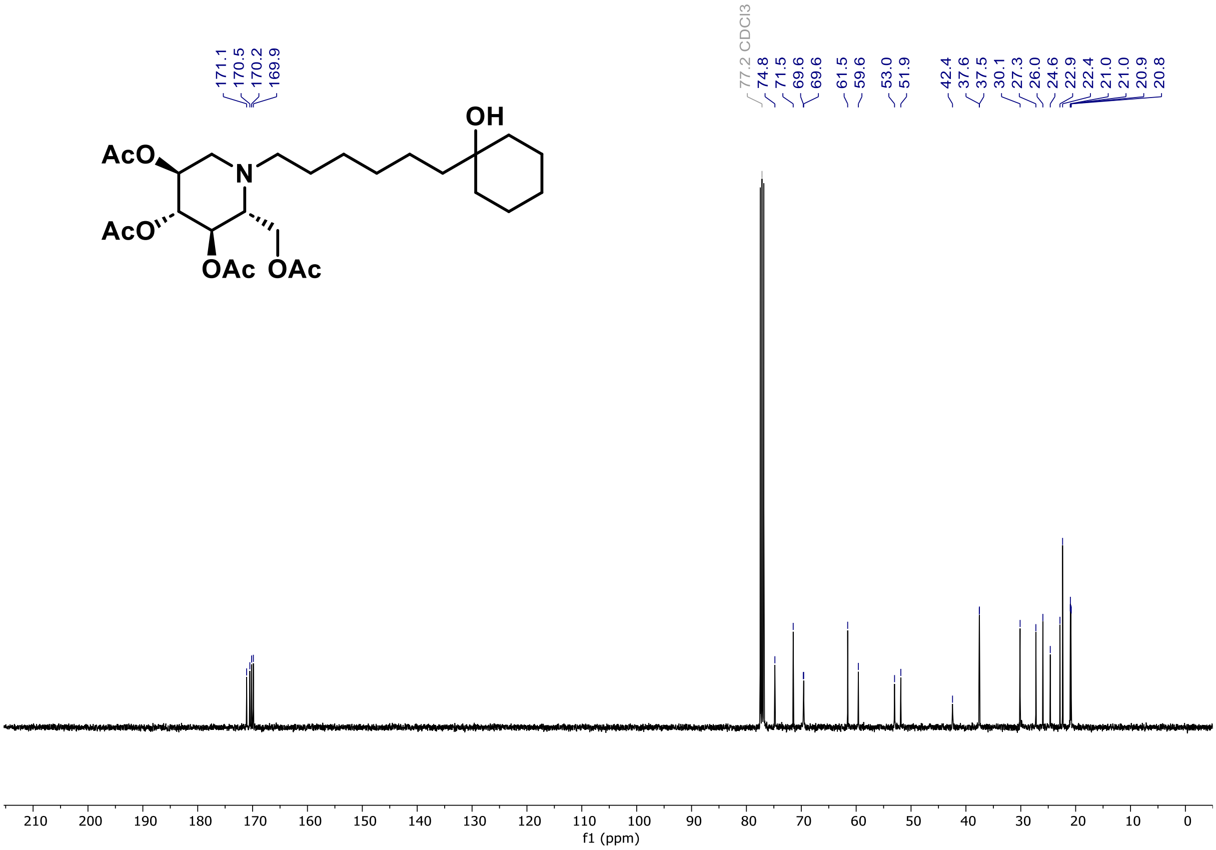

76 – ^{1}H NMR (400 MHz, CDCl$_3$)

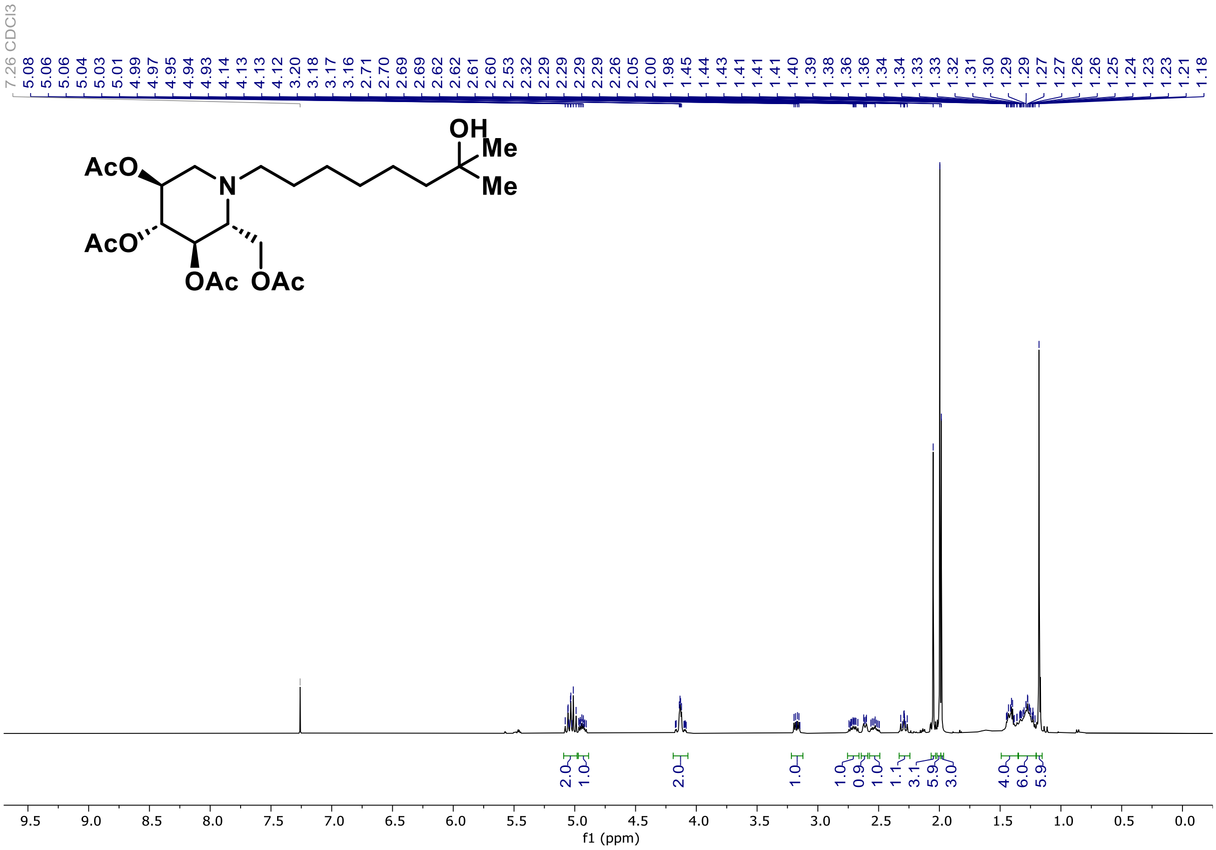

76 – ^{13}C NMR (101 MHz, $CDCl_3$)

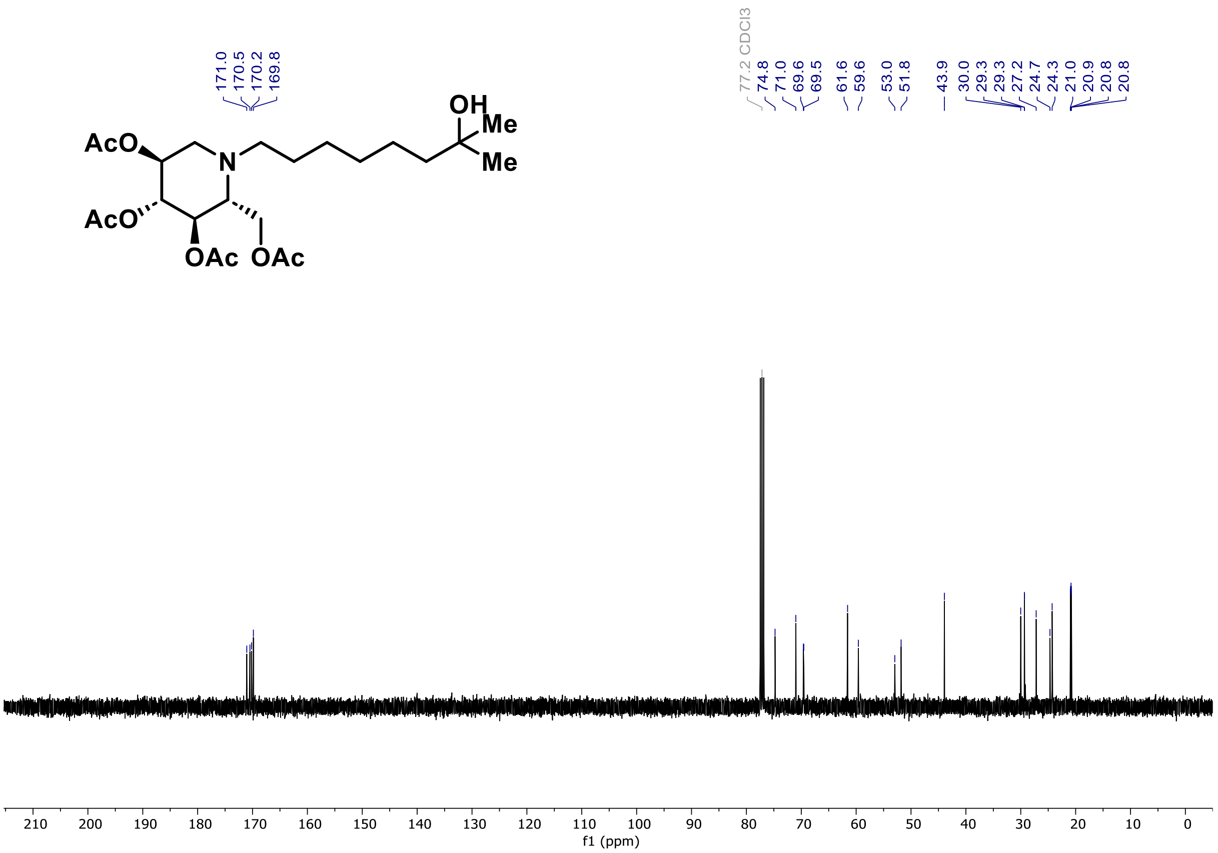

77 – ^{1}H NMR (400 MHz, $CDCl_3$)

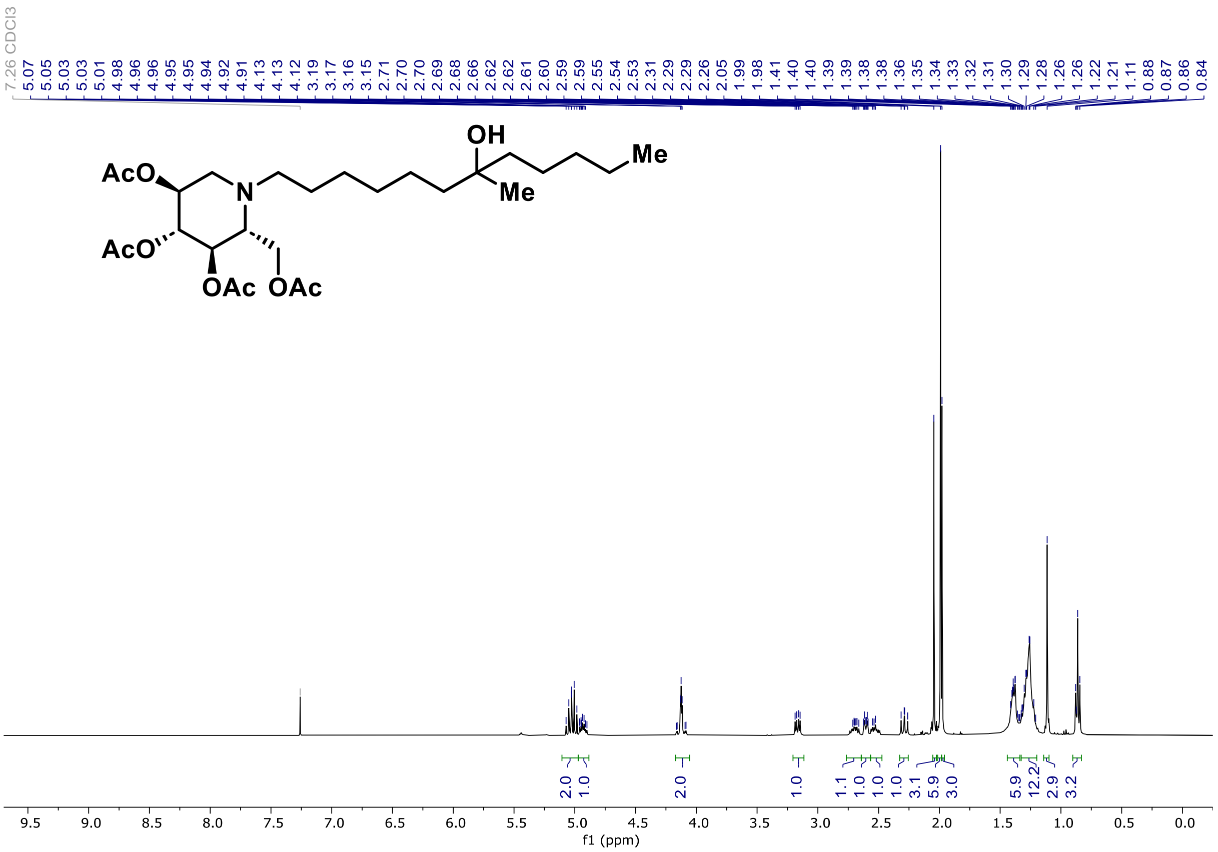

77 – ^{13}C NMR (101 MHz, $CDCl_3$)

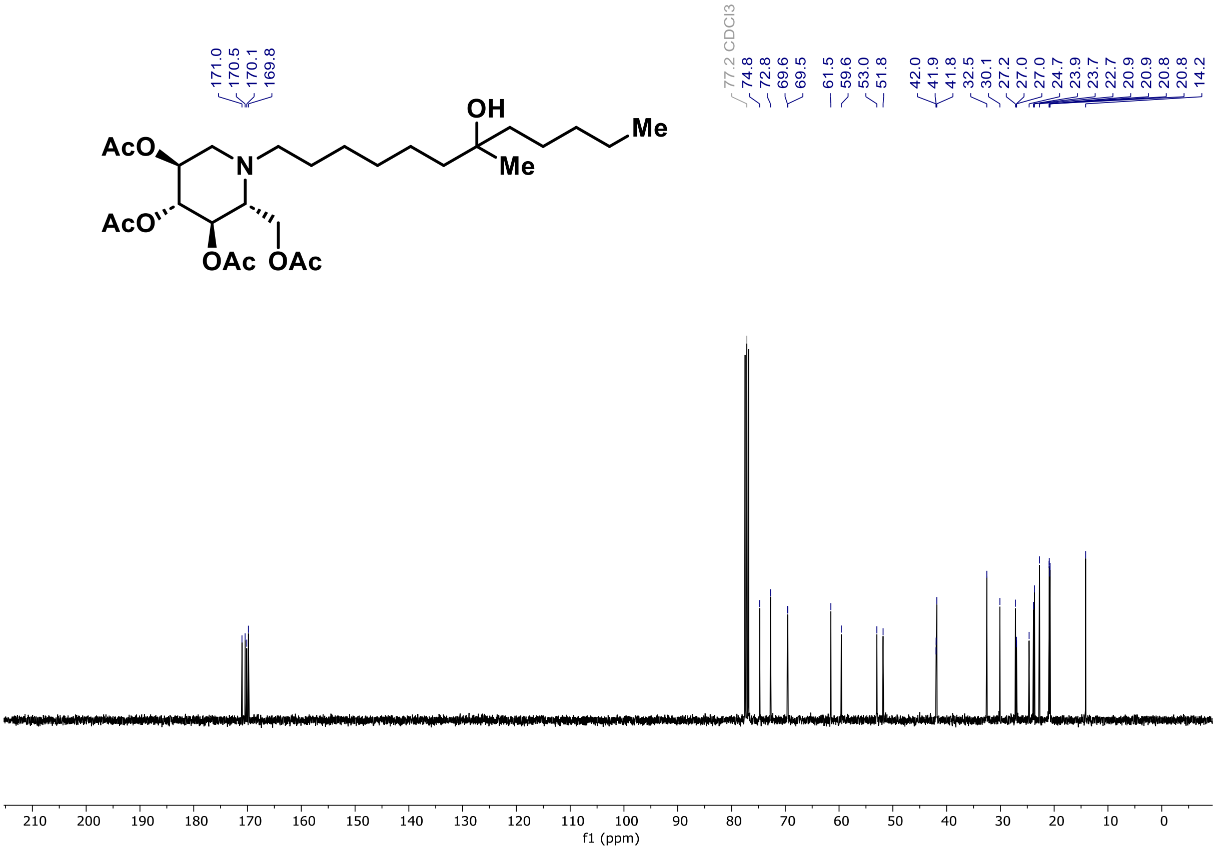

78 – ^{1}H NMR (400 MHz, $CDCl_3$)

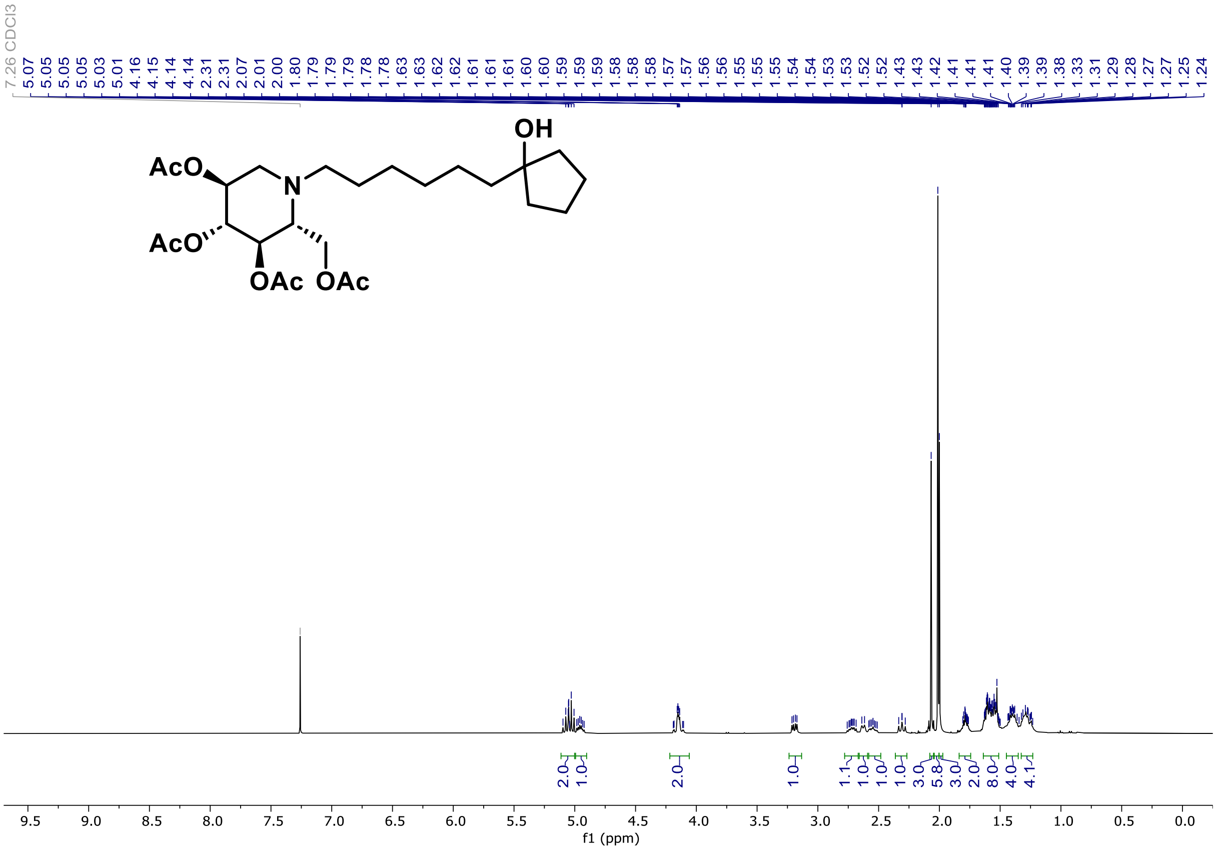

78 – ^{13}C NMR (101 MHz, CDCl$_3$)

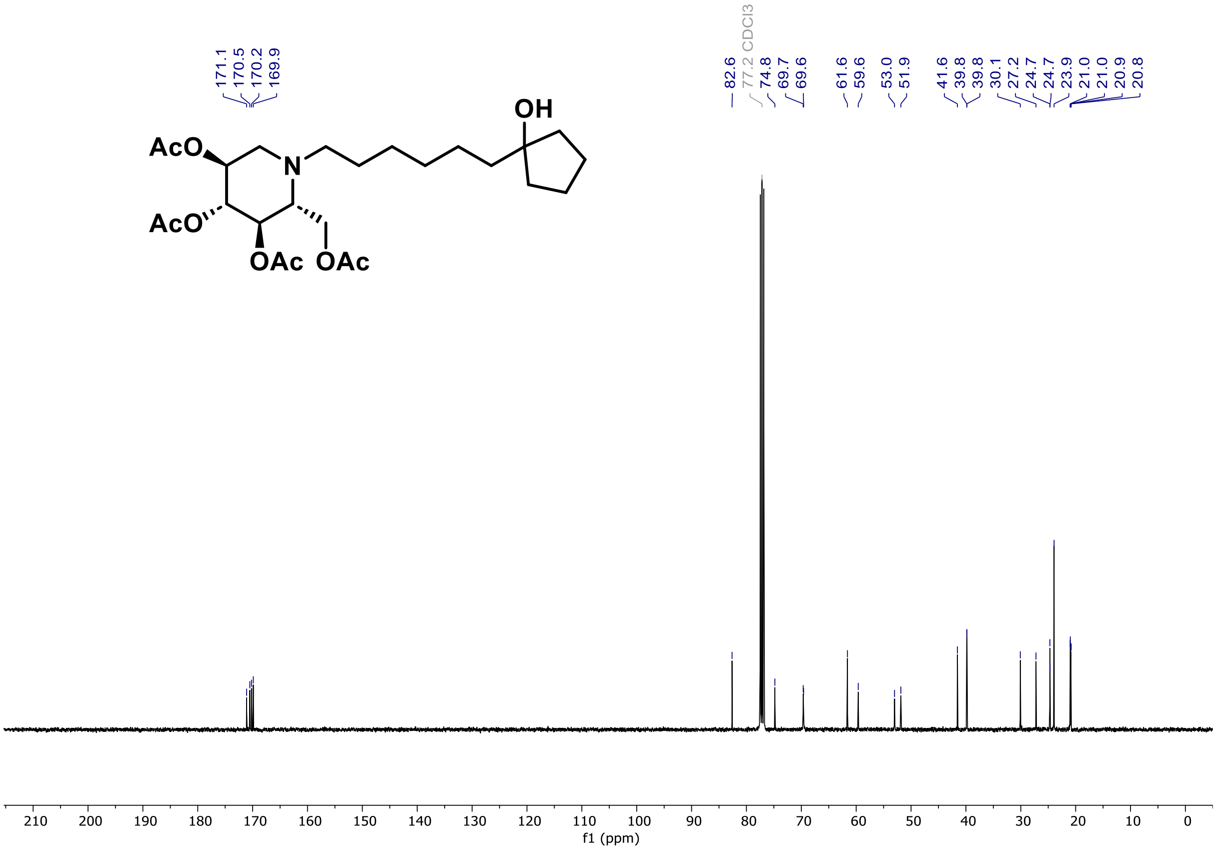

79 – ^{1}H NMR (400 MHz, CDCl$_3$)

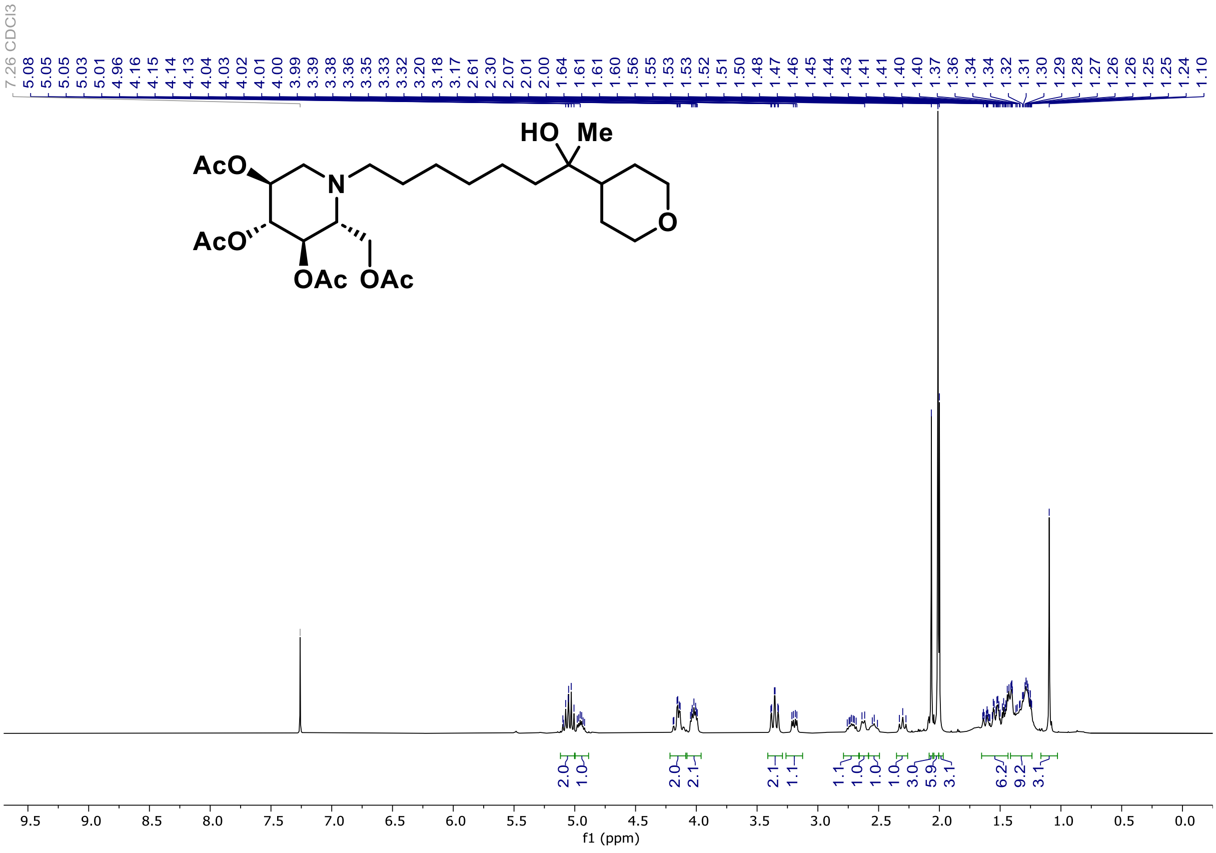

79 – ^{13}C NMR (101 MHz, CDCl$_3$)

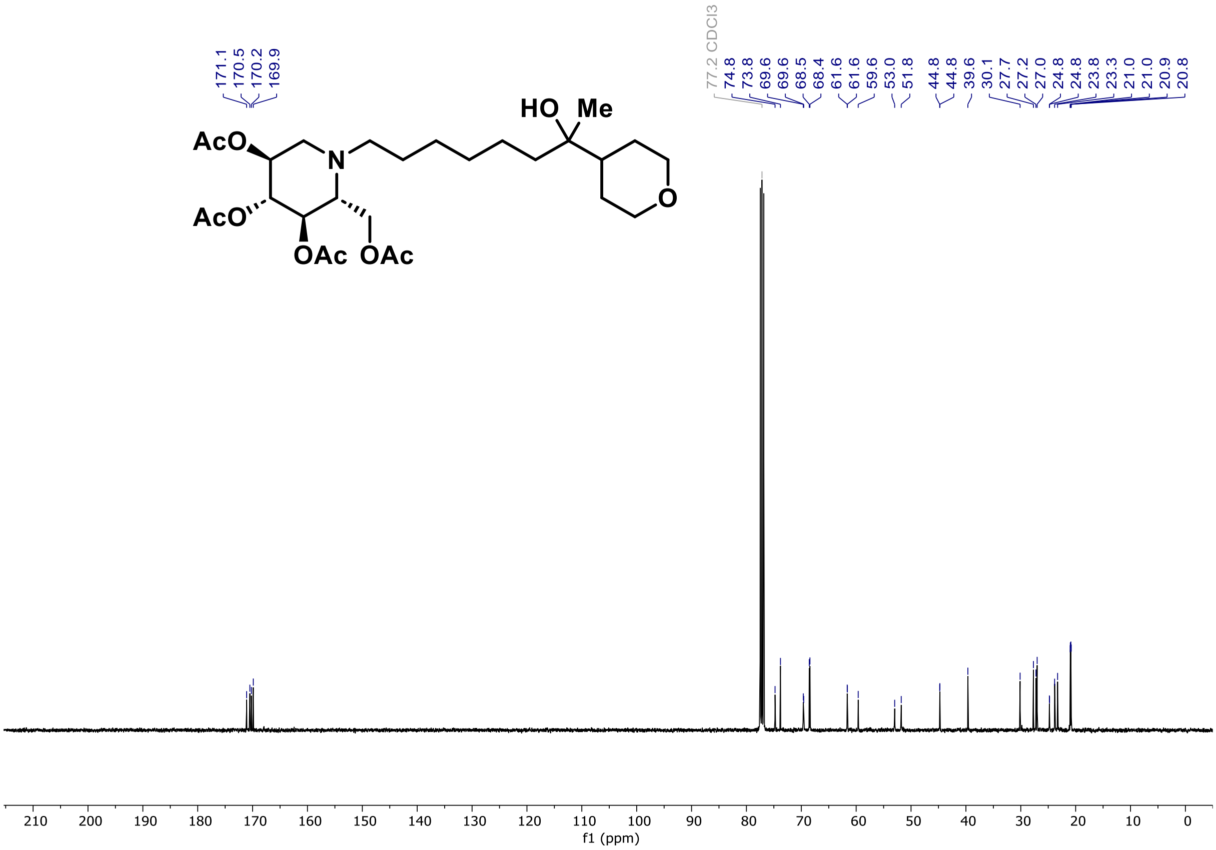

80 – ^{1}H NMR (400 MHz, CDCl$_3$)

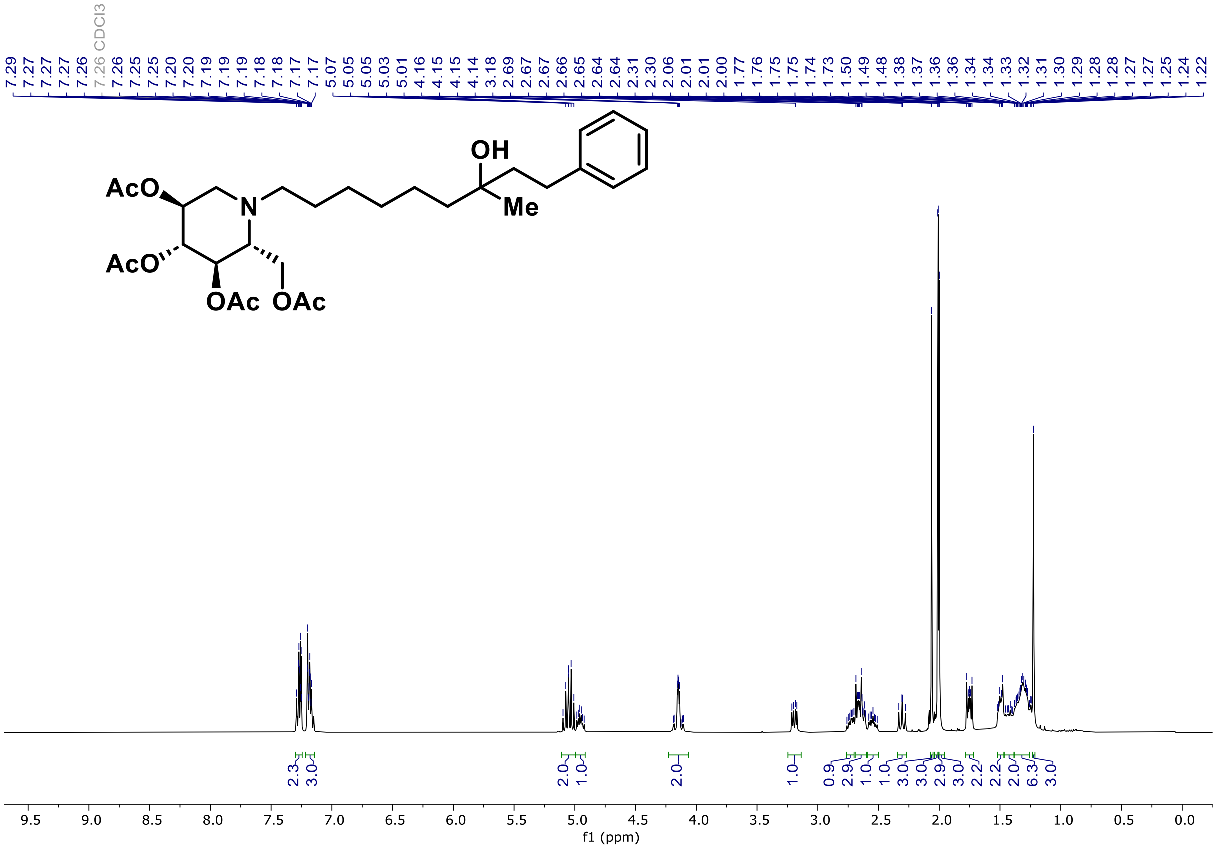

80 – ^{13}C NMR (101 MHz, CDCl$_3$)

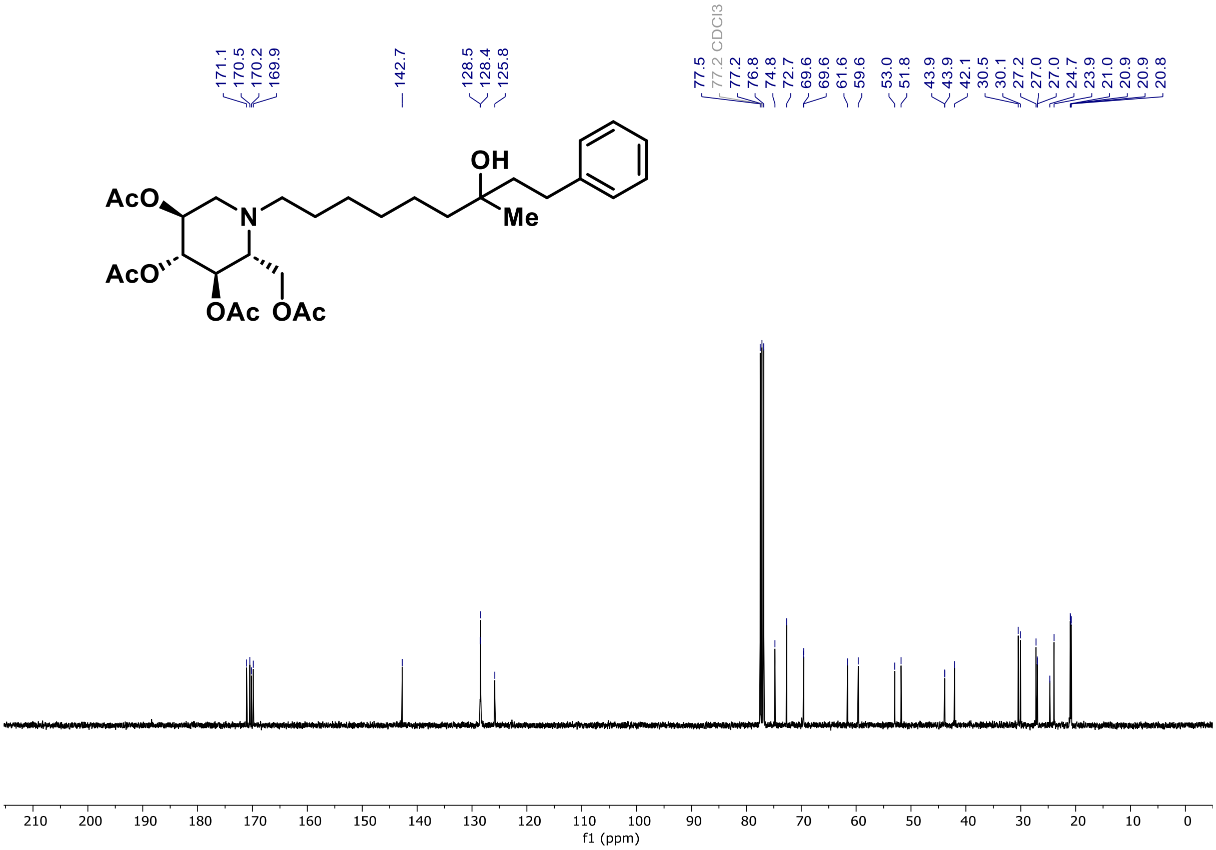

81 – ^{1}H NMR (400 MHz, CDCl$_3$)

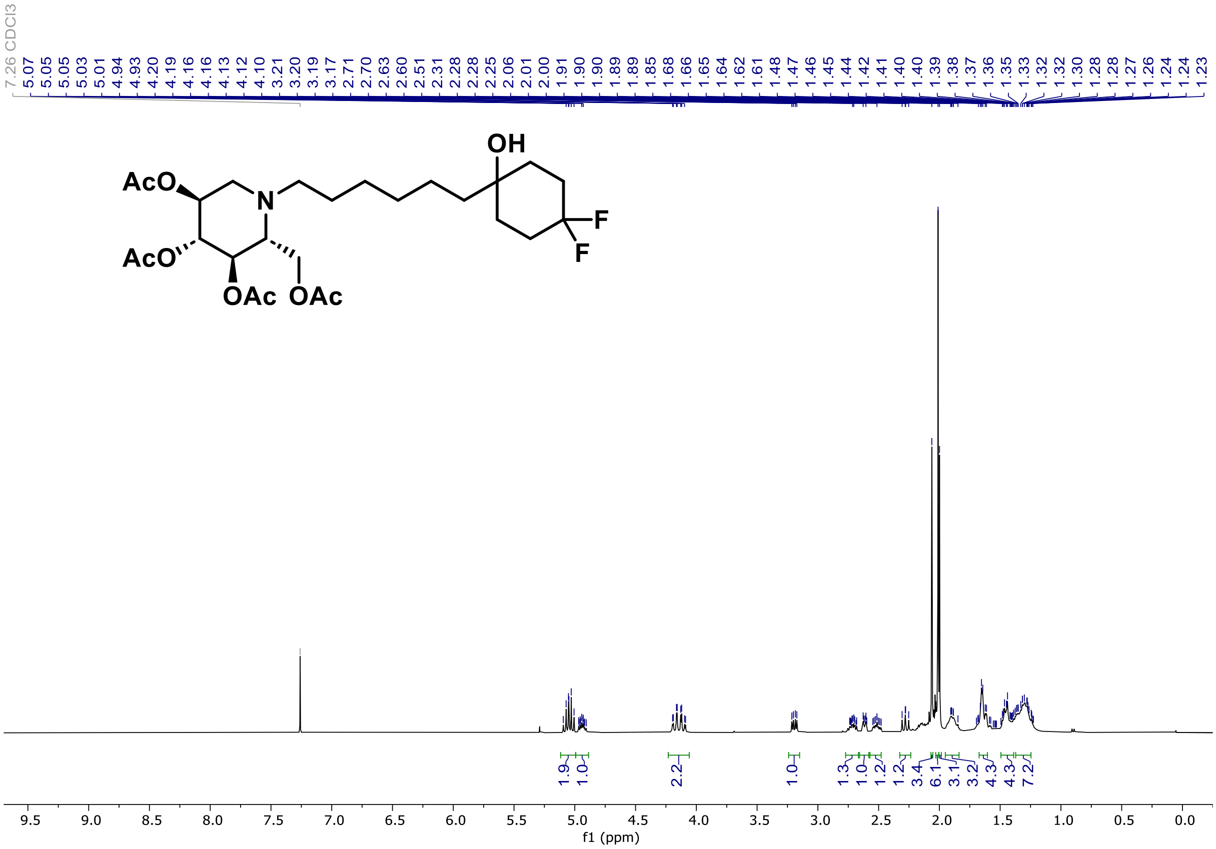

81 – ^{13}C NMR (101 MHz, $CDCl_3$)

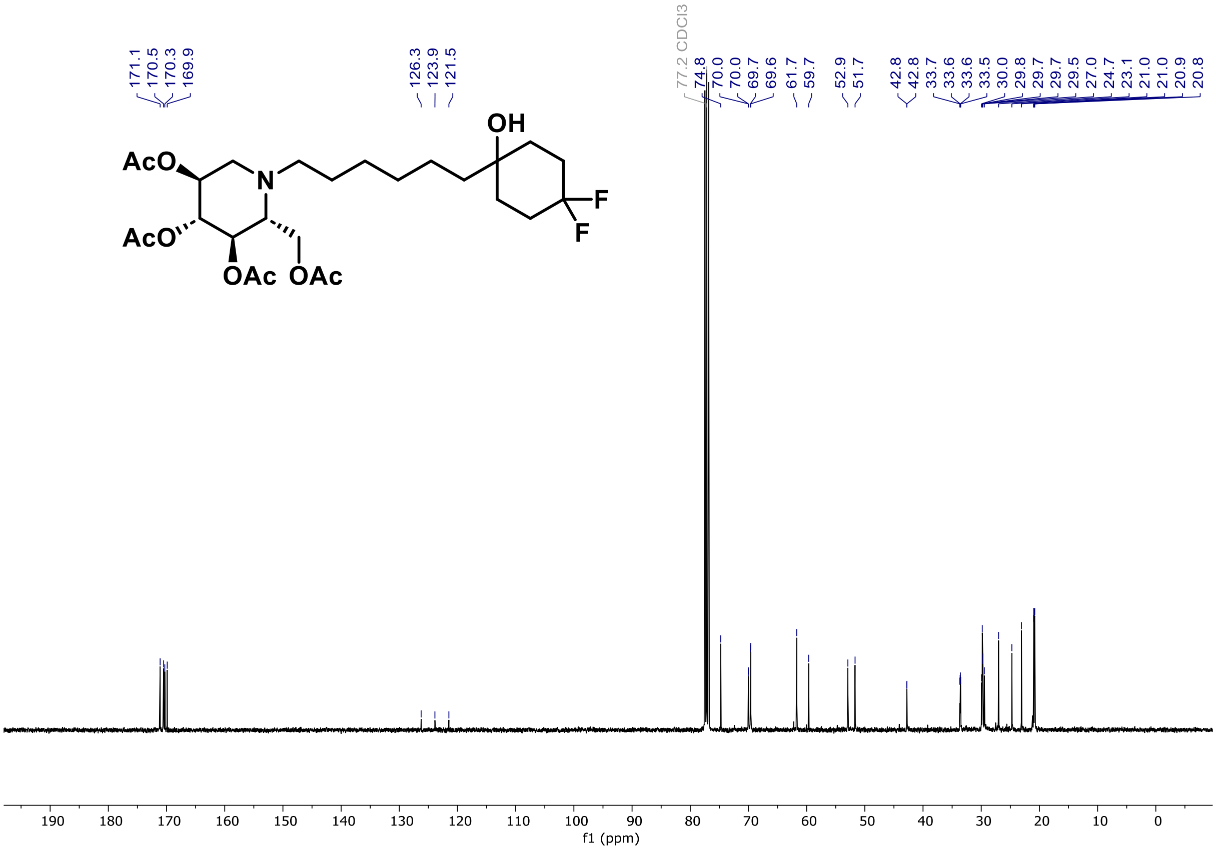

81 – ^{19}F NMR (377 MHz, $CDCl_3$)

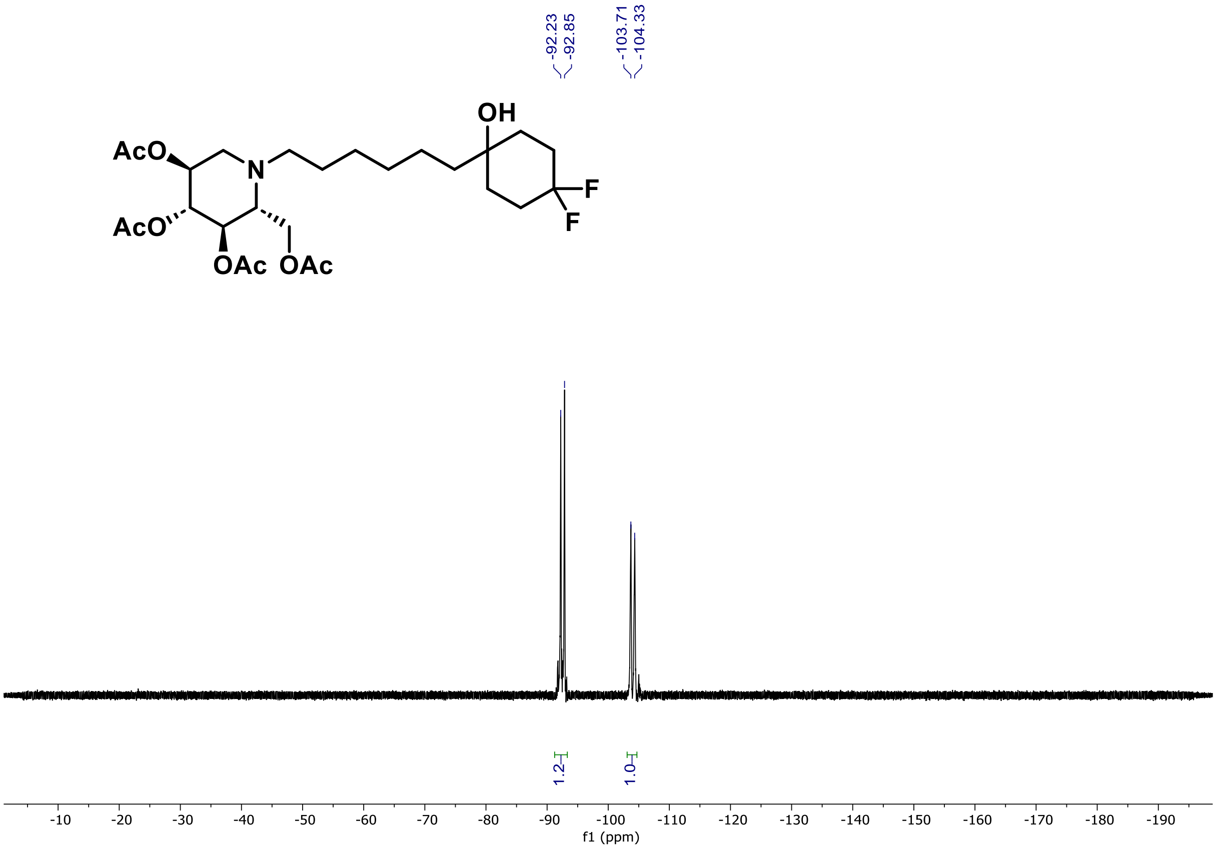

82 – ^{1}H NMR (400 MHz, $CDCl_3$)

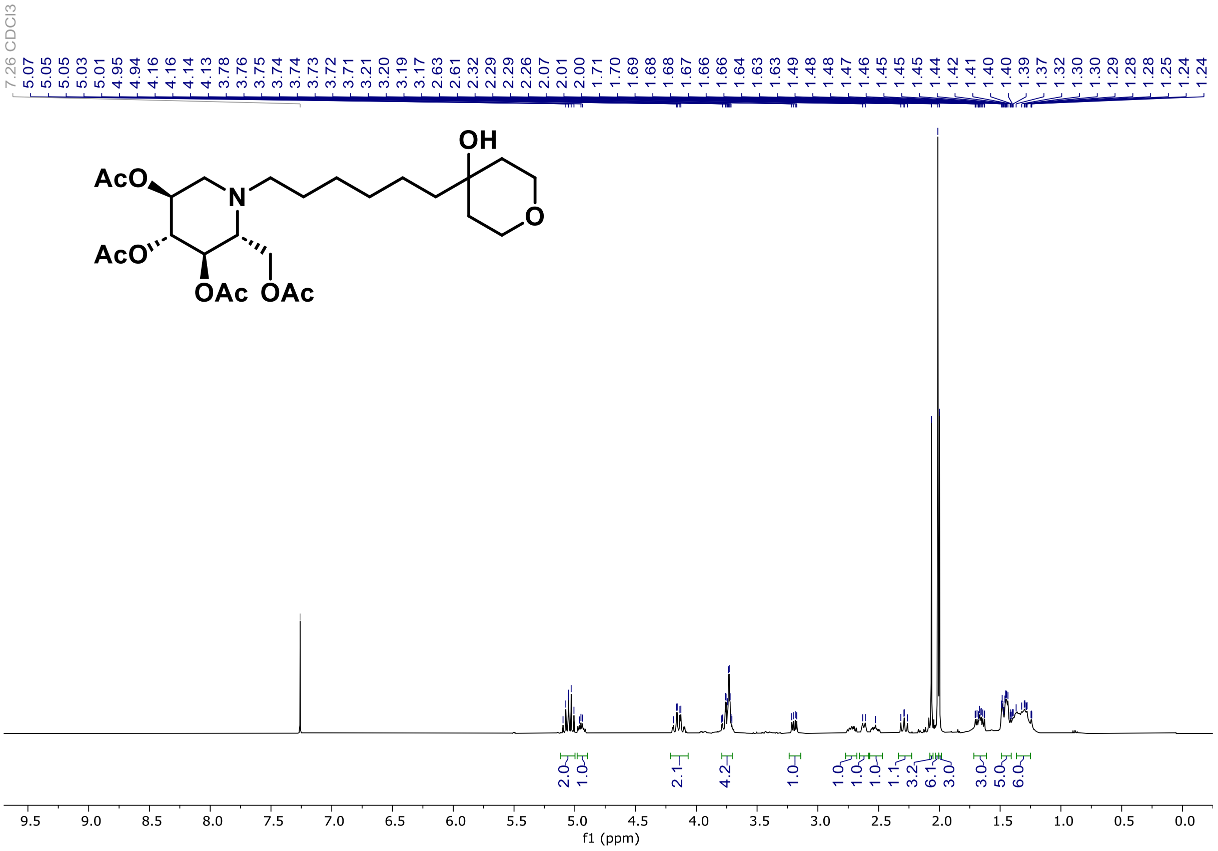

82 – ^{13}C NMR (101 MHz, $CDCl_3$)

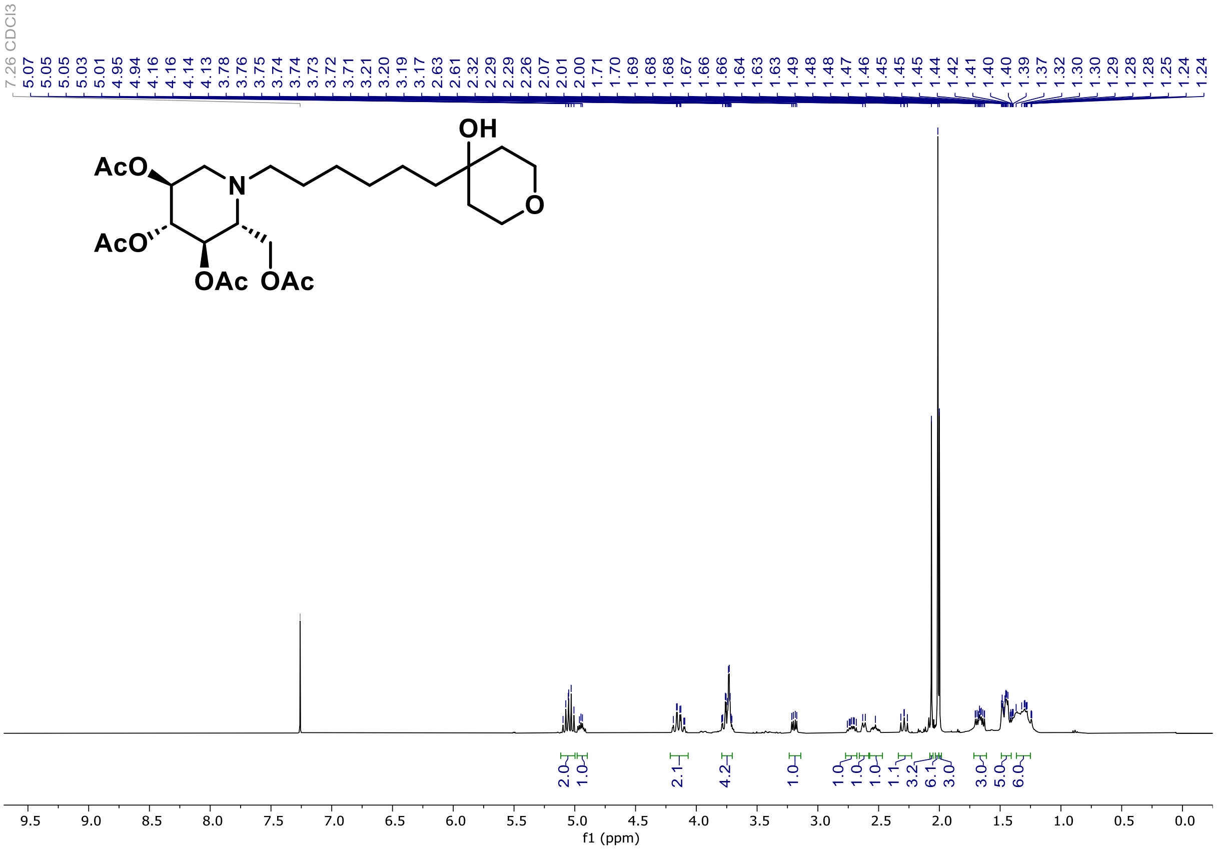

83 – ^{1}H NMR (400 MHz, $CDCl_3$)

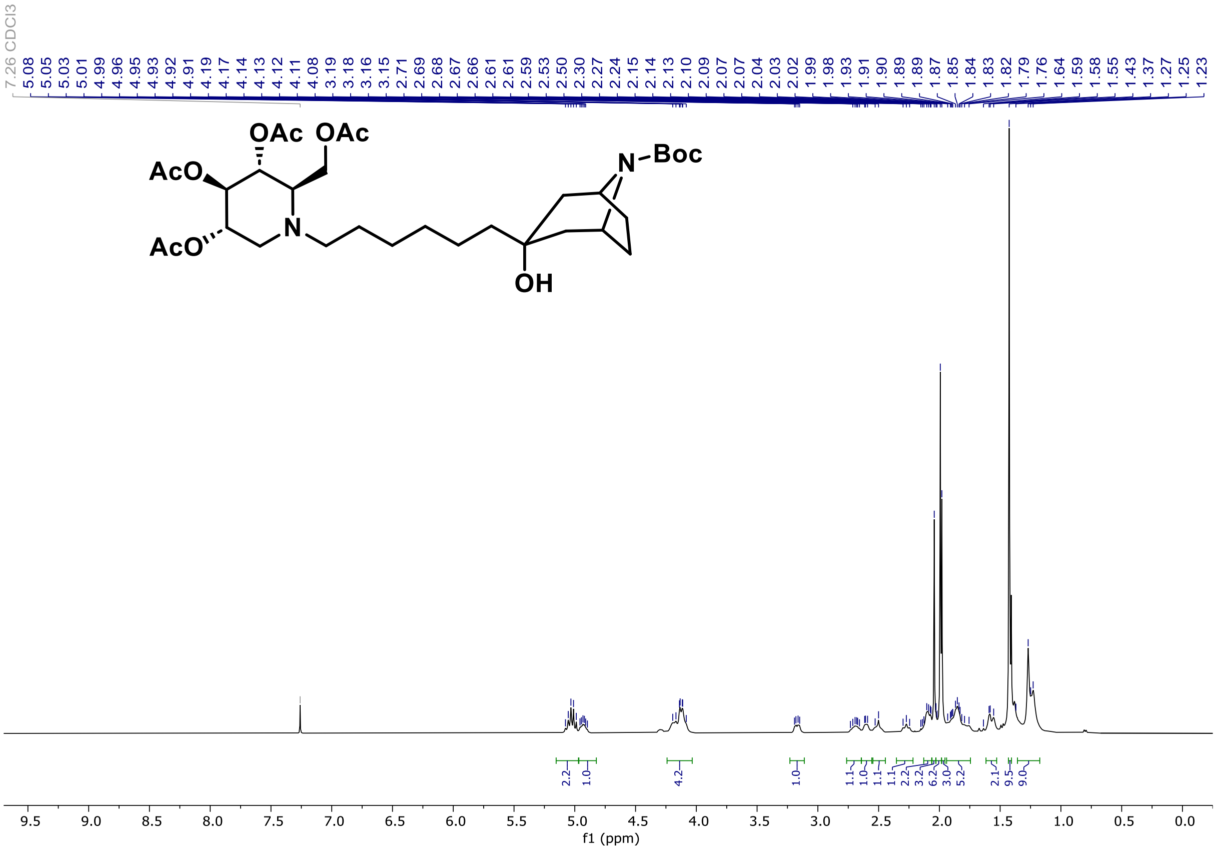

83 – ^{13}C NMR (101 MHz, $CDCl_3$)

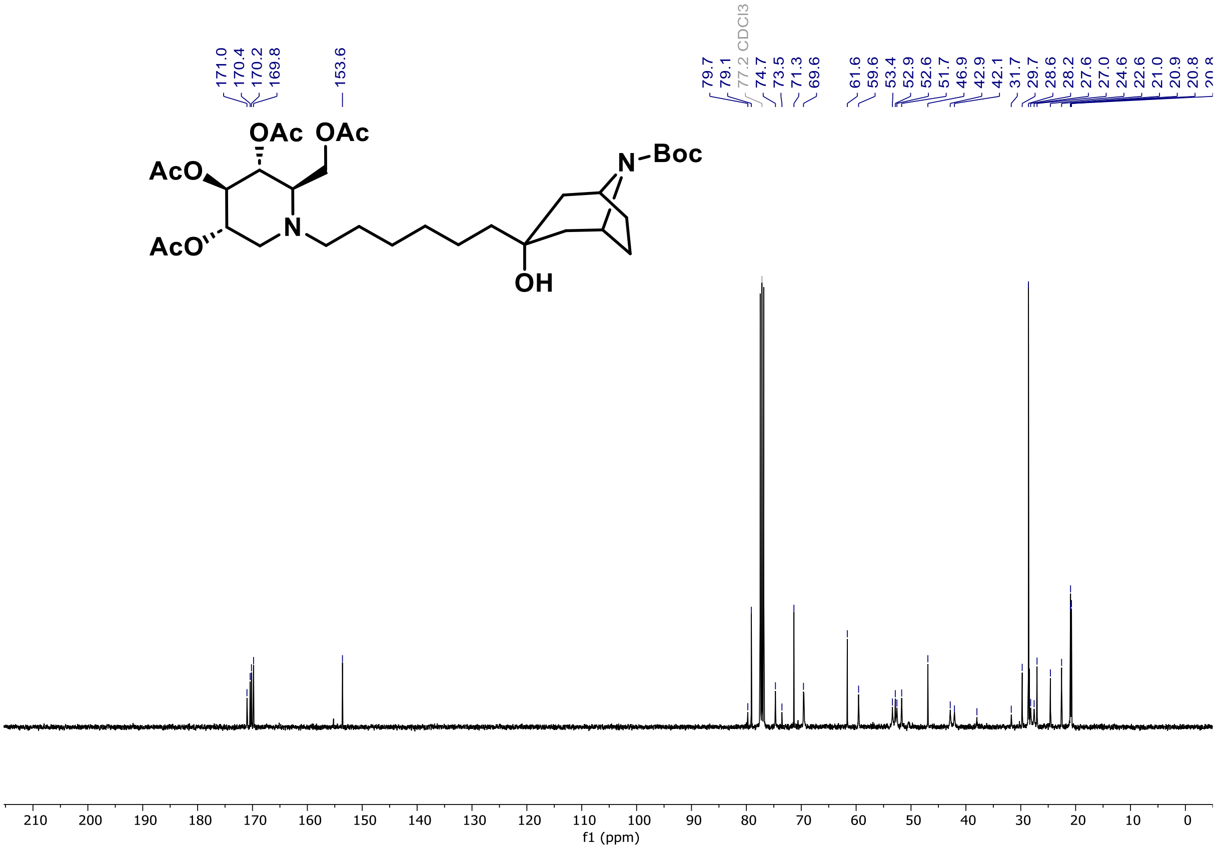

S2 – ^{1}H NMR (400 MHz, CDCl$_3$)

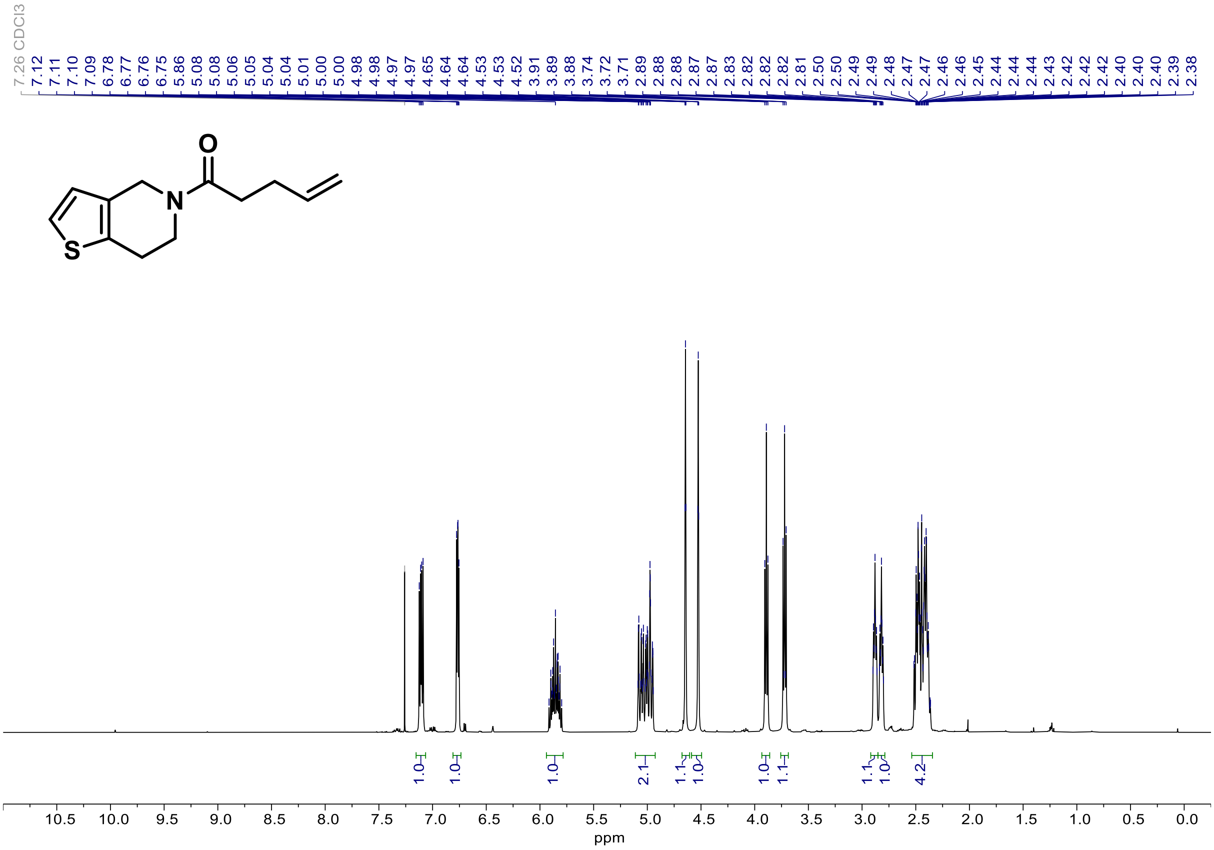

S2 – ^{13}C NMR (101 MHz, CDCl$_3$)

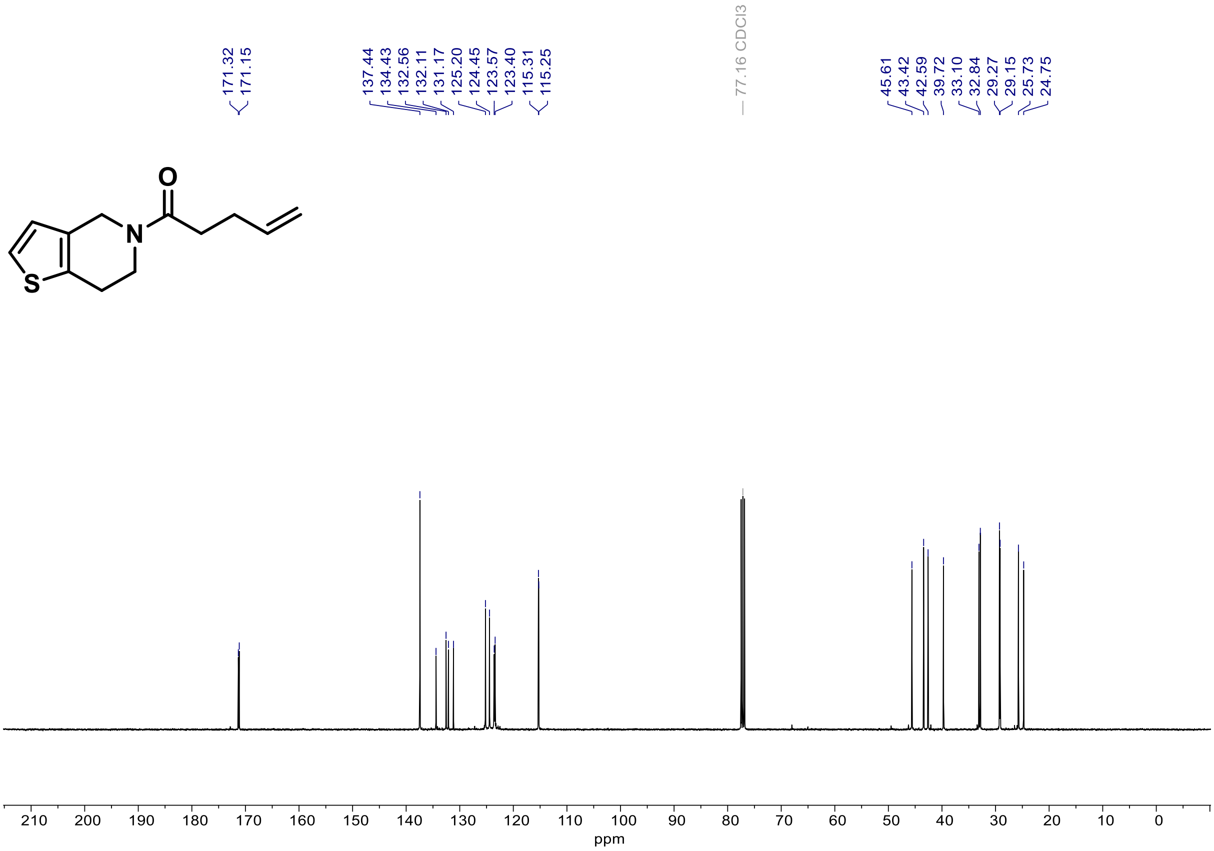

S3 – ^{1}H NMR (400 MHz, CDCl$_3$)

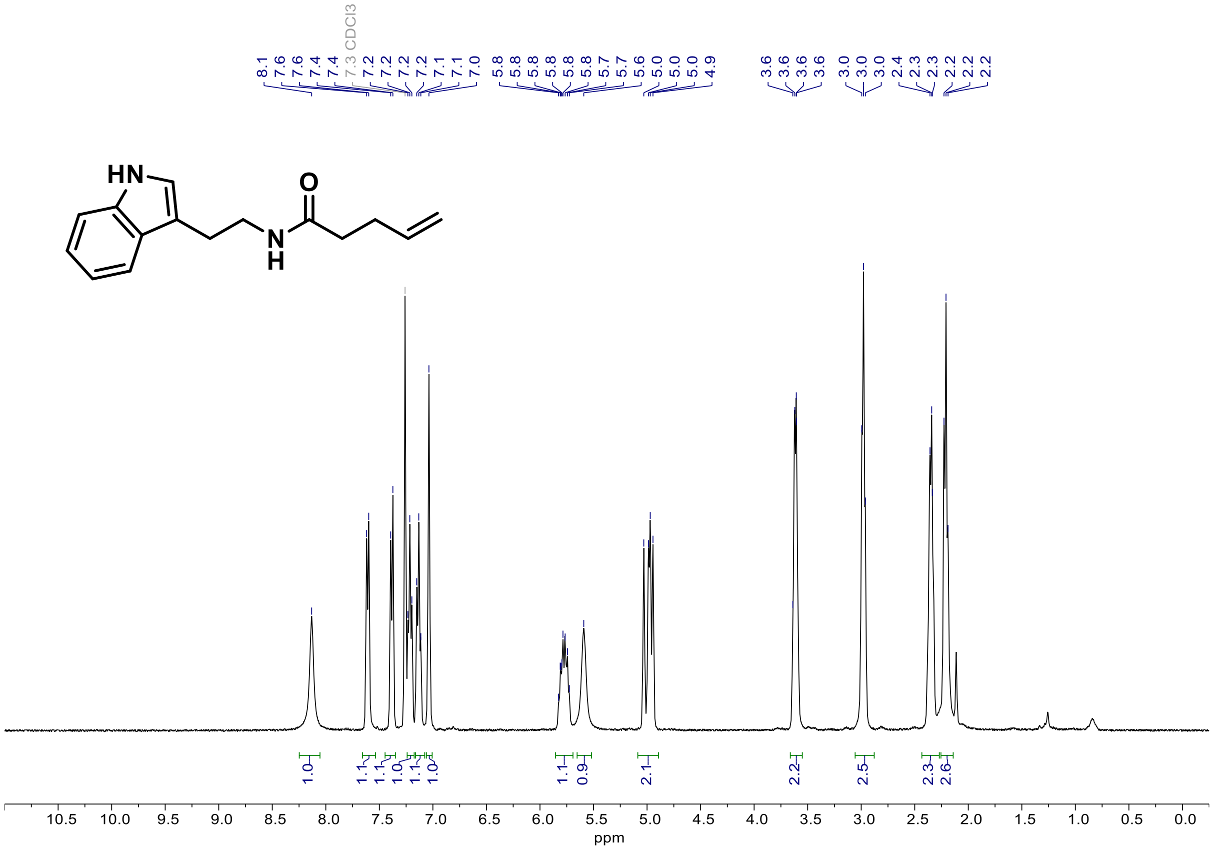

S3 – ^{13}C NMR (101 MHz, CDCl$_3$)

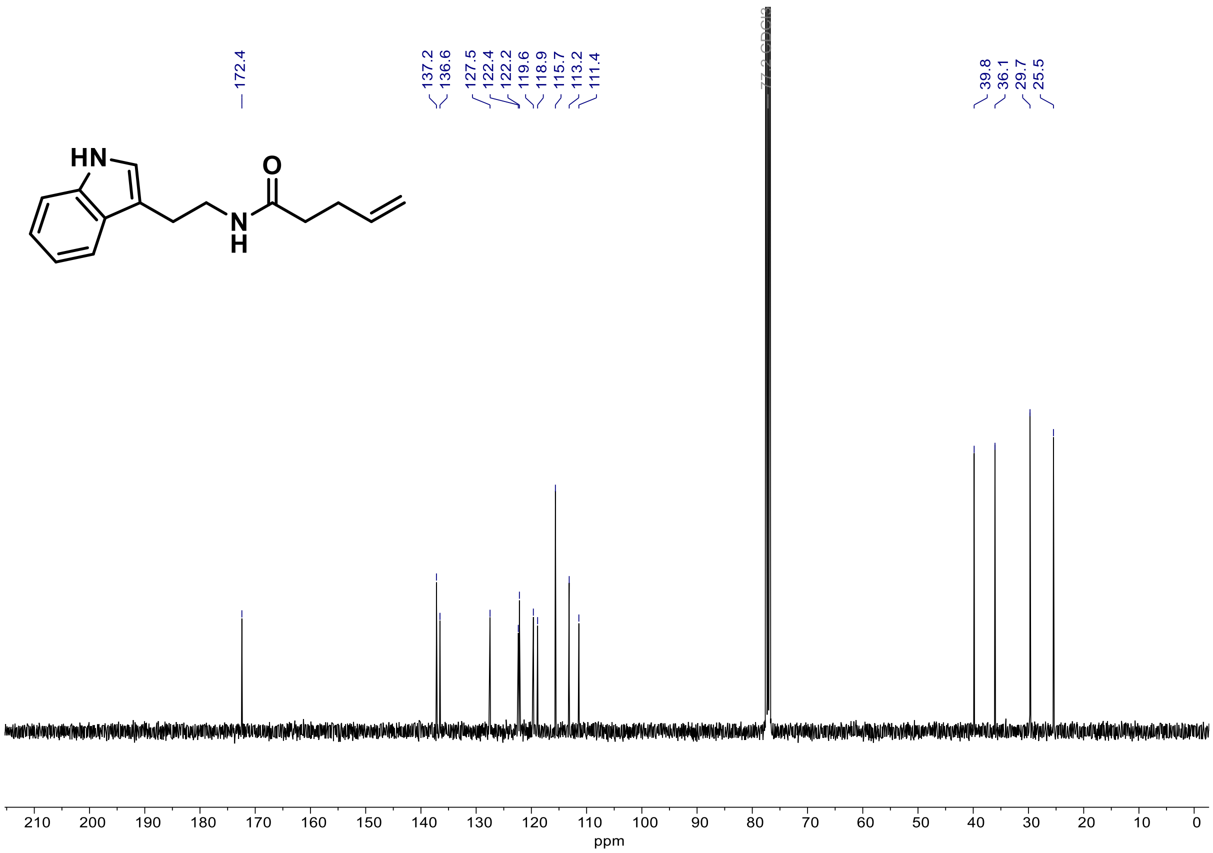

S4 – ^{1}H NMR (400 MHz, $CDCl_3$)

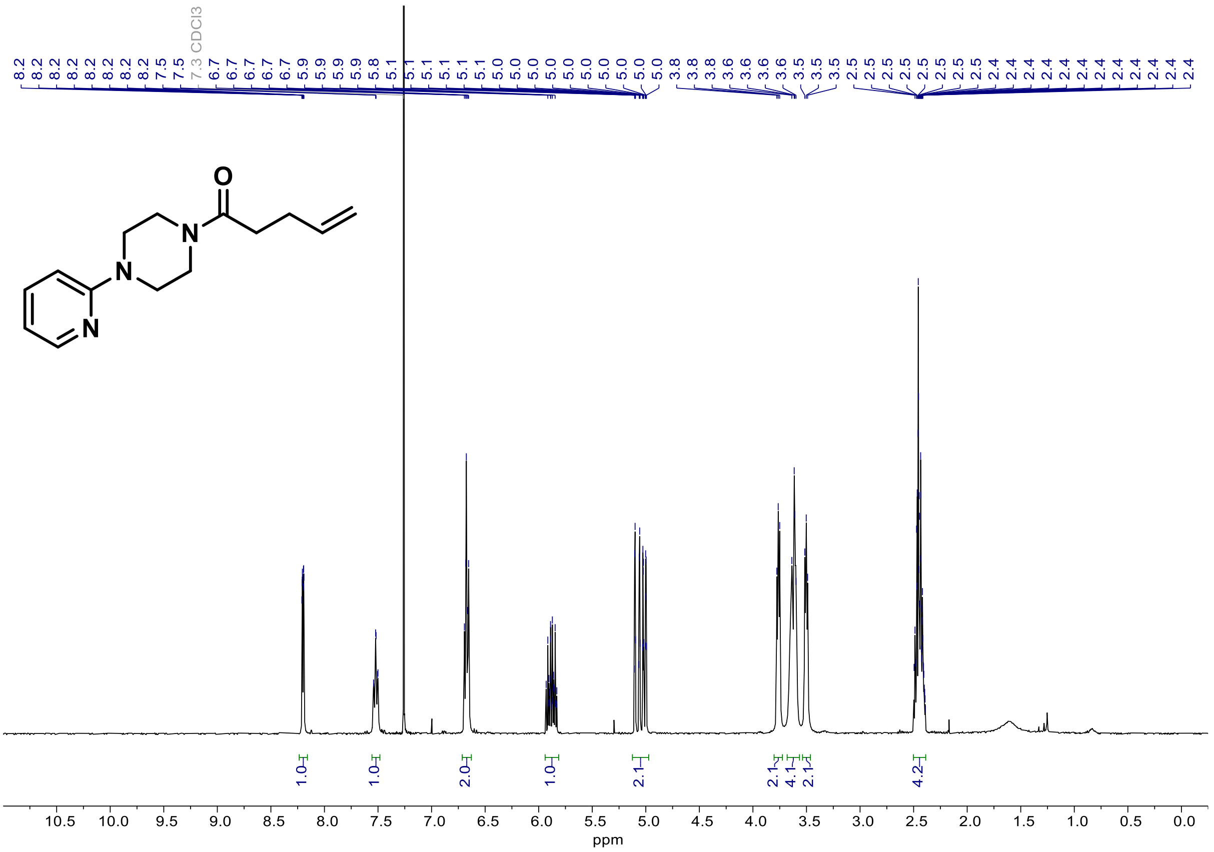

S4 – ^{13}C NMR (101 MHz, $CDCl_3$)

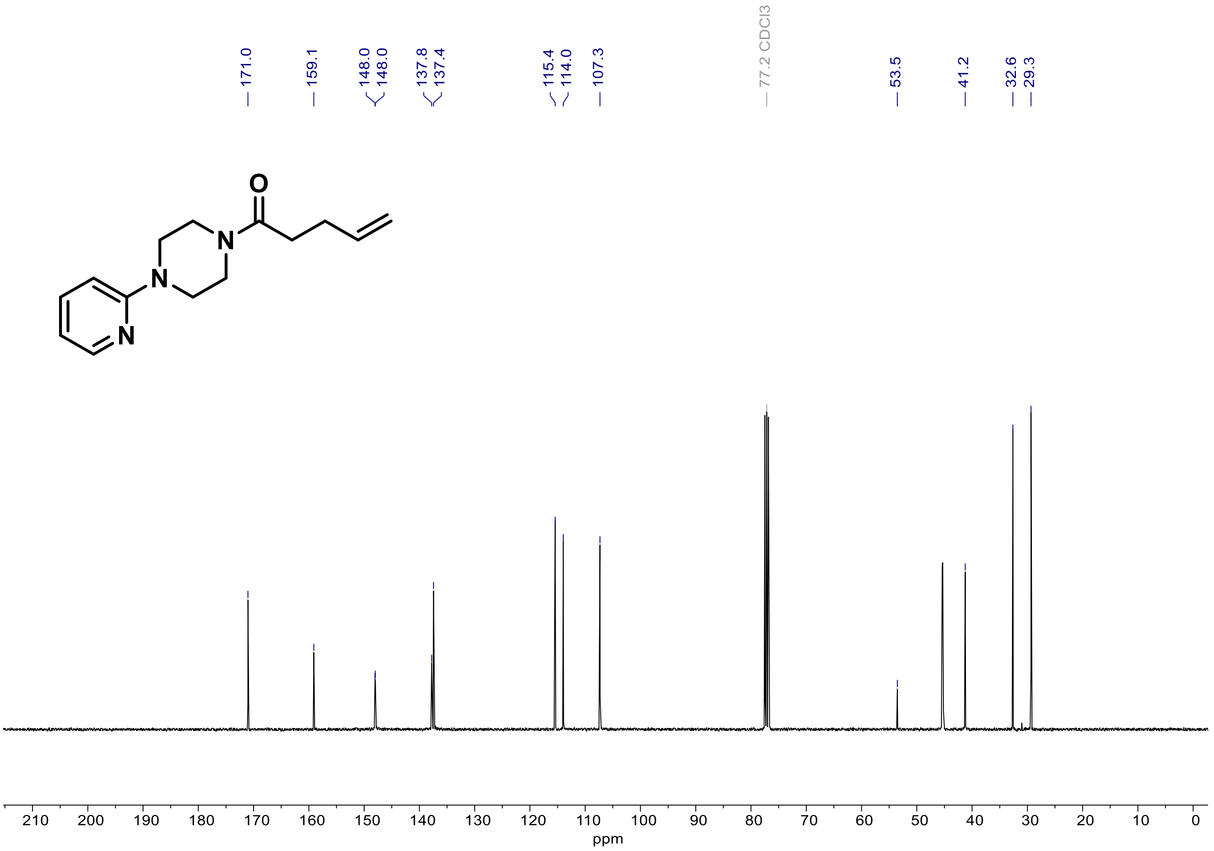

S6 – ^{1}H NMR (400 MHz, $CDCl_3$)

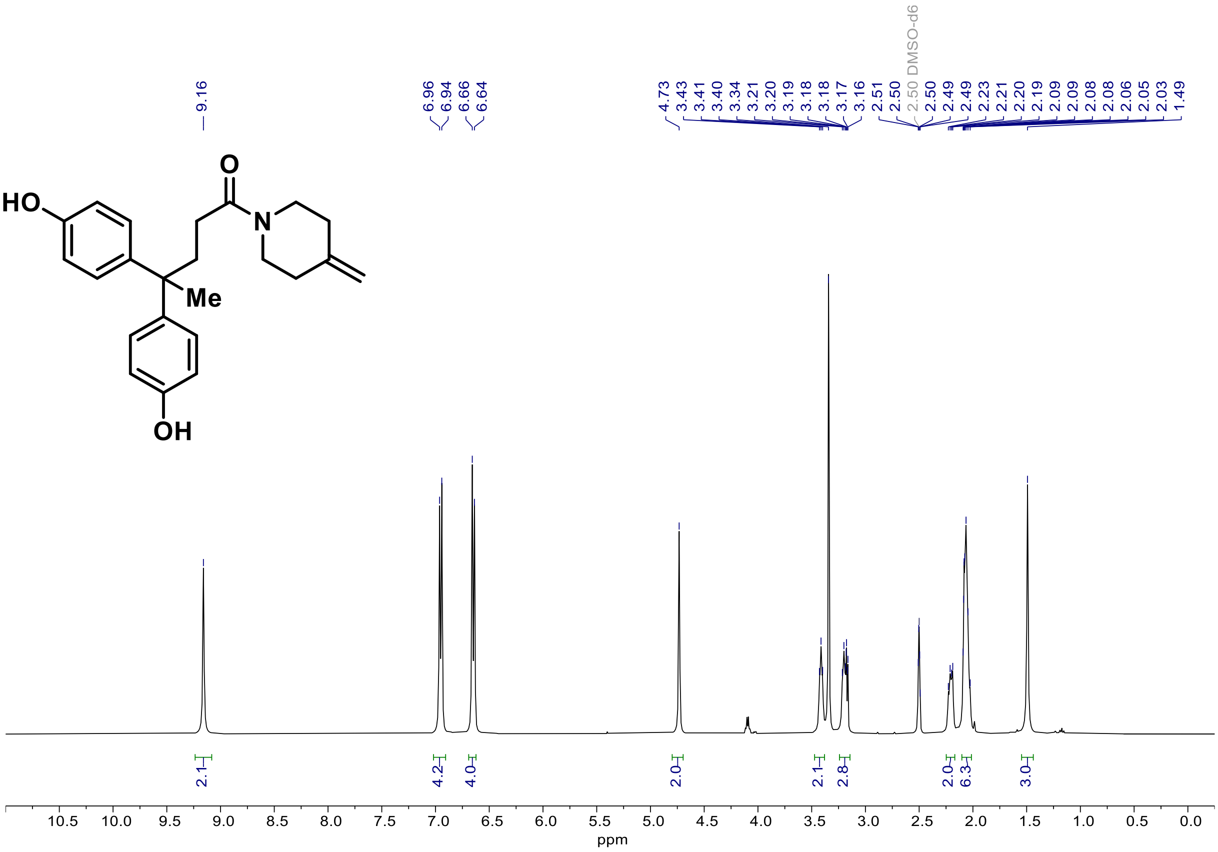

S6 – ^{13}C NMR (101 MHz, $CDCl_3$)

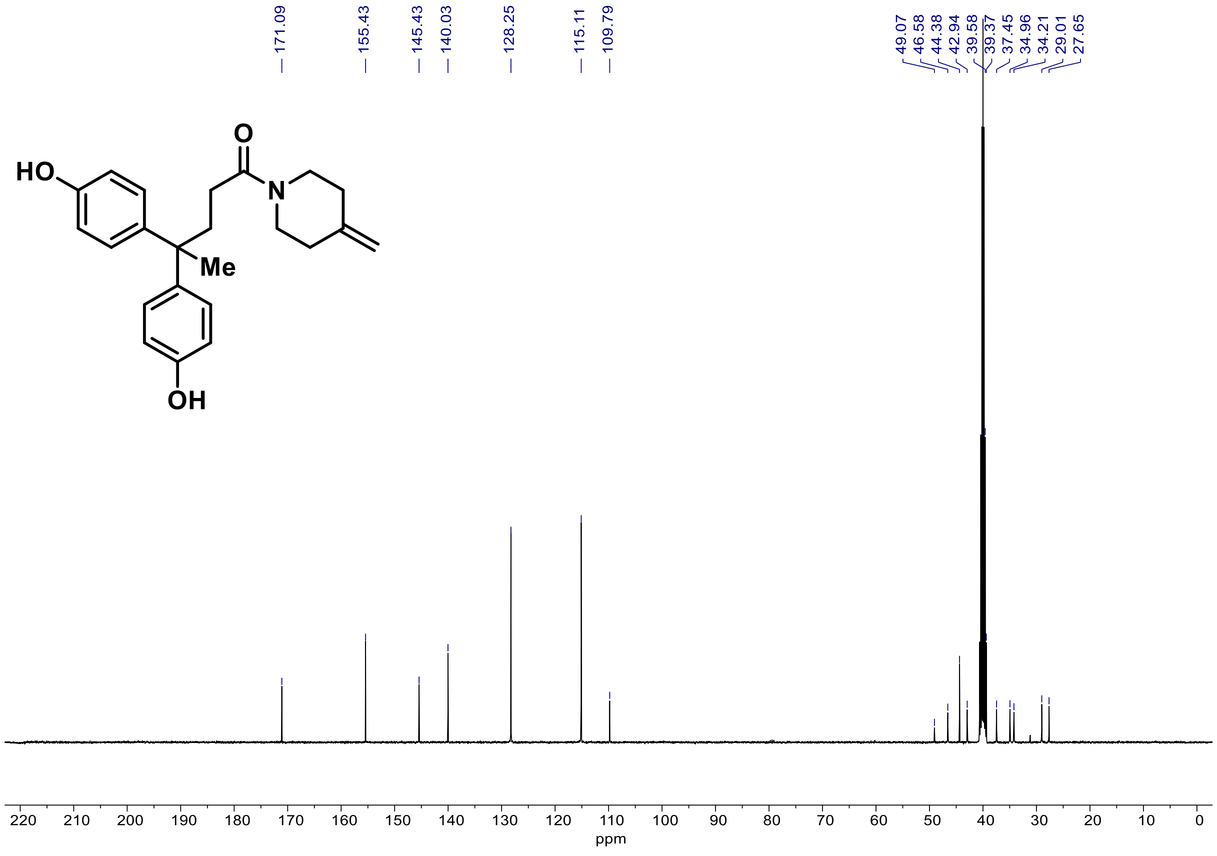

S7 – ^{1}H NMR (400 MHz, $CDCl_3$)

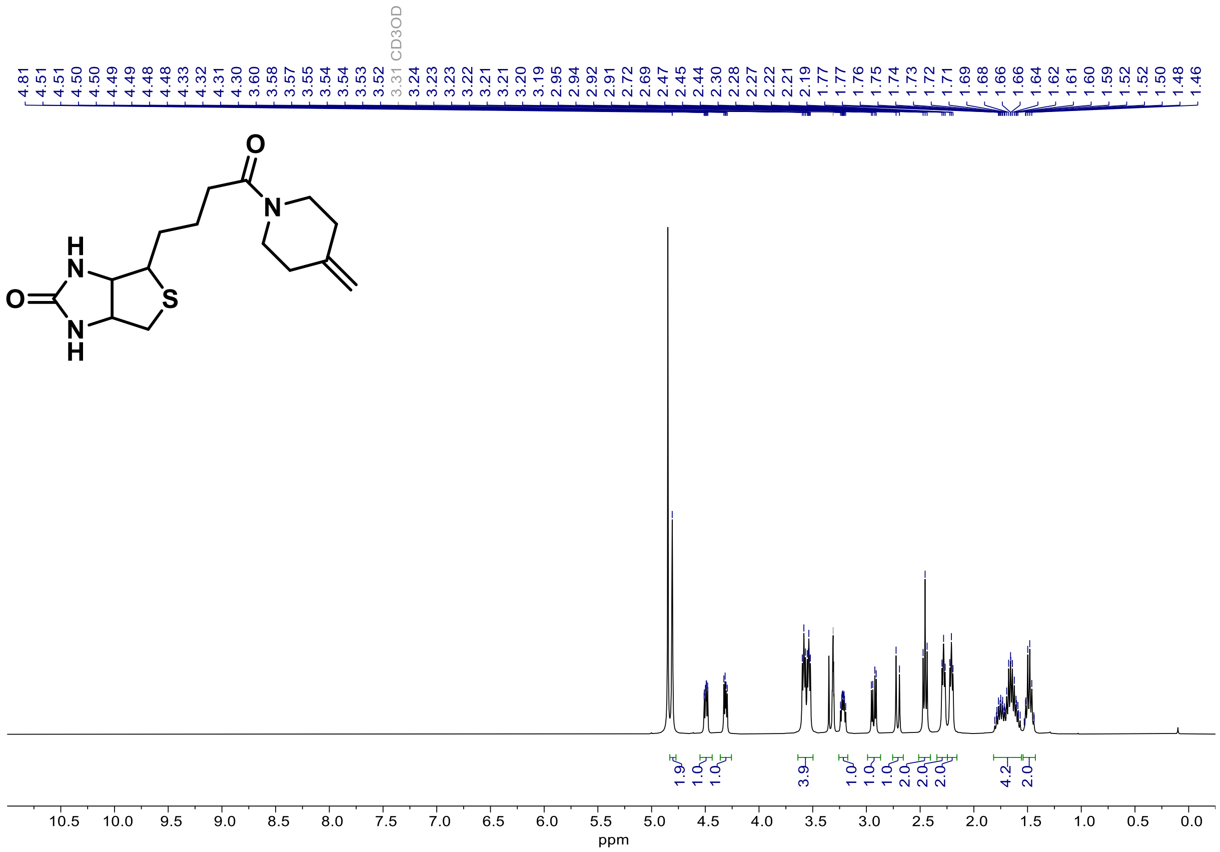

S7 – ^{13}C NMR (101 MHz, $CDCl_3$)

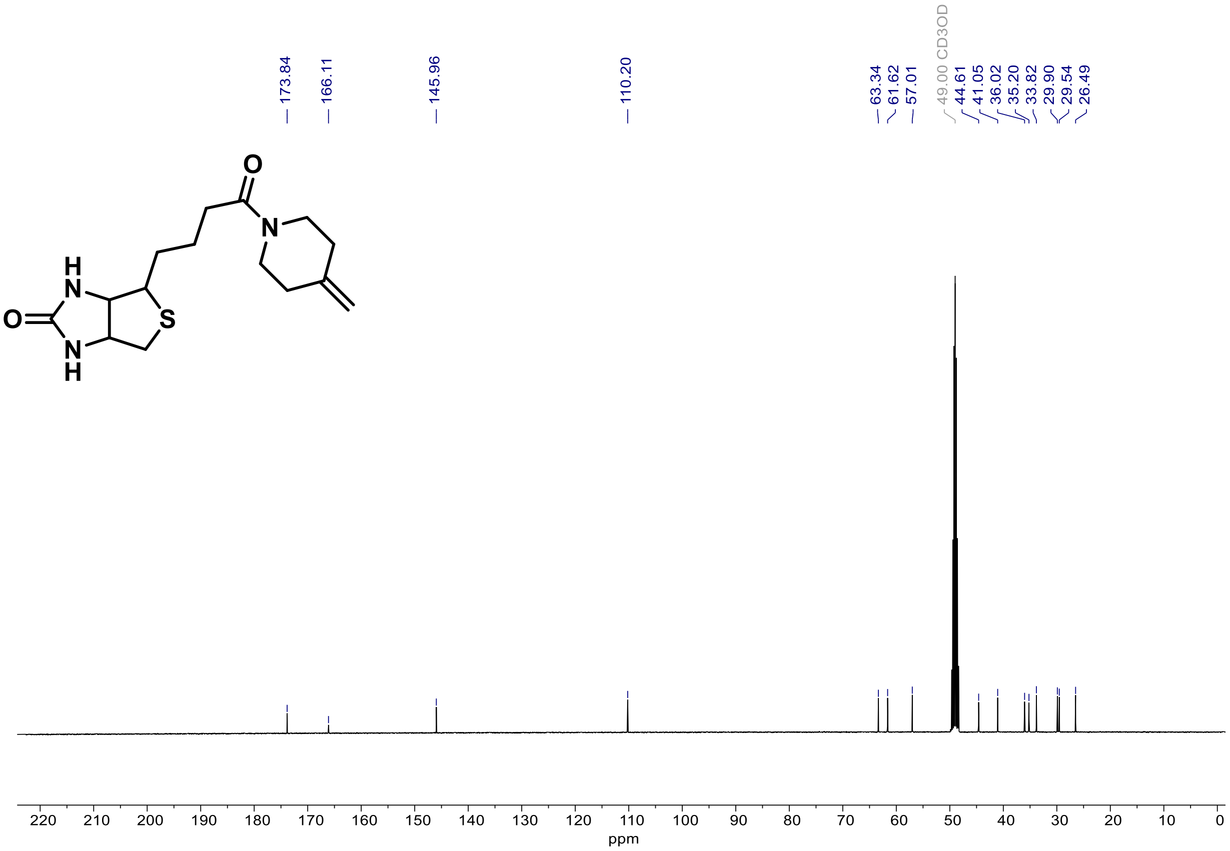

S11 – ^{1}H NMR (400 MHz, $CDCl_3$)

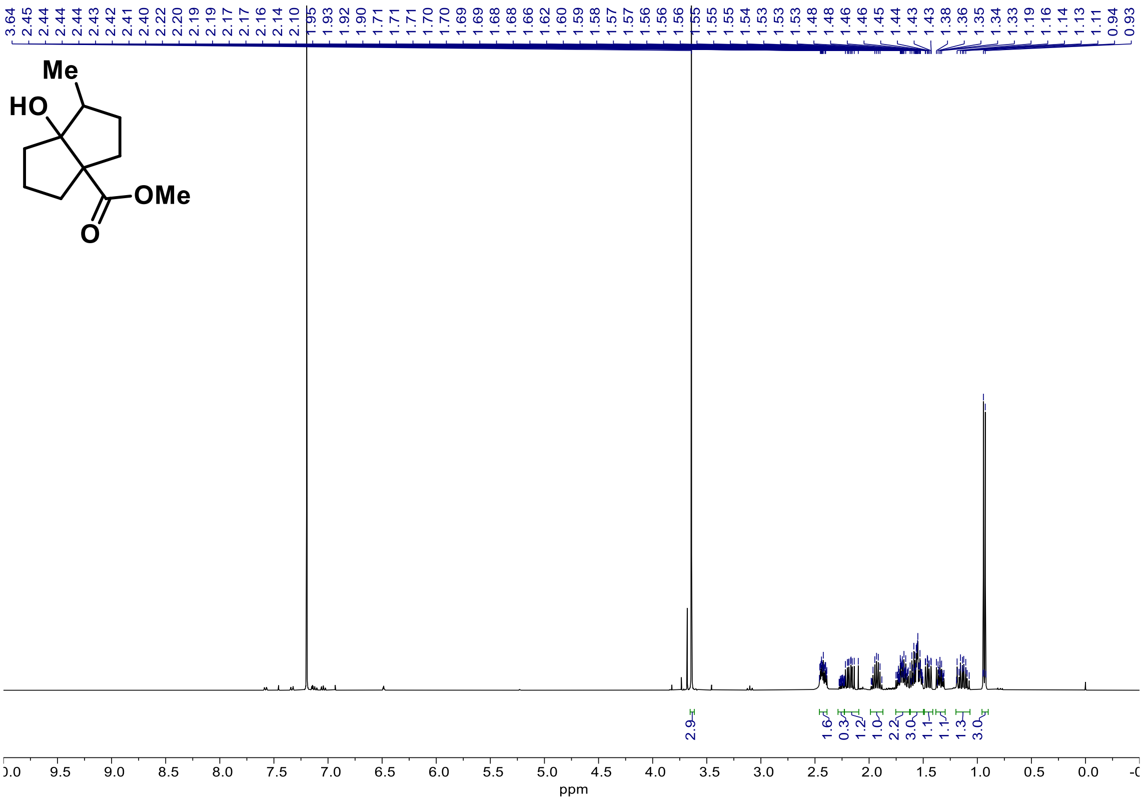

S11 – ^{13}C NMR (101 MHz, $CDCl_3$)

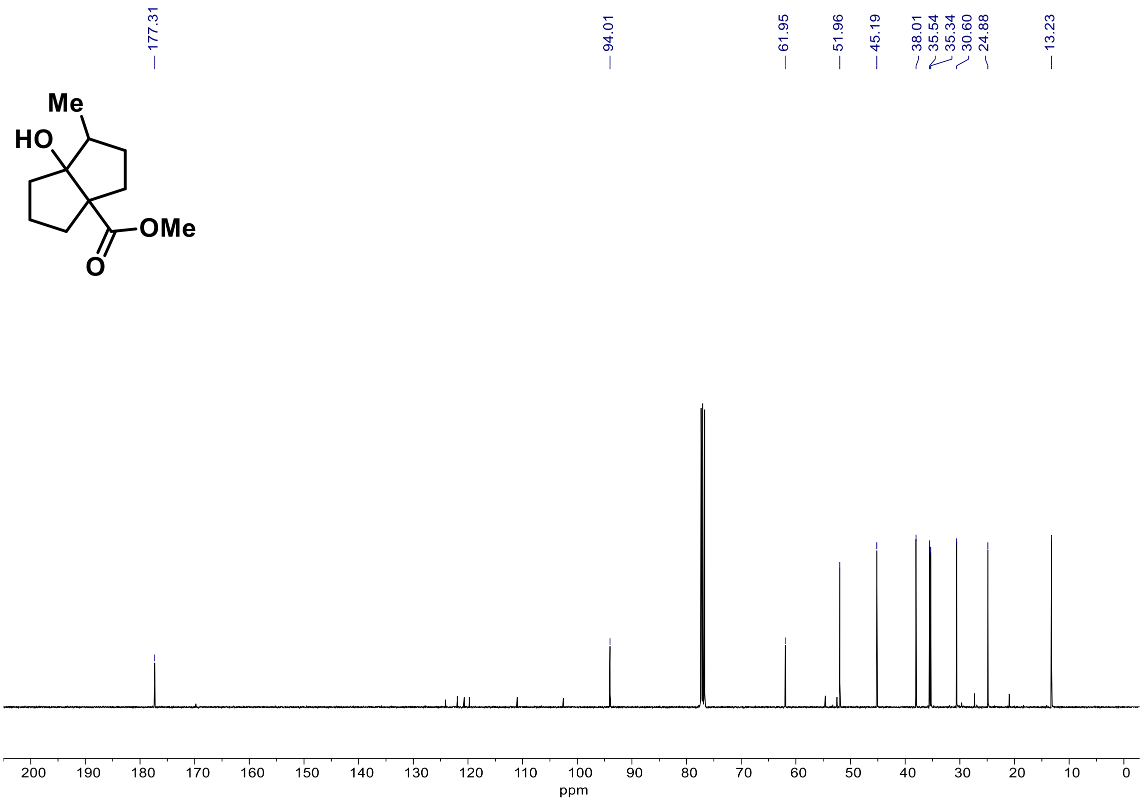

S12 – ^{1}H NMR (400 MHz, $CDCl_3$)

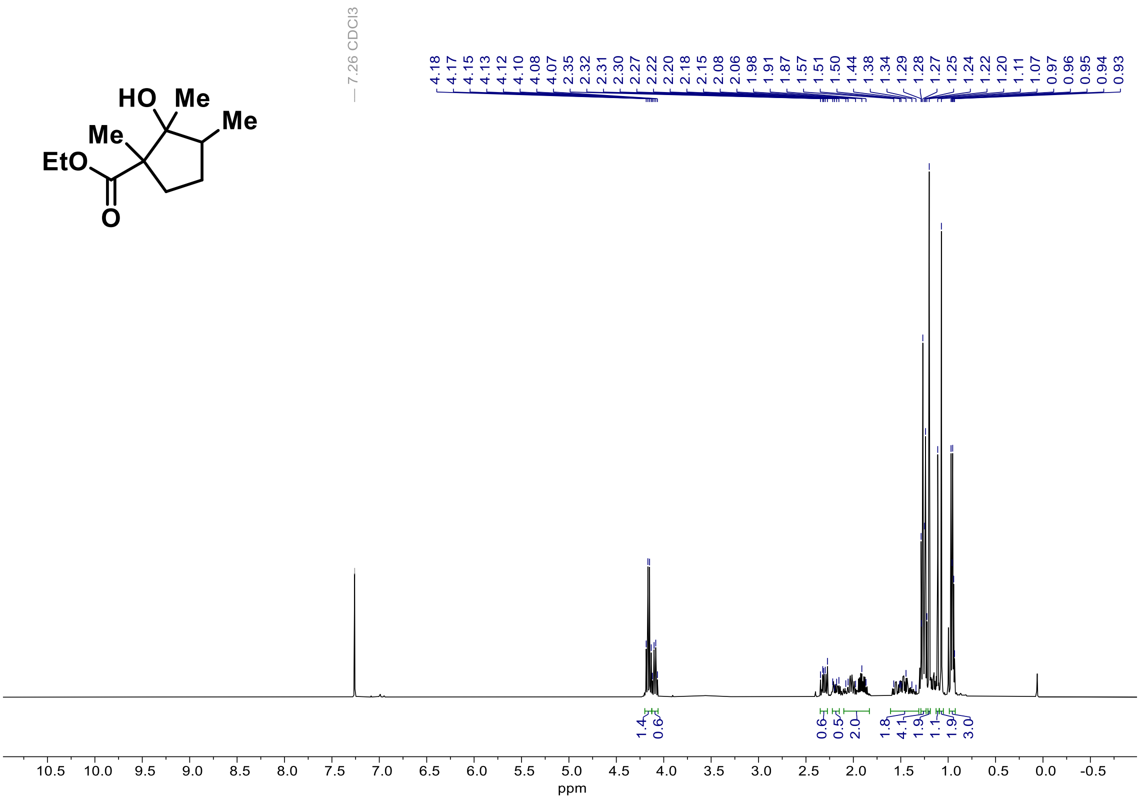

S12 – ^{13}C NMR (101 MHz, $CDCl_3$)

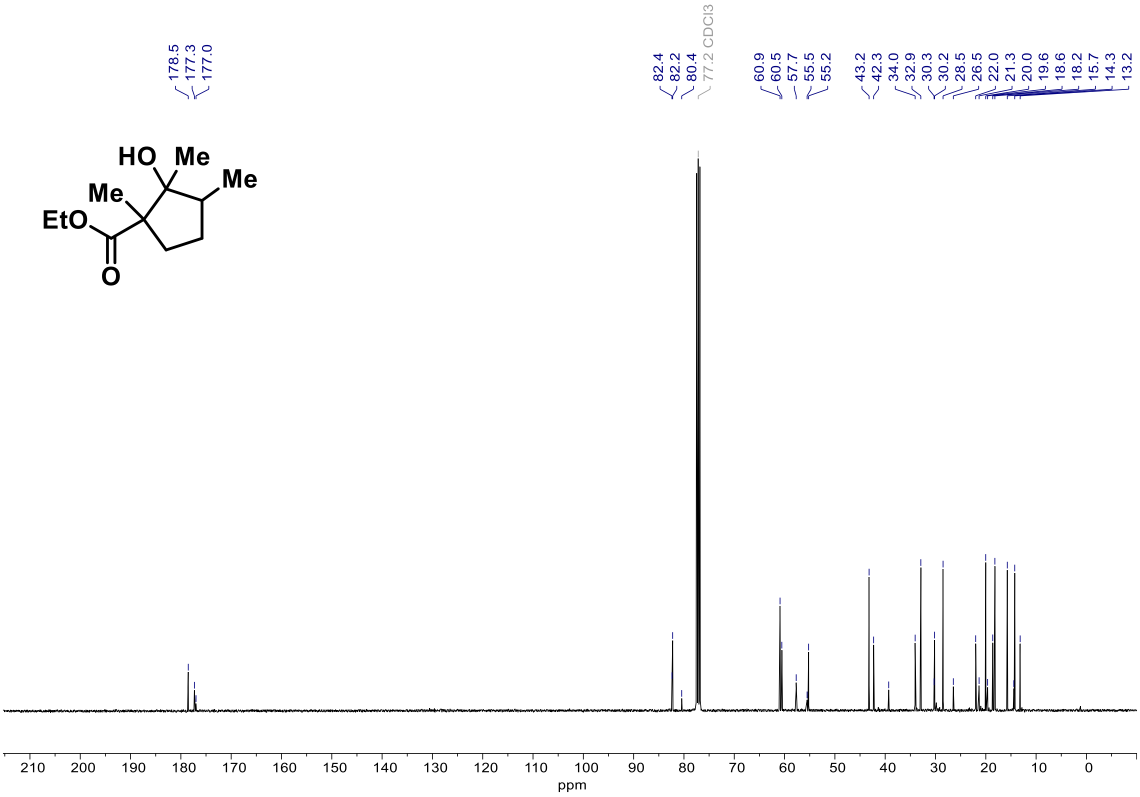

S13 – ^{1}H NMR (400 MHz, $CDCl_3$)

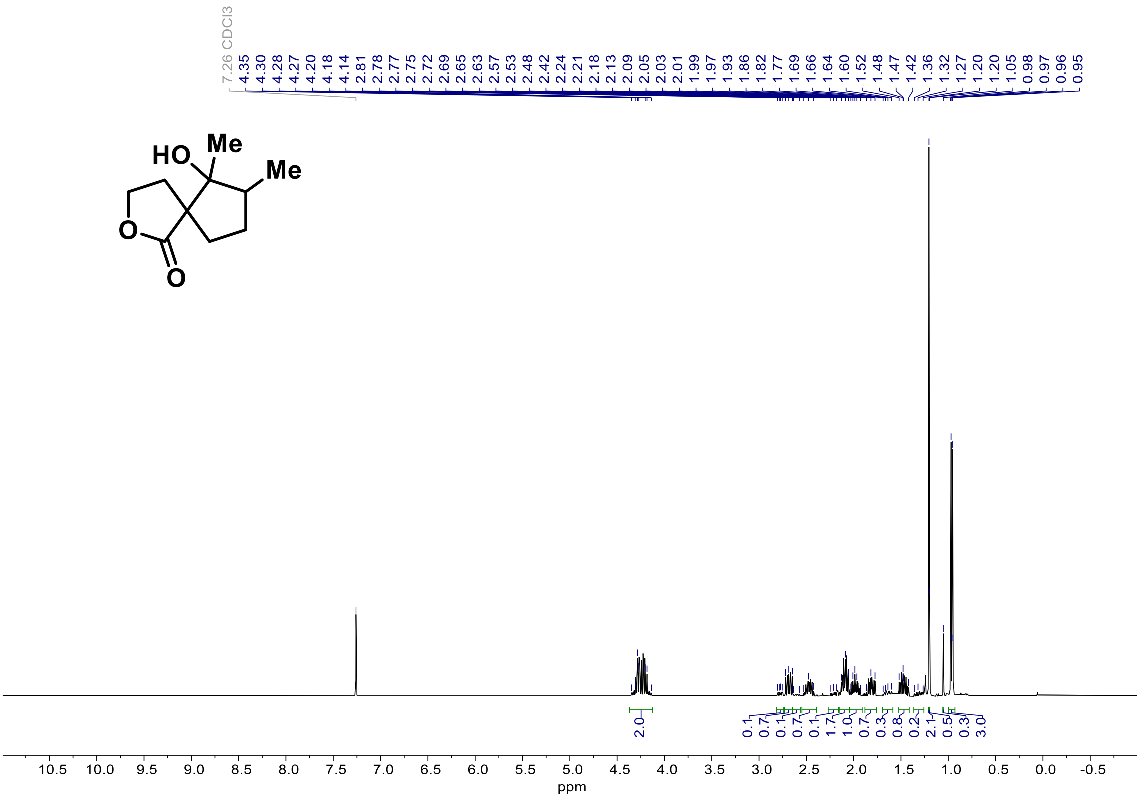

S13 – ^{13}C NMR (101 MHz, $CDCl_3$)

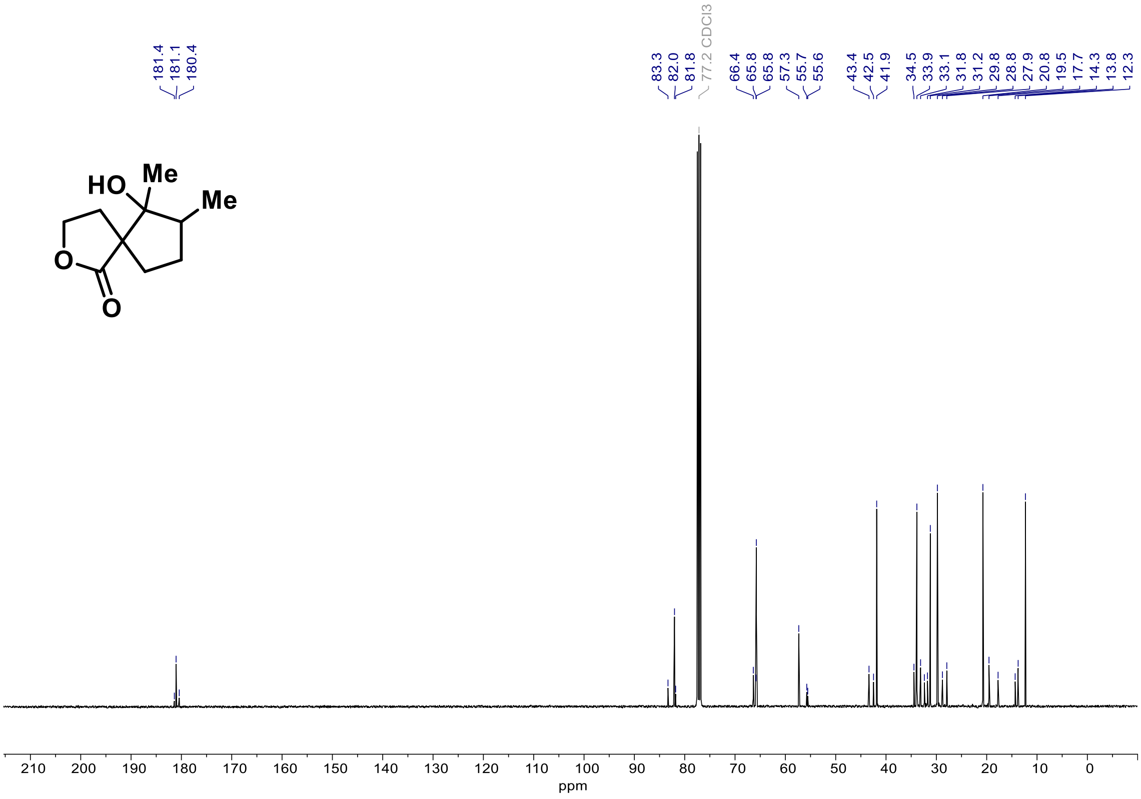

S14 – ^{1}H NMR (400 MHz, $CDCl_3$)

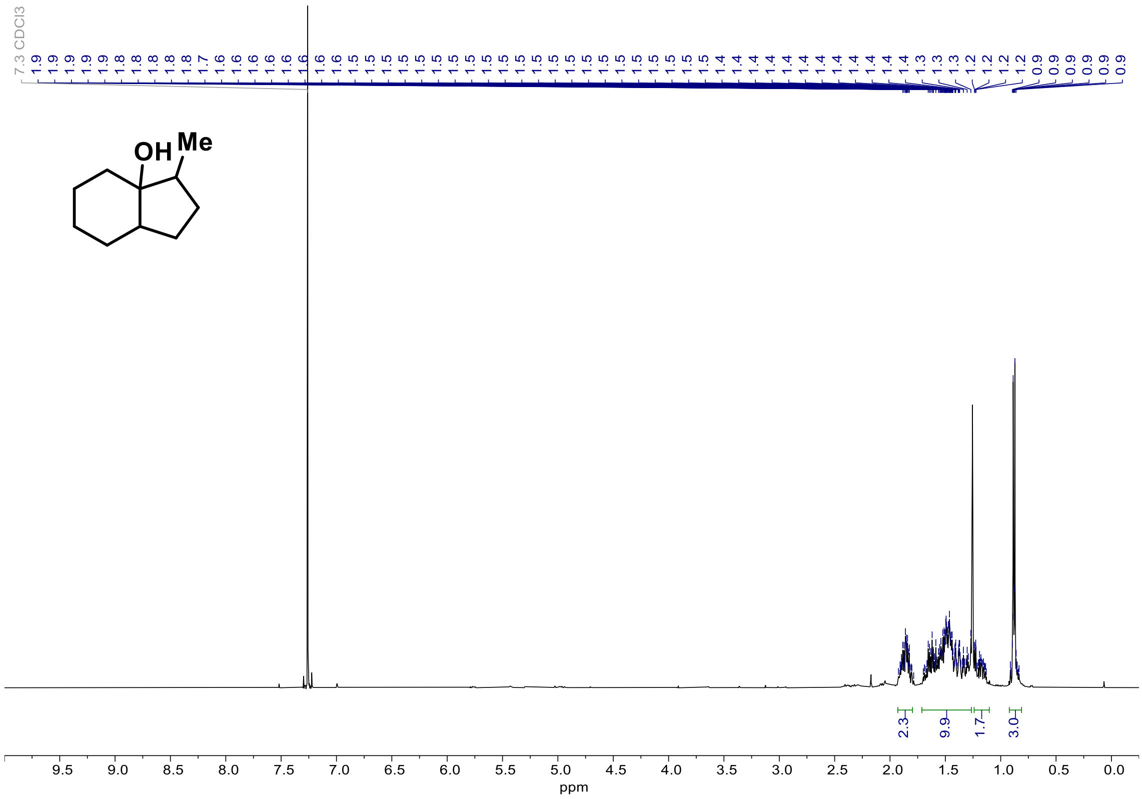

S14 – ^{13}C NMR (101 MHz, $CDCl_3$)

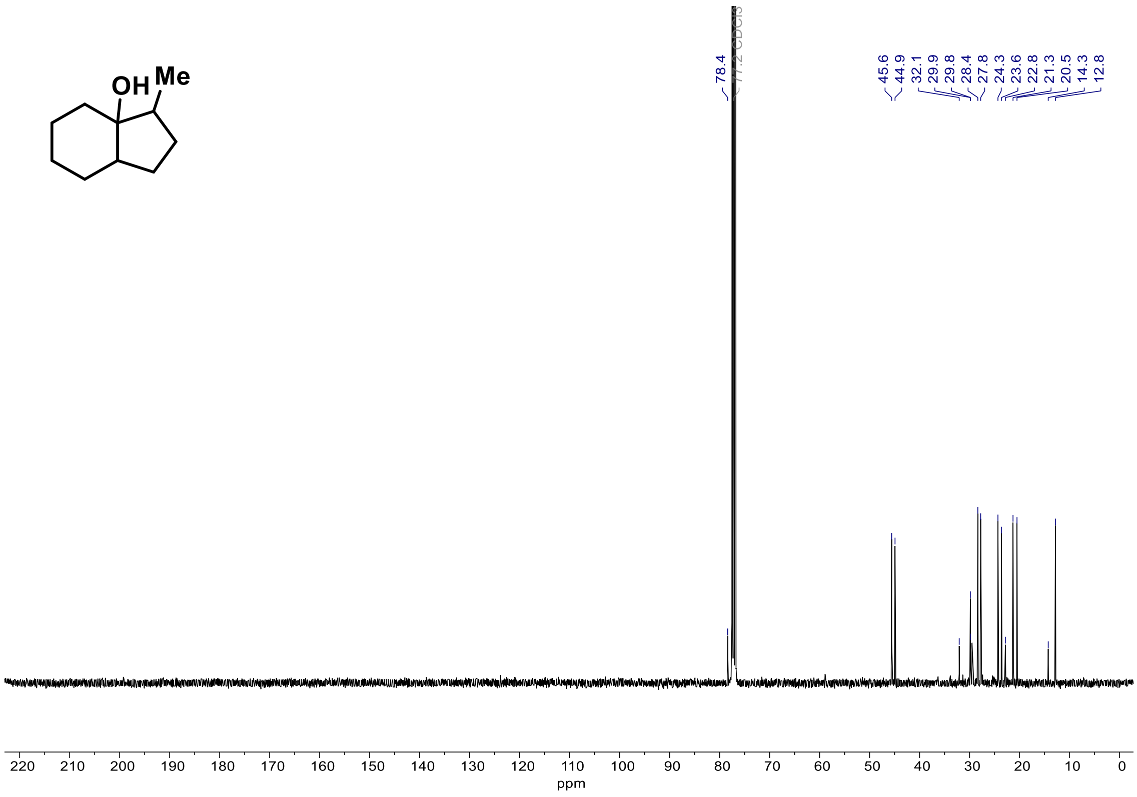

S16 – ^{1}H NMR (400 MHz, $CDCl_3$)

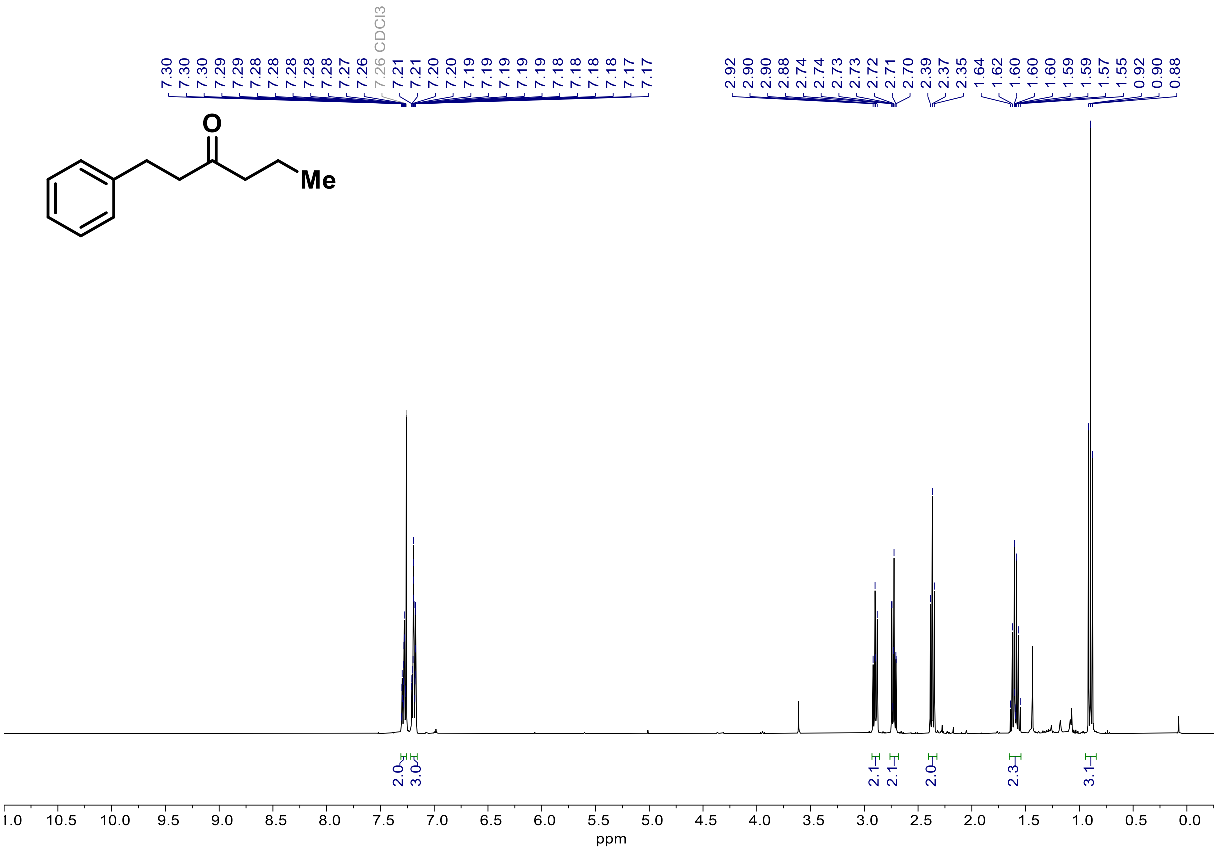

S16 – ^{13}C NMR (101 MHz, $CDCl_3$)

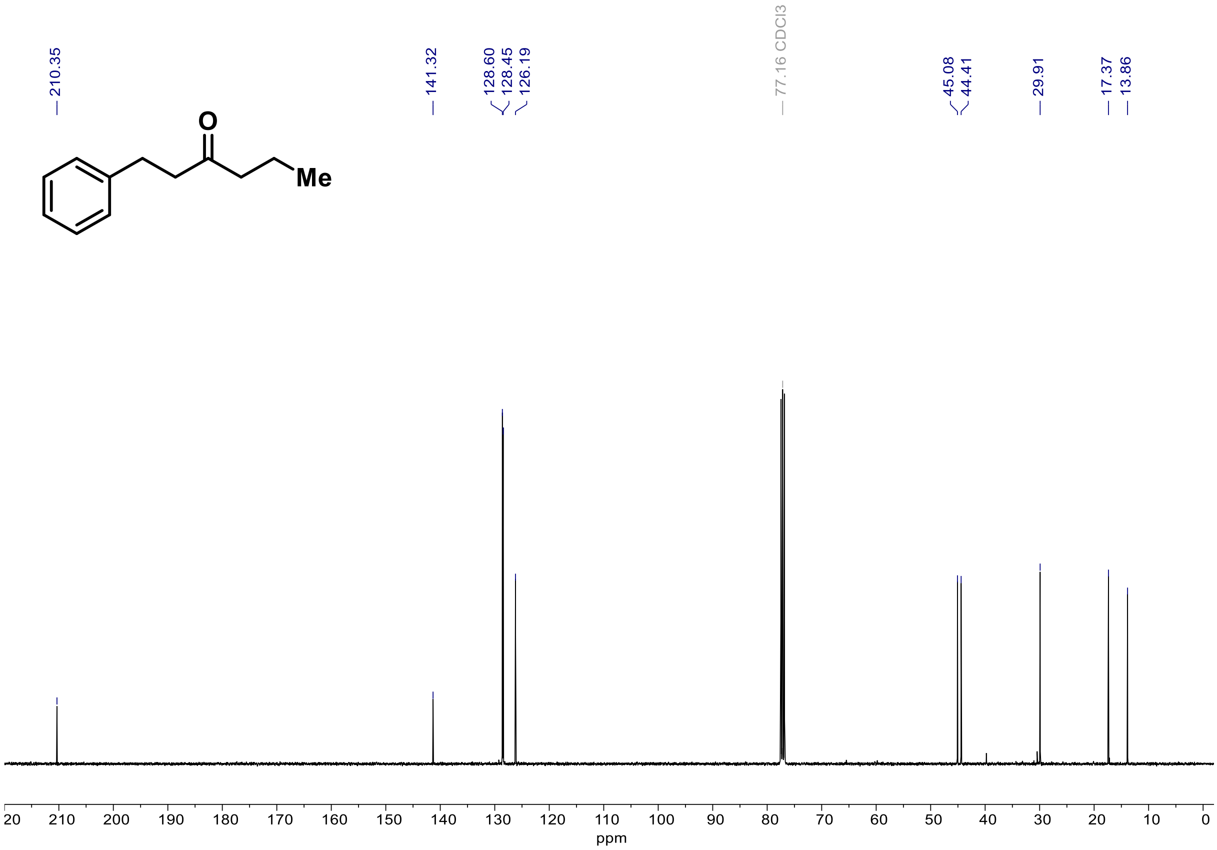

S17 – ^{1}H NMR (400 MHz, $CDCl_3$)

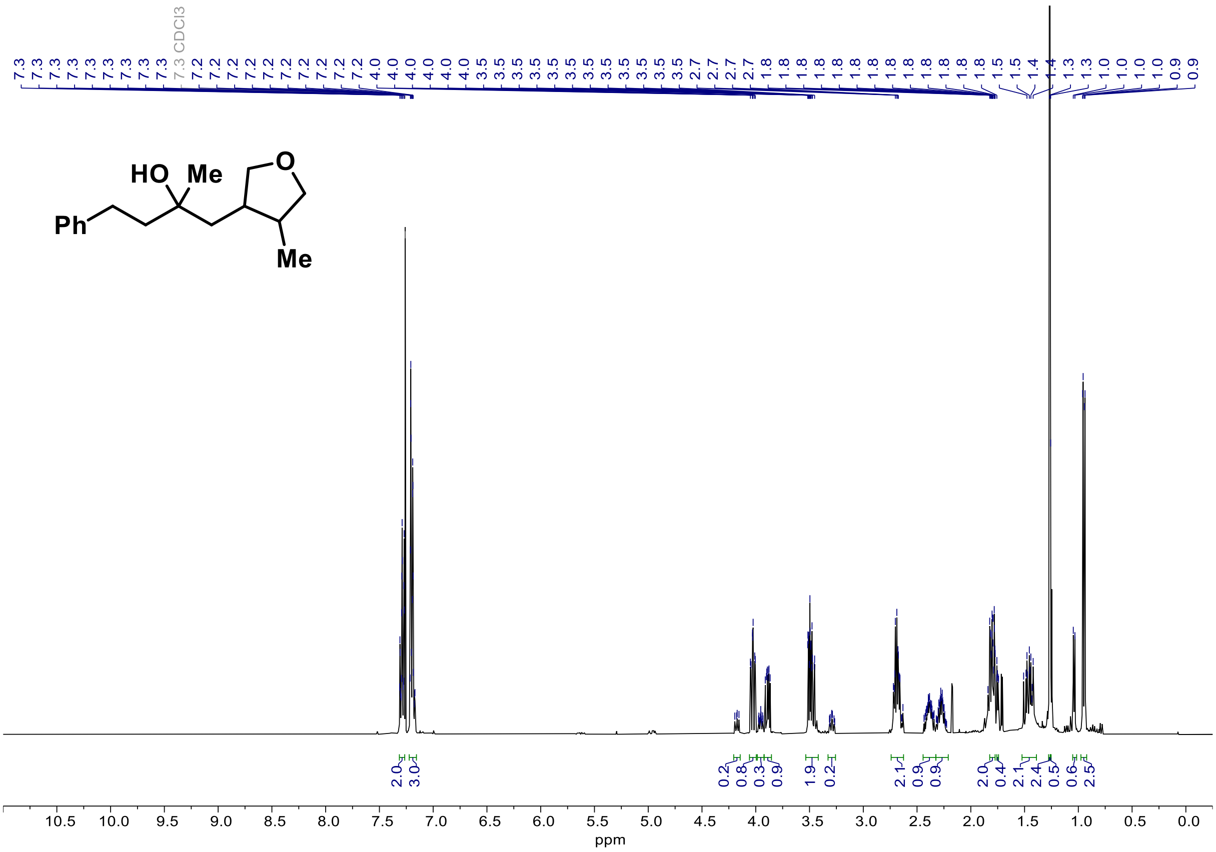

S17 – ^{13}C NMR (101 MHz, $CDCl_3$)

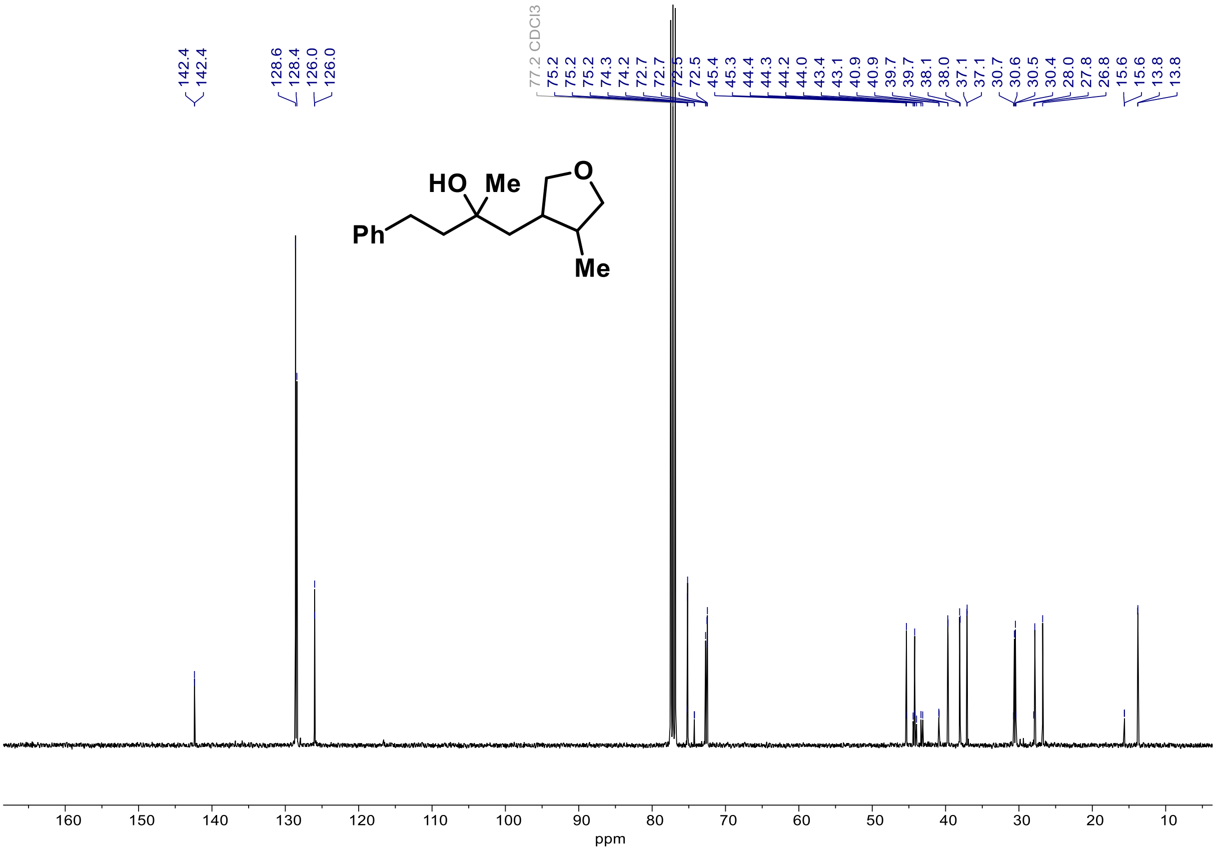

S18-a – ^{1}H NMR (400 MHz, $CDCl_3$)

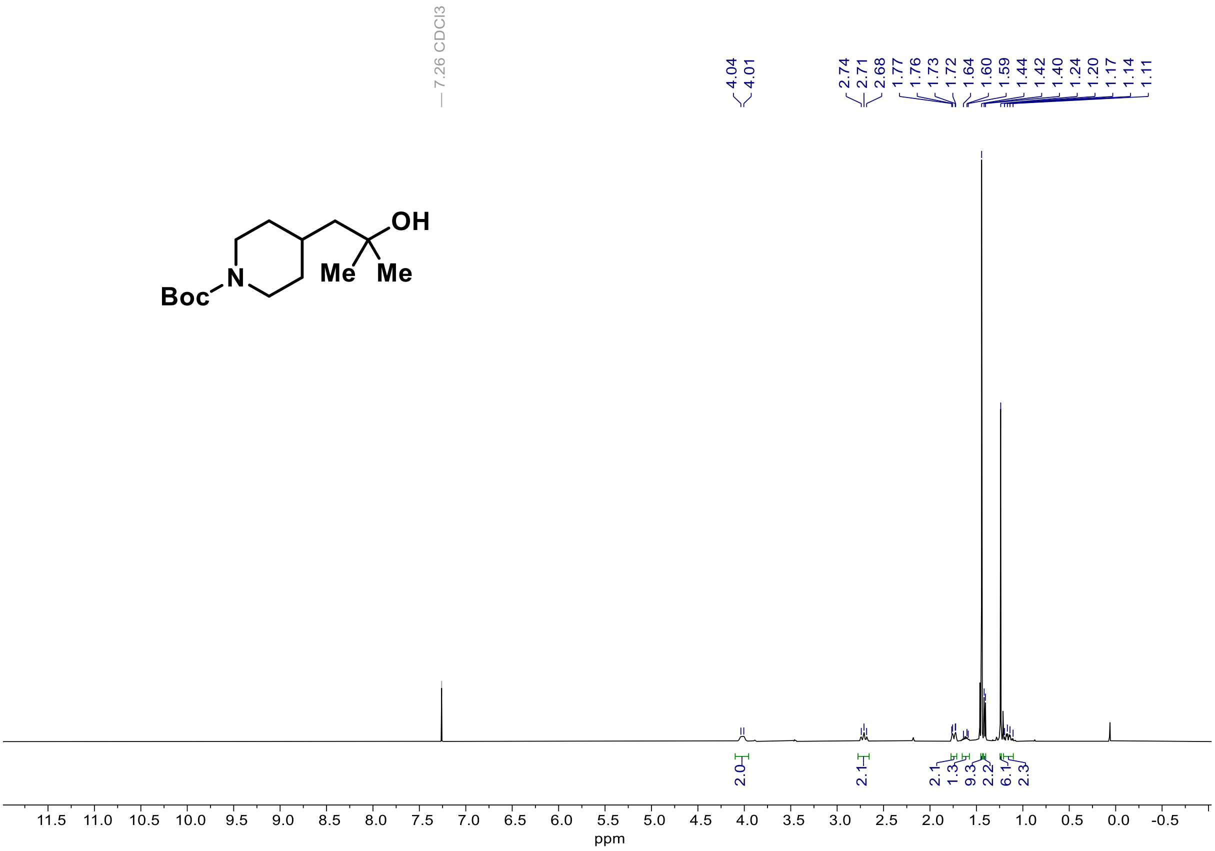

S18-a – ^{13}C NMR (101 MHz, $CDCl_3$)

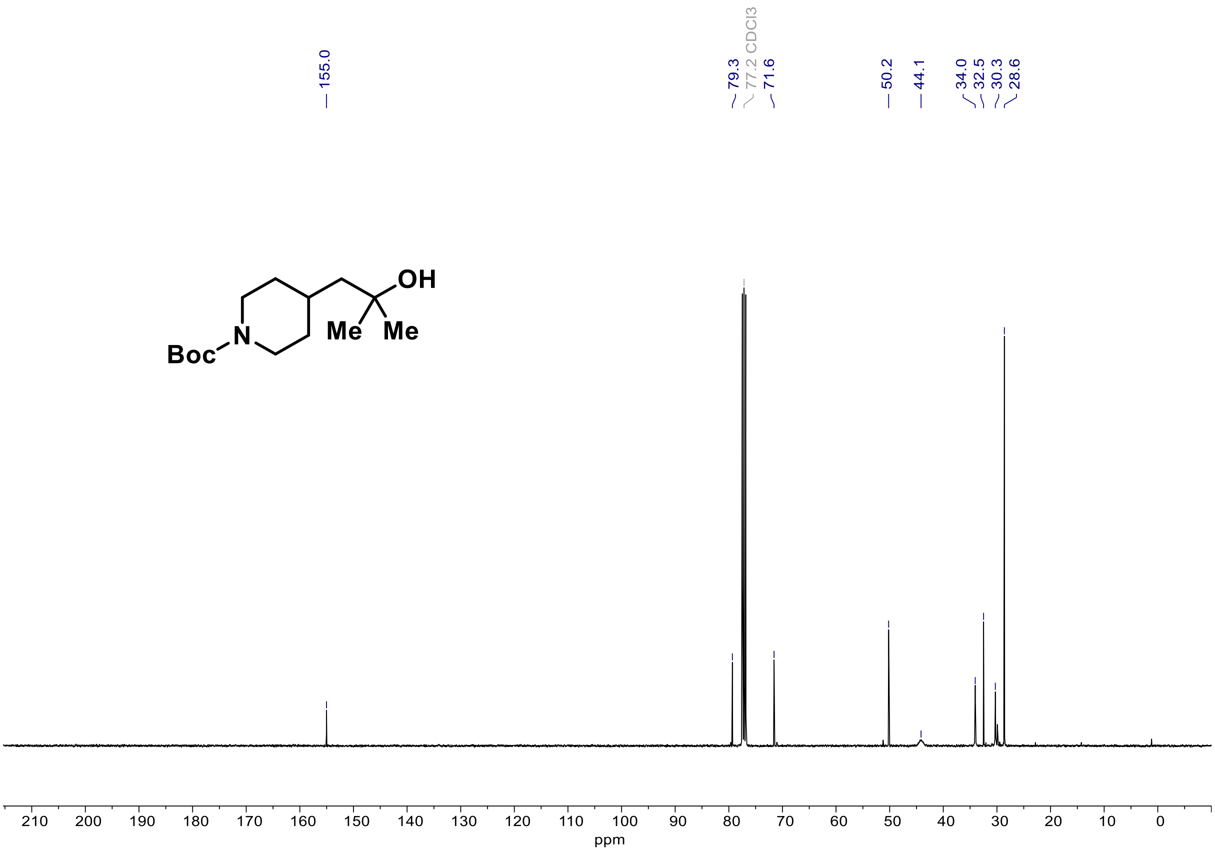

S18-b – ^{1}H NMR (400 MHz, $CDCl_3$)

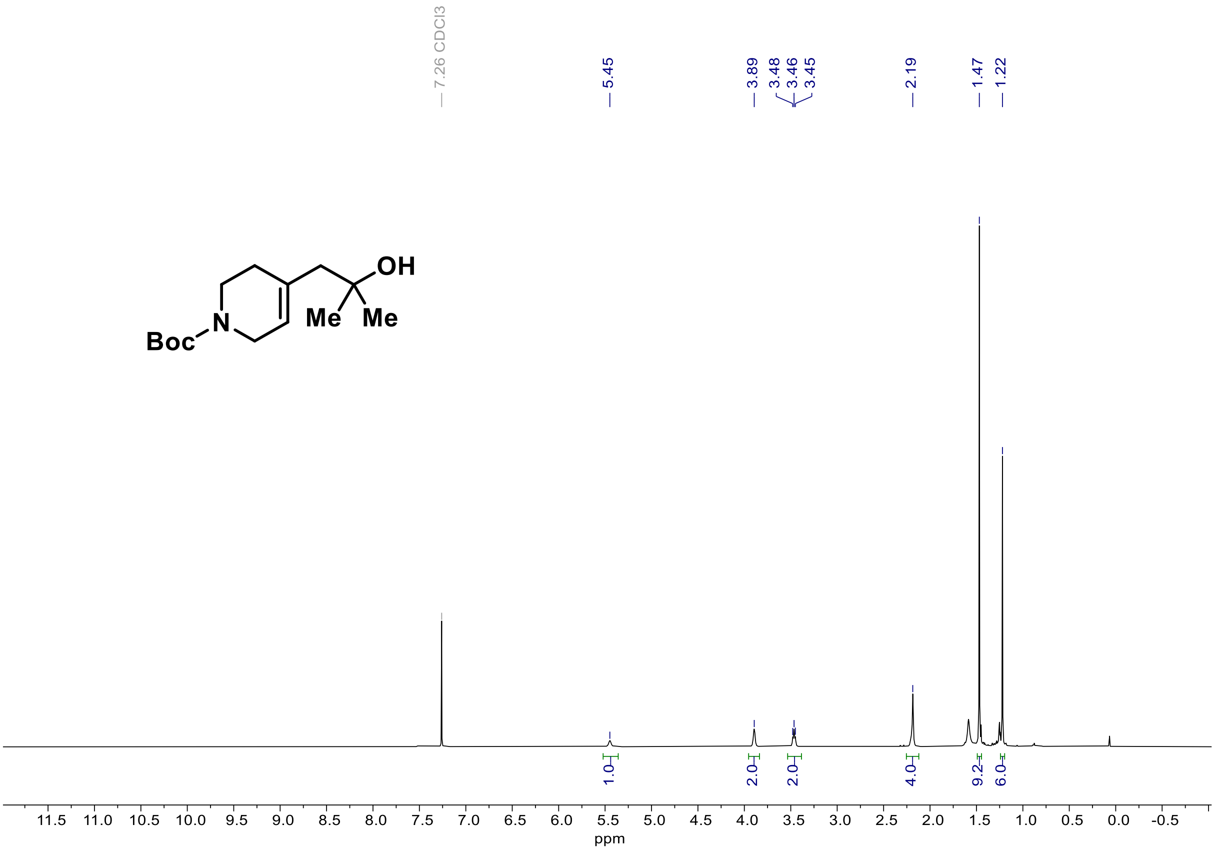

S18-b – ^{13}C NMR (101 MHz, $CDCl_3$)

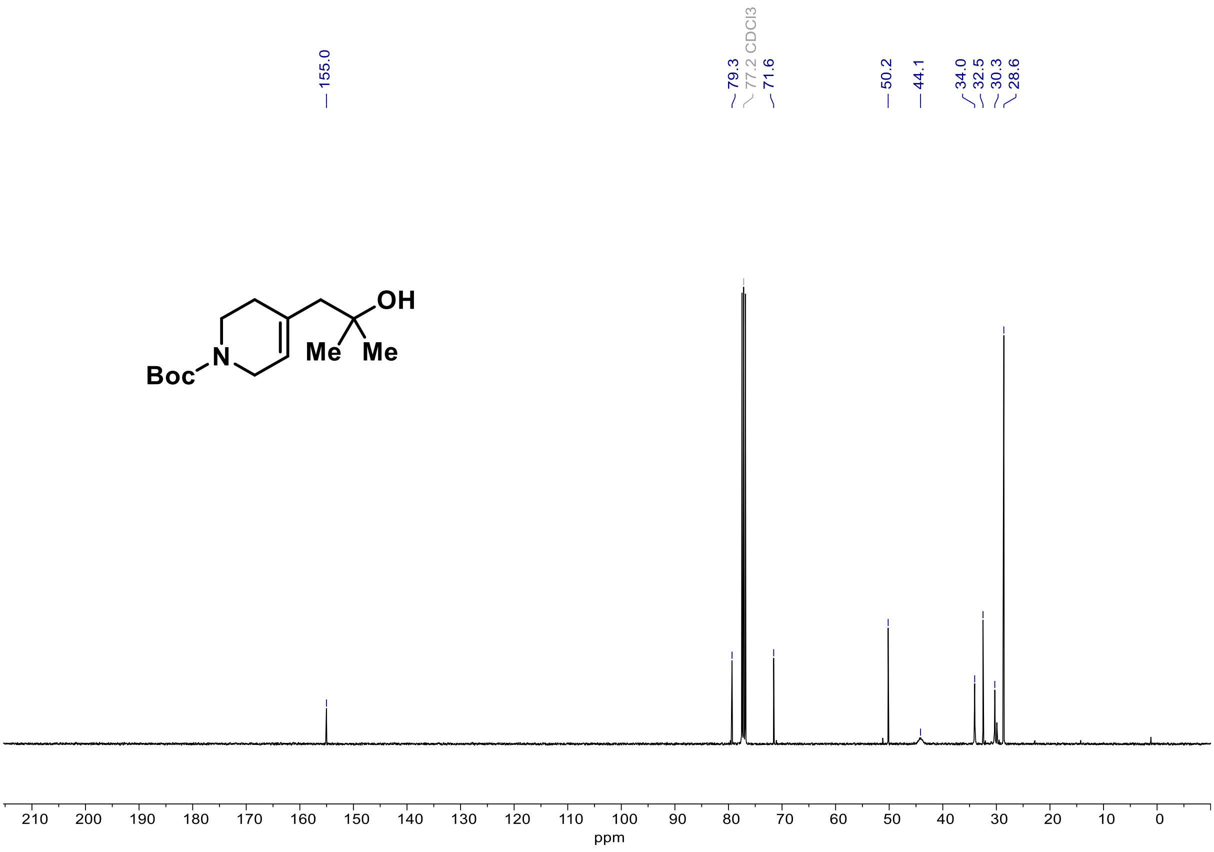

S19 – ^{1}H NMR (400 MHz, $CDCl_3$)

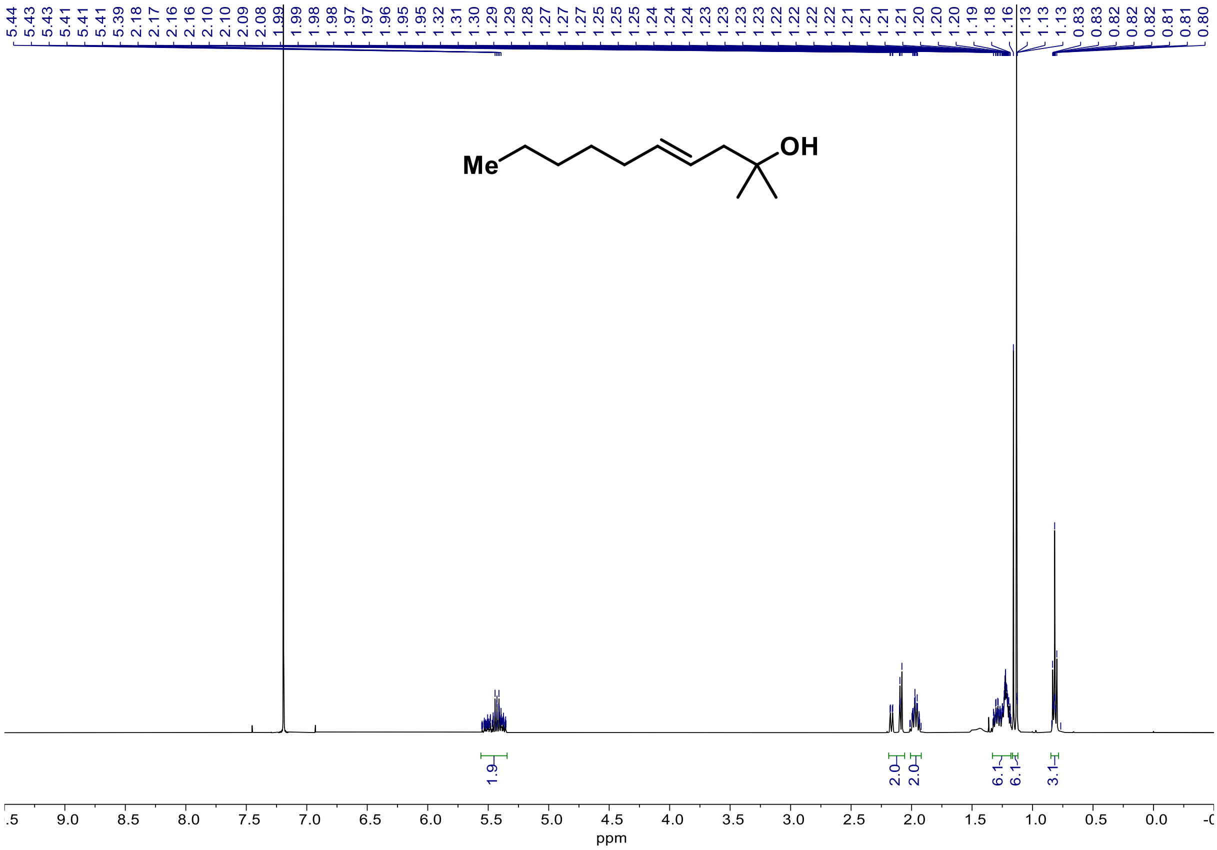

S19 – ^{13}C NMR (101 MHz, $CDCl_3$)

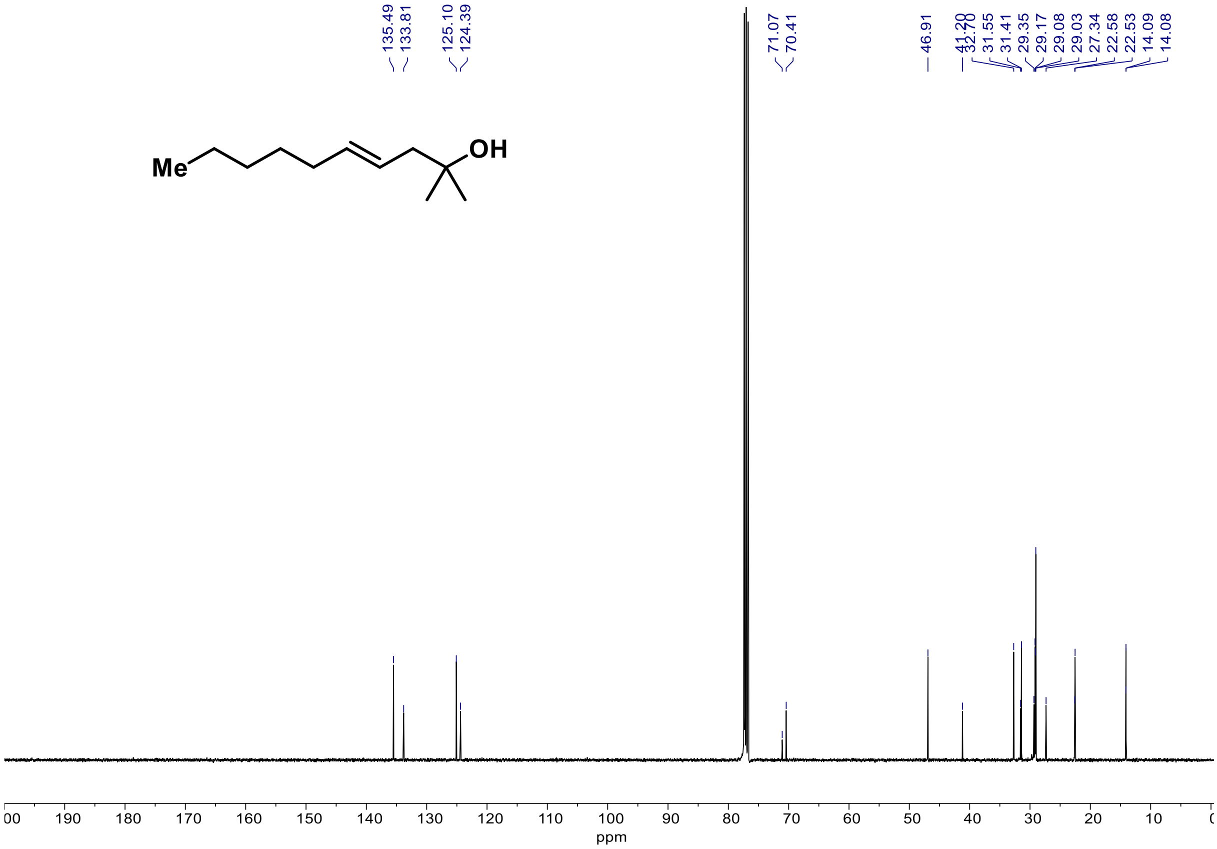

Supplement: Supplementary file 1 [file ja6c10799_si_001.pdf]
